# Supplementary material for: Malaria inflammation by xanthine oxidase‐produced reactive oxygen species
Source: EMBO Mol Med. 2019 Jul 2;11(8):e9903. doi: 10.15252/emmm.201809903 (PMC6685105; doi:10.15252/emmm.201809903)
Supplement: Supplementary file 1 — Appendix [file EMMM-11-e9903-s001.pdf]

Malaria Inflammation by Xanthine Oxidase-produced Reactive Oxygen Species

Maureen C. Ty, Marisol Zuniga, Anton Götz, Sriti Kayal, Praveen K. Sahu, Akshaya Mohanty, Sanjib Mohanty, Samuel C. Wassmer and Ana Rodriguez

Appendix

Appendix Table S1. Antibodies used.

Appendix Fig. S1. Macrophages are viable after incubation with XO.

Appendix Fig. S2. Macrophages fail to secrete cytokines with increasing ratios of iRBCL.

Appendix Fig. S3. Priming with IFNa does not affect macrophage inflammatory response to iRBCL.

Appendix Fig. S4. Macrophages secrete cytokines in response to xanthine oxidase (XO) in a dose dependent manner.

Appendix Fig. S5. Addition of H_2O_2 does not prompt macrophages to secrete cytokines.

Appendix Fig. S6. siRNA transfection reduces NLRP3 mRNA expression in macrophages.

Appendix Fig. S7. NLRP3-siRNA transfection does not affect levels of chemokines**.**

Appendix Table S1. Antibodies used.

Antibodies	Source; dilution; species: reference
Anti-IL1β	Abcam 9722; 1:1000; rabbit; Master SS et al. 2008 (PMID 18407066)
Anti-β-actin	Cell Signaling 4970; 1:1000; rabbit; Gallego-Delgado et al. 2016 (PMID 27643439)

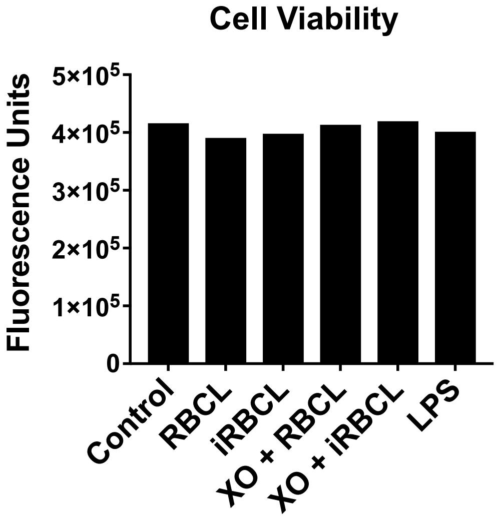

Appendix Fig. S1: Macrophages are viable after incubation with XO. Viability was determined by addition of Presto Blue (Life Technologies) 24 h after the assay. This is a representative result of two independent experiments.

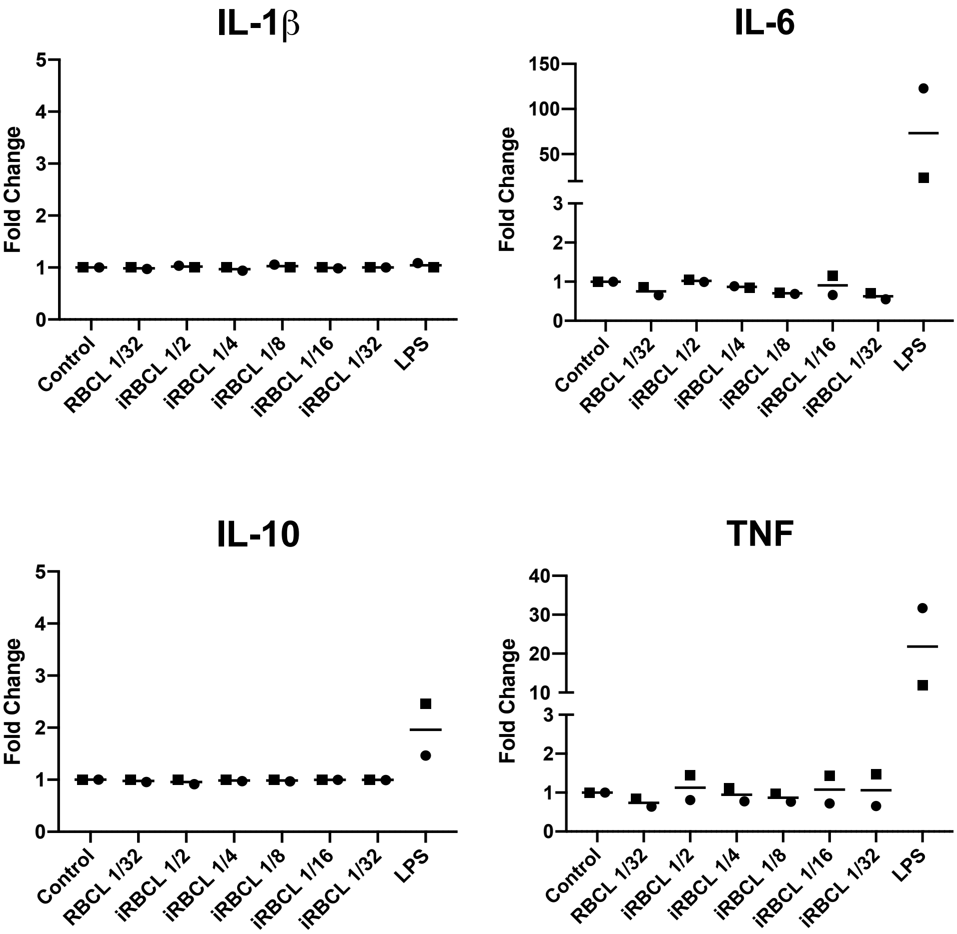

Appendix Fig. S2: Macrophages fail to secrete cytokines with increasing ratios of iRBCL. Cytokine levels 24 h after incubation with iRBCL at ratios of 1:2, 1:4, 1:8, 1:16, or 1:32 (macrophage:iRBCL), uninfected RBCL at a ratio of 1:32 (macrophage:RBCL), or LPS. Each symbol represents the average value of three technical replicates. Results from two independent donors in two independent experiments are shown.

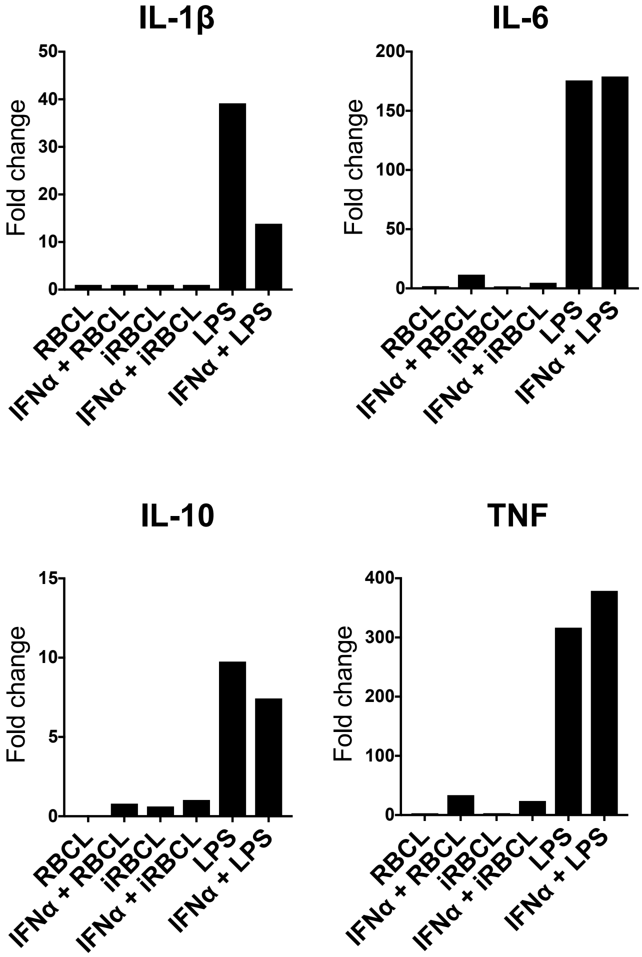

Appendix Fig. S3: Priming with IFNα does not affect macrophage inflammatory response to iRBCL. Macrophages were co-incubated with IFNα (2000 pg/ml) for 24 h. This is a representative result of two independent experiments.

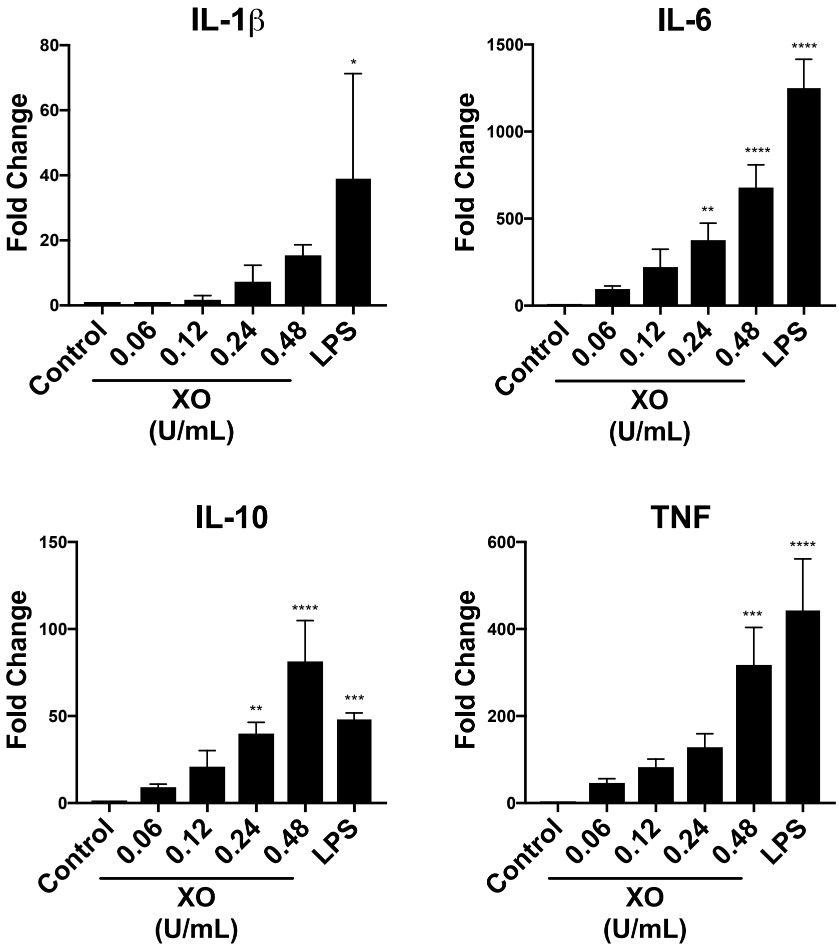

Appendix Fig. S4: Macrophages secrete cytokines in response to xanthine oxidase (XO) in a dose dependent manner. Cytokine levels 24 h after incubation with increasing concentrations of XO or LPS. Each bar represents the average value of three technical replicates. Asterisks denote level of significance: *p<0.05, **p<0.01, ***p<0.001, ****p<0001.

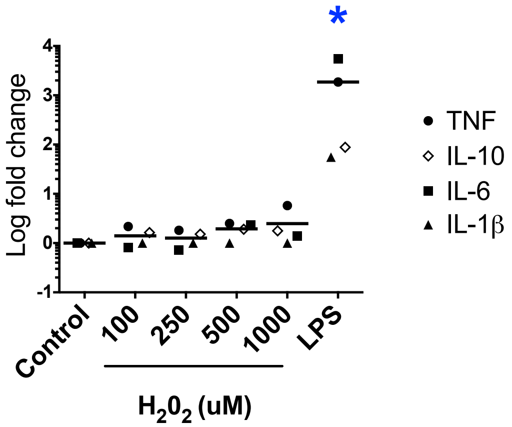

Appendix Fig. S5: Addition of H_2O_2 does not prompt macrophages to secrete cytokines. Cytokine levels 24 h after supplementation with increasing concentrations of H_2O_2 or LPS. This is a representative result of two independent experiments.

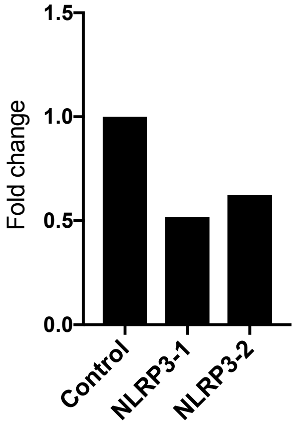

Appendix Fig. S6: siRNA transfection reduces NLRP3 mRNA expression in macrophages. RT-PCR of macrophages transfected with either control siRNA or siRNA against NLRP3 (NLRP3-1, NLRP3-2). Transfection of siRNAs targeting NLRP3 lead to a 50% decrease NLPR3 mRNA levels compared to control siRNA. Results are representative of 2 independent experiments with different donors.

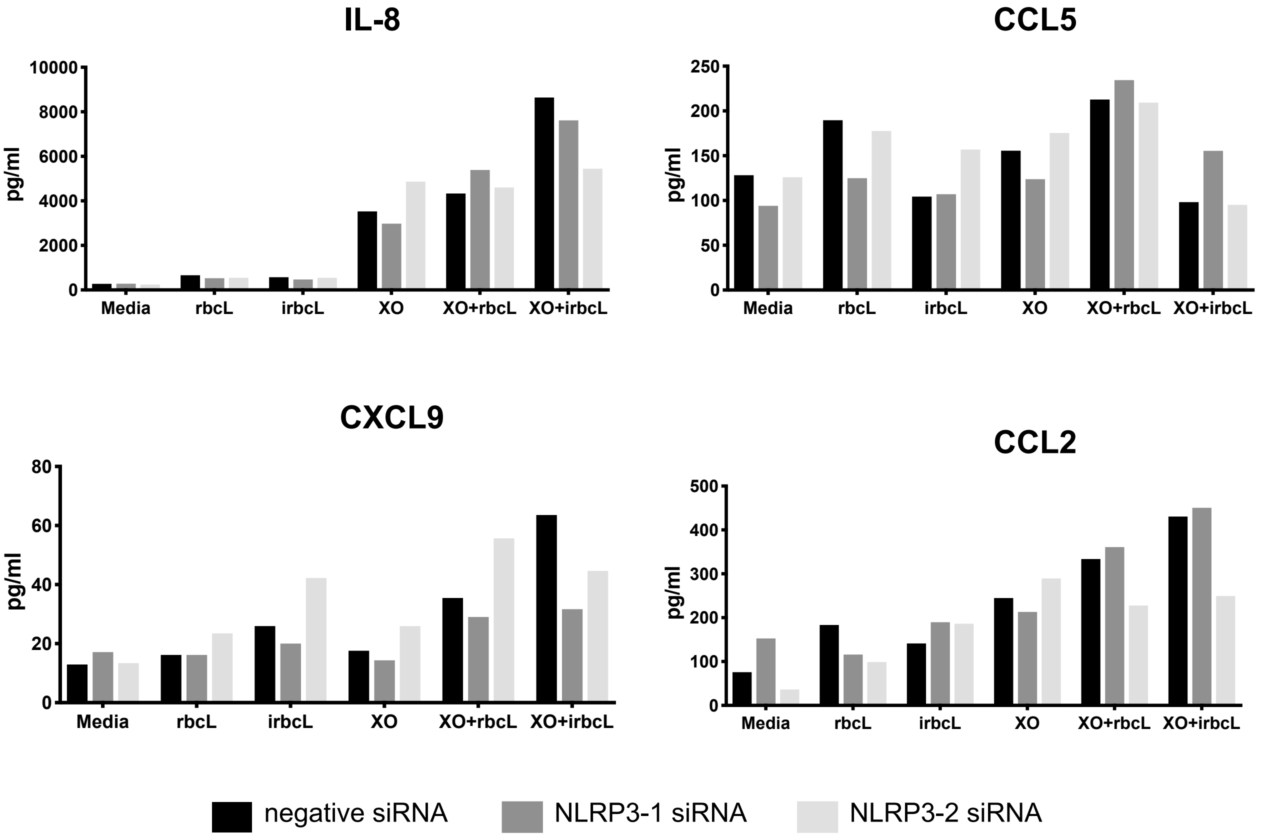

Appendix Fig. S7: NLRP3-siRNA transfection does not affect levels of chemokines. Two distinct siRNAs against NLRP3 and an irrelevant control siRNA were utilized to knock down RNA levels by lipofectamine transfection in macrophages that were later incubated with iRBCL, or RBCL as control, with or without XO, before quantification of secreted IL-8, CCL5, CXCL9 and CCL2. Results are representative of 2 independent experiments with different donors.
