# Supplementary material for: Malaria inflammation by xanthine oxidase‐produced reactive oxygen species
Source: EMBO Mol Med. 2019 Jul 2;11(8):e9903. doi: 10.15252/emmm.201809903 (PMC6685105; doi:10.15252/emmm.201809903)
Supplement: Supplementary file 2 — Source Data for Appendix [file EMMM-11-e9903-s008.zip › EV_source_data/Source_Data_Appendix_Fig_S1.pdf]

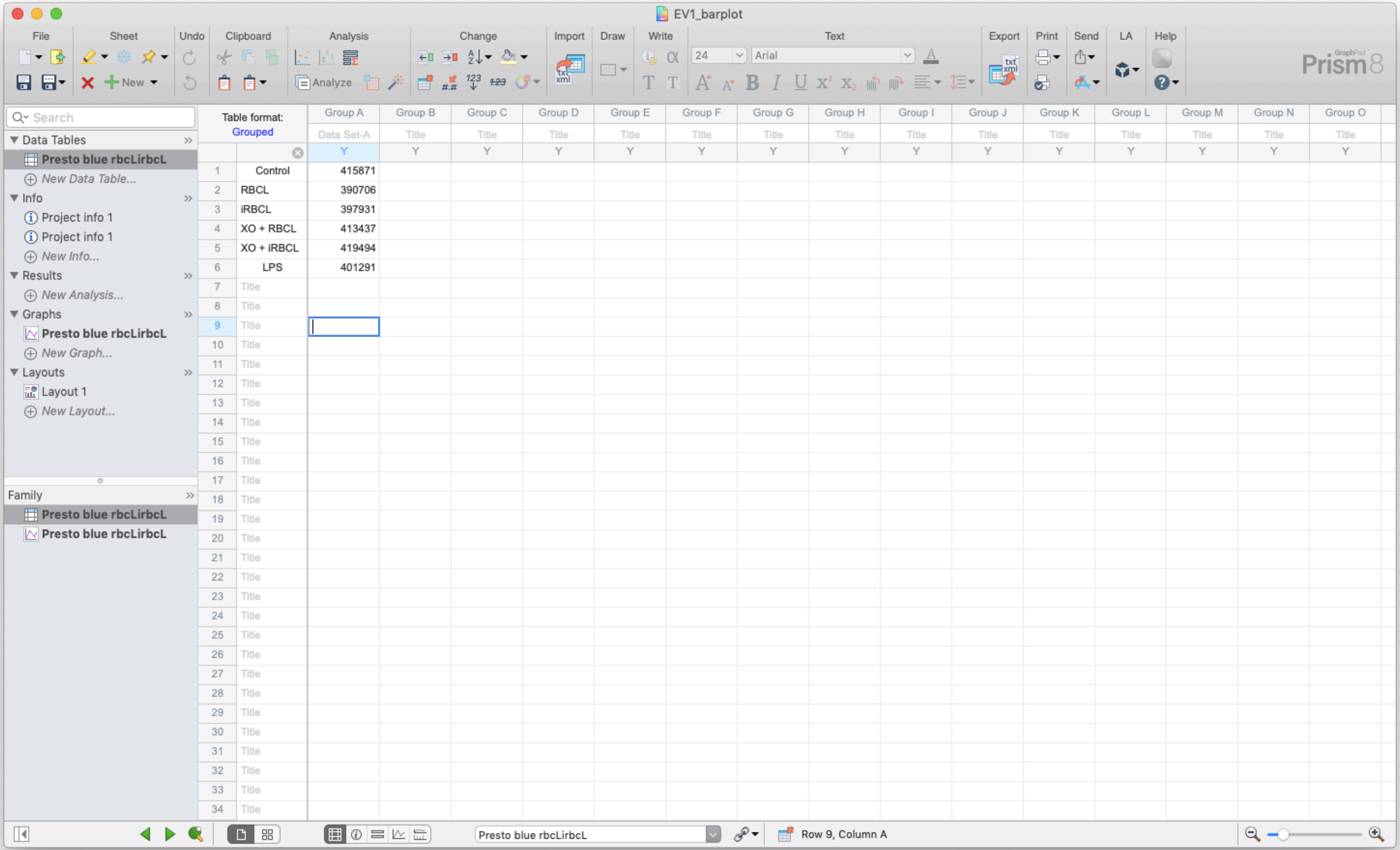
EV1_barplot
File
Sheet
Undo
Clipboard
Analysis
Change
Import
Draw
Write
Text
Export
Print
Send
LA
Help
Prism 8
Search
Data Tables
Presto blue rbcLirbcL
New Data Table...
Info
Project info 1
Project info 1
New Info...
Results
New Analysis...
Graphs
Presto blue rbcLirbcL
New Graph...
Layouts
Layout 1
New Layout...
Family
Presto blue rbcLirbcL
Presto blue rbcLirbcL
Table format:
Grouped
Group A
Data Set-A
Y
1 Control 415871
2 RBCL 390706
3 iRBCL 397931
4 XO + RBCL 413437
5 XO + iRBCL 419494
6 LPS 401291
Presto blue rbcLirbcL
Row 9, Column A
