# Supplementary material for: Malaria inflammation by xanthine oxidase‐produced reactive oxygen species
Source: EMBO Mol Med. 2019 Jul 2;11(8):e9903. doi: 10.15252/emmm.201809903 (PMC6685105; doi:10.15252/emmm.201809903)
Supplement: Supplementary file 2 — Source Data for Appendix [file EMMM-11-e9903-s008.zip › EV_source_data/Source_Data_Appendix_Fig_S2.pdf]

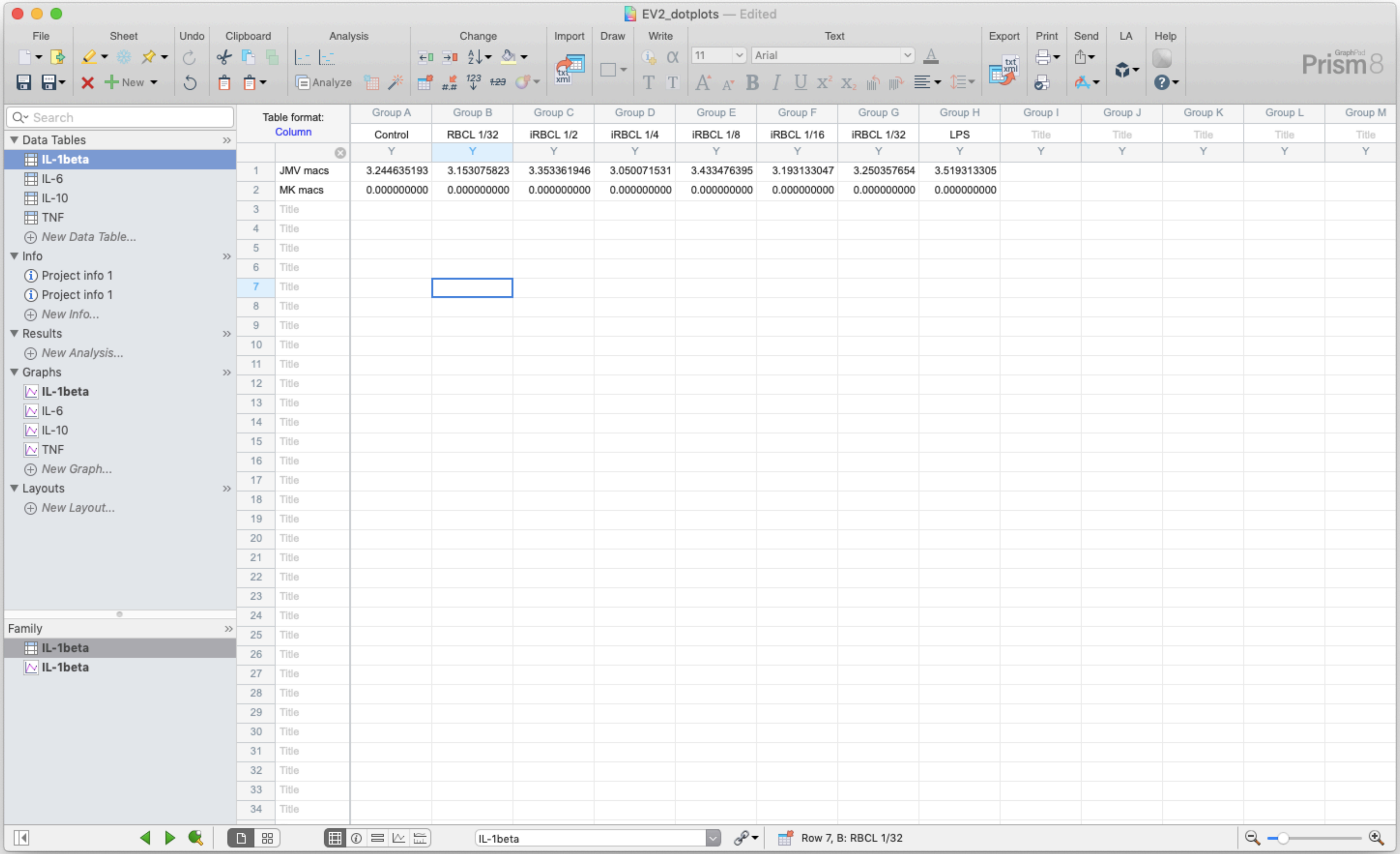

	Group A	Group B	Group C	Group D	Group E	Group F	Group G	Group H
	Control	RBCL 1/32	iRBCL 1/2	iRBCL 1/4	iRBCL 1/8	iRBCL 1/16	iRBCL 1/32	LPS
	Y	Y	Y	Y	Y	Y	Y	Y
1 JMV macs	3.244635193	3.153075823	3.353361946	3.050071531	3.433476395	3.193133047	3.250357654	3.519313305
2 MK macs	0.000000000	0.000000000	0.000000000	0.000000000	0.000000000	0.000000000	0.000000000	0.000000000

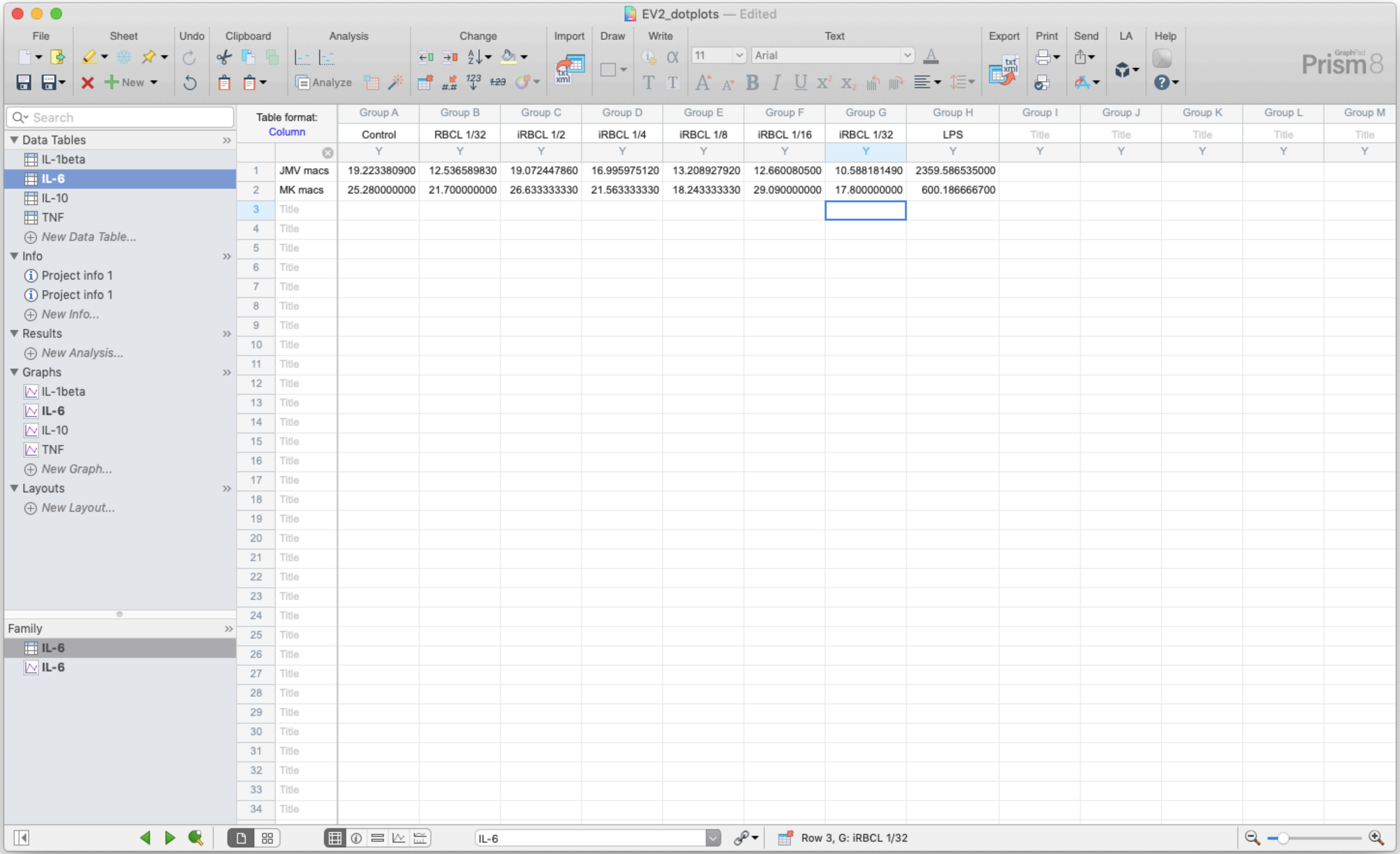

EV2_dotplots — Edited
Data Tables
IL-1beta
IL-6
IL-10
TNF
New Data Table...
Info
Project info 1
Project info 1
New Info...
Results
New Analysis...
Graphs
IL-1beta
IL-6
IL-10
TNF
New Graph...
Layouts
New Layout...
Family
IL-6
IL-6
Table format: Column
Group A Control: JMV macs 19.223380900; MK macs 25.280000000
Group B RBCL 1/32: JMV macs 12.536589830; MK macs 21.700000000
Group C iRBCL 1/2: JMV macs 19.072447860; MK macs 26.633333330
Group D iRBCL 1/4: JMV macs 16.995975120; MK macs 21.563333330
Group E iRBCL 1/8: JMV macs 13.208927920; MK macs 18.243333330
Group F iRBCL 1/16: JMV macs 12.660080500; MK macs 29.090000000
Group G iRBCL 1/32: JMV macs 10.588181490; MK macs 17.800000000
Group H LPS: JMV macs 2359.586535000; MK macs 600.186666700
Row 3, G: iRBCL 1/32

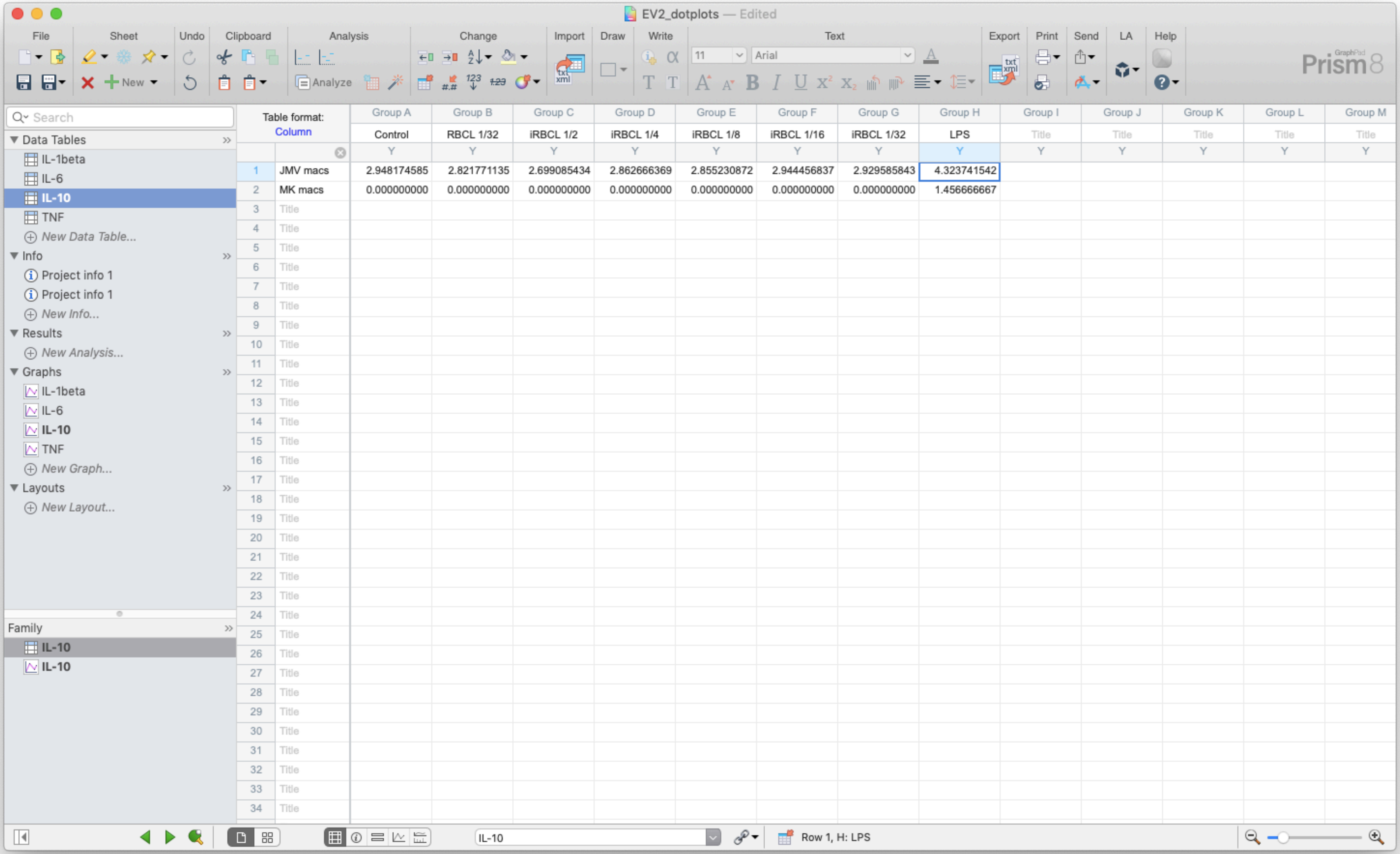

	Group A	Group B	Group C	Group D	Group E	Group F	Group G	Group H
	Control	RBCL 1/32	iRBCL 1/2	iRBCL 1/4	iRBCL 1/8	iRBCL 1/16	iRBCL 1/32	LPS
	Y	Y	Y	Y	Y	Y	Y	Y
1 JMV macs	2.948174585	2.821771135	2.699085434	2.862666369	2.855230872	2.944456837	2.929585843	4.323741542
2 MK macs	0.000000000	0.000000000	0.000000000	0.000000000	0.000000000	0.000000000	0.000000000	1.456666667

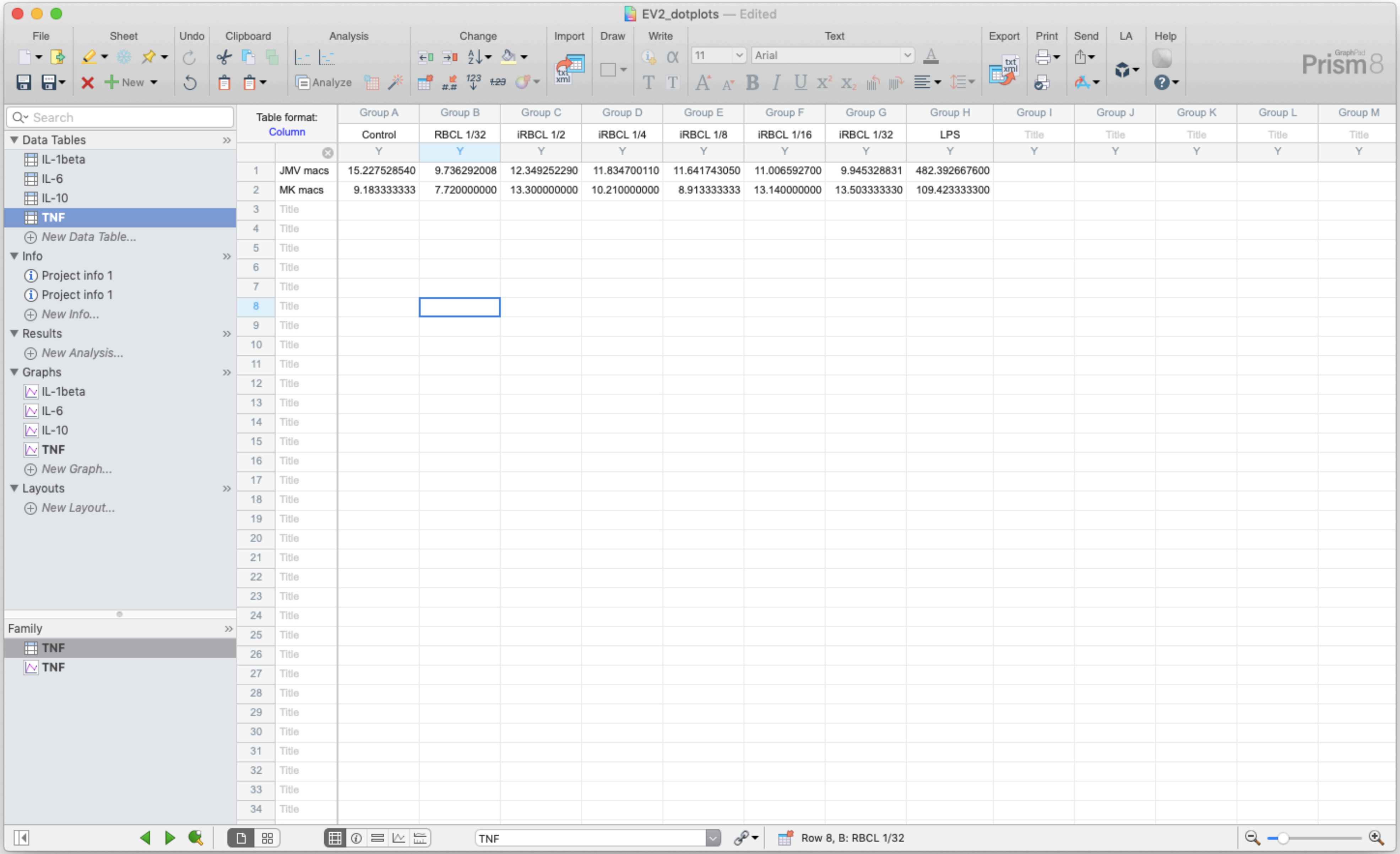
EV2_dotplots — Edited
Prism 8
Data Tables
IL-1beta
IL-6
IL-10
TNF
New Data Table...
Info
Project info 1
Project info 1
New Info...
Results
New Analysis...
Graphs
IL-1beta
IL-6
IL-10
TNF
New Graph...
Layouts
New Layout...
Family
TNF
TNF
Table format: Column
Group A Control; Group B RBCL 1/32; Group C iRBCL 1/2; Group D iRBCL 1/4; Group E iRBCL 1/8; Group F iRBCL 1/16; Group G iRBCL 1/32; Group H LPS
1 JMV macs 15.227528540 9.736292008 12.349252290 11.834700110 11.641743050 11.006592700 9.945328831 482.392667600
2 MK macs 9.183333333 7.720000000 13.300000000 10.210000000 8.913333333 13.140000000 13.503333330 109.423333300
Row 8, B: RBCL 1/32
