# Supplementary material for: Malaria inflammation by xanthine oxidase‐produced reactive oxygen species
Source: EMBO Mol Med. 2019 Jul 2;11(8):e9903. doi: 10.15252/emmm.201809903 (PMC6685105; doi:10.15252/emmm.201809903)
Supplement: Supplementary file 2 — Source Data for Appendix [file EMMM-11-e9903-s008.zip › EV_source_data/Source_Data_Appendix_Fig_S3.pdf]

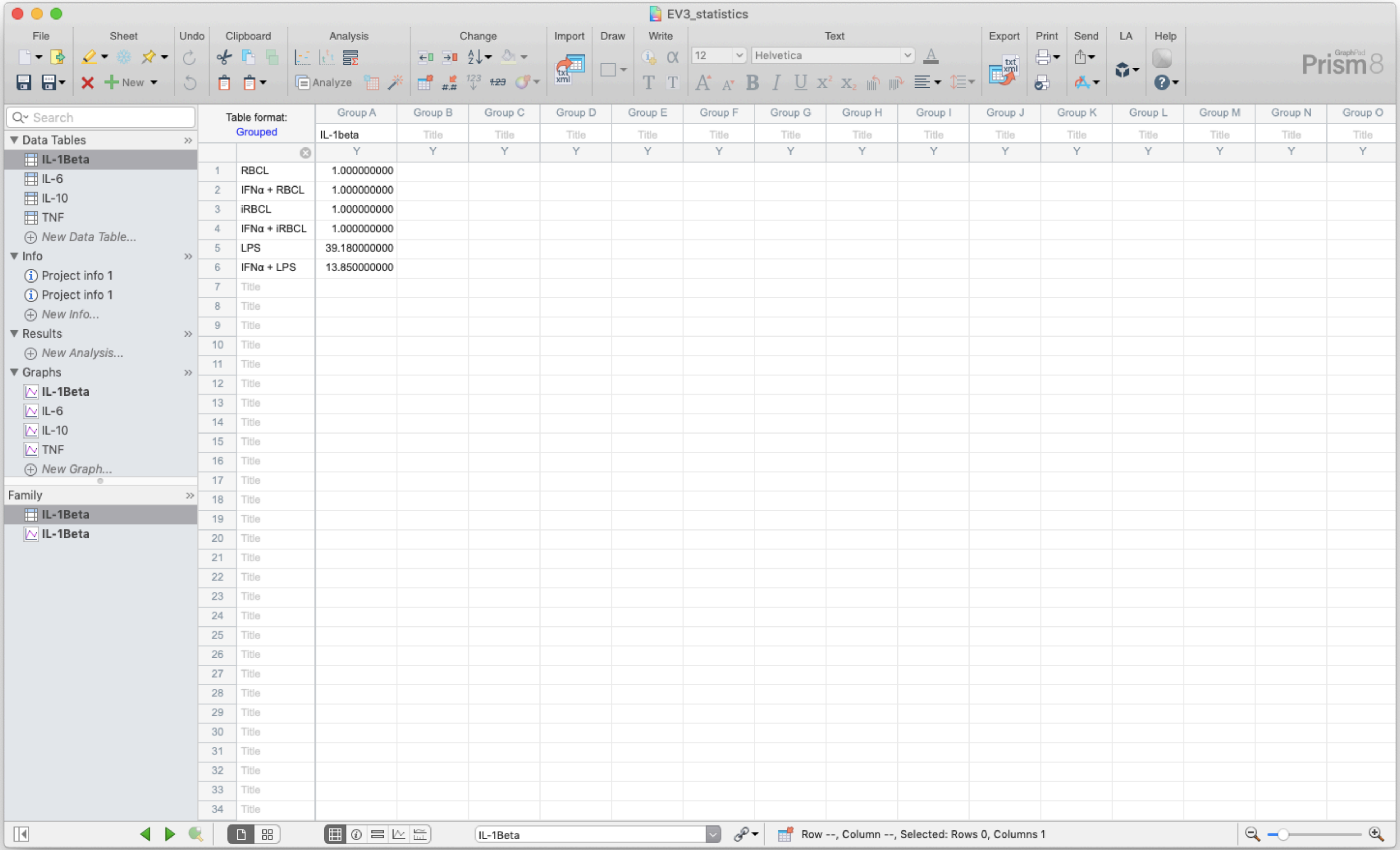
EV3_statistics
File
Sheet
Undo
Clipboard
Analysis
Change
Import
Draw
Write
Text
Export
Print
Send
LA
Help
Prism 8
Search
Data Tables
IL-1Beta
IL-6
IL-10
TNF
New Data Table...
Info
Project info 1
Project info 1
New Info...
Results
New Analysis...
Graphs
IL-1Beta
IL-6
IL-10
TNF
New Graph...
Family
IL-1Beta
IL-1Beta
Table format:
Grouped
Group A
IL-1beta
1 RBCL 1.000000000
2 IFNα + RBCL 1.000000000
3 iRBCL 1.000000000
4 IFNα + iRBCL 1.000000000
5 LPS 39.180000000
6 IFNα + LPS 13.850000000
Row --, Column --, Selected: Rows 0, Columns 1

EV3_statistics

Table format: Grouped

		Group A
		IL-6
		Y
1	RBCL	1.180914513
2	IFNα + RBCL	11.572564610
3	iRBCL	0.862602165
4	IFNα + iRBCL	4.782416611
5	LPS	175.690744400
6	IFNα + LPS	178.964214700

Data Tables: IL-1Beta, IL-6, IL-10, TNF

Info: Project info 1, Project info 1

Graphs: IL-1Beta, IL-6, IL-10, TNF

Family: IL-6, IL-6

Row --, Column --, Selected: Rows 0, Columns 1

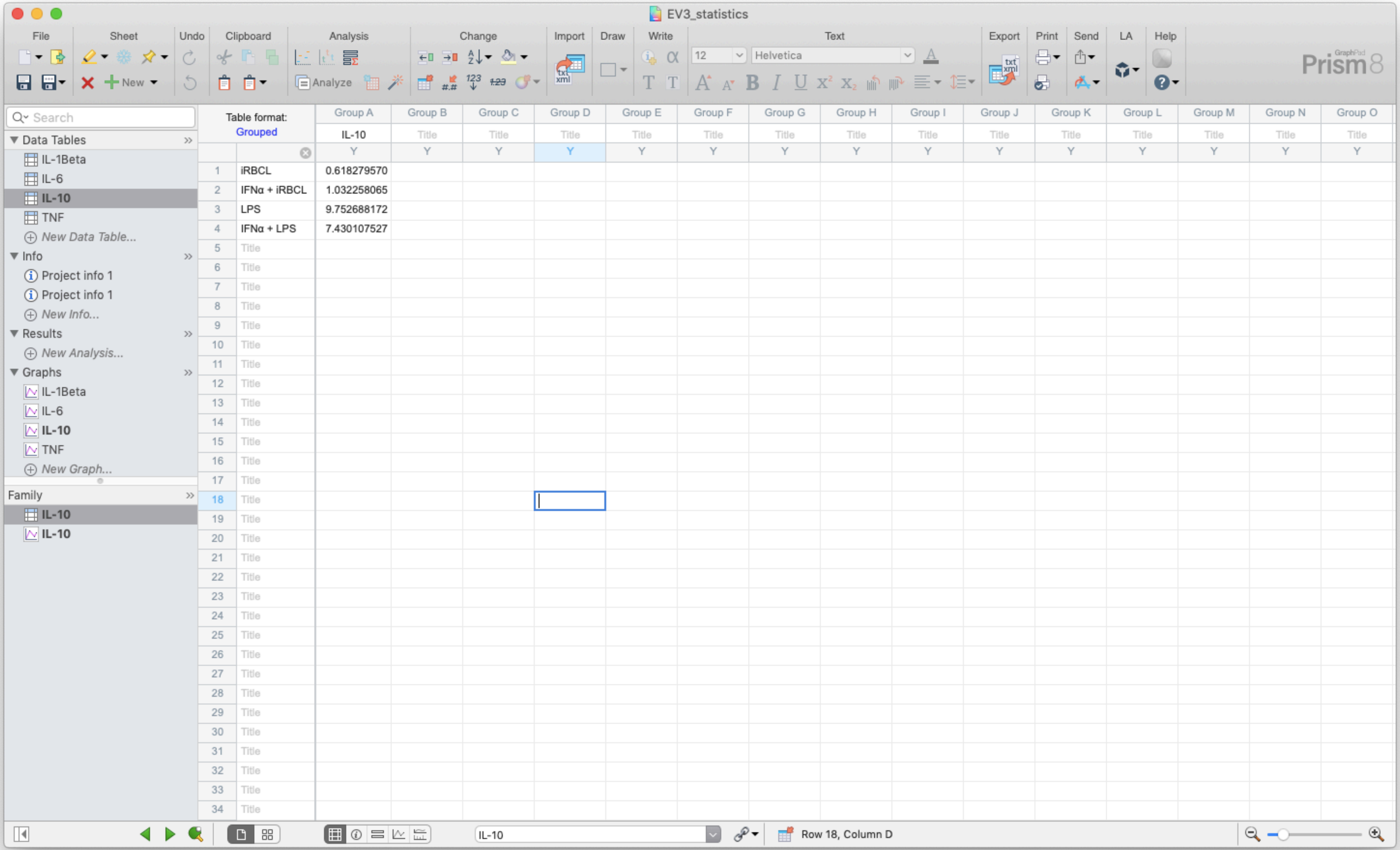
EV3_statistics
File
Sheet
Undo
Clipboard
Analysis
Change
Import
Draw
Write
Text
Export
Print
Send
LA
Help
Prism 8
New
Analyze
Helvetica
12
Search
Data Tables
IL-1Beta
IL-6
IL-10
TNF
New Data Table...
Info
Project info 1
Project info 1
New Info...
Results
New Analysis...
Graphs
IL-1Beta
IL-6
IL-10
TNF
New Graph...
Family
IL-10
IL-10
Table format:
Grouped
Group A
IL-10
Group B
Group C
Group D
Group E
Group F
Group G
Group H
Group I
Group J
Group K
Group L
Group M
Group N
Group O
1 iRBCL 0.618279570
2 IFNα + iRBCL 1.032258065
3 LPS 9.752688172
4 IFNα + LPS 7.430107527
IL-10
Row 18, Column D

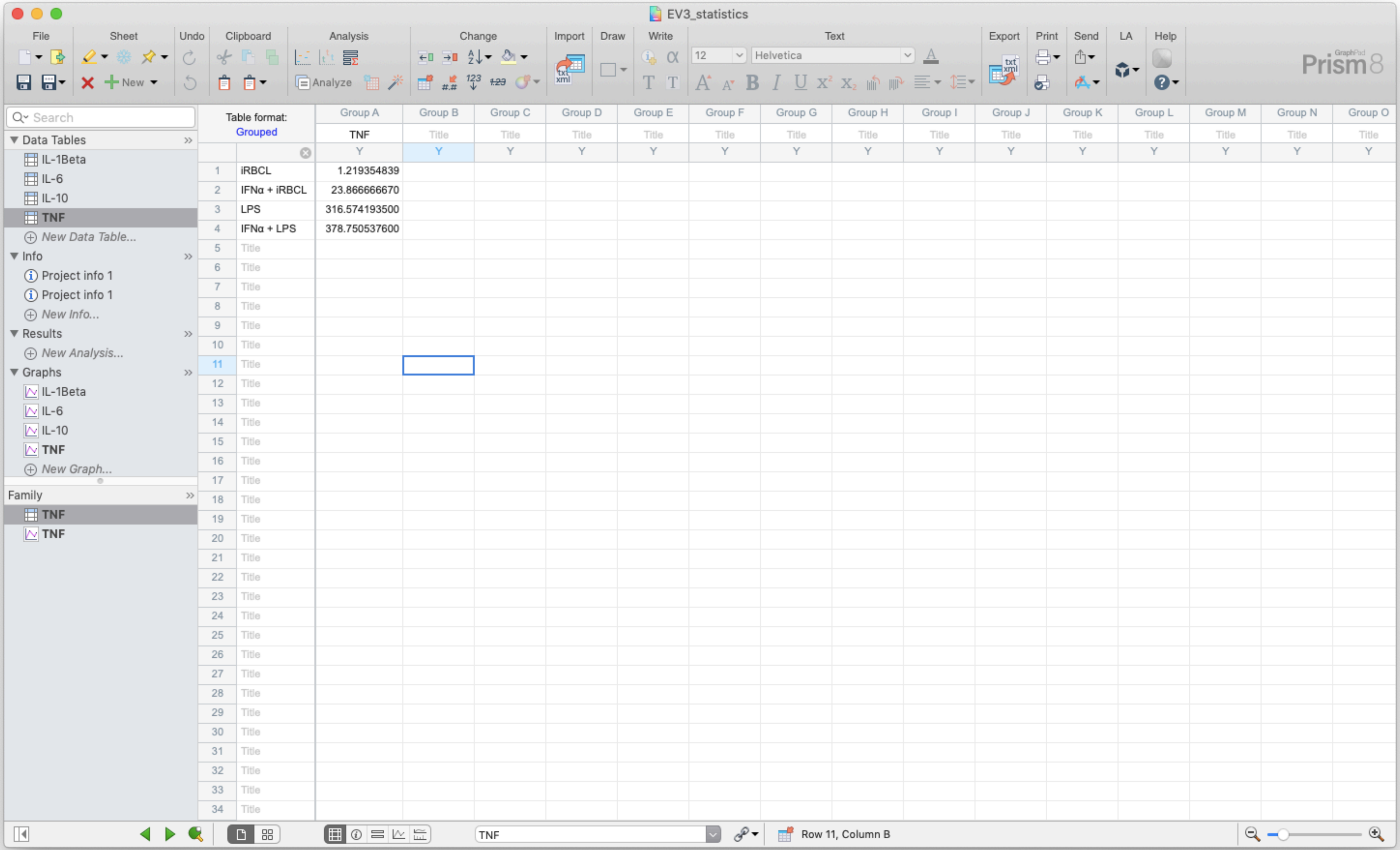
EV3_statistics
Table format:
Grouped
Data Tables
IL-1Beta
IL-6
IL-10
TNF
New Data Table...
Info
Project info 1
Project info 1
New Info...
Results
New Analysis...
Graphs
IL-1Beta
IL-6
IL-10
TNF
New Graph...
Family
TNF
TNF
Group A
TNF
1 iRBCL 1.219354839
2 IFNα + iRBCL 23.866666670
3 LPS 316.574193500
4 IFNα + LPS 378.750537600
Row 11, Column B
Prism 8
