# Supplementary material for: Malaria inflammation by xanthine oxidase‐produced reactive oxygen species
Source: EMBO Mol Med. 2019 Jul 2;11(8):e9903. doi: 10.15252/emmm.201809903 (PMC6685105; doi:10.15252/emmm.201809903)
Supplement: Supplementary file 2 — Source Data for Appendix [file EMMM-11-e9903-s008.zip › EV_source_data/Source_Data_Appendix_Fig_S4.pdf]

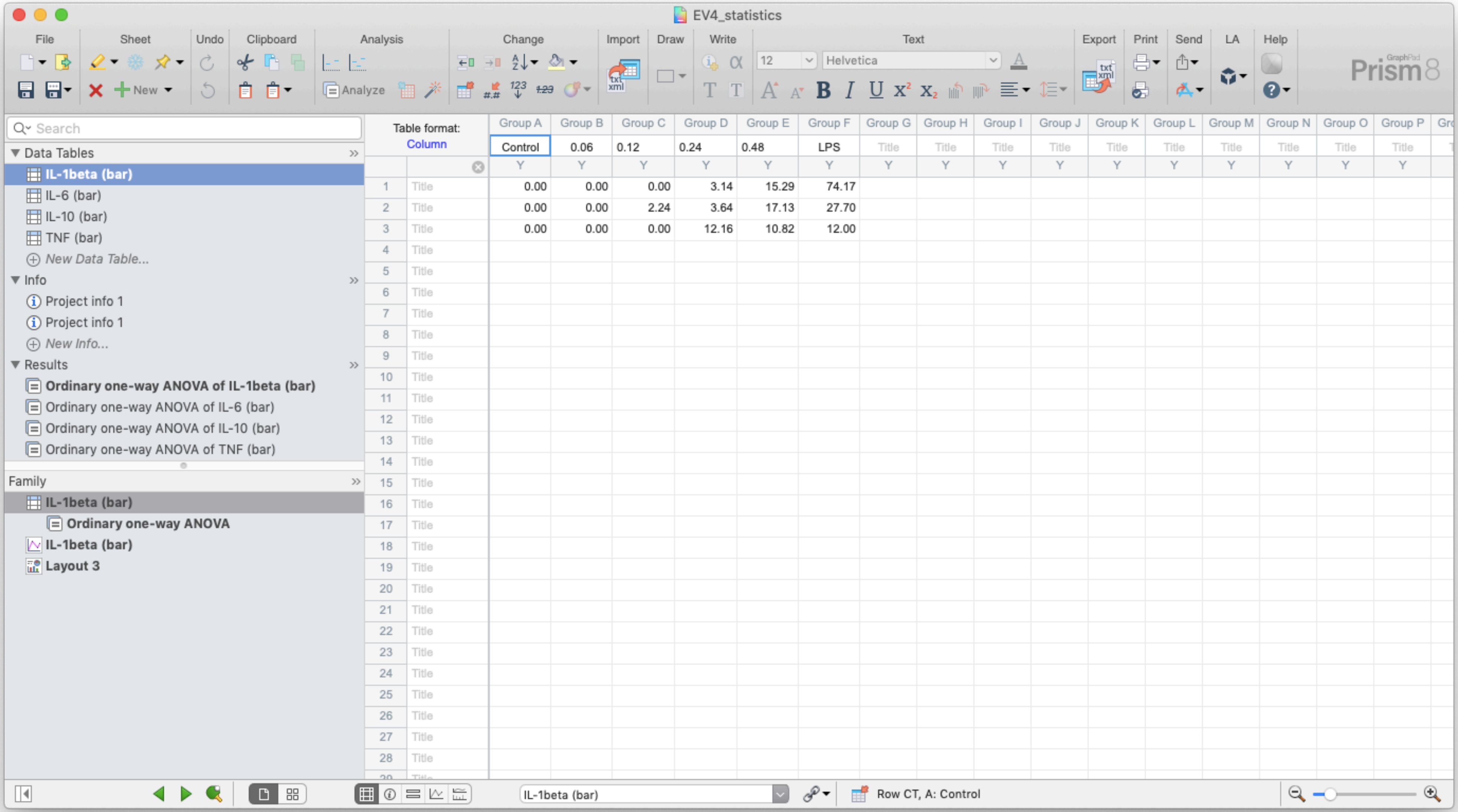
EV4_statistics
File
Sheet
Undo
Clipboard
Analysis
Change
Import
Draw
Write
Text
Export
Print
Send
LA
Help
Prism 8
Analyze
New
Search
Data Tables
IL-1beta (bar)
IL-6 (bar)
IL-10 (bar)
TNF (bar)
New Data Table...
Info
Project info 1
Project info 1
New Info...
Results
Ordinary one-way ANOVA of IL-1beta (bar)
Ordinary one-way ANOVA of IL-6 (bar)
Ordinary one-way ANOVA of IL-10 (bar)
Ordinary one-way ANOVA of TNF (bar)
Family
IL-1beta (bar)
Ordinary one-way ANOVA
IL-1beta (bar)
Layout 3
Table format:
Column
Group A Group B Group C Group D Group E Group F
Control 0.06 0.12 0.24 0.48 LPS
1 0.00 0.00 0.00 3.14 15.29 74.17
2 0.00 0.00 2.24 3.64 17.13 27.70
3 0.00 0.00 0.00 12.16 10.82 12.00
IL-1beta (bar)
Row CT, A: Control

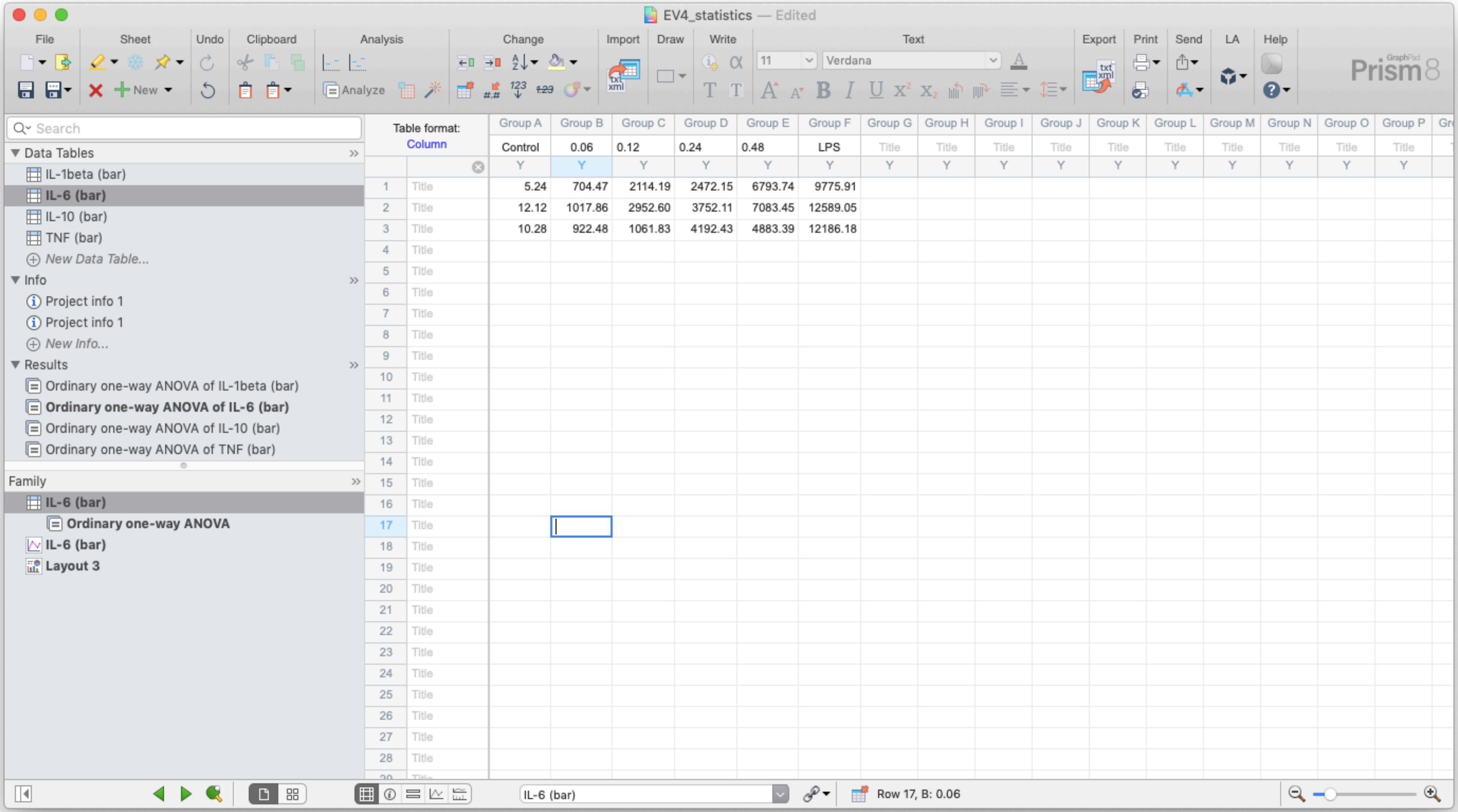
EV4_statistics — Edited
File
Sheet
Undo
Clipboard
Analysis
Change
Import
Draw
Write
Text
Export
Print
Send
LA
Help
New
Analyze
Verdana
Prism 8
Search
Data Tables
IL-1beta (bar)
IL-6 (bar)
IL-10 (bar)
TNF (bar)
New Data Table...
Info
Project info 1
Project info 1
New Info...
Results
Ordinary one-way ANOVA of IL-1beta (bar)
Ordinary one-way ANOVA of IL-6 (bar)
Ordinary one-way ANOVA of IL-10 (bar)
Ordinary one-way ANOVA of TNF (bar)
Family
IL-6 (bar)
Ordinary one-way ANOVA
IL-6 (bar)
Layout 3
Table format:
Column
Group A
Group B
Group C
Group D
Group E
Group F
Control
0.06
0.12
0.24
0.48
LPS
5.24
704.47
2114.19
2472.15
6793.74
9775.91
12.12
1017.86
2952.60
3752.11
7083.45
12589.05
10.28
922.48
1061.83
4192.43
4883.39
12186.18
IL-6 (bar)
Row 17, B: 0.06

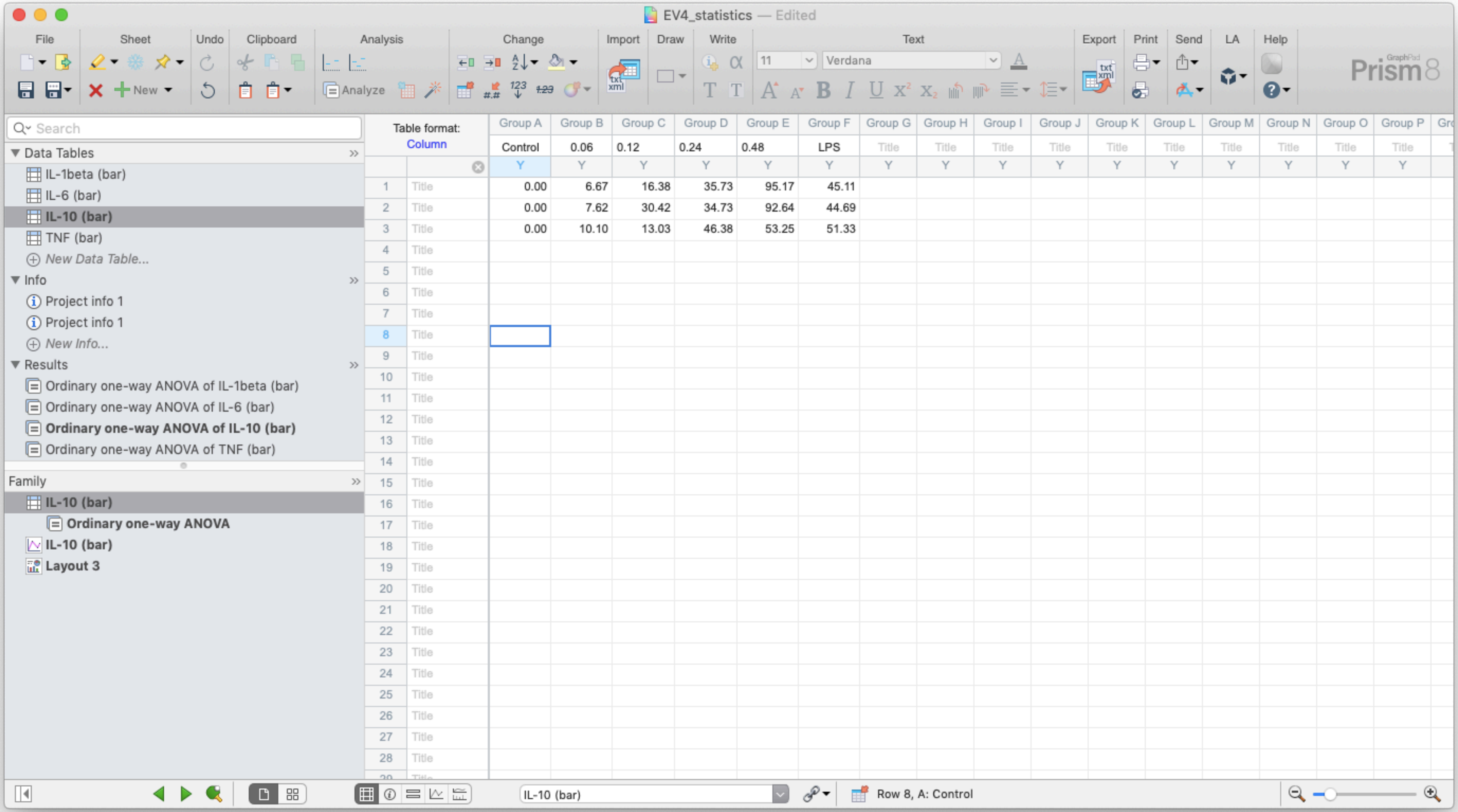
EV4_statistics — Edited
File
Sheet
Undo
Clipboard
Analysis
Change
Import
Draw
Write
Text
Export
Print
Send
LA
Help
Prism 8
Analyze
New
Verdana
Search
Data Tables
IL-1beta (bar)
IL-6 (bar)
IL-10 (bar)
TNF (bar)
New Data Table...
Info
Project info 1
Project info 1
New Info...
Results
Ordinary one-way ANOVA of IL-1beta (bar)
Ordinary one-way ANOVA of IL-6 (bar)
Ordinary one-way ANOVA of IL-10 (bar)
Ordinary one-way ANOVA of TNF (bar)
Family
IL-10 (bar)
Ordinary one-way ANOVA
IL-10 (bar)
Layout 3
Table format: Column
Group A Control
Group B 0.06
Group C 0.12
Group D 0.24
Group E 0.48
Group F LPS
1 0.00 6.67 16.38 35.73 95.17 45.11
2 0.00 7.62 30.42 34.73 92.64 44.69
3 0.00 10.10 13.03 46.38 53.25 51.33
IL-10 (bar)
Row 8, A: Control

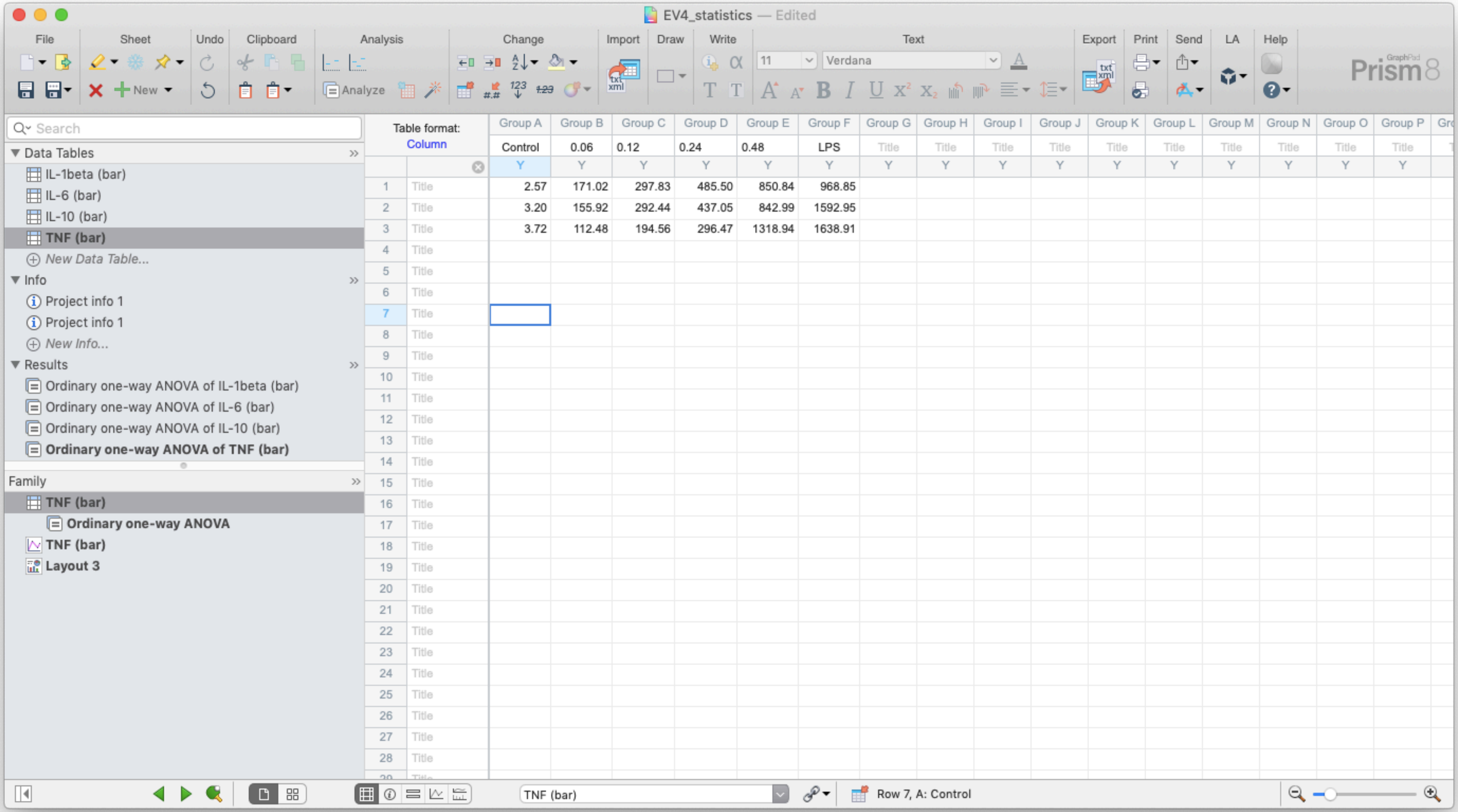
EV4_statistics — Edited
File
Sheet
Undo
Clipboard
Analysis
Change
Import
Draw
Write
Text
Export
Print
Send
LA
Help
Verdana
Analyze
New
Prism 8
Search
Data Tables
IL-1beta (bar)
IL-6 (bar)
IL-10 (bar)
TNF (bar)
New Data Table...
Info
Project info 1
Project info 1
New Info...
Results
Ordinary one-way ANOVA of IL-1beta (bar)
Ordinary one-way ANOVA of IL-6 (bar)
Ordinary one-way ANOVA of IL-10 (bar)
Ordinary one-way ANOVA of TNF (bar)
Family
TNF (bar)
Ordinary one-way ANOVA
TNF (bar)
Layout 3
Table format: Column
Group A Group B Group C Group D Group E Group F
Control 0.06 0.12 0.24 0.48 LPS
1 2.57 171.02 297.83 485.50 850.84 968.85
2 3.20 155.92 292.44 437.05 842.99 1592.95
3 3.72 112.48 194.56 296.47 1318.94 1638.91
TNF (bar)
Row 7, A: Control

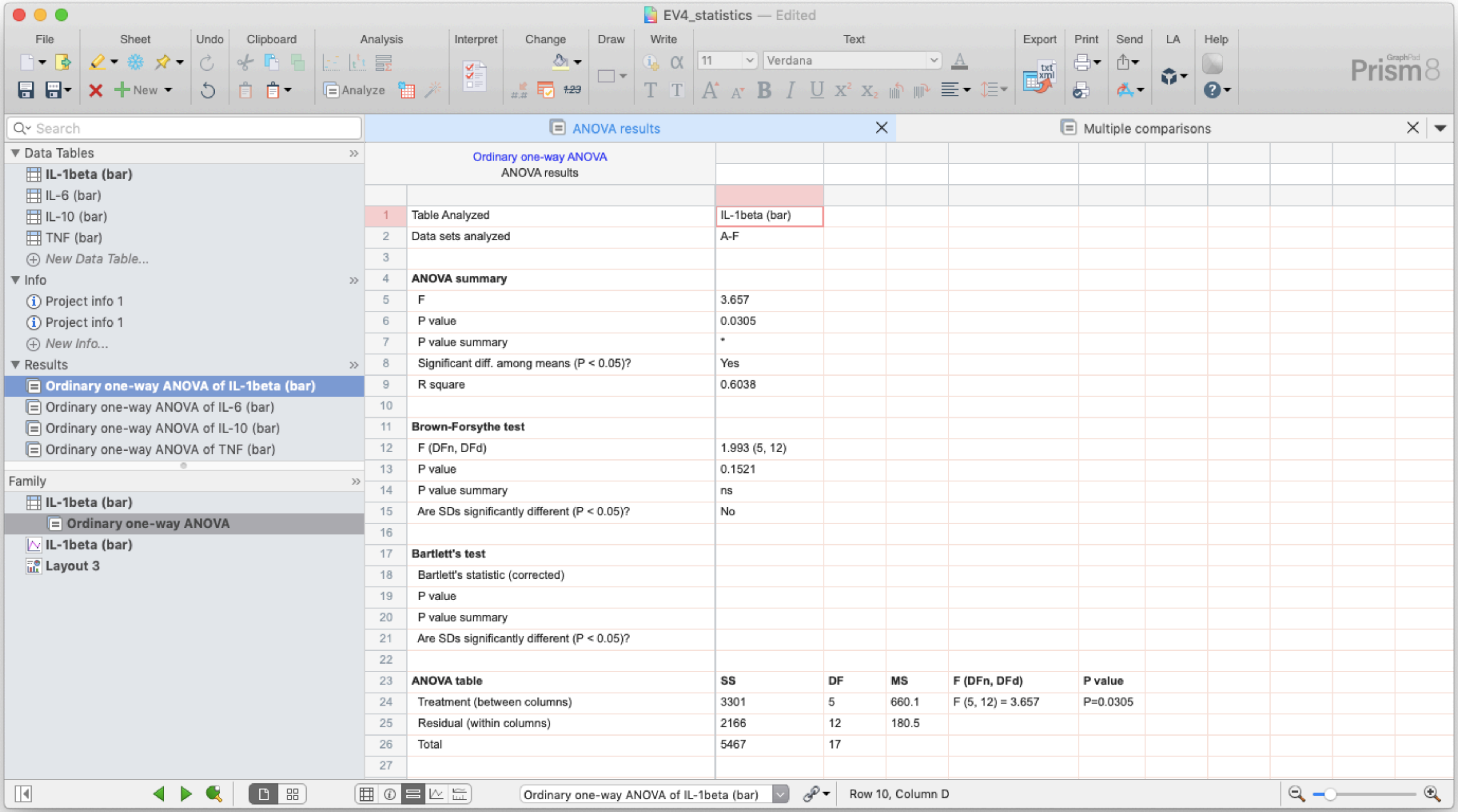

Ordinary one-way ANOVA
ANOVA results

1	Table Analyzed	IL-1beta (bar)				
2	Data sets analyzed	A-F				
3						
4	**ANOVA summary**					
5	F	3.657				
6	P value	0.0305				
7	P value summary	*				
8	Significant diff. among means (P < 0.05)?	Yes				
9	R square	0.6038				
10						
11	**Brown-Forsythe test**					
12	F (DFn, DFd)	1.993 (5, 12)				
13	P value	0.1521				
14	P value summary	ns				
15	Are SDs significantly different (P < 0.05)?	No				
16						
17	**Bartlett's test**					
18	Bartlett's statistic (corrected)					
19	P value					
20	P value summary					
21	Are SDs significantly different (P < 0.05)?					
22						
23	**ANOVA table**	**SS**	**DF**	**MS**	**F (DFn, DFd)**	**P value**
24	Treatment (between columns)	3301	5	660.1	F (5, 12) = 3.657	P=0.0305
25	Residual (within columns)	2166	12	180.5		
26	Total	5467	17			
27						

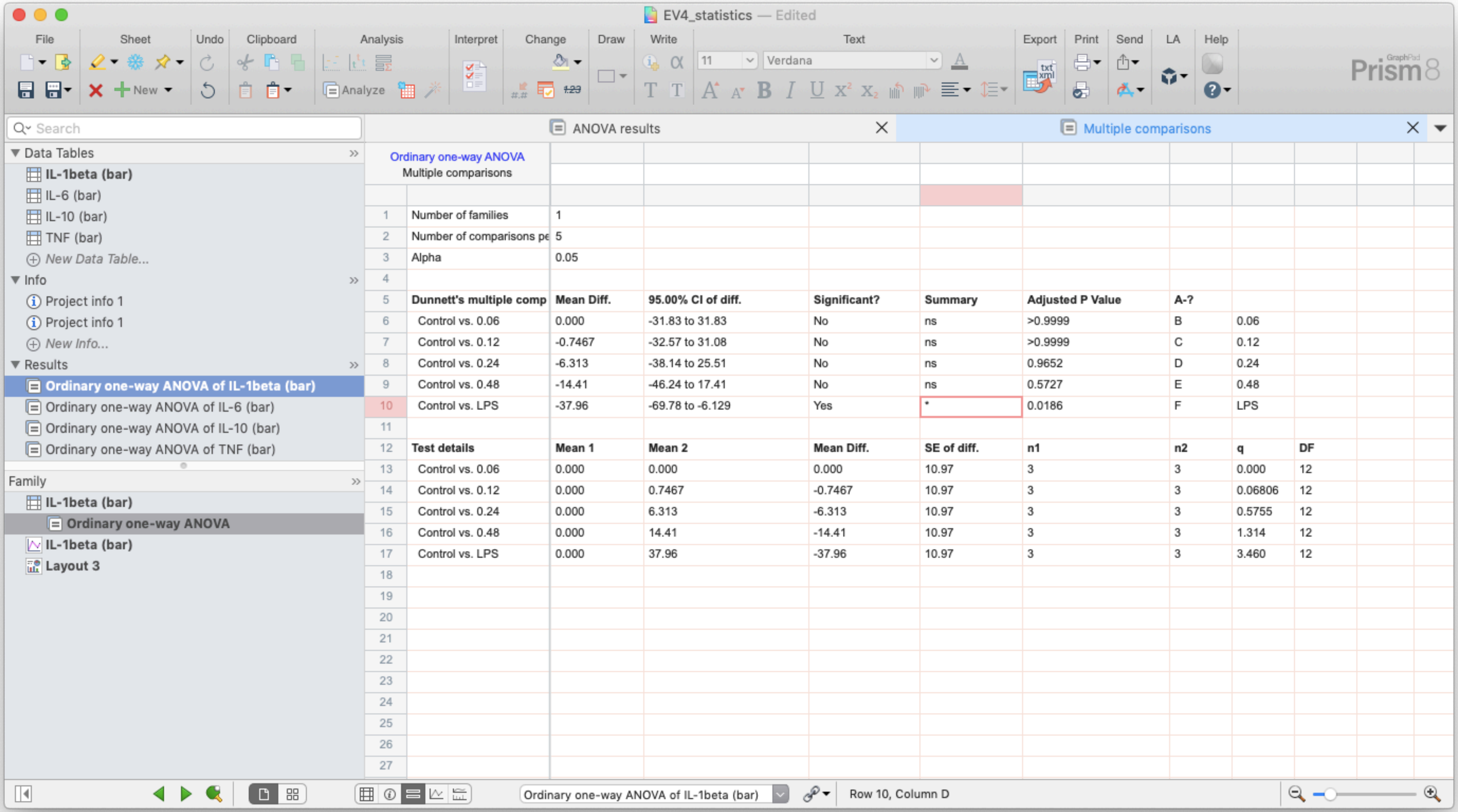

Ordinary one-way ANOVA
Multiple comparisons

1	Number of families	1							
2	Number of comparisons pe	5							
3	Alpha	0.05							
4									
5	Dunnett's multiple comp	Mean Diff.	95.00% CI of diff.	Significant?	Summary	Adjusted P Value	A-?		
6	Control vs. 0.06	0.000	-31.83 to 31.83	No	ns	>0.9999	B	0.06	
7	Control vs. 0.12	-0.7467	-32.57 to 31.08	No	ns	>0.9999	C	0.12	
8	Control vs. 0.24	-6.313	-38.14 to 25.51	No	ns	0.9652	D	0.24	
9	Control vs. 0.48	-14.41	-46.24 to 17.41	No	ns	0.5727	E	0.48	
10	Control vs. LPS	-37.96	-69.78 to -6.129	Yes	*	0.0186	F	LPS	
11									
12	Test details	Mean 1	Mean 2	Mean Diff.	SE of diff.	n1	n2	q	DF
13	Control vs. 0.06	0.000	0.000	0.000	10.97	3	3	0.000	12
14	Control vs. 0.12	0.000	0.7467	-0.7467	10.97	3	3	0.06806	12
15	Control vs. 0.24	0.000	6.313	-6.313	10.97	3	3	0.5755	12
16	Control vs. 0.48	0.000	14.41	-14.41	10.97	3	3	1.314	12
17	Control vs. LPS	0.000	37.96	-37.96	10.97	3	3	3.460	12

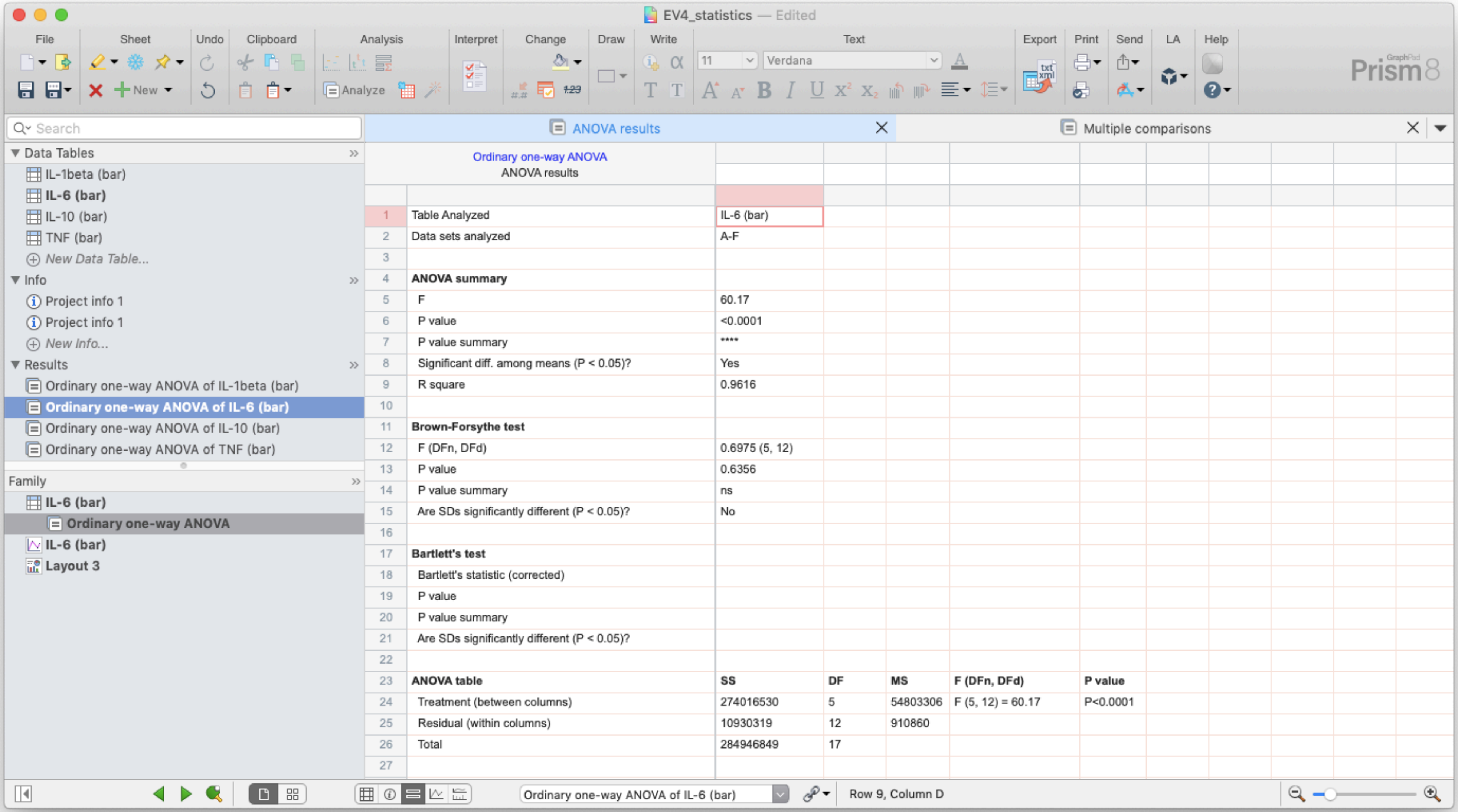

Ordinary one-way ANOVA
ANOVA results

1	Table Analyzed	IL-6 (bar)				
2	Data sets analyzed	A-F				
3						
4	**ANOVA summary**					
5	F	60.17				
6	P value	<0.0001				
7	P value summary	****				
8	Significant diff. among means (P < 0.05)?	Yes				
9	R square	0.9616				
10						
11	**Brown-Forsythe test**					
12	F (DFn, DFd)	0.6975 (5, 12)				
13	P value	0.6356				
14	P value summary	ns				
15	Are SDs significantly different (P < 0.05)?	No				
16						
17	**Bartlett's test**					
18	Bartlett's statistic (corrected)					
19	P value					
20	P value summary					
21	Are SDs significantly different (P < 0.05)?					
22						
23	**ANOVA table**	**SS**	**DF**	**MS**	**F (DFn, DFd)**	**P value**
24	Treatment (between columns)	274016530	5	54803306	F (5, 12) = 60.17	P<0.0001
25	Residual (within columns)	10930319	12	910860		
26	Total	284946849	17			
27						

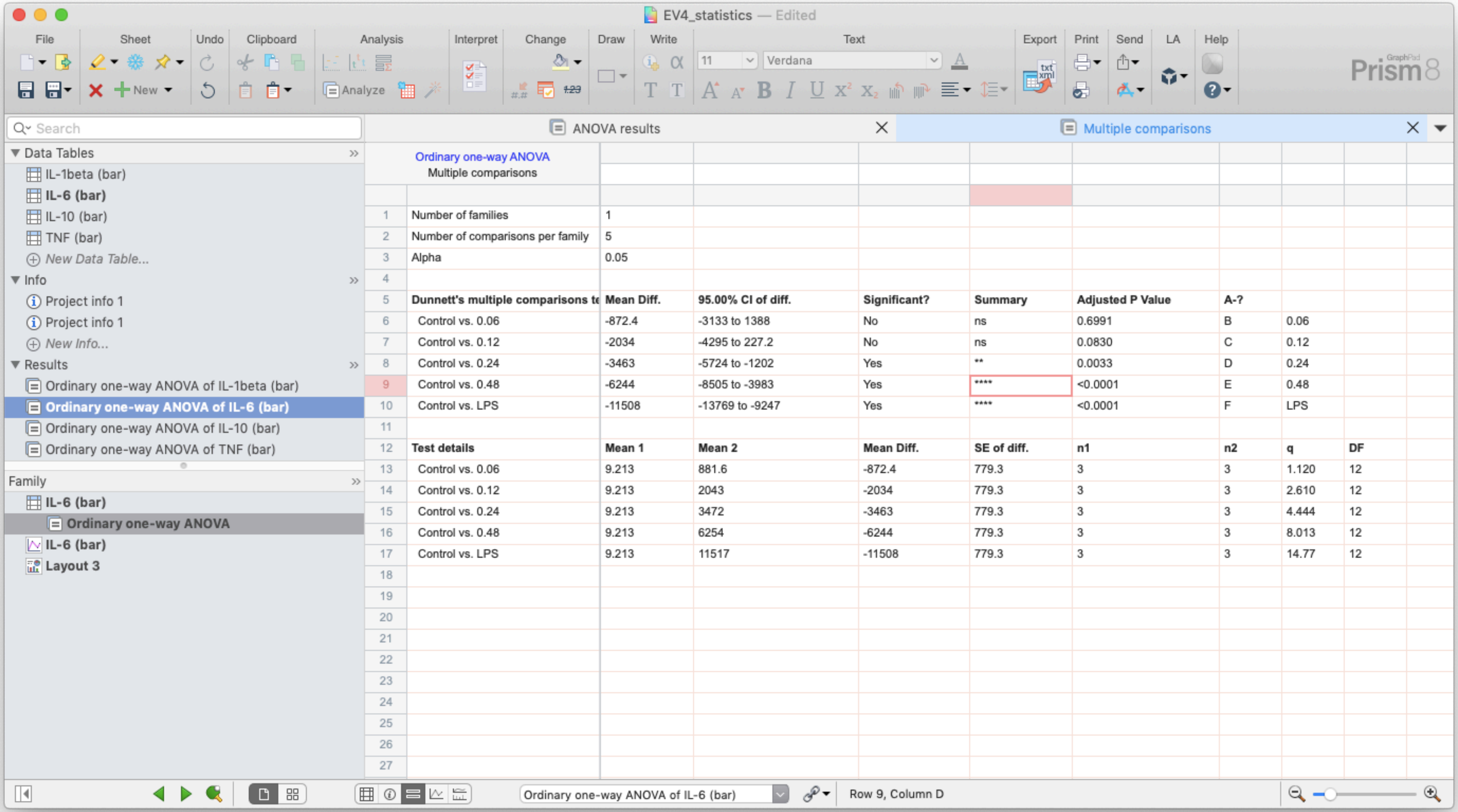

Number of families	1							
Number of comparisons per family	5							
Alpha	0.05							
Dunnett's multiple comparisons te	Mean Diff.	95.00% CI of diff.	Significant?	Summary	Adjusted P Value	A-?		
Control vs. 0.06	-872.4	-3133 to 1388	No	ns	0.6991	B	0.06	
Control vs. 0.12	-2034	-4295 to 227.2	No	ns	0.0830	C	0.12	
Control vs. 0.24	-3463	-5724 to -1202	Yes	**	0.0033	D	0.24	
Control vs. 0.48	-6244	-8505 to -3983	Yes	****	<0.0001	E	0.48	
Control vs. LPS	-11508	-13769 to -9247	Yes	****	<0.0001	F	LPS	
Test details	Mean 1	Mean 2	Mean Diff.	SE of diff.	n1	n2	q	DF
Control vs. 0.06	9.213	881.6	-872.4	779.3	3	3	1.120	12
Control vs. 0.12	9.213	2043	-2034	779.3	3	3	2.610	12
Control vs. 0.24	9.213	3472	-3463	779.3	3	3	4.444	12
Control vs. 0.48	9.213	6254	-6244	779.3	3	3	8.013	12
Control vs. LPS	9.213	11517	-11508	779.3	3	3	14.77	12

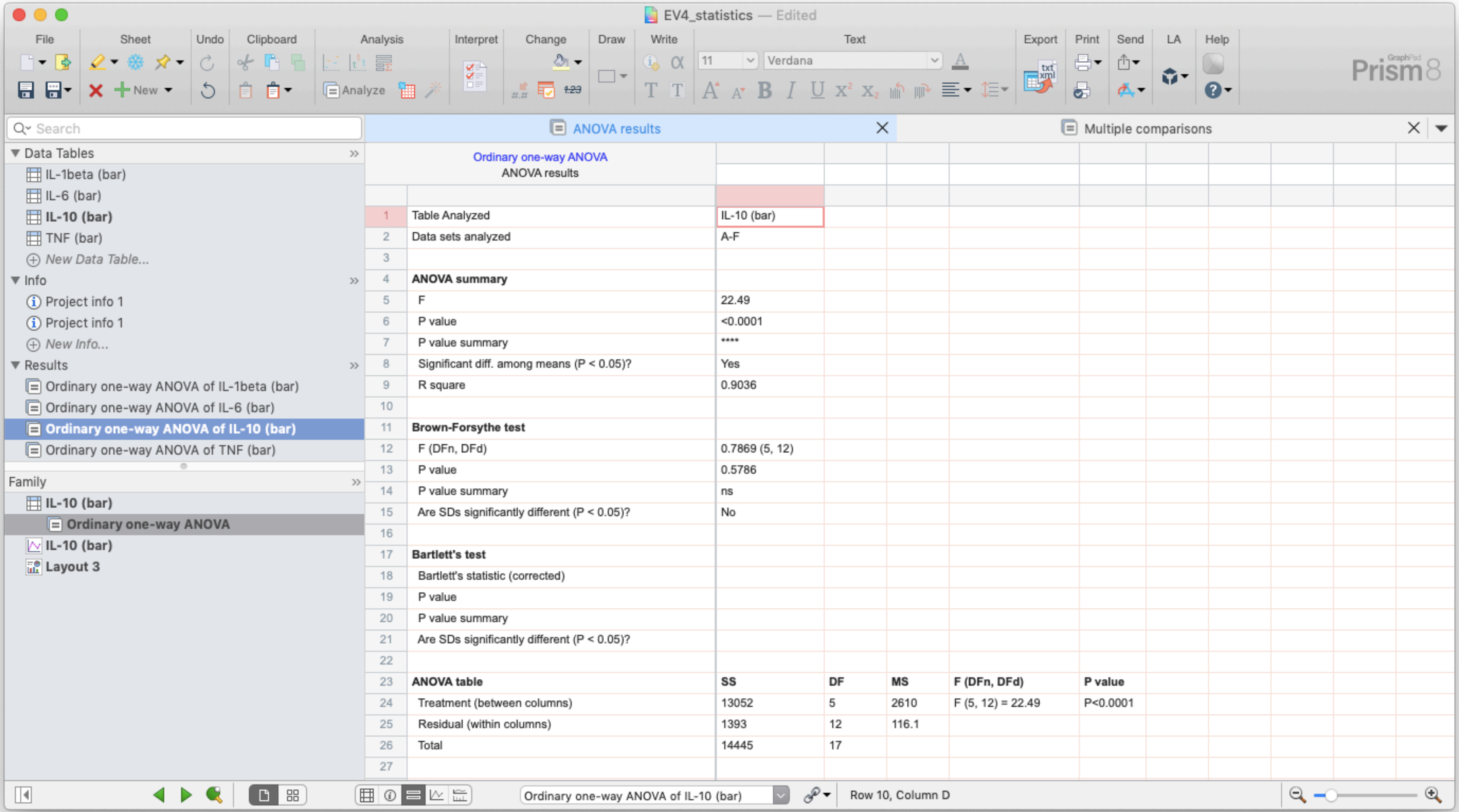

1	Table Analyzed	IL-10 (bar)				
2	Data sets analyzed	A-F				
3						
4	**ANOVA summary**					
5	F	22.49				
6	P value	<0.0001				
7	P value summary	****				
8	Significant diff. among means (P < 0.05)?	Yes				
9	R square	0.9036				
10						
11	**Brown-Forsythe test**					
12	F (DFn, DFd)	0.7869 (5, 12)				
13	P value	0.5786				
14	P value summary	ns				
15	Are SDs significantly different (P < 0.05)?	No				
16						
17	**Bartlett's test**					
18	Bartlett's statistic (corrected)					
19	P value					
20	P value summary					
21	Are SDs significantly different (P < 0.05)?					
22						
23	**ANOVA table**	**SS**	**DF**	**MS**	**F (DFn, DFd)**	**P value**
24	Treatment (between columns)	13052	5	2610	F (5, 12) = 22.49	P<0.0001
25	Residual (within columns)	1393	12	116.1		
26	Total	14445	17			
27						

EV4_statistics — Edited

Data Tables
- IL-1beta (bar)
- IL-6 (bar)
- **IL-10 (bar)**
- TNF (bar)
- New Data Table...

Info
- Project info 1
- Project info 1
- New Info...

Results
- Ordinary one-way ANOVA of IL-1beta (bar)
- Ordinary one-way ANOVA of IL-6 (bar)
- **Ordinary one-way ANOVA of IL-10 (bar)**
- Ordinary one-way ANOVA of TNF (bar)

Family
- **IL-10 (bar)**
  - **Ordinary one-way ANOVA**
- **IL-10 (bar)**
- **Layout 3**

ANOVA results | Multiple comparisons

Ordinary one-way ANOVA
Multiple comparisons

1	Number of families	1							
2	Number of comparisons per	5							
3	Alpha	0.05							
4									
5	**Dunnett's multiple compar**	**Mean Diff.**	**95.00% CI of diff.**	**Significant?**	**Summary**	**Adjusted P Value**	**A-?**		
6	Control vs. 0.06	-8.130	-33.65 to 17.39	No	ns	0.8195	B	0.06	
7	Control vs. 0.12	-19.94	-45.46 to 5.577	No	ns	0.1474	C	0.12	
8	Control vs. 0.24	-38.95	-64.47 to -13.43	Yes	**	0.0034	D	0.24	
9	Control vs. 0.48	-80.35	-105.9 to -54.83	Yes	****	<0.0001	E	0.48	
10	Control vs. LPS	-47.04	-72.56 to -21.52	Yes	***	0.0008	F	LPS	
11									
12	**Test details**	**Mean 1**	**Mean 2**	**Mean Diff.**	**SE of diff.**	**n1**	**n2**	**q**	**DF**
13	Control vs. 0.06	0.000	8.130	-8.130	8.796	3	3	0.9243	12
14	Control vs. 0.12	0.000	19.94	-19.94	8.796	3	3	2.267	12
15	Control vs. 0.24	0.000	38.95	-38.95	8.796	3	3	4.428	12
16	Control vs. 0.48	0.000	80.35	-80.35	8.796	3	3	9.135	12
17	Control vs. LPS	0.000	47.04	-47.04	8.796	3	3	5.348	12

Ordinary one-way ANOVA of IL-10 (bar) — Row 10, Column D

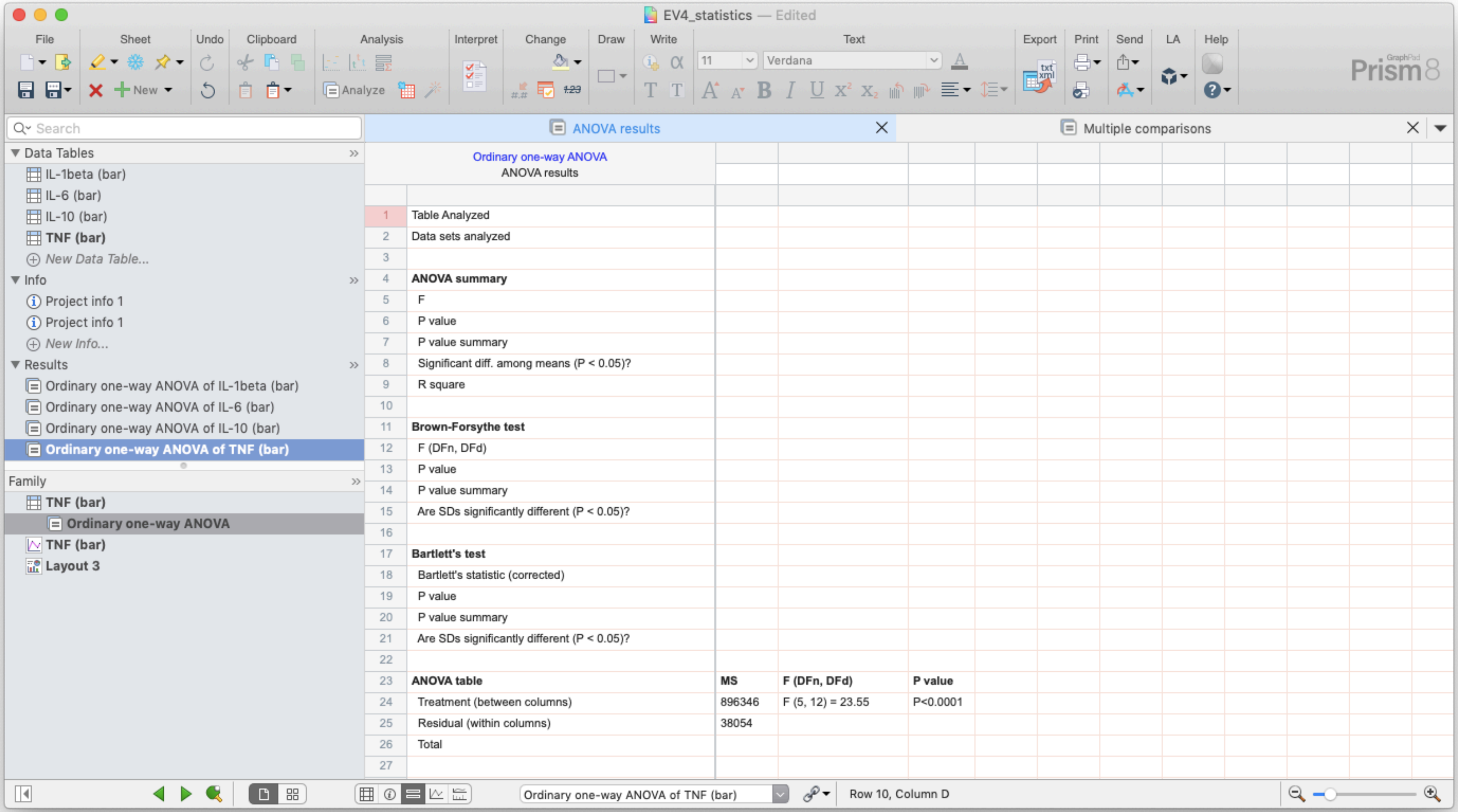
EV4_statistics — Edited
File
Sheet
Undo
Clipboard
Analysis
Interpret
Change
Draw
Write
Text
Export
Print
Send
LA
Help
New
Analyze
Verdana
Prism 8
Search
ANOVA results
Multiple comparisons
Data Tables
IL-1beta (bar)
IL-6 (bar)
IL-10 (bar)
TNF (bar)
New Data Table...
Info
Project info 1
Project info 1
New Info...
Results
Ordinary one-way ANOVA of IL-1beta (bar)
Ordinary one-way ANOVA of IL-6 (bar)
Ordinary one-way ANOVA of IL-10 (bar)
Ordinary one-way ANOVA of TNF (bar)
Family
TNF (bar)
Ordinary one-way ANOVA
TNF (bar)
Layout 3
Ordinary one-way ANOVA
ANOVA results
1 Table Analyzed
2 Data sets analyzed
3
4 ANOVA summary
5 F
6 P value
7 P value summary
8 Significant diff. among means (P < 0.05)?
9 R square
10
11 Brown-Forsythe test
12 F (DFn, DFd)
13 P value
14 P value summary
15 Are SDs significantly different (P < 0.05)?
16
17 Bartlett's test
18 Bartlett's statistic (corrected)
19 P value
20 P value summary
21 Are SDs significantly different (P < 0.05)?
22
23 ANOVA table MS F (DFn, DFd) P value
24 Treatment (between columns) 896346 F (5, 12) = 23.55 P<0.0001
25 Residual (within columns) 38054
26 Total
27
Ordinary one-way ANOVA of TNF (bar)
Row 10, Column D

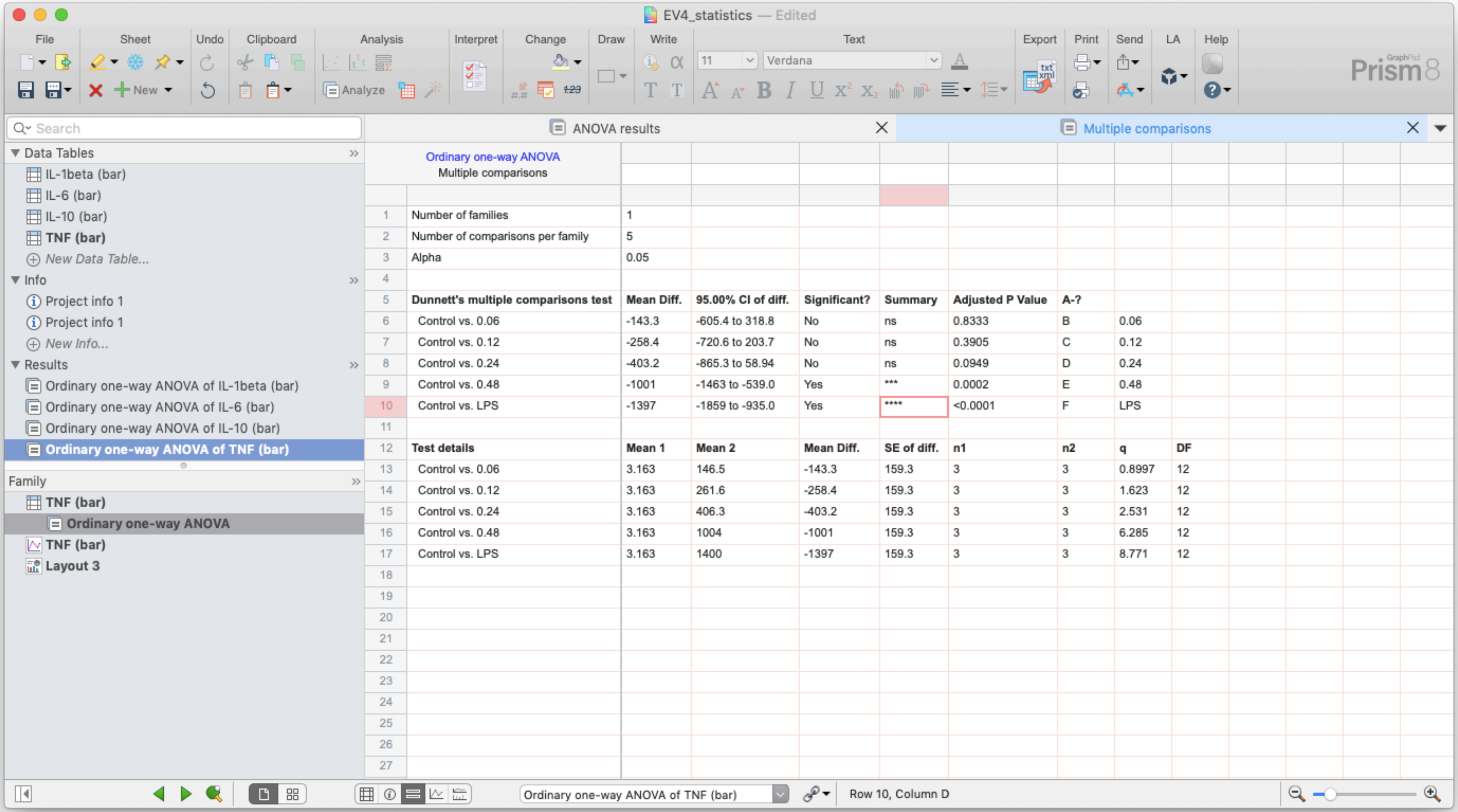

Ordinary one-way ANOVA
Multiple comparisons

1	Number of families	1							
2	Number of comparisons per family	5							
3	Alpha	0.05							
4									
5	**Dunnett's multiple comparisons test**	**Mean Diff.**	**95.00% CI of diff.**	**Significant?**	**Summary**	**Adjusted P Value**	**A-?**		
6	Control vs. 0.06	-143.3	-605.4 to 318.8	No	ns	0.8333	B	0.06	
7	Control vs. 0.12	-258.4	-720.6 to 203.7	No	ns	0.3905	C	0.12	
8	Control vs. 0.24	-403.2	-865.3 to 58.94	No	ns	0.0949	D	0.24	
9	Control vs. 0.48	-1001	-1463 to -539.0	Yes	***	0.0002	E	0.48	
10	Control vs. LPS	-1397	-1859 to -935.0	Yes	****	<0.0001	F	LPS	
11									
12	**Test details**	**Mean 1**	**Mean 2**	**Mean Diff.**	**SE of diff.**	**n1**	**n2**	**q**	**DF**
13	Control vs. 0.06	3.163	146.5	-143.3	159.3	3	3	0.8997	12
14	Control vs. 0.12	3.163	261.6	-258.4	159.3	3	3	1.623	12
15	Control vs. 0.24	3.163	406.3	-403.2	159.3	3	3	2.531	12
16	Control vs. 0.48	3.163	1004	-1001	159.3	3	3	6.285	12
17	Control vs. LPS	3.163	1400	-1397	159.3	3	3	8.771	12
