# Supplementary material for: Malaria inflammation by xanthine oxidase‐produced reactive oxygen species
Source: EMBO Mol Med. 2019 Jul 2;11(8):e9903. doi: 10.15252/emmm.201809903 (PMC6685105; doi:10.15252/emmm.201809903)
Supplement: Supplementary file 2 — Source Data for Appendix [file EMMM-11-e9903-s008.zip › EV_source_data/Source_Data_Appendix_Fig_S5.pdf]

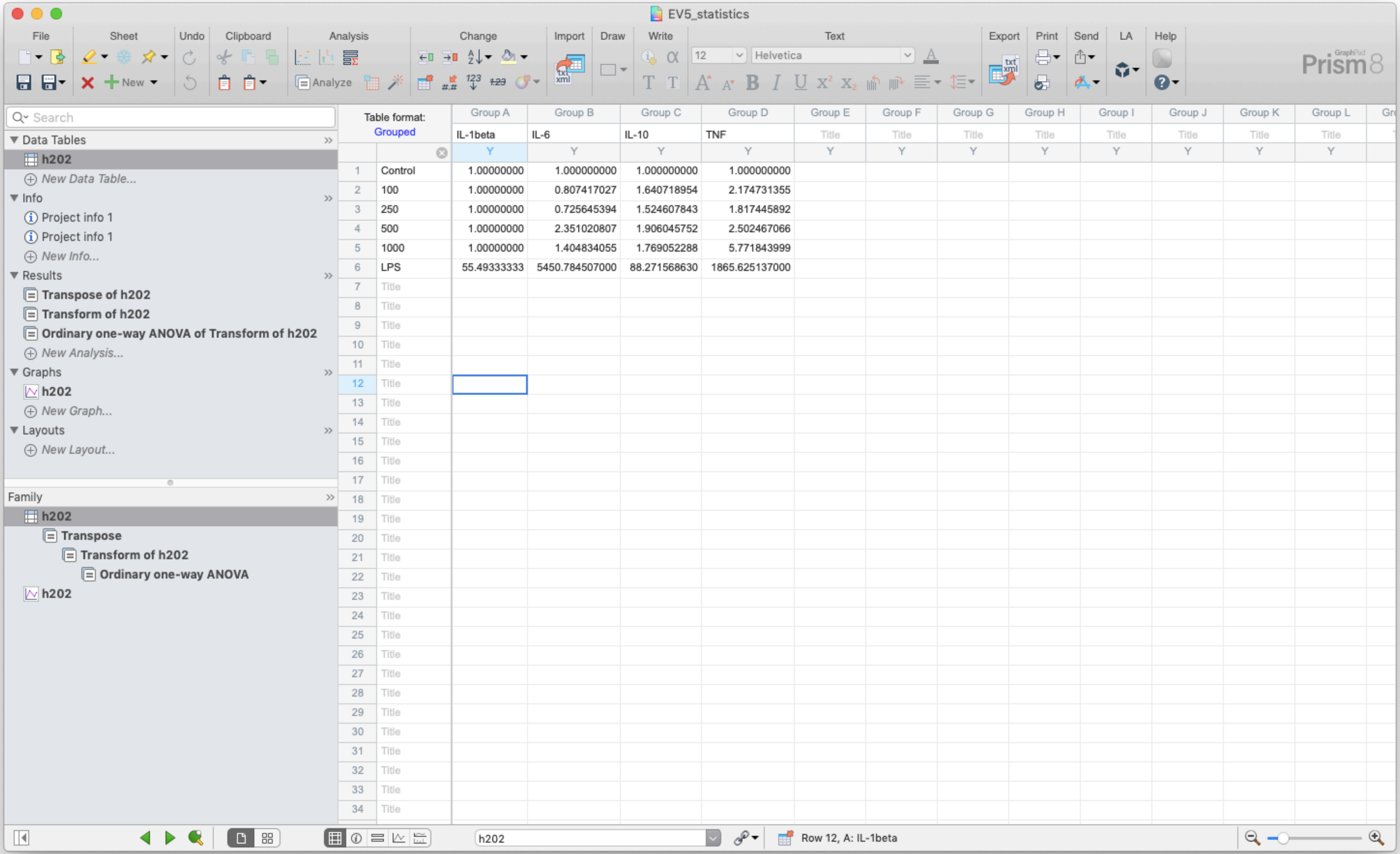

		Group A	Group B	Group C	Group D
		IL-1beta	IL-6	IL-10	TNF
		Y	Y	Y	Y
1	Control	1.00000000	1.000000000	1.000000000	1.000000000
2	100	1.00000000	0.807417027	1.640718954	2.174731355
3	250	1.00000000	0.725645394	1.524607843	1.817445892
4	500	1.00000000	2.351020807	1.906045752	2.502467066
5	1000	1.00000000	1.404834055	1.769052288	5.771843999
6	LPS	55.49333333	5450.784507000	88.271568630	1865.625137000

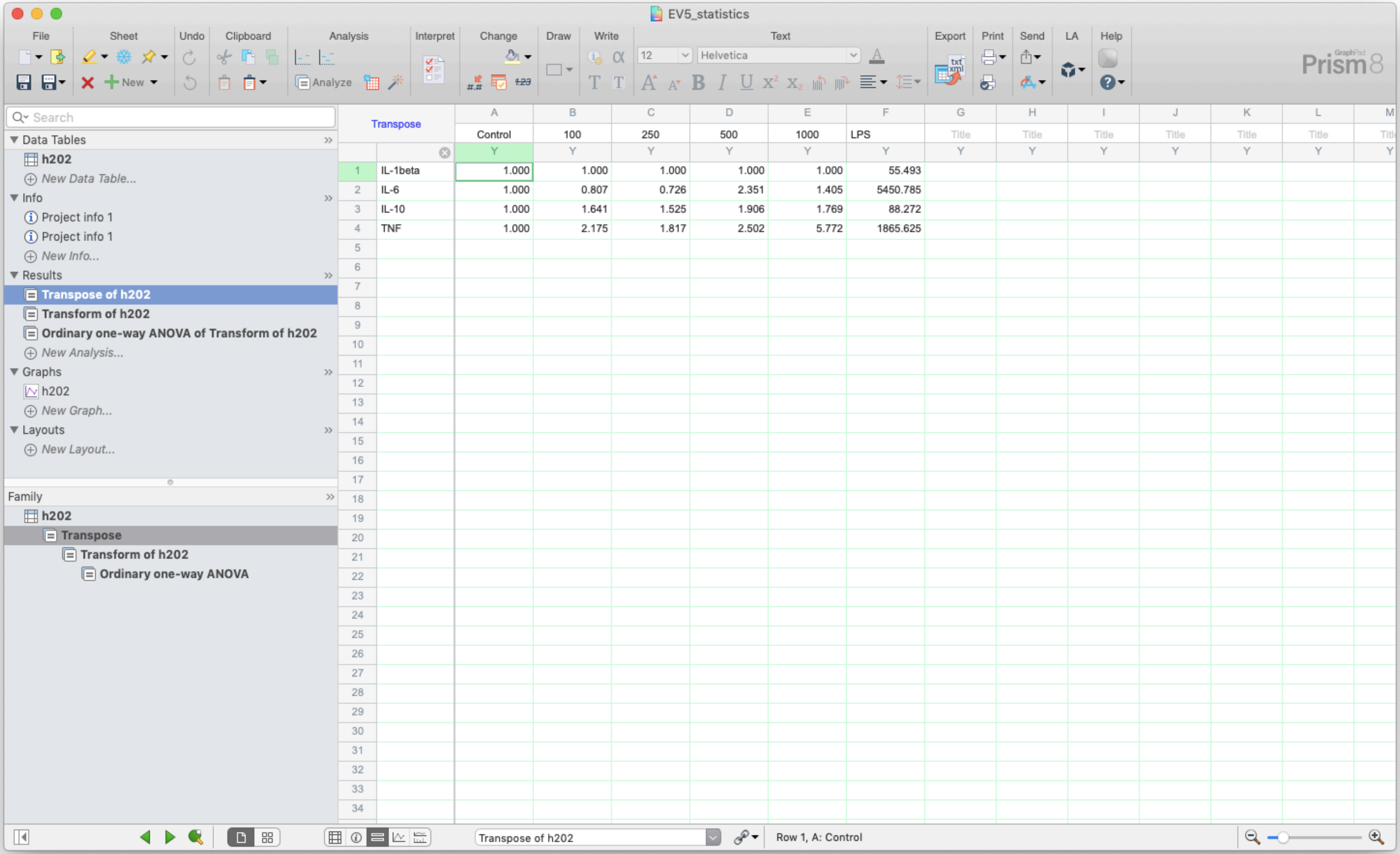
EV5_statistics
File
Sheet
Undo
Clipboard
Analysis
Interpret
Change
Draw
Write
Text
Export
Print
Send
LA
Help
Prism 8
Analyze
New
Search
Data Tables
h202
New Data Table...
Info
Project info 1
Project info 1
New Info...
Results
Transpose of h202
Transform of h202
Ordinary one-way ANOVA of Transform of h202
New Analysis...
Graphs
h202
New Graph...
Layouts
New Layout...
Family
h202
Transpose
Transform of h202
Ordinary one-way ANOVA
Transpose
	Control	100	250	500	1000	LPS
IL-1beta	1.000	1.000	1.000	1.000	1.000	55.493
IL-6	1.000	0.807	0.726	2.351	1.405	5450.785
IL-10	1.000	1.641	1.525	1.906	1.769	88.272
TNF	1.000	2.175	1.817	2.502	5.772	1865.625
Transpose of h202
Row 1, A: Control

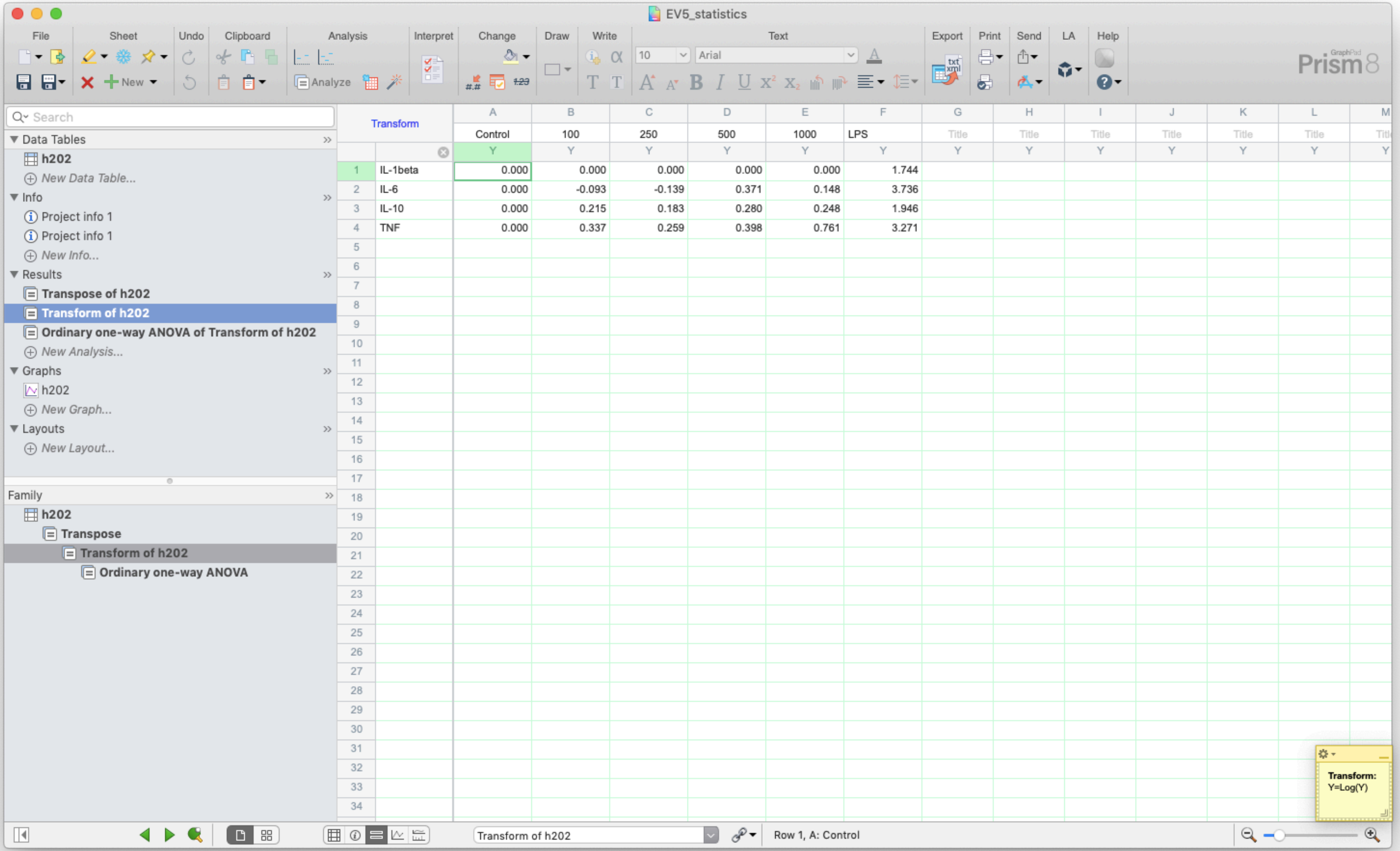

Transform	Control	100	250	500	1000	LPS
IL-1beta	0.000	0.000	0.000	0.000	0.000	1.744
IL-6	0.000	-0.093	-0.139	0.371	0.148	3.736
IL-10	0.000	0.215	0.183	0.280	0.248	1.946
TNF	0.000	0.337	0.259	0.398	0.761	3.271

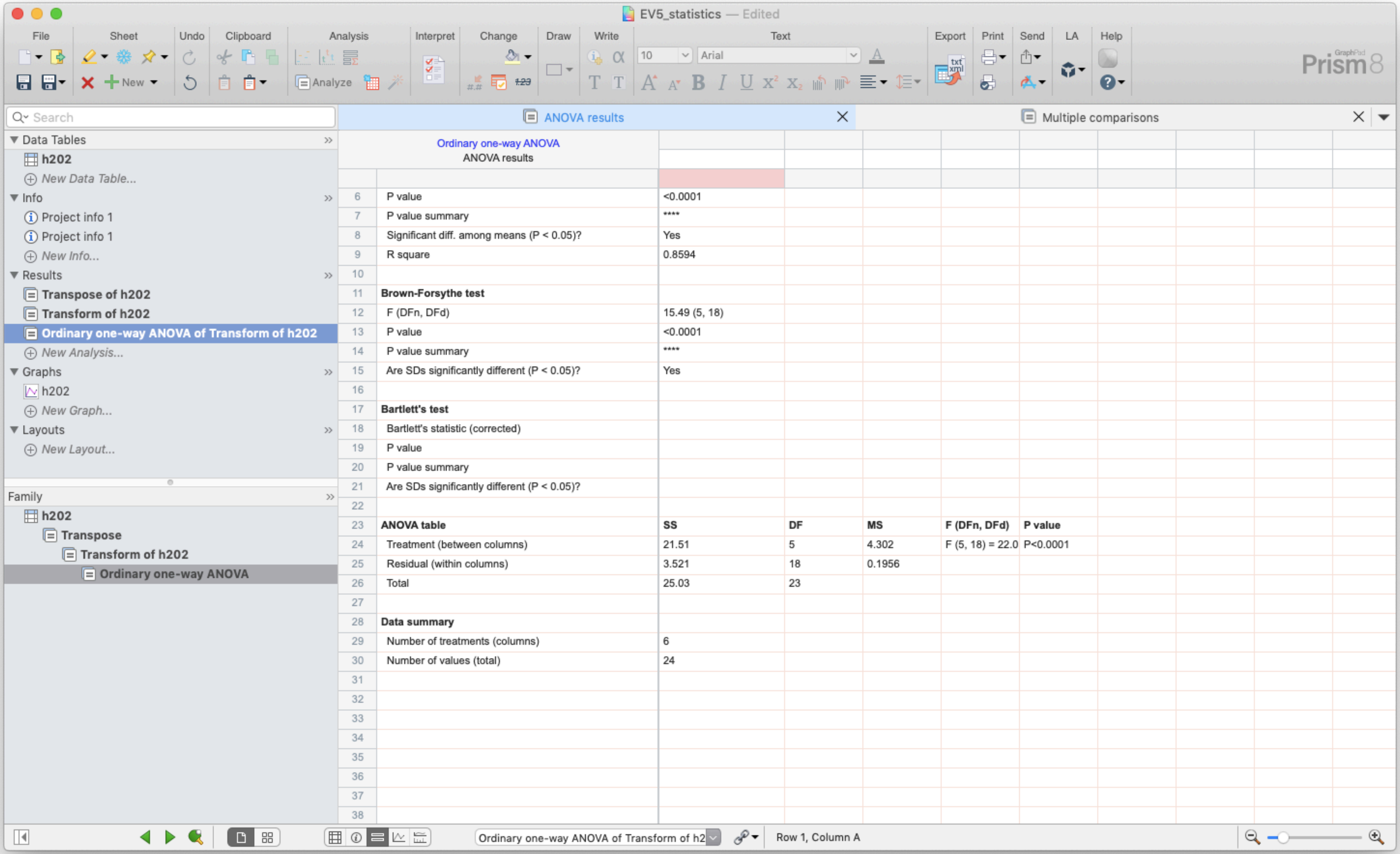
EV5_statistics — Edited
Data Tables
h202
New Data Table...
Info
Project info 1
Project info 1
New Info...
Results
Transpose of h202
Transform of h202
Ordinary one-way ANOVA of Transform of h202
New Analysis...
Graphs
h202
New Graph...
Layouts
New Layout...
Family
h202
Transpose
Transform of h202
Ordinary one-way ANOVA
ANOVA results
Multiple comparisons
Ordinary one-way ANOVA
ANOVA results
6 P value <0.0001
7 P value summary ****
8 Significant diff. among means (P < 0.05)? Yes
9 R square 0.8594
11 Brown-Forsythe test
12 F (DFn, DFd) 15.49 (5, 18)
13 P value <0.0001
14 P value summary ****
15 Are SDs significantly different (P < 0.05)? Yes
17 Bartlett's test
18 Bartlett's statistic (corrected)
19 P value
20 P value summary
21 Are SDs significantly different (P < 0.05)?
23 ANOVA table SS DF MS F (DFn, DFd) P value
24 Treatment (between columns) 21.51 5 4.302 F (5, 18) = 22.0 P<0.0001
25 Residual (within columns) 3.521 18 0.1956
26 Total 25.03 23
28 Data summary
29 Number of treatments (columns) 6
30 Number of values (total) 24
Ordinary one-way ANOVA of Transform of h2
Row 1, Column A

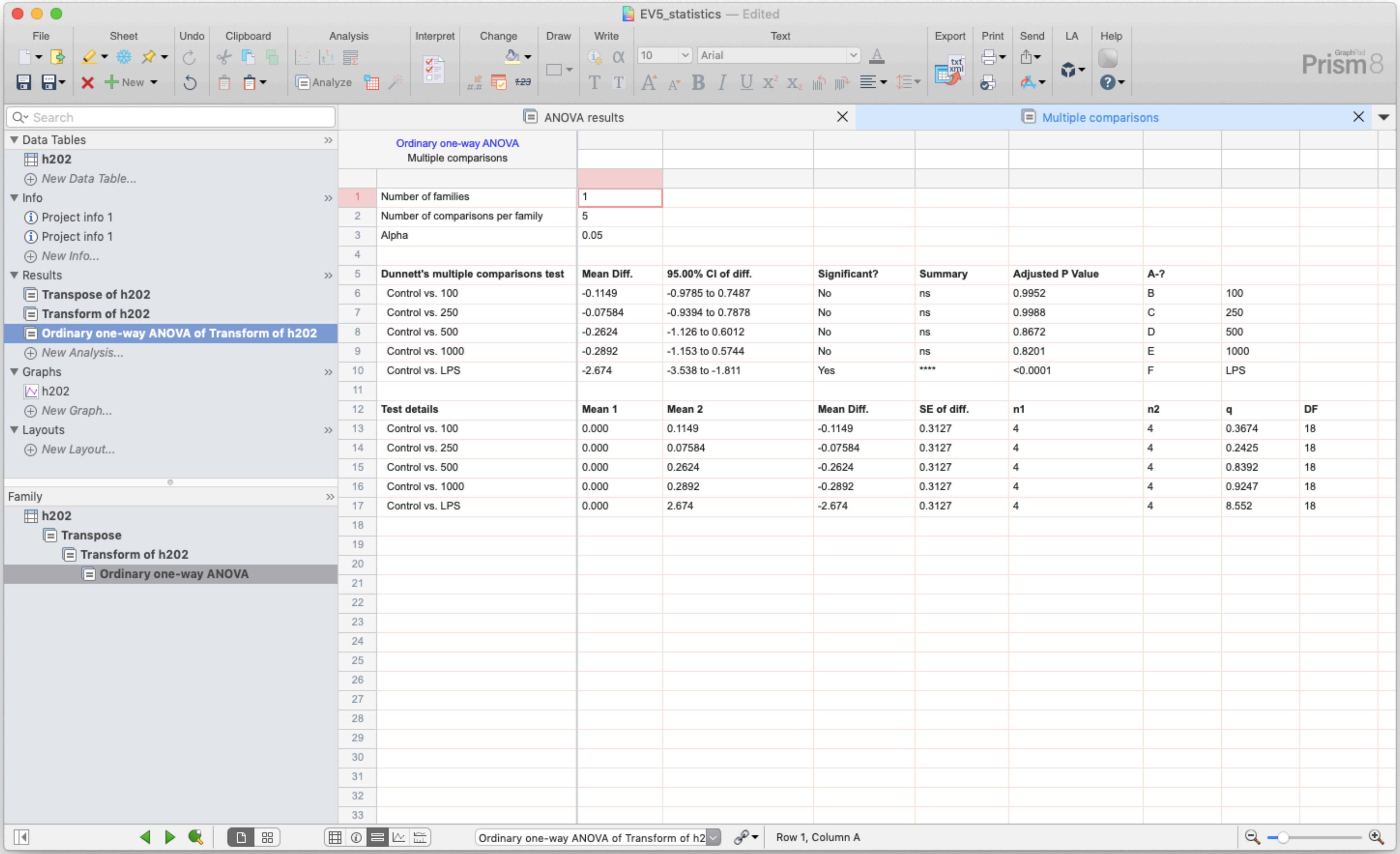

EV5_statistics — Edited

Ordinary one-way ANOVA
Multiple comparisons

1	Number of families	1							
2	Number of comparisons per family	5							
3	Alpha	0.05							
4									
5	**Dunnett's multiple comparisons test**	**Mean Diff.**	**95.00% CI of diff.**	**Significant?**	**Summary**	**Adjusted P Value**	**A-?**		
6	Control vs. 100	-0.1149	-0.9785 to 0.7487	No	ns	0.9952	B	100	
7	Control vs. 250	-0.07584	-0.9394 to 0.7878	No	ns	0.9988	C	250	
8	Control vs. 500	-0.2624	-1.126 to 0.6012	No	ns	0.8672	D	500	
9	Control vs. 1000	-0.2892	-1.153 to 0.5744	No	ns	0.8201	E	1000	
10	Control vs. LPS	-2.674	-3.538 to -1.811	Yes	****	<0.0001	F	LPS	
11									
12	**Test details**	**Mean 1**	**Mean 2**	**Mean Diff.**	**SE of diff.**	**n1**	**n2**	**q**	**DF**
13	Control vs. 100	0.000	0.1149	-0.1149	0.3127	4	4	0.3674	18
14	Control vs. 250	0.000	0.07584	-0.07584	0.3127	4	4	0.2425	18
15	Control vs. 500	0.000	0.2624	-0.2624	0.3127	4	4	0.8392	18
16	Control vs. 1000	0.000	0.2892	-0.2892	0.3127	4	4	0.9247	18
17	Control vs. LPS	0.000	2.674	-2.674	0.3127	4	4	8.552	18

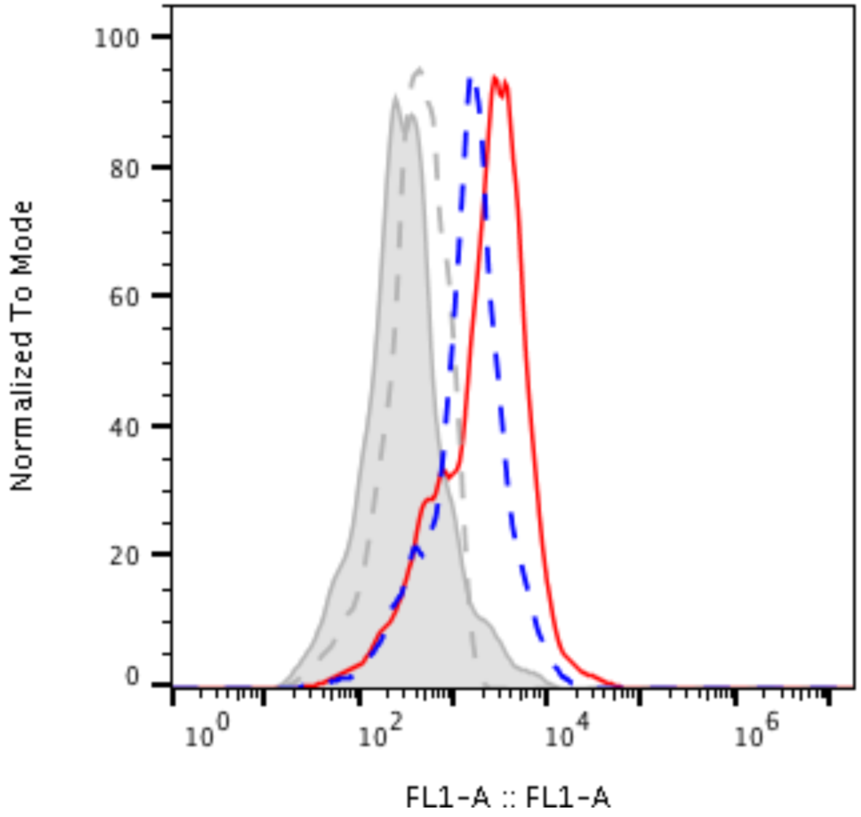

	Sample Name	Subset Name	Count
	D03 XO irbcL.fcs	macrophage	9676
	D02 XO rbcL.fcs	macrophage	4328
	C03 irbcL.fcs	macrophage	9047
	C02 rbcL.fcs	macrophage	1228
