# Supplementary material for: Malaria inflammation by xanthine oxidase‐produced reactive oxygen species
Source: EMBO Mol Med. 2019 Jul 2;11(8):e9903. doi: 10.15252/emmm.201809903 (PMC6685105; doi:10.15252/emmm.201809903)
Supplement: Supplementary file 2 — Source Data for Appendix [file EMMM-11-e9903-s008.zip › EV_source_data/Source_Data_Appendix_Fig_S6.pdf]

EV8_barplot

File | Sheet | Undo | Clipboard | Analysis | Change | Import | Draw | Write | Text | Export | Print | Send | LA | Help

Search

Data Tables
- rt_pcr
- mRNA fold change
- New Data Table...

Info
- Project info 1
- New Info...

Results
- New Analysis...

Graphs
- mRNA fold change
- New Graph...

Layouts
- New Layout...

Family
- rt_pcr

Table format: Column

		Group A	Group B	Group C	Group D	Group E	Group F	Group G	Group H	Group I	Group J	Group K
		CT	Ct	Ct Average	Ct h18SRNA	Delta Ct	2^(-Delta Ct)	Fold Change	Title	Title	Title	Title
		Y	Y	Y	Y	Y	Y	Y	Y	Y	Y	Y
1	AC siRNA 488	28.548000000	28.608	28.578	15.672	12.906	0.0001303280	1.000000000				
2	AC NLRP3-1	29.602000000	29.278	29.440	15.583	13.857	0.0000674048	0.517193554				
3	AC NLRP3-2	29.387000000	29.339	29.363	15.775	13.588	0.0000812309	0.623280545				
4	Title											
5	Title											
6	Title											
7	Title											
8	Title											
9	Title											
10	Title											
11	Title											
12	Title											
13	Title											
14	Title											
15	Title											
16	Title											
17	Title											
18	Title											
19	Title											
20	Title											
21	Title											
22	Title											
23	Title											
24	Title											
25	Title											
26	Title											
27	Title											
28	Title											
29	Title											

rt_pcr — Row 8, B: Ct

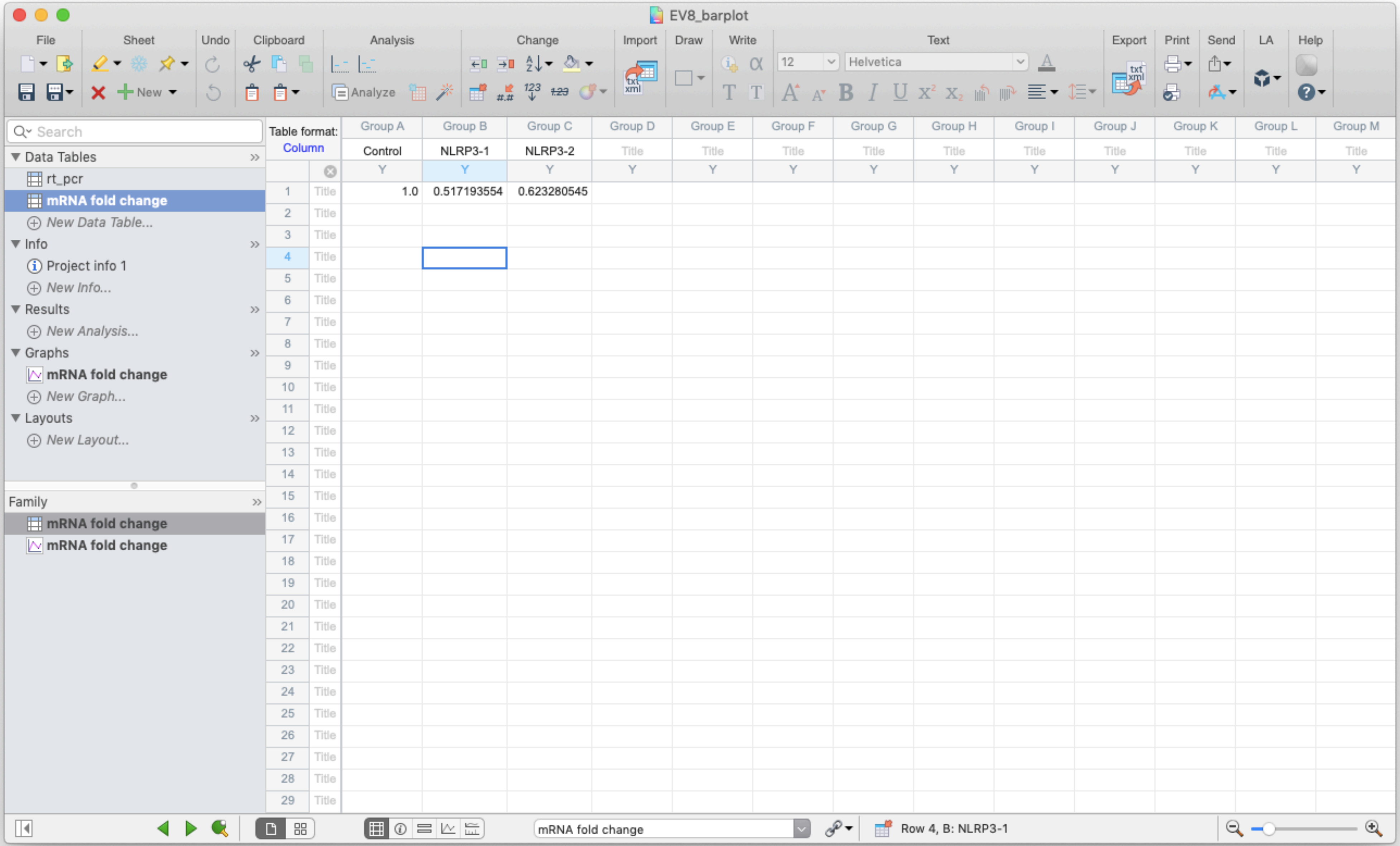
EV8_barplot
File
Sheet
Undo
Clipboard
Analysis
Change
Import
Draw
Write
Text
Export
Print
Send
LA
Help
New
Analyze
12
Helvetica
Search
Data Tables
rt_pcr
mRNA fold change
New Data Table...
Info
Project info 1
New Info...
Results
New Analysis...
Graphs
mRNA fold change
New Graph...
Layouts
New Layout...
Family
mRNA fold change
mRNA fold change
Table format:
Column
Group A Group B Group C Group D Group E Group F Group G Group H Group I Group J Group K Group L Group M
Control NLRP3-1 NLRP3-2
1.0 0.517193554 0.623280545
mRNA fold change
Row 4, B: NLRP3-1
