# Supplementary material for: Malaria inflammation by xanthine oxidase‐produced reactive oxygen species
Source: EMBO Mol Med. 2019 Jul 2;11(8):e9903. doi: 10.15252/emmm.201809903 (PMC6685105; doi:10.15252/emmm.201809903)
Supplement: Supplementary file 2 — Source Data for Appendix [file EMMM-11-e9903-s008.zip › EV_source_data/Source_Data_Appendix_Fig_S7.pdf]

EV9_barplots

Data Tables: IL8, CCL5, CXCL9, CCL2

Info: Project info 1, Project info 1

Graphs: IL8, CCL5, CXCL9

Family: IL8, IL8

Table format: Grouped

		Group A	Group B	Group C
		Media	siRNA NLRP3	siRNA NLRP3-2
		Y	Y	Y
1	Media	274.050000000	274.050000000	241.920000000
2	rbcL	660.490000000	519.240000000	546.130000000
3	irbcL	570.410000000	463.090000000	550.160000000
4	XO	3529.390000000	2973.990000000	4864.290000000
5	XO+rbcL	4335.010000000	5384.180000000	4602.700000000
6	XO+irbcL	8644.360000000	7612.420000000	5445.860000000

Row 9, A: Media

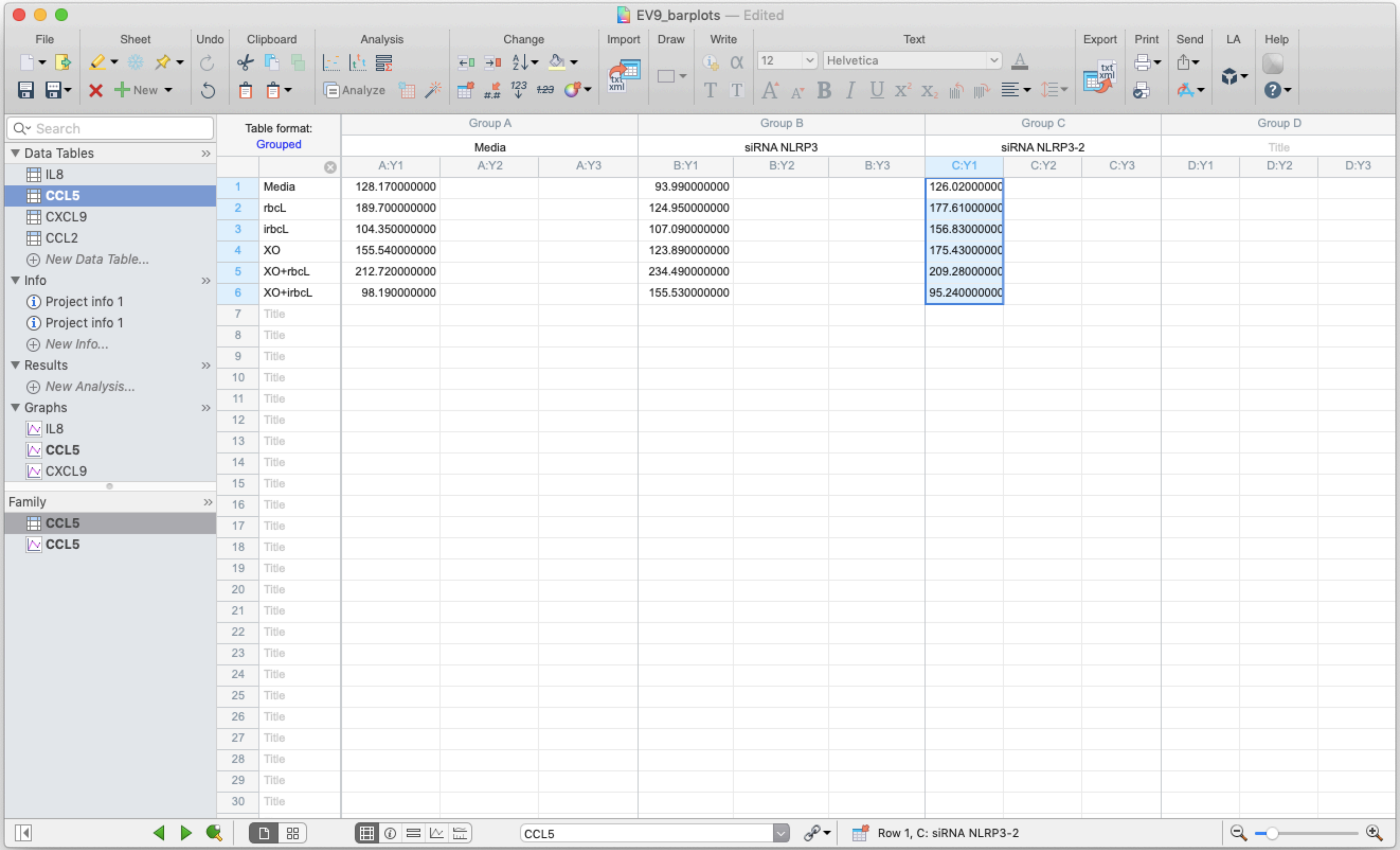

Table format: Grouped	Group A			Group B			Group C			Group D		
	Media			siRNA NLRP3			siRNA NLRP3-2			Title		
	A:Y1	A:Y2	A:Y3	B:Y1	B:Y2	B:Y3	C:Y1	C:Y2	C:Y3	D:Y1	D:Y2	D:Y3
1 Media	128.170000000			93.990000000			126.0200000					
2 rbcL	189.700000000			124.950000000			177.6100000					
3 irbcL	104.350000000			107.090000000			156.8300000					
4 XO	155.540000000			123.890000000			175.4300000					
5 XO+rbcL	212.720000000			234.490000000			209.2800000					
6 XO+irbcL	98.190000000			155.530000000			95.2400000					

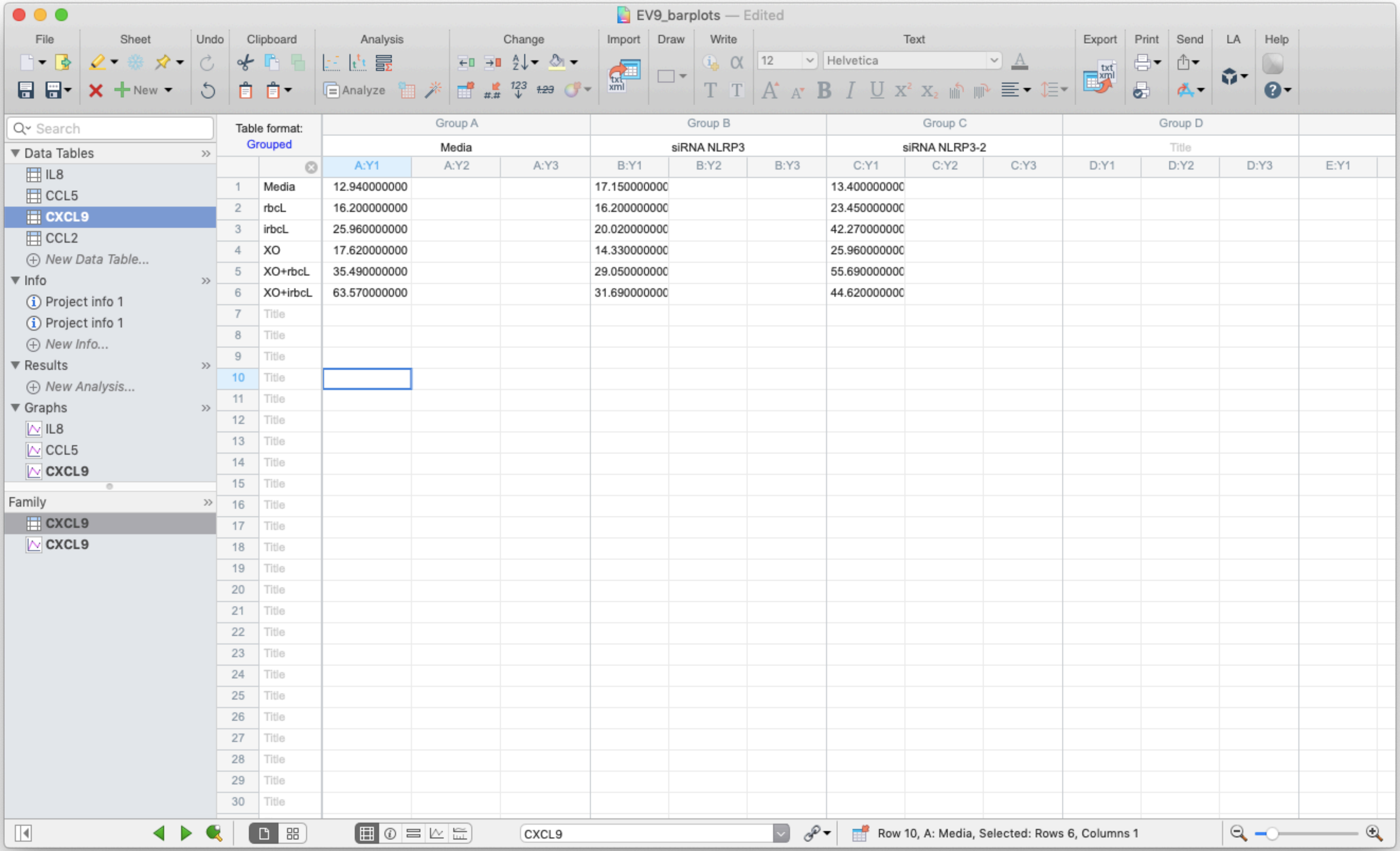

EV9_barplots — Edited

Table format: Grouped

	Group A			Group B			Group C			Group D			
	Media			siRNA NLRP3			siRNA NLRP3-2			Title			
	A:Y1	A:Y2	A:Y3	B:Y1	B:Y2	B:Y3	C:Y1	C:Y2	C:Y3	D:Y1	D:Y2	D:Y3	E:Y1
Media	12.940000000			17.150000000			13.400000000						
rbcL	16.200000000			16.200000000			23.450000000						
irbcL	25.960000000			20.020000000			42.270000000						
XO	17.620000000			14.330000000			25.960000000						
XO+rbcL	35.490000000			29.050000000			55.690000000						
XO+irbcL	63.570000000			31.690000000			44.620000000						

Row 10, A: Media, Selected: Rows 6, Columns 1

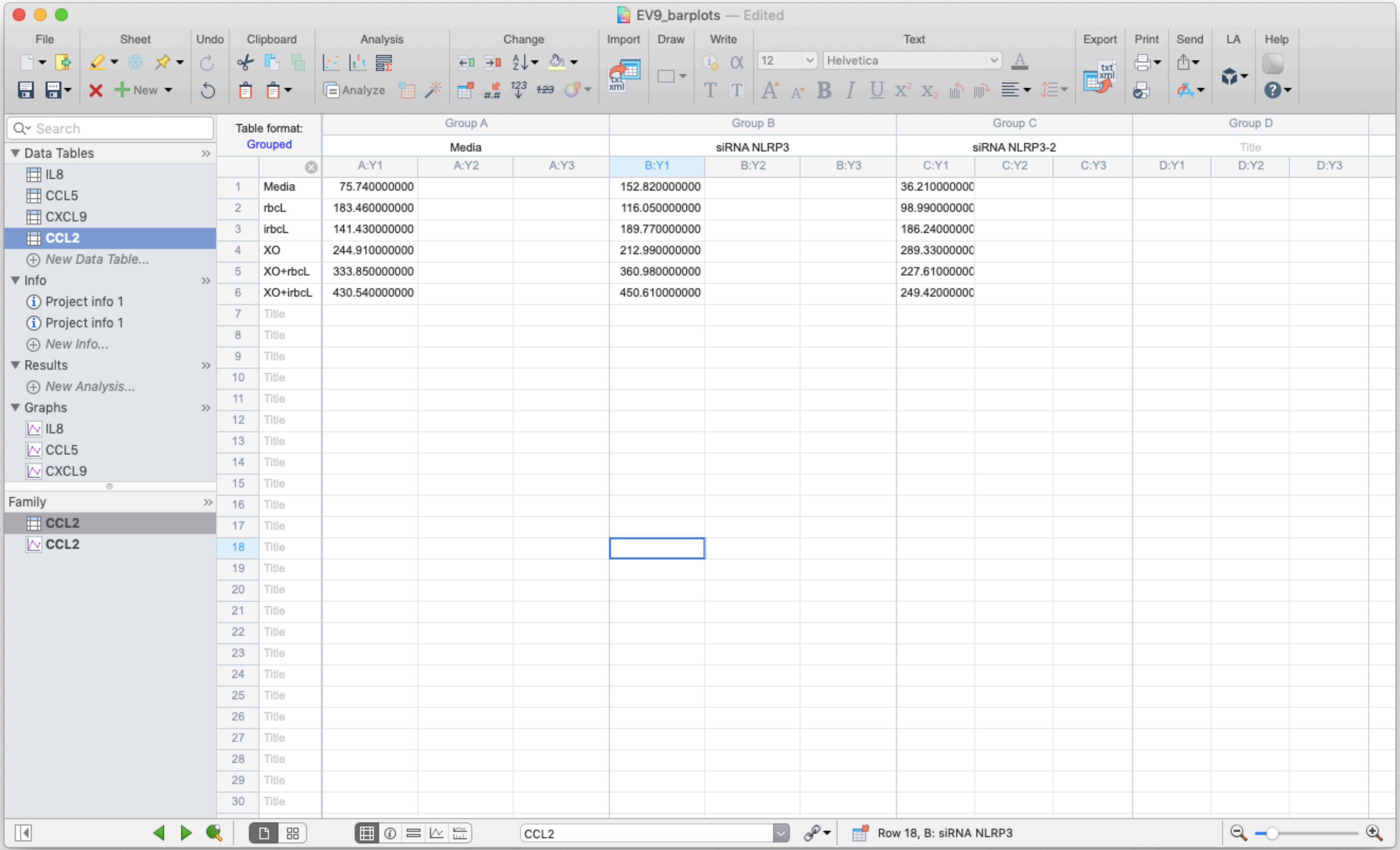

	Group A			Group B			Group C			Group D		
	Media			siRNA NLRP3			siRNA NLRP3-2			Title		
	A:Y1	A:Y2	A:Y3	B:Y1	B:Y2	B:Y3	C:Y1	C:Y2	C:Y3	D:Y1	D:Y2	D:Y3
Media	75.740000000			152.820000000			36.210000000					
rbcL	183.460000000			116.050000000			98.99000000					
irbcL	141.430000000			189.770000000			186.24000000					
XO	244.910000000			212.990000000			289.33000000					
XO+rbcL	333.850000000			360.980000000			227.61000000					
XO+irbcL	430.540000000			450.610000000			249.42000000					
