# Supplementary material for: Malaria inflammation by xanthine oxidase‐produced reactive oxygen species
Source: EMBO Mol Med. 2019 Jul 2;11(8):e9903. doi: 10.15252/emmm.201809903 (PMC6685105; doi:10.15252/emmm.201809903)
Supplement: Supplementary file 4 — Source Data for Figure 1 [file EMMM-11-e9903-s002.pdf]

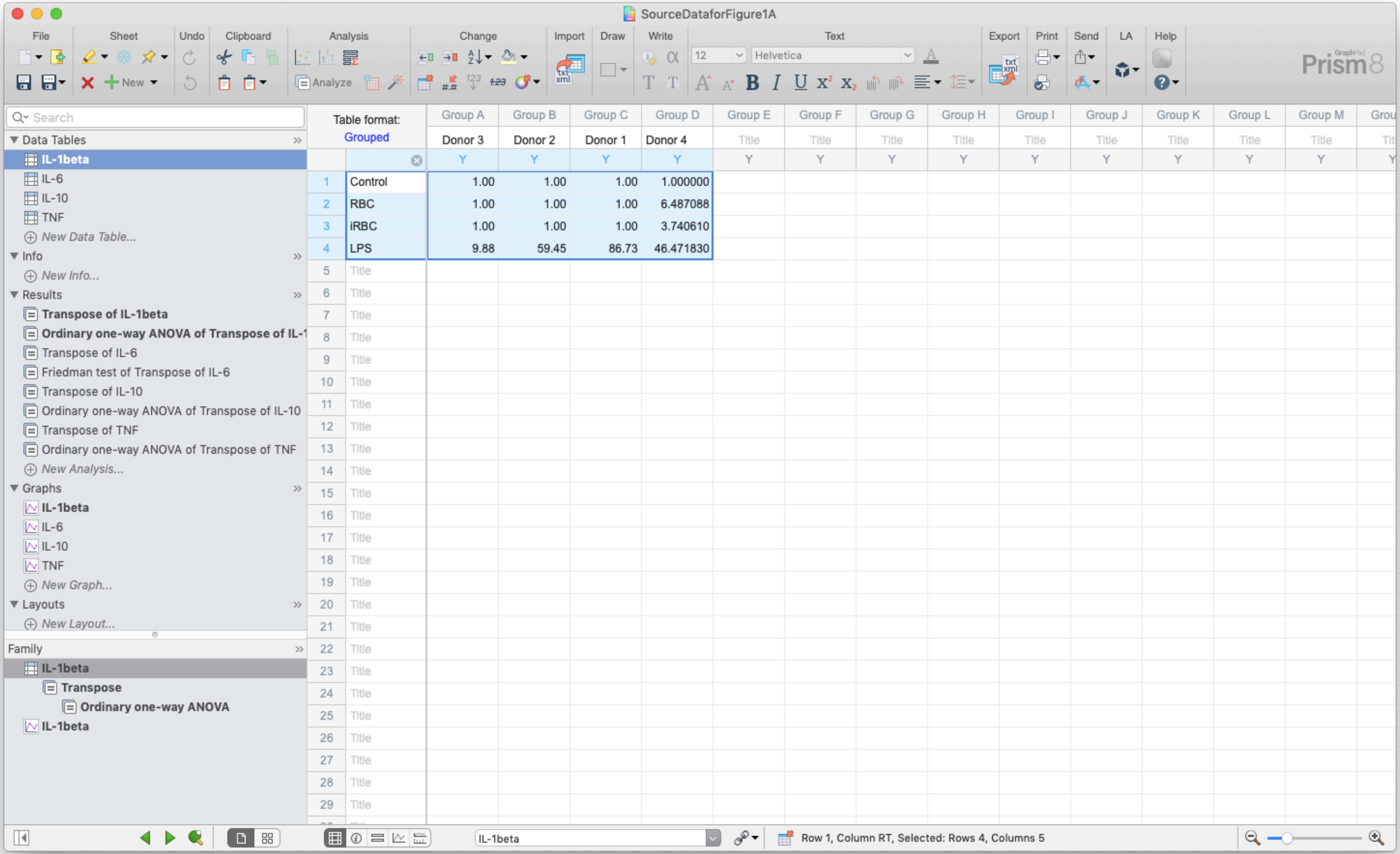

	Group A	Group B	Group C	Group D
	Donor 3	Donor 2	Donor 1	Donor 4
	Y	Y	Y	Y
Control	1.00	1.00	1.00	1.000000
RBC	1.00	1.00	1.00	6.487088
iRBC	1.00	1.00	1.00	3.740610
LPS	9.88	59.45	86.73	46.471830

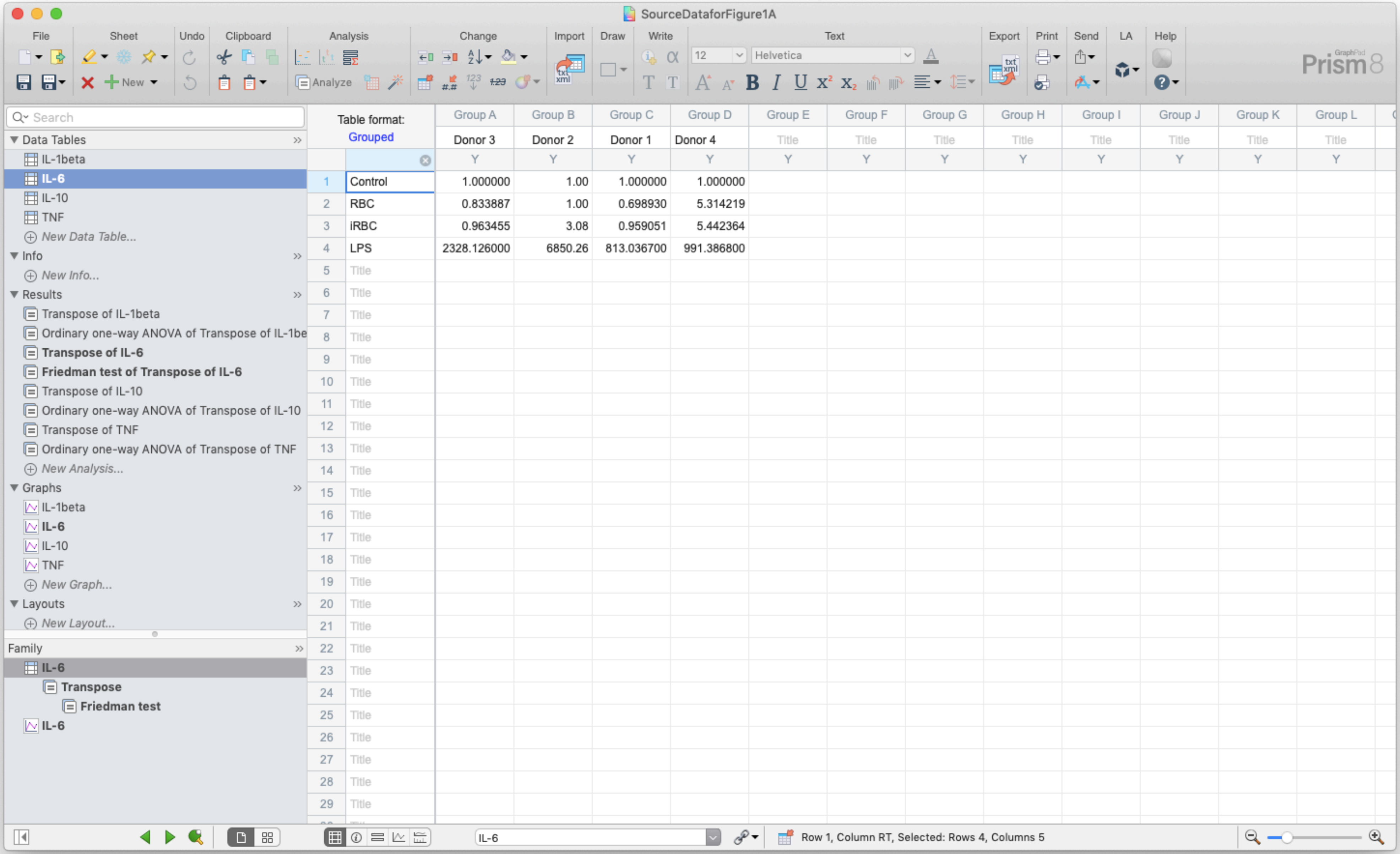

Table format: Grouped	Group A	Group B	Group C	Group D
	Donor 3	Donor 2	Donor 1	Donor 4
	Y	Y	Y	Y
Control	1.000000	1.00	1.000000	1.000000
RBC	0.833887	1.00	0.698930	5.314219
iRBC	0.963455	3.08	0.959051	5.442364
LPS	2328.126000	6850.26	813.036700	991.386800

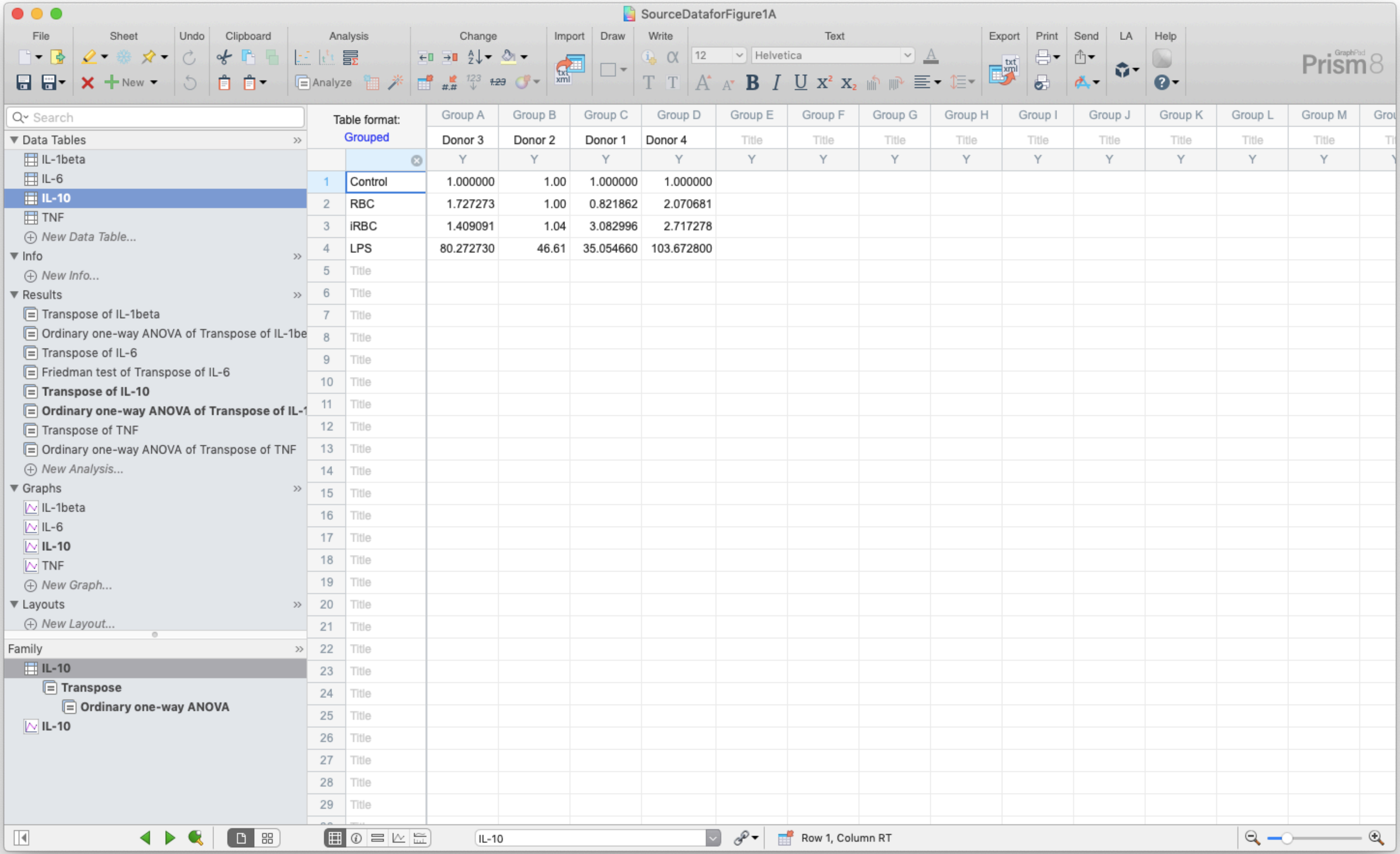

	Group A	Group B	Group C	Group D
	Donor 3	Donor 2	Donor 1	Donor 4
Control	1.000000	1.00	1.000000	1.000000
RBC	1.727273	1.00	0.821862	2.070681
iRBC	1.409091	1.04	3.082996	2.717278
LPS	80.272730	46.61	35.054660	103.672800

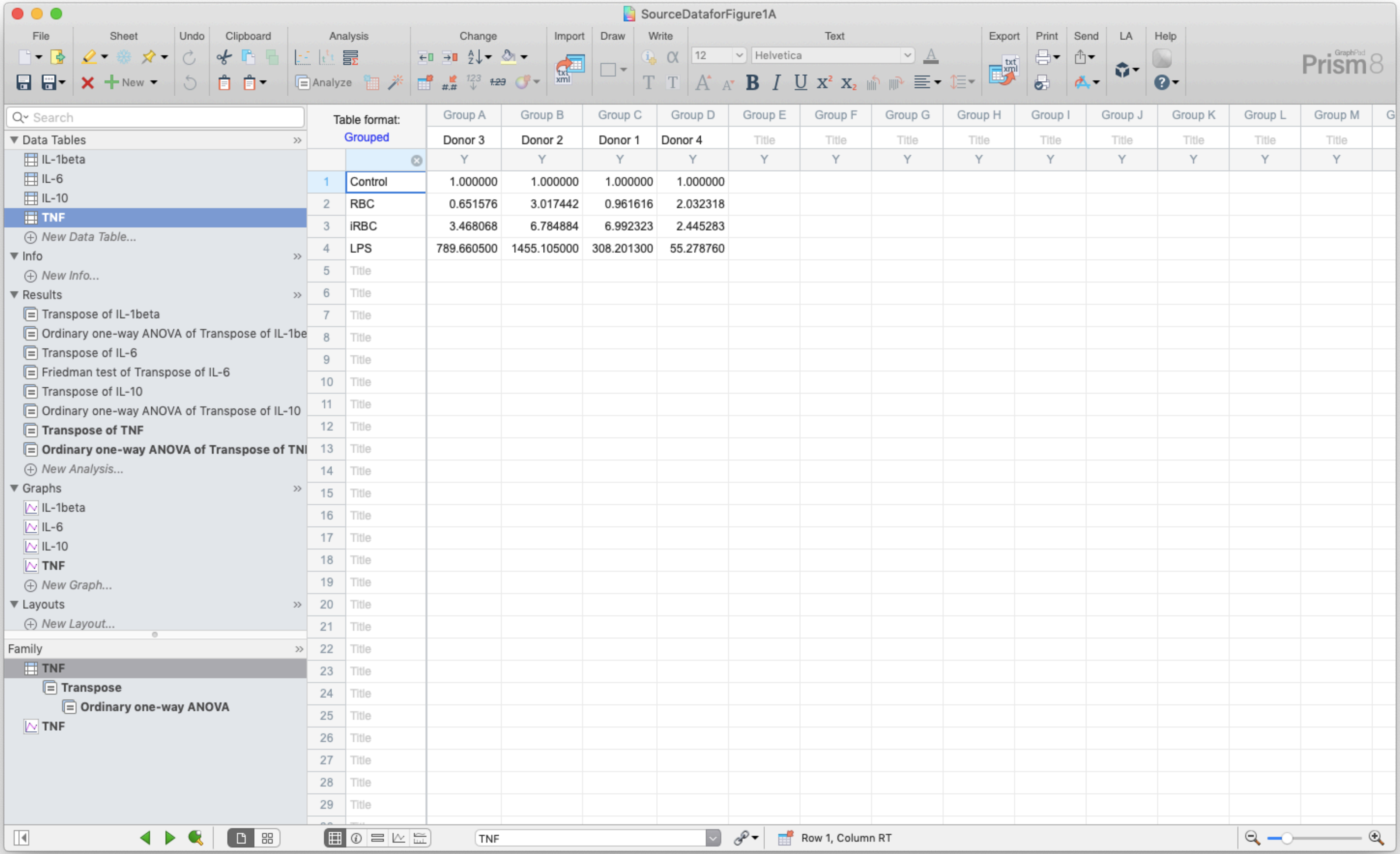

Table format: Grouped

	Group A	Group B	Group C	Group D
	Donor 3	Donor 2	Donor 1	Donor 4
	Y	Y	Y	Y
Control	1.000000	1.000000	1.000000	1.000000
RBC	0.651576	3.017442	0.961616	2.032318
iRBC	3.468068	6.784884	6.992323	2.445283
LPS	789.660500	1455.105000	308.201300	55.278760

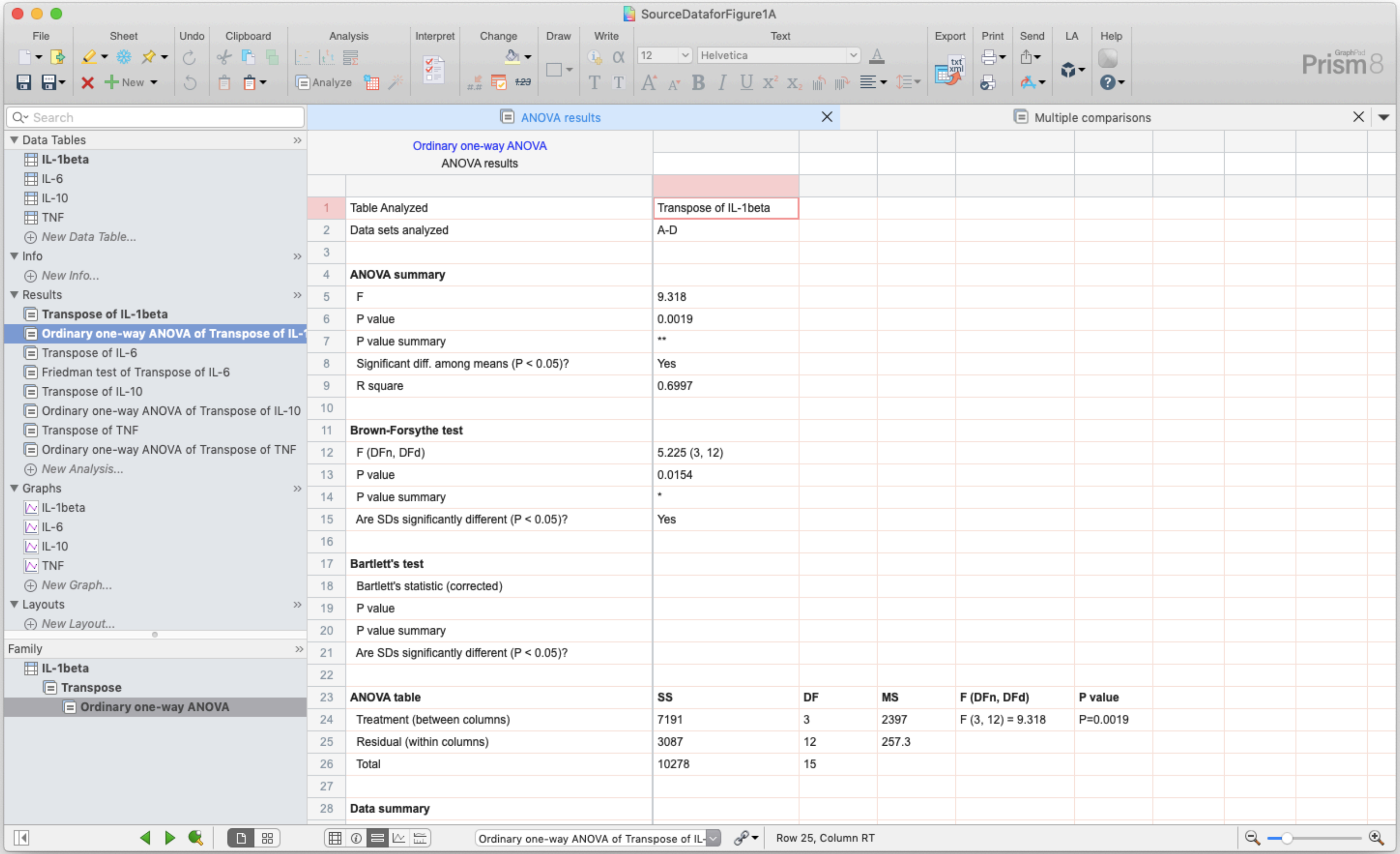

1	Table Analyzed	Transpose of IL-1beta				
2	Data sets analyzed	A-D				
3						
4	**ANOVA summary**					
5	F	9.318				
6	P value	0.0019				
7	P value summary	**				
8	Significant diff. among means (P < 0.05)?	Yes				
9	R square	0.6997				
10						
11	**Brown-Forsythe test**					
12	F (DFn, DFd)	5.225 (3, 12)				
13	P value	0.0154				
14	P value summary	*				
15	Are SDs significantly different (P < 0.05)?	Yes				
16						
17	**Bartlett's test**					
18	Bartlett's statistic (corrected)					
19	P value					
20	P value summary					
21	Are SDs significantly different (P < 0.05)?					
22						
23	**ANOVA table**	**SS**	**DF**	**MS**	**F (DFn, DFd)**	**P value**
24	Treatment (between columns)	7191	3	2397	F (3, 12) = 9.318	P=0.0019
25	Residual (within columns)	3087	12	257.3		
26	Total	10278	15			
27						
28	**Data summary**					

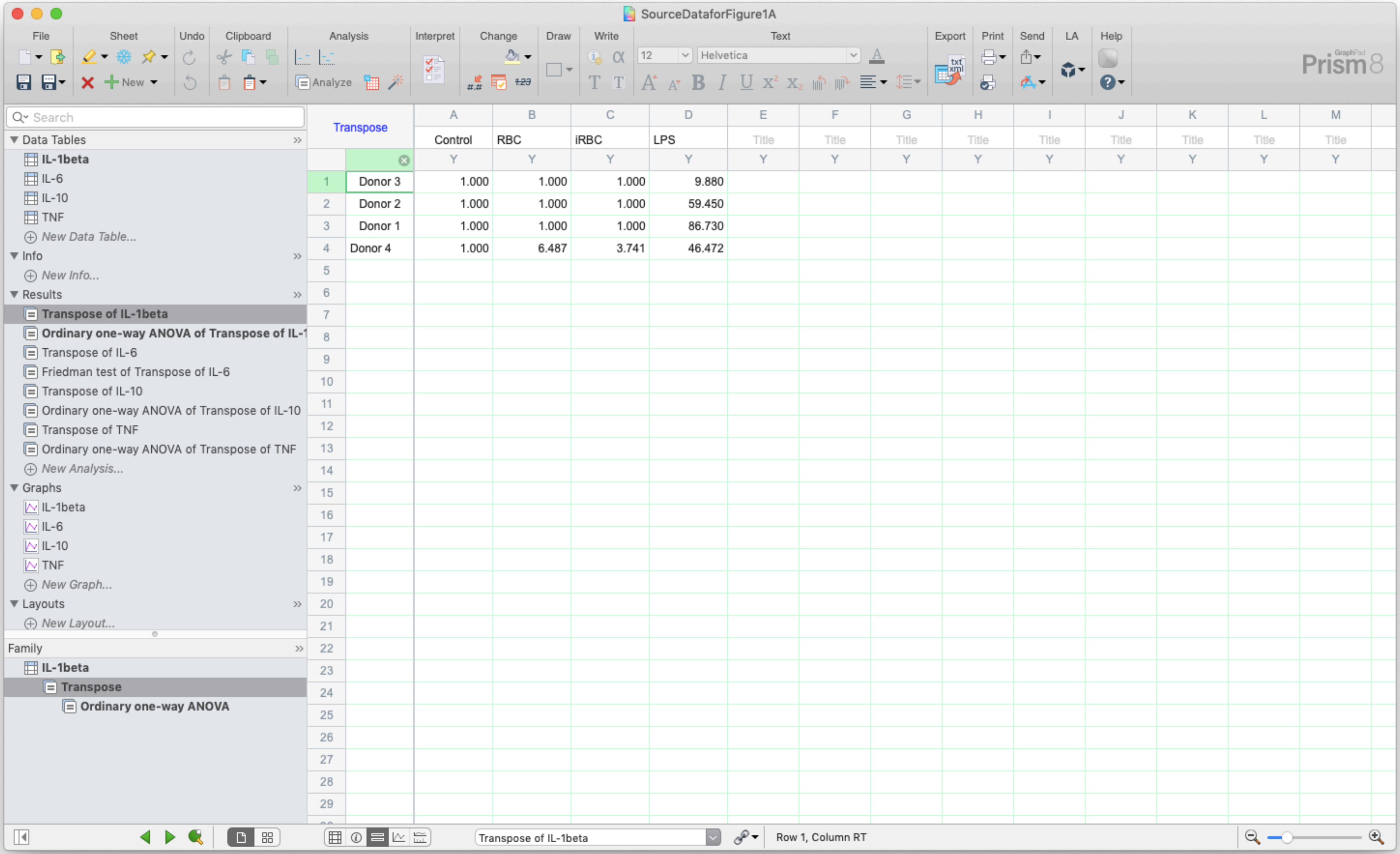

Transpose	Control	RBC	iRBC	LPS
1 Donor 3	1.000	1.000	1.000	9.880
2 Donor 2	1.000	1.000	1.000	59.450
3 Donor 1	1.000	1.000	1.000	86.730
4 Donor 4	1.000	6.487	3.741	46.472

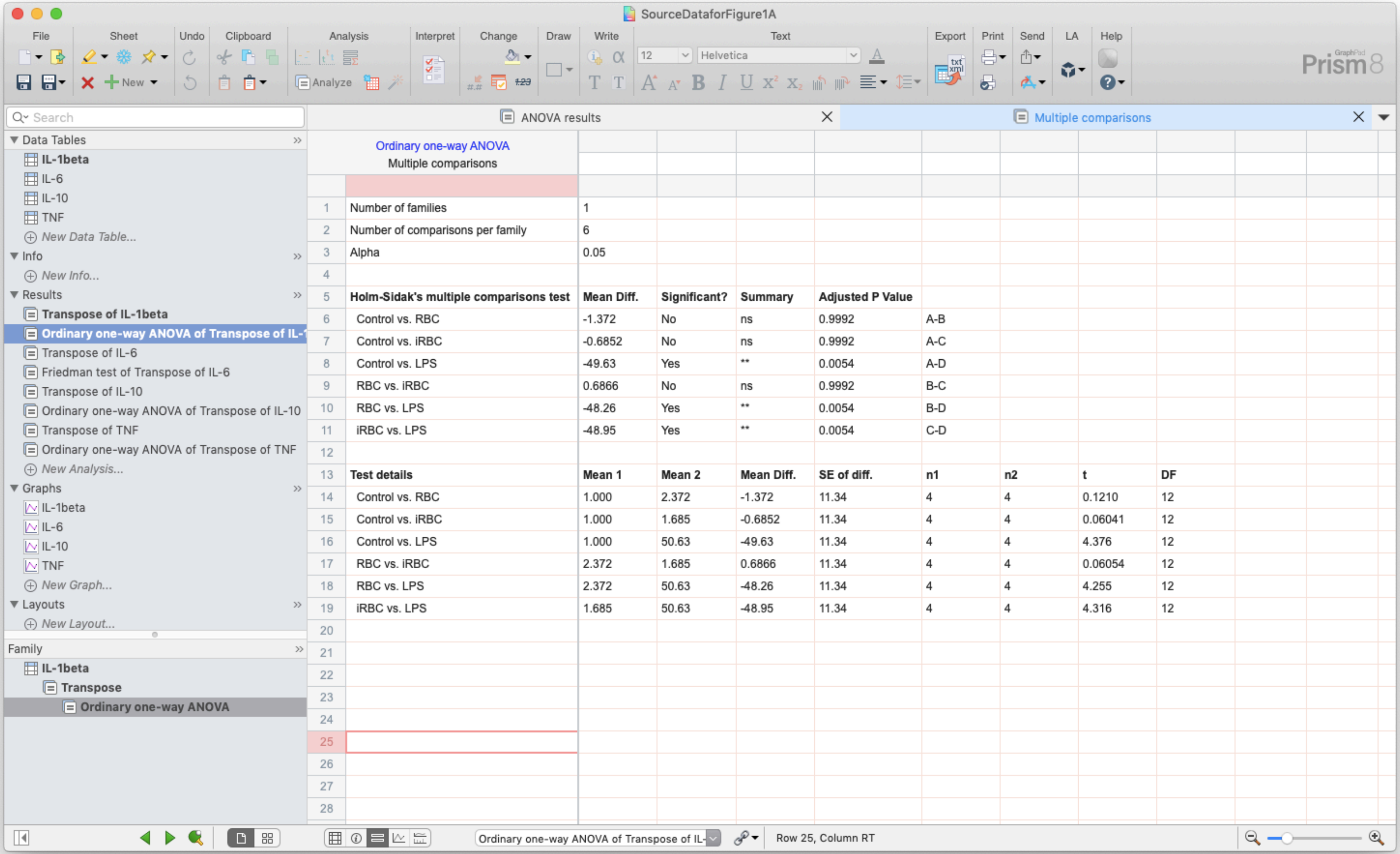

Ordinary one-way ANOVA
Multiple comparisons

1	Number of families	1							
2	Number of comparisons per family	6							
3	Alpha	0.05							
4									
5	**Holm-Sidak's multiple comparisons test**	**Mean Diff.**	**Significant?**	**Summary**	**Adjusted P Value**				
6	Control vs. RBC	-1.372	No	ns	0.9992	A-B			
7	Control vs. iRBC	-0.6852	No	ns	0.9992	A-C			
8	Control vs. LPS	-49.63	Yes	**	0.0054	A-D			
9	RBC vs. iRBC	0.6866	No	ns	0.9992	B-C			
10	RBC vs. LPS	-48.26	Yes	**	0.0054	B-D			
11	iRBC vs. LPS	-48.95	Yes	**	0.0054	C-D			
12									
13	**Test details**	**Mean 1**	**Mean 2**	**Mean Diff.**	**SE of diff.**	**n1**	**n2**	**t**	**DF**
14	Control vs. RBC	1.000	2.372	-1.372	11.34	4	4	0.1210	12
15	Control vs. iRBC	1.000	1.685	-0.6852	11.34	4	4	0.06041	12
16	Control vs. LPS	1.000	50.63	-49.63	11.34	4	4	4.376	12
17	RBC vs. iRBC	2.372	1.685	0.6866	11.34	4	4	0.06054	12
18	RBC vs. LPS	2.372	50.63	-48.26	11.34	4	4	4.255	12
19	iRBC vs. LPS	1.685	50.63	-48.95	11.34	4	4	4.316	12

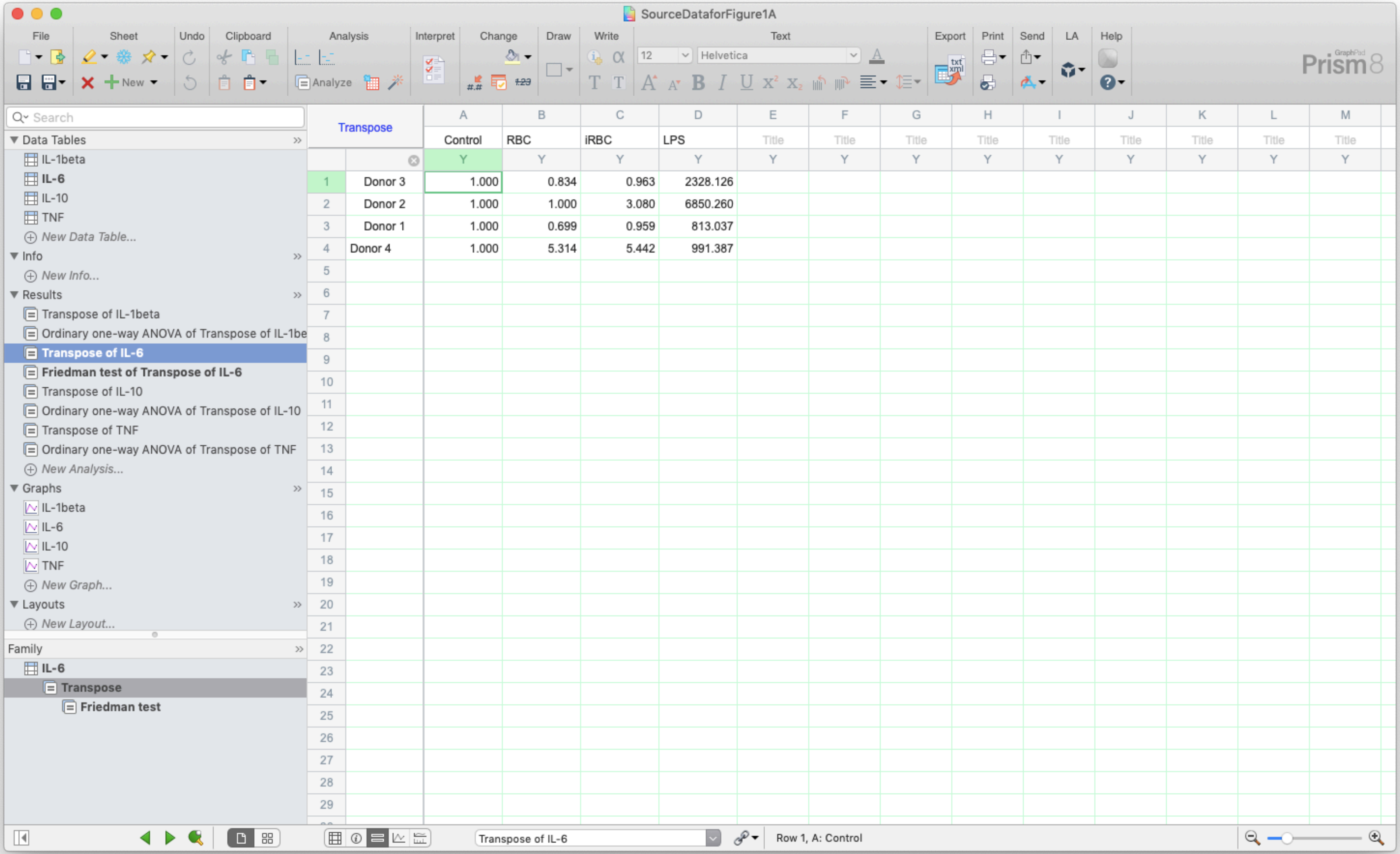

		A	B	C	D
Transpose		Control	RBC	iRBC	LPS
		Y	Y	Y	Y
1	Donor 3	1.000	0.834	0.963	2328.126
2	Donor 2	1.000	1.000	3.080	6850.260
3	Donor 1	1.000	0.699	0.959	813.037
4	Donor 4	1.000	5.314	5.442	991.387

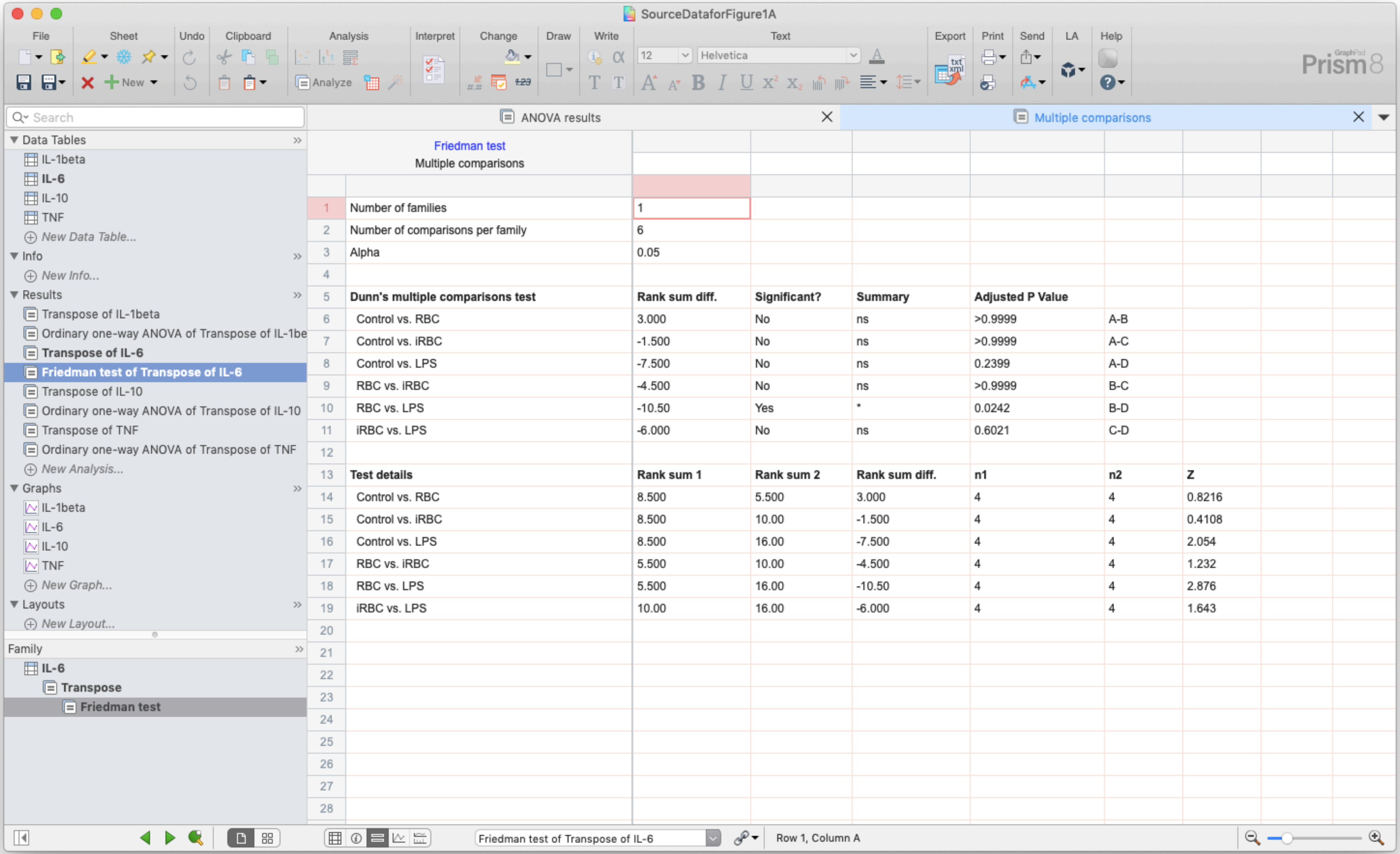

1	Number of families	1					
2	Number of comparisons per family	6					
3	Alpha	0.05					
4							
5	**Dunn's multiple comparisons test**	**Rank sum diff.**	**Significant?**	**Summary**	**Adjusted P Value**		
6	Control vs. RBC	3.000	No	ns	>0.9999	A-B	
7	Control vs. iRBC	-1.500	No	ns	>0.9999	A-C	
8	Control vs. LPS	-7.500	No	ns	0.2399	A-D	
9	RBC vs. iRBC	-4.500	No	ns	>0.9999	B-C	
10	RBC vs. LPS	-10.50	Yes	*	0.0242	B-D	
11	iRBC vs. LPS	-6.000	No	ns	0.6021	C-D	
12							
13	**Test details**	**Rank sum 1**	**Rank sum 2**	**Rank sum diff.**	**n1**	**n2**	**Z**
14	Control vs. RBC	8.500	5.500	3.000	4	4	0.8216
15	Control vs. iRBC	8.500	10.00	-1.500	4	4	0.4108
16	Control vs. LPS	8.500	16.00	-7.500	4	4	2.054
17	RBC vs. iRBC	5.500	10.00	-4.500	4	4	1.232
18	RBC vs. LPS	5.500	16.00	-10.50	4	4	2.876
19	iRBC vs. LPS	10.00	16.00	-6.000	4	4	1.643

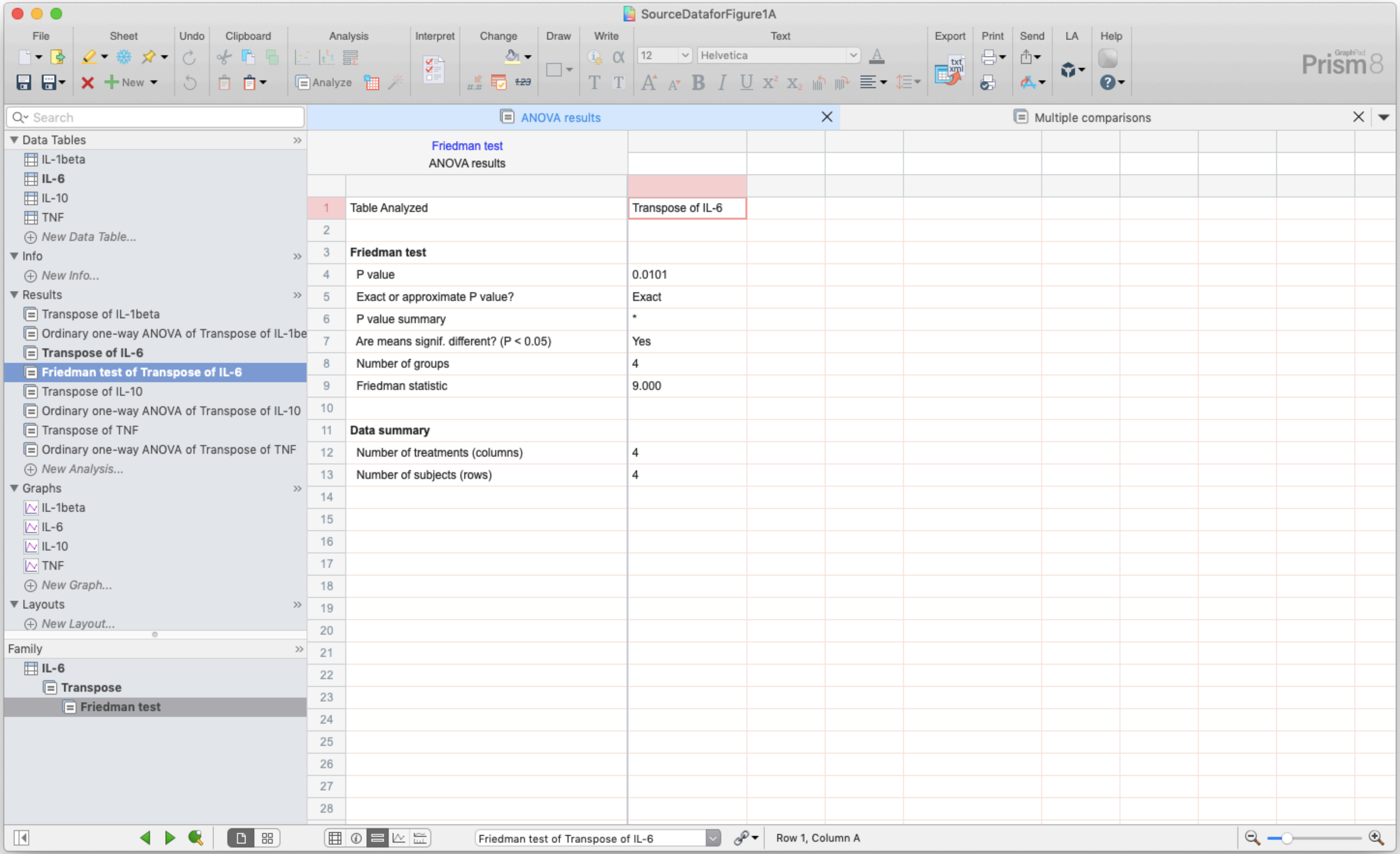

Friedman test ANOVA results		
1	Table Analyzed	Transpose of IL-6
2		
3	**Friedman test**	
4	P value	0.0101
5	Exact or approximate P value?	Exact
6	P value summary	*
7	Are means signif. different? (P < 0.05)	Yes
8	Number of groups	4
9	Friedman statistic	9.000
10		
11	**Data summary**	
12	Number of treatments (columns)	4
13	Number of subjects (rows)	4

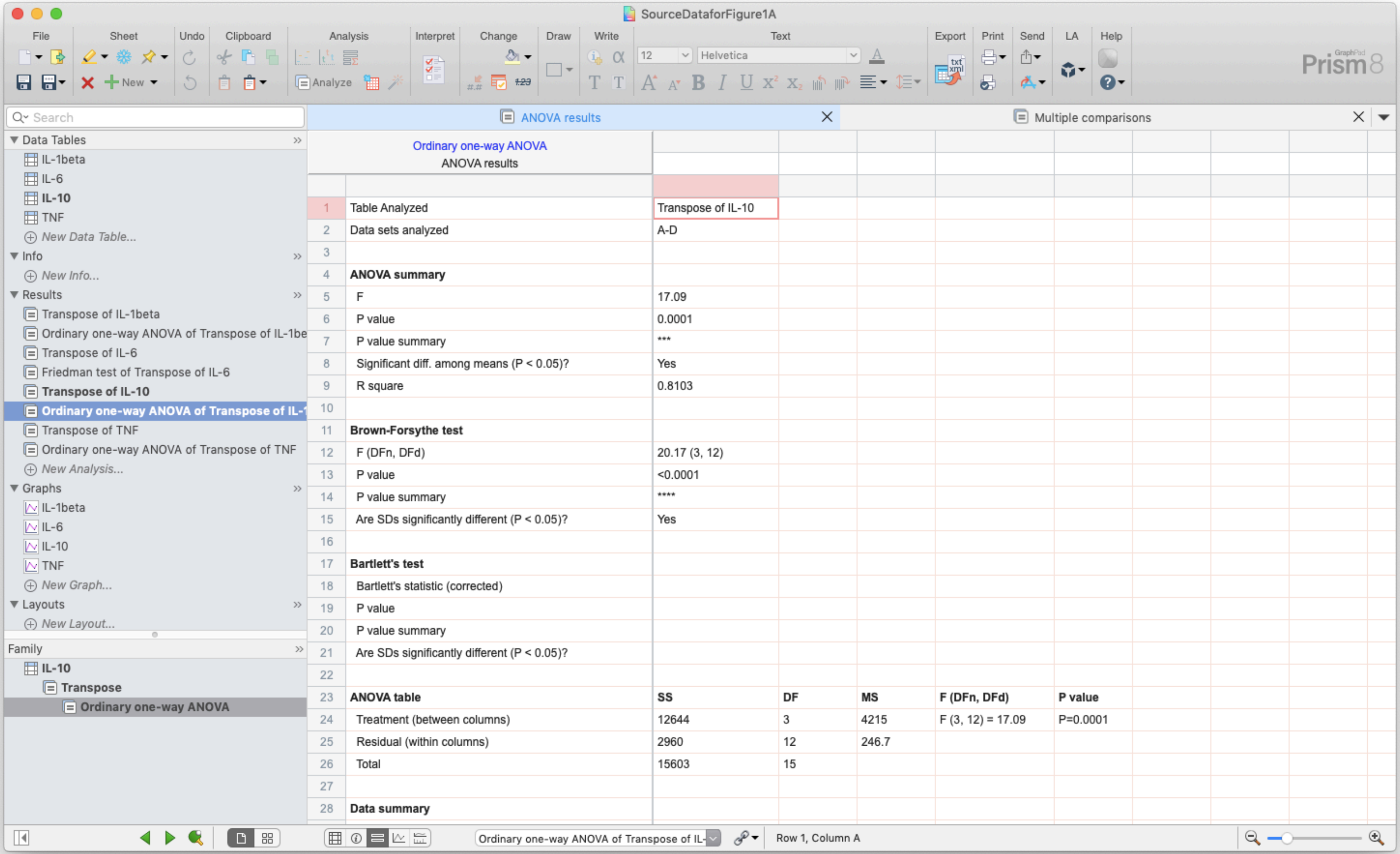

1	Table Analyzed	Transpose of IL-10				
2	Data sets analyzed	A-D				
3						
4	**ANOVA summary**					
5	F	17.09				
6	P value	0.0001				
7	P value summary	***				
8	Significant diff. among means (P < 0.05)?	Yes				
9	R square	0.8103				
10						
11	**Brown-Forsythe test**					
12	F (DFn, DFd)	20.17 (3, 12)				
13	P value	<0.0001				
14	P value summary	****				
15	Are SDs significantly different (P < 0.05)?	Yes				
16						
17	**Bartlett's test**					
18	Bartlett's statistic (corrected)					
19	P value					
20	P value summary					
21	Are SDs significantly different (P < 0.05)?					
22						
23	**ANOVA table**	**SS**	**DF**	**MS**	**F (DFn, DFd)**	**P value**
24	Treatment (between columns)	12644	3	4215	F (3, 12) = 17.09	P=0.0001
25	Residual (within columns)	2960	12	246.7		
26	Total	15603	15			
27						
28	**Data summary**					

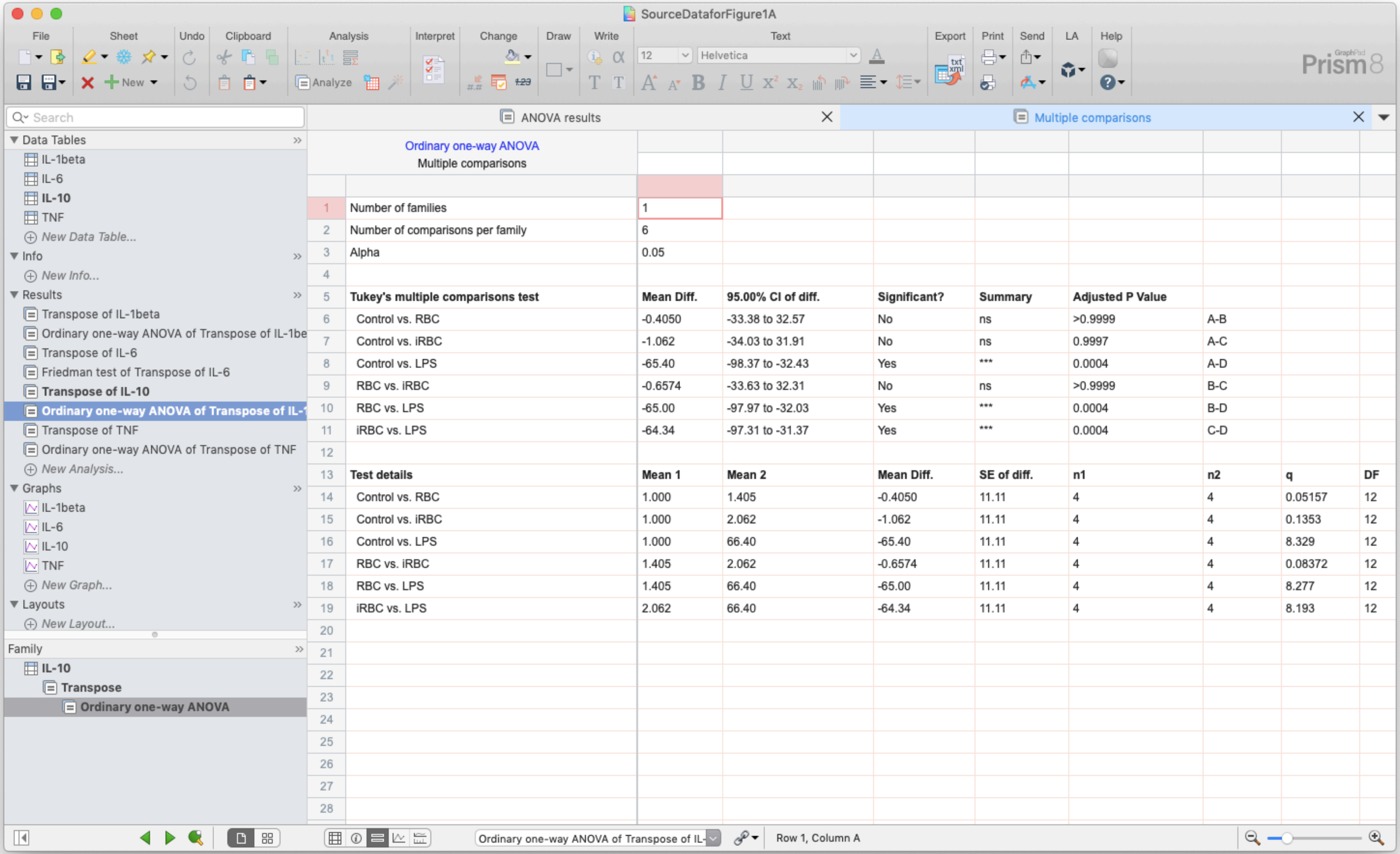

Ordinary one-way ANOVA
Multiple comparisons

1	Number of families	1							
2	Number of comparisons per family	6							
3	Alpha	0.05							
4									
5	**Tukey's multiple comparisons test**	**Mean Diff.**	**95.00% CI of diff.**	**Significant?**	**Summary**	**Adjusted P Value**			
6	Control vs. RBC	-0.4050	-33.38 to 32.57	No	ns	>0.9999	A-B		
7	Control vs. iRBC	-1.062	-34.03 to 31.91	No	ns	0.9997	A-C		
8	Control vs. LPS	-65.40	-98.37 to -32.43	Yes	***	0.0004	A-D		
9	RBC vs. iRBC	-0.6574	-33.63 to 32.31	No	ns	>0.9999	B-C		
10	RBC vs. LPS	-65.00	-97.97 to -32.03	Yes	***	0.0004	B-D		
11	iRBC vs. LPS	-64.34	-97.31 to -31.37	Yes	***	0.0004	C-D		
12									
13	**Test details**	**Mean 1**	**Mean 2**	**Mean Diff.**	**SE of diff.**	**n1**	**n2**	**q**	**DF**
14	Control vs. RBC	1.000	1.405	-0.4050	11.11	4	4	0.05157	12
15	Control vs. iRBC	1.000	2.062	-1.062	11.11	4	4	0.1353	12
16	Control vs. LPS	1.000	66.40	-65.40	11.11	4	4	8.329	12
17	RBC vs. iRBC	1.405	2.062	-0.6574	11.11	4	4	0.08372	12
18	RBC vs. LPS	1.405	66.40	-65.00	11.11	4	4	8.277	12
19	iRBC vs. LPS	2.062	66.40	-64.34	11.11	4	4	8.193	12

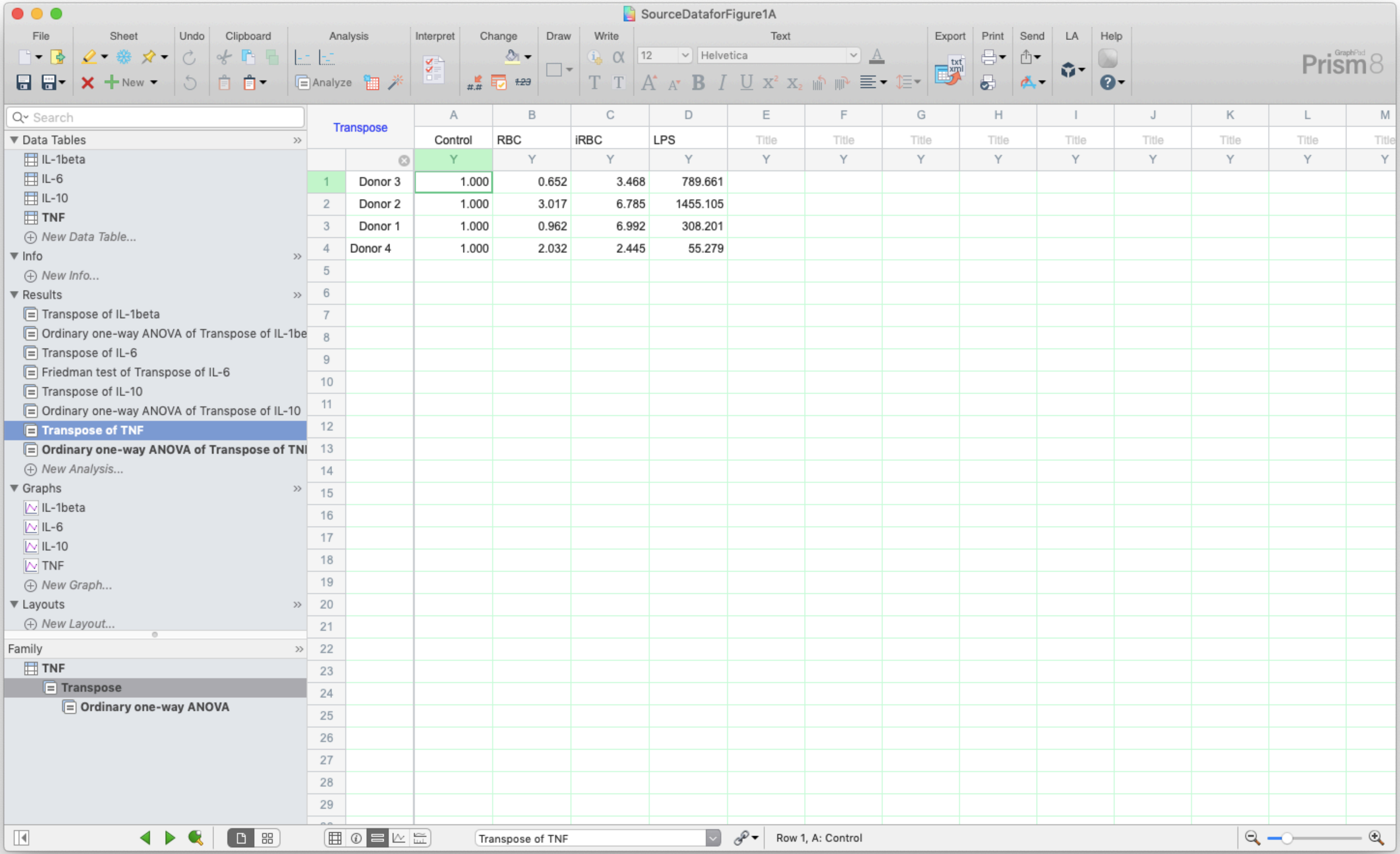

SourceDataforFigure1A

Transpose

	Control	RBC	iRBC	LPS
Donor 3	1.000	0.652	3.468	789.661
Donor 2	1.000	3.017	6.785	1455.105
Donor 1	1.000	0.962	6.992	308.201
Donor 4	1.000	2.032	2.445	55.279

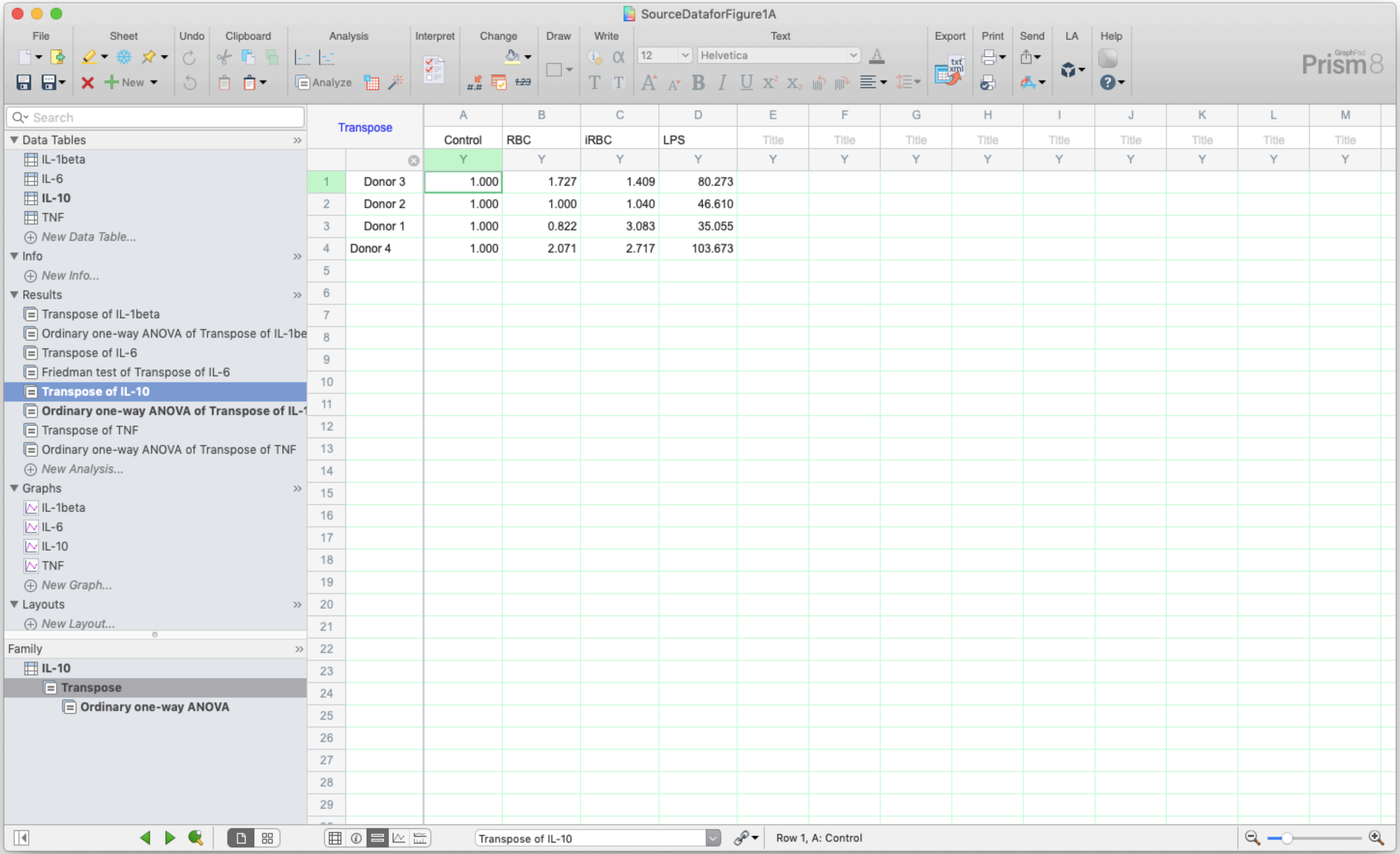

Transpose	Control	RBC	iRBC	LPS
	Y	Y	Y	Y
Donor 3	1.000	1.727	1.409	80.273
Donor 2	1.000	1.000	1.040	46.610
Donor 1	1.000	0.822	3.083	35.055
Donor 4	1.000	2.071	2.717	103.673

SourceDataforFigure1A

Ordinary one-way ANOVA
Multiple comparisons

1	Number of families	1							
2	Number of comparisons per family	6							
3	Alpha	0.05							
4									
5	**Tukey's multiple comparisons test**	**Mean Diff.**	**95.00% CI of diff.**	**Significant?**	**Summary**	**Adjusted P Value**			
6	Control vs. RBC	-0.6657	-647.2 to 645.9	No	ns	>0.9999	A-B		
7	Control vs. iRBC	-3.923	-650.5 to 642.6	No	ns	>0.9999	A-C		
8	Control vs. LPS	-651.1	-1298 to -4.509	Yes	*	0.0482	A-D		
9	RBC vs. iRBC	-3.257	-649.8 to 643.3	No	ns	>0.9999	B-C		
10	RBC vs. LPS	-650.4	-1297 to -3.844	Yes	*	0.0485	B-D		
11	iRBC vs. LPS	-647.1	-1294 to -0.5867	Yes	*	0.0498	C-D		
12									
13	**Test details**	**Mean 1**	**Mean 2**	**Mean Diff.**	**SE of diff.**	**n1**	**n2**	**q**	**DF**
14	Control vs. RBC	1.000	1.666	-0.6657	217.8	4	4	0.004323	12
15	Control vs. iRBC	1.000	4.923	-3.923	217.8	4	4	0.02547	12
16	Control vs. LPS	1.000	652.1	-651.1	217.8	4	4	4.228	12
17	RBC vs. iRBC	1.666	4.923	-3.257	217.8	4	4	0.02115	12
18	RBC vs. LPS	1.666	652.1	-650.4	217.8	4	4	4.224	12
19	iRBC vs. LPS	4.923	652.1	-647.1	217.8	4	4	4.202	12

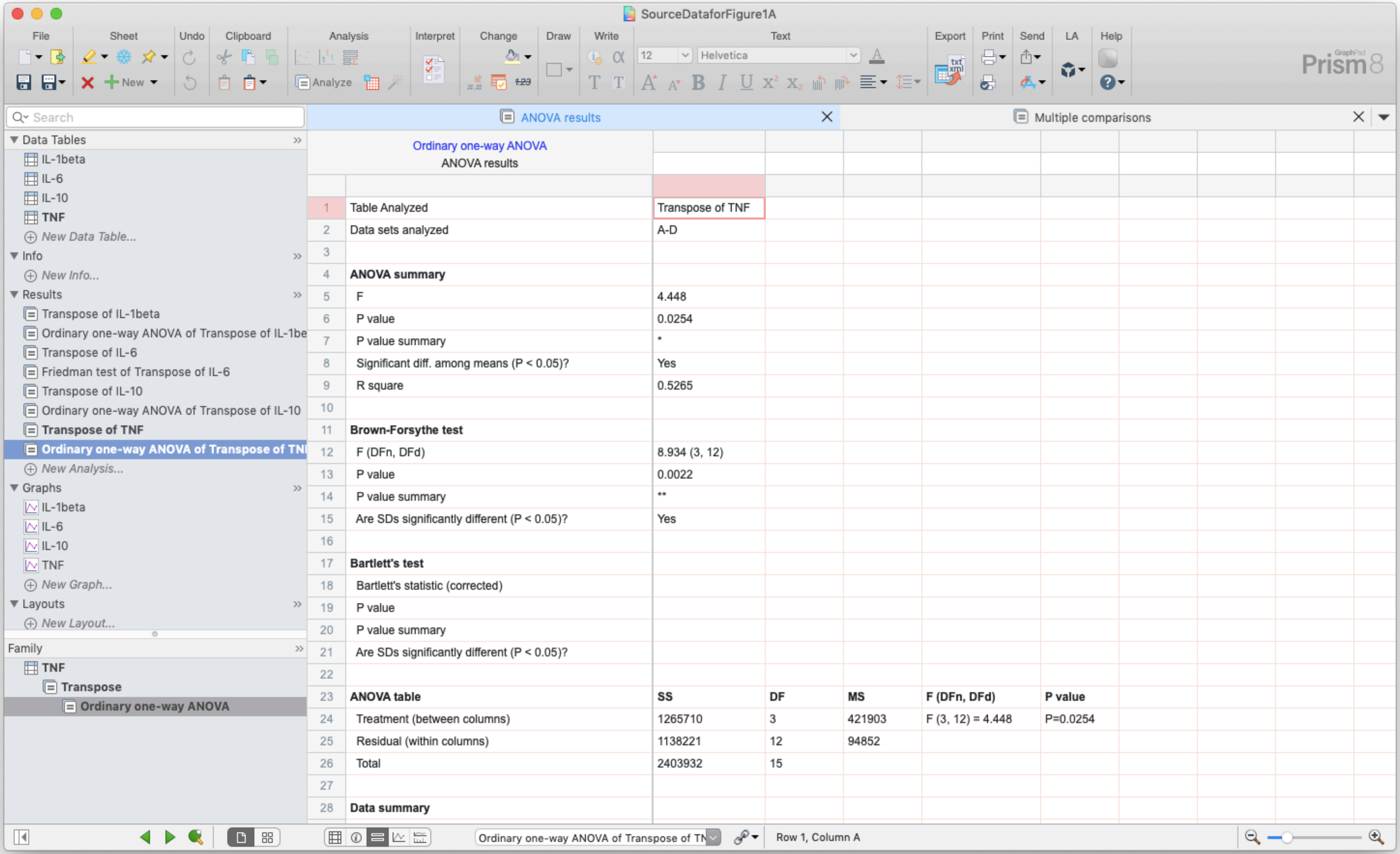

Ordinary one-way ANOVA
ANOVA results

1	Table Analyzed	Transpose of TNF				
2	Data sets analyzed	A-D				
3						
4	**ANOVA summary**					
5	F	4.448				
6	P value	0.0254				
7	P value summary	*				
8	Significant diff. among means (P < 0.05)?	Yes				
9	R square	0.5265				
10						
11	**Brown-Forsythe test**					
12	F (DFn, DFd)	8.934 (3, 12)				
13	P value	0.0022				
14	P value summary	**				
15	Are SDs significantly different (P < 0.05)?	Yes				
16						
17	**Bartlett's test**					
18	Bartlett's statistic (corrected)					
19	P value					
20	P value summary					
21	Are SDs significantly different (P < 0.05)?					
22						
23	**ANOVA table**	**SS**	**DF**	**MS**	**F (DFn, DFd)**	**P value**
24	Treatment (between columns)	1265710	3	421903	F (3, 12) = 4.448	P=0.0254
25	Residual (within columns)	1138221	12	94852		
26	Total	2403932	15			
27						
28	**Data summary**					

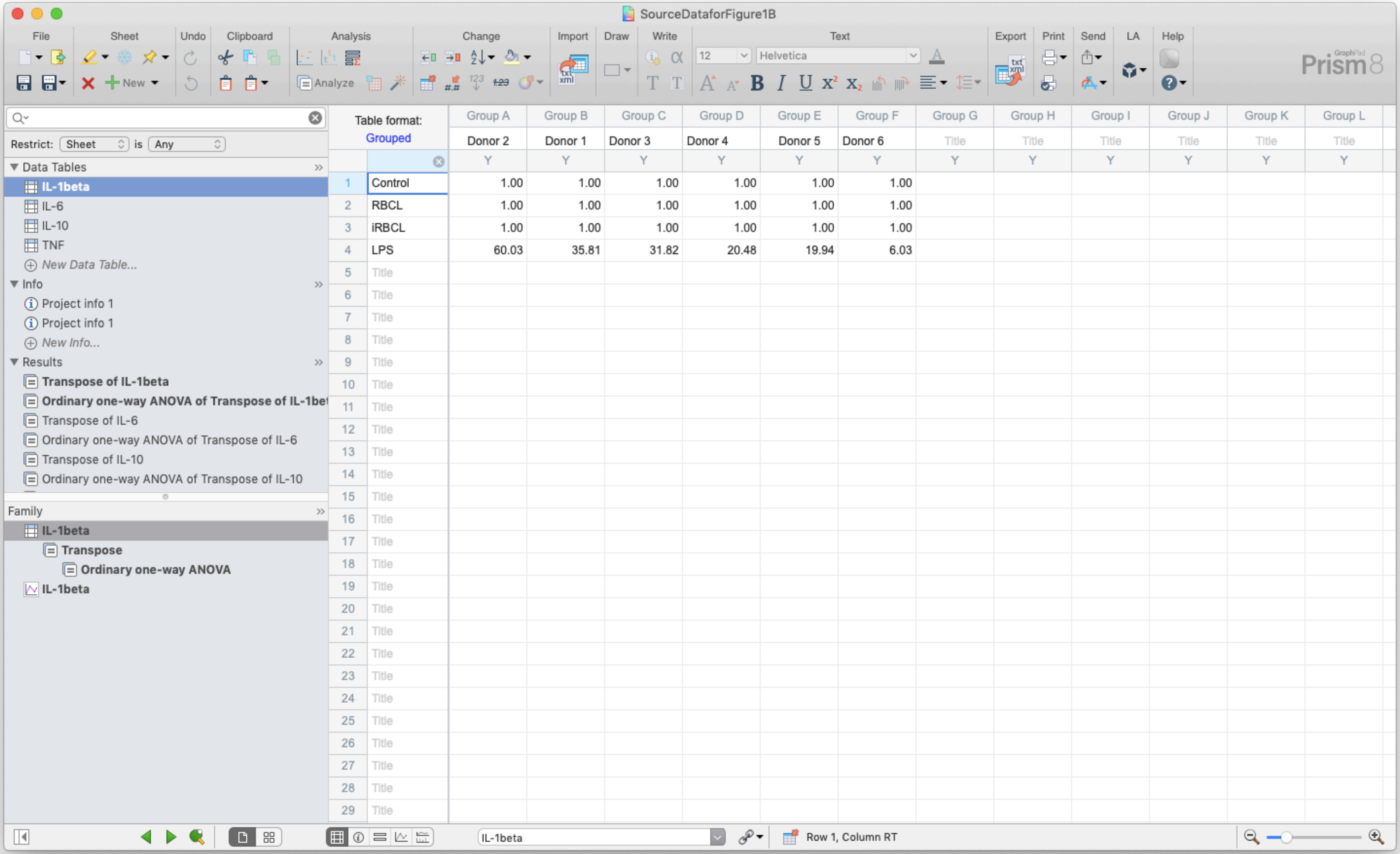

	Group A	Group B	Group C	Group D	Group E	Group F
	Donor 2	Donor 1	Donor 3	Donor 4	Donor 5	Donor 6
	Y	Y	Y	Y	Y	Y
1 Control	1.00	1.00	1.00	1.00	1.00	1.00
2 RBCL	1.00	1.00	1.00	1.00	1.00	1.00
3 iRBCL	1.00	1.00	1.00	1.00	1.00	1.00
4 LPS	60.03	35.81	31.82	20.48	19.94	6.03

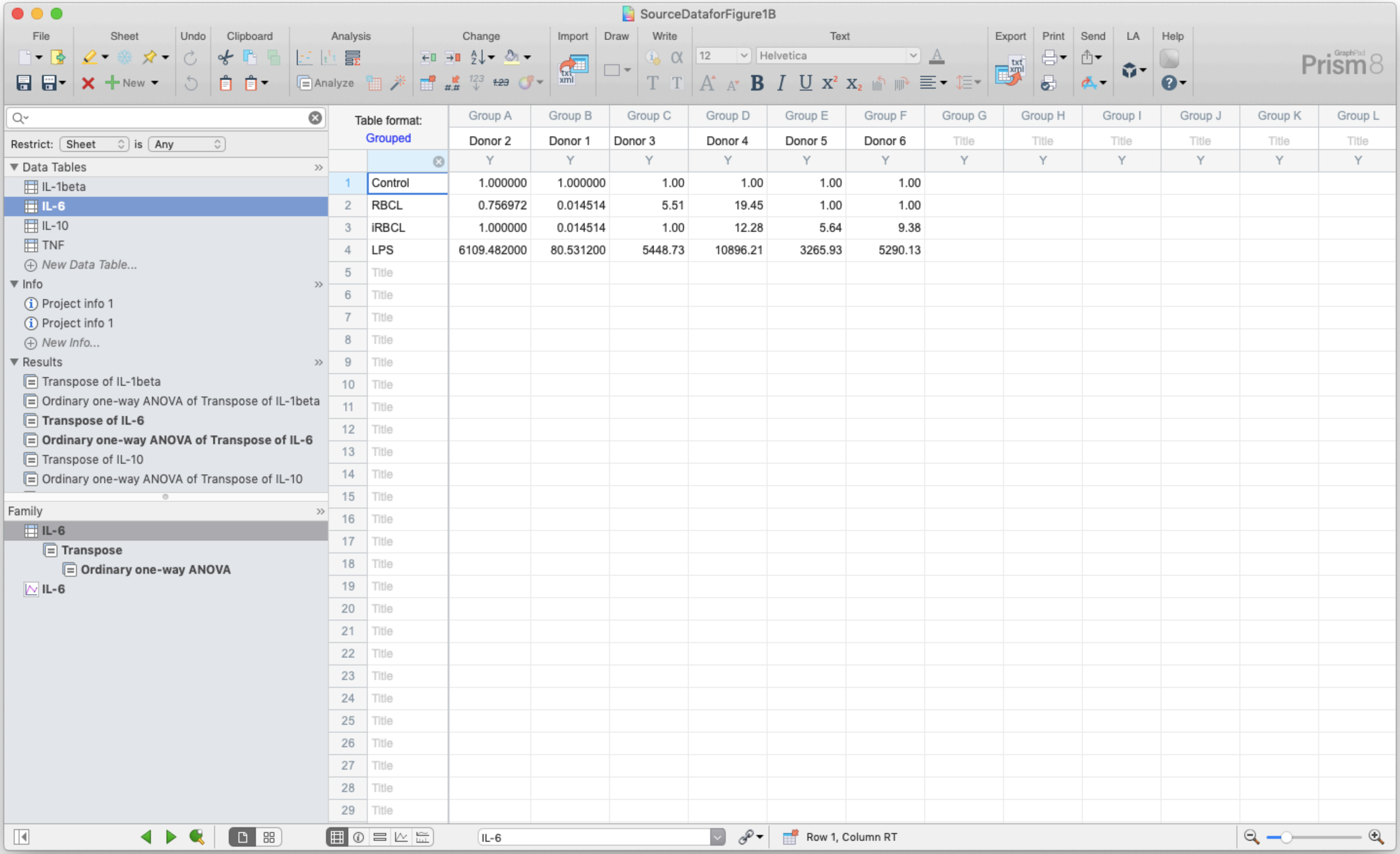

	Group A	Group B	Group C	Group D	Group E	Group F
	Donor 2	Donor 1	Donor 3	Donor 4	Donor 5	Donor 6
Control	1.000000	1.000000	1.00	1.00	1.00	1.00
RBCL	0.756972	0.014514	5.51	19.45	1.00	1.00
iRBCL	1.000000	0.014514	1.00	12.28	5.64	9.38
LPS	6109.482000	80.531200	5448.73	10896.21	3265.93	5290.13

SourceDataforFigure1B

Data Tables
- IL-1beta
- IL-6
- IL-10
- TNF
- New Data Table...

Info
- Project info 1
- Project info 1
- New Info...

Results
- Transpose of IL-1beta
- Ordinary one-way ANOVA of Transpose of IL-1beta
- Transpose of IL-6
- Ordinary one-way ANOVA of Transpose of IL-6
- **Transpose of IL-10**
- **Ordinary one-way ANOVA of Transpose of IL-10**

Family
- **IL-10**
  - **Transpose**
    - **Ordinary one-way ANOVA**
- **IL-10**

Table format: Grouped

		Group A	Group B	Group C	Group D	Group E	Group F
		Donor 2	Donor 1	Donor 3	Donor 4	Donor 5	Donor 6
		Y	Y	Y	Y	Y	Y
1	Control	1.000000	1.000000	1.00	1.00	1.00	1.00
2	RBCL	0.864516	0.271739	1.00	2.51	1.00	1.00
3	iRBCL	1.135484	0.739130	1.00	2.41	1.00	2.08
4	LPS	19.187100	34.331520	62.09	35.26	4.92	13.71

IL-10

Row 1, Column RT

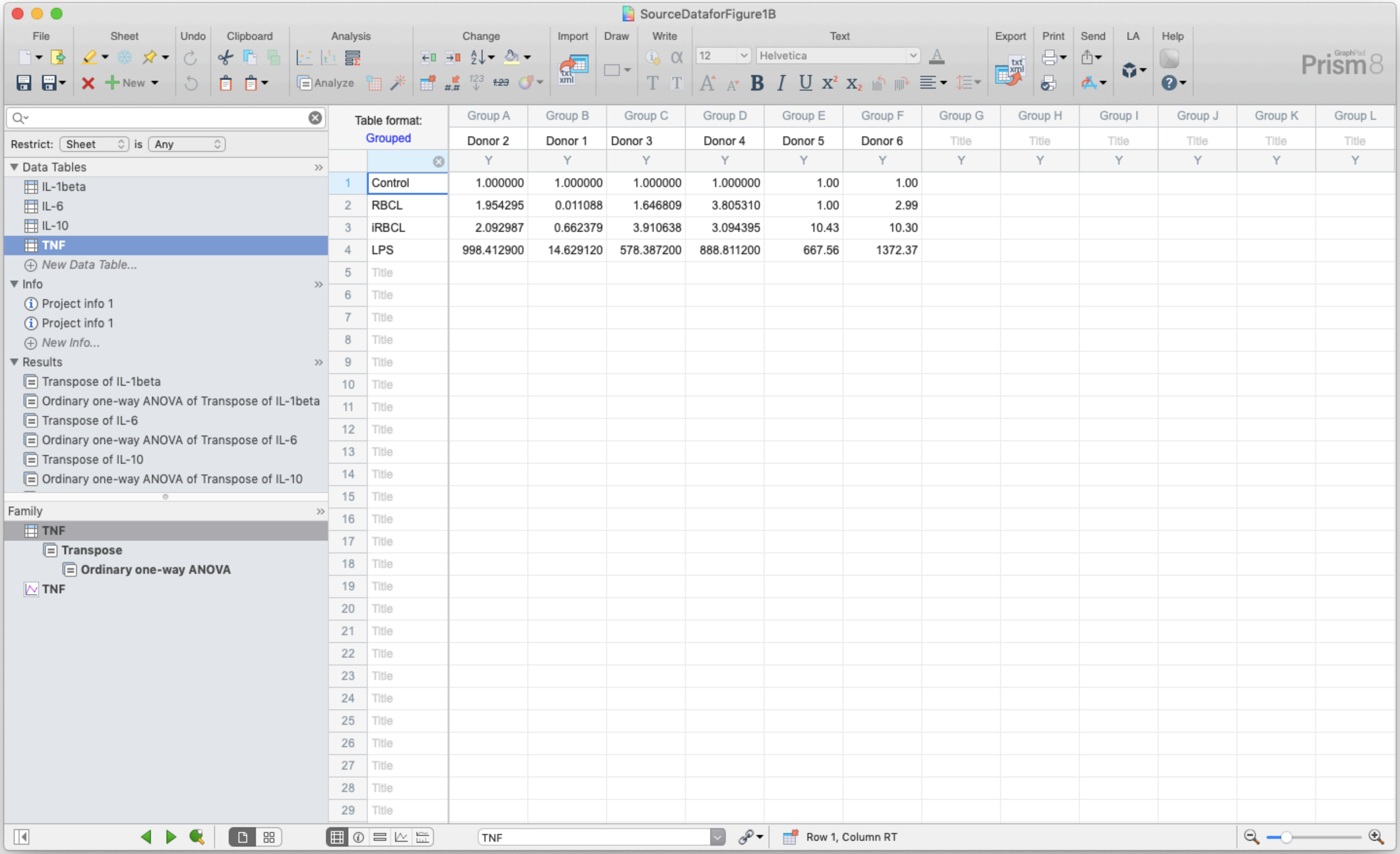

SourceDataforFigure1B

Table format: Grouped

	Group A	Group B	Group C	Group D	Group E	Group F
	Donor 2	Donor 1	Donor 3	Donor 4	Donor 5	Donor 6
	Y	Y	Y	Y	Y	Y
Control	1.000000	1.000000	1.000000	1.000000	1.00	1.00
RBCL	1.954295	0.011088	1.646809	3.805310	1.00	2.99
iRBCL	2.092987	0.662379	3.910638	3.094395	10.43	10.30
LPS	998.412900	14.629120	578.387200	888.811200	667.56	1372.37

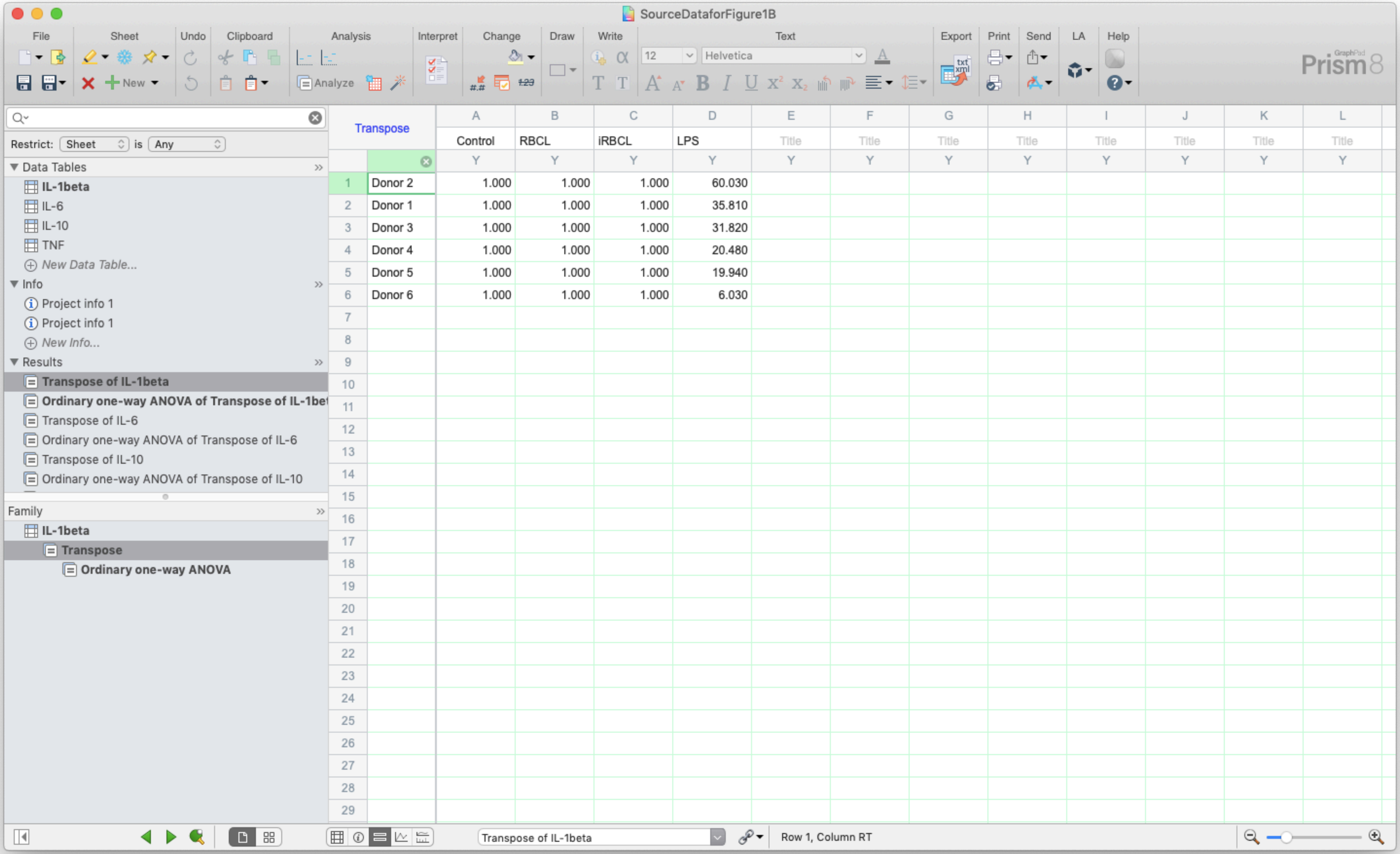

		Control	RBCL	iRBCL	LPS
1	Donor 2	1.000	1.000	1.000	60.030
2	Donor 1	1.000	1.000	1.000	35.810
3	Donor 3	1.000	1.000	1.000	31.820
4	Donor 4	1.000	1.000	1.000	20.480
5	Donor 5	1.000	1.000	1.000	19.940
6	Donor 6	1.000	1.000	1.000	6.030

SourceDataforFigure1B

Data Tables
- IL-1beta
- IL-6
- IL-10
- TNF

Info
- Project info 1
- Project info 1

Results
- Transpose of IL-1beta
- Ordinary one-way ANOVA of Transpose of IL-1bet
- Transpose of IL-6
- Ordinary one-way ANOVA of Transpose of IL-6
- Transpose of IL-10
- Ordinary one-way ANOVA of Transpose of IL-10

Family
- IL-1beta
  - Transpose
    - Ordinary one-way ANOVA

ANOVA results | Multiple comparisons

Ordinary one-way ANOVA
ANOVA results

1	Table Analyzed	Transpose of IL-1beta				
2	Data sets analyzed	A-D				
3						
4	**ANOVA summary**					
5	F	13.86				
6	P value	<0.0001				
7	P value summary	****				
8	Significant diff. among means (P < 0.05)?	Yes				
9	R square	0.6752				
10						
11	**Brown-Forsythe test**					
12	F (DFn, DFd)	8.460 (3, 20)				
13	P value	0.0008				
14	P value summary	***				
15	Are SDs significantly different (P < 0.05)?	Yes				
16						
17	**Bartlett's test**					
18	Bartlett's statistic (corrected)					
19	P value					
20	P value summary					
21	Are SDs significantly different (P < 0.05)?					
22						
23	**ANOVA table**	**SS**	**DF**	**MS**	**F (DFn, DFd)**	**P value**
24	Treatment (between columns)	3533	3	1178	F (3, 20) = 13.86	P<0.0001
25	Residual (within columns)	1699	20	84.97		
26	Total	5232	23			
27						
28	**Data summary**					

Ordinary one-way ANOVA of Transpose of IL- | Row 1, Column A

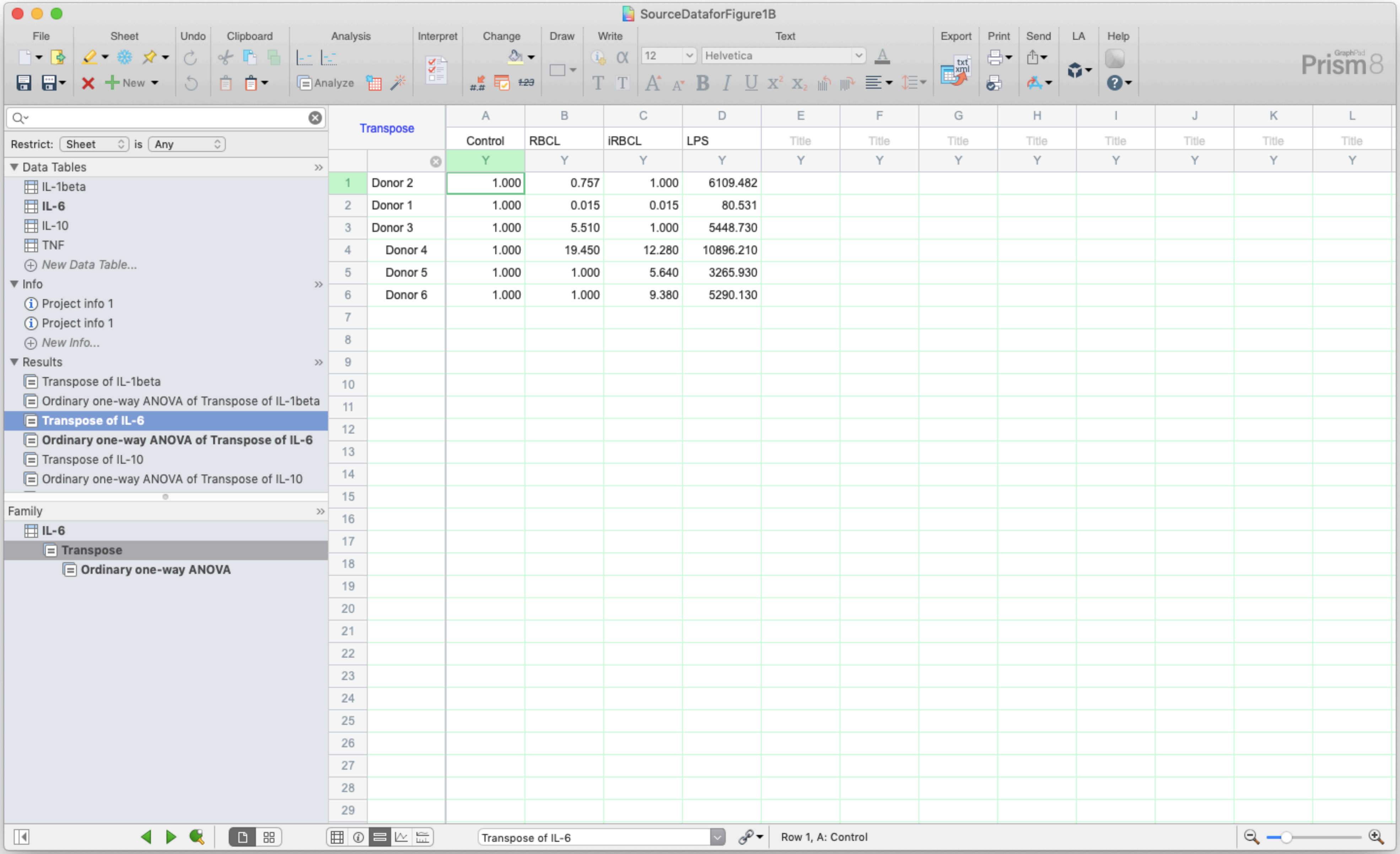

Transpose	A: Control (Y)	B: RBCL (Y)	C: iRBCL (Y)	D: LPS (Y)
1 Donor 2	1.000	0.757	1.000	6109.482
2 Donor 1	1.000	0.015	0.015	80.531
3 Donor 3	1.000	5.510	1.000	5448.730
4 Donor 4	1.000	19.450	12.280	10896.210
5 Donor 5	1.000	1.000	5.640	3265.930
6 Donor 6	1.000	1.000	9.380	5290.130

SourceDataforFigure1B

Ordinary one-way ANOVA
ANOVA results

1	Table Analyzed	Transpose of IL-6				
2	Data sets analyzed	A-D				
3						
4	**ANOVA summary**					
5	F	12.71				
6	P value	<0.0001				
7	P value summary	****				
8	Significant diff. among means (P < 0.05)?	Yes				
9	R square	0.6559				
10						
11	**Brown-Forsythe test**					
12	F (DFn, DFd)	5.010 (3, 20)				
13	P value	0.0094				
14	P value summary	**				
15	Are SDs significantly different (P < 0.05)?	Yes				
16						
17	**Bartlett's test**					
18	Bartlett's statistic (corrected)					
19	P value					
20	P value summary					
21	Are SDs significantly different (P < 0.05)?					
22						
23	**ANOVA table**	**SS**	**DF**	**MS**	**F (DFn, DFd)**	**P value**
24	Treatment (between columns)	120668153	3	40222718	F (3, 20) = 12.71	P<0.0001
25	Residual (within columns)	63291976	20	3164599		
26	Total	183960129	23			
27						
28	**Data summary**					

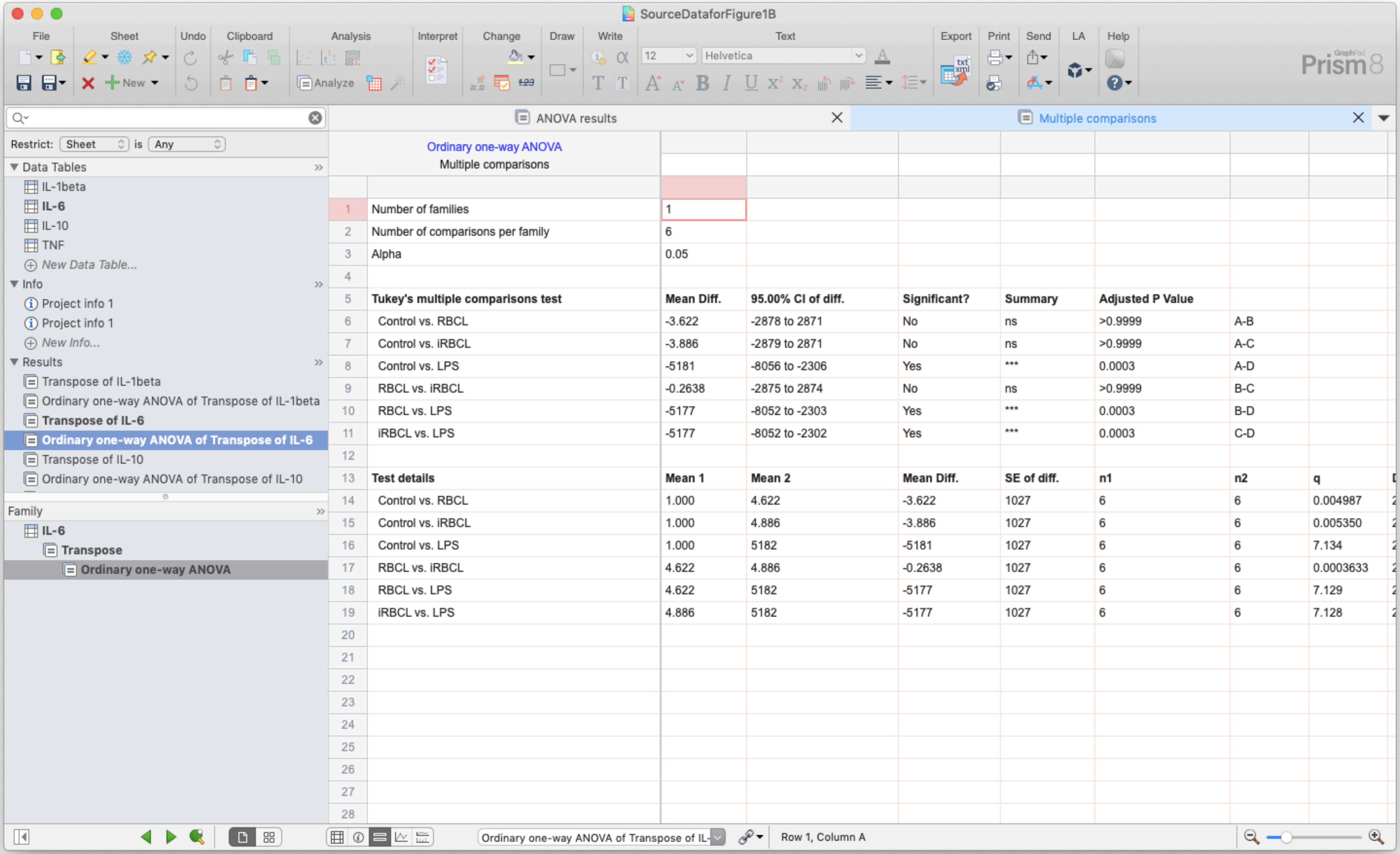

Ordinary one-way ANOVA
Multiple comparisons

1	Number of families	1							
2	Number of comparisons per family	6							
3	Alpha	0.05							
4									
5	**Tukey's multiple comparisons test**	**Mean Diff.**	**95.00% CI of diff.**	**Significant?**	**Summary**	**Adjusted P Value**			
6	Control vs. RBCL	-3.622	-2878 to 2871	No	ns	>0.9999	A-B		
7	Control vs. iRBCL	-3.886	-2879 to 2871	No	ns	>0.9999	A-C		
8	Control vs. LPS	-5181	-8056 to -2306	Yes	***	0.0003	A-D		
9	RBCL vs. iRBCL	-0.2638	-2875 to 2874	No	ns	>0.9999	B-C		
10	RBCL vs. LPS	-5177	-8052 to -2303	Yes	***	0.0003	B-D		
11	iRBCL vs. LPS	-5177	-8052 to -2302	Yes	***	0.0003	C-D		
12									
13	**Test details**	**Mean 1**	**Mean 2**	**Mean Diff.**	**SE of diff.**	**n1**	**n2**	**q**	[illegible]
14	Control vs. RBCL	1.000	4.622	-3.622	1027	6	6	0.004987	[illegible]
15	Control vs. iRBCL	1.000	4.886	-3.886	1027	6	6	0.005350	[illegible]
16	Control vs. LPS	1.000	5182	-5181	1027	6	6	7.134	[illegible]
17	RBCL vs. iRBCL	4.622	4.886	-0.2638	1027	6	6	0.0003633	[illegible]
18	RBCL vs. LPS	4.622	5182	-5177	1027	6	6	7.129	[illegible]
19	iRBCL vs. LPS	4.886	5182	-5177	1027	6	6	7.128	[illegible]

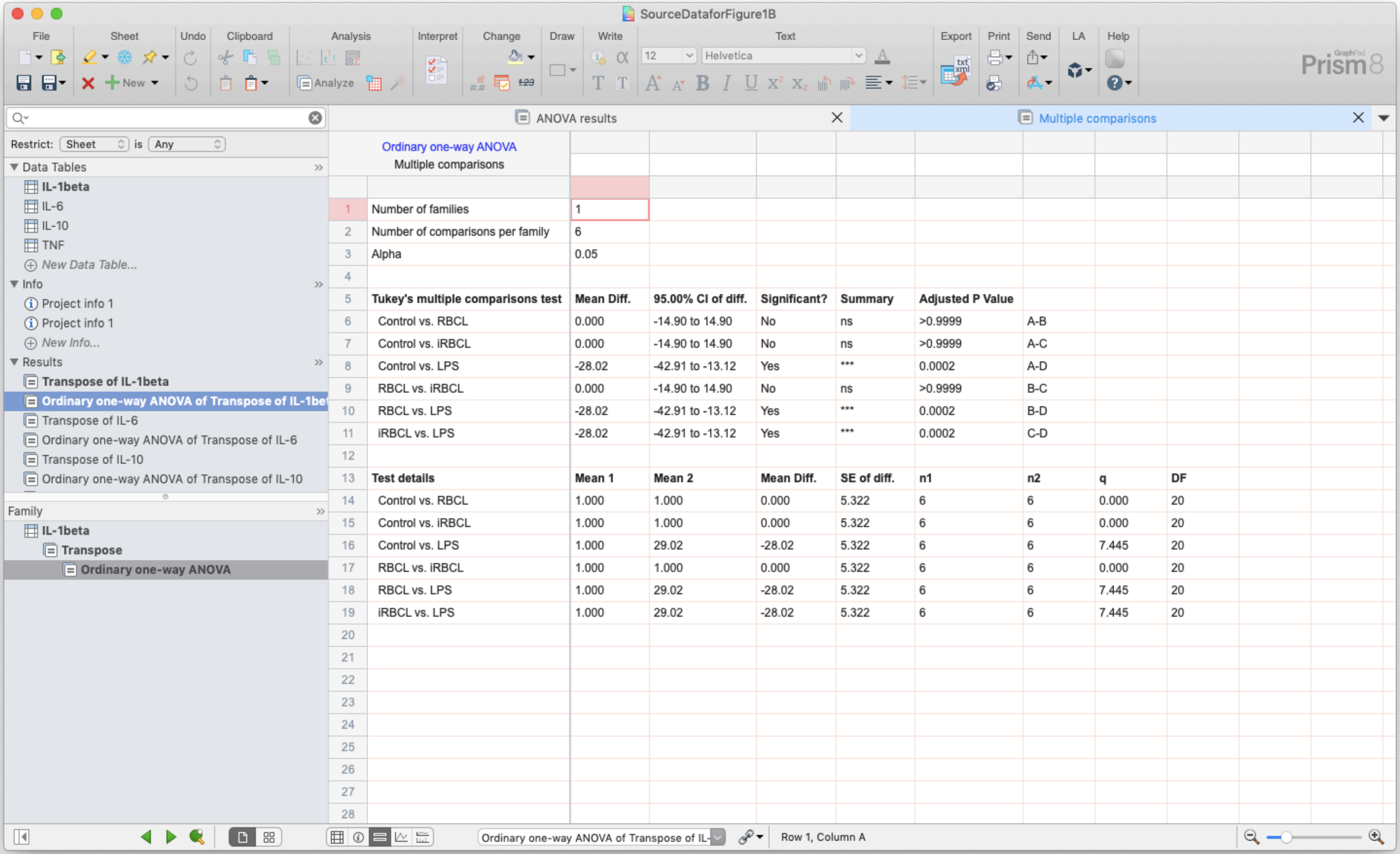

	Ordinary one-way ANOVA<br>Multiple comparisons									
1	Number of families	1								
2	Number of comparisons per family	6								
3	Alpha	0.05								
4										
5	**Tukey's multiple comparisons test**	**Mean Diff.**	**95.00% CI of diff.**	**Significant?**	**Summary**	**Adjusted P Value**				
6	Control vs. RBCL	0.000	-14.90 to 14.90	No	ns	>0.9999	A-B			
7	Control vs. iRBCL	0.000	-14.90 to 14.90	No	ns	>0.9999	A-C			
8	Control vs. LPS	-28.02	-42.91 to -13.12	Yes	***	0.0002	A-D			
9	RBCL vs. iRBCL	0.000	-14.90 to 14.90	No	ns	>0.9999	B-C			
10	RBCL vs. LPS	-28.02	-42.91 to -13.12	Yes	***	0.0002	B-D			
11	iRBCL vs. LPS	-28.02	-42.91 to -13.12	Yes	***	0.0002	C-D			
12										
13	**Test details**	**Mean 1**	**Mean 2**	**Mean Diff.**	**SE of diff.**	**n1**	**n2**	**q**	**DF**	
14	Control vs. RBCL	1.000	1.000	0.000	5.322	6	6	0.000	20	
15	Control vs. iRBCL	1.000	1.000	0.000	5.322	6	6	0.000	20	
16	Control vs. LPS	1.000	29.02	-28.02	5.322	6	6	7.445	20	
17	RBCL vs. iRBCL	1.000	1.000	0.000	5.322	6	6	0.000	20	
18	RBCL vs. LPS	1.000	29.02	-28.02	5.322	6	6	7.445	20	
19	iRBCL vs. LPS	1.000	29.02	-28.02	5.322	6	6	7.445	20	

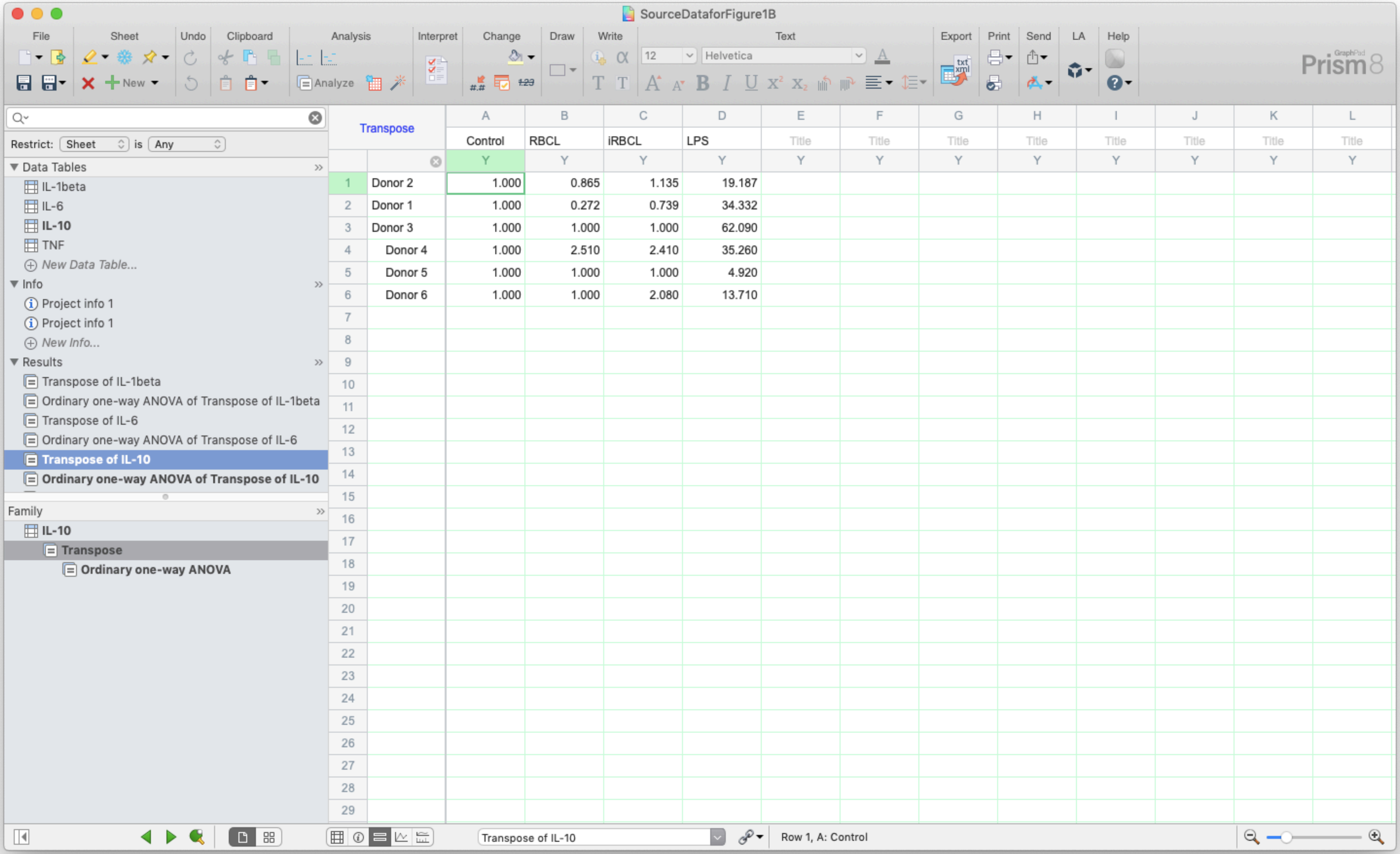

Transpose	Control	RBCL	iRBCL	LPS
Donor 2	1.000	0.865	1.135	19.187
Donor 1	1.000	0.272	0.739	34.332
Donor 3	1.000	1.000	1.000	62.090
Donor 4	1.000	2.510	2.410	35.260
Donor 5	1.000	1.000	1.000	4.920
Donor 6	1.000	1.000	2.080	13.710

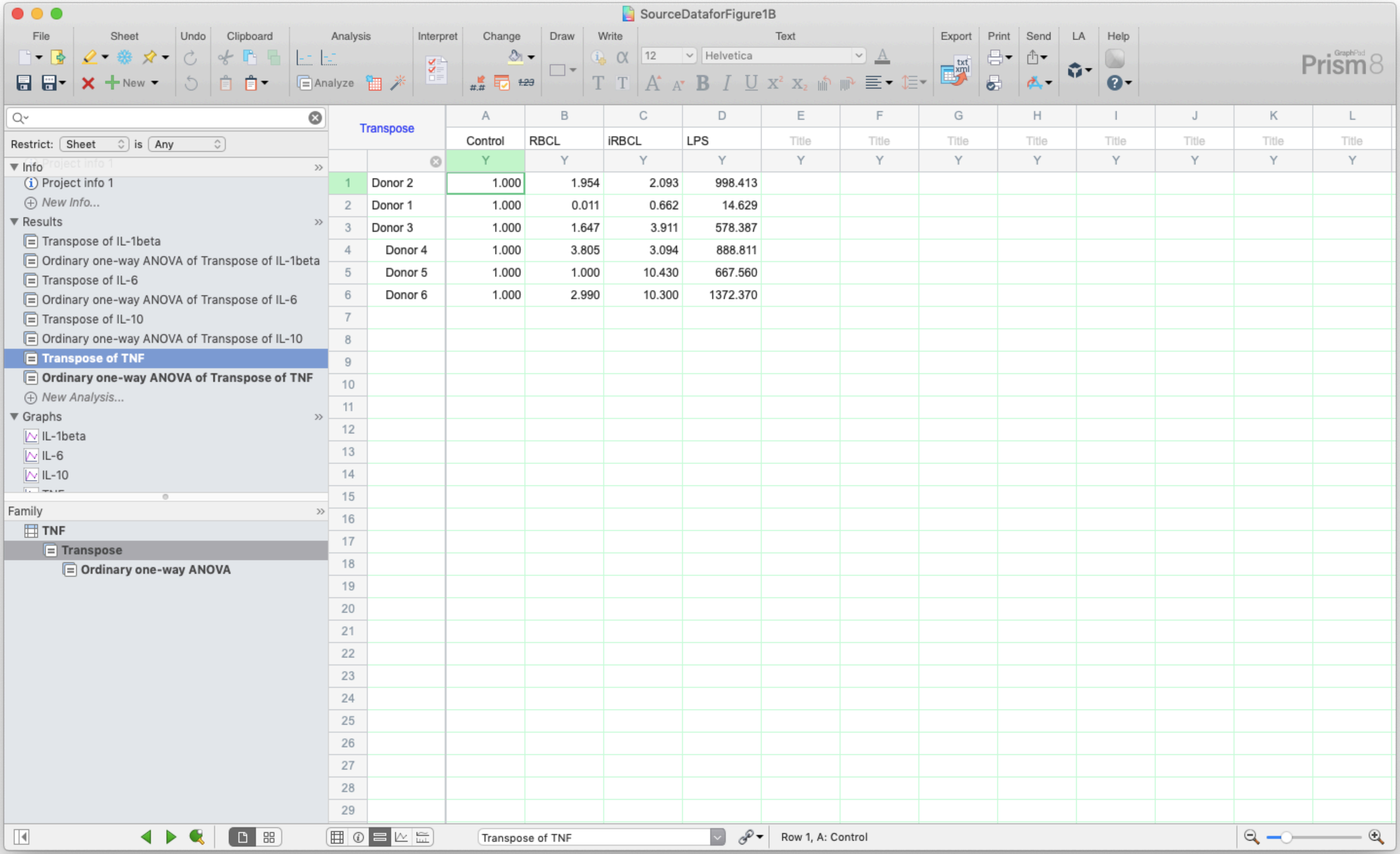

	Transpose	A Control	B RBCL	C iRBCL	D LPS
		Y	Y	Y	Y
1	Donor 2	1.000	1.954	2.093	998.413
2	Donor 1	1.000	0.011	0.662	14.629
3	Donor 3	1.000	1.647	3.911	578.387
4	Donor 4	1.000	3.805	3.094	888.811
5	Donor 5	1.000	1.000	10.430	667.560
6	Donor 6	1.000	2.990	10.300	1372.370

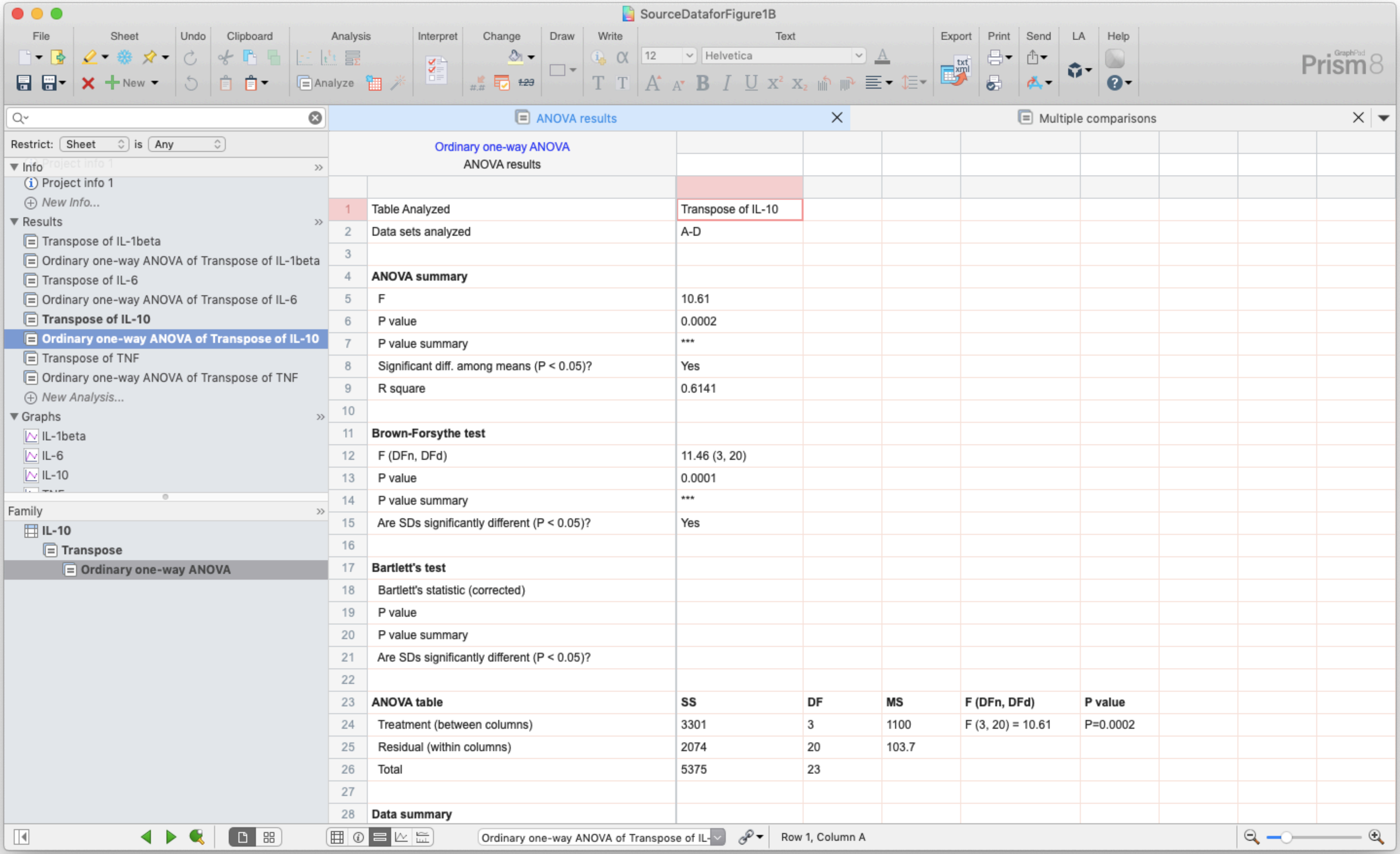
SourceDataforFigure1B

Ordinary one-way ANOVA
ANOVA results

1	Table Analyzed	Transpose of IL-10				
2	Data sets analyzed	A-D				
3						
4	**ANOVA summary**					
5	F	10.61				
6	P value	0.0002				
7	P value summary	***				
8	Significant diff. among means (P < 0.05)?	Yes				
9	R square	0.6141				
10						
11	**Brown-Forsythe test**					
12	F (DFn, DFd)	11.46 (3, 20)				
13	P value	0.0001				
14	P value summary	***				
15	Are SDs significantly different (P < 0.05)?	Yes				
16						
17	**Bartlett's test**					
18	Bartlett's statistic (corrected)					
19	P value					
20	P value summary					
21	Are SDs significantly different (P < 0.05)?					
22						
23	**ANOVA table**	**SS**	**DF**	**MS**	**F (DFn, DFd)**	**P value**
24	Treatment (between columns)	3301	3	1100	F (3, 20) = 10.61	P=0.0002
25	Residual (within columns)	2074	20	103.7		
26	Total	5375	23			
27						
28	**Data summary**					

SourceDataforFigure1B

ANOVA results | Multiple comparisons

Info
- Project info 1
- New Info...

Results
- Transpose of IL-1beta
- Ordinary one-way ANOVA of Transpose of IL-1beta
- Transpose of IL-6
- Ordinary one-way ANOVA of Transpose of IL-6
- **Transpose of IL-10**
- **Ordinary one-way ANOVA of Transpose of IL-10**
- Transpose of TNF
- Ordinary one-way ANOVA of Transpose of TNF
- New Analysis...

Graphs
- IL-1beta
- IL-6
- IL-10

Family
- **IL-10**
  - **Transpose**
    - **Ordinary one-way ANOVA**

Ordinary one-way ANOVA
Multiple comparisons

1	Number of families	1							
2	Number of comparisons per family	6							
3	Alpha	0.05							
4									
5	**Tukey's multiple comparisons test**	**Mean Diff.**	**95.00% CI of diff.**	**Significant?**	**Summary**	**Adjusted P Value**			
6	Control vs. RBCL	-0.1077	-16.56 to 16.35	No	ns	>0.9999	A-B		
7	Control vs. iRBCL	-0.3941	-16.85 to 16.06	No	ns	0.9999	A-C		
8	Control vs. LPS	-27.25	-43.71 to -10.79	Yes	***	0.0009	A-D		
9	RBCL vs. iRBCL	-0.2864	-16.74 to 16.17	No	ns	>0.9999	B-C		
10	RBCL vs. LPS	-27.14	-43.60 to -10.69	Yes	***	0.0009	B-D		
11	iRBCL vs. LPS	-26.86	-43.31 to -10.40	Yes	***	0.0010	C-D		
12									
13	**Test details**	**Mean 1**	**Mean 2**	**Mean Diff.**	**SE of diff.**	**n1**	**n2**	**q**	[illegible]
14	Control vs. RBCL	1.000	1.108	-0.1077	5.880	6	6	0.02591	[illegible]
15	Control vs. iRBCL	1.000	1.394	-0.3941	5.880	6	6	0.09479	[illegible]
16	Control vs. LPS	1.000	28.25	-27.25	5.880	6	6	6.554	[illegible]
17	RBCL vs. iRBCL	1.108	1.394	-0.2864	5.880	6	6	0.06889	[illegible]
18	RBCL vs. LPS	1.108	28.25	-27.14	5.880	6	6	6.528	[illegible]
19	iRBCL vs. LPS	1.394	28.25	-26.86	5.880	6	6	6.460	[illegible]

Ordinary one-way ANOVA of Transpose of IL- | Row 1, Column A

SourceDataforFigure1B

Ordinary one-way ANOVA
Multiple comparisons

1	Number of families							
2	Number of comparisons per family							
3	Alpha							
4								
5	**Tukey's multiple comparisons test**	**95.00% CI of diff.**	**Significant?**	**Summary**	**Adjusted P Value**			
6	Control vs. RBCL	-370.3 to 368.5	No	ns	>0.9999	A-B		
7	Control vs. iRBCL	-373.5 to 365.4	No	ns	>0.9999	A-C		
8	Control vs. LPS	-1122 to -382.9	Yes	****	<0.0001	A-D		
9	RBCL vs. iRBCL	-372.6 to 366.3	No	ns	>0.9999	B-C		
10	RBCL vs. LPS	-1121 to -382.0	Yes	****	<0.0001	B-D		
11	iRBCL vs. LPS	-1118 to -378.8	Yes	****	<0.0001	C-D		
12								
13	**Test details**	**Mean 2**	**Mean Diff.**	**SE of diff.**	**n1**	**n2**	**q**	**DF**
14	Control vs. RBCL	1.901	-0.9013	132.0	6	6	0.009656	20
15	Control vs. iRBCL	5.082	-4.082	132.0	6	6	0.04373	20
16	Control vs. LPS	753.4	-752.4	132.0	6	6	8.061	20
17	RBCL vs. iRBCL	5.082	-3.180	132.0	6	6	0.03408	20
18	RBCL vs. LPS	753.4	-751.5	132.0	6	6	8.051	20
19	iRBCL vs. LPS	753.4	-748.3	132.0	6	6	8.017	20

SourceDataforFigure1B

ANVOA results | Multiple comparisons

Info
- Project info 1
- New Info...

Results
- Transpose of IL-1beta
- Ordinary one-way ANOVA of Transpose of IL-1beta
- Transpose of IL-6
- Ordinary one-way ANOVA of Transpose of IL-6
- Transpose of IL-10
- Ordinary one-way ANOVA of Transpose of IL-10
- **Transpose of TNF**
- **Ordinary one-way ANOVA of Transpose of TNF**
- New Analysis...

Graphs
- IL-1beta
- IL-6
- IL-10

Family
- **TNF**
  - **Transpose**
    - **Ordinary one-way ANOVA**

	Ordinary one-way ANOVA<br>ANOVA results					
1	Table Analyzed	Transpose of TNF				
2	Data sets analyzed	A-D				
3						
4	**ANOVA summary**					
5	F	16.17				
6	P value	<0.0001				
7	P value summary	****				
8	Significant diff. among means (P < 0.05)?	Yes				
9	R square	0.7081				
10						
11	**Brown-Forsythe test**					
12	F (DFn, DFd)	8.623 (3, 20)				
13	P value	0.0007				
14	P value summary	***				
15	Are SDs significantly different (P < 0.05)?	Yes				
16						
17	**Bartlett's test**					
18	Bartlett's statistic (corrected)					
19	P value					
20	P value summary					
21	Are SDs significantly different (P < 0.05)?					
22						
23	**ANOVA table**	**SS**	**DF**	**MS**	**F (DFn, DFd)**	**P value**
24	Treatment (between columns)	2536037	3	845346	F (3, 20) = 16.17	P<0.0001
25	Residual (within columns)	1045371	20	52269		
26	Total	3581408	23			
27						
28	**Data summary**					

Ordinary one-way ANOVA of Transpose of TNF | Row 1, Column A

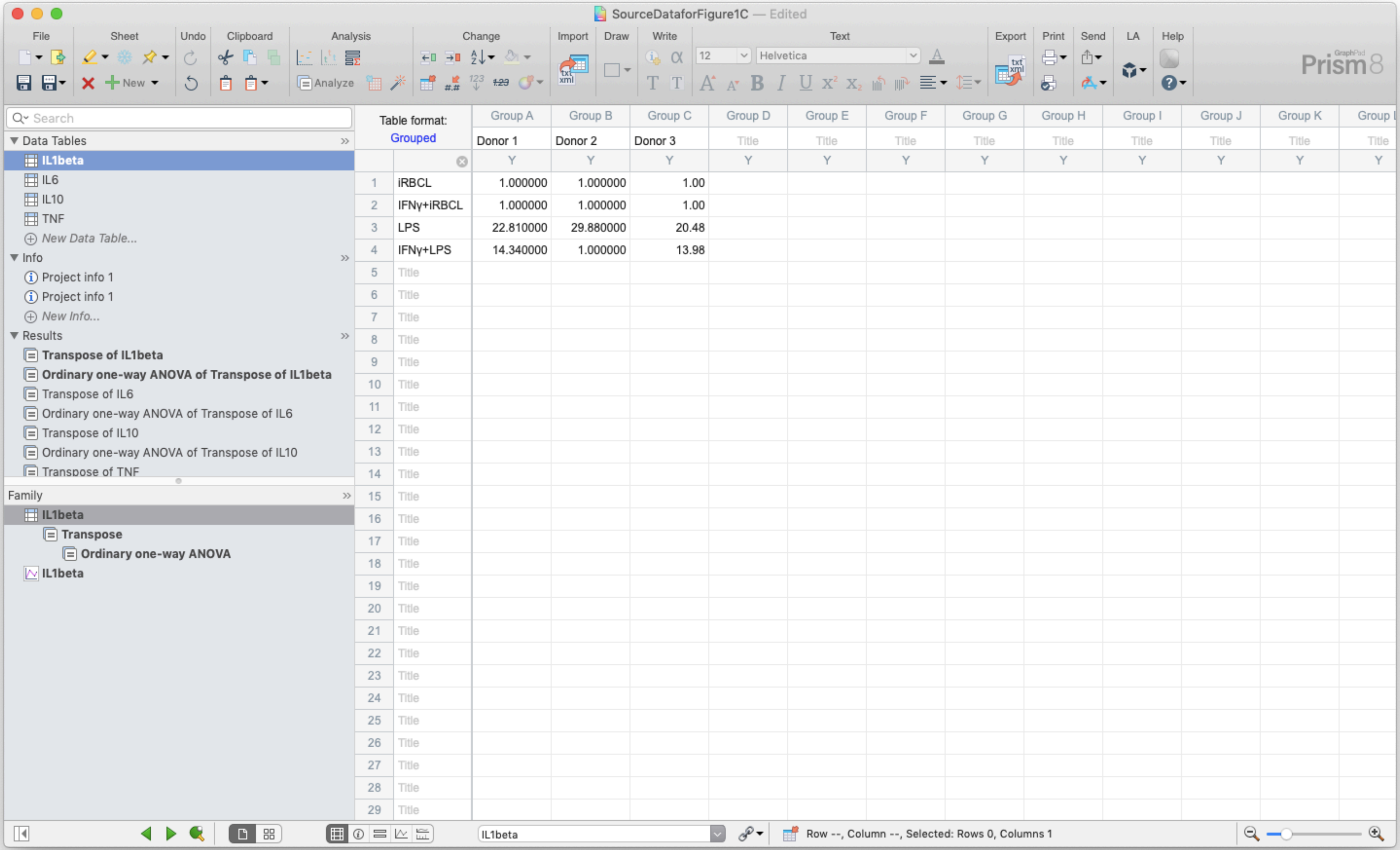

	Group A	Group B	Group C
	Donor 1	Donor 2	Donor 3
	Y	Y	Y
iRBCL	1.000000	1.000000	1.00
IFNγ+iRBCL	1.000000	1.000000	1.00
LPS	22.810000	29.880000	20.48
IFNγ+LPS	14.340000	1.000000	13.98

SourceDataforFigure1C — Edited

Data Tables
- IL1beta
- IL6
- IL10
- TNF
- New Data Table...

Info
- Project info 1
- Project info 1
- New Info...

Results
- Transpose of IL1beta
- Ordinary one-way ANOVA of Transpose of IL1beta
- Transpose of IL6
- Ordinary one-way ANOVA of Transpose of IL6
- Transpose of IL10
- Ordinary one-way ANOVA of Transpose of IL10
- Transpose of TNF

Family
- IL6
  - Transpose
    - Ordinary one-way ANOVA
- IL6

Table format: Grouped

		Group A	Group B	Group C
		Donor 1	Donor 2	Donor 3
		Y	Y	Y
1	iRBCL	2.112069	2.960000	12.28
2	IFNγ+iRBCL	7.071839	36.340000	43.84
3	LPS	1435.457000	5126.320000	10896.21
4	IFNγ+LPS	2782.621000	8085.630000	15510.43

Row --, Column --, Selected: Rows 0, Columns 1

SourceDataforFigure1C — Edited

Data Tables
- IL1beta
- IL6
- IL10
- TNF
- New Data Table...

Info
- Project info 1
- Project info 1
- New Info...

Results
- Transpose of IL1beta
- Ordinary one-way ANOVA of Transpose of IL1beta
- Transpose of IL6
- Ordinary one-way ANOVA of Transpose of IL6
- **Transpose of IL10**
- **Ordinary one-way ANOVA of Transpose of IL10**
- Transpose of TNF

Family
- **IL10**
  - **Transpose**
    - **Ordinary one-way ANOVA**
- **IL10**

Table format: Grouped

		Group A	Group B	Group C
		Donor 1	Donor 2	Donor 3
		Y	Y	Y
1	iRBCL	1.000000	1.000000	2.41
2	IFNγ+iRBCL	1.000000	1.000000	1.00
3	LPS	13.510000	33.740000	35.26
4	IFNγ+LPS	6.460000	7.600000	31.44

IL10

Row --, Column --, Selected: Rows 0, Columns 1

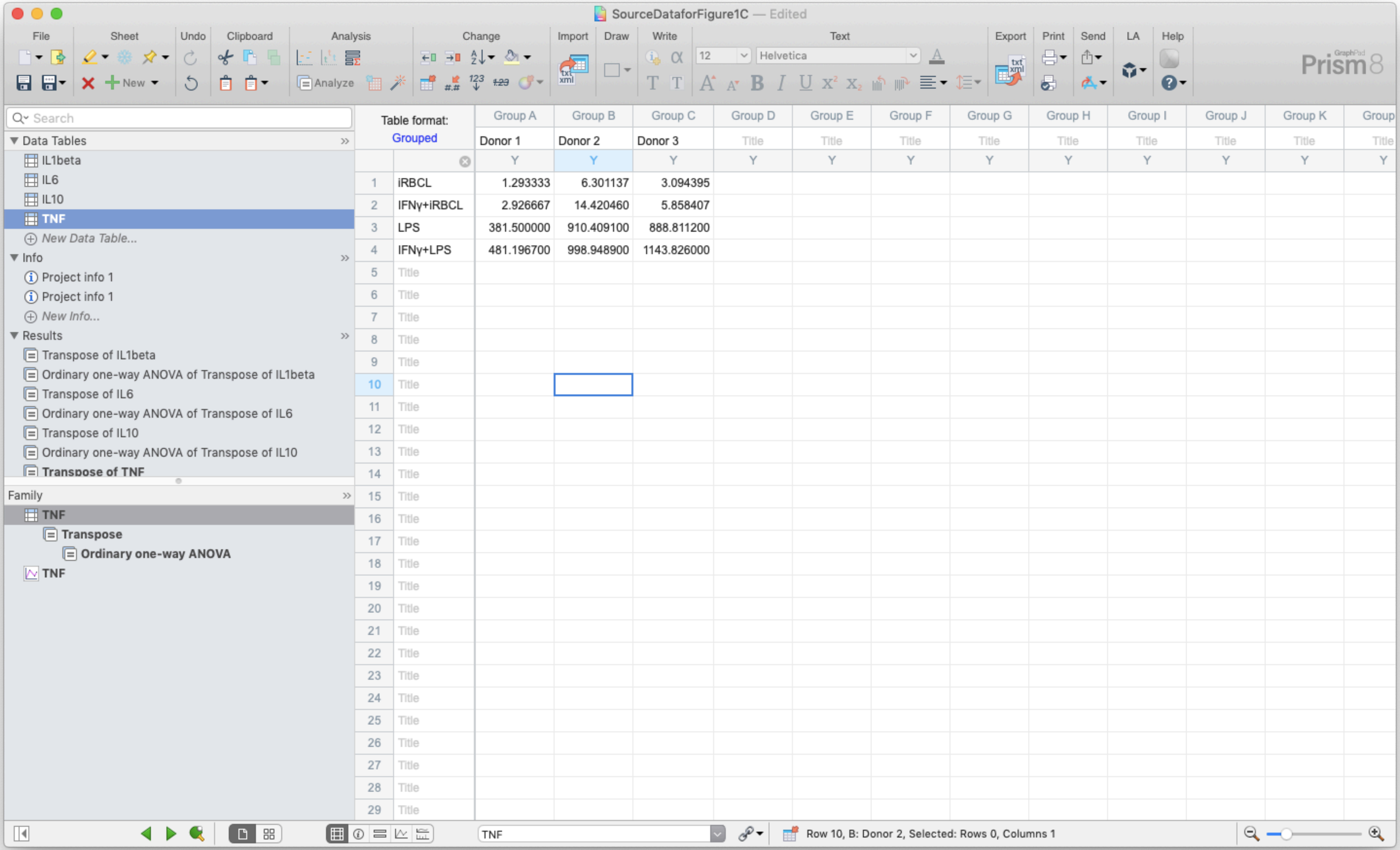

		Group A	Group B	Group C
		Donor 1	Donor 2	Donor 3
1	iRBCL	1.293333	6.301137	3.094395
2	IFNγ+iRBCL	2.926667	14.420460	5.858407
3	LPS	381.500000	910.409100	888.811200
4	IFNγ+LPS	481.196700	998.948900	1143.826000

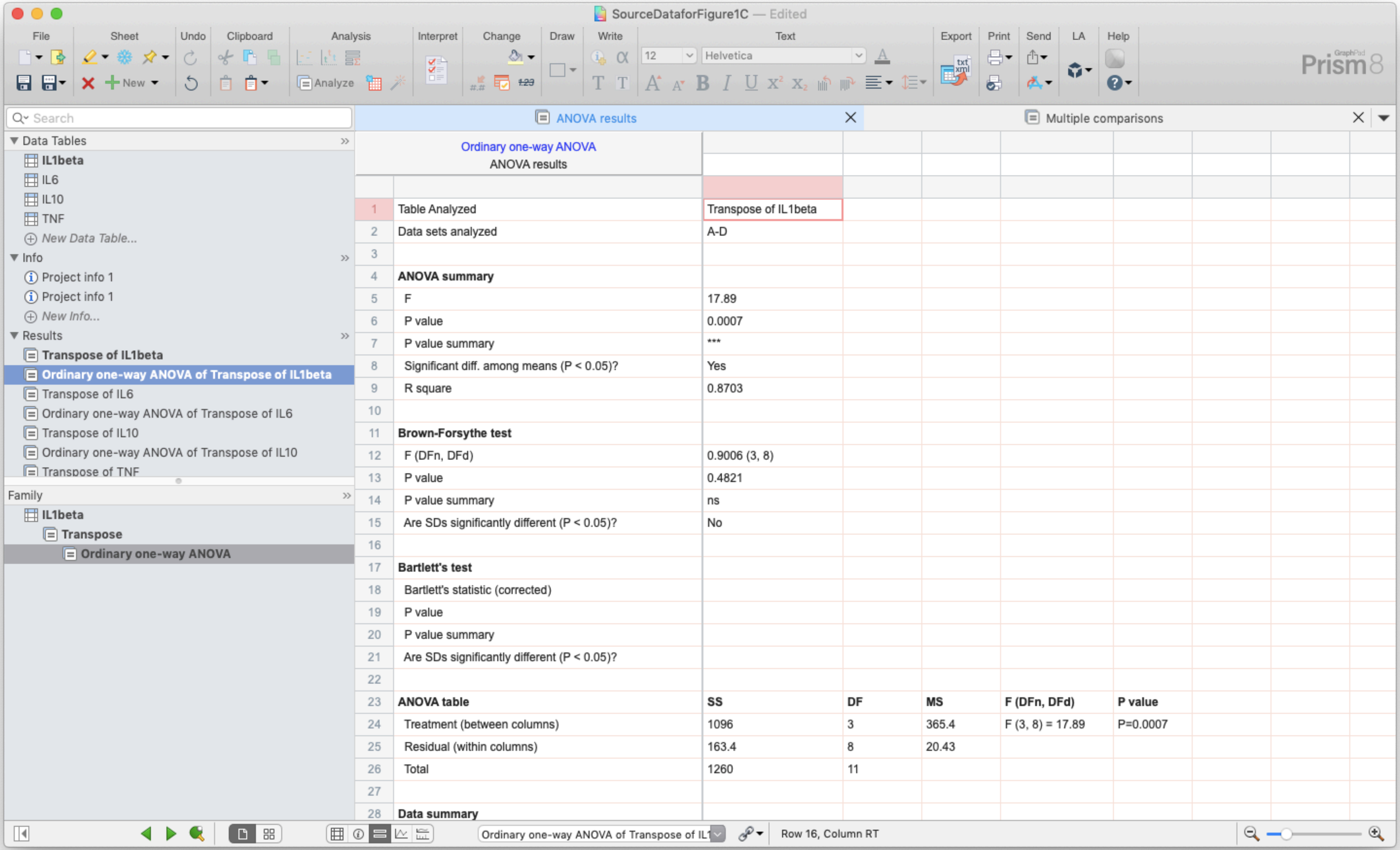
SourceDataforFigure1C — Edited

Ordinary one-way ANOVA
ANOVA results

	ANOVA results					
1	Table Analyzed	Transpose of IL1beta				
2	Data sets analyzed	A-D				
3						
4	**ANOVA summary**					
5	F	17.89				
6	P value	0.0007				
7	P value summary	***				
8	Significant diff. among means (P < 0.05)?	Yes				
9	R square	0.8703				
10						
11	**Brown-Forsythe test**					
12	F (DFn, DFd)	0.9006 (3, 8)				
13	P value	0.4821				
14	P value summary	ns				
15	Are SDs significantly different (P < 0.05)?	No				
16						
17	**Bartlett's test**					
18	Bartlett's statistic (corrected)					
19	P value					
20	P value summary					
21	Are SDs significantly different (P < 0.05)?					
22						
23	**ANOVA table**	**SS**	**DF**	**MS**	**F (DFn, DFd)**	**P value**
24	Treatment (between columns)	1096	3	365.4	F (3, 8) = 17.89	P=0.0007
25	Residual (within columns)	163.4	8	20.43		
26	Total	1260	11			
27						
28	**Data summary**					

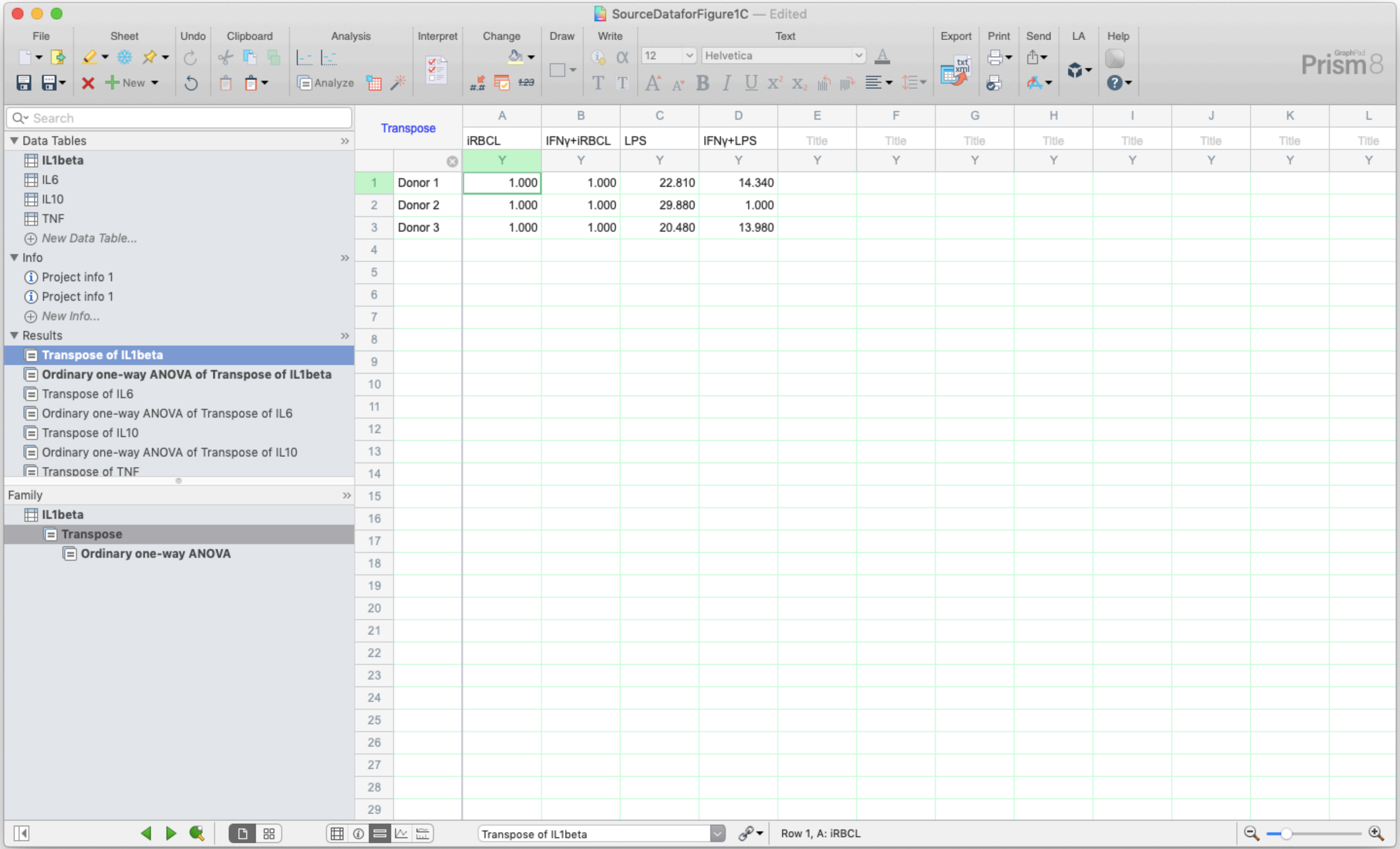

Transpose	iRBCL	IFNγ+iRBCL	LPS	IFNγ+LPS
Donor 1	1.000	1.000	22.810	14.340
Donor 2	1.000	1.000	29.880	1.000
Donor 3	1.000	1.000	20.480	13.980

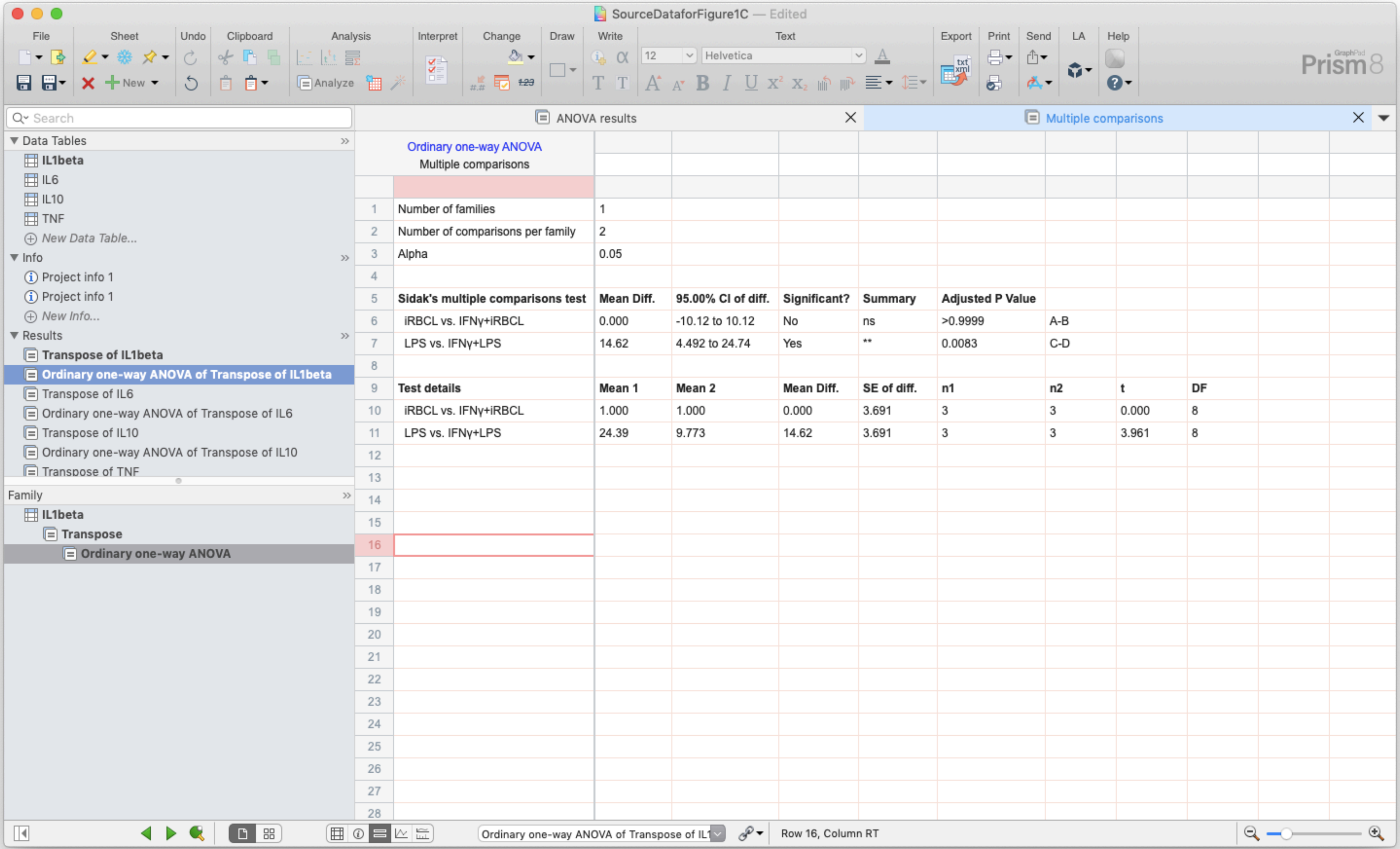

Ordinary one-way ANOVA
Multiple comparisons

1	Number of families	1								
2	Number of comparisons per family	2								
3	Alpha	0.05								
4										
5	**Sidak's multiple comparisons test**	**Mean Diff.**	**95.00% CI of diff.**	**Significant?**	**Summary**	**Adjusted P Value**				
6	iRBCL vs. IFNγ+iRBCL	0.000	-10.12 to 10.12	No	ns	>0.9999	A-B			
7	LPS vs. IFNγ+LPS	14.62	4.492 to 24.74	Yes	**	0.0083	C-D			
8										
9	**Test details**	**Mean 1**	**Mean 2**	**Mean Diff.**	**SE of diff.**	**n1**	**n2**	**t**	**DF**	
10	iRBCL vs. IFNγ+iRBCL	1.000	1.000	0.000	3.691	3	3	0.000	8	
11	LPS vs. IFNγ+LPS	24.39	9.773	14.62	3.691	3	3	3.961	8	

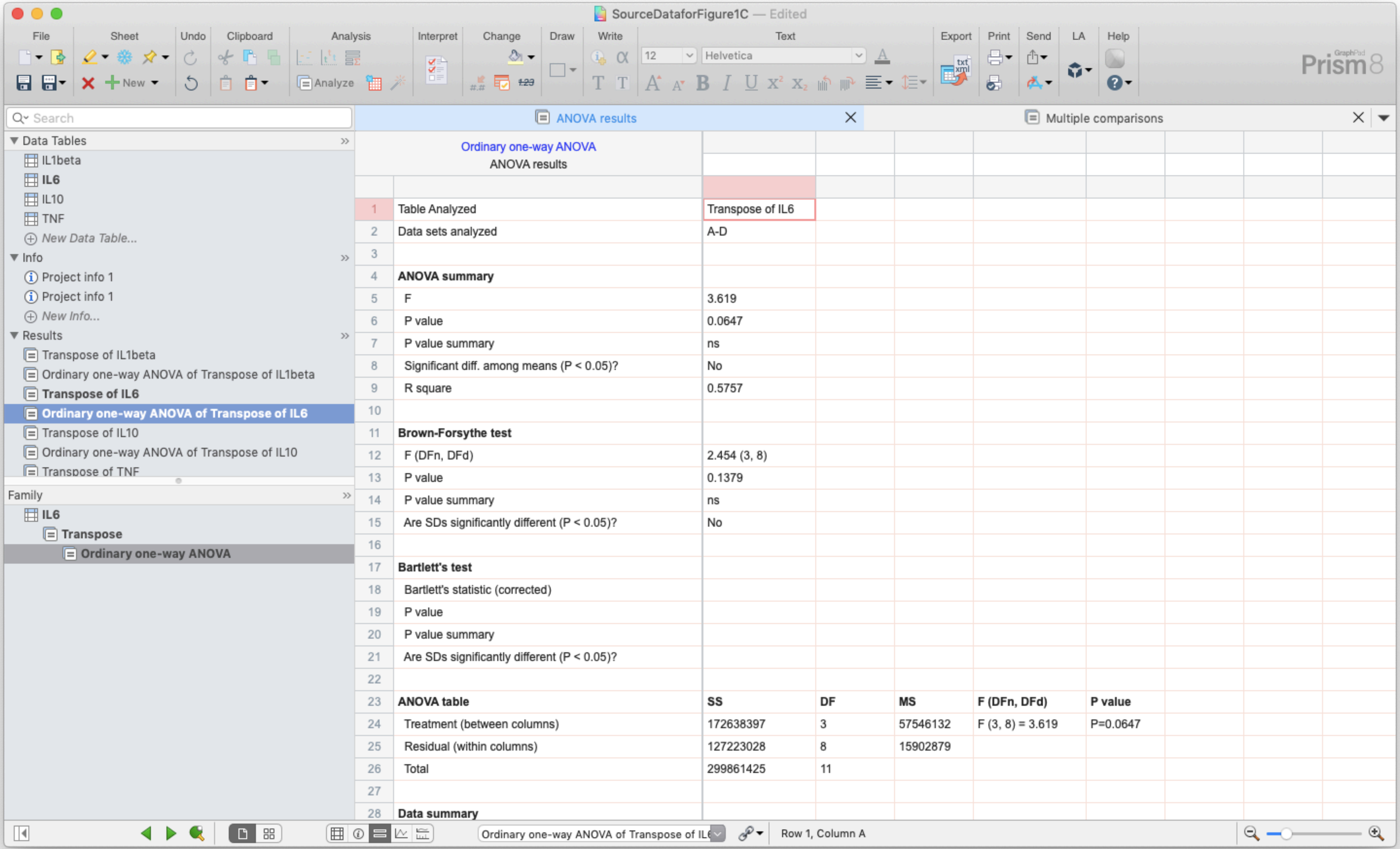

Ordinary one-way ANOVA
ANOVA results

1	Table Analyzed	Transpose of IL6				
2	Data sets analyzed	A-D				
3						
4	**ANOVA summary**					
5	F	3.619				
6	P value	0.0647				
7	P value summary	ns				
8	Significant diff. among means (P < 0.05)?	No				
9	R square	0.5757				
10						
11	**Brown-Forsythe test**					
12	F (DFn, DFd)	2.454 (3, 8)				
13	P value	0.1379				
14	P value summary	ns				
15	Are SDs significantly different (P < 0.05)?	No				
16						
17	**Bartlett's test**					
18	Bartlett's statistic (corrected)					
19	P value					
20	P value summary					
21	Are SDs significantly different (P < 0.05)?					
22						
23	**ANOVA table**	**SS**	**DF**	**MS**	**F (DFn, DFd)**	**P value**
24	Treatment (between columns)	172638397	3	57546132	F (3, 8) = 3.619	P=0.0647
25	Residual (within columns)	127223028	8	15902879		
26	Total	299861425	11			
27						
28	**Data summary**					

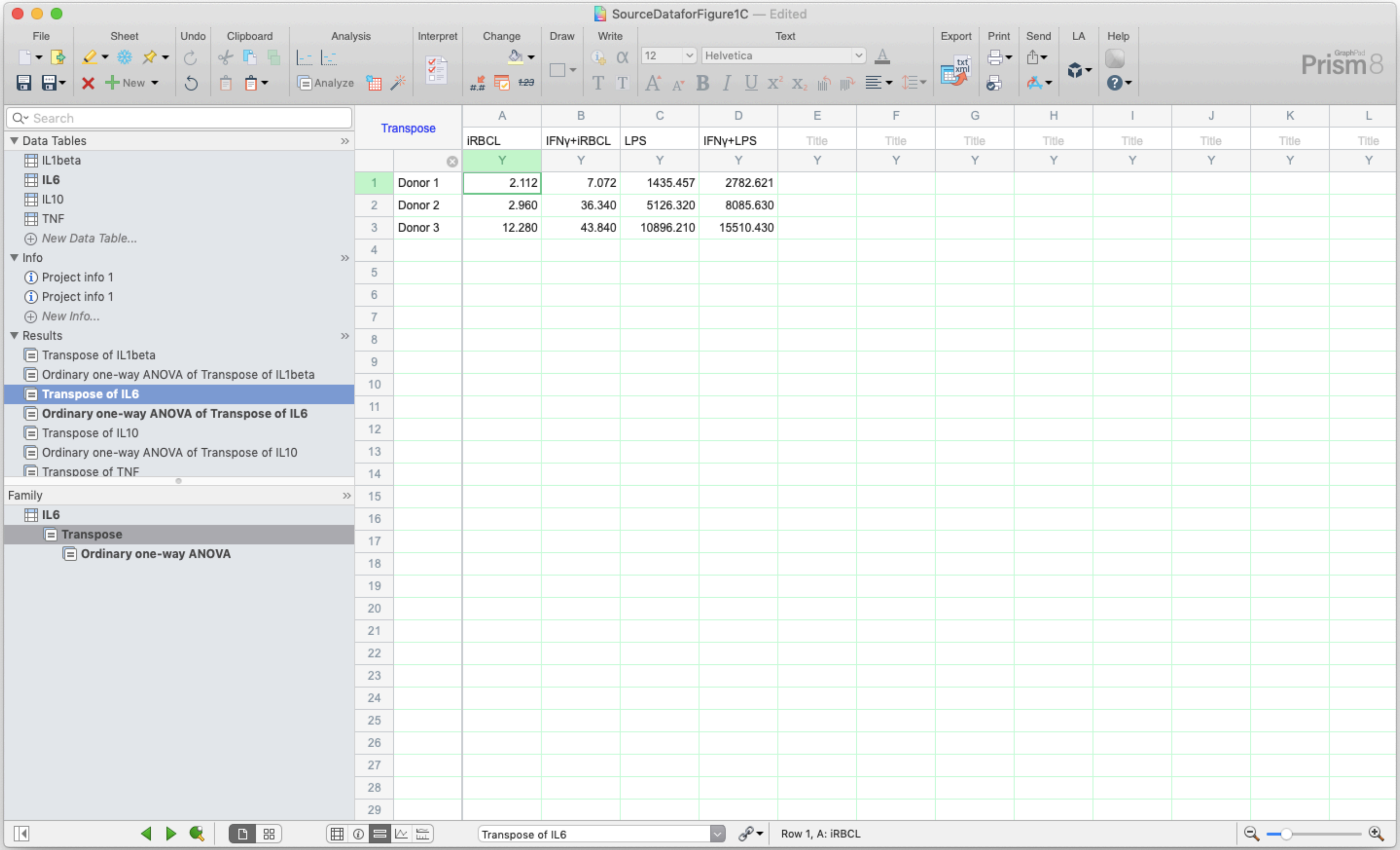

Transpose	iRBCL	IFNγ+iRBCL	LPS	IFNγ+LPS
	Y	Y	Y	Y
Donor 1	2.112	7.072	1435.457	2782.621
Donor 2	2.960	36.340	5126.320	8085.630
Donor 3	12.280	43.840	10896.210	15510.430

SourceDataforFigure1C — Edited

Data Tables
- IL1beta
- IL6
- IL10
- TNF
- New Data Table...

Info
- Project info 1
- Project info 1
- New Info...

Results
- Transpose of IL1beta
- Ordinary one-way ANOVA of Transpose of IL1beta
- Transpose of IL6
- Ordinary one-way ANOVA of Transpose of IL6
- Transpose of IL10
- Ordinary one-way ANOVA of Transpose of IL10
- Transpose of TNF

Family
- IL10
  - Transpose
    - Ordinary one-way ANOVA

Transpose	A: iRBCL (Y)	B: IFNγ+iRBCL (Y)	C: LPS (Y)	D: IFNγ+LPS (Y)
Donor 1	1.000	1.000	13.510	6.460
Donor 2	1.000	1.000	33.740	7.600
Donor 3	2.410	1.000	35.260	31.440

Transpose of IL10

Row 1, A: iRBCL

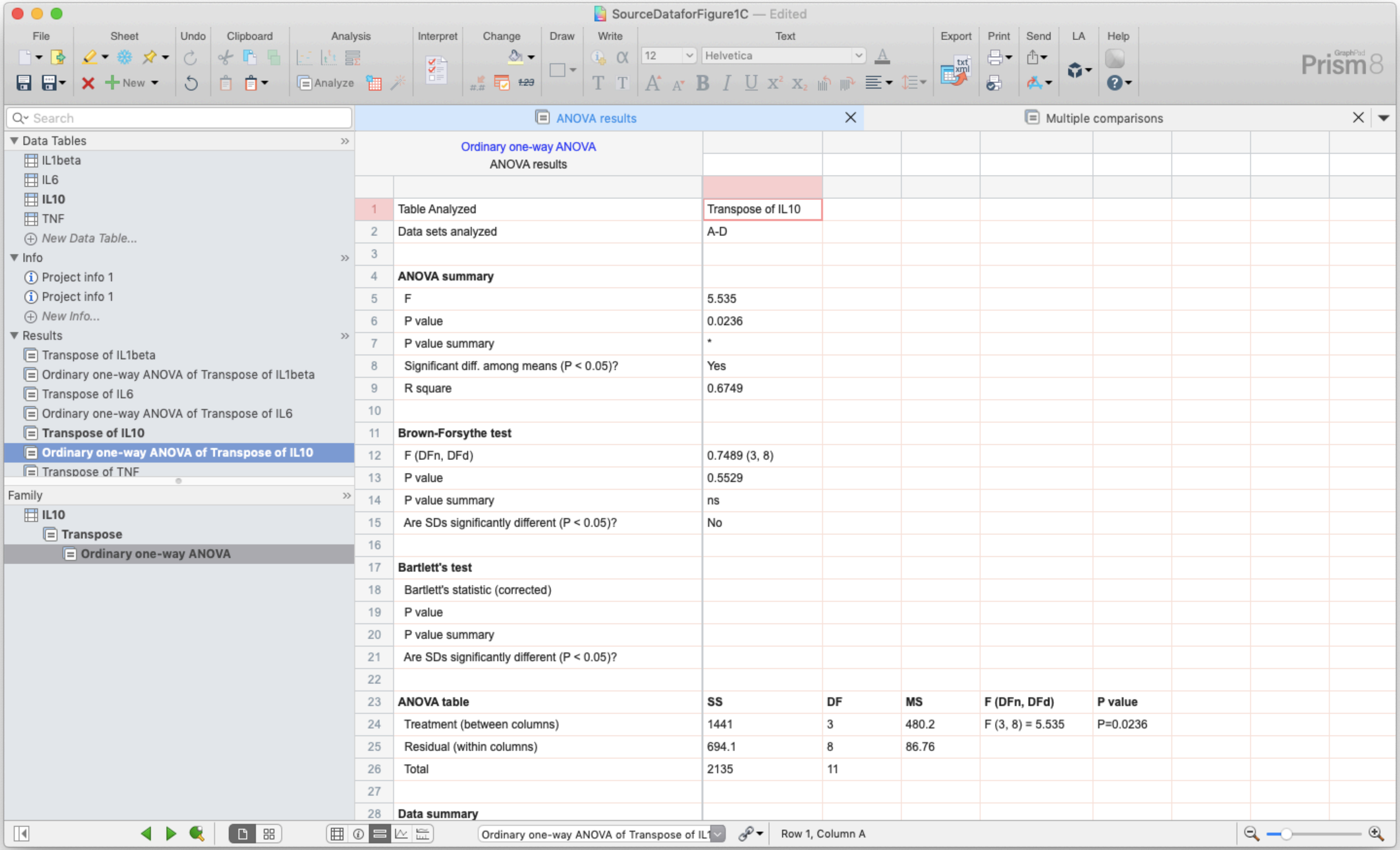

Ordinary one-way ANOVA
ANOVA results

1	Table Analyzed	Transpose of IL10				
2	Data sets analyzed	A-D				
3						
4	**ANOVA summary**					
5	F	5.535				
6	P value	0.0236				
7	P value summary	*				
8	Significant diff. among means (P < 0.05)?	Yes				
9	R square	0.6749				
10						
11	**Brown-Forsythe test**					
12	F (DFn, DFd)	0.7489 (3, 8)				
13	P value	0.5529				
14	P value summary	ns				
15	Are SDs significantly different (P < 0.05)?	No				
16						
17	**Bartlett's test**					
18	Bartlett's statistic (corrected)					
19	P value					
20	P value summary					
21	Are SDs significantly different (P < 0.05)?					
22						
23	**ANOVA table**	**SS**	**DF**	**MS**	**F (DFn, DFd)**	**P value**
24	Treatment (between columns)	1441	3	480.2	F (3, 8) = 5.535	P=0.0236
25	Residual (within columns)	694.1	8	86.76		
26	Total	2135	11			
27						
28	**Data summary**					

SourceDataforFigure1C — Edited

File Sheet Undo Clipboard Analysis Interpret Change Draw Write Text Export Print Send LA Help

Prism 8

Data Tables
- IL1beta
- **IL6**
- IL10
- TNF
- New Data Table...

Info
- Project info 1
- Project info 1
- New Info...

Results
- Transpose of IL1beta
- Ordinary one-way ANOVA of Transpose of IL1beta
- **Transpose of IL6**
- **Ordinary one-way ANOVA of Transpose of IL6**
- Transpose of IL10
- Ordinary one-way ANOVA of Transpose of IL10
- Transpose of TNF

Family
- **IL6**
- **Transpose**
- **Ordinary one-way ANOVA**

ANOVA results | Multiple comparisons

Ordinary one-way ANOVA
Multiple comparisons

1	Number of families	1							
2	Number of comparisons per family	2							
3	Alpha	0.05							
4									
5	**Sidak's multiple comparisons test**	**Mean Diff.**	**95.00% CI of diff.**	**Significant?**	**Summary**	**Adjusted P Value**			
6	iRBCL vs. IFNγ+iRBCL	-23.30	-8956 to 8909	No	ns	>0.9999	A-B		
7	LPS vs. IFNγ+LPS	-2974	-11906 to 5959	No	ns	0.6252	C-D		
8									
9	**Test details**	**Mean 1**	**Mean 2**	**Mean Diff.**	**SE of diff.**	**n1**	**n2**	**t**	**DF**
10	iRBCL vs. IFNγ+iRBCL	5.784	29.08	-23.30	3256	3	3	0.007156	8
11	LPS vs. IFNγ+LPS	5819	8793	-2974	3256	3	3	0.9132	8

Ordinary one-way ANOVA of Transpose of IL6 — Row 1, Column A

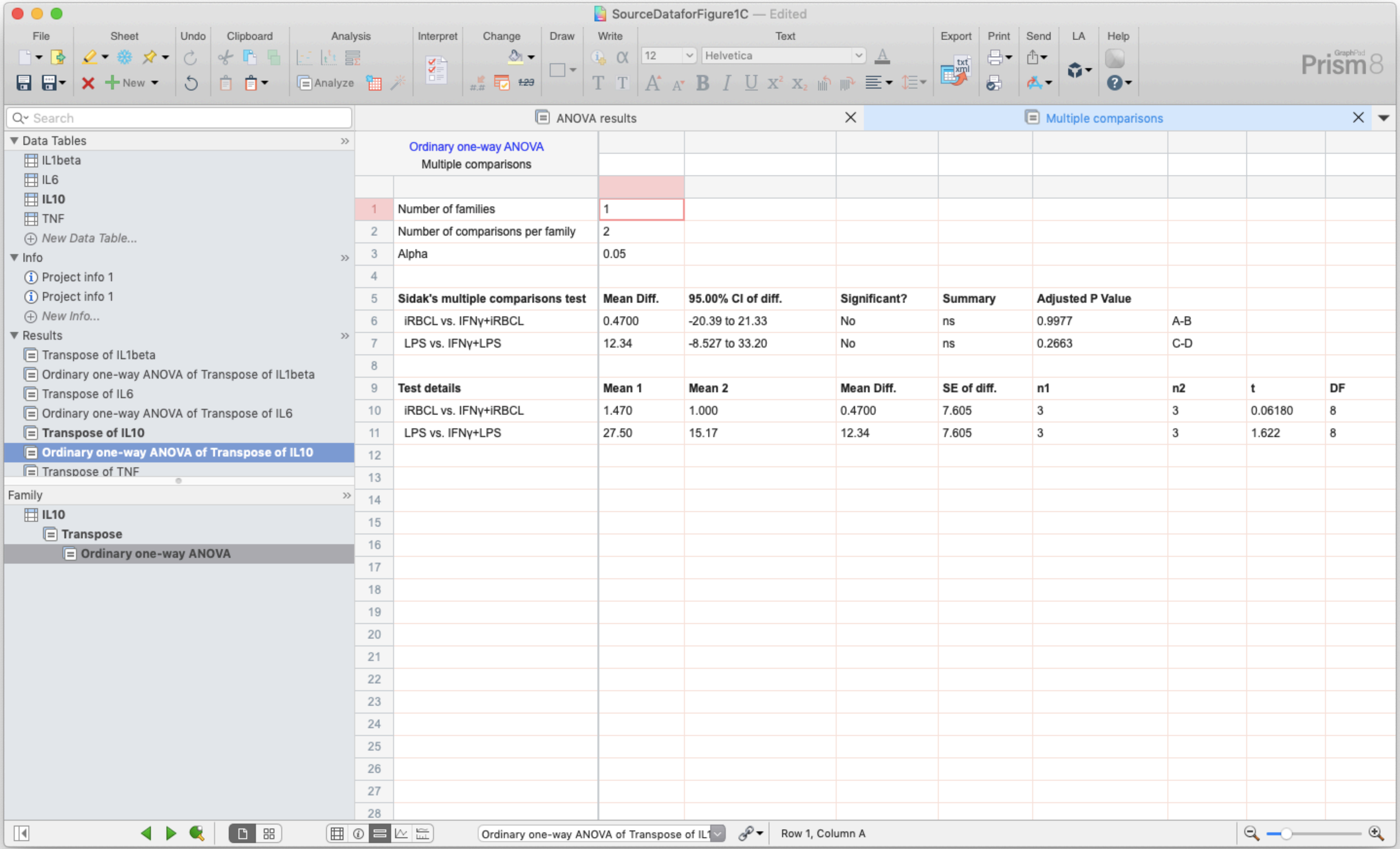

Ordinary one-way ANOVA
Multiple comparisons

1	Number of families	1							
2	Number of comparisons per family	2							
3	Alpha	0.05							
4									
5	**Sidak's multiple comparisons test**	**Mean Diff.**	**95.00% CI of diff.**	**Significant?**	**Summary**	**Adjusted P Value**			
6	iRBCL vs. IFNγ+iRBCL	0.4700	-20.39 to 21.33	No	ns	0.9977	A-B		
7	LPS vs. IFNγ+LPS	12.34	-8.527 to 33.20	No	ns	0.2663	C-D		
8									
9	**Test details**	**Mean 1**	**Mean 2**	**Mean Diff.**	**SE of diff.**	**n1**	**n2**	**t**	**DF**
10	iRBCL vs. IFNγ+iRBCL	1.470	1.000	0.4700	7.605	3	3	0.06180	8
11	LPS vs. IFNγ+LPS	27.50	15.17	12.34	7.605	3	3	1.622	8

SourceDataforFigure1C — Edited

ANOVA results

Ordinary one-way ANOVA
ANOVA results

1	Table Analyzed	Transpose of TNF				
2	Data sets analyzed	A-D				
3						
4	**ANOVA summary**					
5	F	12.19				
6	P value	0.0024				
7	P value summary	**				
8	Significant diff. among means (P < 0.05)?	Yes				
9	R square	0.8205				
10						
11	**Brown-Forsythe test**					
12	F (DFn, DFd)	1.024 (3, 8)				
13	P value	0.4317				
14	P value summary	ns				
15	Are SDs significantly different (P < 0.05)?	No				
16						
17	**Bartlett's test**					
18	Bartlett's statistic (corrected)					
19	P value					
20	P value summary					
21	Are SDs significantly different (P < 0.05)?					
22						
23	**ANOVA table**	**SS**	**DF**	**MS**	**F (DFn, DFd)**	**P value**
24	Treatment (between columns)	1929480	3	643160	F (3, 8) = 12.19	P=0.0024
25	Residual (within columns)	421988	8	52748		
26	Total	2351468	11			
27						
28	**Data summary**					

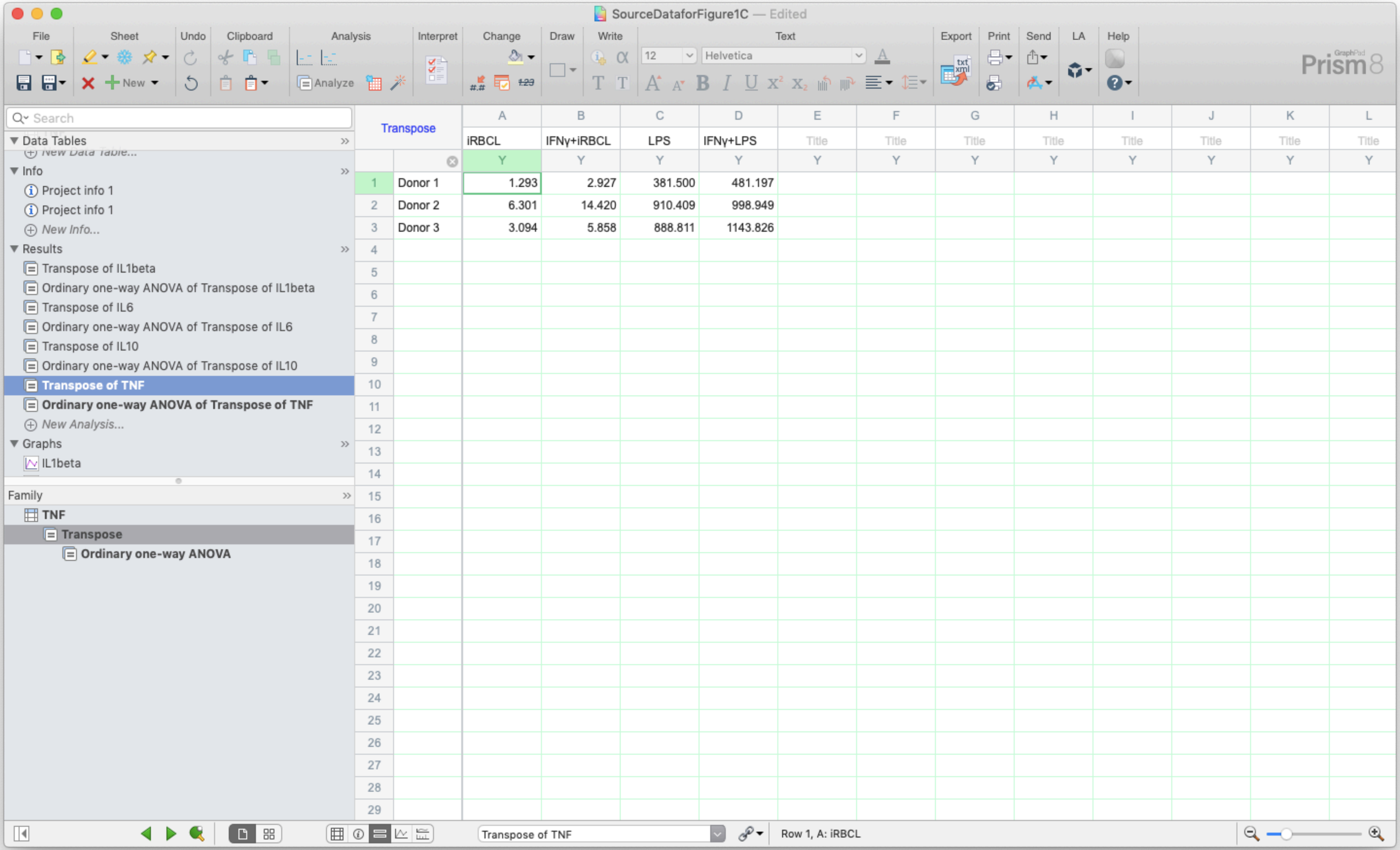

Transpose	iRBCL	IFNγ+iRBCL	LPS	IFNγ+LPS
Donor 1	1.293	2.927	381.500	481.197
Donor 2	6.301	14.420	910.409	998.949
Donor 3	3.094	5.858	888.811	1143.826

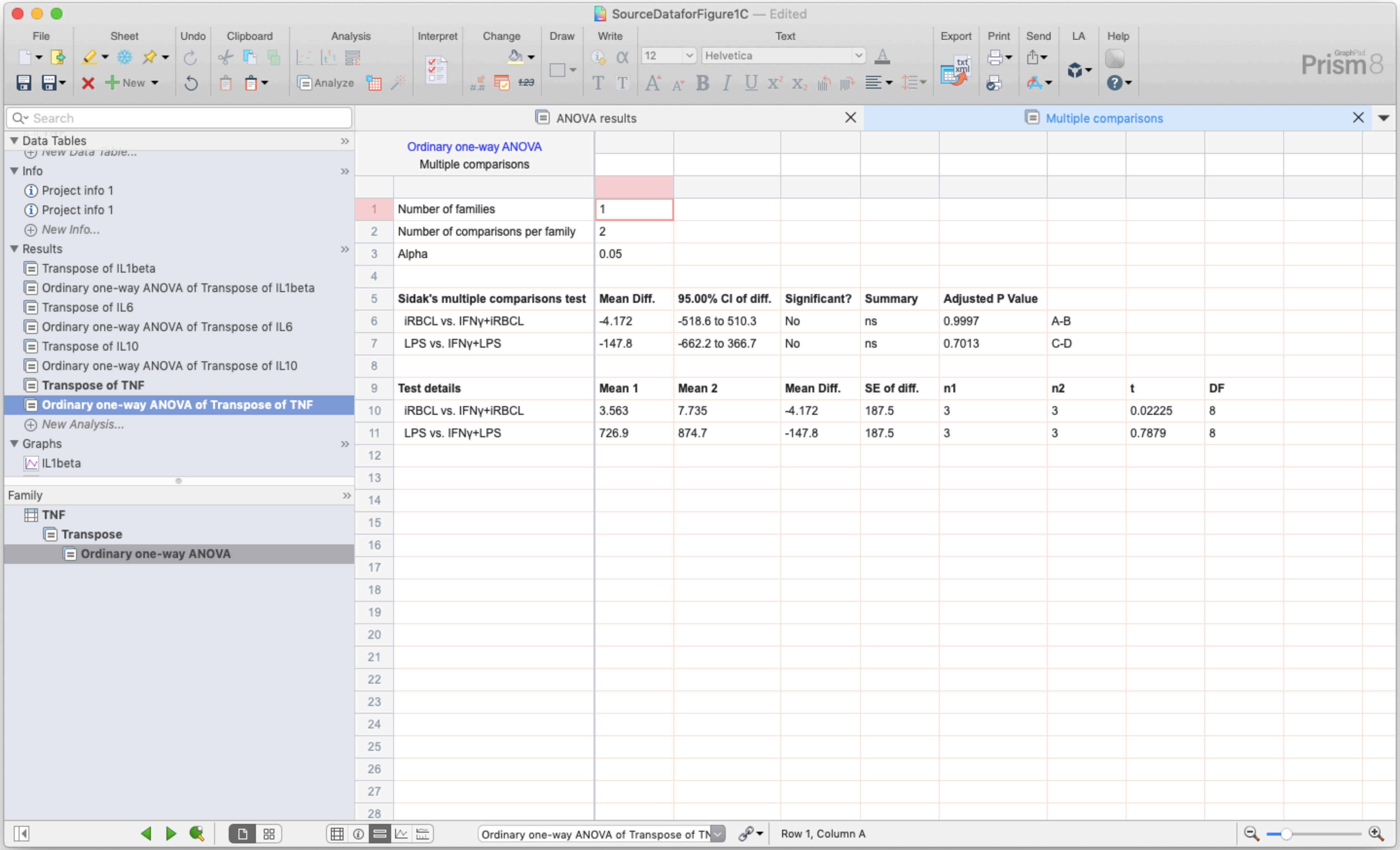

Ordinary one-way ANOVA
Multiple comparisons

1	Number of families	1							
2	Number of comparisons per family	2							
3	Alpha	0.05							
4									
5	**Sidak's multiple comparisons test**	**Mean Diff.**	**95.00% CI of diff.**	**Significant?**	**Summary**	**Adjusted P Value**			
6	iRBCL vs. IFNγ+iRBCL	-4.172	-518.6 to 510.3	No	ns	0.9997	A-B		
7	LPS vs. IFNγ+LPS	-147.8	-662.2 to 366.7	No	ns	0.7013	C-D		
8									
9	**Test details**	**Mean 1**	**Mean 2**	**Mean Diff.**	**SE of diff.**	**n1**	**n2**	**t**	**DF**
10	iRBCL vs. IFNγ+iRBCL	3.563	7.735	-4.172	187.5	3	3	0.02225	8
11	LPS vs. IFNγ+LPS	726.9	874.7	-147.8	187.5	3	3	0.7879	8

Control

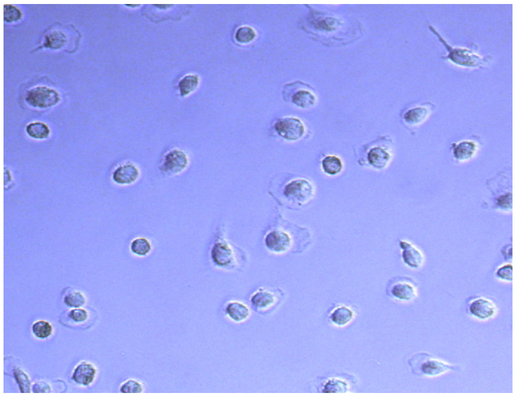

RBC

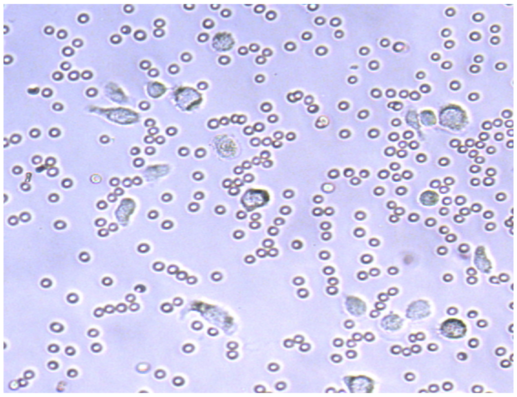

iRBC

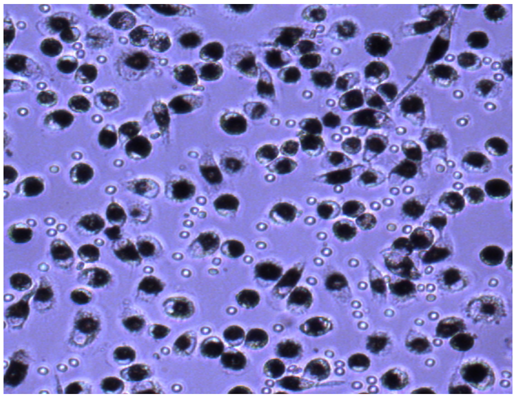

LPS

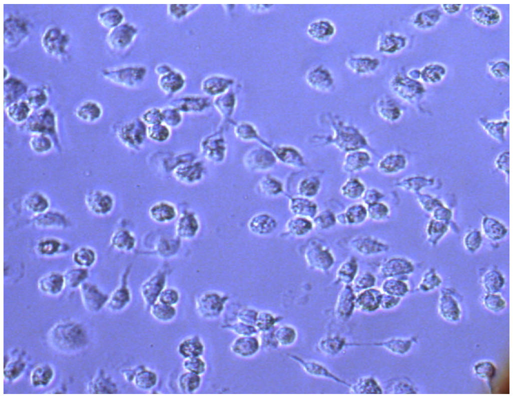

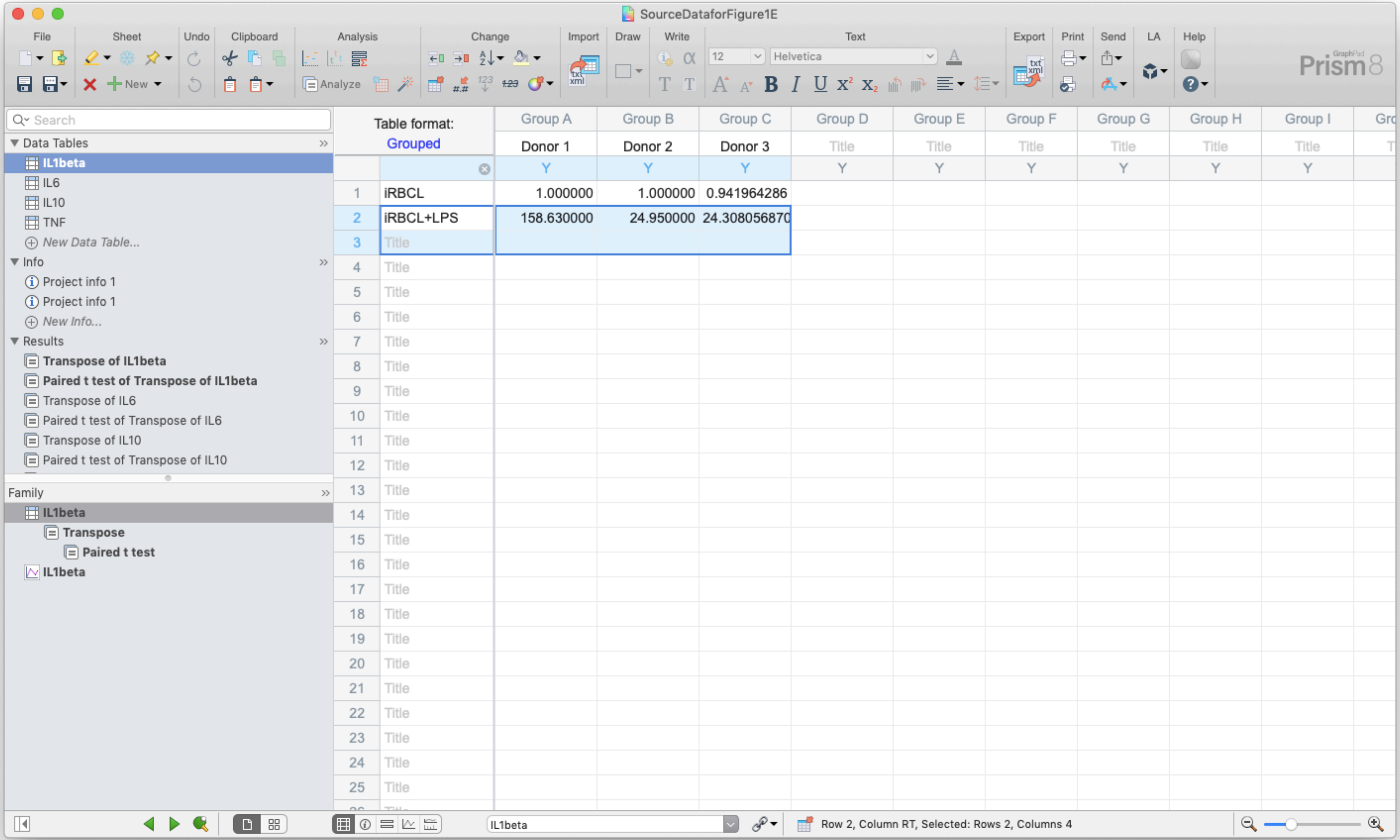

	Table format: Grouped	Group A	Group B	Group C
		Donor 1	Donor 2	Donor 3
		Y	Y	Y
1	iRBCL	1.000000	1.000000	0.941964286
2	iRBCL+LPS	158.630000	24.950000	24.308056870

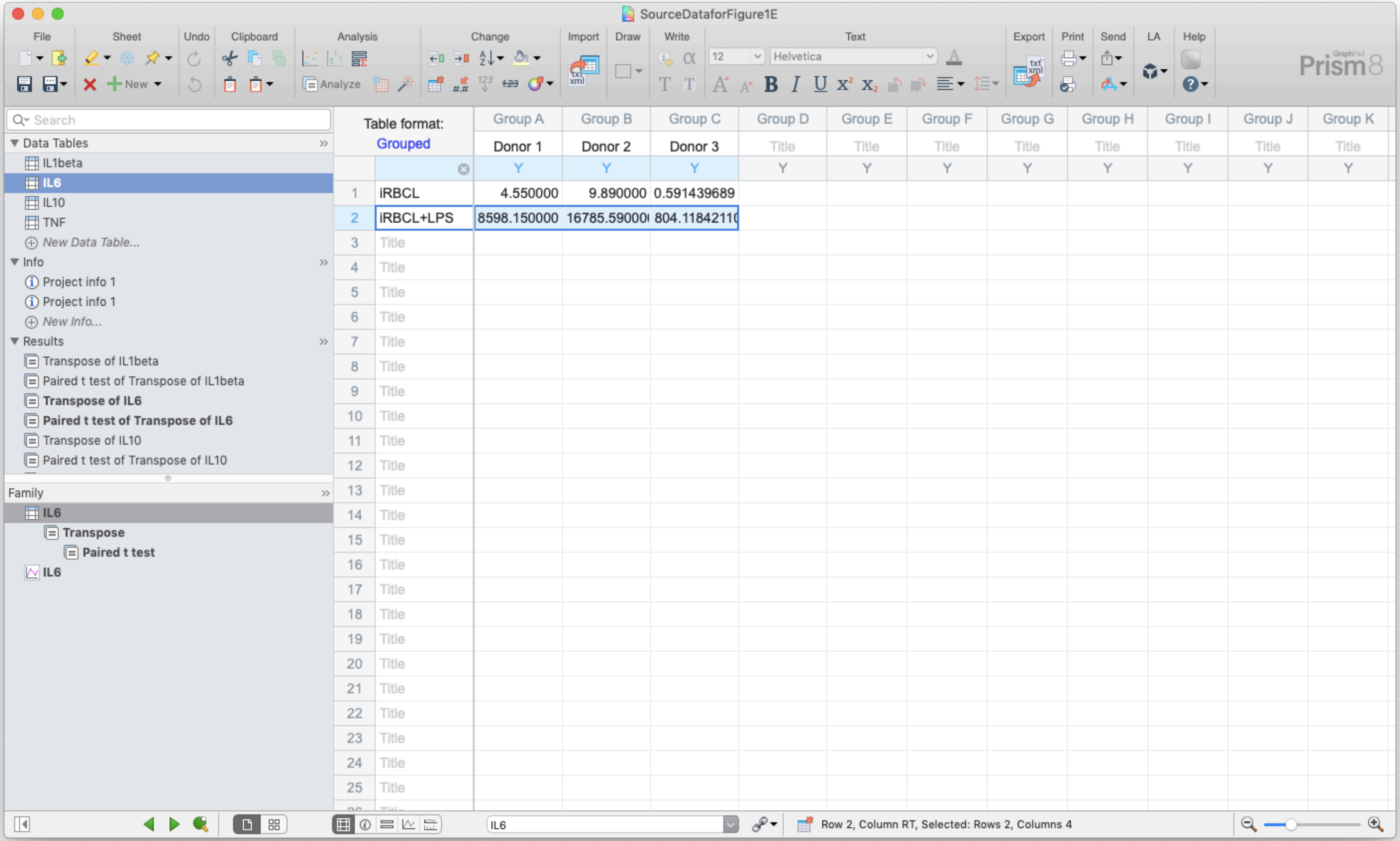

SourceDataforFigure1E
File
Sheet
Undo
Clipboard
Analysis
Change
Import
Draw
Write
Text
Export
Print
Send
LA
Help
Prism8
Search
Data Tables
IL1beta
IL6
IL10
TNF
New Data Table...
Info
Project info 1
Project info 1
New Info...
Results
Transpose of IL1beta
Paired t test of Transpose of IL1beta
Transpose of IL6
Paired t test of Transpose of IL6
Transpose of IL10
Paired t test of Transpose of IL10
Family
IL6
Transpose
Paired t test
IL6
Table format:
Grouped
Group A
Group B
Group C
Donor 1
Donor 2
Donor 3
1 iRBCL 4.550000 9.890000 0.591439689
2 iRBCL+LPS 8598.150000 16785.59000 804.1184211
Row 2, Column RT, Selected: Rows 2, Columns 4

SourceDataforFigure1E

Data Tables
- IL1beta
- IL6
- IL10
- TNF
- New Data Table...

Info
- Project info 1
- Project info 1
- New Info...

Results
- Transpose of IL1beta
- Paired t test of Transpose of IL1beta
- Transpose of IL6
- Paired t test of Transpose of IL6
- Transpose of IL10
- Paired t test of Transpose of IL10

Family
- IL10
  - Transpose
    - Paired t test
- IL10

Table format: Grouped

		Group A	Group B	Group C
		Donor 1	Donor 2	Donor 3
		Y	Y	Y
1	iRBCL	3.170000	2.140000	2.18000000
2	iRBCL+LPS	72.060000	53.180000	2.14678899

Row 2, Column RT, Selected: Rows 1, Columns 4

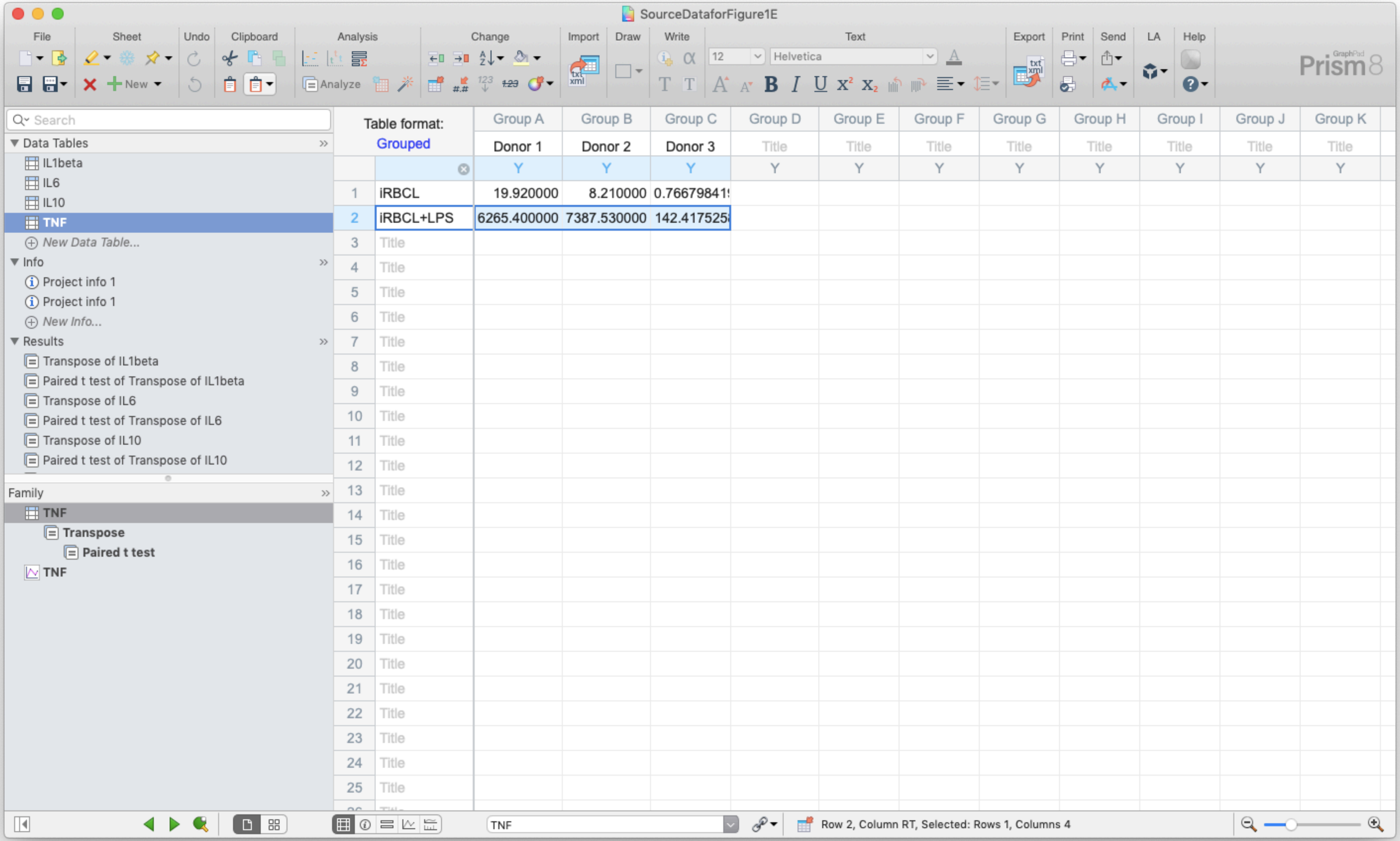
SourceDataforFigure1E
File
Sheet
Undo
Clipboard
Analysis
Change
Import
Draw
Write
Text
Export
Print
Send
LA
Help
Prism 8
Search
Data Tables
IL1beta
IL6
IL10
TNF
New Data Table...
Info
Project info 1
Project info 1
New Info...
Results
Transpose of IL1beta
Paired t test of Transpose of IL1beta
Transpose of IL6
Paired t test of Transpose of IL6
Transpose of IL10
Paired t test of Transpose of IL10
Family
TNF
Transpose
Paired t test
TNF
Table format: Grouped
Group A | Group B | Group C
Donor 1 | Donor 2 | Donor 3
1 iRBCL | 19.920000 | 8.210000 | 0.76679841
2 iRBCL+LPS | 6265.400000 | 7387.530000 | 142.417525
Row 2, Column RT, Selected: Rows 1, Columns 4

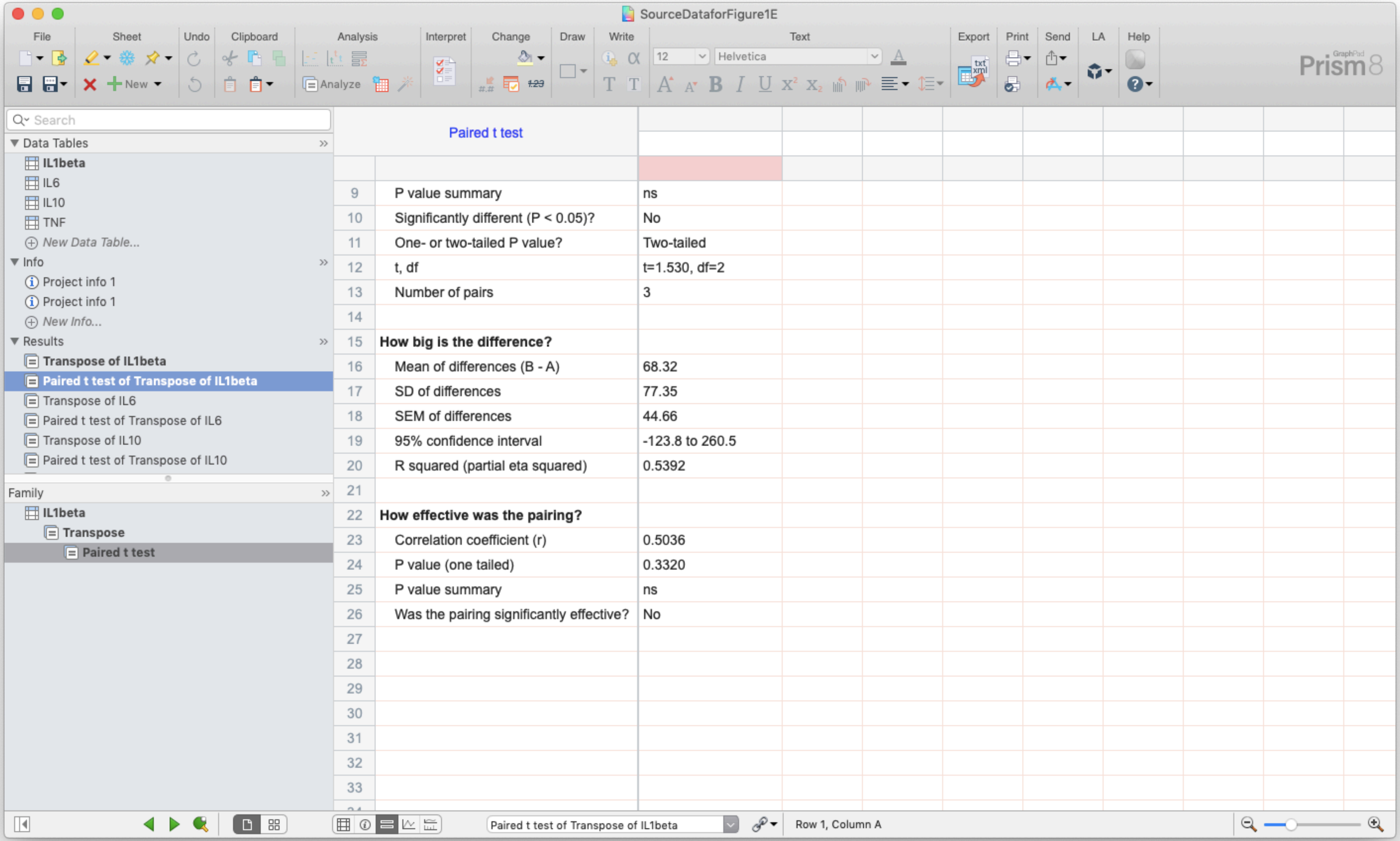

	Paired t test	
9	P value summary	ns
10	Significantly different (P < 0.05)?	No
11	One- or two-tailed P value?	Two-tailed
12	t, df	t=1.530, df=2
13	Number of pairs	3
14		
15	**How big is the difference?**	
16	Mean of differences (B - A)	68.32
17	SD of differences	77.35
18	SEM of differences	44.66
19	95% confidence interval	-123.8 to 260.5
20	R squared (partial eta squared)	0.5392
21		
22	**How effective was the pairing?**	
23	Correlation coefficient (r)	0.5036
24	P value (one tailed)	0.3320
25	P value summary	ns
26	Was the pairing significantly effective?	No
27		
28		
29		
30		
31		
32		
33		

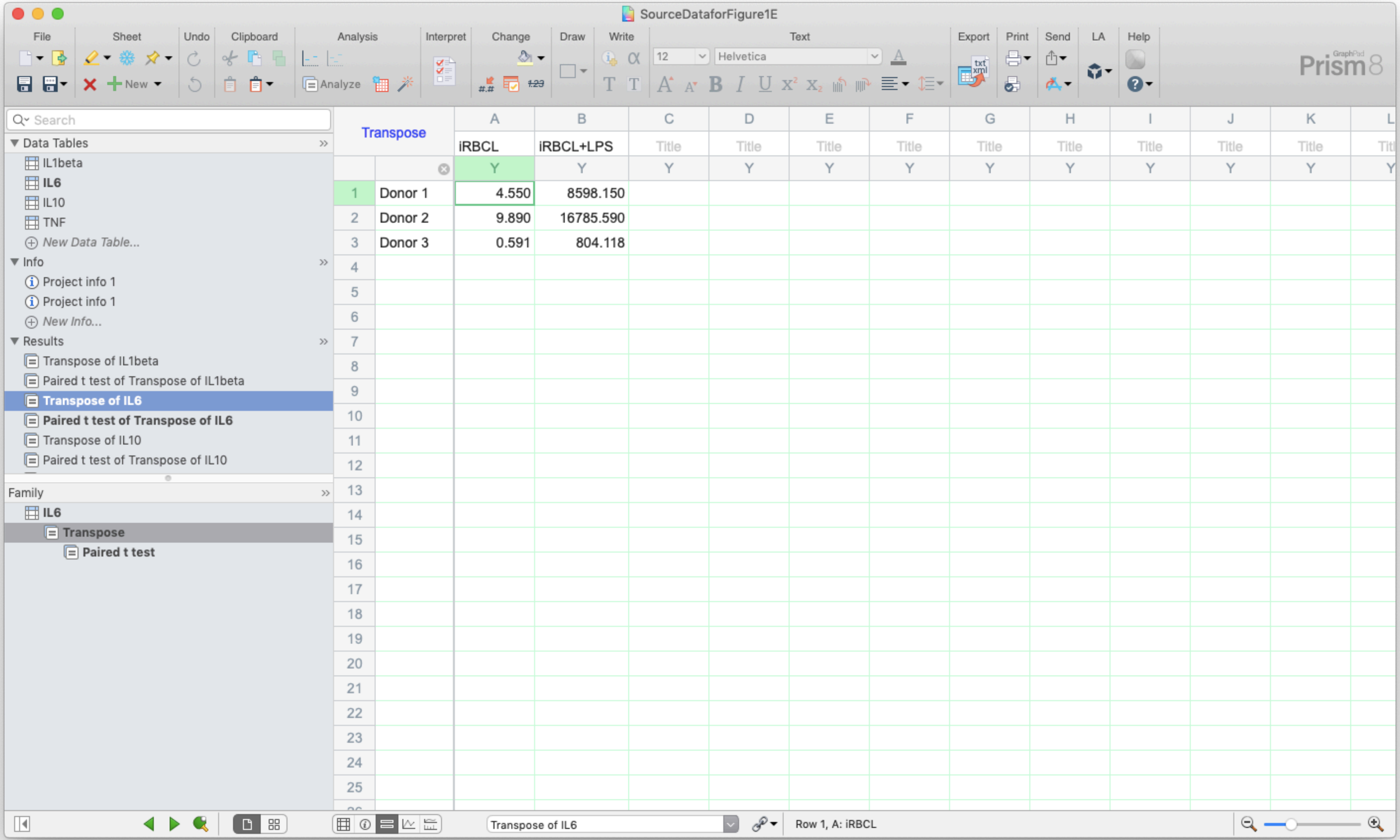
SourceDataforFigure1E
File
Sheet
Undo
Clipboard
Analysis
Interpret
Change
Draw
Write
Text
Export
Print
Send
LA
Help
New
Analyze
12
Helvetica
Prism 8
Search
Data Tables
IL1beta
IL6
IL10
TNF
New Data Table...
Info
Project info 1
Project info 1
New Info...
Results
Transpose of IL1beta
Paired t test of Transpose of IL1beta
Transpose of IL6
Paired t test of Transpose of IL6
Transpose of IL10
Paired t test of Transpose of IL10
Family
IL6
Transpose
Paired t test
Transpose
iRBCL
iRBCL+LPS
Donor 1
4.550
8598.150
Donor 2
9.890
16785.590
Donor 3
0.591
804.118
Transpose of IL6
Row 1, A: iRBCL

SourceDataforFigure1E

Paired t test

9	P value summary	ns
10	Significantly different (P < 0.05)?	No
11	One- or two-tailed P value?	Two-tailed
12	t, df	t=1.892, df=2
13	Number of pairs	3
14		
15	**How big is the difference?**	
16	Mean of differences (B - A)	8724
17	SD of differences	7987
18	SEM of differences	4611
19	95% confidence interval	-11116 to 28565
20	R squared (partial eta squared)	0.6415
21		
22	**How effective was the pairing?**	
23	Correlation coefficient (r)	0.9975
24	P value (one tailed)	0.0227
25	P value summary	*
26	Was the pairing significantly effective?	Yes

Data Tables: IL1beta, IL6, IL10, TNF

Results: Transpose of IL1beta; Paired t test of Transpose of IL1beta; Transpose of IL6; Paired t test of Transpose of IL6; Transpose of IL10; Paired t test of Transpose of IL10

Family: IL6 > Transpose > Paired t test

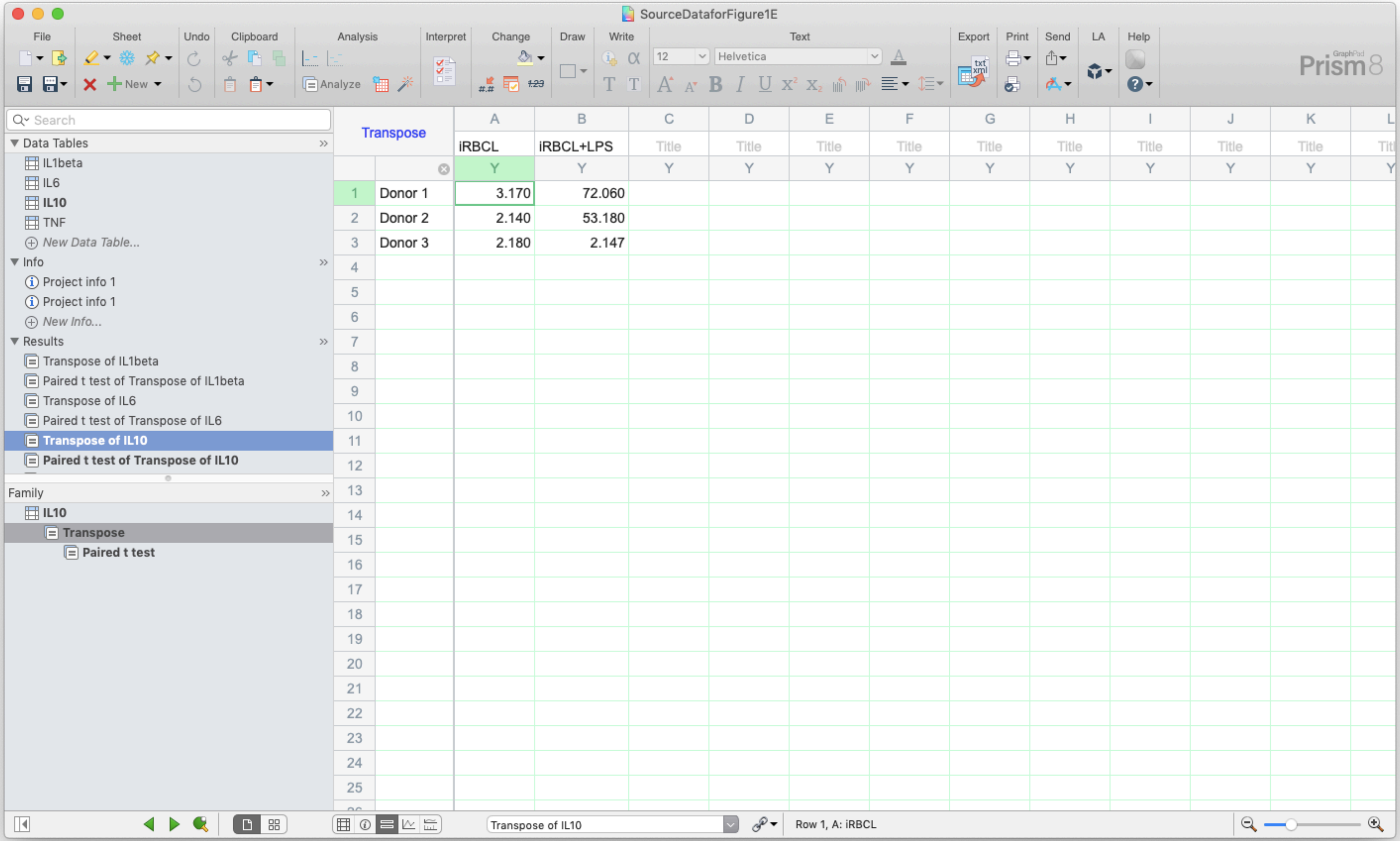
SourceDataforFigure1E
File
Sheet
Undo
Clipboard
Analysis
Interpret
Change
Draw
Write
Text
Export
Print
Send
LA
Help
New
Analyze
12
Helvetica
Prism 8
Search
Data Tables
IL1beta
IL6
IL10
TNF
New Data Table...
Info
Project info 1
Project info 1
New Info...
Results
Transpose of IL1beta
Paired t test of Transpose of IL1beta
Transpose of IL6
Paired t test of Transpose of IL6
Transpose of IL10
Paired t test of Transpose of IL10
Family
IL10
Transpose
Paired t test
Transpose
iRBCL
iRBCL+LPS
Donor 1 3.170 72.060
Donor 2 2.140 53.180
Donor 3 2.180 2.147
Transpose of IL10
Row 1, A: iRBCL

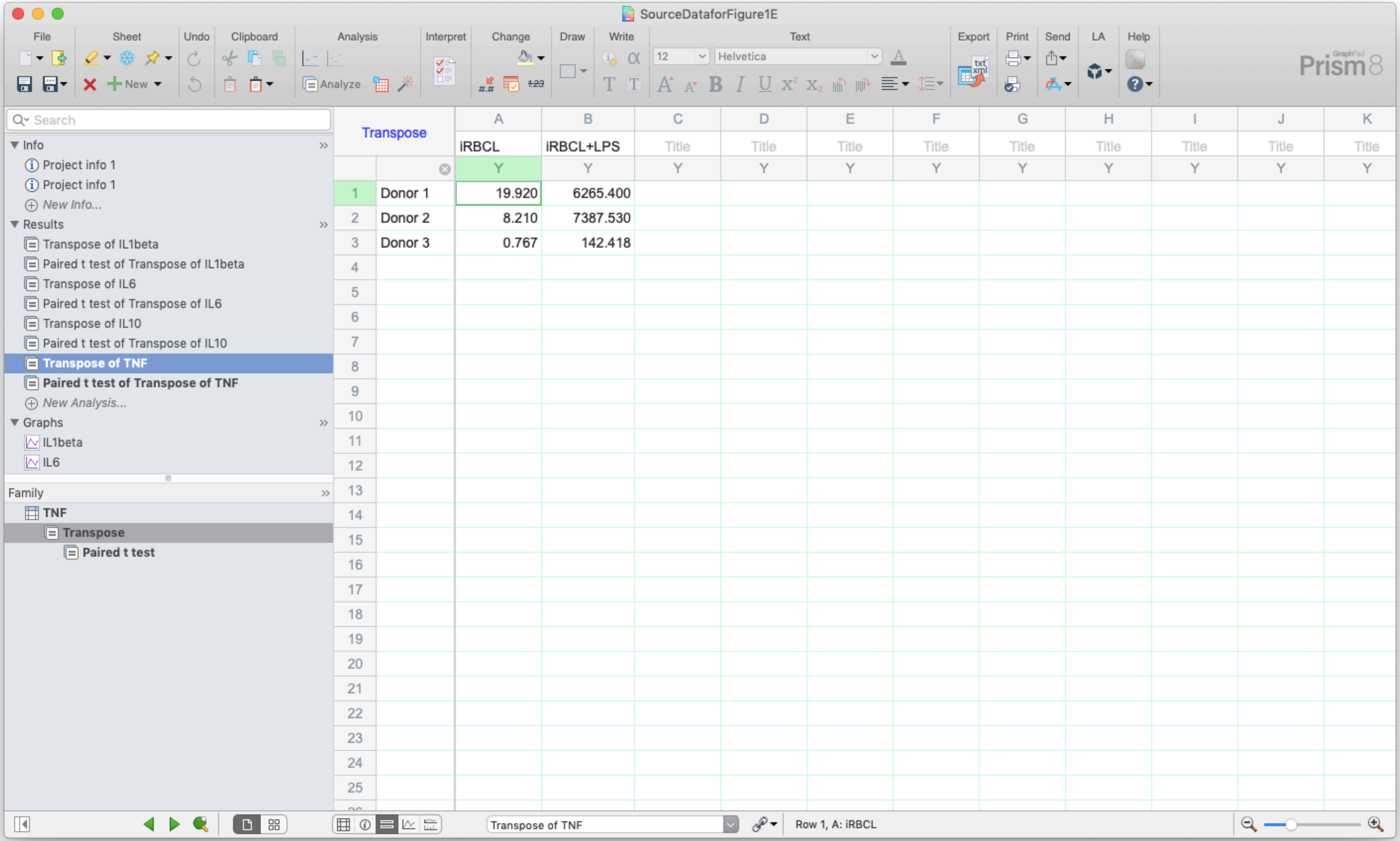
SourceDataforFigure1E
Info
Project info 1
Project info 1
New Info...
Results
Transpose of IL1beta
Paired t test of Transpose of IL1beta
Transpose of IL6
Paired t test of Transpose of IL6
Transpose of IL10
Paired t test of Transpose of IL10
Transpose of TNF
Paired t test of Transpose of TNF
New Analysis...
Graphs
IL1beta
IL6
Family
TNF
Transpose
Paired t test
Transpose
iRBCL
iRBCL+LPS
Donor 1 19.920 6265.400
Donor 2 8.210 7387.530
Donor 3 0.767 142.418
Transpose of TNF
Row 1, A: iRBCL

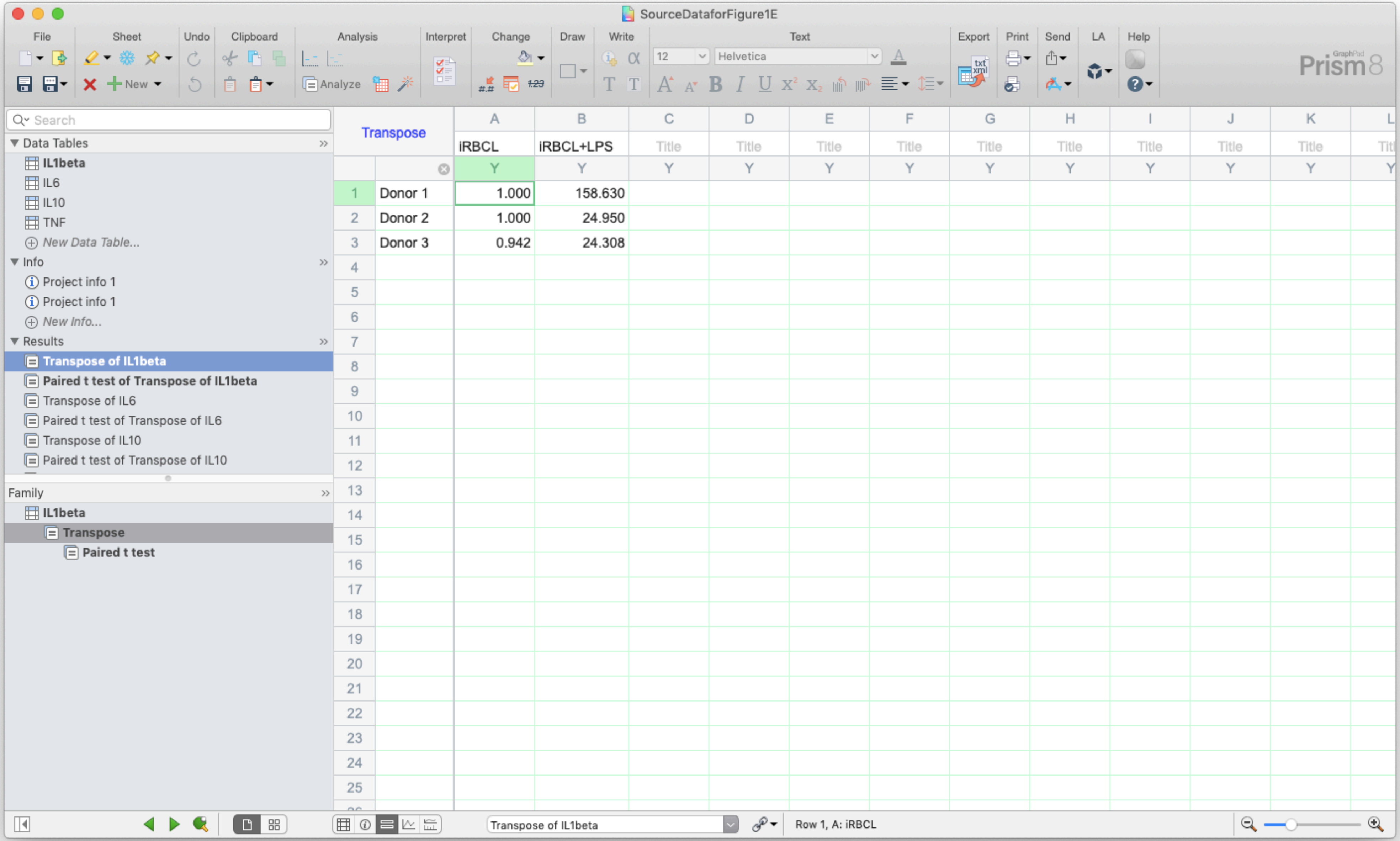
SourceDataforFigure1E
Data Tables
IL1beta
IL6
IL10
TNF
New Data Table...
Info
Project info 1
Project info 1
New Info...
Results
Transpose of IL1beta
Paired t test of Transpose of IL1beta
Transpose of IL6
Paired t test of Transpose of IL6
Transpose of IL10
Paired t test of Transpose of IL10
Family
IL1beta
Transpose
Paired t test
Transpose
iRBCL
iRBCL+LPS
Donor 1 1.000 158.630
Donor 2 1.000 24.950
Donor 3 0.942 24.308
Transpose of IL1beta
Row 1, A: iRBCL

SourceDataforFigure1E

Info
- Project info 1
- Project info 1
- New Info...

Results
- Transpose of IL1beta
- Paired t test of Transpose of IL1beta
- Transpose of IL6
- Paired t test of Transpose of IL6
- Transpose of IL10
- Paired t test of Transpose of IL10
- **Transpose of TNF**
- **Paired t test of Transpose of TNF**
- New Analysis...

Graphs
- IL1beta
- IL6

Family
- **TNF**
  - **Transpose**
    - **Paired t test**

	Paired t test	
1	Table Analyzed	Transpose of TNF
2		
3	Column B	iRBCL+LPS
4	vs.	vs.
5	Column A	iRBCL
6		
7	**Paired t test**	
8	P value	0.1779
9	P value summary	ns
10	Significantly different (P < 0.05)?	No
11	One- or two-tailed P value?	Two-tailed
12	t, df	t=2.042, df=2
13	Number of pairs	3
14		
15	**How big is the difference?**	
16	Mean of differences (B - A)	4589
17	SD of differences	3893
18	SEM of differences	2248
19	95% confidence interval	-5082 to 14259
20	R squared (partial eta squared)	0.6758
21		
22	**How effective was the pairing?**	
23	Correlation coefficient (r)	0.6996
24	P value (one tailed)	0.2533
25	P value summary	ns
26	[illegible]	[illegible]

Paired t test of Transpose of TNF

Row 1, Column A

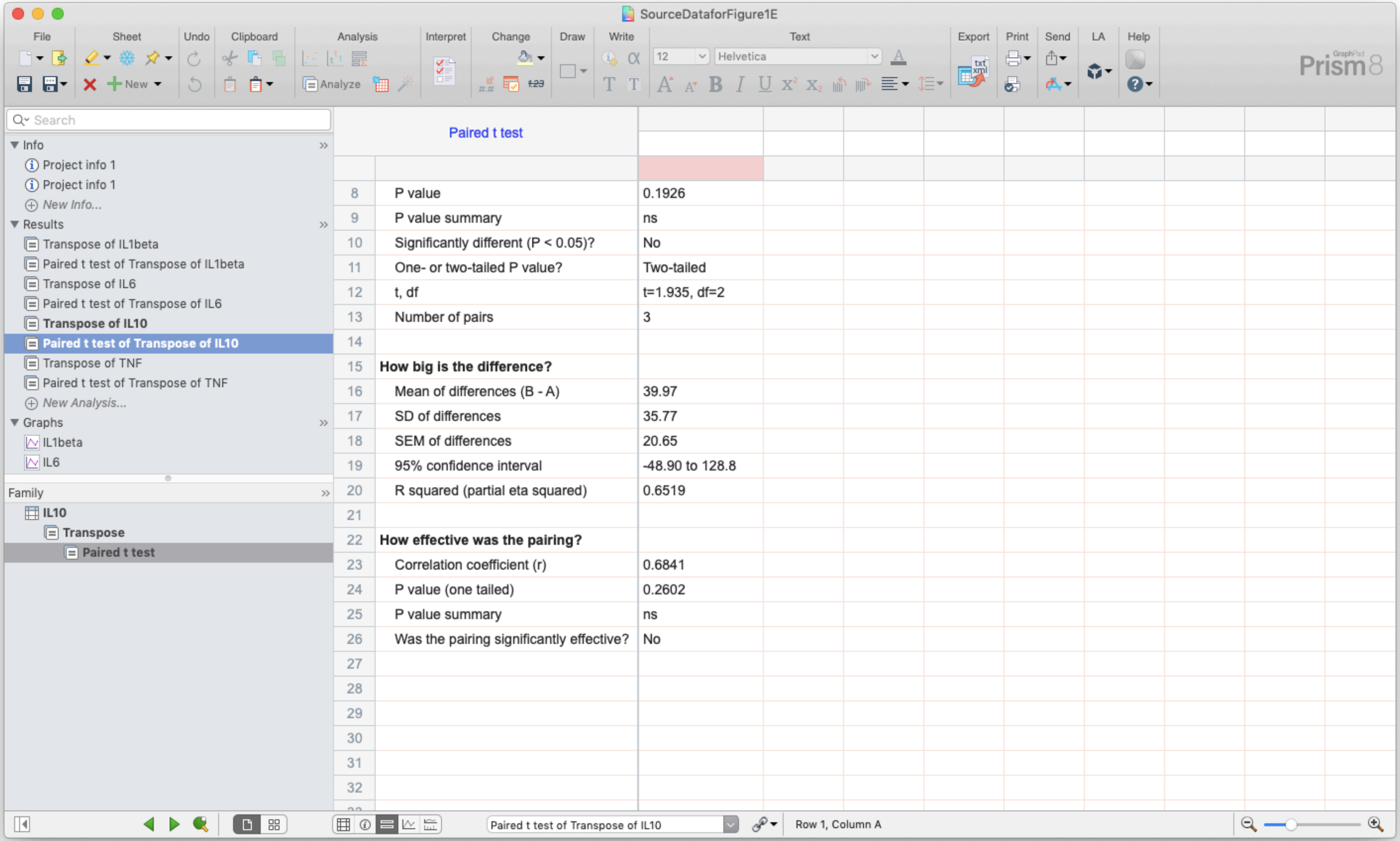
SourceDataforFigure1E
File
Sheet
Undo
Clipboard
Analysis
Interpret
Change
Draw
Write
Text
Export
Print
Send
LA
Help
New
Analyze
Helvetica
Prism 8
Search
Info
Project info 1
Project info 1
New Info...
Results
Transpose of IL1beta
Paired t test of Transpose of IL1beta
Transpose of IL6
Paired t test of Transpose of IL6
Transpose of IL10
Paired t test of Transpose of IL10
Transpose of TNF
Paired t test of Transpose of TNF
New Analysis...
Graphs
IL1beta
IL6
Family
IL10
Transpose
Paired t test
Paired t test
8 P value 0.1926
9 P value summary ns
10 Significantly different (P < 0.05)? No
11 One- or two-tailed P value? Two-tailed
12 t, df t=1.935, df=2
13 Number of pairs 3
14
15 How big is the difference?
16 Mean of differences (B - A) 39.97
17 SD of differences 35.77
18 SEM of differences 20.65
19 95% confidence interval -48.90 to 128.8
20 R squared (partial eta squared) 0.6519
21
22 How effective was the pairing?
23 Correlation coefficient (r) 0.6841
24 P value (one tailed) 0.2602
25 P value summary ns
26 Was the pairing significantly effective? No
Paired t test of Transpose of IL10
Row 1, Column A
