# Supplementary material for: Malaria inflammation by xanthine oxidase‐produced reactive oxygen species
Source: EMBO Mol Med. 2019 Jul 2;11(8):e9903. doi: 10.15252/emmm.201809903 (PMC6685105; doi:10.15252/emmm.201809903)
Supplement: Supplementary file 5 — Source Data for Figure 2 [file EMMM-11-e9903-s003.pdf]

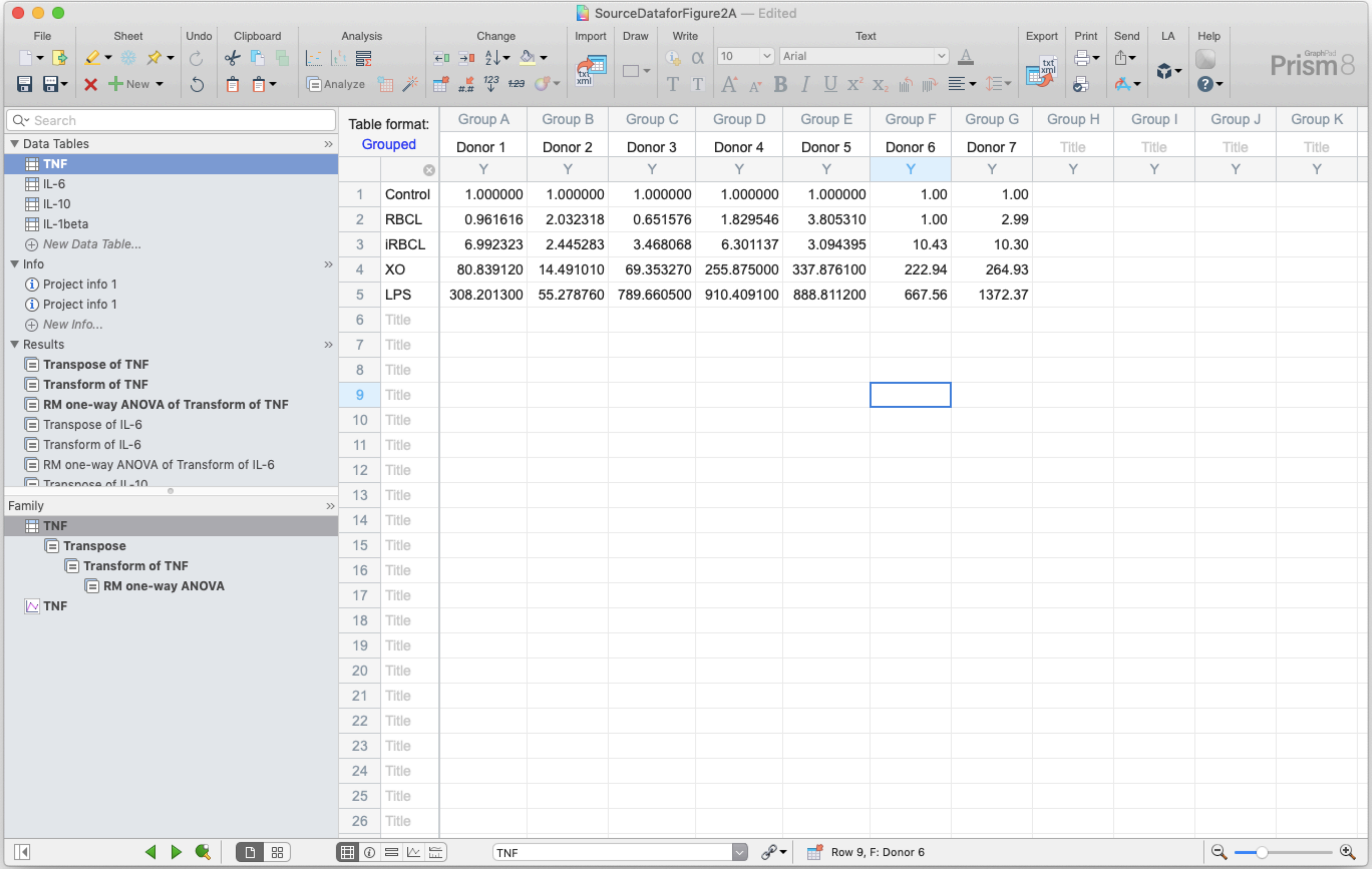

	Group A	Group B	Group C	Group D	Group E	Group F	Group G
	Donor 1	Donor 2	Donor 3	Donor 4	Donor 5	Donor 6	Donor 7
	Y	Y	Y	Y	Y	Y	Y
1 Control	1.000000	1.000000	1.000000	1.000000	1.000000	1.00	1.00
2 RBCL	0.961616	2.032318	0.651576	1.829546	3.805310	1.00	2.99
3 iRBCL	6.992323	2.445283	3.468068	6.301137	3.094395	10.43	10.30
4 XO	80.839120	14.491010	69.353270	255.875000	337.876100	222.94	264.93
5 LPS	308.201300	55.278760	789.660500	910.409100	888.811200	667.56	1372.37

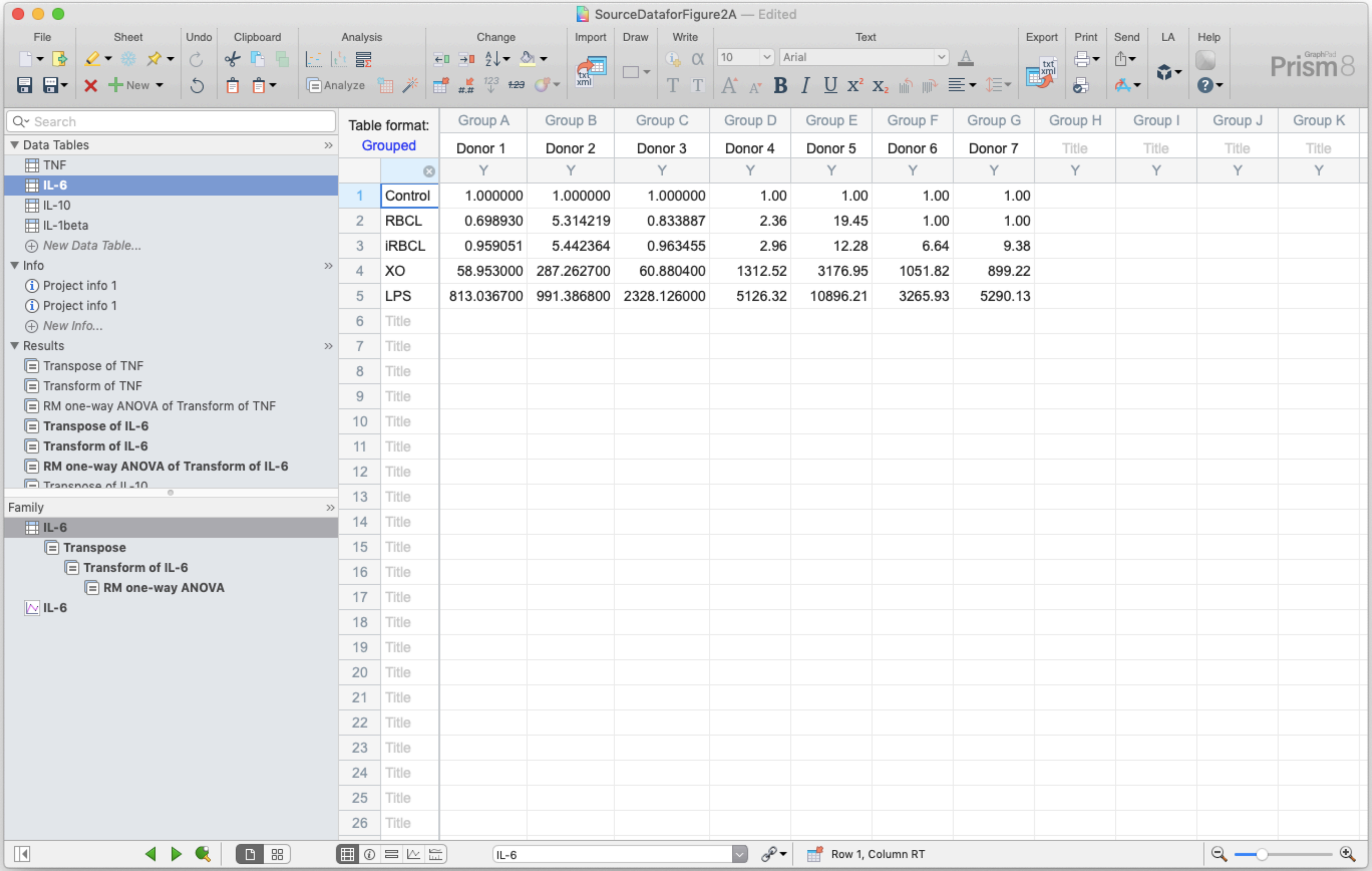

Table format: Grouped	Group A	Group B	Group C	Group D	Group E	Group F	Group G
	Donor 1	Donor 2	Donor 3	Donor 4	Donor 5	Donor 6	Donor 7
1 Control	1.000000	1.000000	1.000000	1.00	1.00	1.00	1.00
2 RBCL	0.698930	5.314219	0.833887	2.36	19.45	1.00	1.00
3 iRBCL	0.959051	5.442364	0.963455	2.96	12.28	6.64	9.38
4 XO	58.953000	287.262700	60.880400	1312.52	3176.95	1051.82	899.22
5 LPS	813.036700	991.386800	2328.126000	5126.32	10896.21	3265.93	5290.13

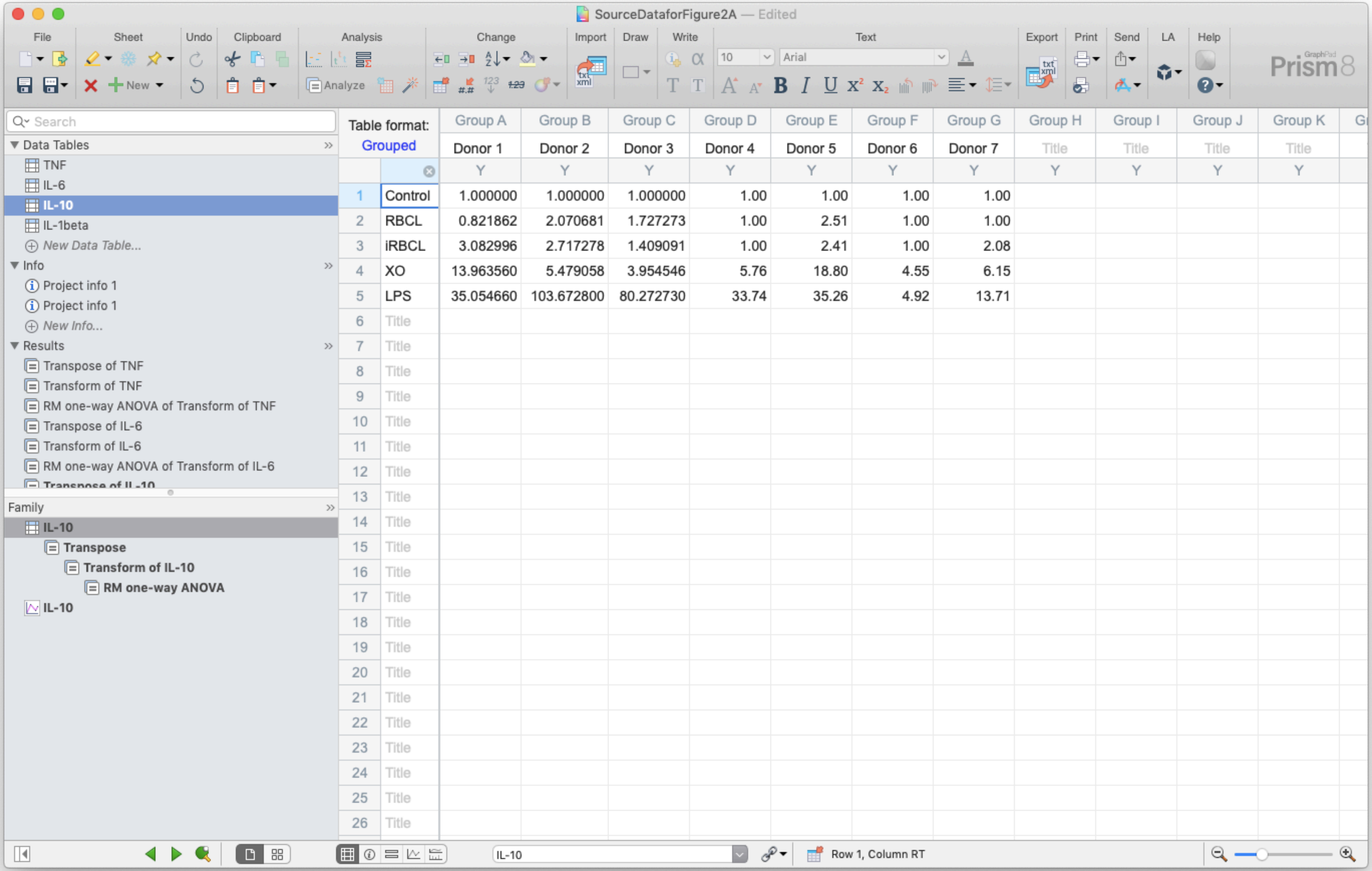

	Group A	Group B	Group C	Group D	Group E	Group F	Group G
	Donor 1	Donor 2	Donor 3	Donor 4	Donor 5	Donor 6	Donor 7
	Y	Y	Y	Y	Y	Y	Y
1 Control	1.000000	1.000000	1.000000	1.00	1.00	1.00	1.00
2 RBCL	0.821862	2.070681	1.727273	1.00	2.51	1.00	1.00
3 iRBCL	3.082996	2.717278	1.409091	1.00	2.41	1.00	2.08
4 XO	13.963560	5.479058	3.954546	5.76	18.80	4.55	6.15
5 LPS	35.054660	103.672800	80.272730	33.74	35.26	4.92	13.71

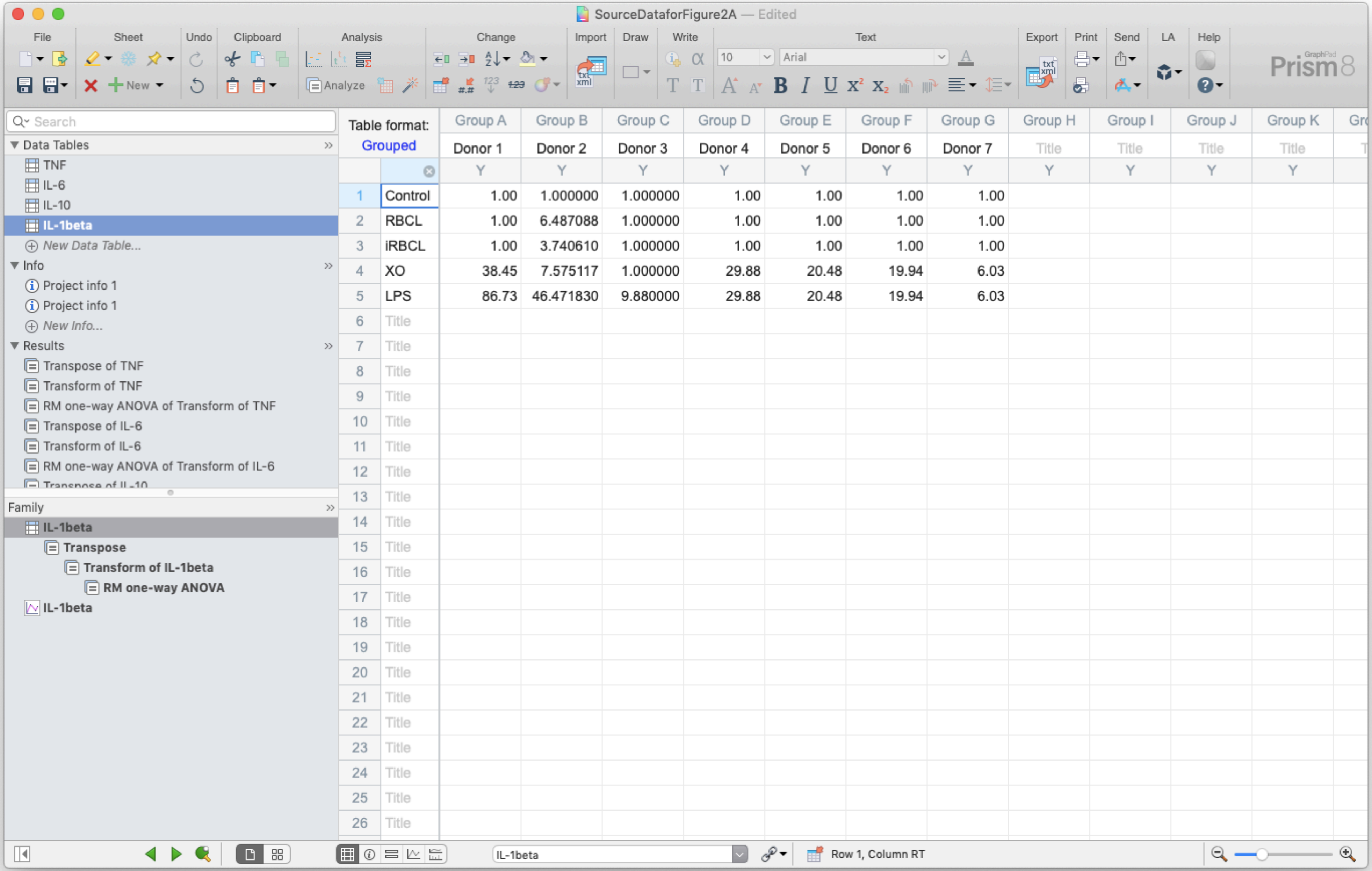

		Group A	Group B	Group C	Group D	Group E	Group F	Group G
		Donor 1	Donor 2	Donor 3	Donor 4	Donor 5	Donor 6	Donor 7
		Y	Y	Y	Y	Y	Y	Y
1	Control	1.00	1.000000	1.000000	1.00	1.00	1.00	1.00
2	RBCL	1.00	6.487088	1.000000	1.00	1.00	1.00	1.00
3	iRBCL	1.00	3.740610	1.000000	1.00	1.00	1.00	1.00
4	XO	38.45	7.575117	1.000000	29.88	20.48	19.94	6.03
5	LPS	86.73	46.471830	9.880000	29.88	20.48	19.94	6.03

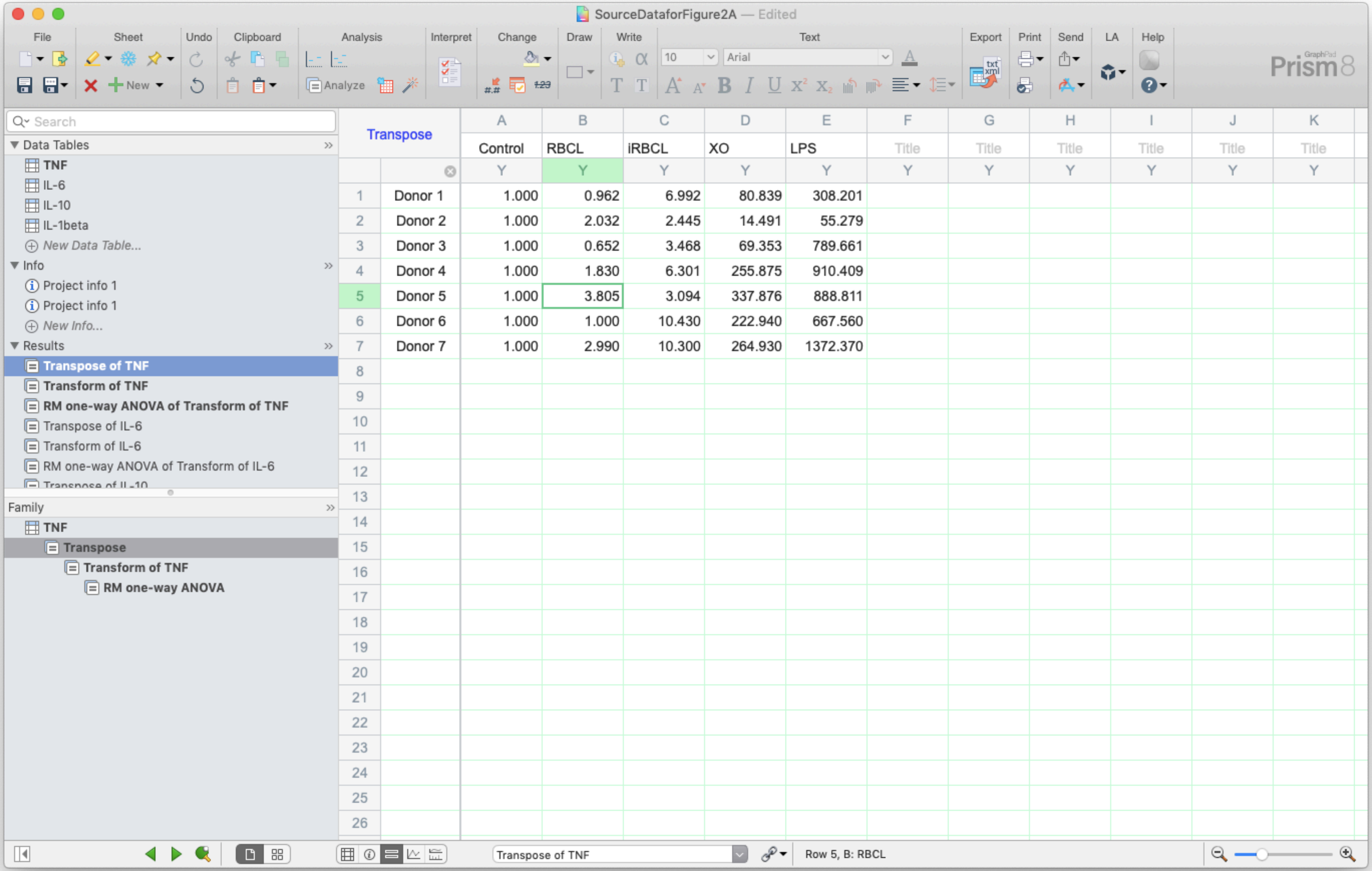

Transpose	Control	RBCL	iRBCL	XO	LPS
	Y	Y	Y	Y	Y
Donor 1	1.000	0.962	6.992	80.839	308.201
Donor 2	1.000	2.032	2.445	14.491	55.279
Donor 3	1.000	0.652	3.468	69.353	789.661
Donor 4	1.000	1.830	6.301	255.875	910.409
Donor 5	1.000	3.805	3.094	337.876	888.811
Donor 6	1.000	1.000	10.430	222.940	667.560
Donor 7	1.000	2.990	10.300	264.930	1372.370

SourceDataforFigure2A — Edited

Data Tables
- TNF
- IL-6
- IL-10
- IL-1beta
- New Data Table...

Info
- Project info 1
- Project info 1
- New Info...

Results
- Transpose of TNF
- Transform of TNF
- RM one-way ANOVA of Transform of TNF
- Transpose of IL-6
- Transform of IL-6
- RM one-way ANOVA of Transform of IL-6
- Transpose of IL-10

Family
- TNF
  - Transpose
    - Transform of TNF
      - RM one-way ANOVA

Transform	Control	RBCL	iRBCL	XO	LPS
	Y	Y	Y	Y	Y
Donor 1	0.000	-0.017	0.845	1.908	2.489
Donor 2	0.000	0.308	0.388	1.161	1.743
Donor 3	0.000	-0.186	0.540	1.841	2.897
Donor 4	0.000	0.262	0.799	2.408	2.959
Donor 5	0.000	0.580	0.491	2.529	2.949
Donor 6	0.000	0.000	1.018	2.348	2.824
Donor 7	0.000	0.476	1.013	2.423	3.137

Transform: Y=Log(Y)

Transform of TNF

Row 1, A: Control

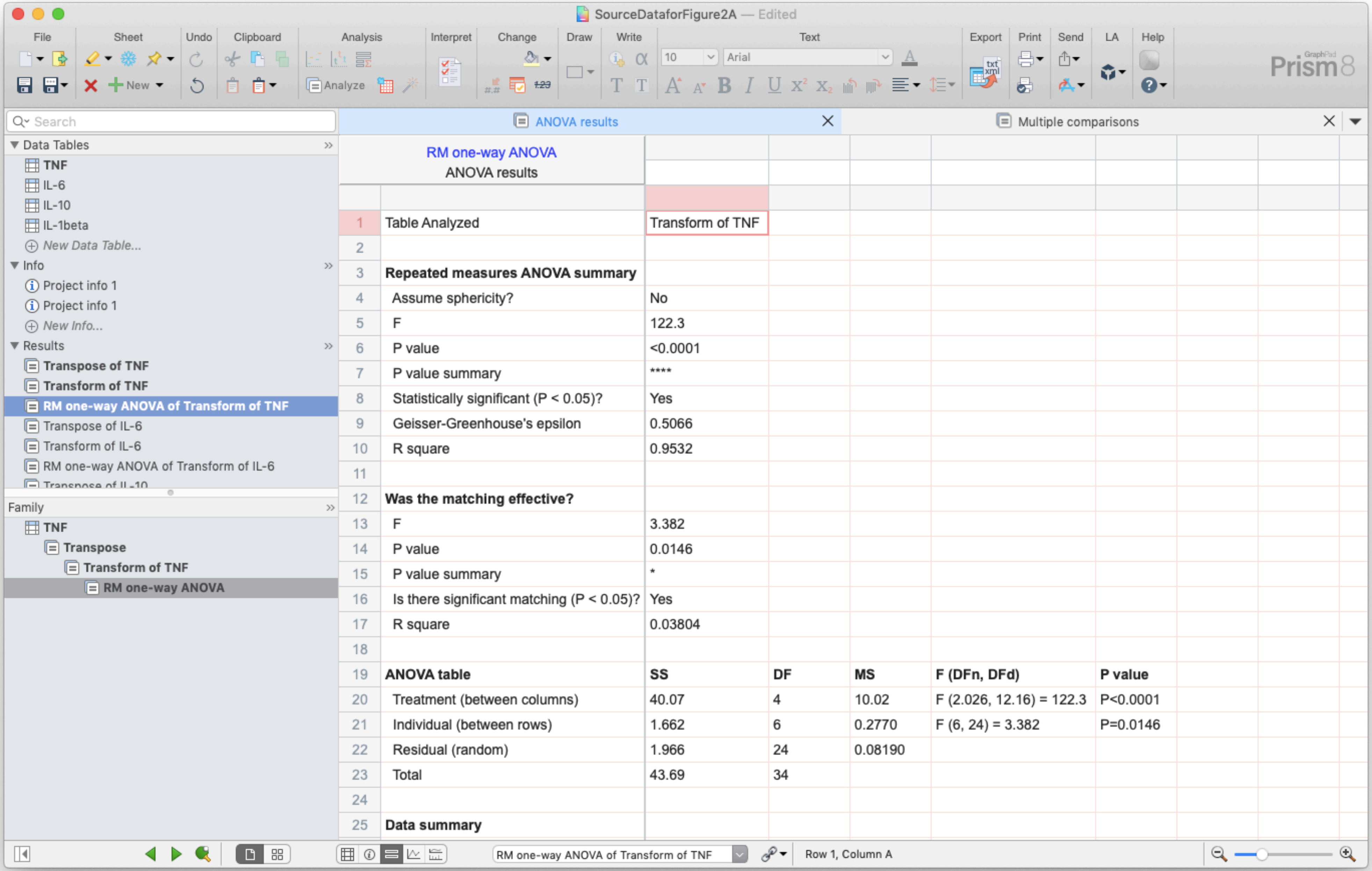

RM one-way ANOVA
ANOVA results

1	Table Analyzed	Transform of TNF				
2						
3	**Repeated measures ANOVA summary**					
4	Assume sphericity?	No				
5	F	122.3				
6	P value	<0.0001				
7	P value summary	****				
8	Statistically significant (P < 0.05)?	Yes				
9	Geisser-Greenhouse's epsilon	0.5066				
10	R square	0.9532				
11						
12	**Was the matching effective?**					
13	F	3.382				
14	P value	0.0146				
15	P value summary	*				
16	Is there significant matching (P < 0.05)?	Yes				
17	R square	0.03804				
18						
19	**ANOVA table**	**SS**	**DF**	**MS**	**F (DFn, DFd)**	**P value**
20	Treatment (between columns)	40.07	4	10.02	F (2.026, 12.16) = 122.3	P<0.0001
21	Individual (between rows)	1.662	6	0.2770	F (6, 24) = 3.382	P=0.0146
22	Residual (random)	1.966	24	0.08190		
23	Total	43.69	34			
24						
25	**Data summary**					

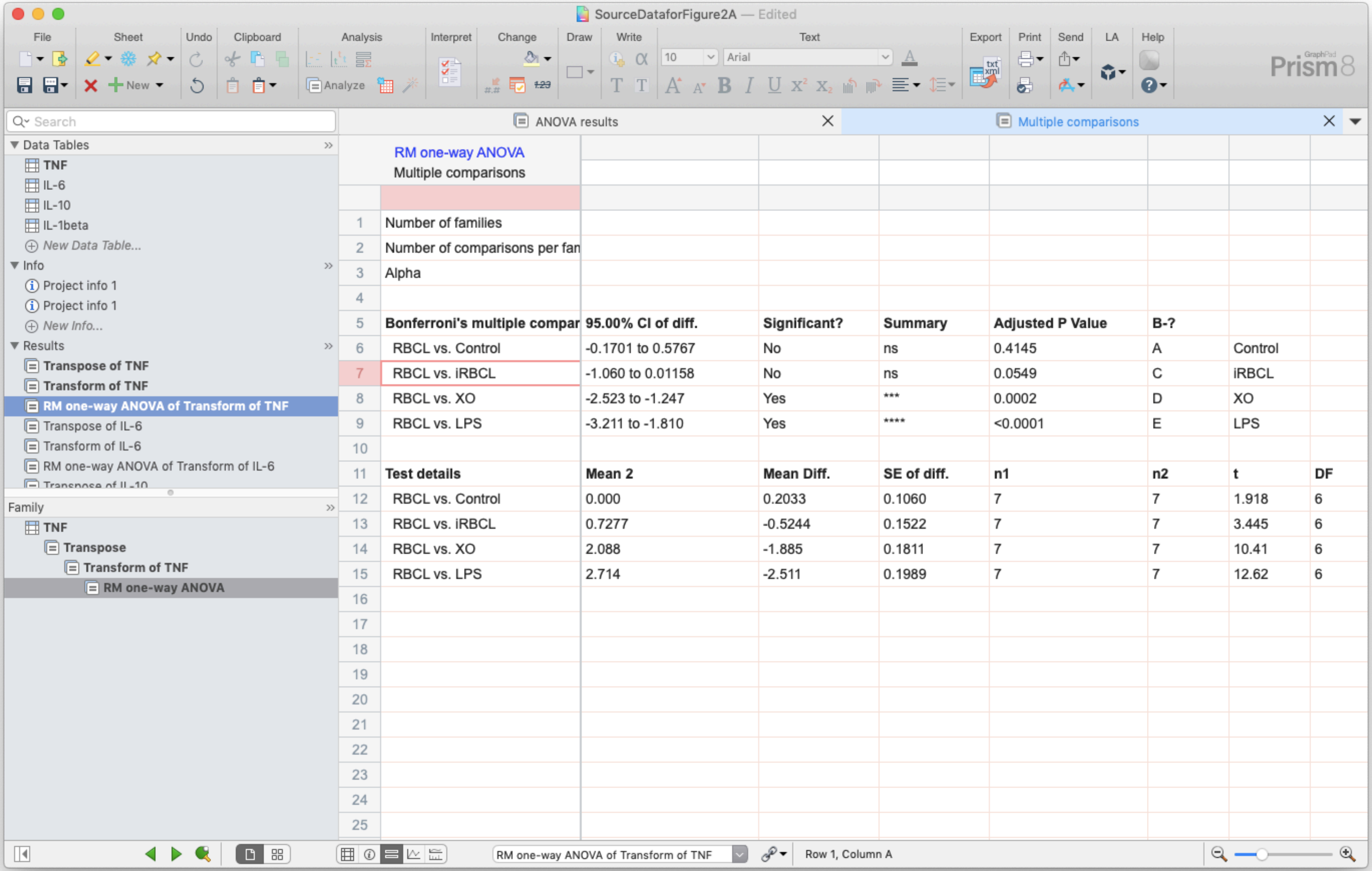

	RM one-way ANOVA<br>Multiple comparisons							
1	Number of families							
2	Number of comparisons per fan							
3	Alpha							
4								
5	**Bonferroni's multiple compar**	**95.00% CI of diff.**	**Significant?**	**Summary**	**Adjusted P Value**	**B-?**		
6	RBCL vs. Control	-0.1701 to 0.5767	No	ns	0.4145	A	Control	
7	RBCL vs. iRBCL	-1.060 to 0.01158	No	ns	0.0549	C	iRBCL	
8	RBCL vs. XO	-2.523 to -1.247	Yes	***	0.0002	D	XO	
9	RBCL vs. LPS	-3.211 to -1.810	Yes	****	<0.0001	E	LPS	
10								
11	**Test details**	**Mean 2**	**Mean Diff.**	**SE of diff.**	**n1**	**n2**	**t**	**DF**
12	RBCL vs. Control	0.000	0.2033	0.1060	7	7	1.918	6
13	RBCL vs. iRBCL	0.7277	-0.5244	0.1522	7	7	3.445	6
14	RBCL vs. XO	2.088	-1.885	0.1811	7	7	10.41	6
15	RBCL vs. LPS	2.714	-2.511	0.1989	7	7	12.62	6

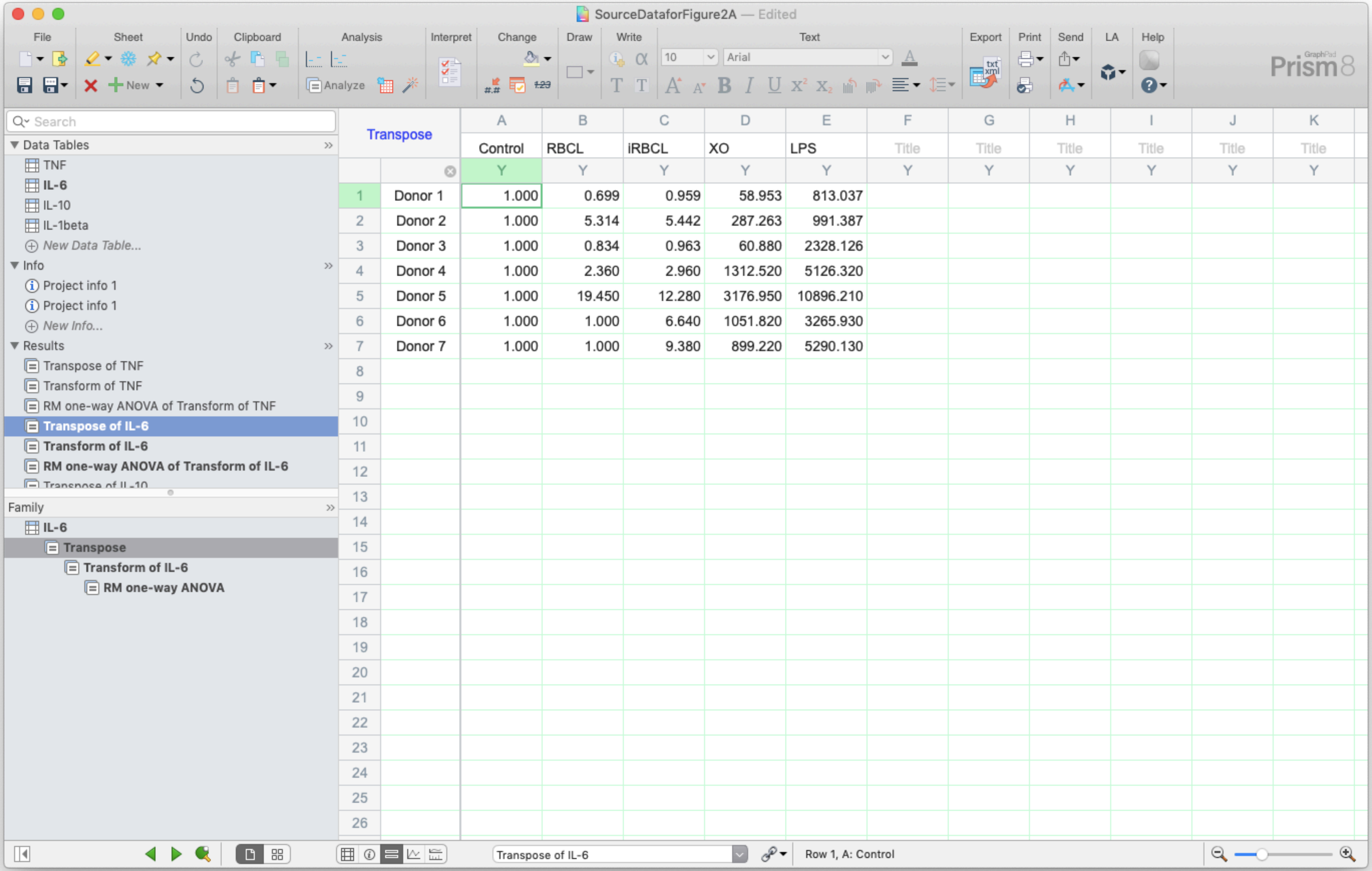

Transpose	Control	RBCL	iRBCL	XO	LPS
	Y	Y	Y	Y	Y
Donor 1	1.000	0.699	0.959	58.953	813.037
Donor 2	1.000	5.314	5.442	287.263	991.387
Donor 3	1.000	0.834	0.963	60.880	2328.126
Donor 4	1.000	2.360	2.960	1312.520	5126.320
Donor 5	1.000	19.450	12.280	3176.950	10896.210
Donor 6	1.000	1.000	6.640	1051.820	3265.930
Donor 7	1.000	1.000	9.380	899.220	5290.130

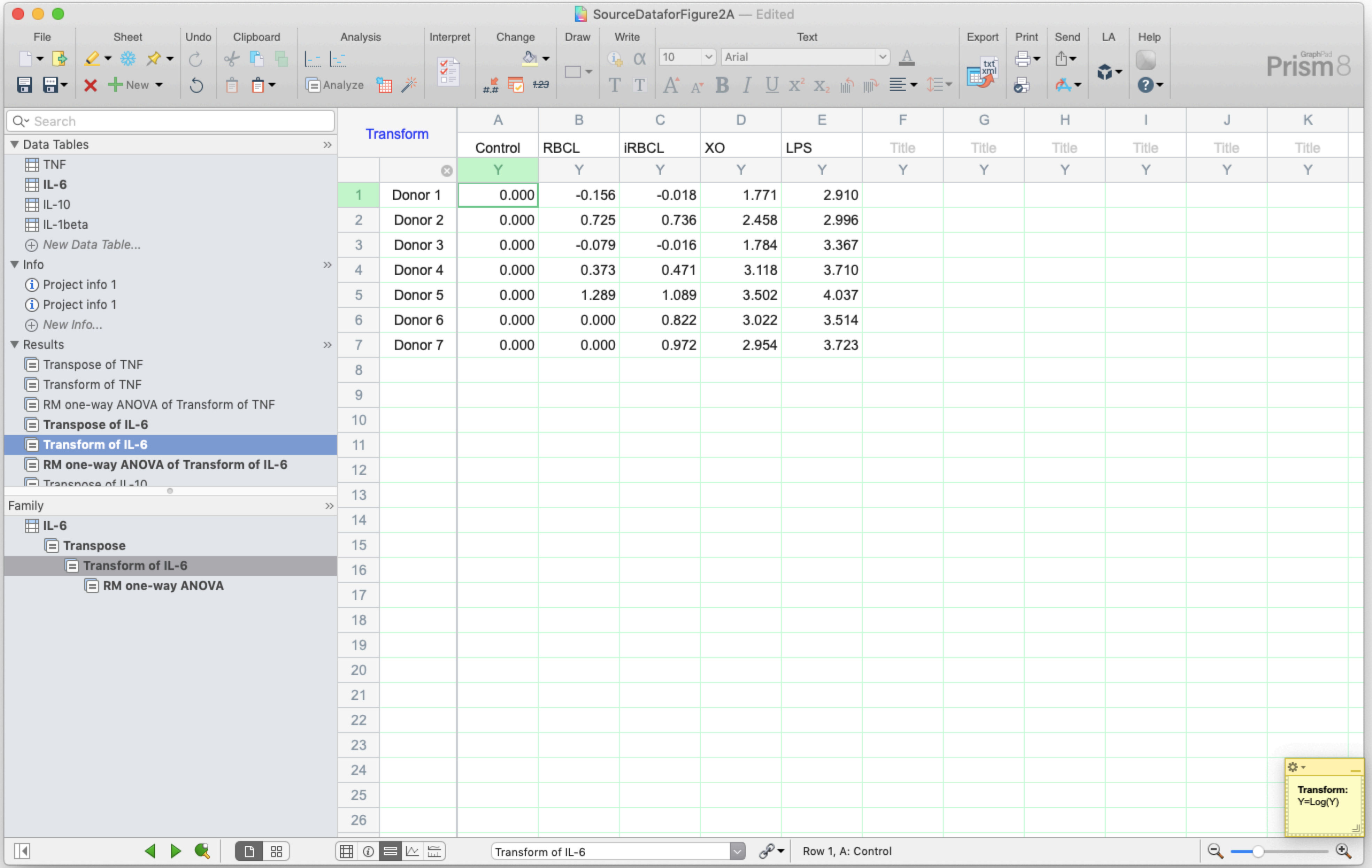

Transform	Control	RBCL	iRBCL	XO	LPS
Donor 1	0.000	-0.156	-0.018	1.771	2.910
Donor 2	0.000	0.725	0.736	2.458	2.996
Donor 3	0.000	-0.079	-0.016	1.784	3.367
Donor 4	0.000	0.373	0.471	3.118	3.710
Donor 5	0.000	1.289	1.089	3.502	4.037
Donor 6	0.000	0.000	0.822	3.022	3.514
Donor 7	0.000	0.000	0.972	2.954	3.723

SourceDataforFigure2A — Edited

Results:
- Transpose of TNF
- Transform of TNF
- RM one-way ANOVA of Transform of TNF
- **Transpose of IL-6**
- **Transform of IL-6**
- **RM one-way ANOVA of Transform of IL-6**
- Transpose of IL-10
- Transform of IL-10
- RM one-way ANOVA of Transform of IL-10
- Transpose of IL-1beta
- Transform of IL-1beta
- RM one-way ANOVA of Transform of IL-1beta

Family:
- **IL-6**
  - **Transpose**
    - **Transform of IL-6**
      - **RM one-way ANOVA**

ANOVA results | Multiple comparisons

RM one-way ANOVA

ANOVA results

1	Table Analyzed	Transform of IL-6				
2						
3	**Repeated measures ANOVA summary**					
4	Assume sphericity?	No				
5	F	148.2				
6	P value	<0.0001				
7	P value summary	****				
8	Statistically significant (P < 0.05)?	Yes				
9	Geisser-Greenhouse's epsilon	0.6553				
10	R square	0.9611				
11						
12	**Was the matching effective?**					
13	F	5.693				
14	P value	0.0009				
15	P value summary	***				
16	Is there significant matching (P < 0.05)?	Yes				
17	R square	0.05247				
18						
19	**ANOVA table**	**SS**	**DF**	**MS**	**F (DFn, DFd)**	**P value**
20	Treatment (between columns)	67.73	4	16.93	F (2.621, 15.73) = 148.2	P<0.0001
21	Individual (between rows)	3.902	6	0.6504	F (6, 24) = 5.693	P=0.0009
22	Residual (random)	2.742	24	0.1143		
23	Total	74.38	34			
24						
25	**Data summary**					

RM one-way ANOVA of Transform of IL-6 — Row 1, Column A

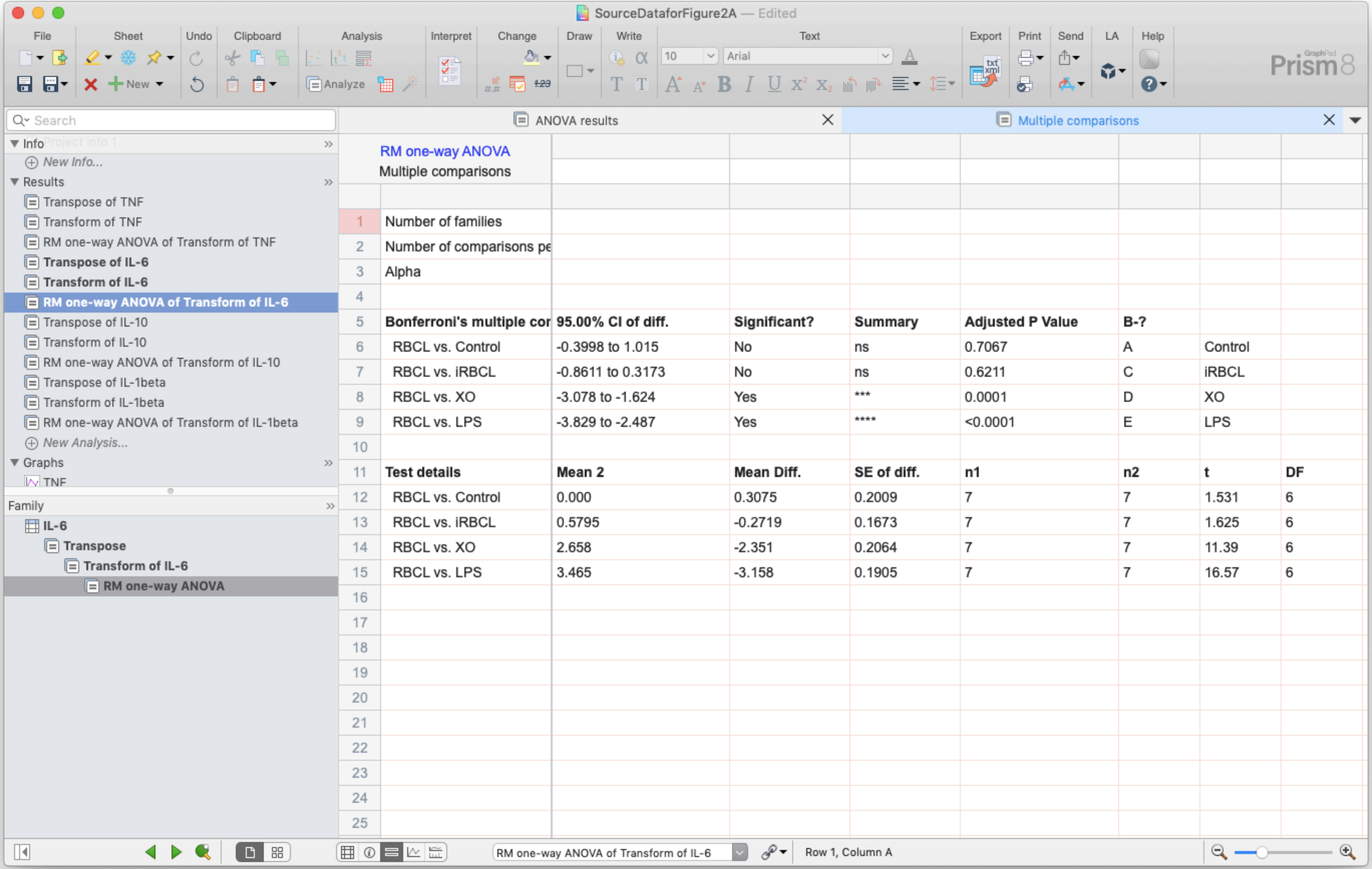

RM one-way ANOVA Multiple comparisons								
1	Number of families							
2	Number of comparisons pe							
3	Alpha							
4								
5	**Bonferroni's multiple cor**	**95.00% CI of diff.**	**Significant?**	**Summary**	**Adjusted P Value**	**B-?**		
6	RBCL vs. Control	-0.3998 to 1.015	No	ns	0.7067	A	Control	
7	RBCL vs. iRBCL	-0.8611 to 0.3173	No	ns	0.6211	C	iRBCL	
8	RBCL vs. XO	-3.078 to -1.624	Yes	***	0.0001	D	XO	
9	RBCL vs. LPS	-3.829 to -2.487	Yes	****	<0.0001	E	LPS	
10								
11	**Test details**	**Mean 2**	**Mean Diff.**	**SE of diff.**	**n1**	**n2**	**t**	**DF**
12	RBCL vs. Control	0.000	0.3075	0.2009	7	7	1.531	6
13	RBCL vs. iRBCL	0.5795	-0.2719	0.1673	7	7	1.625	6
14	RBCL vs. XO	2.658	-2.351	0.2064	7	7	11.39	6
15	RBCL vs. LPS	3.465	-3.158	0.1905	7	7	16.57	6

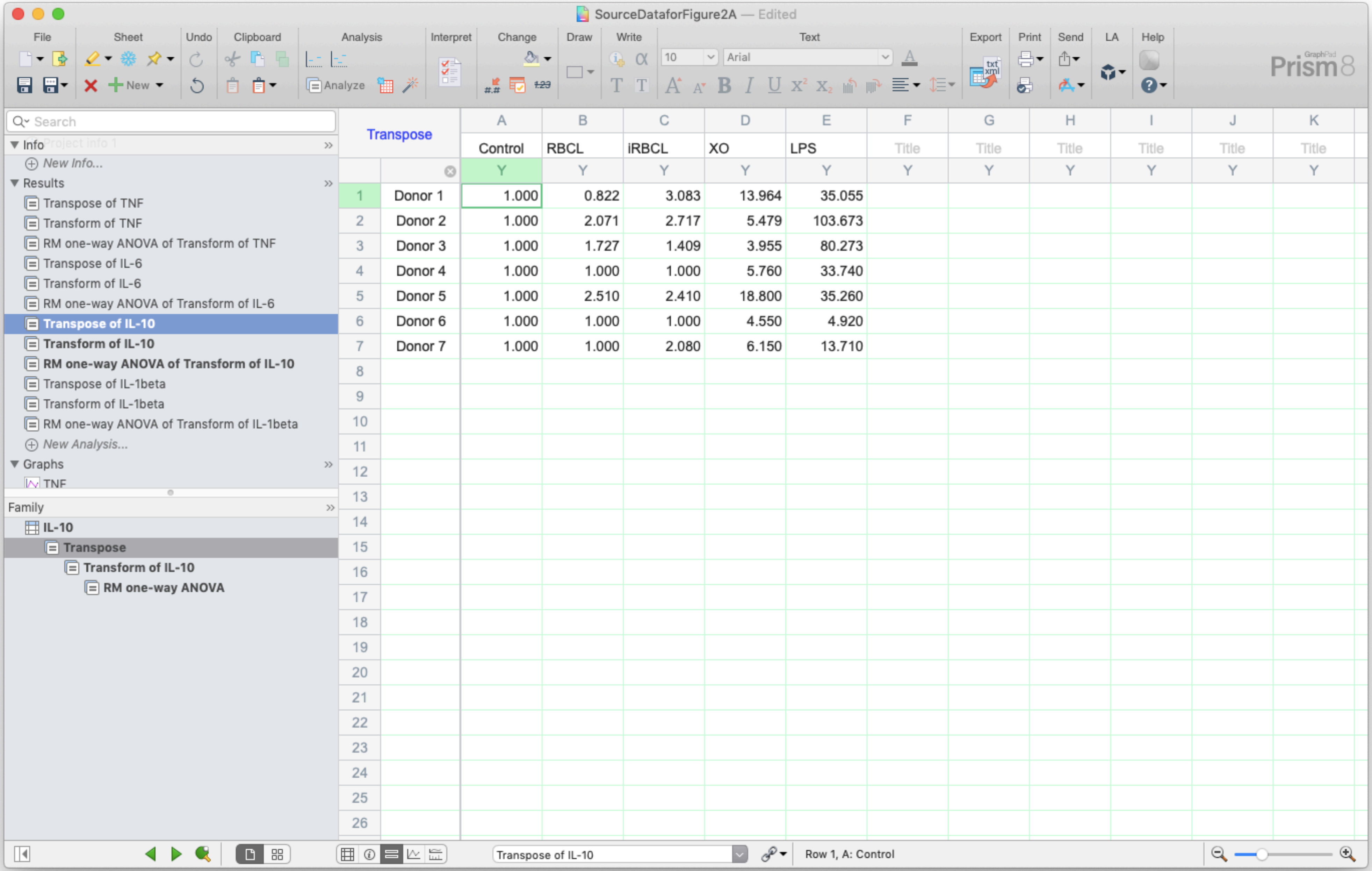

Transpose	Control	RBCL	iRBCL	XO	LPS
Donor 1	1.000	0.822	3.083	13.964	35.055
Donor 2	1.000	2.071	2.717	5.479	103.673
Donor 3	1.000	1.727	1.409	3.955	80.273
Donor 4	1.000	1.000	1.000	5.760	33.740
Donor 5	1.000	2.510	2.410	18.800	35.260
Donor 6	1.000	1.000	1.000	4.550	4.920
Donor 7	1.000	1.000	2.080	6.150	13.710

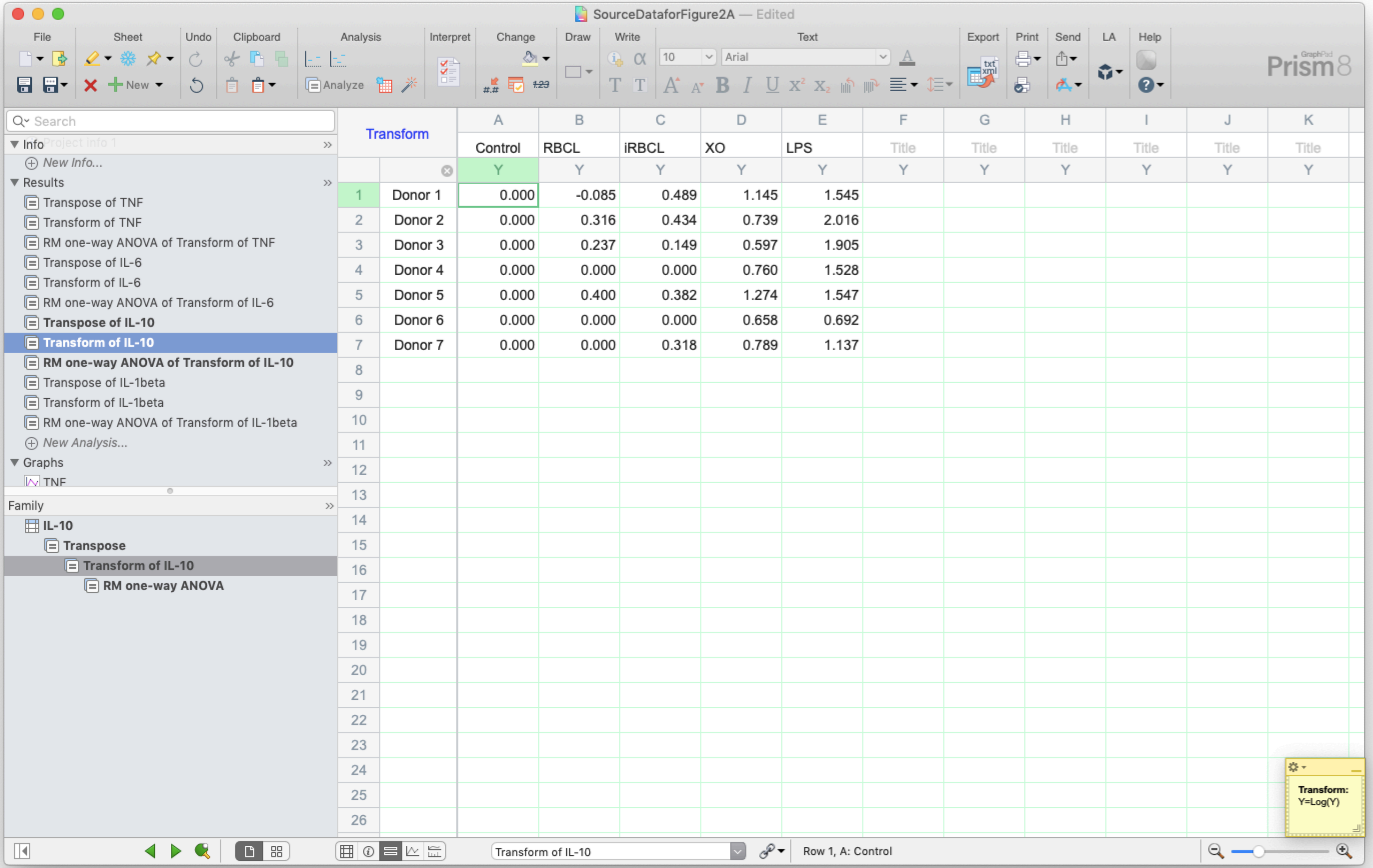

Transform	Control	RBCL	iRBCL	XO	LPS
Donor 1	0.000	-0.085	0.489	1.145	1.545
Donor 2	0.000	0.316	0.434	0.739	2.016
Donor 3	0.000	0.237	0.149	0.597	1.905
Donor 4	0.000	0.000	0.000	0.760	1.528
Donor 5	0.000	0.400	0.382	1.274	1.547
Donor 6	0.000	0.000	0.000	0.658	0.692
Donor 7	0.000	0.000	0.318	0.789	1.137

SourceDataforFigure2A — Edited

RM one-way ANOVA

ANOVA results

1	Table Analyzed	Transform of IL-10				
2						
3	**Repeated measures ANOVA summary**					
4	Assume sphericity?	No				
5	F	49.36				
6	P value	<0.0001				
7	P value summary	****				
8	Statistically significant (P < 0.05)?	Yes				
9	Geisser-Greenhouse's epsilon	0.4940				
10	R square	0.8916				
11						
12	**Was the matching effective?**					
13	F	2.367				
14	P value	0.0614				
15	P value summary	ns				
16	Is there significant matching (P < 0.05)?	No				
17	R square	0.06027				
18						
19	**ANOVA table**	**SS**	**DF**	**MS**	**F (DFn, DFd)**	**P value**
20	Treatment (between columns)	10.71	4	2.678	F (1.976, 11.85) = 49.36	P<0.0001
21	Individual (between rows)	0.7705	6	0.1284	F (6, 24) = 2.367	P=0.0614
22	Residual (random)	1.302	24	0.05426		
23	Total	12.78	34			
24						
25	**Data summary**					

Results: Transpose of TNF; Transform of TNF; RM one-way ANOVA of Transform of TNF; Transpose of IL-6; Transform of IL-6; RM one-way ANOVA of Transform of IL-6; Transpose of IL-10; Transform of IL-10; RM one-way ANOVA of Transform of IL-10; Transpose of IL-1beta; Transform of IL-1beta; RM one-way ANOVA of Transform of IL-1beta

Family: IL-10 > Transpose > Transform of IL-10 > RM one-way ANOVA

RM one-way ANOVA of Transform of IL-10 — Row 1, Column A

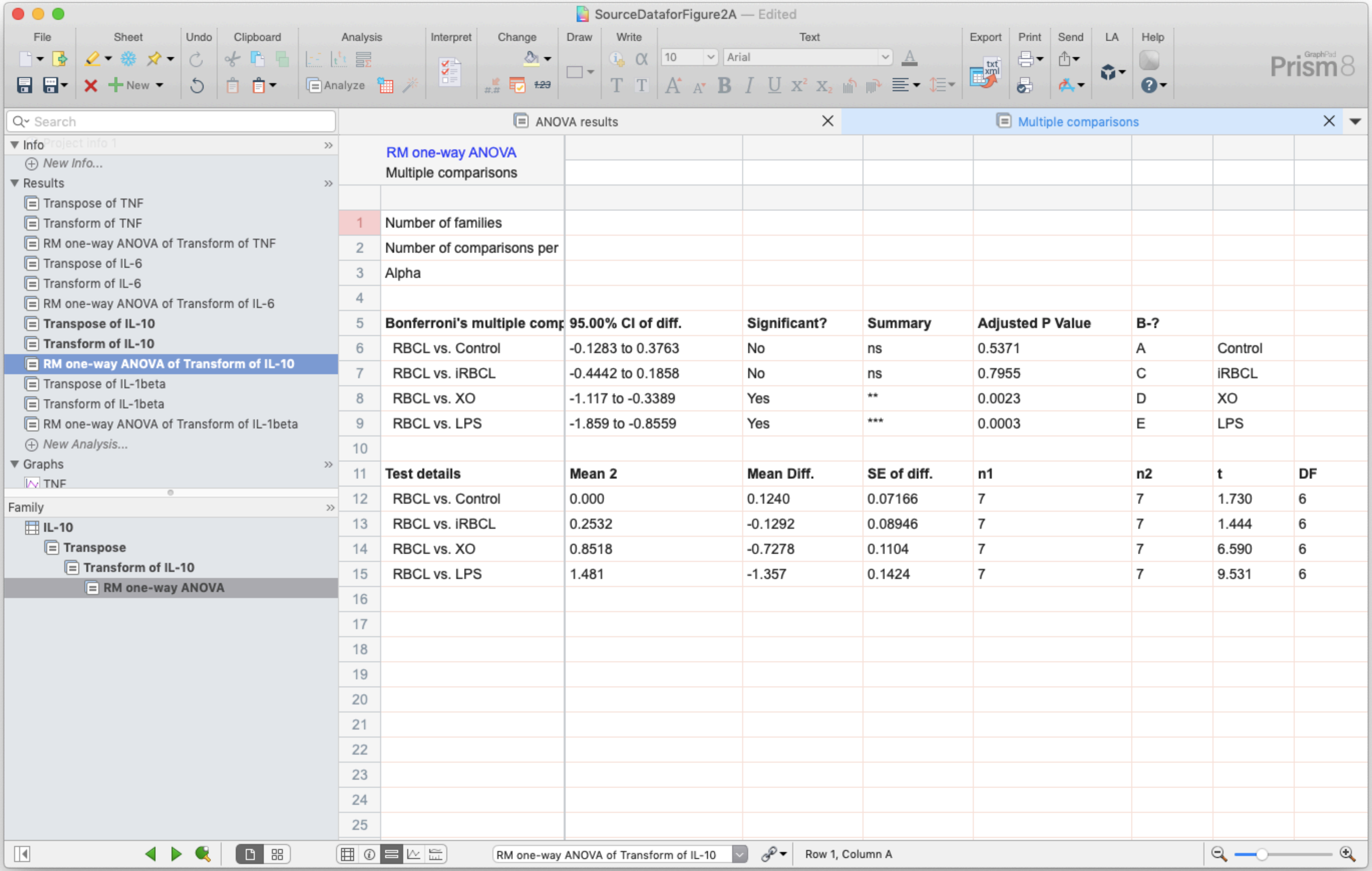

RM one-way ANOVA

Multiple comparisons

1	Number of families							
2	Number of comparisons per							
3	Alpha							
4								
5	**Bonferroni's multiple comp**	**95.00% CI of diff.**	**Significant?**	**Summary**	**Adjusted P Value**	**B-?**		
6	RBCL vs. Control	-0.1283 to 0.3763	No	ns	0.5371	A	Control	
7	RBCL vs. iRBCL	-0.4442 to 0.1858	No	ns	0.7955	C	iRBCL	
8	RBCL vs. XO	-1.117 to -0.3389	Yes	**	0.0023	D	XO	
9	RBCL vs. LPS	-1.859 to -0.8559	Yes	***	0.0003	E	LPS	
10								
11	**Test details**	**Mean 2**	**Mean Diff.**	**SE of diff.**	**n1**	**n2**	**t**	**DF**
12	RBCL vs. Control	0.000	0.1240	0.07166	7	7	1.730	6
13	RBCL vs. iRBCL	0.2532	-0.1292	0.08946	7	7	1.444	6
14	RBCL vs. XO	0.8518	-0.7278	0.1104	7	7	6.590	6
15	RBCL vs. LPS	1.481	-1.357	0.1424	7	7	9.531	6

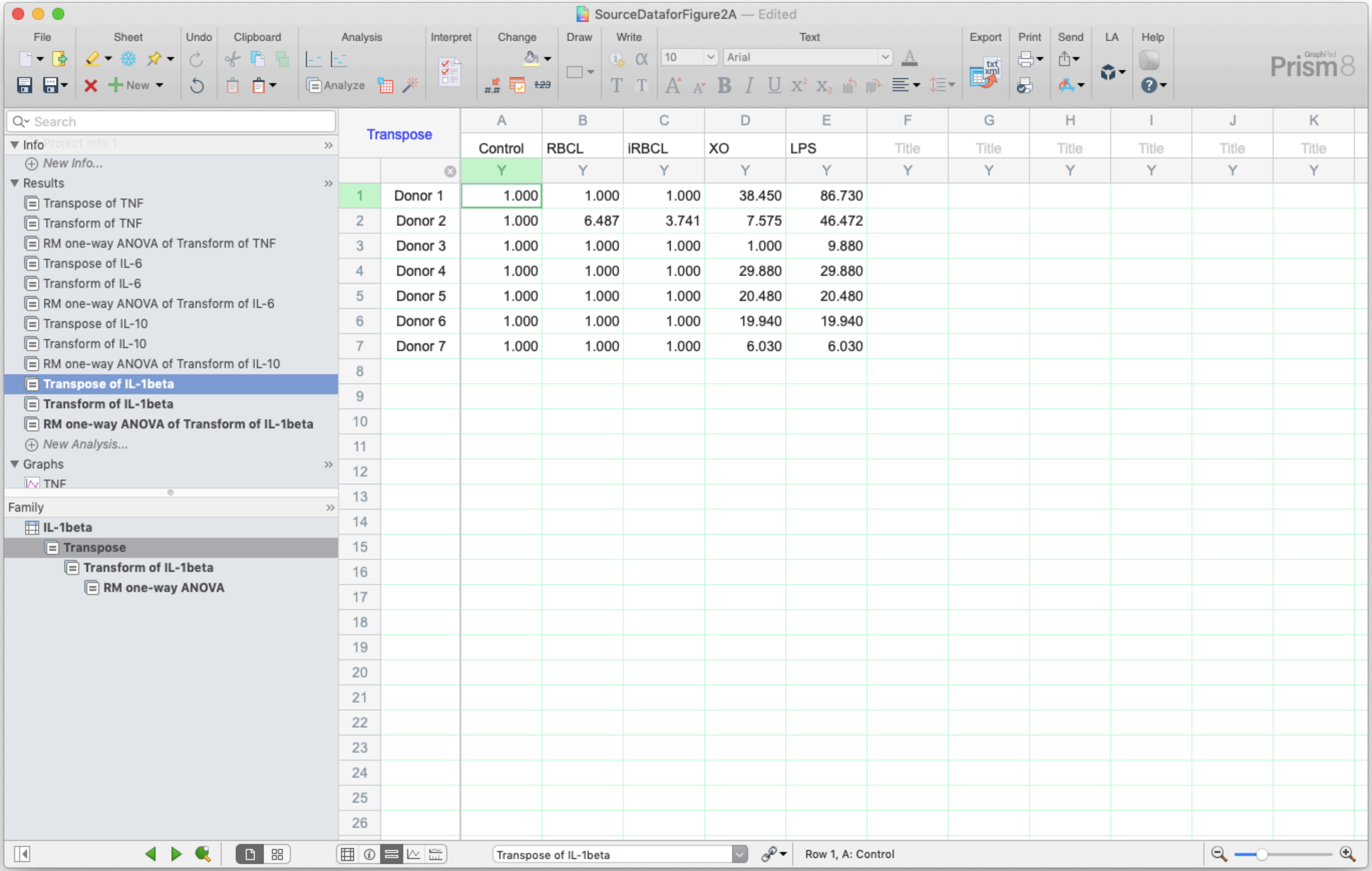

Transpose	A Control Y	B RBCL Y	C iRBCL Y	D XO Y	E LPS Y
1 Donor 1	1.000	1.000	1.000	38.450	86.730
2 Donor 2	1.000	6.487	3.741	7.575	46.472
3 Donor 3	1.000	1.000	1.000	1.000	9.880
4 Donor 4	1.000	1.000	1.000	29.880	29.880
5 Donor 5	1.000	1.000	1.000	20.480	20.480
6 Donor 6	1.000	1.000	1.000	19.940	19.940
7 Donor 7	1.000	1.000	1.000	6.030	6.030

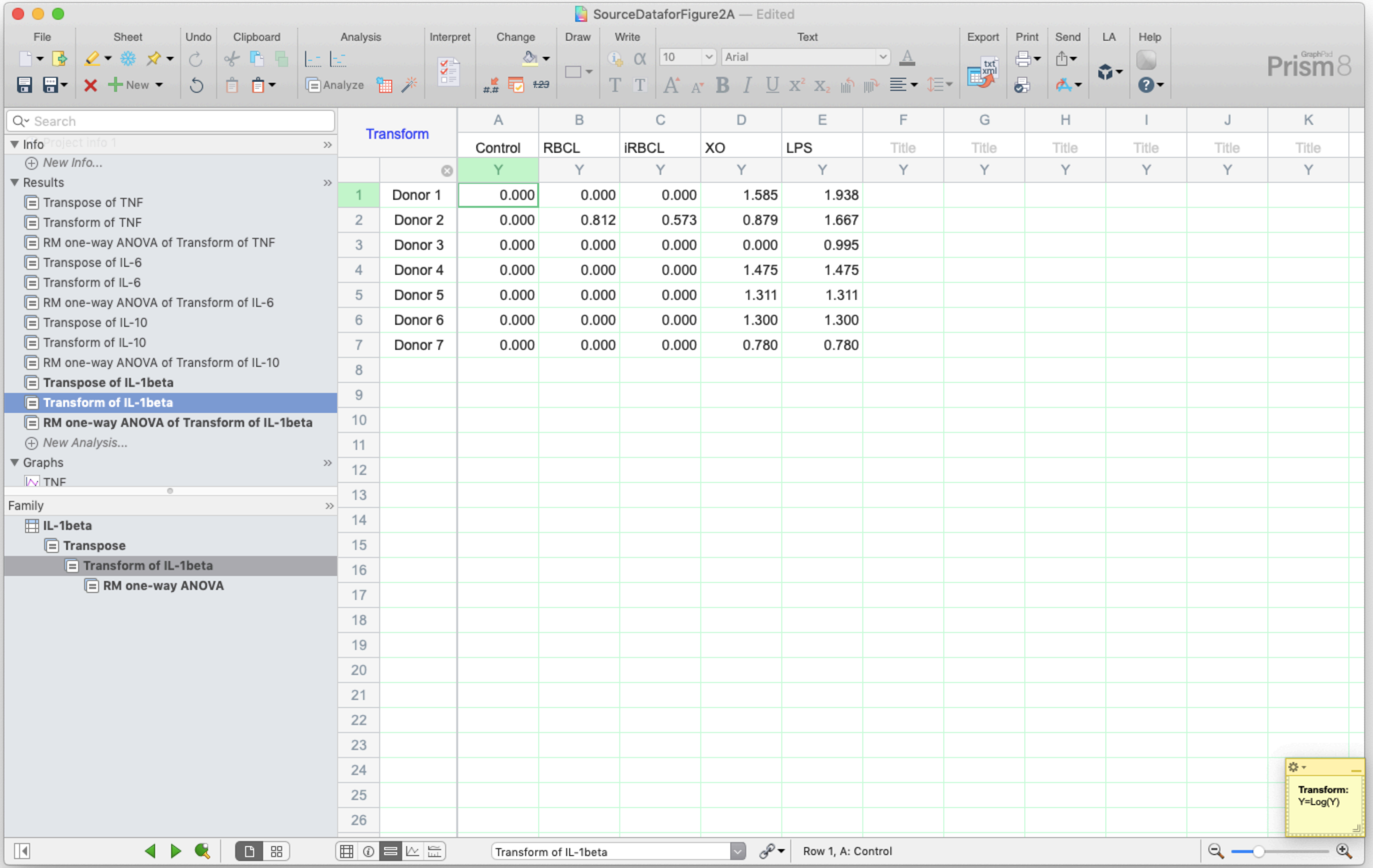

	Control	RBCL	iRBCL	XO	LPS
Donor 1	0.000	0.000	0.000	1.585	1.938
Donor 2	0.000	0.812	0.573	0.879	1.667
Donor 3	0.000	0.000	0.000	0.000	0.995
Donor 4	0.000	0.000	0.000	1.475	1.475
Donor 5	0.000	0.000	0.000	1.311	1.311
Donor 6	0.000	0.000	0.000	1.300	1.300
Donor 7	0.000	0.000	0.000	0.780	0.780

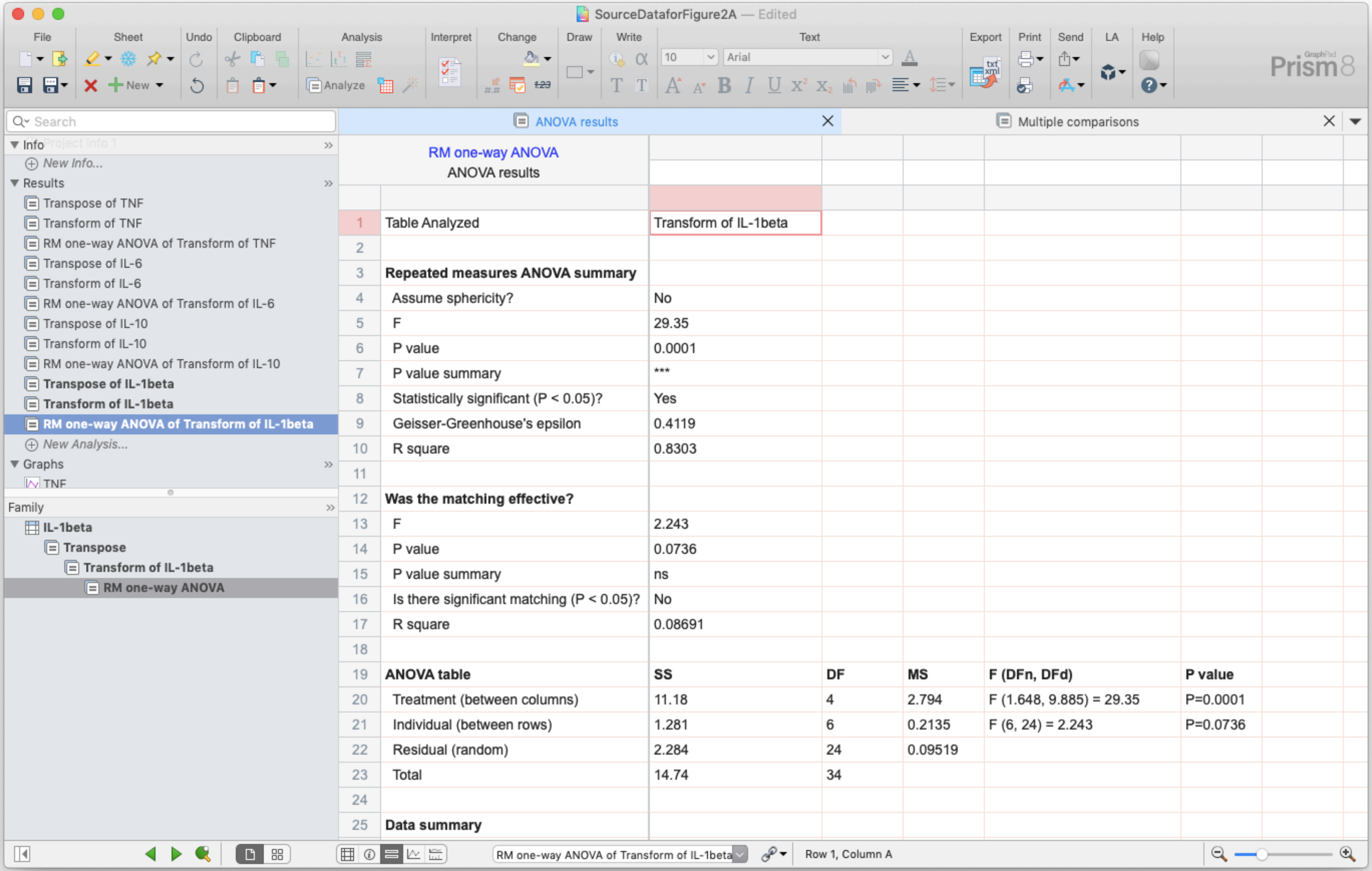

	RM one-way ANOVA<br>ANOVA results				
1	Table Analyzed	Transform of IL-1beta			
2					
3	**Repeated measures ANOVA summary**				
4	Assume sphericity?	No			
5	F	29.35			
6	P value	0.0001			
7	P value summary	***			
8	Statistically significant (P < 0.05)?	Yes			
9	Geisser-Greenhouse's epsilon	0.4119			
10	R square	0.8303			
11					
12	**Was the matching effective?**				
13	F	2.243			
14	P value	0.0736			
15	P value summary	ns			
16	Is there significant matching (P < 0.05)?	No			
17	R square	0.08691			
18					
19	**ANOVA table**	**SS**	**DF**	**MS**	**F (DFn, DFd)**	**P value**
20	Treatment (between columns)	11.18	4	2.794	F (1.648, 9.885) = 29.35	P=0.0001
21	Individual (between rows)	1.281	6	0.2135	F (6, 24) = 2.243	P=0.0736
22	Residual (random)	2.284	24	0.09519		
23	Total	14.74	34			
24						
25	**Data summary**					

RM one-way ANOVA
Multiple comparisons

1	Number of families							
2	Number of comparisons p							
3	Alpha							
4								
5	**Bonferroni's multiple co**	**95.00% CI of diff.**	**Significant?**	**Summary**	**Adjusted P Value**	**B-?**		
6	RBCL vs. Control	-0.2925 to 0.5245	No	ns	>0.9999	A	Control	
7	RBCL vs. iRBCL	-0.08612 to 0.1544	No	ns	>0.9999	C	iRBCL	
8	RBCL vs. XO	-1.814 to -0.04848	Yes	*	0.0397	D	XO	
9	RBCL vs. LPS	-1.772 to -0.7010	Yes	***	0.0007	E	LPS	
10								
11	**Test details**	**Mean 2**	**Mean Diff.**	**SE of diff.**	**n1**	**n2**	**t**	**DF**
12	RBCL vs. Control	0.000	0.1160	0.1160	7	7	1.000	6
13	RBCL vs. iRBCL	0.08185	0.03416	0.03416	7	7	1.000	6
14	RBCL vs. XO	1.047	-0.9313	0.2507	7	7	3.715	6
15	RBCL vs. LPS	1.352	-1.236	0.1520	7	7	8.132	6

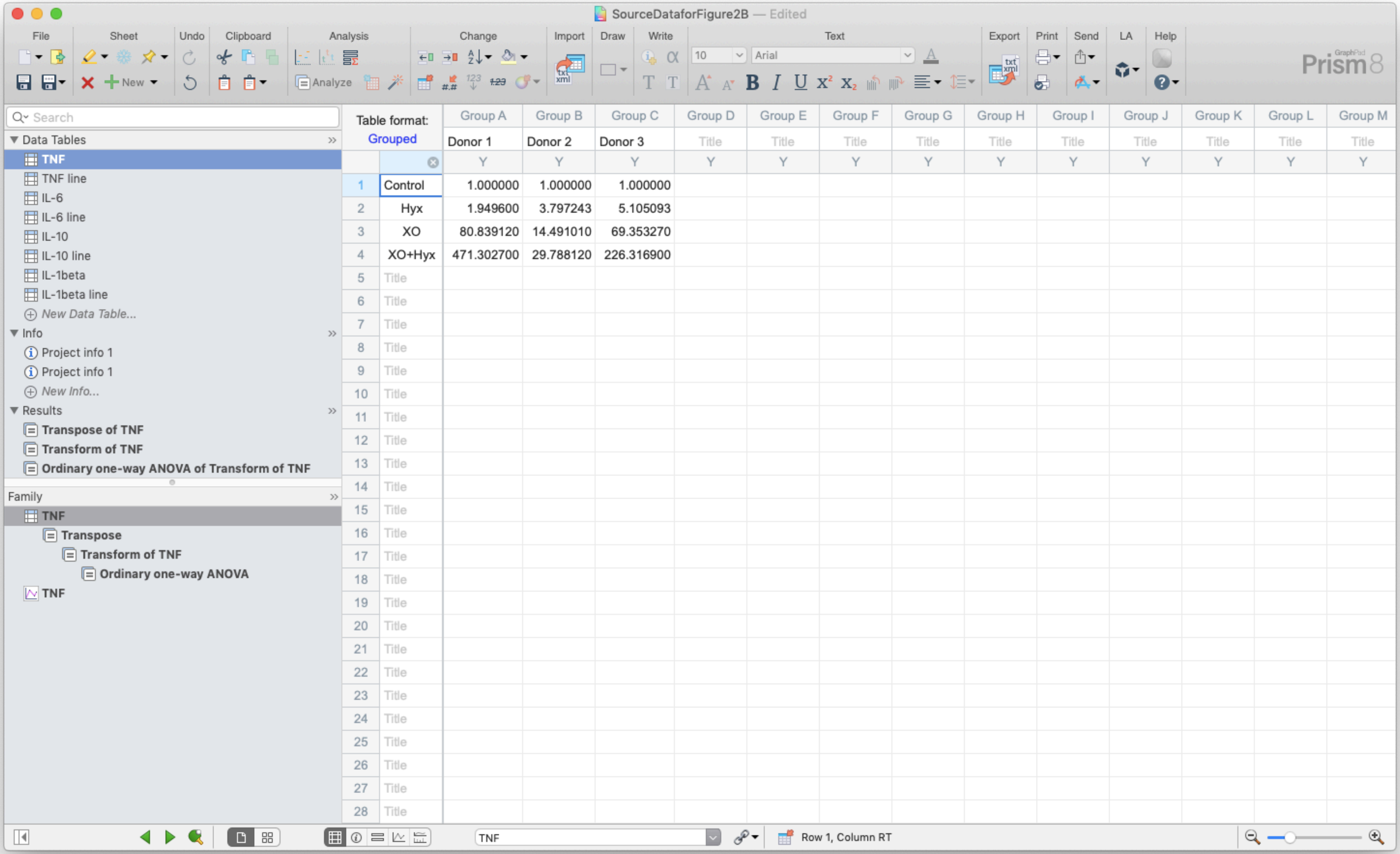

		Group A	Group B	Group C
		Donor 1	Donor 2	Donor 3
		Y	Y	Y
1	Control	1.000000	1.000000	1.000000
2	Hyx	1.949600	3.797243	5.105093
3	XO	80.839120	14.491010	69.353270
4	XO+Hyx	471.302700	29.788120	226.316900

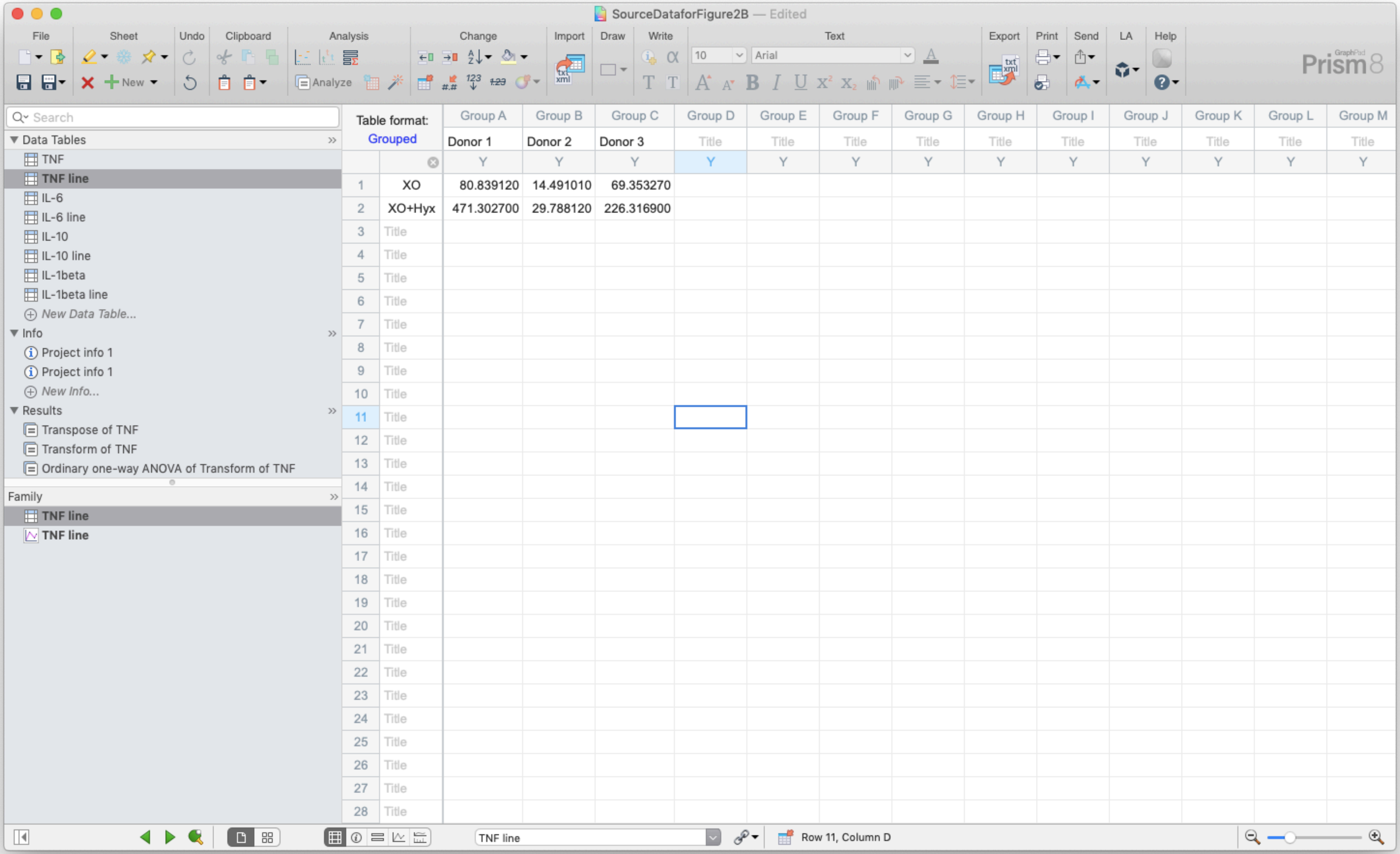
SourceDataforFigure2B — Edited
Data Tables
TNF
TNF line
IL-6
IL-6 line
IL-10
IL-10 line
IL-1beta
IL-1beta line
New Data Table...
Info
Project info 1
Project info 1
New Info...
Results
Transpose of TNF
Transform of TNF
Ordinary one-way ANOVA of Transform of TNF
Family
TNF line
TNF line
Table format: Grouped

	Group A	Group B	Group C
	Donor 1	Donor 2	Donor 3
	Y	Y	Y
XO	80.839120	14.491010	69.353270
XO+Hyx	471.302700	29.788120	226.316900

TNF line
Row 11, Column D

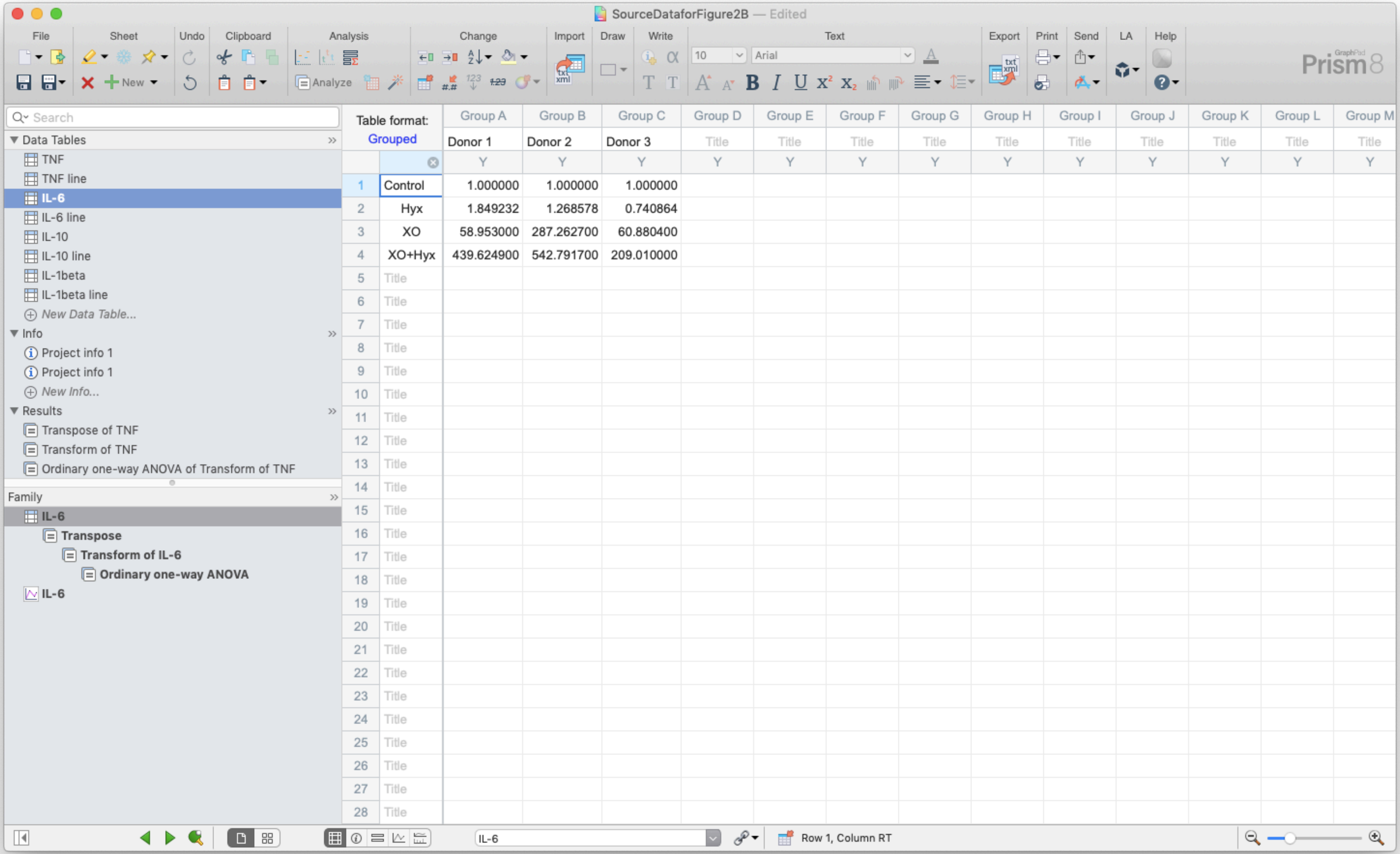

	Group A	Group B	Group C
	Donor 1	Donor 2	Donor 3
Control	1.000000	1.000000	1.000000
Hyx	1.849232	1.268578	0.740864
XO	58.953000	287.262700	60.880400
XO+Hyx	439.624900	542.791700	209.010000

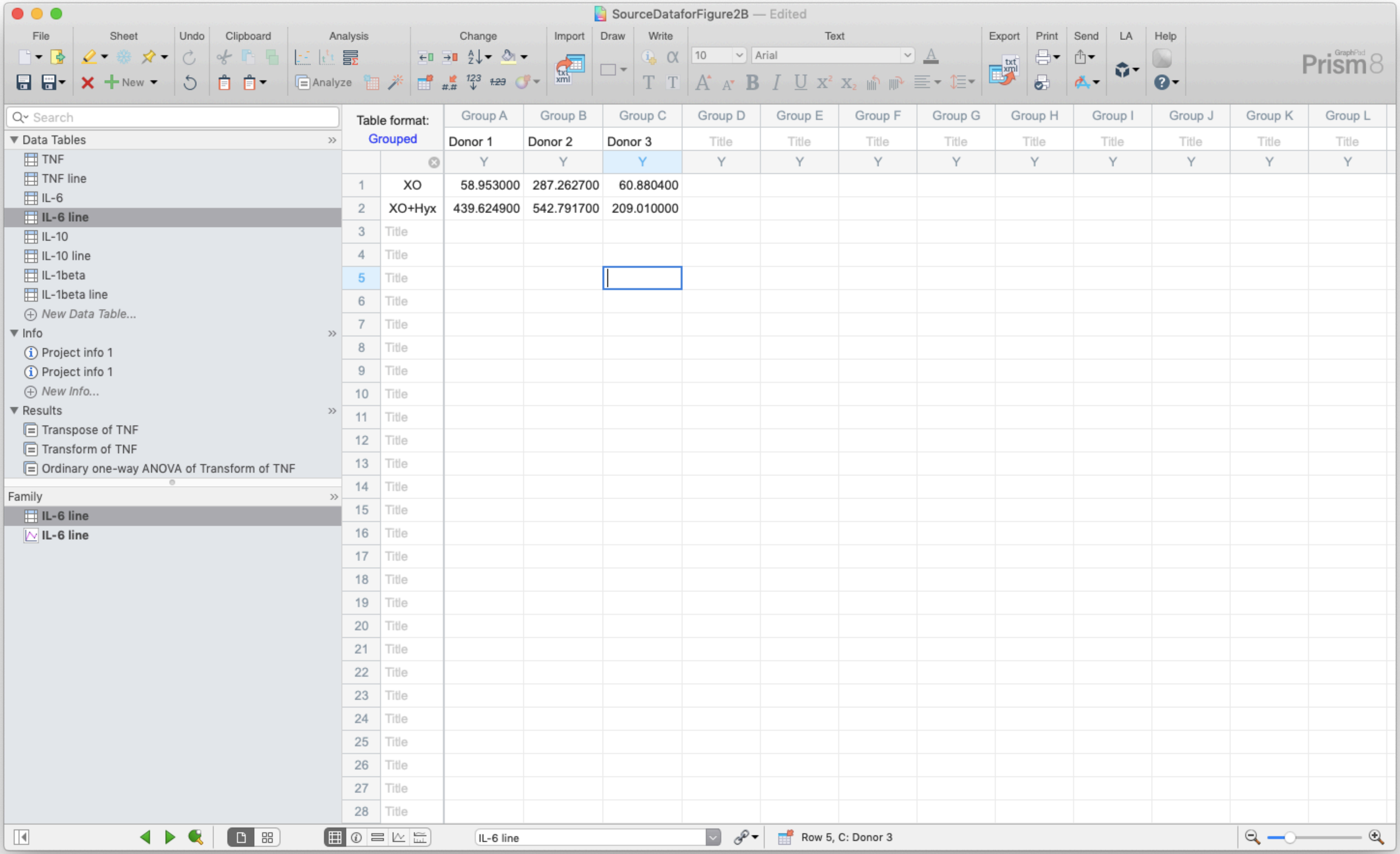

		Group A	Group B	Group C
		Donor 1	Donor 2	Donor 3
		Y	Y	Y
1	XO	58.953000	287.262700	60.880400
2	XO+Hyx	439.624900	542.791700	209.010000

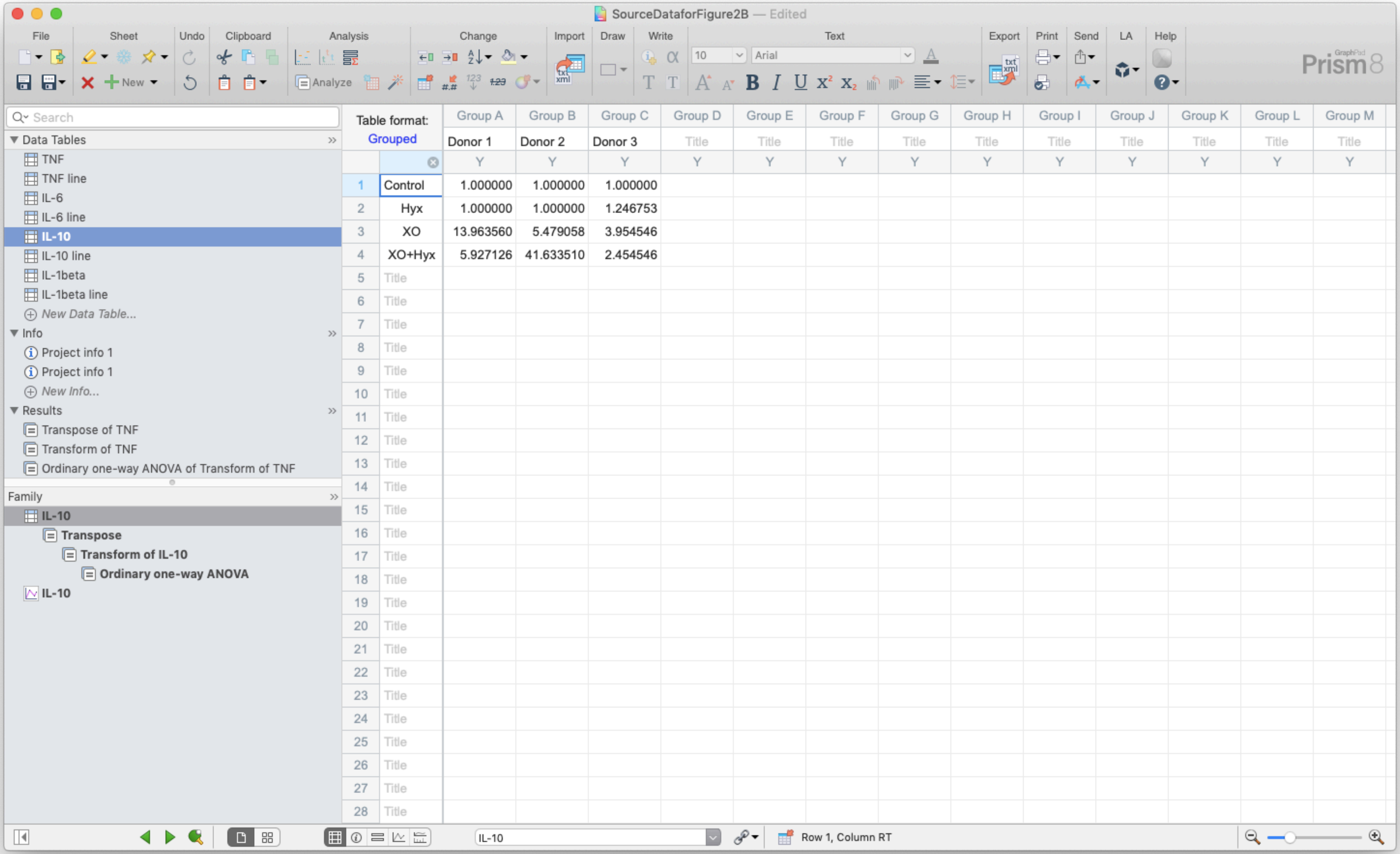

	Group A	Group B	Group C
	Donor 1	Donor 2	Donor 3
Control	1.000000	1.000000	1.000000
Hyx	1.000000	1.000000	1.246753
XO	13.963560	5.479058	3.954546
XO+Hyx	5.927126	41.633510	2.454546

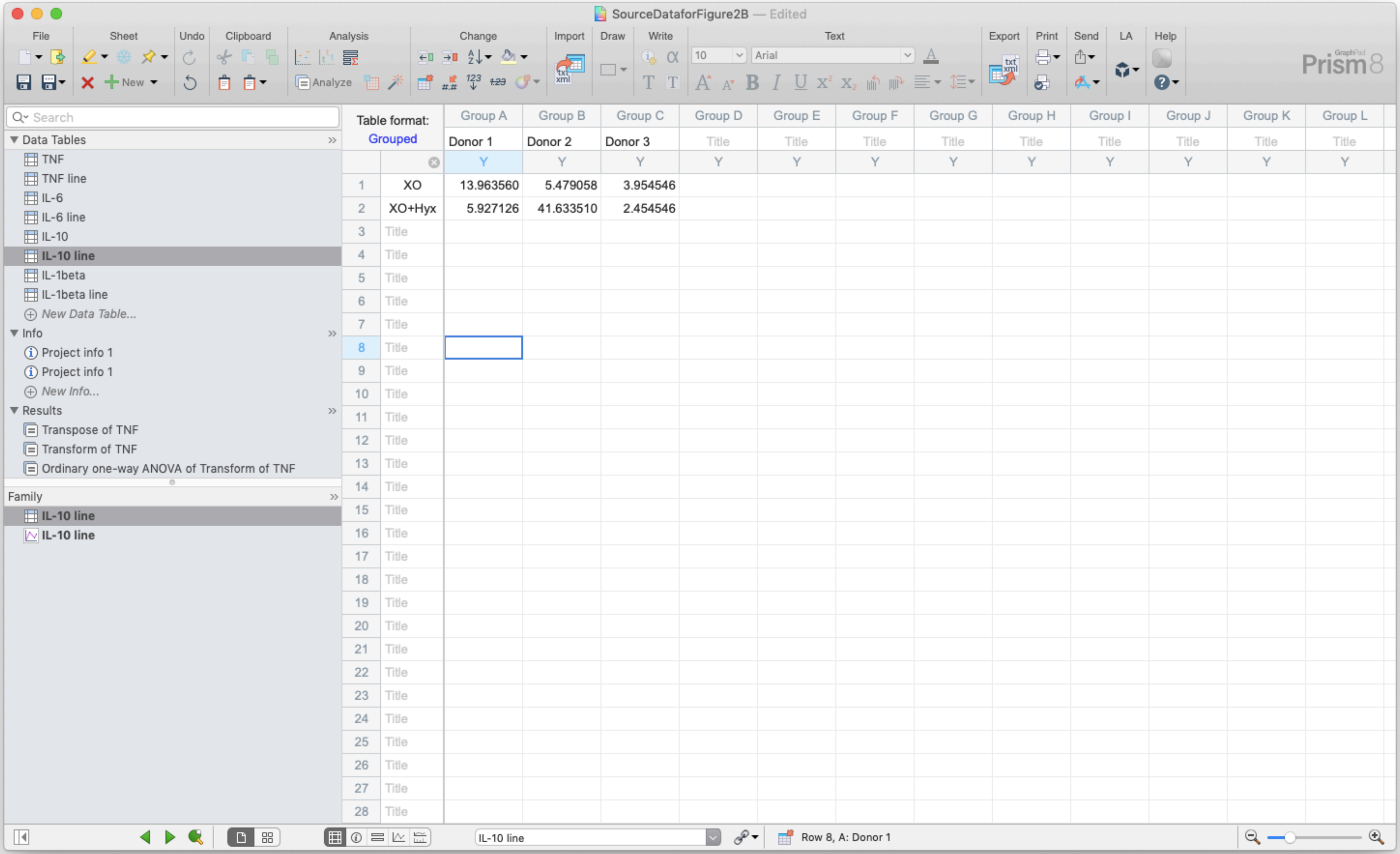

	Group A	Group B	Group C	Group D	Group E	Group F	Group G	Group H	Group I	Group J	Group K	Group L
	Donor 1	Donor 2	Donor 3	Title	Title	Title	Title	Title	Title	Title	Title	Title
	Y	Y	Y	Y	Y	Y	Y	Y	Y	Y	Y	Y
1 XO	13.963560	5.479058	3.954546									
2 XO+Hyx	5.927126	41.633510	2.454546									
3 Title												
4 Title												
5 Title												
6 Title												
7 Title												
8 Title												
9 Title												
10 Title												
11 Title												
12 Title												
13 Title												
14 Title												
15 Title												
16 Title												
17 Title												
18 Title												
19 Title												
20 Title												
21 Title												
22 Title												
23 Title												
24 Title												
25 Title												
26 Title												
27 Title												
28 Title												

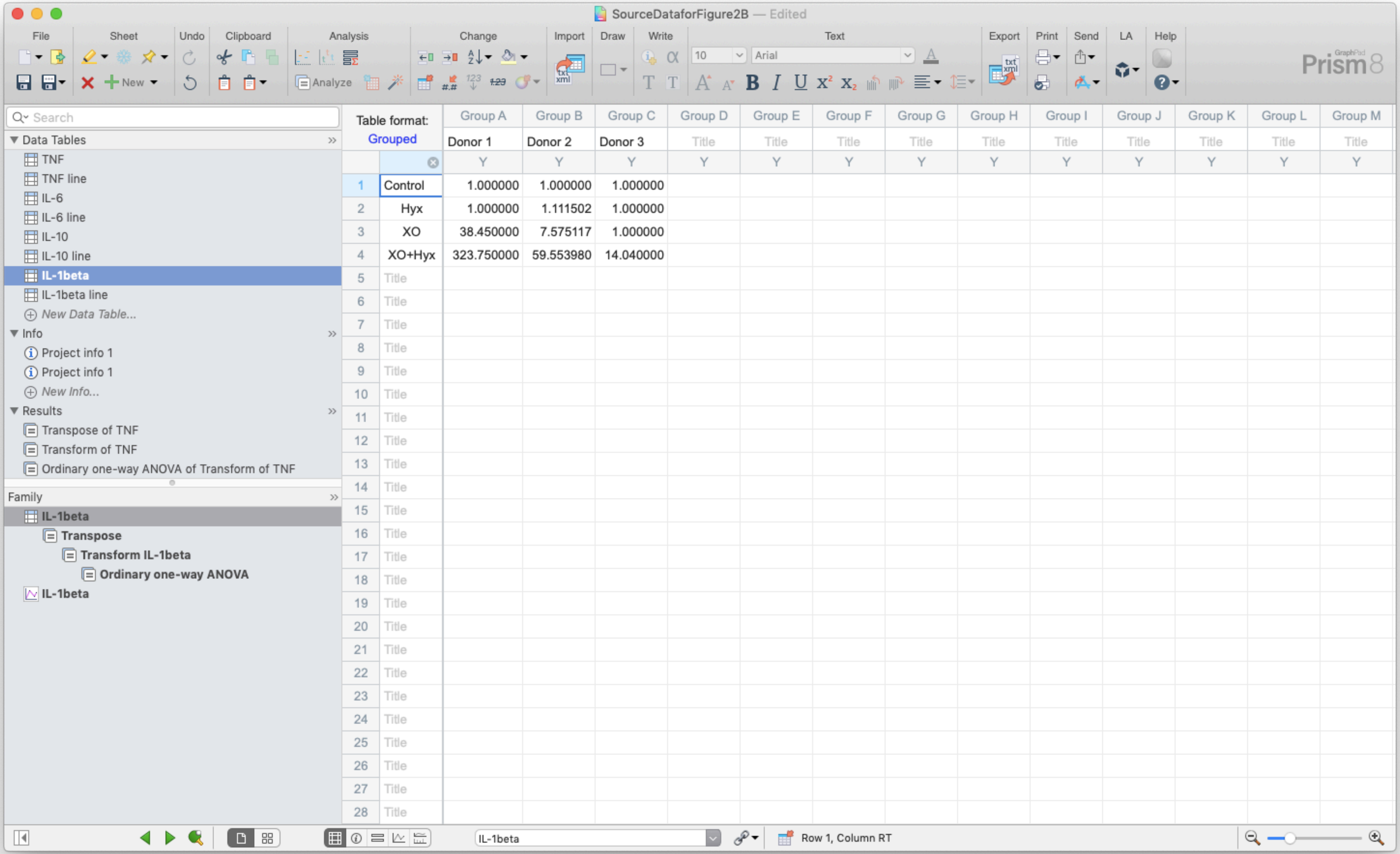

	Group A Donor 1	Group B Donor 2	Group C Donor 3
Control	1.000000	1.000000	1.000000
Hyx	1.000000	1.111502	1.000000
XO	38.450000	7.575117	1.000000
XO+Hyx	323.750000	59.553980	14.040000

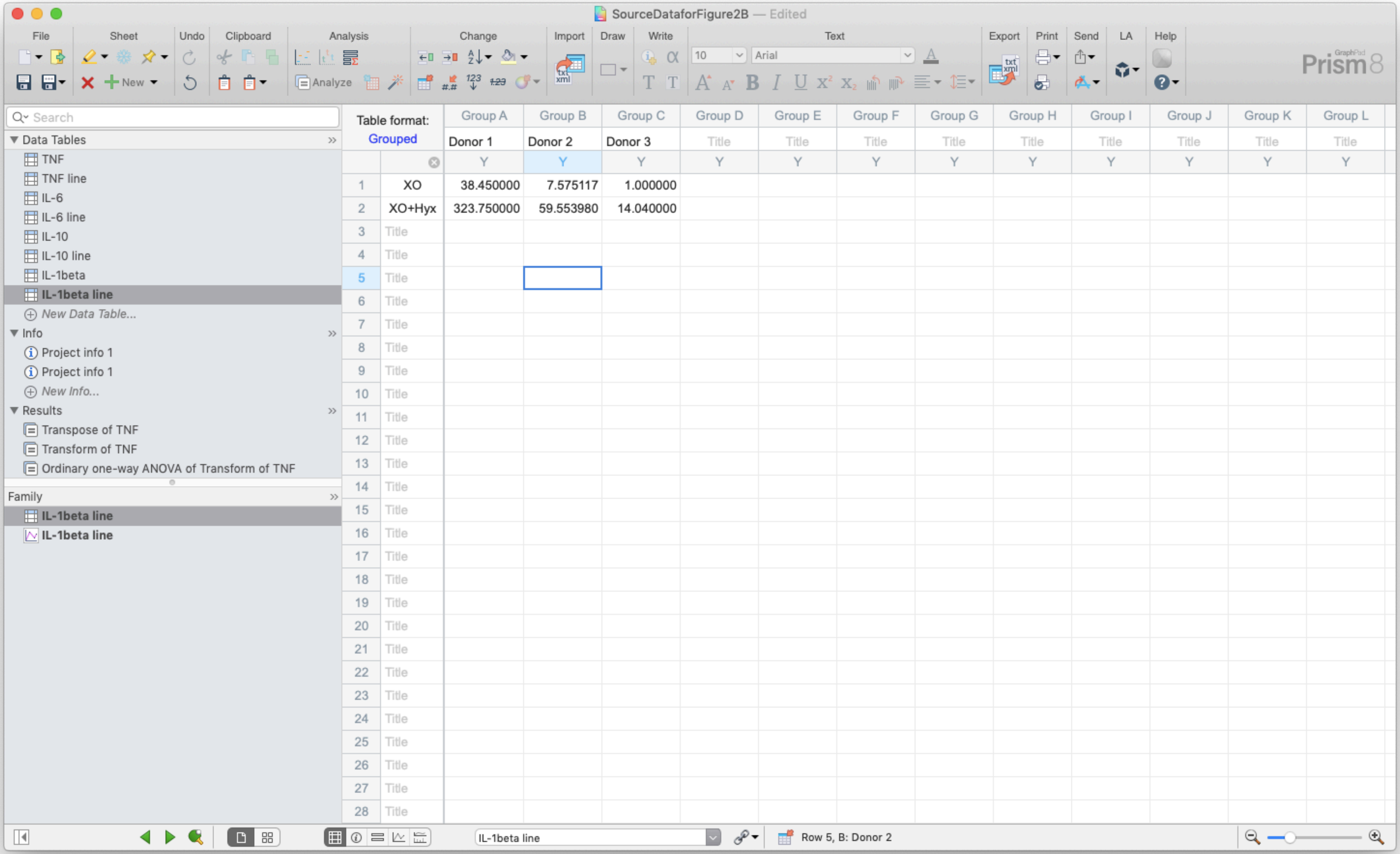
SourceDataforFigure2B — Edited
Data Tables
TNF
TNF line
IL-6
IL-6 line
IL-10
IL-10 line
IL-1beta
IL-1beta line
New Data Table...
Info
Project info 1
Project info 1
New Info...
Results
Transpose of TNF
Transform of TNF
Ordinary one-way ANOVA of Transform of TNF
Family
IL-1beta line
IL-1beta line
Table format: Grouped
Group A Group B Group C
Donor 1 Donor 2 Donor 3
1 XO 38.450000 7.575117 1.000000
2 XO+Hyx 323.750000 59.553980 14.040000
Row 5, B: Donor 2

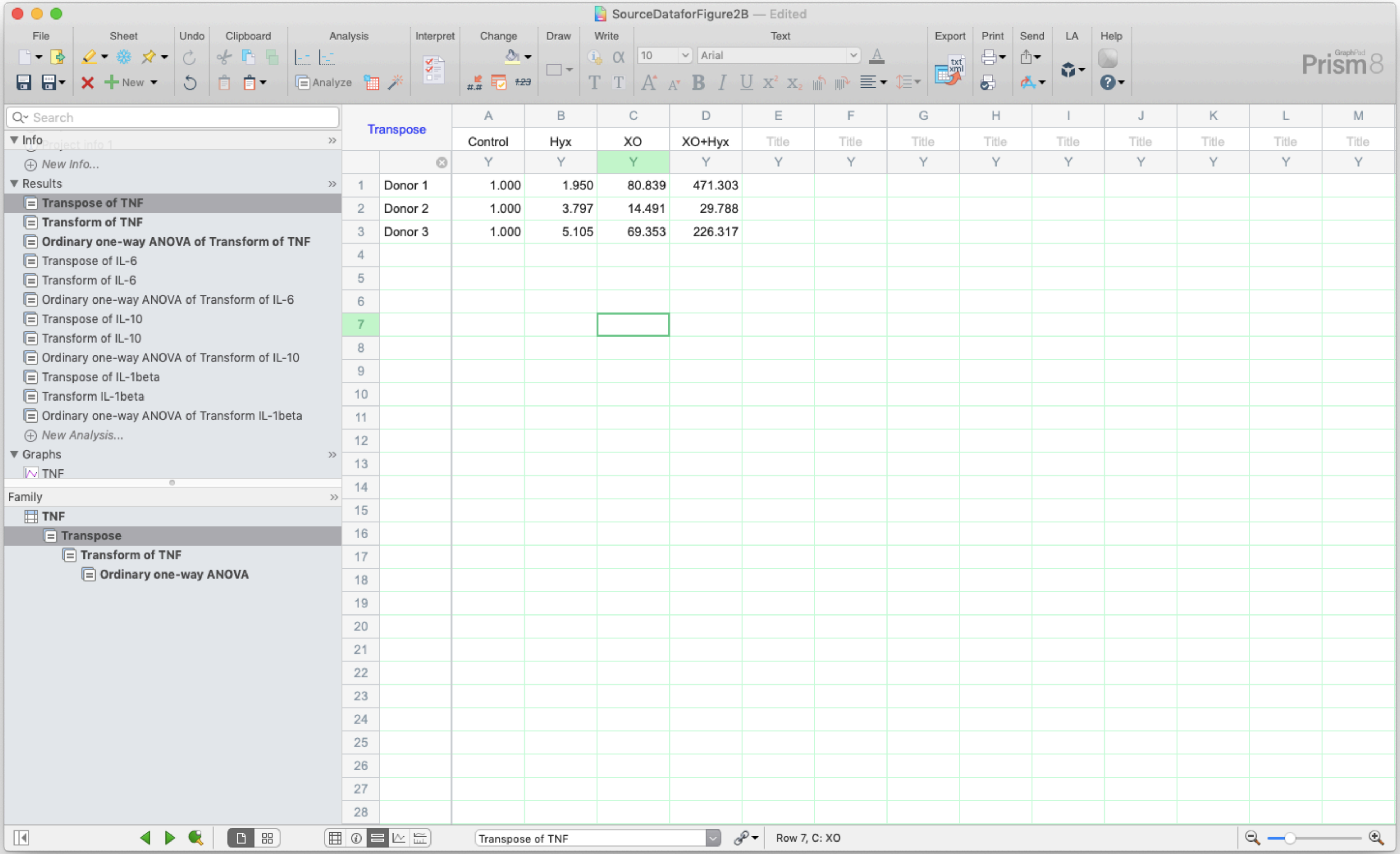

Transpose	Control	Hyx	XO	XO+Hyx
Donor 1	1.000	1.950	80.839	471.303
Donor 2	1.000	3.797	14.491	29.788
Donor 3	1.000	5.105	69.353	226.317

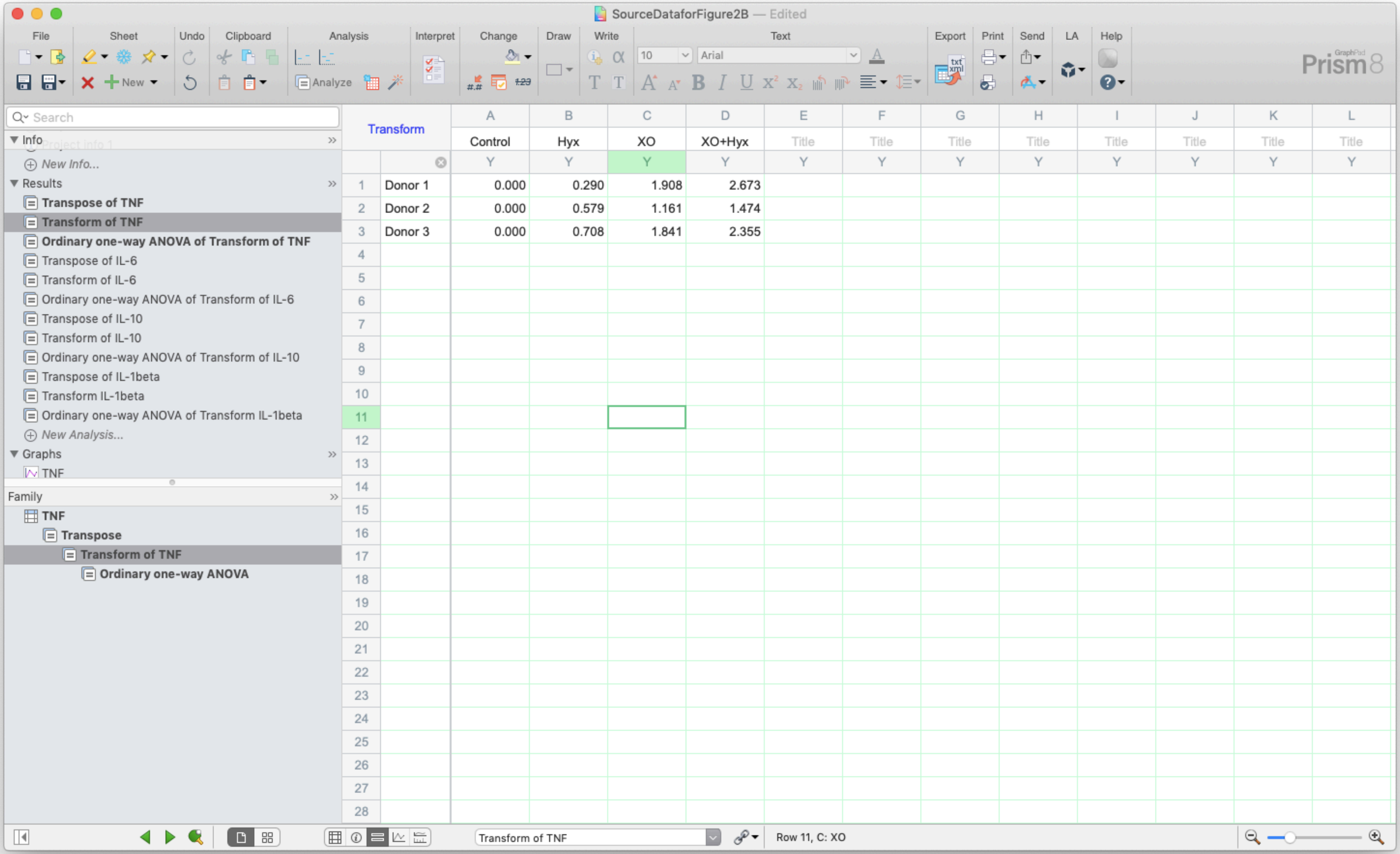

SourceDataforFigure2B — Edited

Transform	A: Control	B: Hyx	C: XO	D: XO+Hyx
	Y	Y	Y	Y
Donor 1	0.000	0.290	1.908	2.673
Donor 2	0.000	0.579	1.161	1.474
Donor 3	0.000	0.708	1.841	2.355

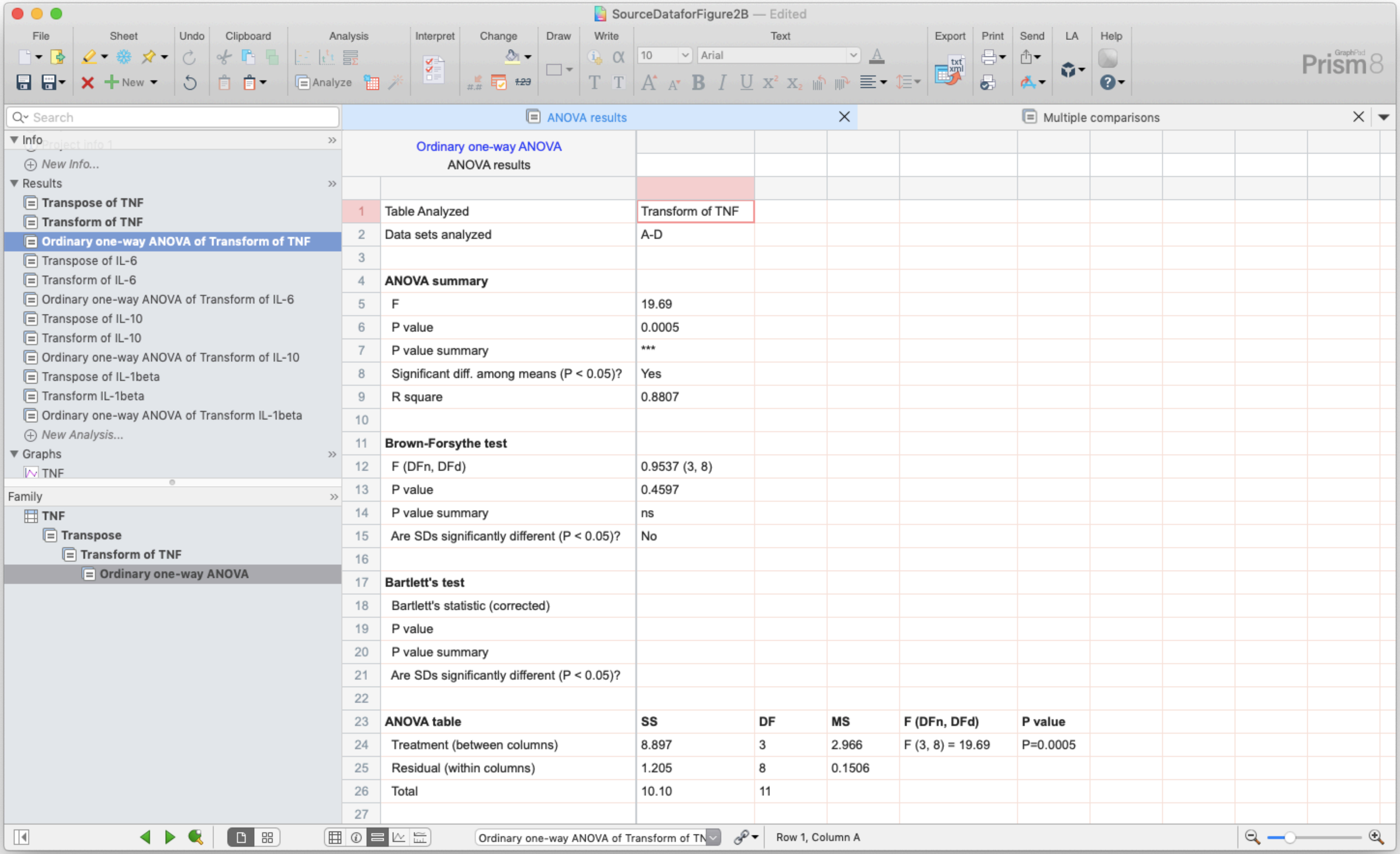

Ordinary one-way ANOVA
ANOVA results

1	Table Analyzed	Transform of TNF				
2	Data sets analyzed	A-D				
3						
4	**ANOVA summary**					
5	F	19.69				
6	P value	0.0005				
7	P value summary	***				
8	Significant diff. among means (P < 0.05)?	Yes				
9	R square	0.8807				
10						
11	**Brown-Forsythe test**					
12	F (DFn, DFd)	0.9537 (3, 8)				
13	P value	0.4597				
14	P value summary	ns				
15	Are SDs significantly different (P < 0.05)?	No				
16						
17	**Bartlett's test**					
18	Bartlett's statistic (corrected)					
19	P value					
20	P value summary					
21	Are SDs significantly different (P < 0.05)?					
22						
23	**ANOVA table**	**SS**	**DF**	**MS**	**F (DFn, DFd)**	**P value**
24	Treatment (between columns)	8.897	3	2.966	F (3, 8) = 19.69	P=0.0005
25	Residual (within columns)	1.205	8	0.1506		
26	Total	10.10	11			
27						

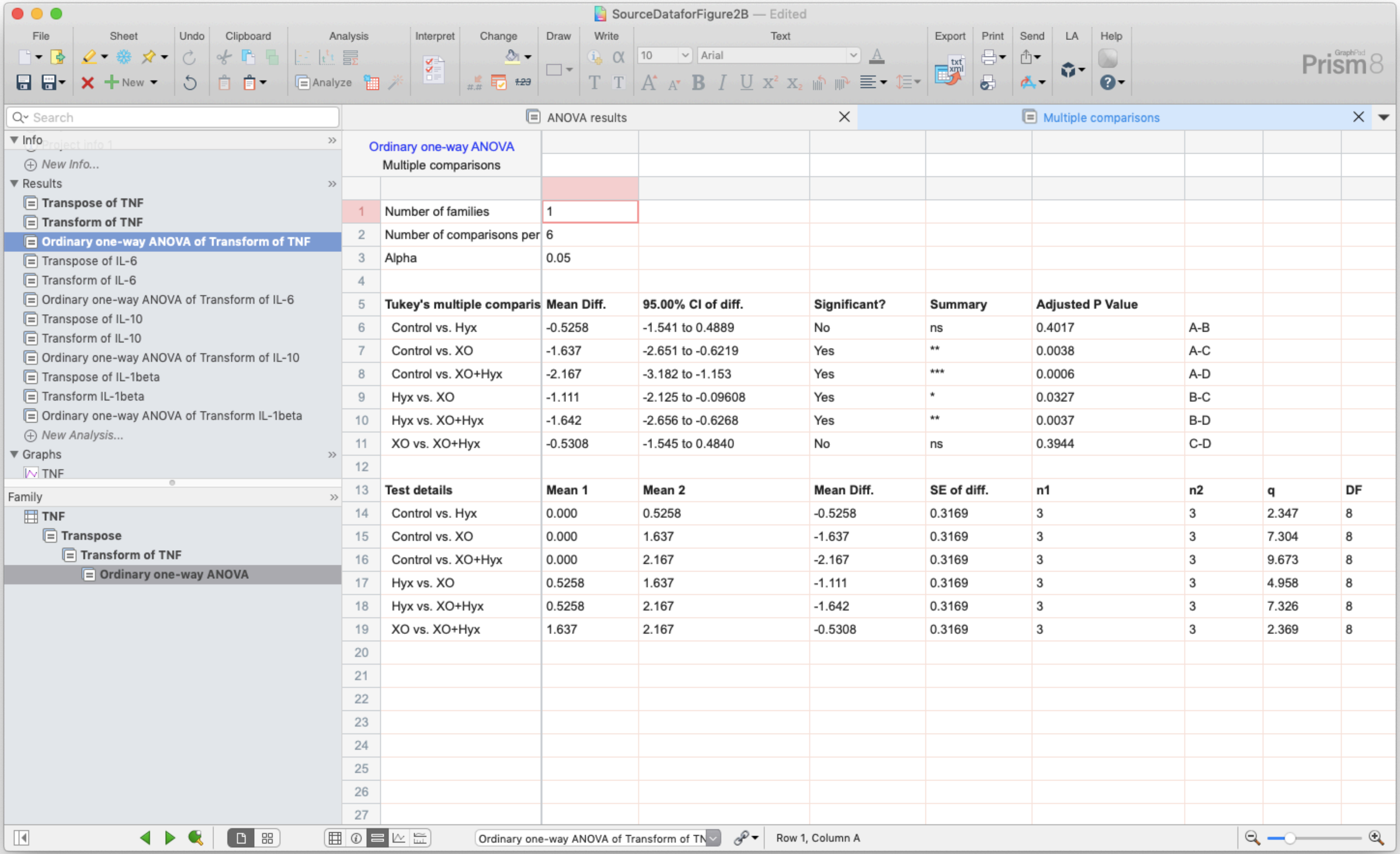

Ordinary one-way ANOVA
Multiple comparisons

1	Number of families	1							
2	Number of comparisons per	6							
3	Alpha	0.05							
4									
5	**Tukey's multiple comparis**	**Mean Diff.**	**95.00% CI of diff.**	**Significant?**	**Summary**	**Adjusted P Value**			
6	Control vs. Hyx	-0.5258	-1.541 to 0.4889	No	ns	0.4017	A-B		
7	Control vs. XO	-1.637	-2.651 to -0.6219	Yes	**	0.0038	A-C		
8	Control vs. XO+Hyx	-2.167	-3.182 to -1.153	Yes	***	0.0006	A-D		
9	Hyx vs. XO	-1.111	-2.125 to -0.09608	Yes	*	0.0327	B-C		
10	Hyx vs. XO+Hyx	-1.642	-2.656 to -0.6268	Yes	**	0.0037	B-D		
11	XO vs. XO+Hyx	-0.5308	-1.545 to 0.4840	No	ns	0.3944	C-D		
12									
13	**Test details**	**Mean 1**	**Mean 2**	**Mean Diff.**	**SE of diff.**	**n1**	**n2**	**q**	**DF**
14	Control vs. Hyx	0.000	0.5258	-0.5258	0.3169	3	3	2.347	8
15	Control vs. XO	0.000	1.637	-1.637	0.3169	3	3	7.304	8
16	Control vs. XO+Hyx	0.000	2.167	-2.167	0.3169	3	3	9.673	8
17	Hyx vs. XO	0.5258	1.637	-1.111	0.3169	3	3	4.958	8
18	Hyx vs. XO+Hyx	0.5258	2.167	-1.642	0.3169	3	3	7.326	8
19	XO vs. XO+Hyx	1.637	2.167	-0.5308	0.3169	3	3	2.369	8

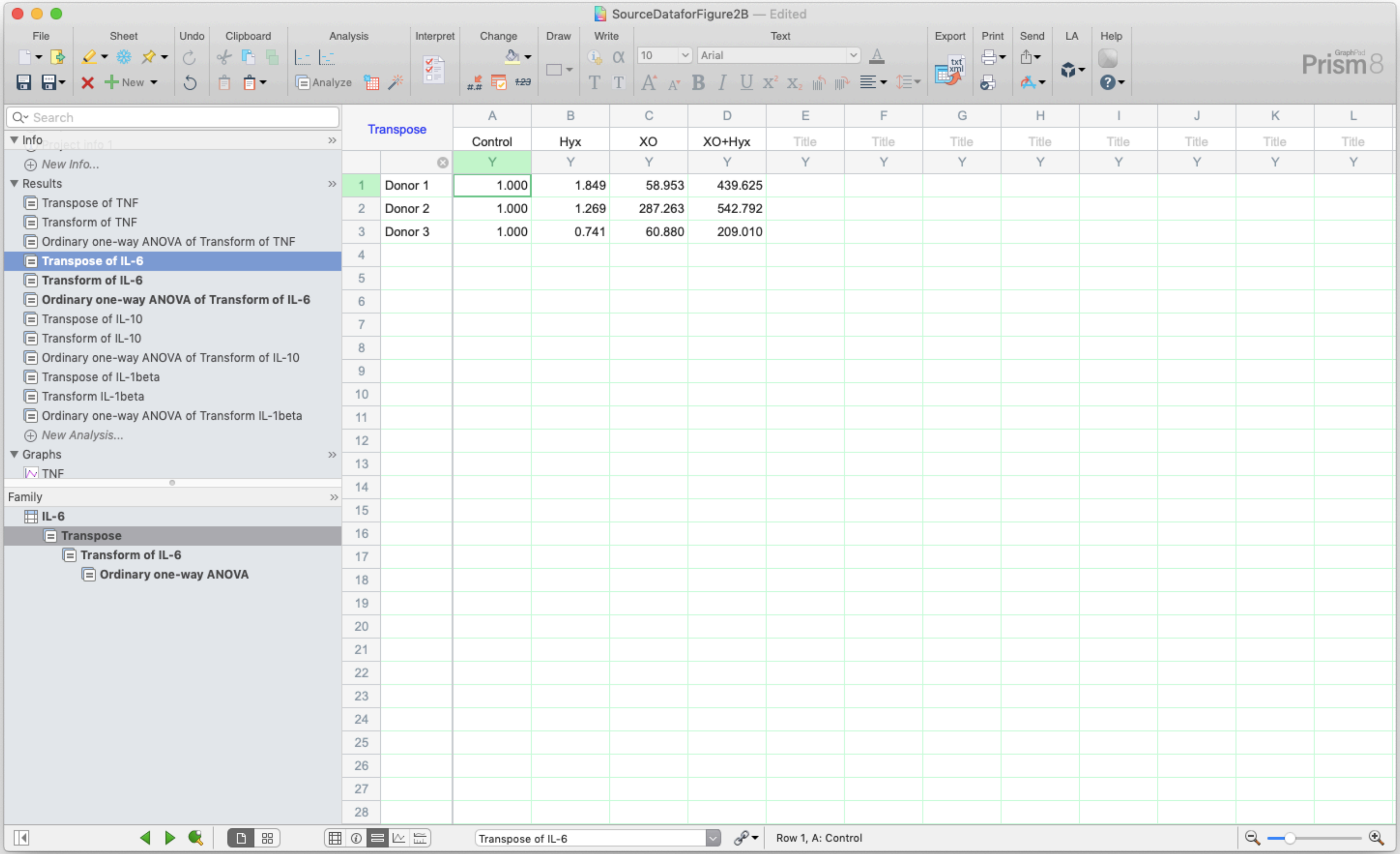
SourceDataforFigure2B — Edited
Transpose
Info
New Info...
Results
Transpose of TNF
Transform of TNF
Ordinary one-way ANOVA of Transform of TNF
Transpose of IL-6
Transform of IL-6
Ordinary one-way ANOVA of Transform of IL-6
Transpose of IL-10
Transform of IL-10
Ordinary one-way ANOVA of Transform of IL-10
Transpose of IL-1beta
Transform IL-1beta
Ordinary one-way ANOVA of Transform IL-1beta
New Analysis...
Graphs
TNF
Family
IL-6
Transpose
Transform of IL-6
Ordinary one-way ANOVA
Control Hyx XO XO+Hyx
Donor 1 1.000 1.849 58.953 439.625
Donor 2 1.000 1.269 287.263 542.792
Donor 3 1.000 0.741 60.880 209.010
Transpose of IL-6
Row 1, A: Control

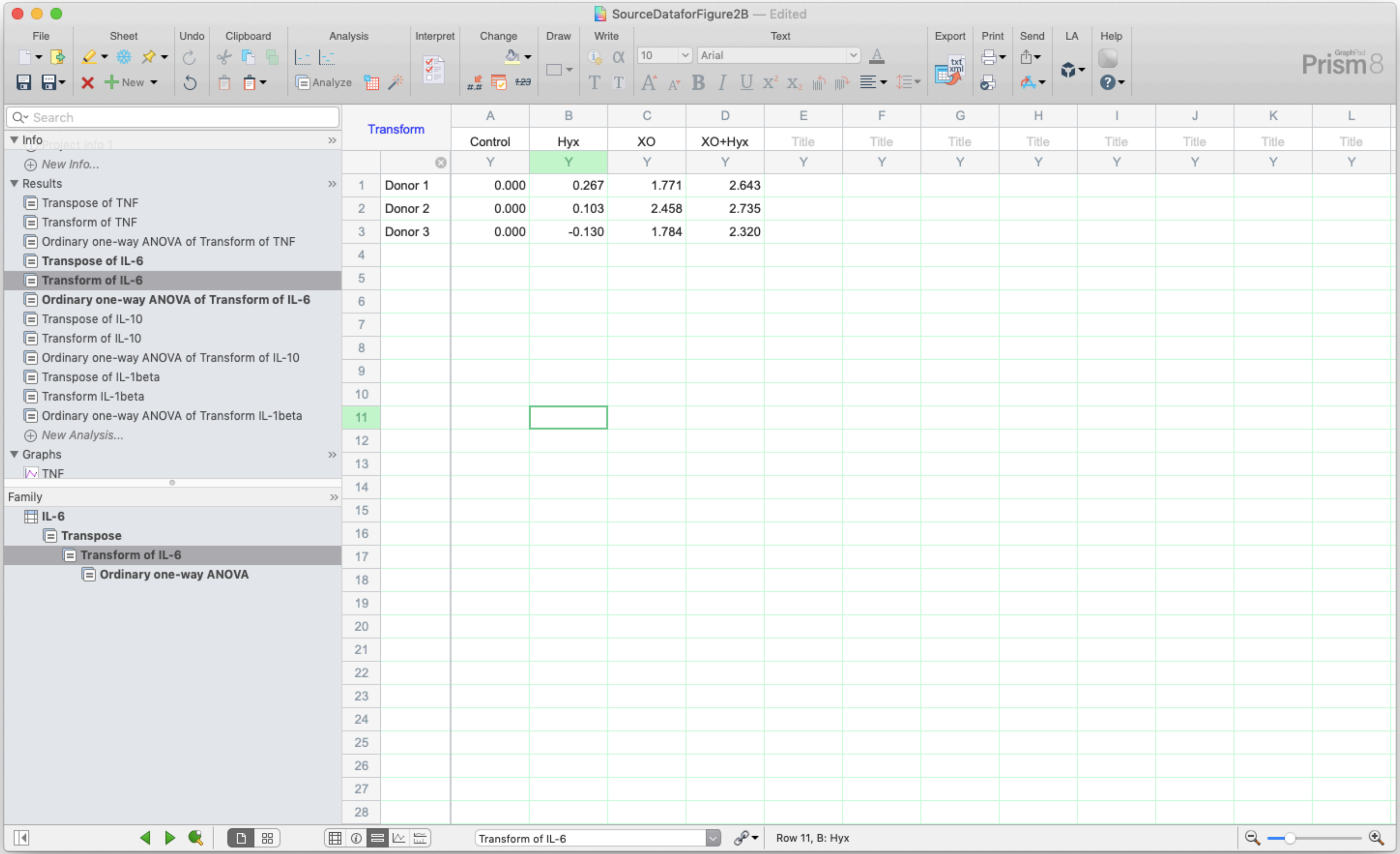

		A	B	C	D
		Control	Hyx	XO	XO+Hyx
		Y	Y	Y	Y
1	Donor 1	0.000	0.267	1.771	2.643
2	Donor 2	0.000	0.103	2.458	2.735
3	Donor 3	0.000	-0.130	1.784	2.320

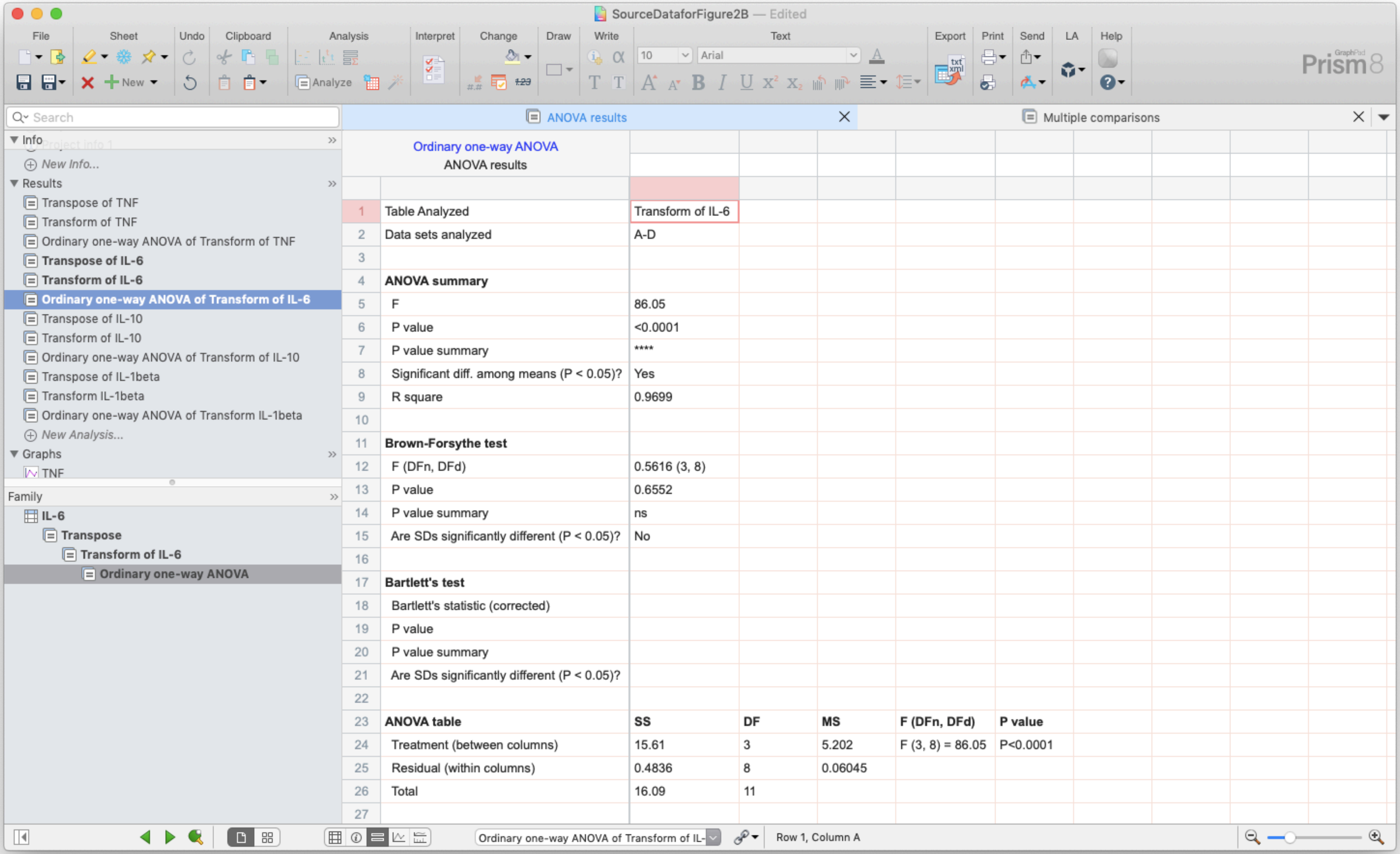

Ordinary one-way ANOVA
ANOVA results

1	Table Analyzed	Transform of IL-6				
2	Data sets analyzed	A-D				
3						
4	**ANOVA summary**					
5	F	86.05				
6	P value	<0.0001				
7	P value summary	****				
8	Significant diff. among means (P < 0.05)?	Yes				
9	R square	0.9699				
10						
11	**Brown-Forsythe test**					
12	F (DFn, DFd)	0.5616 (3, 8)				
13	P value	0.6552				
14	P value summary	ns				
15	Are SDs significantly different (P < 0.05)?	No				
16						
17	**Bartlett's test**					
18	Bartlett's statistic (corrected)					
19	P value					
20	P value summary					
21	Are SDs significantly different (P < 0.05)?					
22						
23	**ANOVA table**	**SS**	**DF**	**MS**	**F (DFn, DFd)**	**P value**
24	Treatment (between columns)	15.61	3	5.202	F (3, 8) = 86.05	P<0.0001
25	Residual (within columns)	0.4836	8	0.06045		
26	Total	16.09	11			
27						

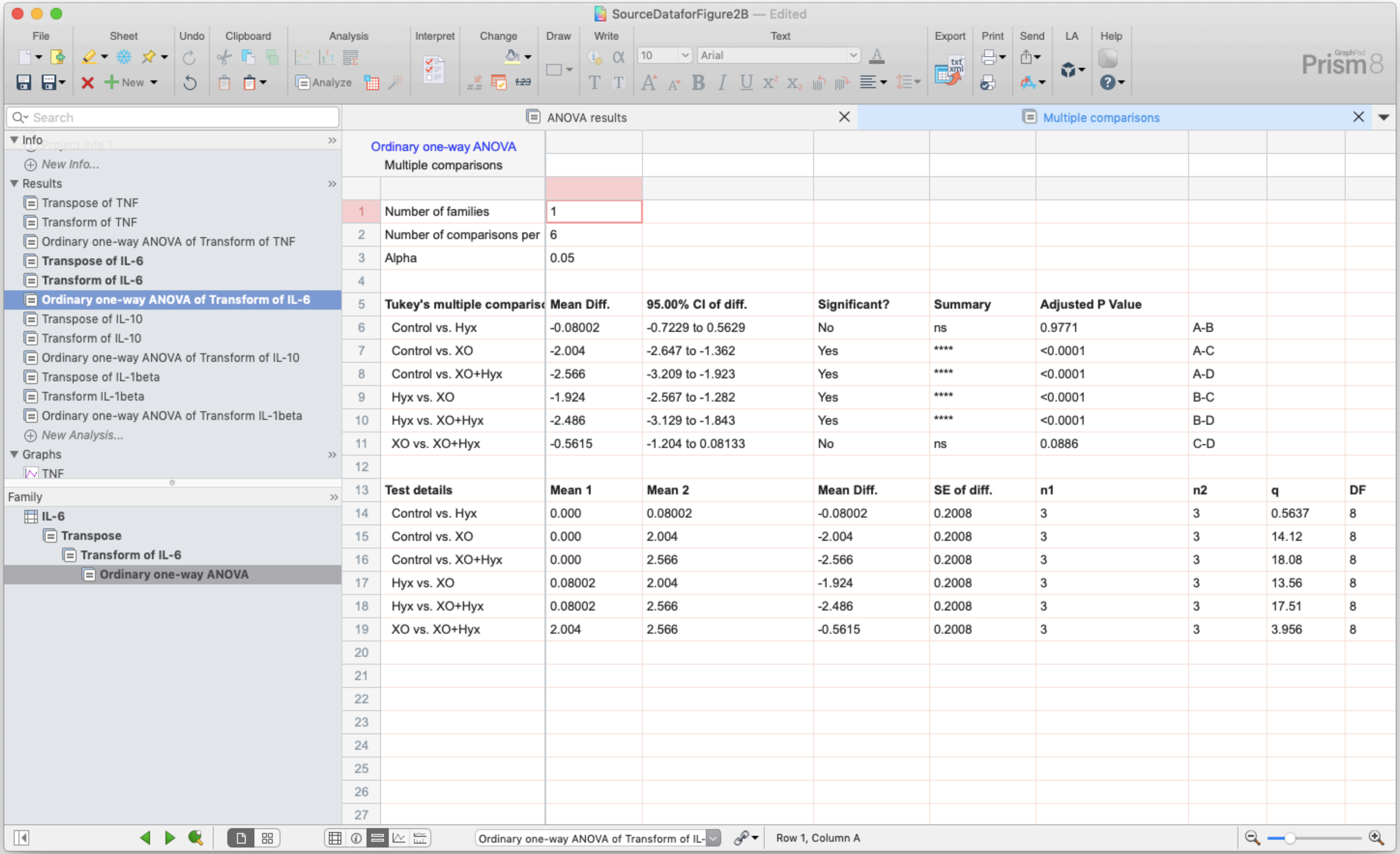

Ordinary one-way ANOVA
Multiple comparisons

1	Number of families	1							
2	Number of comparisons per	6							
3	Alpha	0.05							
4									
5	**Tukey's multiple comparis**	**Mean Diff.**	**95.00% CI of diff.**	**Significant?**	**Summary**	**Adjusted P Value**			
6	Control vs. Hyx	-0.08002	-0.7229 to 0.5629	No	ns	0.9771	A-B		
7	Control vs. XO	-2.004	-2.647 to -1.362	Yes	****	<0.0001	A-C		
8	Control vs. XO+Hyx	-2.566	-3.209 to -1.923	Yes	****	<0.0001	A-D		
9	Hyx vs. XO	-1.924	-2.567 to -1.282	Yes	****	<0.0001	B-C		
10	Hyx vs. XO+Hyx	-2.486	-3.129 to -1.843	Yes	****	<0.0001	B-D		
11	XO vs. XO+Hyx	-0.5615	-1.204 to 0.08133	No	ns	0.0886	C-D		
12									
13	**Test details**	**Mean 1**	**Mean 2**	**Mean Diff.**	**SE of diff.**	**n1**	**n2**	**q**	**DF**
14	Control vs. Hyx	0.000	0.08002	-0.08002	0.2008	3	3	0.5637	8
15	Control vs. XO	0.000	2.004	-2.004	0.2008	3	3	14.12	8
16	Control vs. XO+Hyx	0.000	2.566	-2.566	0.2008	3	3	18.08	8
17	Hyx vs. XO	0.08002	2.004	-1.924	0.2008	3	3	13.56	8
18	Hyx vs. XO+Hyx	0.08002	2.566	-2.486	0.2008	3	3	17.51	8
19	XO vs. XO+Hyx	2.004	2.566	-0.5615	0.2008	3	3	3.956	8

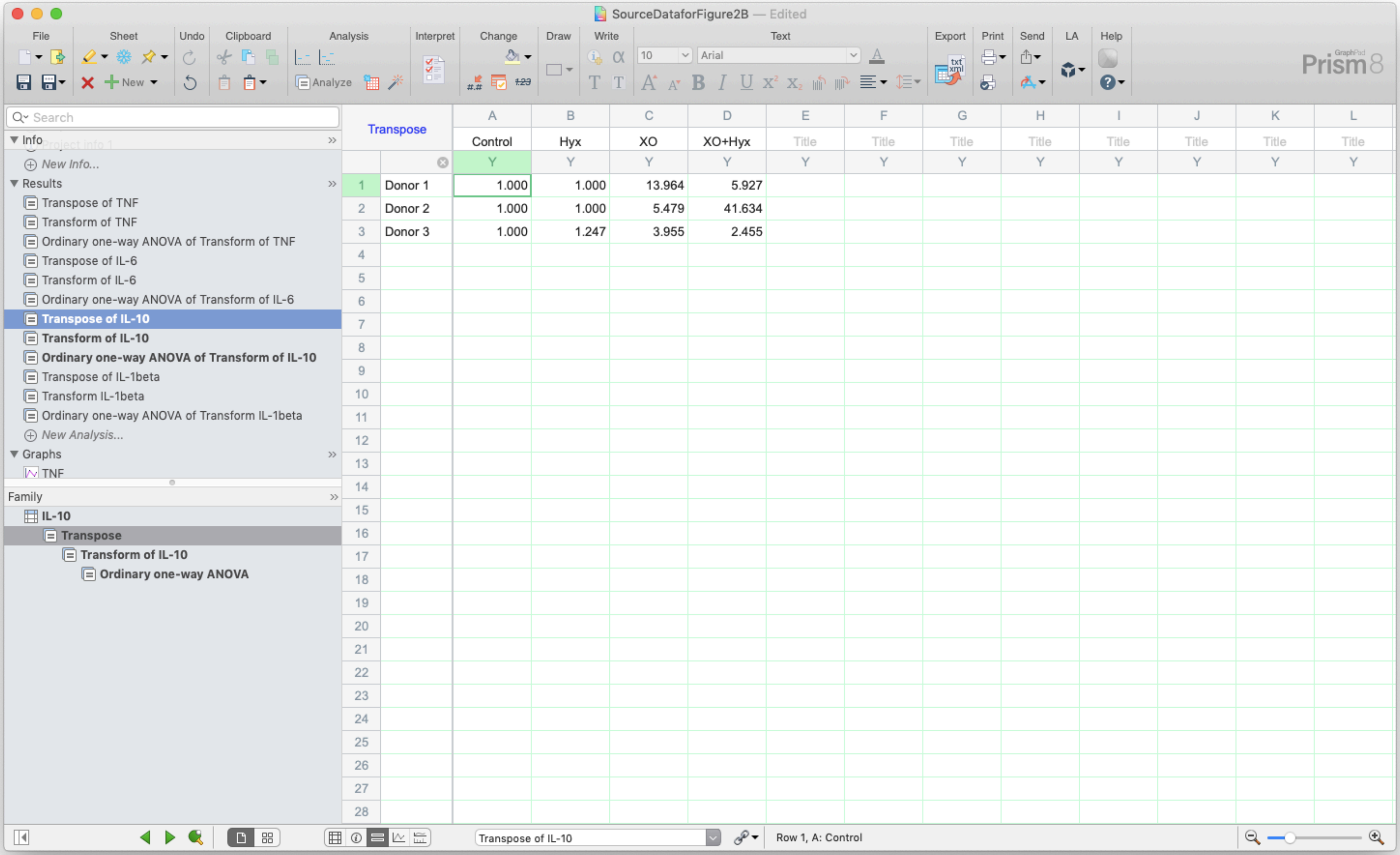

	Control	Hyx	XO	XO+Hyx
Donor 1	1.000	1.000	13.964	5.927
Donor 2	1.000	1.000	5.479	41.634
Donor 3	1.000	1.247	3.955	2.455

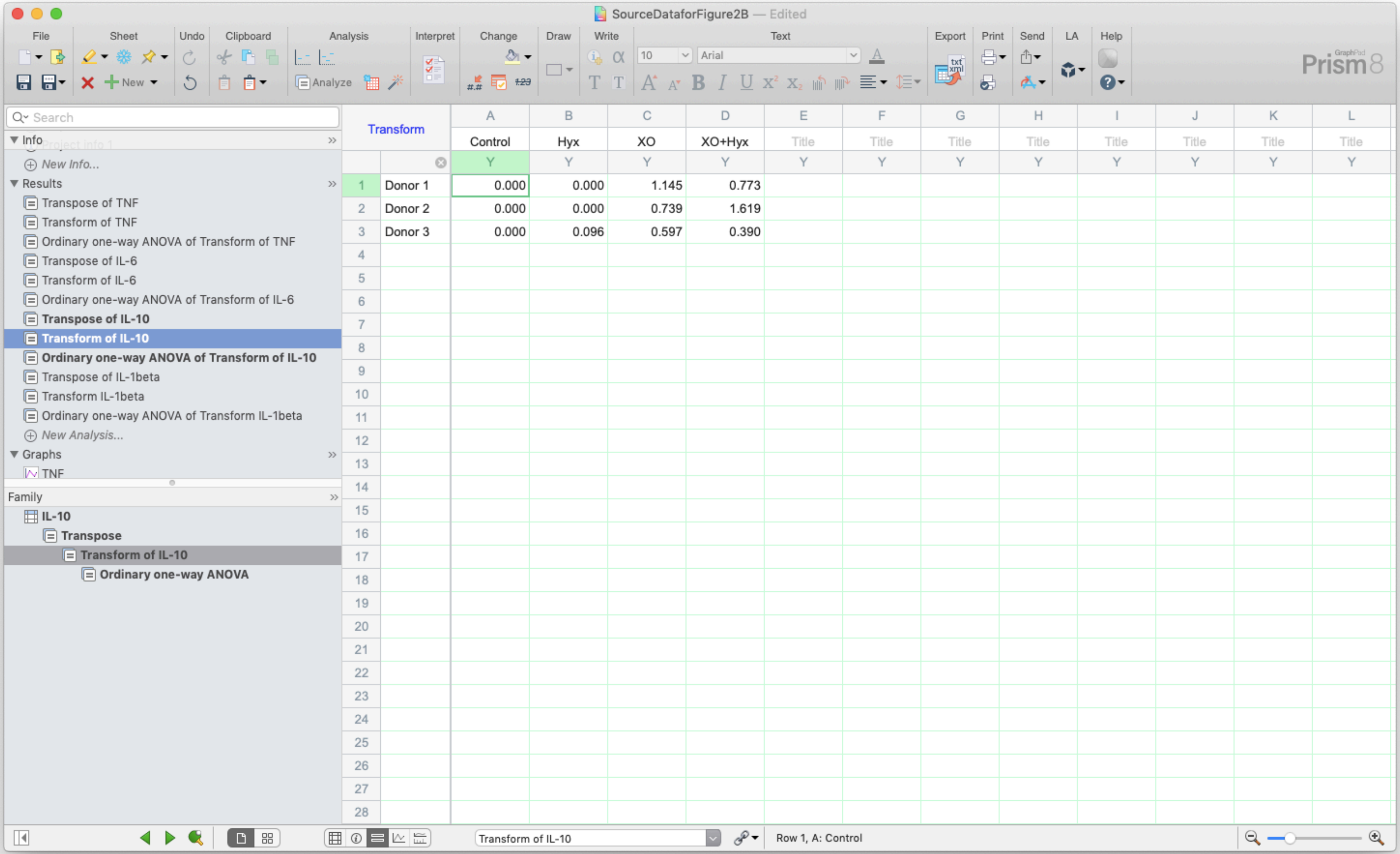

SourceDataforFigure2B — Edited
Transform
Info
New Info...
Results
Transpose of TNF
Transform of TNF
Ordinary one-way ANOVA of Transform of TNF
Transpose of IL-6
Transform of IL-6
Ordinary one-way ANOVA of Transform of IL-6
Transpose of IL-10
Transform of IL-10
Ordinary one-way ANOVA of Transform of IL-10
Transpose of IL-1beta
Transform IL-1beta
Ordinary one-way ANOVA of Transform IL-1beta
New Analysis...
Graphs
TNF
Family
IL-10
Transpose
Transform of IL-10
Ordinary one-way ANOVA
Control
Hyx
XO
XO+Hyx
Donor 1 0.000 0.000 1.145 0.773
Donor 2 0.000 0.000 0.739 1.619
Donor 3 0.000 0.096 0.597 0.390
Row 1, A: Control

SourceDataforFigure2B — Edited

ANOVA results | Multiple comparisons

Ordinary one-way ANOVA
ANOVA results

		A				
1	Table Analyzed	Transform of IL-10				
2	Data sets analyzed	A-D				
3						
4	**ANOVA summary**					
5	F	6.230				
6	P value	0.0173				
7	P value summary	*				
8	Significant diff. among means (P < 0.05)?	Yes				
9	R square	0.7003				
10						
11	**Brown-Forsythe test**					
12	F (DFn, DFd)	1.862 (3, 8)				
13	P value	0.2144				
14	P value summary	ns				
15	Are SDs significantly different (P < 0.05)?	No				
16						
17	**Bartlett's test**					
18	Bartlett's statistic (corrected)					
19	P value					
20	P value summary					
21	Are SDs significantly different (P < 0.05)?					
22						
23	**ANOVA table**	**SS**	**DF**	**MS**	**F (DFn, DFd)**	**P value**
24	Treatment (between columns)	2.242	3	0.7472	F (3, 8) = 6.230	P=0.0173
25	Residual (within columns)	0.9595	8	0.1199		
26	Total	3.201	11			
27						

Ordinary one-way ANOVA of Transform of IL-10 — Row 1, Column A

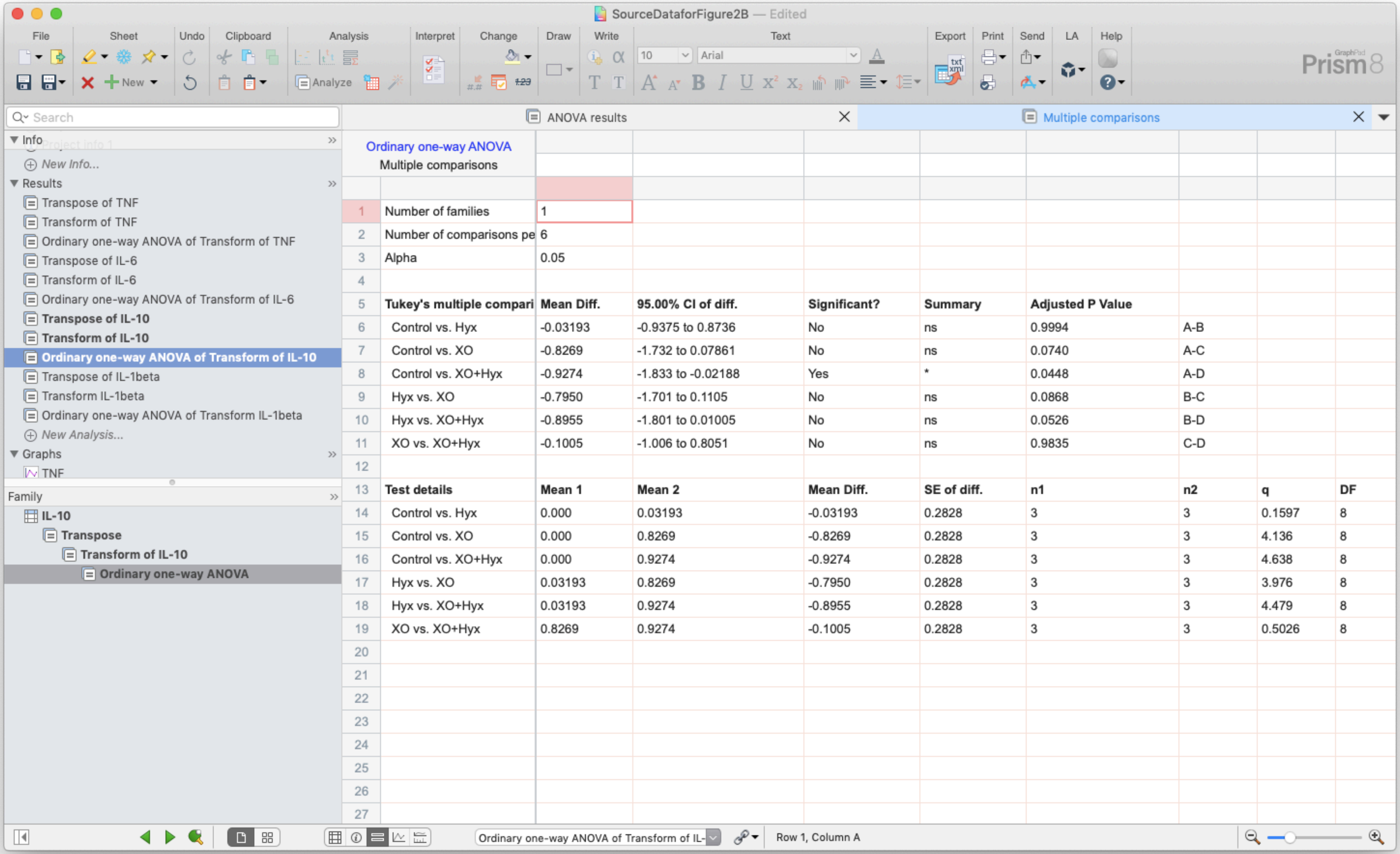

	Ordinary one-way ANOVA<br>Multiple comparisons								
1	Number of families	1							
2	Number of comparisons pe	6							
3	Alpha	0.05							
4									
5	**Tukey's multiple compari**	**Mean Diff.**	**95.00% CI of diff.**	**Significant?**	**Summary**	**Adjusted P Value**			
6	Control vs. Hyx	-0.03193	-0.9375 to 0.8736	No	ns	0.9994	A-B		
7	Control vs. XO	-0.8269	-1.732 to 0.07861	No	ns	0.0740	A-C		
8	Control vs. XO+Hyx	-0.9274	-1.833 to -0.02188	Yes	*	0.0448	A-D		
9	Hyx vs. XO	-0.7950	-1.701 to 0.1105	No	ns	0.0868	B-C		
10	Hyx vs. XO+Hyx	-0.8955	-1.801 to 0.01005	No	ns	0.0526	B-D		
11	XO vs. XO+Hyx	-0.1005	-1.006 to 0.8051	No	ns	0.9835	C-D		
12									
13	**Test details**	**Mean 1**	**Mean 2**	**Mean Diff.**	**SE of diff.**	**n1**	**n2**	**q**	**DF**
14	Control vs. Hyx	0.000	0.03193	-0.03193	0.2828	3	3	0.1597	8
15	Control vs. XO	0.000	0.8269	-0.8269	0.2828	3	3	4.136	8
16	Control vs. XO+Hyx	0.000	0.9274	-0.9274	0.2828	3	3	4.638	8
17	Hyx vs. XO	0.03193	0.8269	-0.7950	0.2828	3	3	3.976	8
18	Hyx vs. XO+Hyx	0.03193	0.9274	-0.8955	0.2828	3	3	4.479	8
19	XO vs. XO+Hyx	0.8269	0.9274	-0.1005	0.2828	3	3	0.5026	8

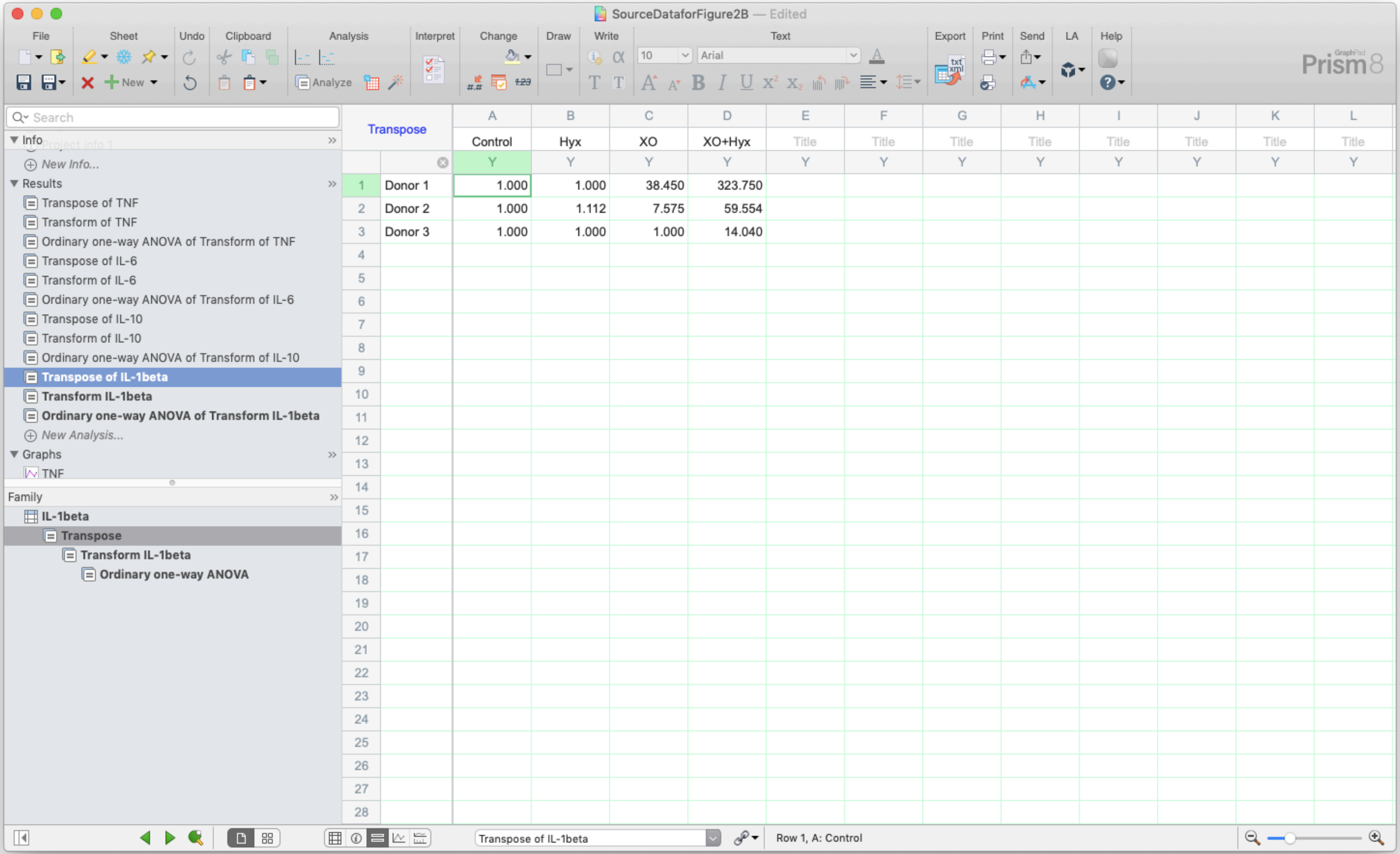

	Transpose	Control	Hyx	XO	XO+Hyx
1	Donor 1	1.000	1.000	38.450	323.750
2	Donor 2	1.000	1.112	7.575	59.554
3	Donor 3	1.000	1.000	1.000	14.040

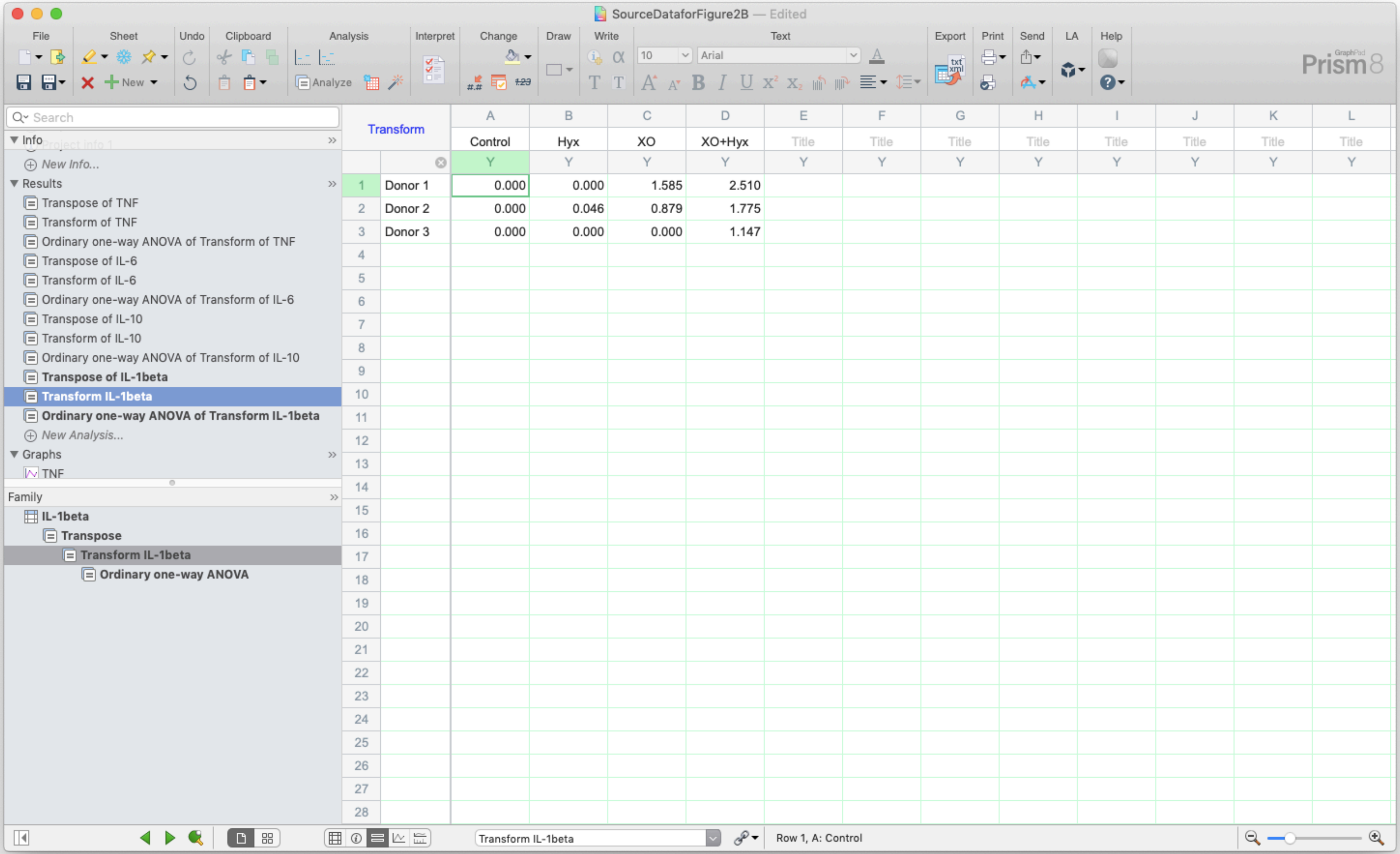

Transform		Control	Hyx	XO	XO+Hyx
		Y	Y	Y	Y
1	Donor 1	0.000	0.000	1.585	2.510
2	Donor 2	0.000	0.046	0.879	1.775
3	Donor 3	0.000	0.000	0.000	1.147

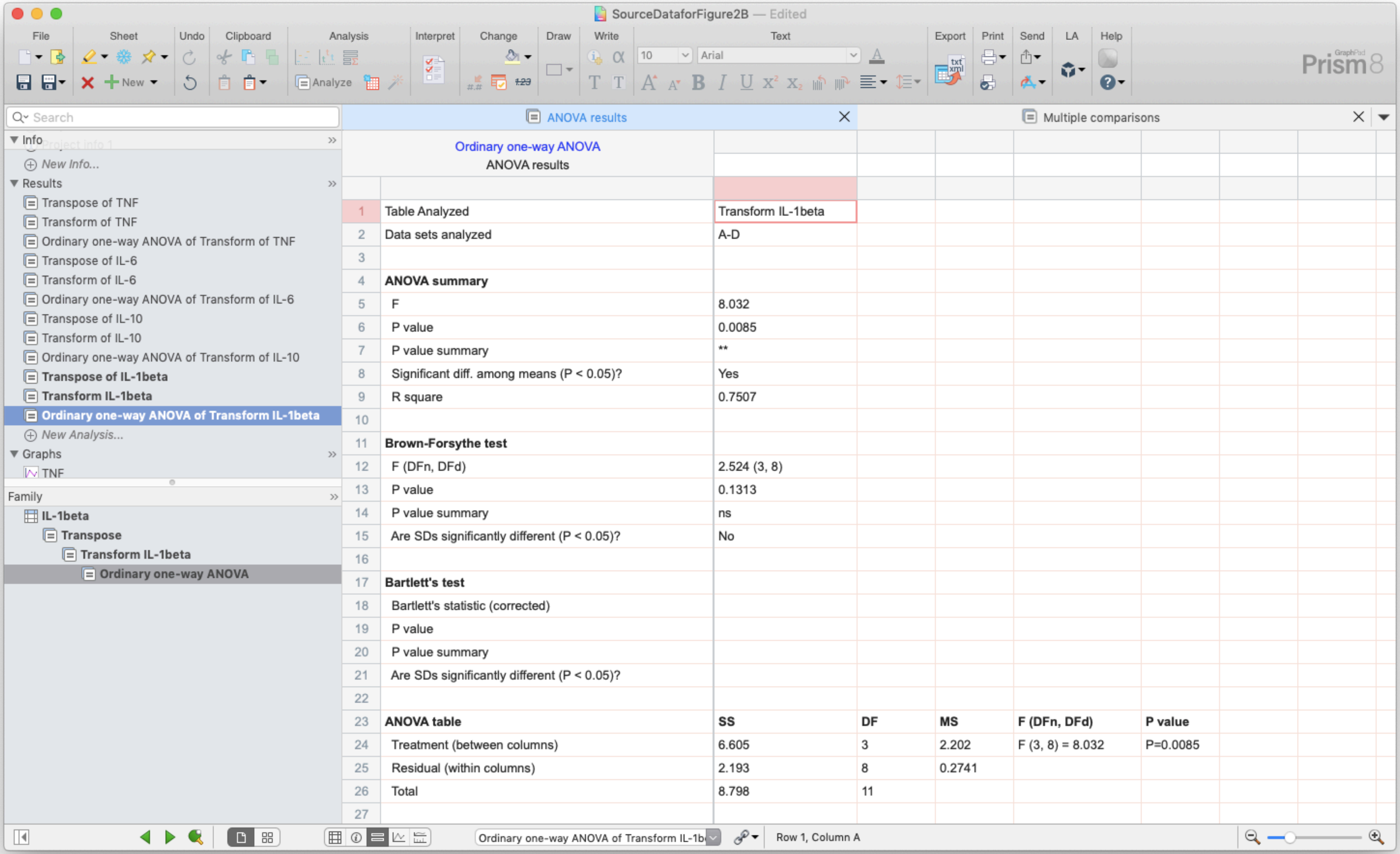

Ordinary one-way ANOVA
ANOVA results

1	Table Analyzed	Transform IL-1beta				
2	Data sets analyzed	A-D				
3						
4	**ANOVA summary**					
5	F	8.032				
6	P value	0.0085				
7	P value summary	**				
8	Significant diff. among means (P < 0.05)?	Yes				
9	R square	0.7507				
10						
11	**Brown-Forsythe test**					
12	F (DFn, DFd)	2.524 (3, 8)				
13	P value	0.1313				
14	P value summary	ns				
15	Are SDs significantly different (P < 0.05)?	No				
16						
17	**Bartlett's test**					
18	Bartlett's statistic (corrected)					
19	P value					
20	P value summary					
21	Are SDs significantly different (P < 0.05)?					
22						
23	**ANOVA table**	**SS**	**DF**	**MS**	**F (DFn, DFd)**	**P value**
24	Treatment (between columns)	6.605	3	2.202	F (3, 8) = 8.032	P=0.0085
25	Residual (within columns)	2.193	8	0.2741		
26	Total	8.798	11			
27						

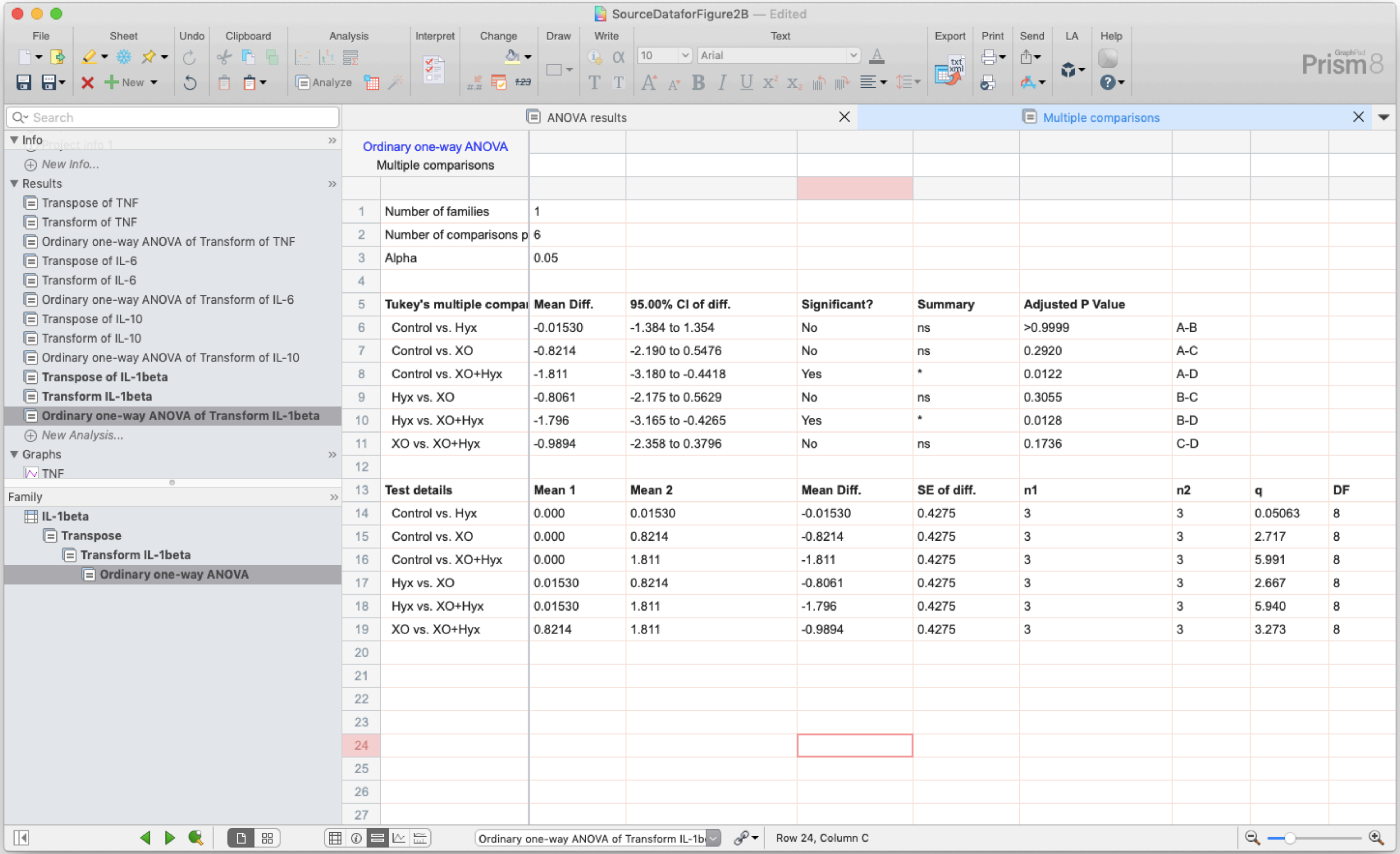

Ordinary one-way ANOVA
Multiple comparisons

1	Number of families	1							
2	Number of comparisons p	6							
3	Alpha	0.05							
4									
5	**Tukey's multiple compai**	**Mean Diff.**	**95.00% CI of diff.**	**Significant?**	**Summary**	**Adjusted P Value**			
6	Control vs. Hyx	-0.01530	-1.384 to 1.354	No	ns	>0.9999	A-B		
7	Control vs. XO	-0.8214	-2.190 to 0.5476	No	ns	0.2920	A-C		
8	Control vs. XO+Hyx	-1.811	-3.180 to -0.4418	Yes	*	0.0122	A-D		
9	Hyx vs. XO	-0.8061	-2.175 to 0.5629	No	ns	0.3055	B-C		
10	Hyx vs. XO+Hyx	-1.796	-3.165 to -0.4265	Yes	*	0.0128	B-D		
11	XO vs. XO+Hyx	-0.9894	-2.358 to 0.3796	No	ns	0.1736	C-D		
12									
13	**Test details**	**Mean 1**	**Mean 2**	**Mean Diff.**	**SE of diff.**	**n1**	**n2**	**q**	**DF**
14	Control vs. Hyx	0.000	0.01530	-0.01530	0.4275	3	3	0.05063	8
15	Control vs. XO	0.000	0.8214	-0.8214	0.4275	3	3	2.717	8
16	Control vs. XO+Hyx	0.000	1.811	-1.811	0.4275	3	3	5.991	8
17	Hyx vs. XO	0.01530	0.8214	-0.8061	0.4275	3	3	2.667	8
18	Hyx vs. XO+Hyx	0.01530	1.811	-1.796	0.4275	3	3	5.940	8
19	XO vs. XO+Hyx	0.8214	1.811	-0.9894	0.4275	3	3	3.273	8
20									
21									
22									
23									
24									
25									
26									
27									

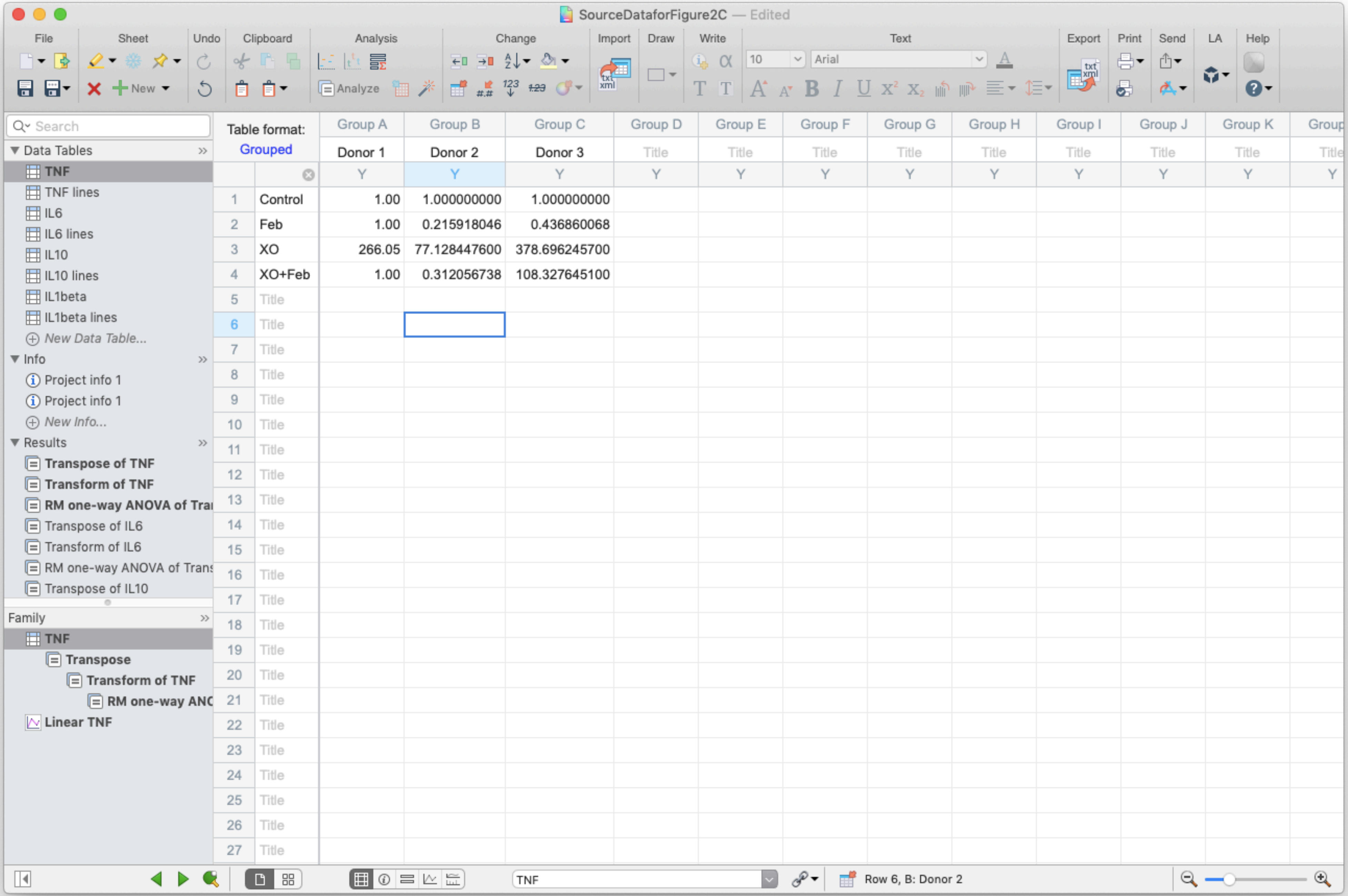

Table format: Grouped	Group A	Group B	Group C
	Donor 1	Donor 2	Donor 3
	Y	Y	Y
Control	1.00	1.000000000	1.000000000
Feb	1.00	0.215918046	0.436860068
XO	266.05	77.128447600	378.696245700
XO+Feb	1.00	0.312056738	108.327645100

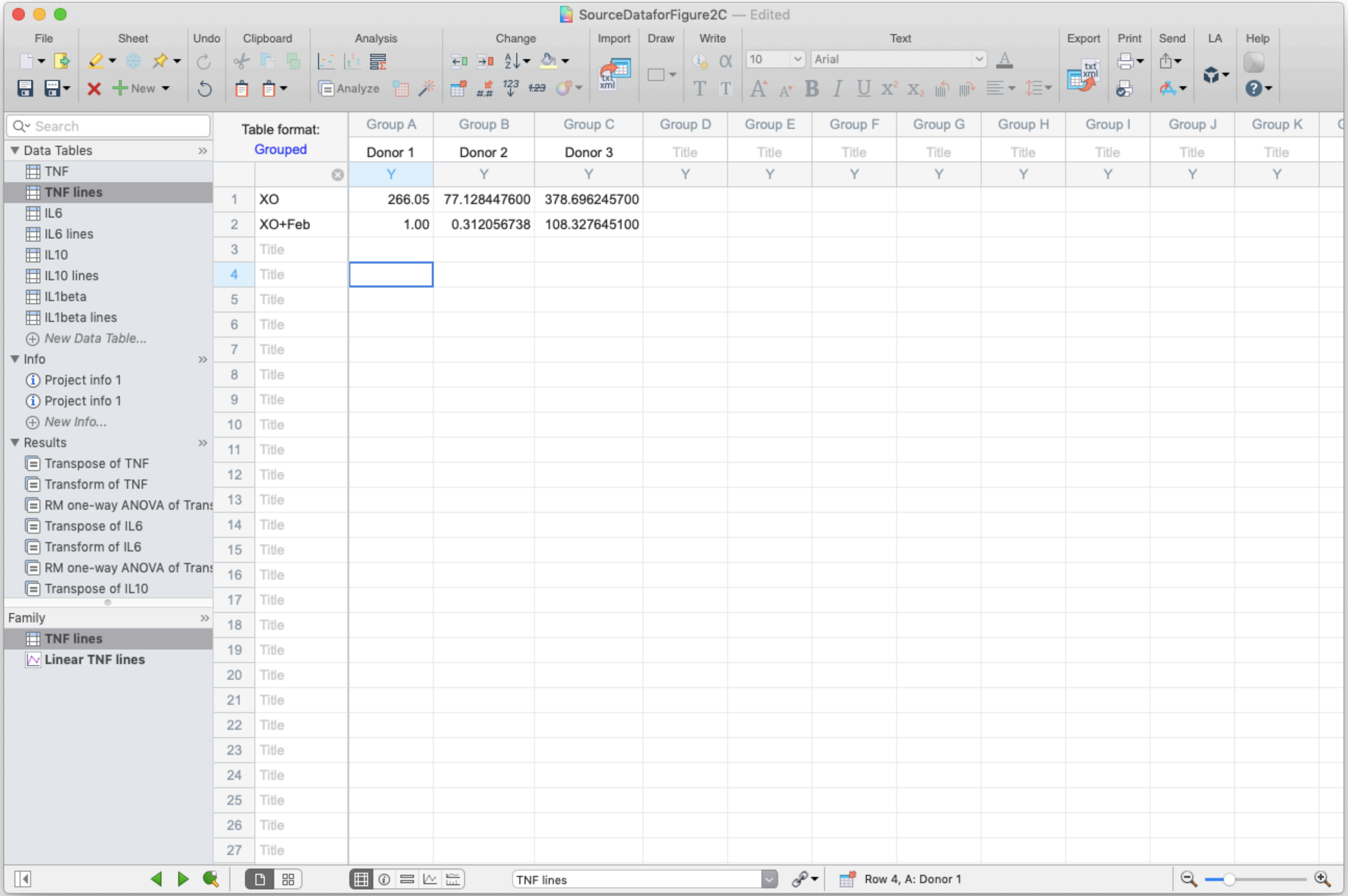

Table format: Grouped	Group A	Group B	Group C
	Donor 1	Donor 2	Donor 3
	Y	Y	Y
XO	266.05	77.128447600	378.696245700
XO+Feb	1.00	0.312056738	108.327645100

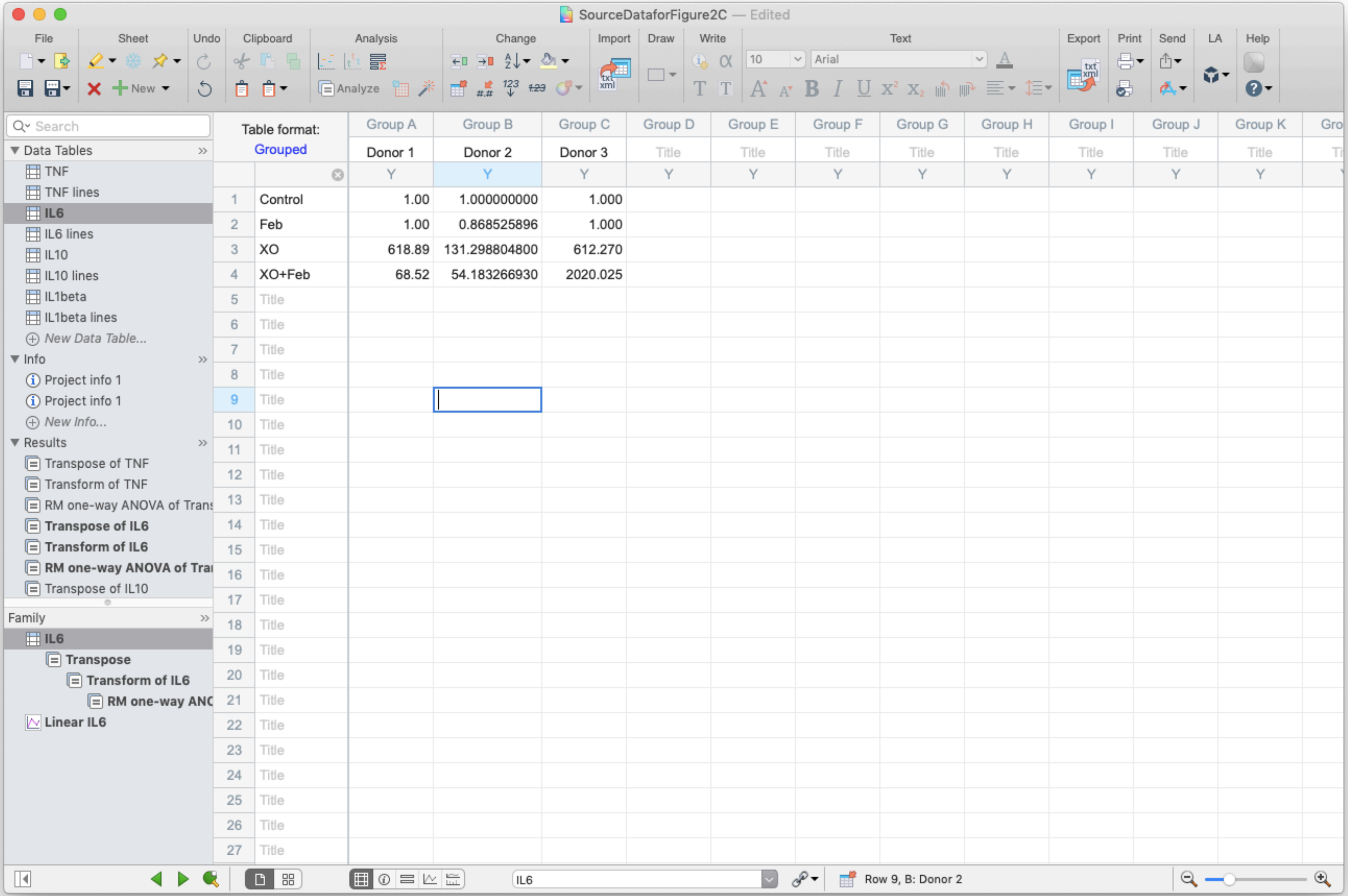

		Group A	Group B	Group C
		Donor 1	Donor 2	Donor 3
		Y	Y	Y
1	Control	1.00	1.000000000	1.000
2	Feb	1.00	0.868525896	1.000
3	XO	618.89	131.298804800	612.270
4	XO+Feb	68.52	54.183266930	2020.025

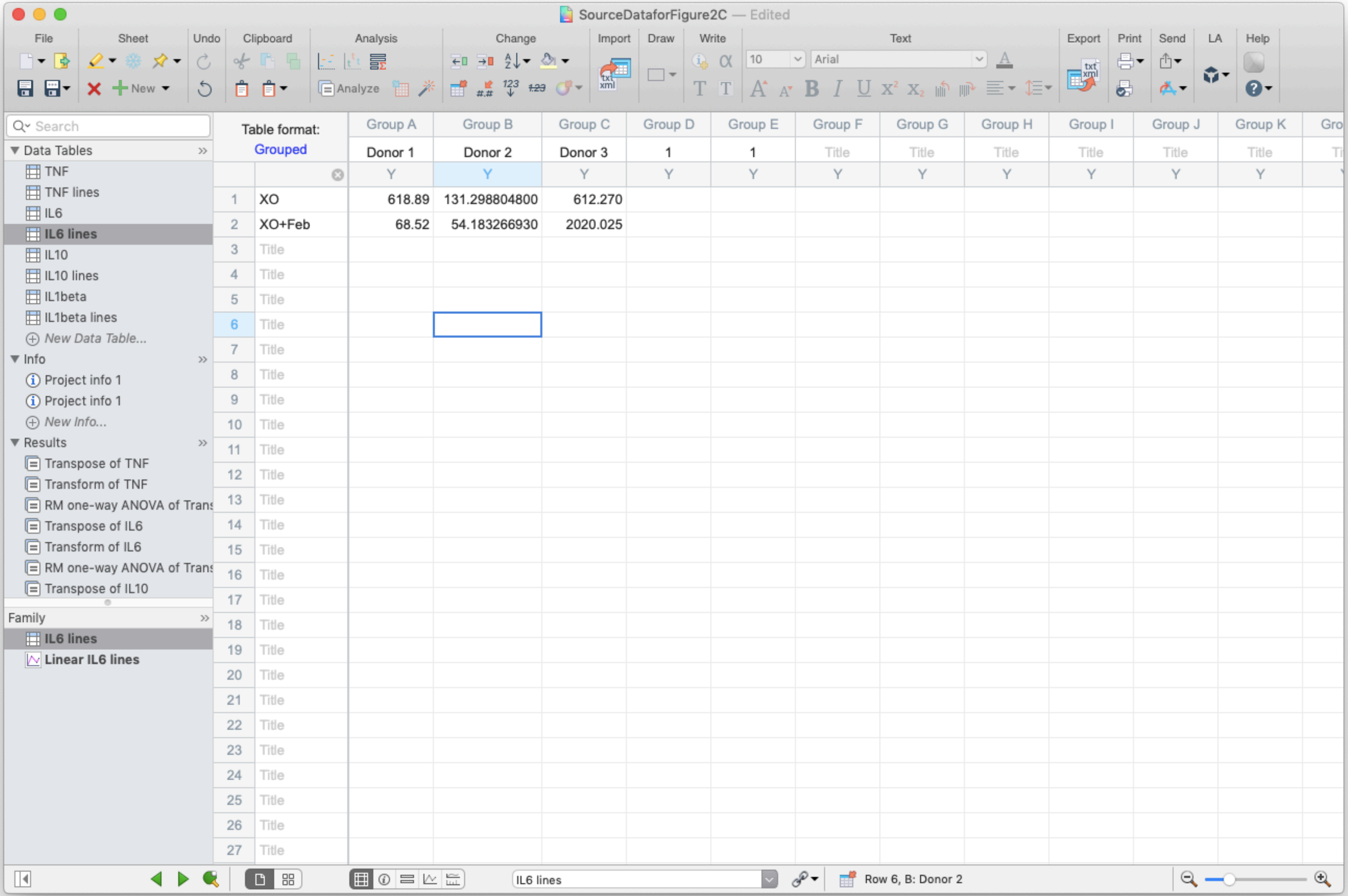
SourceDataforFigure2C — Edited
File
Sheet
Undo
Clipboard
Analysis
Change
Import
Draw
Write
Text
Export
Print
Send
LA
Help
New
Analyze
10
Arial
Search
Data Tables
TNF
TNF lines
IL6
IL6 lines
IL10
IL10 lines
IL1beta
IL1beta lines
New Data Table...
Info
Project info 1
Project info 1
New Info...
Results
Transpose of TNF
Transform of TNF
RM one-way ANOVA of Trans
Transpose of IL6
Transform of IL6
RM one-way ANOVA of Trans
Transpose of IL10
Family
IL6 lines
Linear IL6 lines
Table format:
Grouped
Group A
Group B
Group C
Group D
Group E
Group F
Group G
Group H
Group I
Group J
Group K
Donor 1
Donor 2
Donor 3
1
1
XO
618.89
131.298804800
612.270
XO+Feb
68.52
54.183266930
2020.025
IL6 lines
Row 6, B: Donor 2

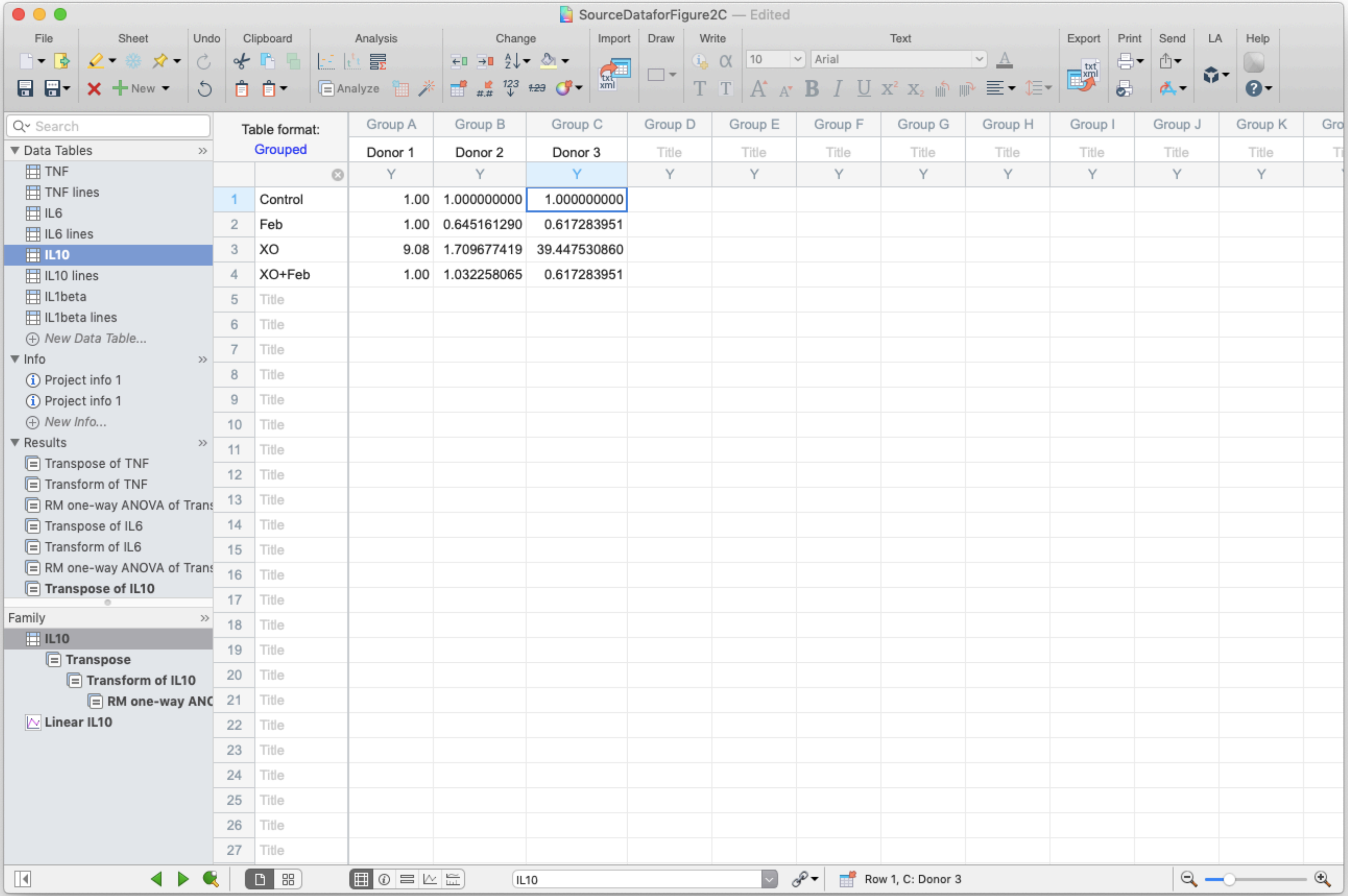

	Group A	Group B	Group C
	Donor 1	Donor 2	Donor 3
	Y	Y	Y
1 Control	1.00	1.000000000	1.000000000
2 Feb	1.00	0.645161290	0.617283951
3 XO	9.08	1.709677419	39.447530860
4 XO+Feb	1.00	1.032258065	0.617283951

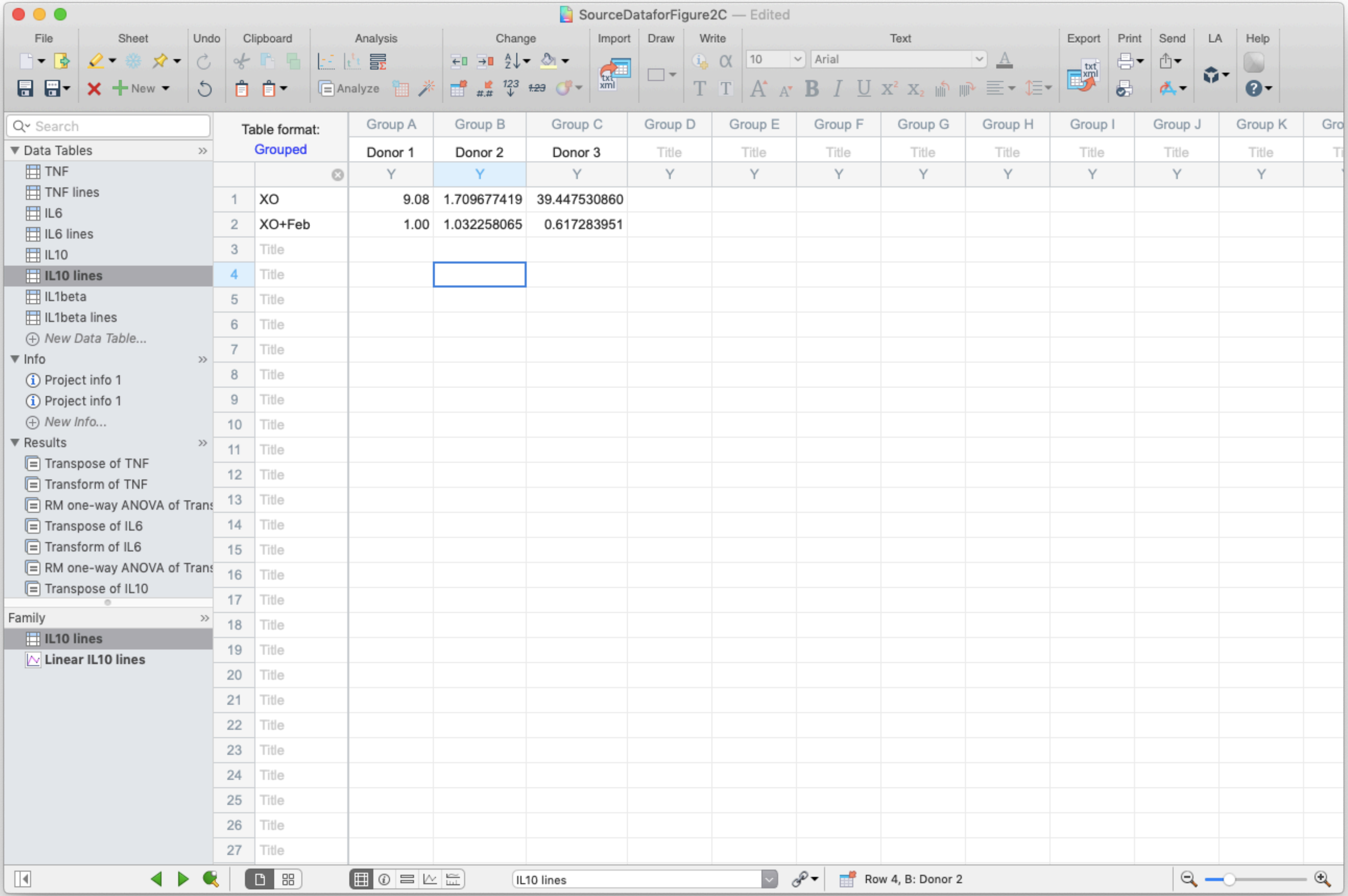
SourceDataforFigure2C — Edited
File
Sheet
Undo
Clipboard
Analysis
Change
Import
Draw
Write
Text
Export
Print
Send
LA
Help
New
Analyze
Arial
10
Search
Data Tables
TNF
TNF lines
IL6
IL6 lines
IL10
IL10 lines
IL1beta
IL1beta lines
New Data Table...
Info
Project info 1
Project info 1
New Info...
Results
Transpose of TNF
Transform of TNF
RM one-way ANOVA of Trans
Transpose of IL6
Transform of IL6
RM one-way ANOVA of Trans
Transpose of IL10
Family
IL10 lines
Linear IL10 lines
Table format:
Grouped
Group A
Group B
Group C
Group D
Group E
Group F
Group G
Group H
Group I
Group J
Group K
Donor 1
Donor 2
Donor 3
1 XO 9.08 1.709677419 39.447530860
2 XO+Feb 1.00 1.032258065 0.617283951
IL10 lines
Row 4, B: Donor 2

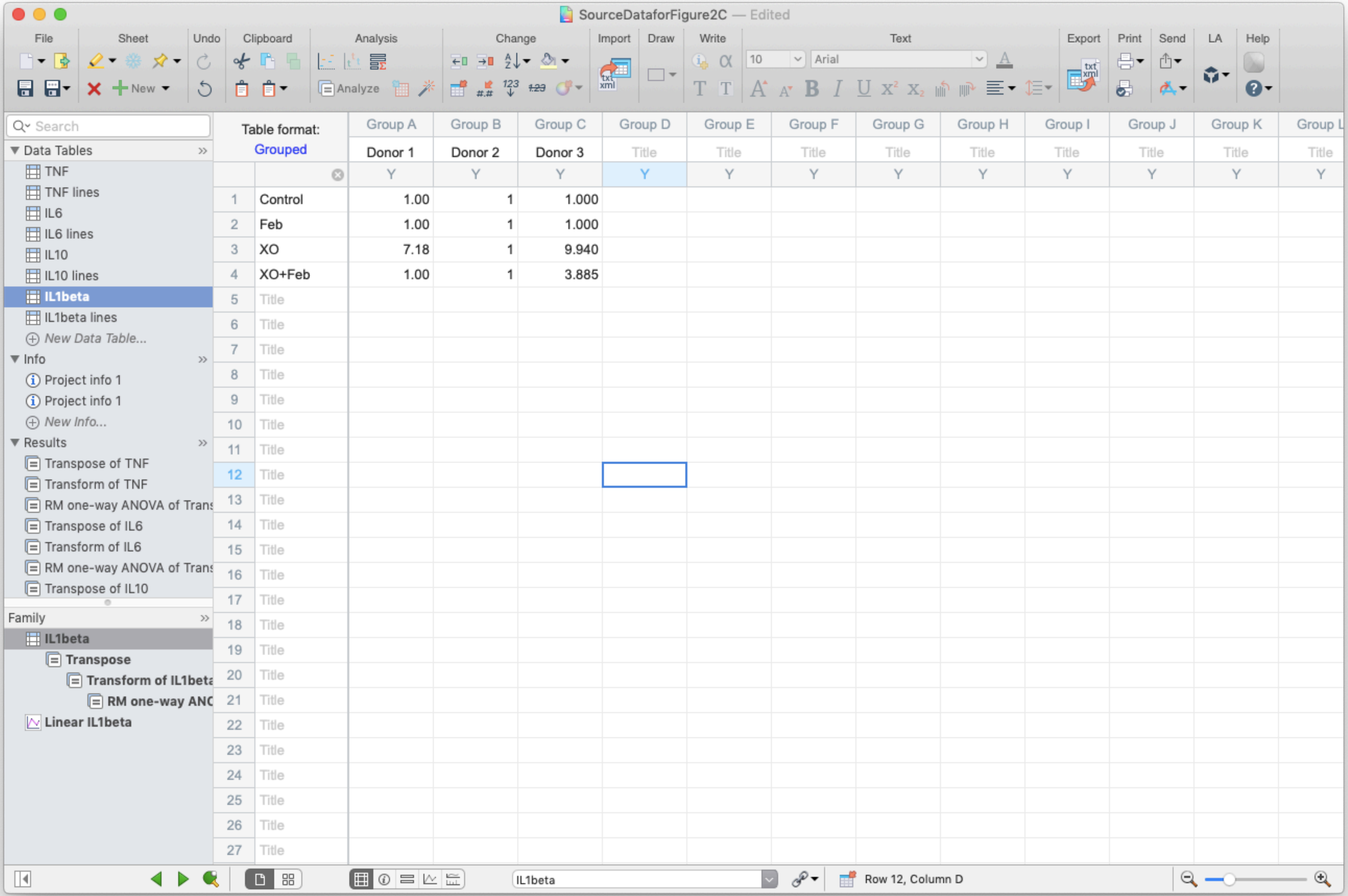

	Table format: Grouped	Group A Donor 1	Group B Donor 2	Group C Donor 3
1	Control	1.00	1	1.000
2	Feb	1.00	1	1.000
3	XO	7.18	1	9.940
4	XO+Feb	1.00	1	3.885

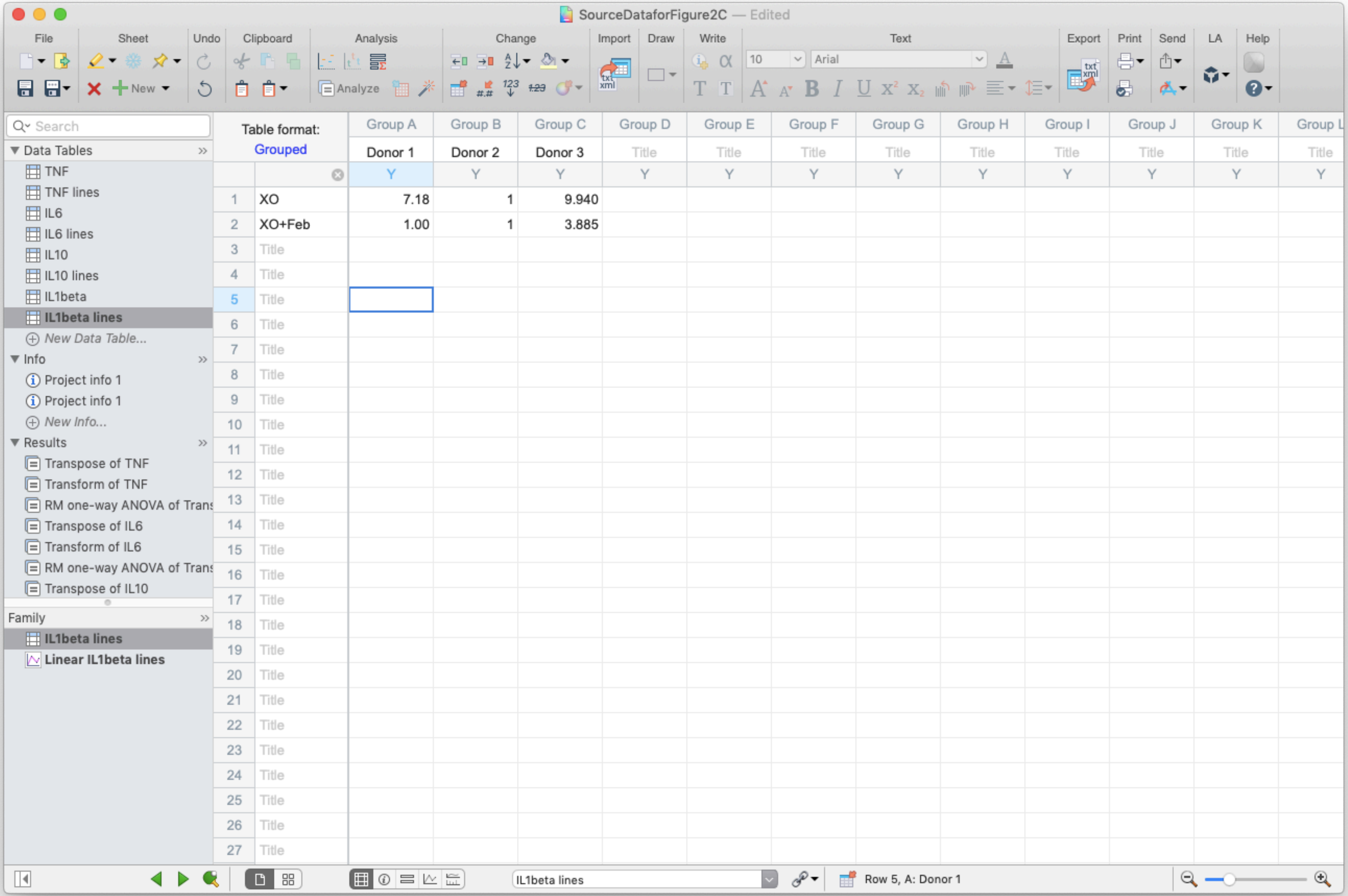

Table format: Grouped	Group A	Group B	Group C
	Donor 1	Donor 2	Donor 3
	Y	Y	Y
1 XO	7.18	1	9.940
2 XO+Feb	1.00	1	3.885

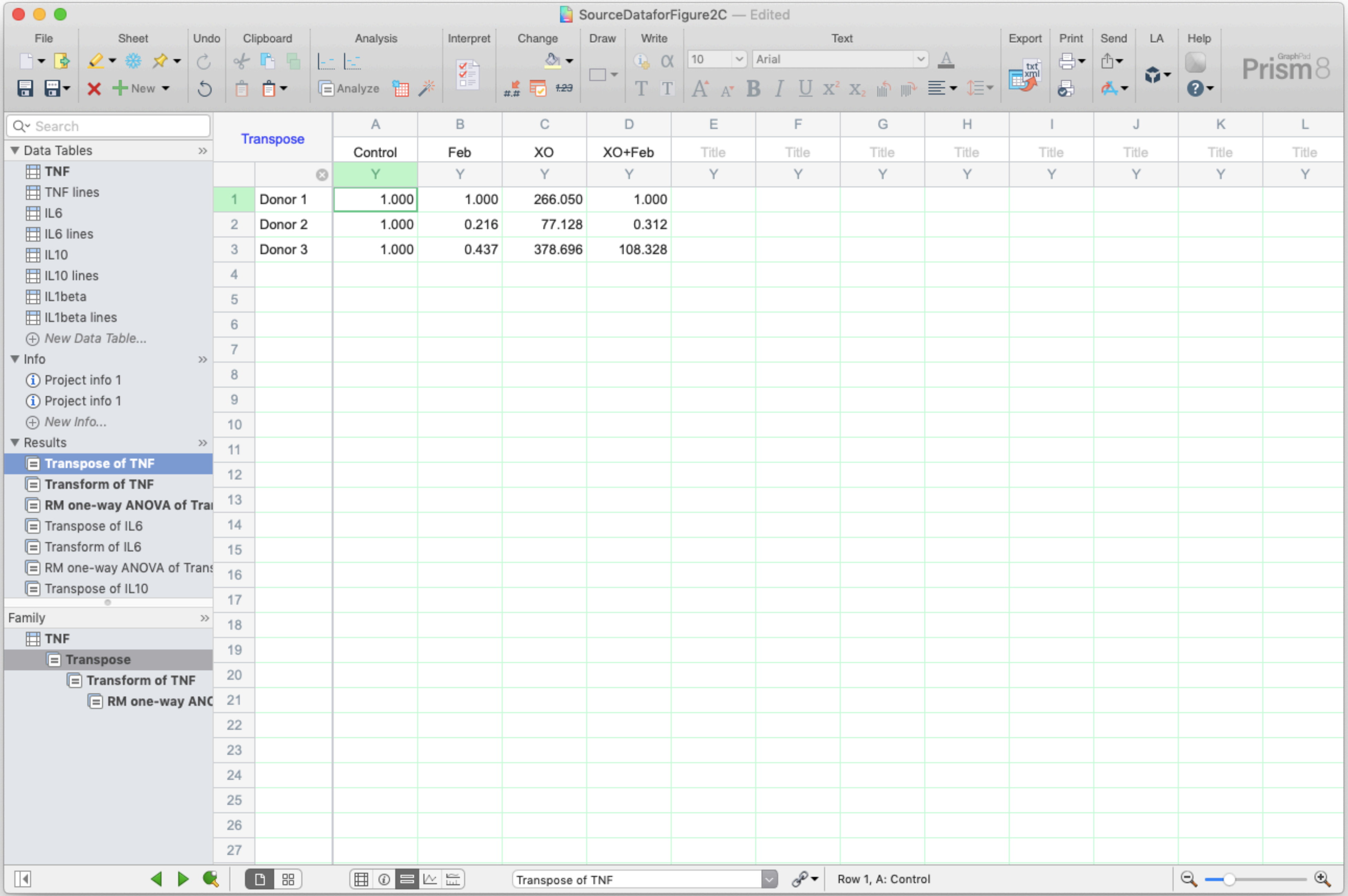

Transpose	Control	Feb	XO	XO+Feb
Donor 1	1.000	1.000	266.050	1.000
Donor 2	1.000	0.216	77.128	0.312
Donor 3	1.000	0.437	378.696	108.328

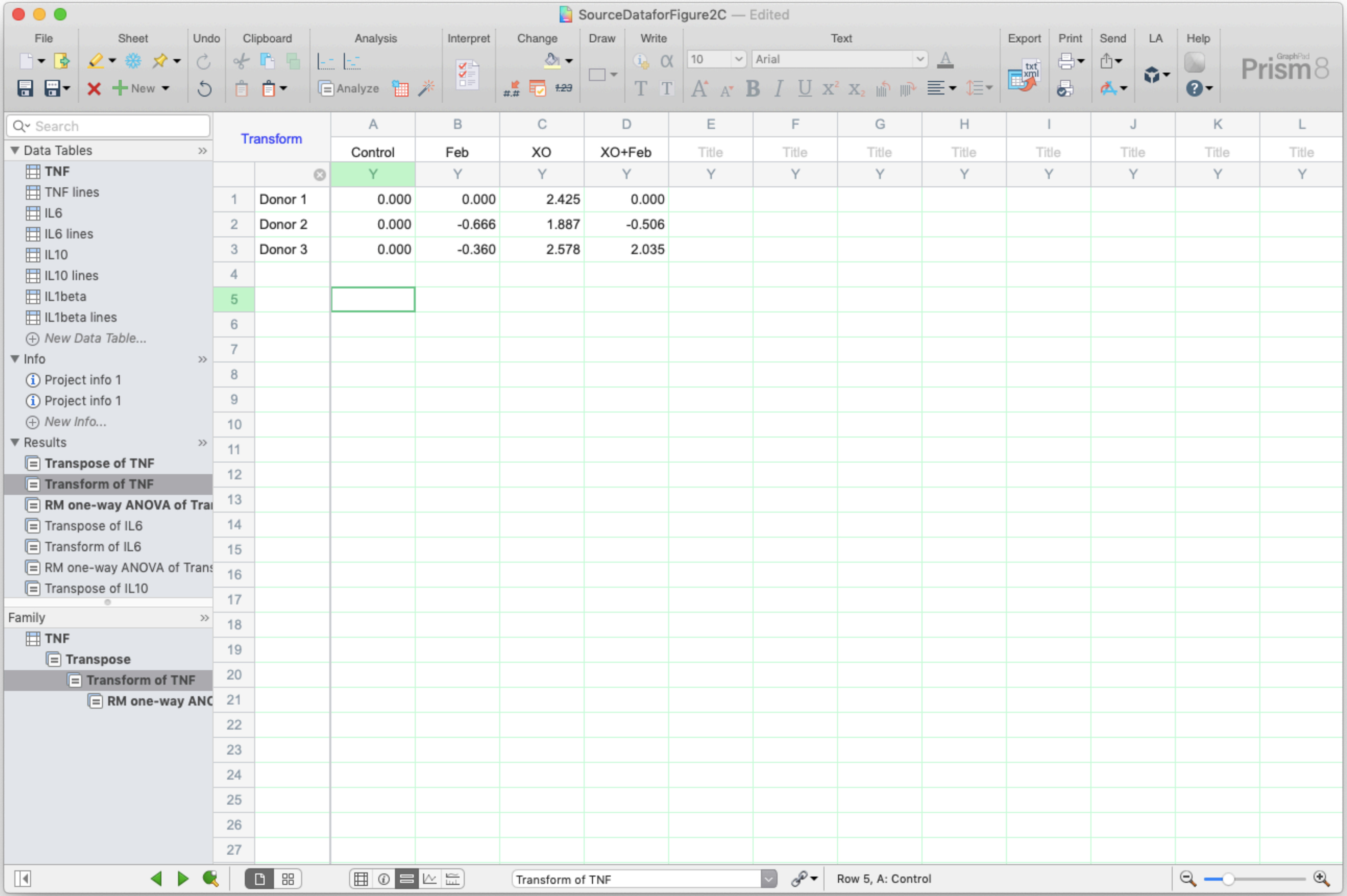

	Transform	Control	Feb	XO	XO+Feb
		Y	Y	Y	Y
1	Donor 1	0.000	0.000	2.425	0.000
2	Donor 2	0.000	-0.666	1.887	-0.506
3	Donor 3	0.000	-0.360	2.578	2.035

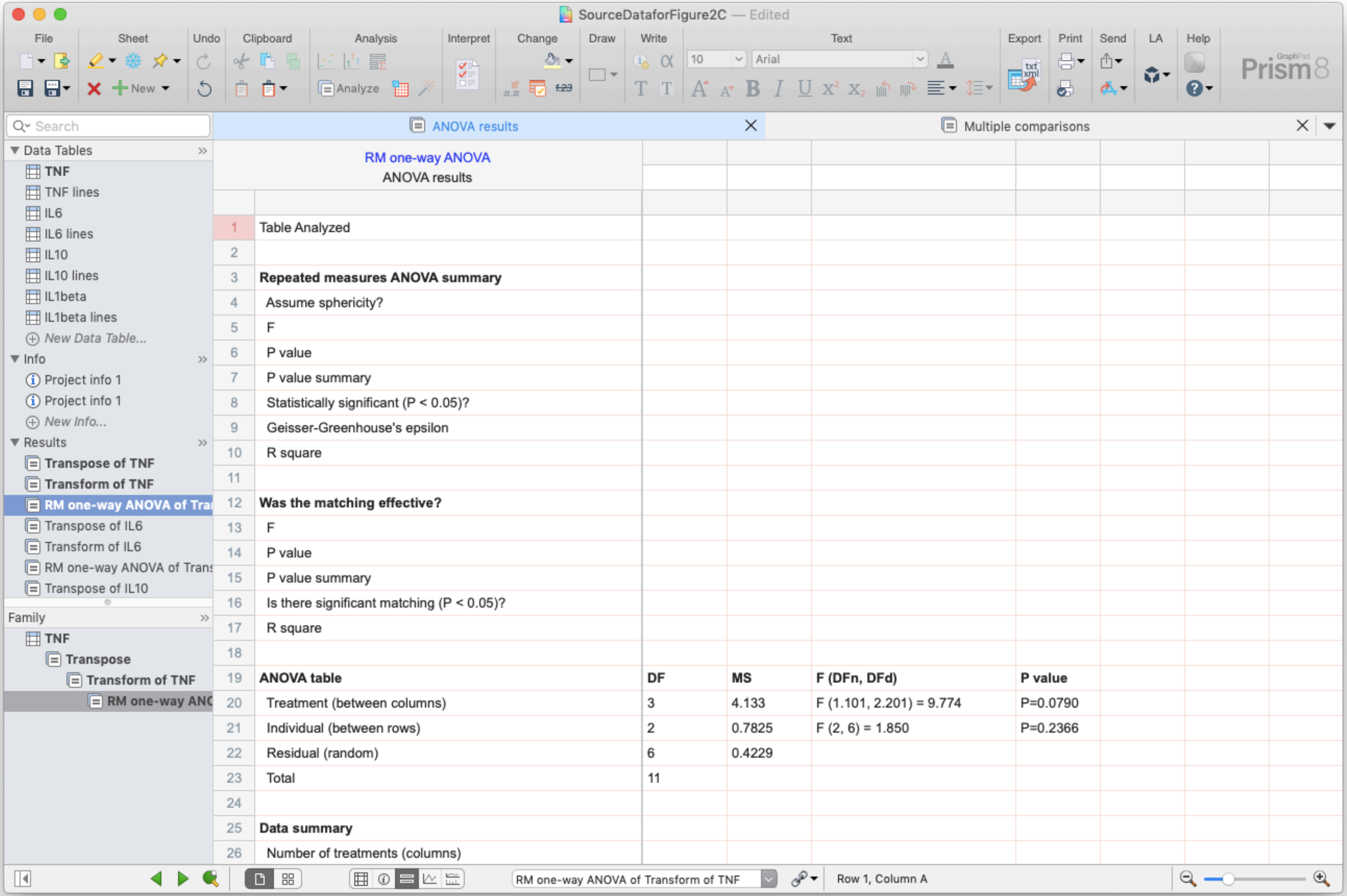

		DF	MS	F (DFn, DFd)	P value
1	Table Analyzed				
2					
3	**Repeated measures ANOVA summary**				
4	Assume sphericity?				
5	F				
6	P value				
7	P value summary				
8	Statistically significant (P < 0.05)?				
9	Geisser-Greenhouse's epsilon				
10	R square				
11					
12	**Was the matching effective?**				
13	F				
14	P value				
15	P value summary				
16	Is there significant matching (P < 0.05)?				
17	R square				
18					
19	**ANOVA table**	**DF**	**MS**	**F (DFn, DFd)**	**P value**
20	Treatment (between columns)	3	4.133	F (1.101, 2.201) = 9.774	P=0.0790
21	Individual (between rows)	2	0.7825	F (2, 6) = 1.850	P=0.2366
22	Residual (random)	6	0.4229		
23	Total	11			
24					
25	**Data summary**				
26	Number of treatments (columns)				

SourceDataforFigure2C — Edited

RM one-way ANOVA
Multiple comparisons

1	Number of families	1							
2	Number of comparisons p	6							
3	Alpha	0.05							
4									
5	**Tukey's multiple compa**	**Mean Diff.**	**95.00% CI of diff.**	**Significant?**	**Summary**	**Adjusted P Value**			
6	Control vs. Feb	0.3418	-0.9912 to 1.675	No	ns	0.4695	A-B		
7	Control vs. XO	-2.297	-3.749 to -0.8450	Yes	*	0.0205	A-C		
8	Control vs. XO+Feb	-0.5097	-5.889 to 4.870	No	ns	0.9054	A-D		
9	Feb vs. XO	-2.639	-3.707 to -1.570	Yes	**	0.0088	B-C		
10	Feb vs. XO+Feb	-0.8514	-6.207 to 4.504	No	ns	0.7244	B-D		
11	XO vs. XO+Feb	1.787	-2.522 to 6.096	No	ns	0.2409	C-D		
12									
13	**Test details**	**Mean 1**	**Mean 2**	**Mean Diff.**	**SE of diff.**	**n1**	**n2**	**q**	**DF**
14	Control vs. Feb	0.000	-0.3418	0.3418	0.1924	3	3	2.513	2
15	Control vs. XO	0.000	2.297	-2.297	0.2095	3	3	15.50	2
16	Control vs. XO+Feb	0.000	0.5097	-0.5097	0.7764	3	3	0.9284	2
17	Feb vs. XO	-0.3418	2.297	-2.639	0.1542	3	3	24.21	2
18	Feb vs. XO+Feb	-0.3418	0.5097	-0.8514	0.7729	3	3	1.558	2
19	XO vs. XO+Feb	2.297	0.5097	1.787	0.6219	3	3	4.064	2

RM one-way ANOVA of Transform of TNF

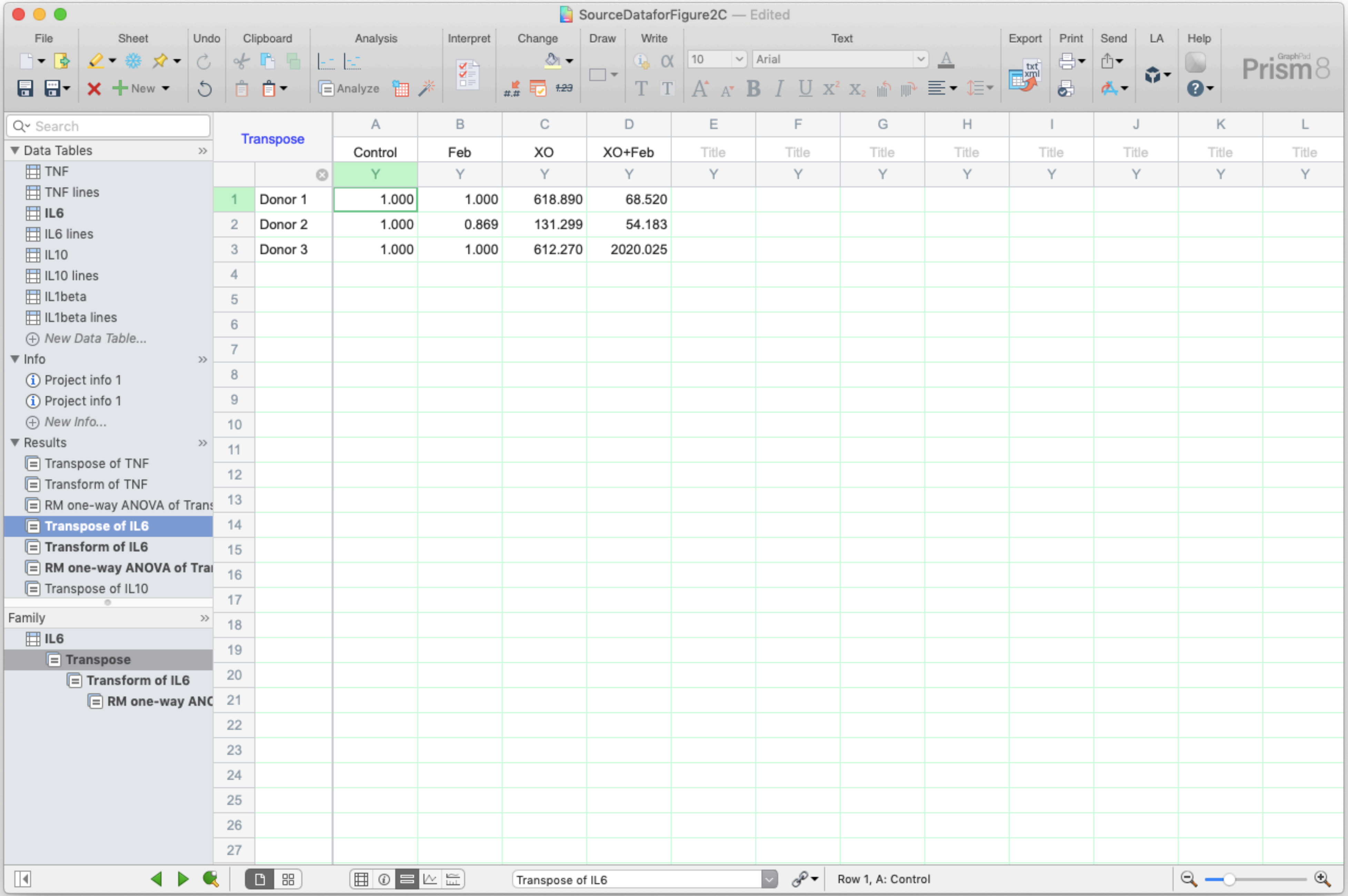

Transpose	Control	Feb	XO	XO+Feb
Donor 1	1.000	1.000	618.890	68.520
Donor 2	1.000	0.869	131.299	54.183
Donor 3	1.000	1.000	612.270	2020.025

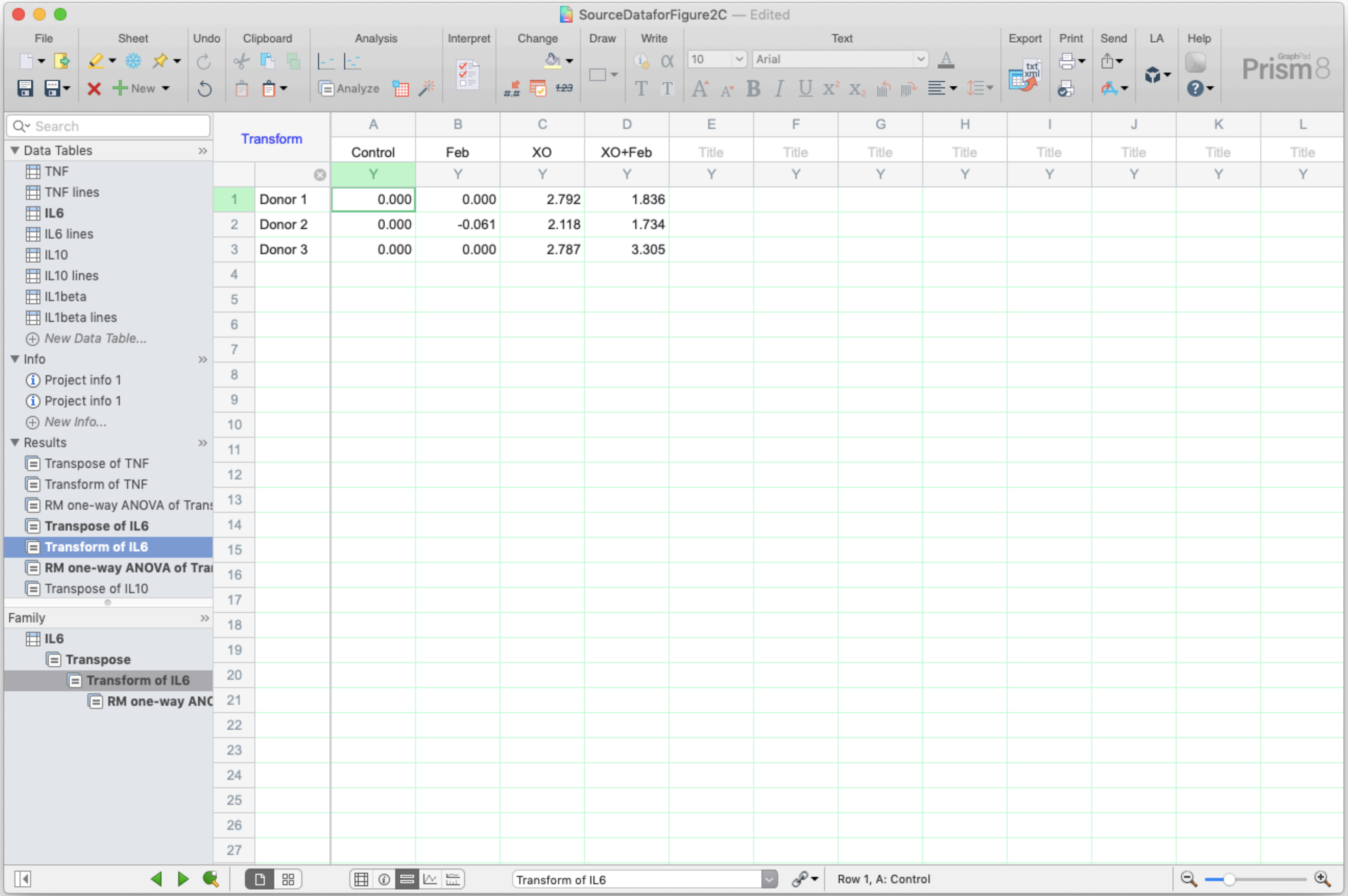

Transform	Control	Feb	XO	XO+Feb
Donor 1	0.000	0.000	2.792	1.836
Donor 2	0.000	-0.061	2.118	1.734
Donor 3	0.000	0.000	2.787	3.305

SourceDataforFigure2C — Edited

ANOVA results | Multiple comparisons

RM one-way ANOVA
ANOVA results

1	Table Analyzed	Transform of IL6				
2						
3	**Repeated measures ANOVA summary**					
4	Assume sphericity?	No				
5	F	30.68				
6	P value	0.0170				
7	P value summary	*				
8	Statistically significant (P < 0.05)?	Yes				
9	Geisser-Greenhouse's epsilon	0.4266				
10	R square	0.9388				
11						
12	**Was the matching effective?**					
13	F	1.739				
14	P value	0.2537				
15	P value summary	ns				
16	Is there significant matching (P < 0.05)?	No				
17	R square	0.03426				
18						
19	**ANOVA table**	**SS**	**DF**	**MS**	**F (DFn, DFd)**	**P value**
20	Treatment (between columns)	17.96	3	5.986	F (1.280, 2.560) = 30.68	P=0.0170
21	Individual (between rows)	0.6785	2	0.3393	F (2, 6) = 1.739	P=0.2537
22	Residual (random)	1.171	6	0.1951		
23	Total	19.81	11			
24						
25	**Data summary**					
26	Number of treatments (columns)	4				

RM one-way ANOVA of Transform of IL6 — Row 1, Column A

SourceDataforFigure2C — Edited

ANOVA results | Multiple comparisons

RM one-way ANOVA
Multiple comparisons

1	Number of families	1							
2	Number of comparisons p	6							
3	Alpha	0.05							
4									
5	**Tukey's multiple compa**	**Mean Diff.**	**95.00% CI of diff.**	**Significant?**	**Summary**	**Adjusted P Value**			
6	Control vs. Feb	0.02041	-0.1210 to 0.1618	No	ns	0.7683	A-B		
7	Control vs. XO	-2.566	-4.115 to -1.016	Yes	*	0.0188	A-C		
8	Control vs. XO+Feb	-2.292	-5.809 to 1.226	No	ns	0.1114	A-D		
9	Feb vs. XO	-2.586	-3.994 to -1.178	Yes	*	0.0154	B-C		
10	Feb vs. XO+Feb	-2.312	-5.754 to 1.130	No	ns	0.1053	B-D		
11	XO vs. XO+Feb	0.2739	-2.700 to 3.247	No	ns	0.9115	C-D		
12									
13	**Test details**	**Mean 1**	**Mean 2**	**Mean Diff.**	**SE of diff.**	**n1**	**n2**	**q**	**DF**
14	Control vs. Feb	0.000	-0.02041	0.02041	0.02041	3	3	1.414	2
15	Control vs. XO	0.000	2.566	-2.566	0.2237	3	3	16.22	2
16	Control vs. XO+Feb	0.000	2.292	-2.292	0.5077	3	3	6.384	2
17	Feb vs. XO	-0.02041	2.566	-2.586	0.2033	3	3	17.99	2
18	Feb vs. XO+Feb	-0.02041	2.292	-2.312	0.4968	3	3	6.582	2
19	XO vs. XO+Feb	2.566	2.292	0.2739	0.4291	3	3	0.9027	2

RM one-way ANOVA of Transform of IL6 — Row 1, Column A

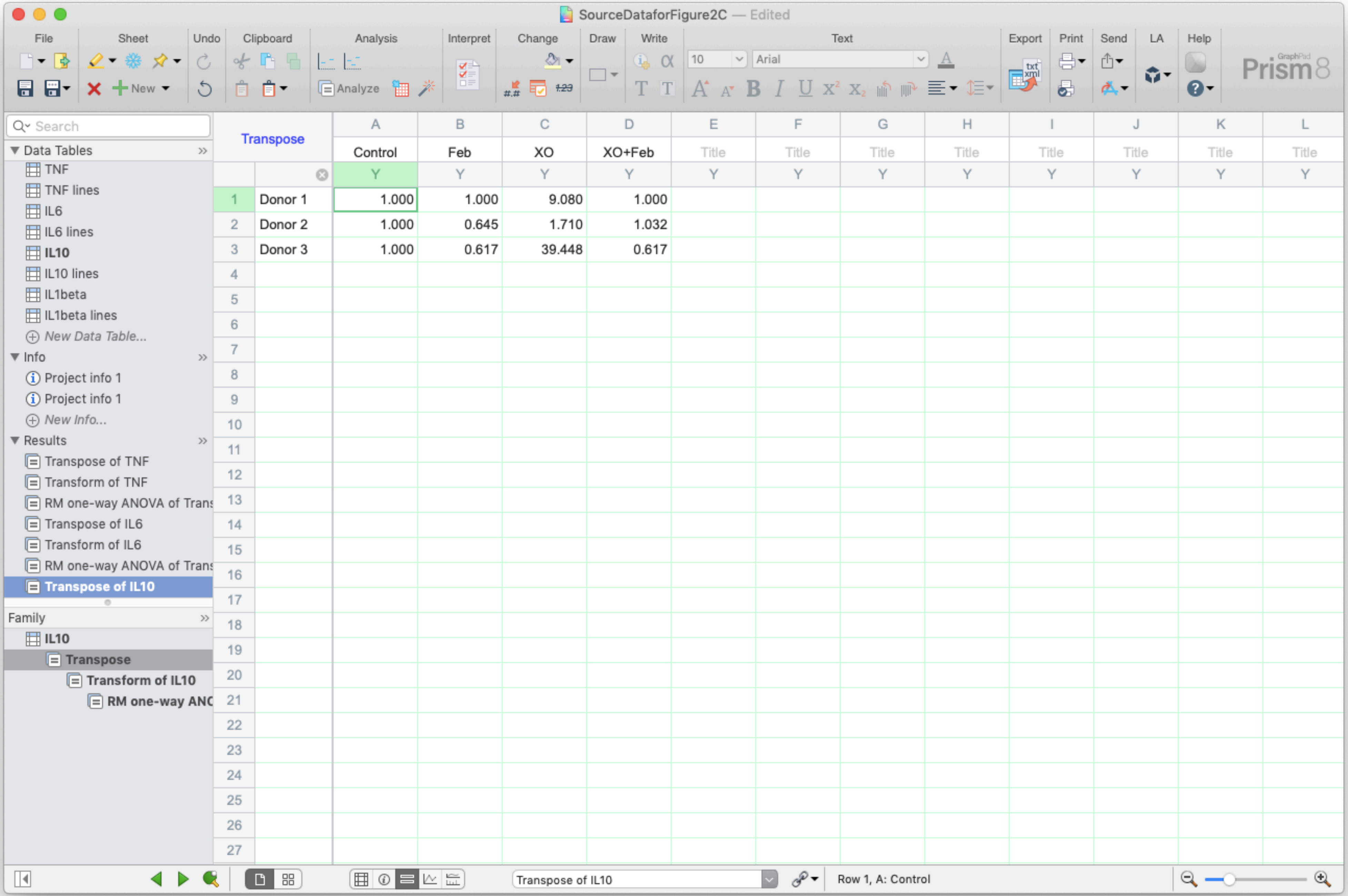

Transpose

	Control	Feb	XO	XO+Feb
Donor 1	1.000	1.000	9.080	1.000
Donor 2	1.000	0.645	1.710	1.032
Donor 3	1.000	0.617	39.448	0.617

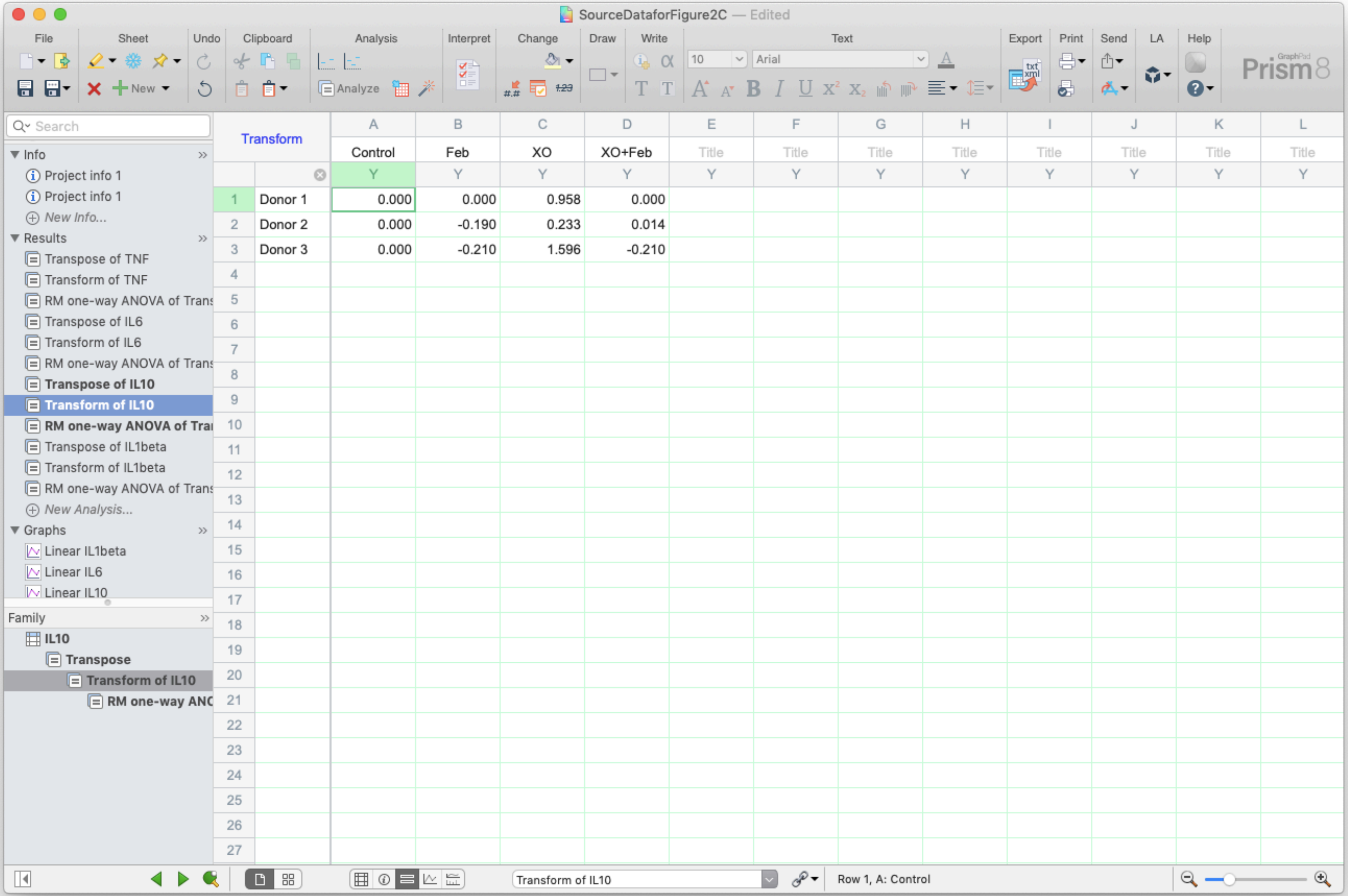
SourceDataforFigure2C — Edited
Transform
Control
Feb
XO
XO+Feb
Donor 1 0.000 0.000 0.958 0.000
Donor 2 0.000 -0.190 0.233 0.014
Donor 3 0.000 -0.210 1.596 -0.210
Info
Project info 1
Project info 1
New Info...
Results
Transpose of TNF
Transform of TNF
RM one-way ANOVA of Trans
Transpose of IL6
Transform of IL6
RM one-way ANOVA of Trans
Transpose of IL10
Transform of IL10
RM one-way ANOVA of Trai
Transpose of IL1beta
Transform of IL1beta
RM one-way ANOVA of Trans
New Analysis...
Graphs
Linear IL1beta
Linear IL6
Linear IL10
Family
IL10
Transpose
Transform of IL10
RM one-way ANC
Transform of IL10
Row 1, A: Control

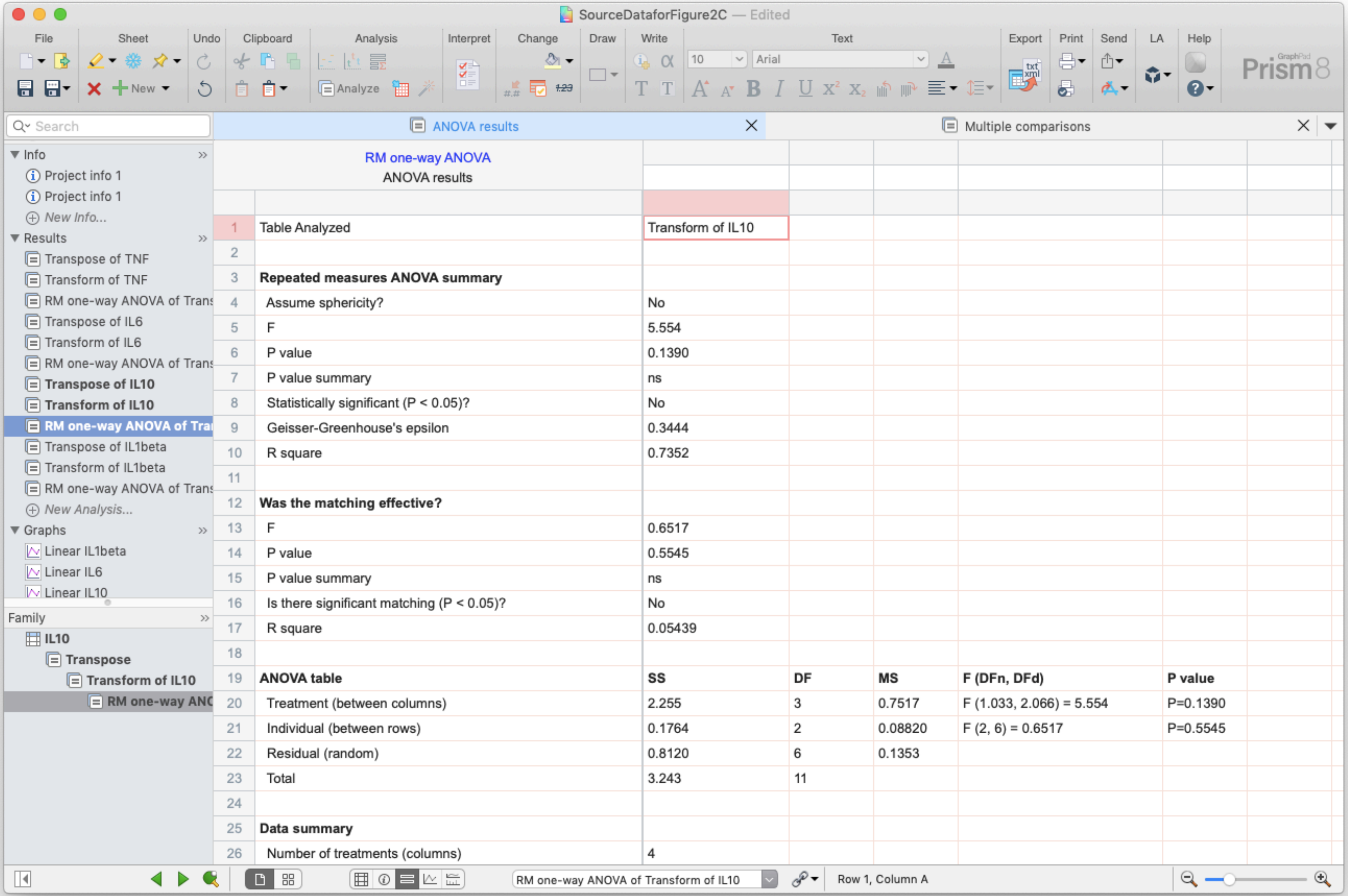

1	Table Analyzed	Transform of IL10				
2						
3	**Repeated measures ANOVA summary**					
4	Assume sphericity?	No				
5	F	5.554				
6	P value	0.1390				
7	P value summary	ns				
8	Statistically significant (P < 0.05)?	No				
9	Geisser-Greenhouse's epsilon	0.3444				
10	R square	0.7352				
11						
12	**Was the matching effective?**					
13	F	0.6517				
14	P value	0.5545				
15	P value summary	ns				
16	Is there significant matching (P < 0.05)?	No				
17	R square	0.05439				
18						
19	**ANOVA table**	**SS**	**DF**	**MS**	**F (DFn, DFd)**	**P value**
20	Treatment (between columns)	2.255	3	0.7517	F (1.033, 2.066) = 5.554	P=0.1390
21	Individual (between rows)	0.1764	2	0.08820	F (2, 6) = 0.6517	P=0.5545
22	Residual (random)	0.8120	6	0.1353		
23	Total	3.243	11			
24						
25	**Data summary**					
26	Number of treatments (columns)	4				

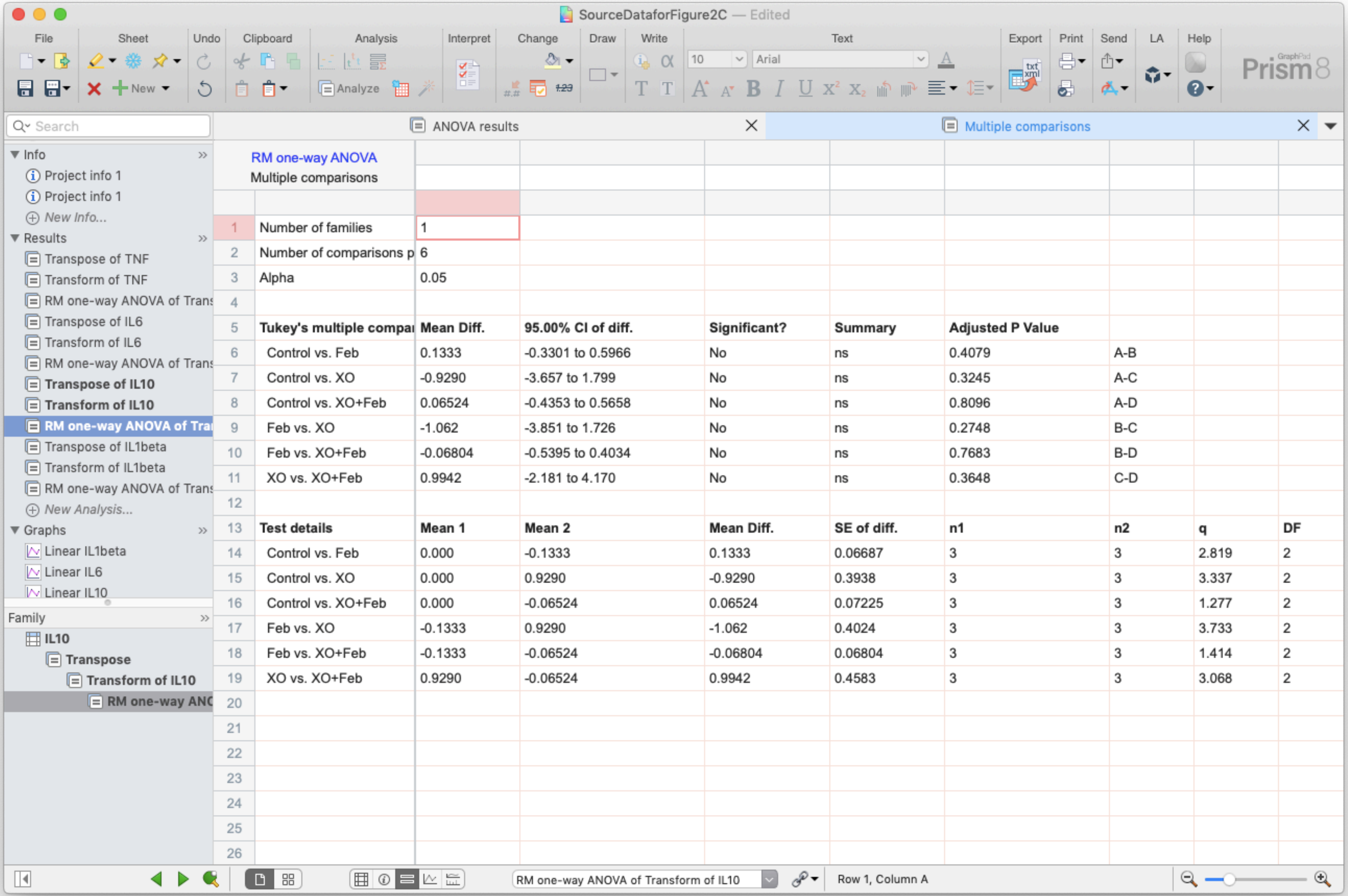

SourceDataforFigure2C — Edited

RM one-way ANOVA
Multiple comparisons

1	Number of families	1							
2	Number of comparisons p	6							
3	Alpha	0.05							
4									
5	Tukey's multiple compa	Mean Diff.	95.00% CI of diff.	Significant?	Summary	Adjusted P Value			
6	Control vs. Feb	0.1333	-0.3301 to 0.5966	No	ns	0.4079	A-B		
7	Control vs. XO	-0.9290	-3.657 to 1.799	No	ns	0.3245	A-C		
8	Control vs. XO+Feb	0.06524	-0.4353 to 0.5658	No	ns	0.8096	A-D		
9	Feb vs. XO	-1.062	-3.851 to 1.726	No	ns	0.2748	B-C		
10	Feb vs. XO+Feb	-0.06804	-0.5395 to 0.4034	No	ns	0.7683	B-D		
11	XO vs. XO+Feb	0.9942	-2.181 to 4.170	No	ns	0.3648	C-D		
12									
13	Test details	Mean 1	Mean 2	Mean Diff.	SE of diff.	n1	n2	q	DF
14	Control vs. Feb	0.000	-0.1333	0.1333	0.06687	3	3	2.819	2
15	Control vs. XO	0.000	0.9290	-0.9290	0.3938	3	3	3.337	2
16	Control vs. XO+Feb	0.000	-0.06524	0.06524	0.07225	3	3	1.277	2
17	Feb vs. XO	-0.1333	0.9290	-1.062	0.4024	3	3	3.733	2
18	Feb vs. XO+Feb	-0.1333	-0.06524	-0.06804	0.06804	3	3	1.414	2
19	XO vs. XO+Feb	0.9290	-0.06524	0.9942	0.4583	3	3	3.068	2

RM one-way ANOVA of Transform of IL10

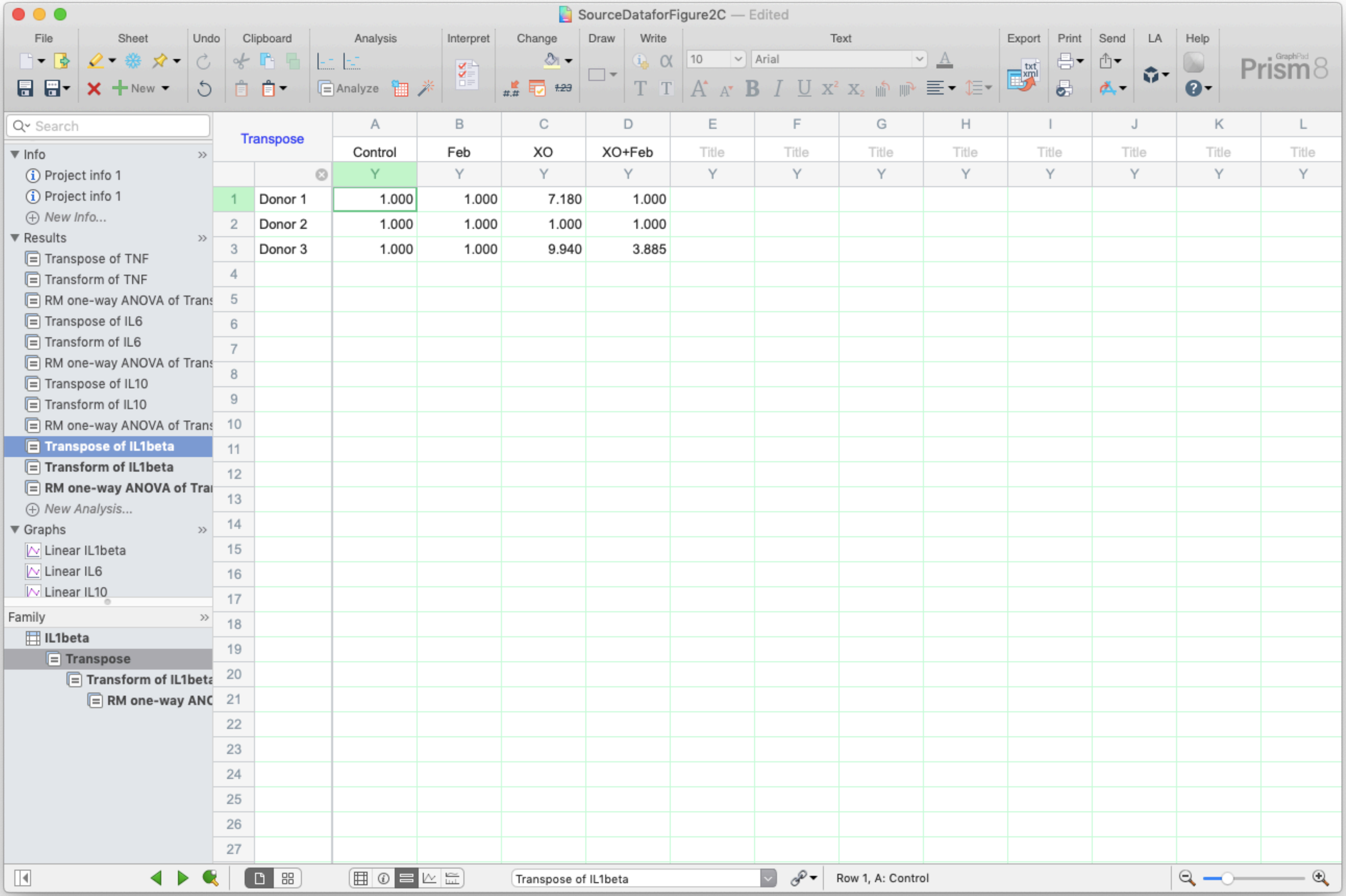

	Control	Feb	XO	XO+Feb
Donor 1	1.000	1.000	7.180	1.000
Donor 2	1.000	1.000	1.000	1.000
Donor 3	1.000	1.000	9.940	3.885

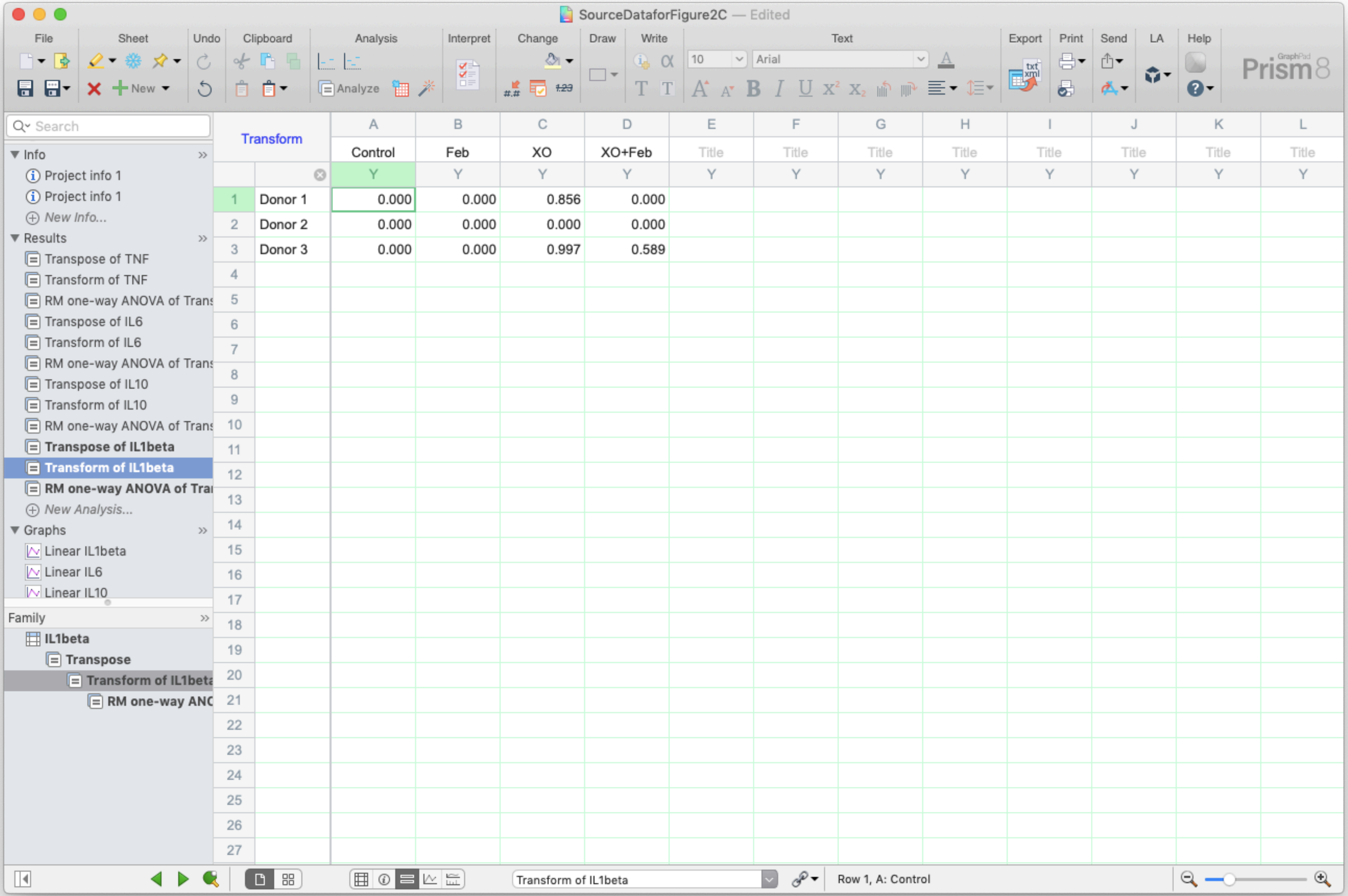
SourceDataforFigure2C — Edited
File
Sheet
Undo
Clipboard
Analysis
Interpret
Change
Draw
Write
Text
Export
Print
Send
LA
Help
New
Analyze
Arial
Prism8
Search
Transform
Info
Project info 1
Project info 1
New Info...
Results
Transpose of TNF
Transform of TNF
RM one-way ANOVA of Trans
Transpose of IL6
Transform of IL6
RM one-way ANOVA of Trans
Transpose of IL10
Transform of IL10
RM one-way ANOVA of Trans
Transpose of IL1beta
Transform of IL1beta
RM one-way ANOVA of Trai
New Analysis...
Graphs
Linear IL1beta
Linear IL6
Linear IL10
Family
IL1beta
Transpose
Transform of IL1beta
RM one-way ANC
Control
Feb
XO
XO+Feb
Donor 1 0.000 0.000 0.856 0.000
Donor 2 0.000 0.000 0.000 0.000
Donor 3 0.000 0.000 0.997 0.589
Transform of IL1beta
Row 1, A: Control

SourceDataforFigure2C — Edited

ANOVA results | Multiple comparisons

- Info
  - Project info 1
  - Project info 1
  - New Info...
- Results
  - Transpose of TNF
  - Transform of TNF
  - RM one-way ANOVA of Trans
  - Transpose of IL6
  - Transform of IL6
  - RM one-way ANOVA of Trans
  - Transpose of IL10
  - Transform of IL10
  - RM one-way ANOVA of Trans
  - **Transpose of IL1beta**
  - **Transform of IL1beta**
  - **RM one-way ANOVA of Tra**
  - New Analysis...
- Graphs
  - Linear IL1beta
  - Linear IL6
  - Linear IL10
- Family
  - **IL1beta**
    - **Transpose**
      - **Transform of IL1beta**
        - **RM one-way ANC**

RM one-way ANOVA
ANOVA results

1	Table Analyzed	Transform of IL1beta				
2						
3	**Repeated measures ANOVA summary**					
4	Assume sphericity?	No				
5	F	3.062				
6	P value	0.1842				
7	P value summary	ns				
8	Statistically significant (P < 0.05)?	No				
9	Geisser-Greenhouse's epsilon	0.5060				
10	R square	0.6049				
11						
12	**Was the matching effective?**					
13	F	1.897				
14	P value	0.2299				
15	P value summary	ns				
16	Is there significant matching (P < 0.05)?	No				
17	R square	0.1999				
18						
19	**ANOVA table**	**SS**	**DF**	**MS**	**F (DFn, DFd)**	**P value**
20	Treatment (between columns)	0.7636	3	0.2545	F (1.518, 3.036) = 3.062	P=0.1842
21	Individual (between rows)	0.3154	2	0.1577	F (2, 6) = 1.897	P=0.2299
22	Residual (random)	0.4988	6	0.08313		
23	Total	1.578	11			
24						
25	**Data summary**					
26	Number of treatments (columns)	4				

RM one-way ANOVA of Transform of IL1beta — Row 1, Column A

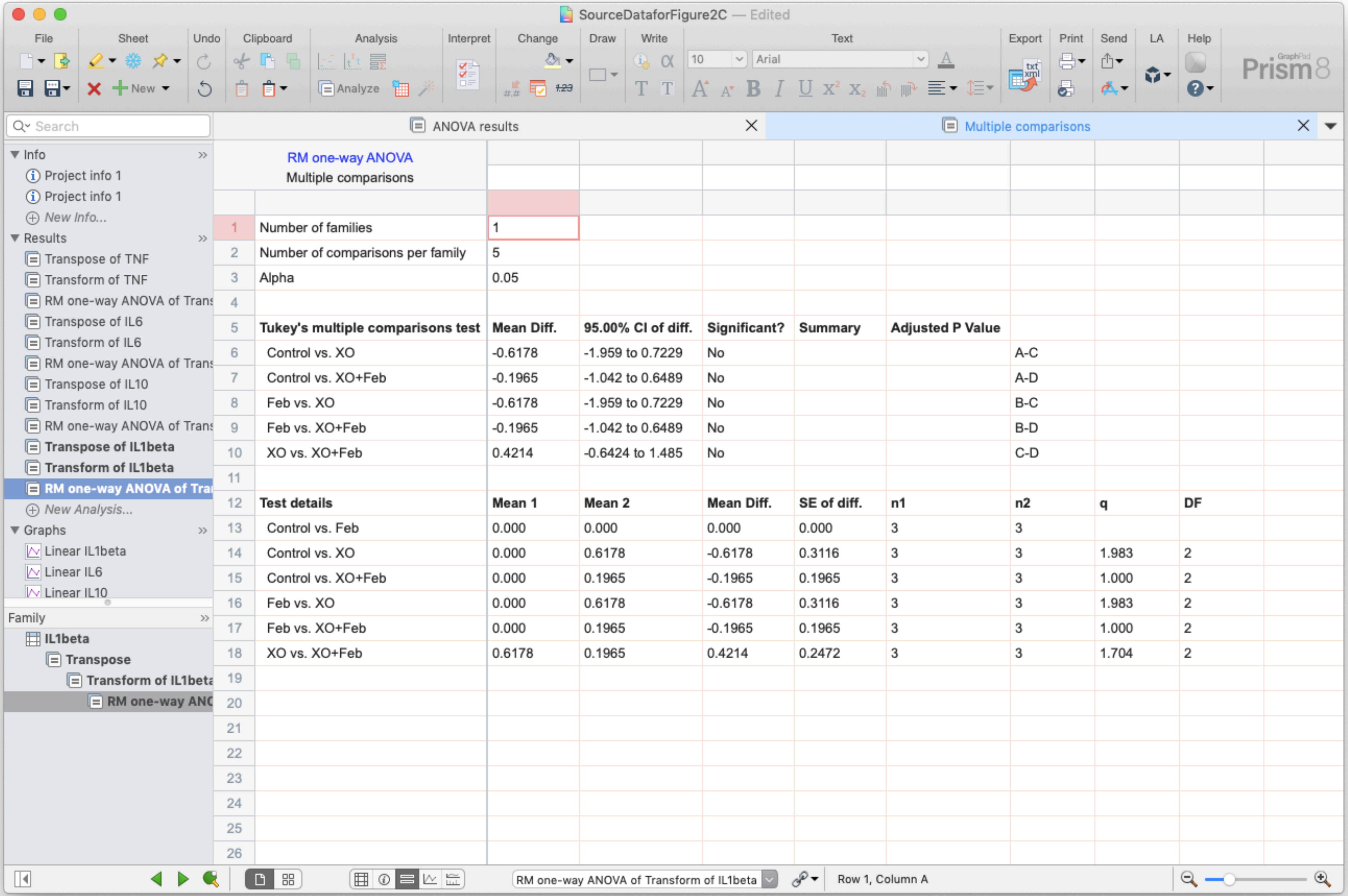

RM one-way ANOVA
Multiple comparisons

1	Number of families	1							
2	Number of comparisons per family	5							
3	Alpha	0.05							
4									
5	**Tukey's multiple comparisons test**	**Mean Diff.**	**95.00% CI of diff.**	**Significant?**	**Summary**	**Adjusted P Value**			
6	Control vs. XO	-0.6178	-1.959 to 0.7229	No			A-C		
7	Control vs. XO+Feb	-0.1965	-1.042 to 0.6489	No			A-D		
8	Feb vs. XO	-0.6178	-1.959 to 0.7229	No			B-C		
9	Feb vs. XO+Feb	-0.1965	-1.042 to 0.6489	No			B-D		
10	XO vs. XO+Feb	0.4214	-0.6424 to 1.485	No			C-D		
11									
12	**Test details**	**Mean 1**	**Mean 2**	**Mean Diff.**	**SE of diff.**	**n1**	**n2**	**q**	**DF**
13	Control vs. Feb	0.000	0.000	0.000	0.000	3	3		
14	Control vs. XO	0.000	0.6178	-0.6178	0.3116	3	3	1.983	2
15	Control vs. XO+Feb	0.000	0.1965	-0.1965	0.1965	3	3	1.000	2
16	Feb vs. XO	0.000	0.6178	-0.6178	0.3116	3	3	1.983	2
17	Feb vs. XO+Feb	0.000	0.1965	-0.1965	0.1965	3	3	1.000	2
18	XO vs. XO+Feb	0.6178	0.1965	0.4214	0.2472	3	3	1.704	2

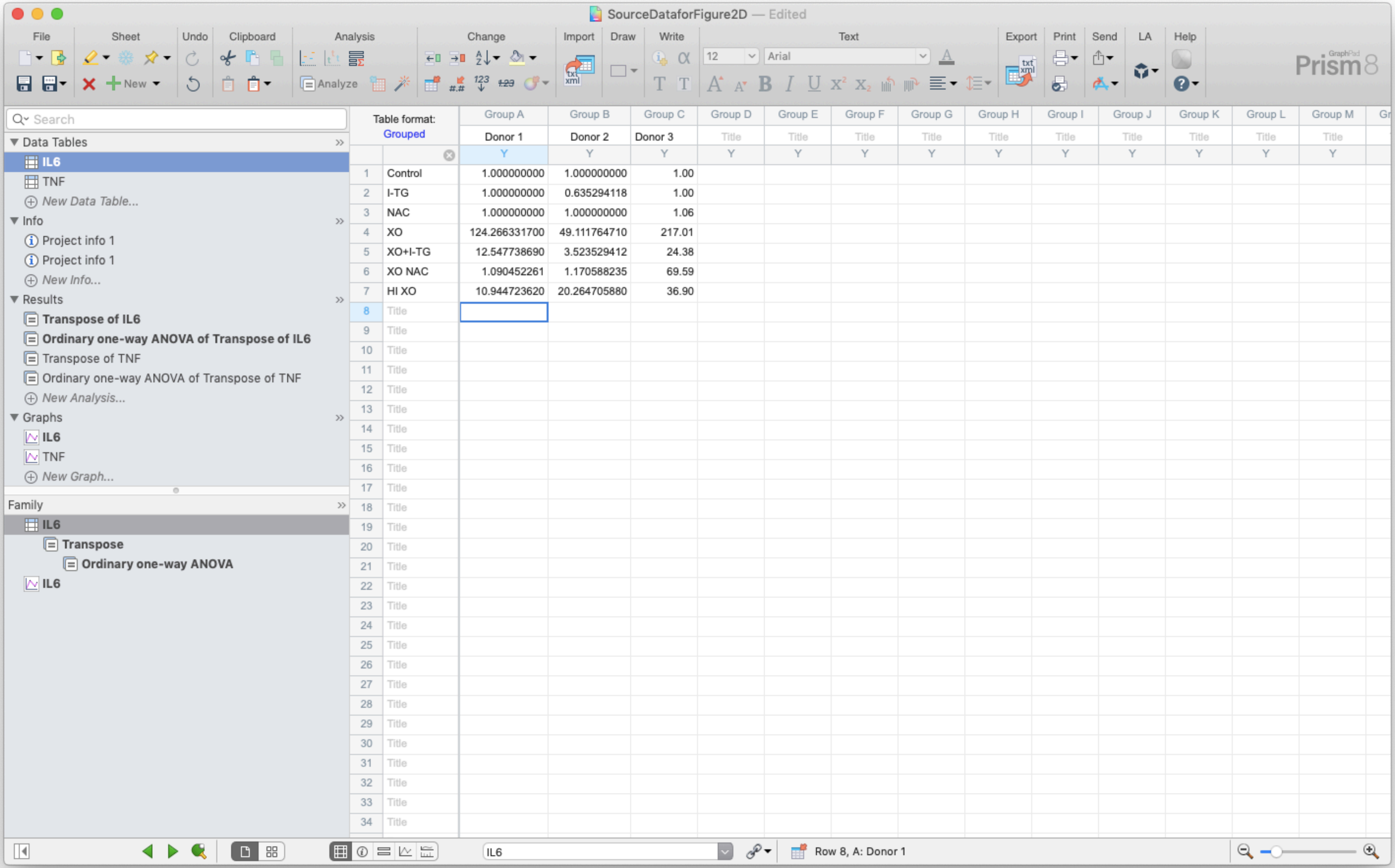

		Group A	Group B	Group C
		Donor 1	Donor 2	Donor 3
1	Control	1.000000000	1.000000000	1.00
2	I-TG	1.000000000	0.635294118	1.00
3	NAC	1.000000000	1.000000000	1.06
4	XO	124.266331700	49.111764710	217.01
5	XO+I-TG	12.547738690	3.523529412	24.38
6	XO NAC	1.090452261	1.170588235	69.59
7	HI XO	10.944723620	20.264705880	36.90

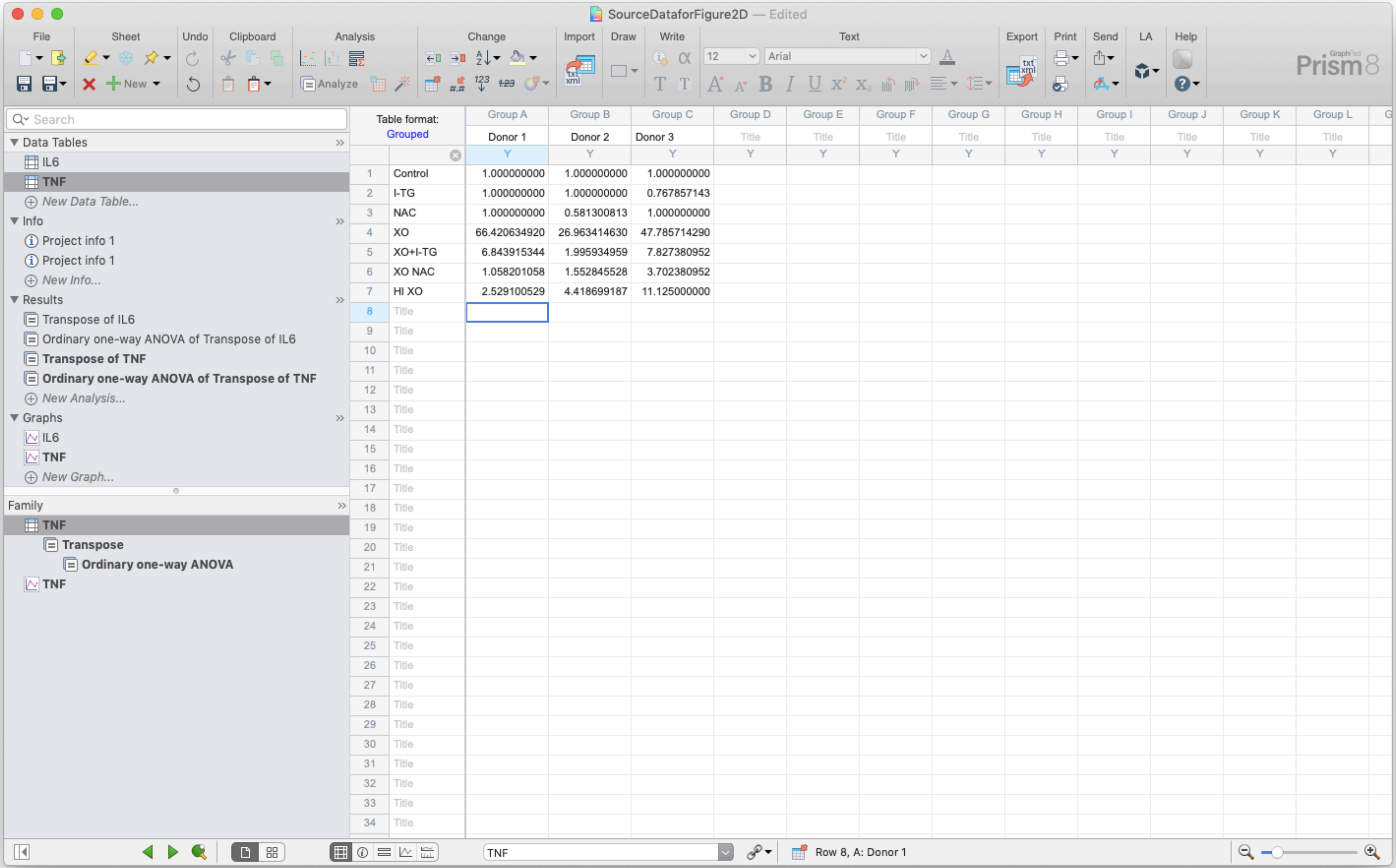

		Group A	Group B	Group C
		Donor 1	Donor 2	Donor 3
1	Control	1.000000000	1.000000000	1.000000000
2	I-TG	1.000000000	1.000000000	0.767857143
3	NAC	1.000000000	0.581300813	1.000000000
4	XO	66.420634920	26.963414630	47.785714290
5	XO+I-TG	6.843915344	1.995934959	7.827380952
6	XO NAC	1.058201058	1.552845528	3.702380952
7	HI XO	2.529100529	4.418699187	11.125000000

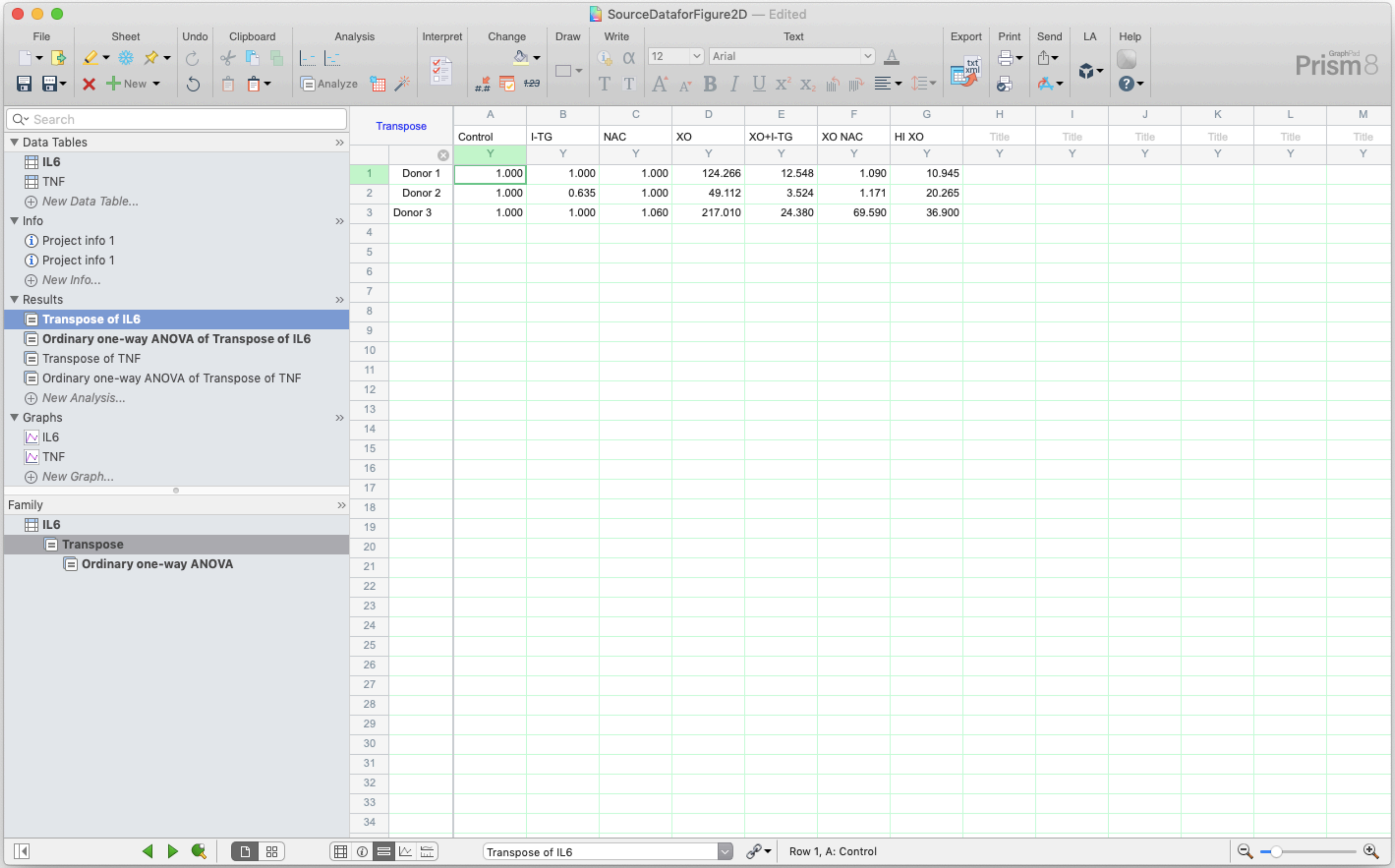

Transpose	Control	I-TG	NAC	XO	XO+I-TG	XO NAC	HI XO
Donor 1	1.000	1.000	1.000	124.266	12.548	1.090	10.945
Donor 2	1.000	0.635	1.000	49.112	3.524	1.171	20.265
Donor 3	1.000	1.000	1.060	217.010	24.380	69.590	36.900

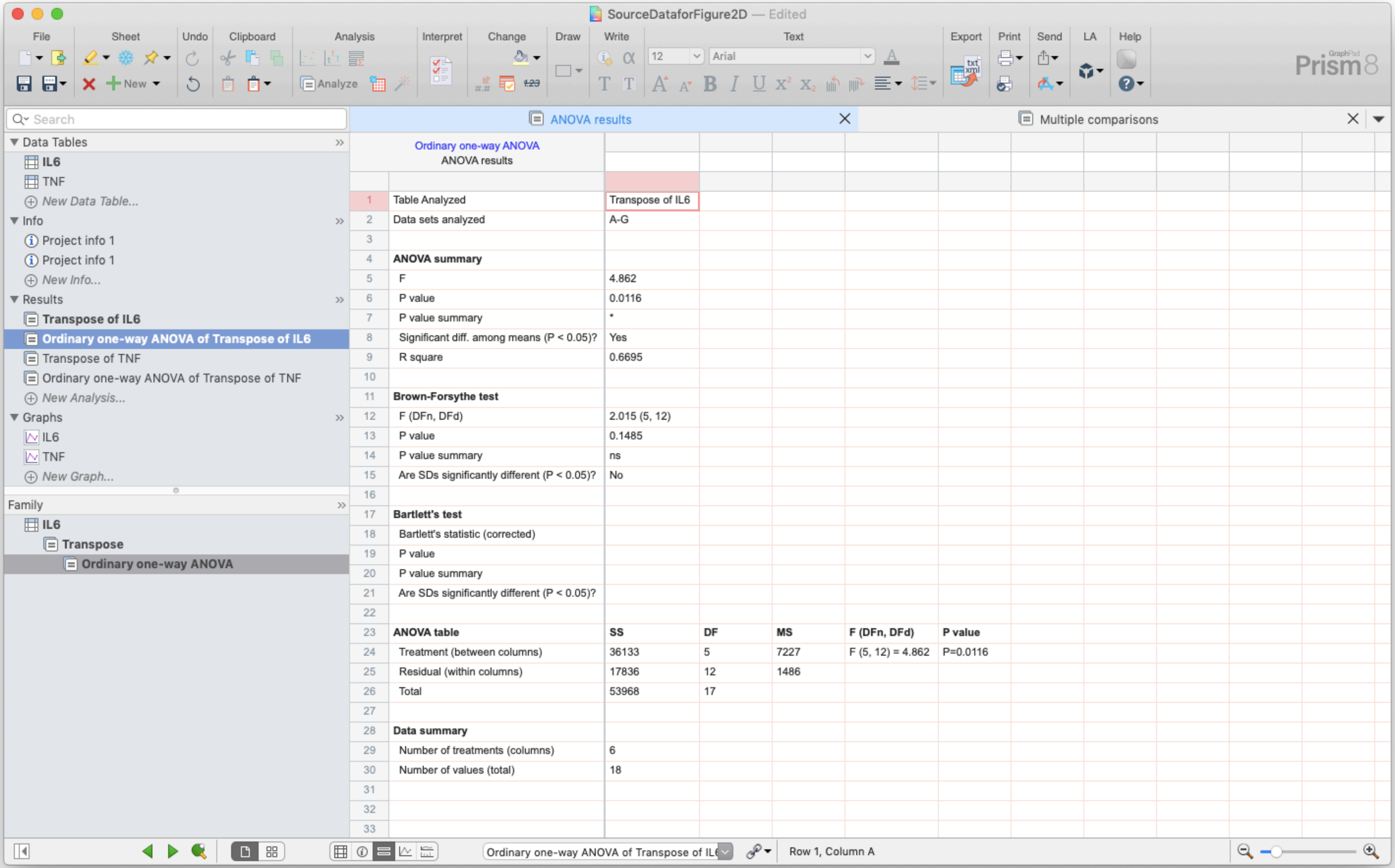

Ordinary one-way ANOVA
ANOVA results

1	Table Analyzed	Transpose of IL6				
2	Data sets analyzed	A-G				
3						
4	**ANOVA summary**					
5	F	4.862				
6	P value	0.0116				
7	P value summary	*				
8	Significant diff. among means (P < 0.05)?	Yes				
9	R square	0.6695				
10						
11	**Brown-Forsythe test**					
12	F (DFn, DFd)	2.015 (5, 12)				
13	P value	0.1485				
14	P value summary	ns				
15	Are SDs significantly different (P < 0.05)?	No				
16						
17	**Bartlett's test**					
18	Bartlett's statistic (corrected)					
19	P value					
20	P value summary					
21	Are SDs significantly different (P < 0.05)?					
22						
23	**ANOVA table**	**SS**	**DF**	**MS**	**F (DFn, DFd)**	**P value**
24	Treatment (between columns)	36133	5	7227	F (5, 12) = 4.862	P=0.0116
25	Residual (within columns)	17836	12	1486		
26	Total	53968	17			
27						
28	**Data summary**					
29	Number of treatments (columns)	6				
30	Number of values (total)	18				
31						
32						
33						

SourceDataforFigure2D — Edited

Ordinary one-way ANOVA
Multiple comparisons

5	Tukey's multiple comparis	Mean Diff.	95.00% CI of diff.	Significant?	Summary	Adjusted P Value			
6	Control vs. NAC	-0.02000	-105.8 to 105.7	No	ns	>0.9999	A-C		
7	Control vs. XO	-129.1	-234.9 to -23.40	Yes	*	0.0143	A-D		
8	Control vs. XO+I-TG	-12.48	-118.2 to 93.25	No	ns	0.9984	A-E		
9	Control vs. XO NAC	-22.95	-128.7 to 82.78	No	ns	0.9743	A-F		
10	Control vs. HI XO	-21.70	-127.4 to 84.03	No	ns	0.9798	A-G		
11	NAC vs. XO	-129.1	-234.8 to -23.38	Yes	*	0.0143	C-D		
12	NAC vs. XO+I-TG	-12.46	-118.2 to 93.27	No	ns	0.9984	C-E		
13	NAC vs. XO NAC	-22.93	-128.7 to 82.80	No	ns	0.9744	C-F		
14	NAC vs. HI XO	-21.68	-127.4 to 84.05	No	ns	0.9798	C-G		
15	XO vs. XO+I-TG	116.6	10.91 to 222.4	Yes	*	0.0279	D-E		
16	XO vs. XO NAC	106.2	0.4471 to 211.9	Yes	*	0.0488	D-F		
17	XO vs. HI XO	107.4	1.694 to 213.2	Yes	*	0.0457	D-G		
18	XO+I-TG vs. XO NAC	-10.47	-116.2 to 95.27	No	ns	0.9993	E-F		
19	XO+I-TG vs. HI XO	-9.219	-115.0 to 96.51	No	ns	0.9996	E-G		
20	XO NAC vs. HI XO	1.247	-104.5 to 107.0	No	ns	>0.9999	F-G		
21									
22	Test details	Mean 1	Mean 2	Mean Diff.	SE of diff.	n1	n2	q	DF
23	Control vs. NAC	1.000	1.020	-0.02000	31.48	3	3	0.0008985	12
24	Control vs. XO	1.000	130.1	-129.1	31.48	3	3	5.801	12
25	Control vs. XO+I-TG	1.000	13.48	-12.48	31.48	3	3	0.5609	12
26	Control vs. XO NAC	1.000	23.95	-22.95	31.48	3	3	1.031	12
27	Control vs. HI XO	1.000	22.70	-21.70	31.48	3	3	0.9751	12
28	NAC vs. XO	1.020	130.1	-129.1	31.48	3	3	5.801	12
29	NAC vs. XO+I-TG	1.020	13.48	-12.46	31.48	3	3	0.5600	12
30	NAC vs. XO NAC	1.020	23.95	-22.93	31.48	3	3	1.030	12
31	NAC vs. HI XO	1.020	22.70	-21.68	31.48	3	3	0.9742	12
32	XO vs. XO+I-TG	130.1	13.48	116.6	31.48	3	3	5.241	12
33	XO vs. XO NAC	130.1	23.95	106.2	31.48	3	3	4.770	12
34	XO vs. HI XO	130.1	22.70	107.4	31.48	3	3	4.826	12
35	XO+I-TG vs. XO NAC	13.48	23.95	-10.47	31.48	3	3	0.4702	12
36	XO+I-TG vs. HI XO	13.48	22.70	-9.219	31.48	3	3	0.4142	12
37	XO NAC vs. HI XO	23.95	22.70	1.247	31.48	3	3	0.05603	12

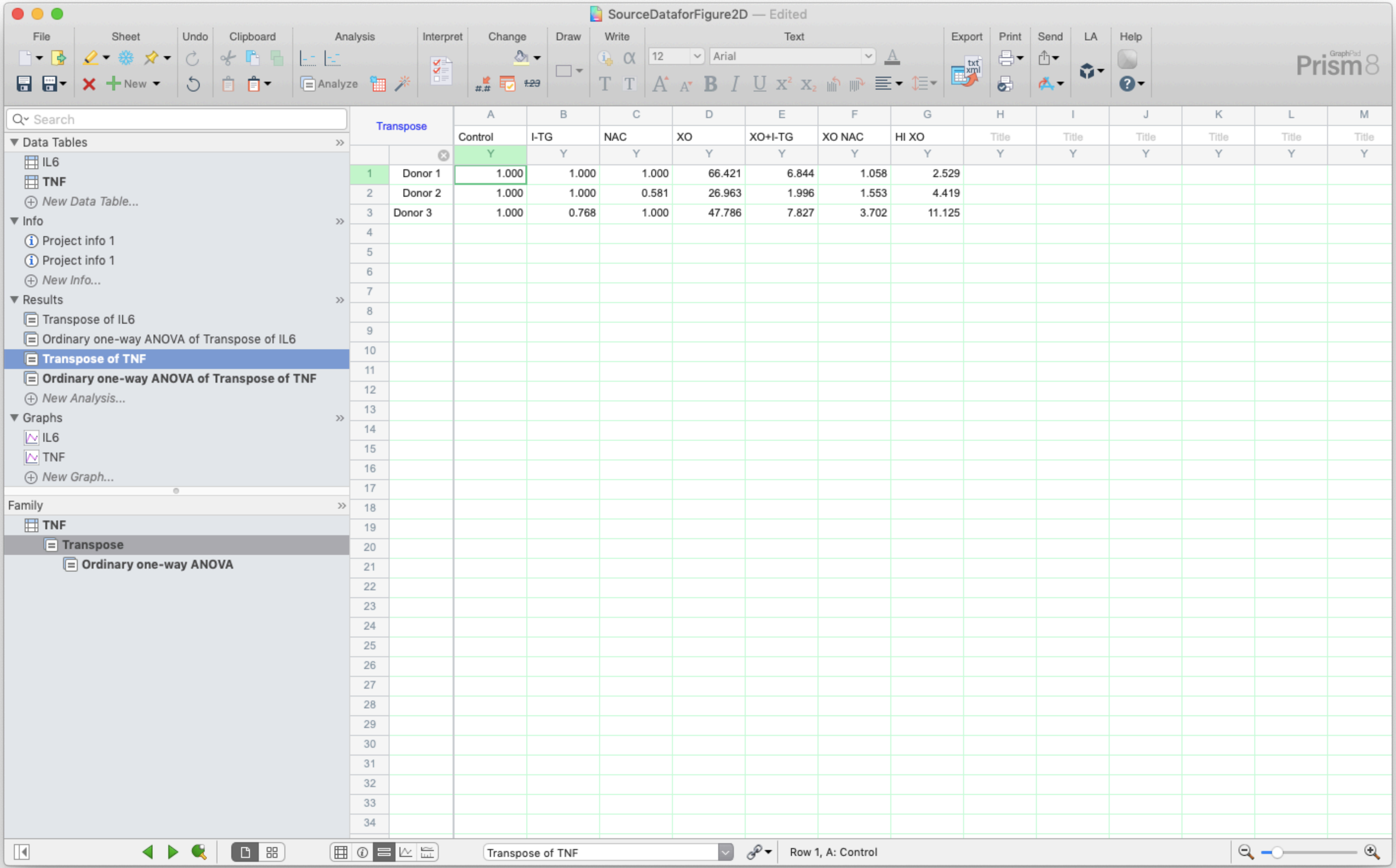

Transpose	Control	I-TG	NAC	XO	XO+I-TG	XO NAC	HI XO
Donor 1	1.000	1.000	1.000	66.421	6.844	1.058	2.529
Donor 2	1.000	1.000	0.581	26.963	1.996	1.553	4.419
Donor 3	1.000	0.768	1.000	47.786	7.827	3.702	11.125

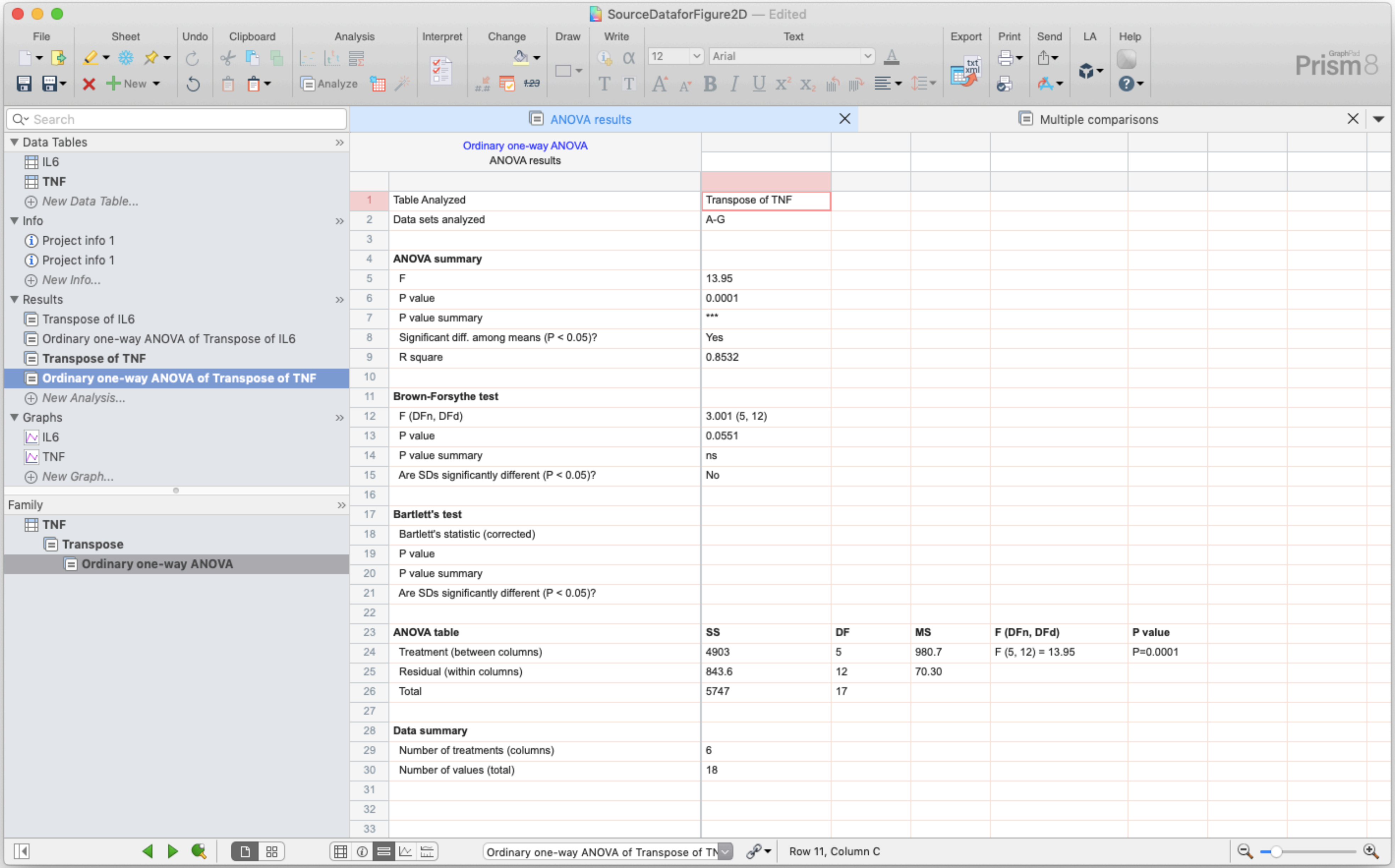

Ordinary one-way ANOVA
ANOVA results

1	Table Analyzed	Transpose of TNF				
2	Data sets analyzed	A-G				
3						
4	**ANOVA summary**					
5	F	13.95				
6	P value	0.0001				
7	P value summary	***				
8	Significant diff. among means (P < 0.05)?	Yes				
9	R square	0.8532				
10						
11	**Brown-Forsythe test**					
12	F (DFn, DFd)	3.001 (5, 12)				
13	P value	0.0551				
14	P value summary	ns				
15	Are SDs significantly different (P < 0.05)?	No				
16						
17	**Bartlett's test**					
18	Bartlett's statistic (corrected)					
19	P value					
20	P value summary					
21	Are SDs significantly different (P < 0.05)?					
22						
23	**ANOVA table**	**SS**	**DF**	**MS**	**F (DFn, DFd)**	**P value**
24	Treatment (between columns)	4903	5	980.7	F (5, 12) = 13.95	P=0.0001
25	Residual (within columns)	843.6	12	70.30		
26	Total	5747	17			
27						
28	**Data summary**					
29	Number of treatments (columns)	6				
30	Number of values (total)	18				
31						
32						
33						

SourceDataforFigure2D — Edited

ANVOA results | Multiple comparisons

Ordinary one-way ANOVA
Multiple comparisons

4							
5	**Tukey's multiple comparis**	**Significant?**	**Summary**	**Adjusted P Value**			
6	Control vs. NAC	No	ns	>0.9999	A-C		
7	Control vs. XO	Yes	***	0.0002	A-D		
8	Control vs. XO+I-TG	No	ns	0.9826	A-E		
9	Control vs. XO NAC	No	ns	>0.9999	A-F		
10	Control vs. HI XO	No	ns	0.9736	A-G		
11	NAC vs. XO	Yes	***	0.0002	C-D		
12	NAC vs. XO+I-TG	No	ns	0.9802	C-E		
13	NAC vs. XO NAC	No	ns	>0.9999	C-F		
14	NAC vs. HI XO	No	ns	0.9703	C-G		
15	XO vs. XO+I-TG	Yes	***	0.0006	D-E		
16	XO vs. XO NAC	Yes	***	0.0003	D-F		
17	XO vs. HI XO	Yes	***	0.0007	D-G		
18	XO+I-TG vs. XO NAC	No	ns	0.9950	E-F		
19	XO+I-TG vs. HI XO	No	ns	>0.9999	E-G		
20	XO NAC vs. HI XO	No	ns	0.9911	F-G		
21							
22	**Test details**	**Mean Diff.**	**SE of diff.**	**n1**	**n2**	**q**	**DF**
23	Control vs. NAC	0.1396	6.846	3	3	0.02883	12
24	Control vs. XO	-46.06	6.846	3	3	9.514	12
25	Control vs. XO+I-TG	-4.556	6.846	3	3	0.9411	12
26	Control vs. XO NAC	-1.104	6.846	3	3	0.2282	12
27	Control vs. HI XO	-5.024	6.846	3	3	1.038	12
28	NAC vs. XO	-46.20	6.846	3	3	9.543	12
29	NAC vs. XO+I-TG	-4.695	6.846	3	3	0.9699	12
30	NAC vs. XO NAC	-1.244	6.846	3	3	0.2570	12
31	NAC vs. HI XO	-5.164	6.846	3	3	1.067	12
32	XO vs. XO+I-TG	41.50	6.846	3	3	8.573	12
33	XO vs. XO NAC	44.95	6.846	3	3	9.286	12
34	XO vs. HI XO	41.03	6.846	3	3	8.476	12
35	XO+I-TG vs. XO NAC	3.451	6.846	3	3	0.7130	12
36	XO+I-TG vs. HI XO	-0.4685	6.846	3	3	0.09679	12

Ordinary one-way ANOVA of Transpose of TNF — Row 11, Column C

SourceDataforFigure2E

Data Tables
- TNF
- IL-6
- IL-10
- IL-1beta
- New Data Table...

Info
- Project info 1
- Project info 1
- New Info...

Results
- **Ordinary one-way ANOVA of TNF**
- Ordinary one-way ANOVA of IL-6
- Ordinary one-way ANOVA of IL-10
- Ordinary one-way ANOVA of IL-1beta

Family
- **TNF**
  - **Ordinary one-way ANOVA**
- **TNF**

Table format: Column

		Group A	Group B	Group C	Group D	Group E	Group F	Group G	Group H
		Control	RBC	iRBC	RBCL	iRBCL	XO	LPS	Title
		Y	Y	Y	Y	Y	Y	Y	Y
1	AC	58.25333333	35.52666667	276.3733333	44.12000000	402.7700000	4283.54000000	7938.19666700	
2	JMV	18.41666667	20.10666667	142.2933333	16.89333333	78.0366667	3445.94000000	5735.90000000	
3	Maureen SJT	11.29000000			21.32000000	346.0800000	746.65000000	1894.32000000	
4	Maureen 2.10.14	3.09000000	1.97000000	30.6100000				3037.01000000	

TNF

Row 8, Column RT

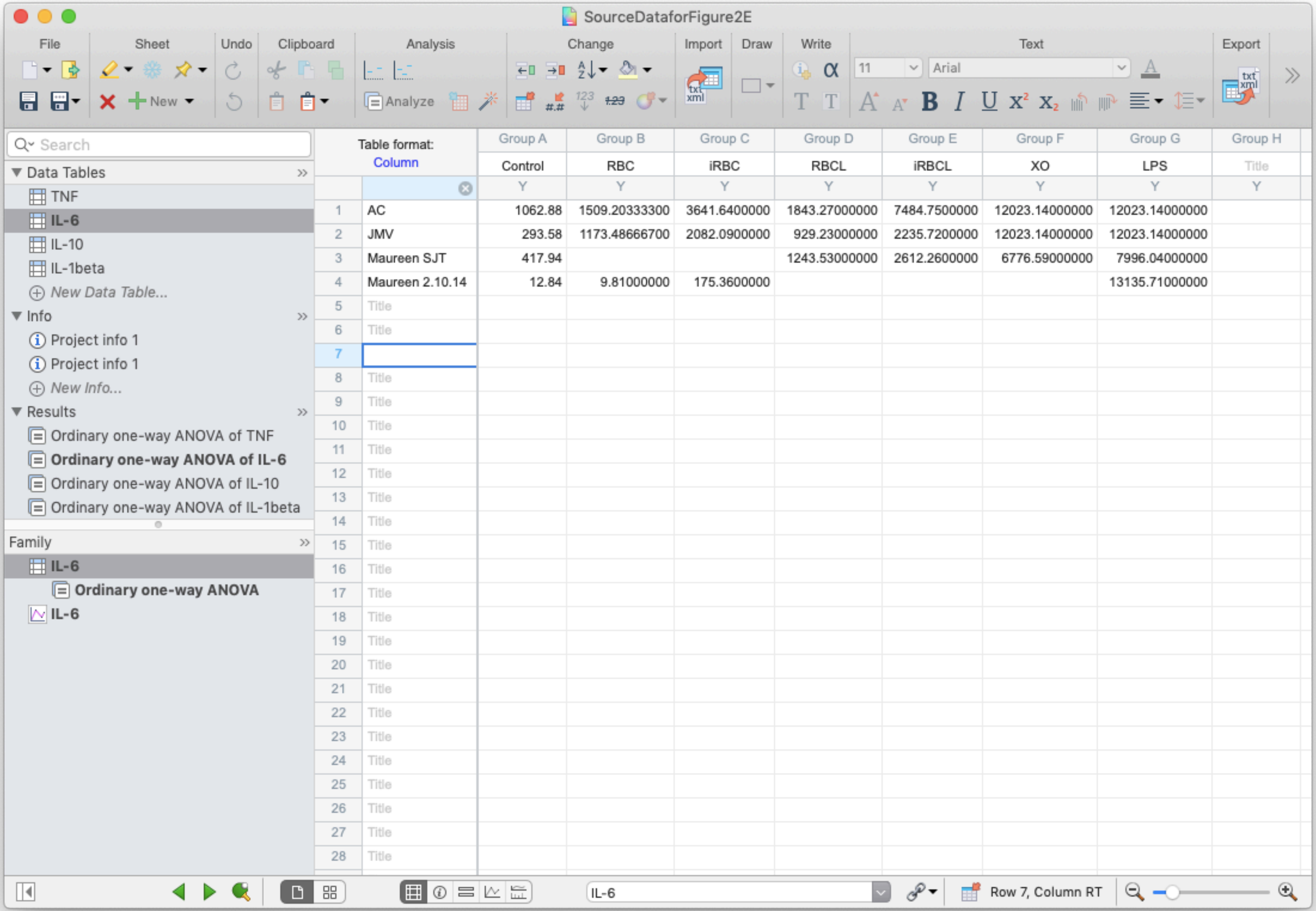

Table format: Column	Group A	Group B	Group C	Group D	Group E	Group F	Group G	Group H
	Control	RBC	iRBC	RBCL	iRBCL	XO	LPS	Title
	Y	Y	Y	Y	Y	Y	Y	Y
1 AC	1062.88	1509.20333300	3641.6400000	1843.27000000	7484.7500000	12023.14000000	12023.14000000	
2 JMV	293.58	1173.48666700	2082.0900000	929.23000000	2235.7200000	12023.14000000	12023.14000000	
3 Maureen SJT	417.94			1243.53000000	2612.2600000	6776.59000000	7996.04000000	
4 Maureen 2.10.14	12.84	9.81000000	175.3600000				13135.71000000	

SourceDataforFigure2E

Table format: Column

	Group A	Group B	Group C	Group D	Group E	Group F	Group G
	Control	RBC	iRBC	RBCL	iRBCL	XO	LPS
	Y	Y	Y	Y	Y	Y	Y
1 AC	2.320000000	4.996666667	28.90666667	4.626666667	30.316666670	373.11666670	650.57333330
2 JMV	1.816666667	2.593333333	8.07000000	2.380000000	5.253333333	160.32000000	234.05333330
3 Maureen SJT	0.000000000			0.000000000	5.290000000	13.31000000	45.60000000
4 Maureen 2.10.14	2.380000000	2.480000000	7.09000000				178.90000000

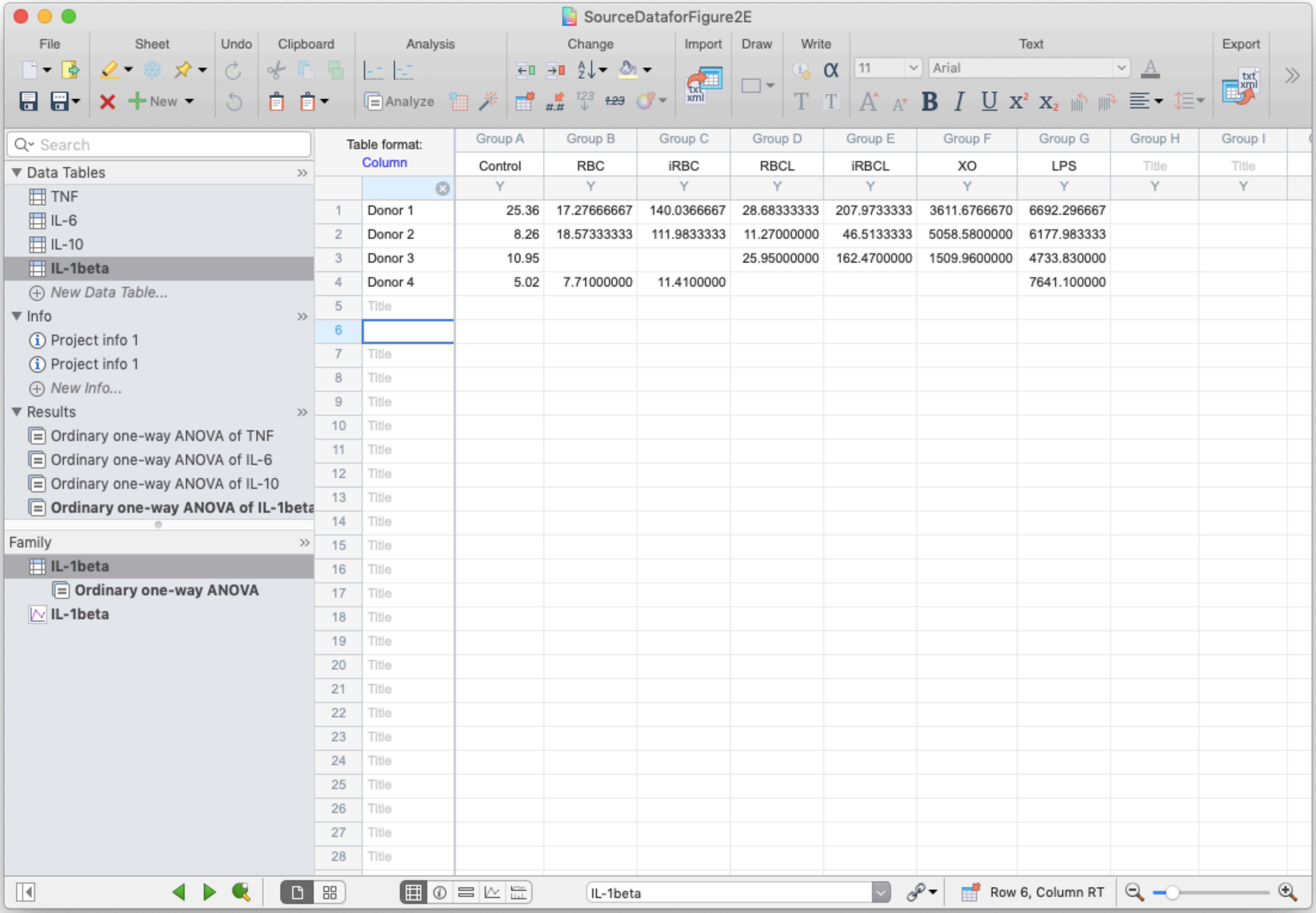

	Group A Control	Group B RBC	Group C iRBC	Group D RBCL	Group E iRBCL	Group F XO	Group G LPS
Donor 1	25.36	17.27666667	140.0366667	28.68333333	207.9733333	3611.6766670	6692.296667
Donor 2	8.26	18.57333333	111.9833333	11.27000000	46.5133333	5058.5800000	6177.983333
Donor 3	10.95			25.95000000	162.4700000	1509.9600000	4733.830000
Donor 4	5.02	7.71000000	11.4100000				7641.100000

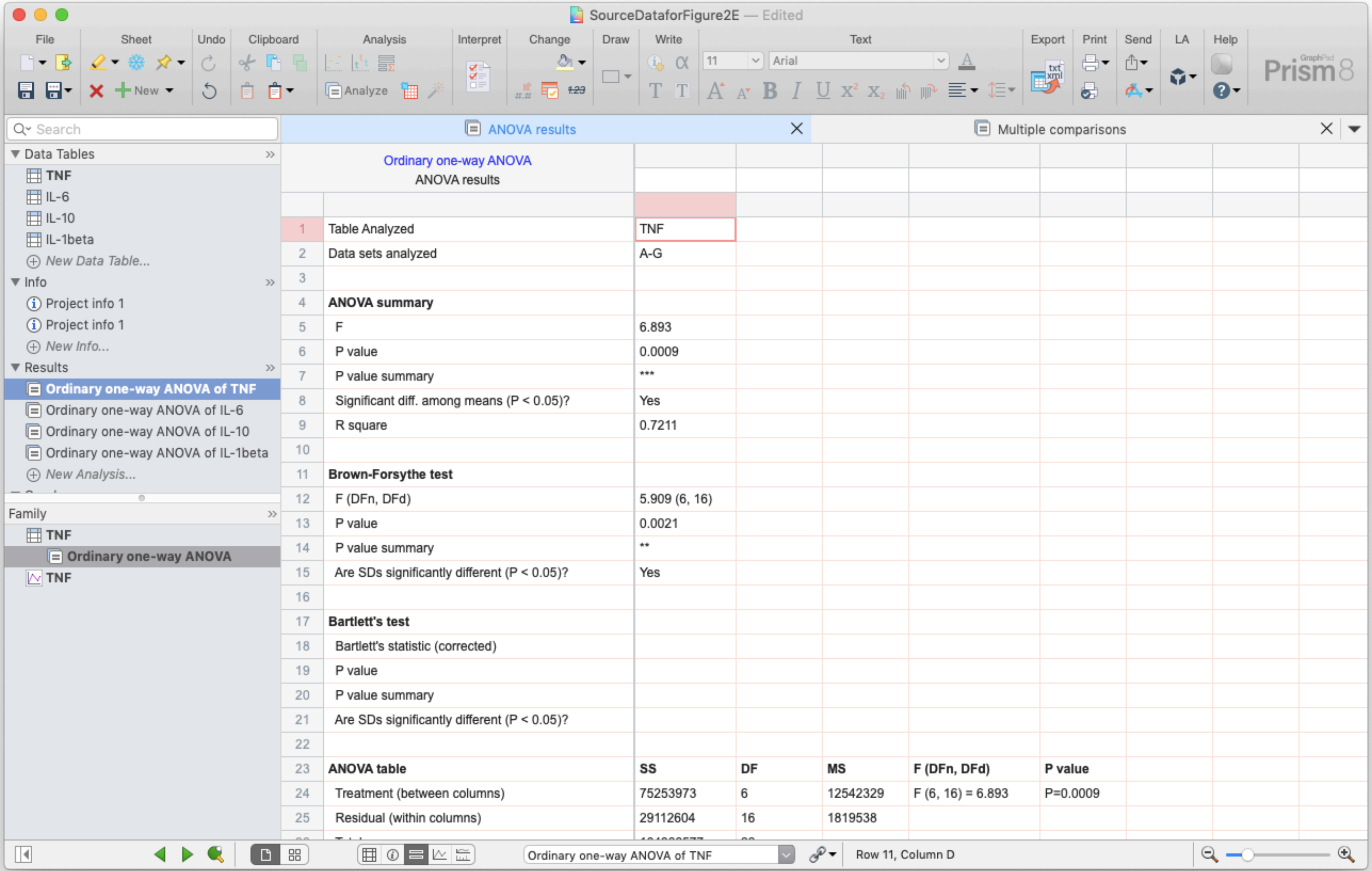

Ordinary one-way ANOVA
ANOVA results

1	Table Analyzed	TNF				
2	Data sets analyzed	A-G				
3						
4	**ANOVA summary**					
5	F	6.893				
6	P value	0.0009				
7	P value summary	***				
8	Significant diff. among means (P < 0.05)?	Yes				
9	R square	0.7211				
10						
11	**Brown-Forsythe test**					
12	F (DFn, DFd)	5.909 (6, 16)				
13	P value	0.0021				
14	P value summary	**				
15	Are SDs significantly different (P < 0.05)?	Yes				
16						
17	**Bartlett's test**					
18	Bartlett's statistic (corrected)					
19	P value					
20	P value summary					
21	Are SDs significantly different (P < 0.05)?					
22						
23	**ANOVA table**	**SS**	**DF**	**MS**	**F (DFn, DFd)**	**P value**
24	Treatment (between columns)	75253973	6	12542329	F (6, 16) = 6.893	P=0.0009
25	Residual (within columns)	29112604	16	1819538		
26	[illegible]	[illegible]	[illegible]			

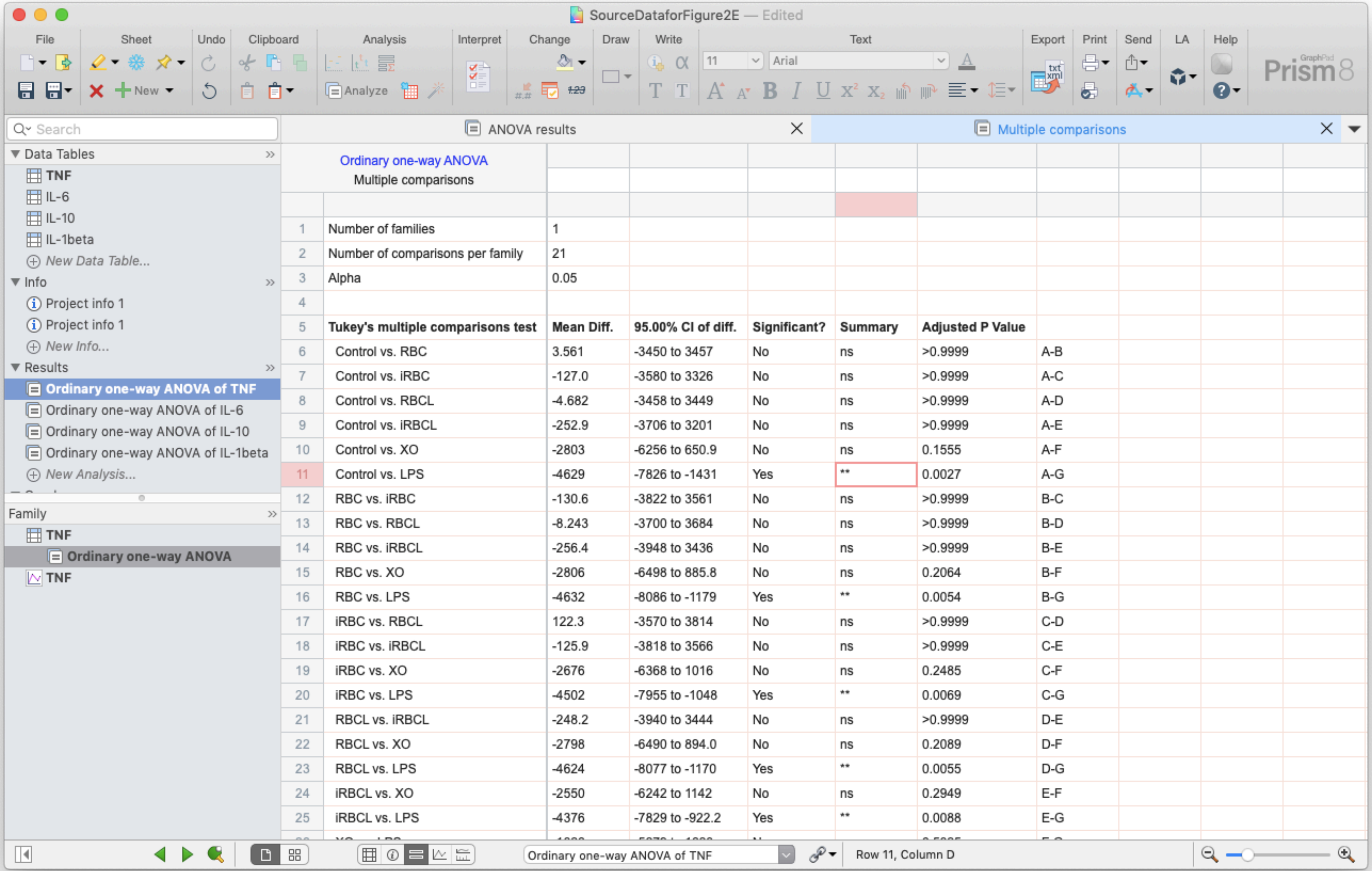

Ordinary one-way ANOVA
Multiple comparisons

1	Number of families	1					
2	Number of comparisons per family	21					
3	Alpha	0.05					
4							
5	**Tukey's multiple comparisons test**	**Mean Diff.**	**95.00% CI of diff.**	**Significant?**	**Summary**	**Adjusted P Value**	
6	Control vs. RBC	3.561	-3450 to 3457	No	ns	>0.9999	A-B
7	Control vs. iRBC	-127.0	-3580 to 3326	No	ns	>0.9999	A-C
8	Control vs. RBCL	-4.682	-3458 to 3449	No	ns	>0.9999	A-D
9	Control vs. iRBCL	-252.9	-3706 to 3201	No	ns	>0.9999	A-E
10	Control vs. XO	-2803	-6256 to 650.9	No	ns	0.1555	A-F
11	Control vs. LPS	-4629	-7826 to -1431	Yes	**	0.0027	A-G
12	RBC vs. iRBC	-130.6	-3822 to 3561	No	ns	>0.9999	B-C
13	RBC vs. RBCL	-8.243	-3700 to 3684	No	ns	>0.9999	B-D
14	RBC vs. iRBCL	-256.4	-3948 to 3436	No	ns	>0.9999	B-E
15	RBC vs. XO	-2806	-6498 to 885.8	No	ns	0.2064	B-F
16	RBC vs. LPS	-4632	-8086 to -1179	Yes	**	0.0054	B-G
17	iRBC vs. RBCL	122.3	-3570 to 3814	No	ns	>0.9999	C-D
18	iRBC vs. iRBCL	-125.9	-3818 to 3566	No	ns	>0.9999	C-E
19	iRBC vs. XO	-2676	-6368 to 1016	No	ns	0.2485	C-F
20	iRBC vs. LPS	-4502	-7955 to -1048	Yes	**	0.0069	C-G
21	RBCL vs. iRBCL	-248.2	-3940 to 3444	No	ns	>0.9999	D-E
22	RBCL vs. XO	-2798	-6490 to 894.0	No	ns	0.2089	D-F
23	RBCL vs. LPS	-4624	-8077 to -1170	Yes	**	0.0055	D-G
24	iRBCL vs. XO	-2550	-6242 to 1142	No	ns	0.2949	E-F
25	iRBCL vs. LPS	-4376	-7829 to -922.2	Yes	**	0.0088	E-G
[illegible]	[illegible]	[illegible]	[illegible]	[illegible]	[illegible]	[illegible]	[illegible]

SourceDataforFigure2E — Edited

ANVOA results | Multiple comparisons

Ordinary one-way ANOVA
Multiple comparisons

25	iRBCL vs. LPS	-4376	-7829 to -922.2	Yes	**	0.0088	E-G		
26	XO vs. LPS	-1826	-5279 to 1628	No	ns	0.5825	F-G		
27									
28	**Test details**	**Mean 1**	**Mean 2**	**Mean Diff.**	**SE of diff.**	**n1**	**n2**	**q**	**DF**
29	Control vs. RBC	22.76	19.20	3.561	1030	4	3	0.004889	16
30	Control vs. iRBC	22.76	149.8	-127.0	1030	4	3	0.1743	16
31	Control vs. RBCL	22.76	27.44	-4.682	1030	4	3	0.006427	16
32	Control vs. iRBCL	22.76	275.6	-252.9	1030	4	3	0.3471	16
33	Control vs. XO	22.76	2825	-2803	1030	4	3	3.847	16
34	Control vs. LPS	22.76	4651	-4629	953.8	4	4	6.863	16
35	RBC vs. iRBC	19.20	149.8	-130.6	1101	3	3	0.1676	16
36	RBC vs. RBCL	19.20	27.44	-8.243	1101	3	3	0.01058	16
37	RBC vs. iRBCL	19.20	275.6	-256.4	1101	3	3	0.3293	16
38	RBC vs. XO	19.20	2825	-2806	1101	3	3	3.603	16
39	RBC vs. LPS	19.20	4651	-4632	1030	3	4	6.359	16
40	iRBC vs. RBCL	149.8	27.44	122.3	1101	3	3	0.1571	16
41	iRBC vs. iRBCL	149.8	275.6	-125.9	1101	3	3	0.1616	16
42	iRBC vs. XO	149.8	2825	-2676	1101	3	3	3.436	16
43	iRBC vs. LPS	149.8	4651	-4502	1030	3	4	6.179	16
44	RBCL vs. iRBCL	27.44	275.6	-248.2	1101	3	3	0.3187	16
45	RBCL vs. XO	27.44	2825	-2798	1101	3	3	3.593	16
46	RBCL vs. LPS	27.44	4651	-4624	1030	3	4	6.347	16
47	iRBCL vs. XO	275.6	2825	-2550	1101	3	3	3.274	16
48	iRBCL vs. LPS	275.6	4651	-4376	1030	3	4	6.007	16
49	XO vs. LPS	2825	4651	-1826	1030	3	4	2.507	16

Data Tables: TNF, IL-6, IL-10, IL-1beta
Results: Ordinary one-way ANOVA of TNF; Ordinary one-way ANOVA of IL-6; Ordinary one-way ANOVA of IL-10; Ordinary one-way ANOVA of IL-1beta

Ordinary one-way ANOVA of TNF — Row 11, Column D

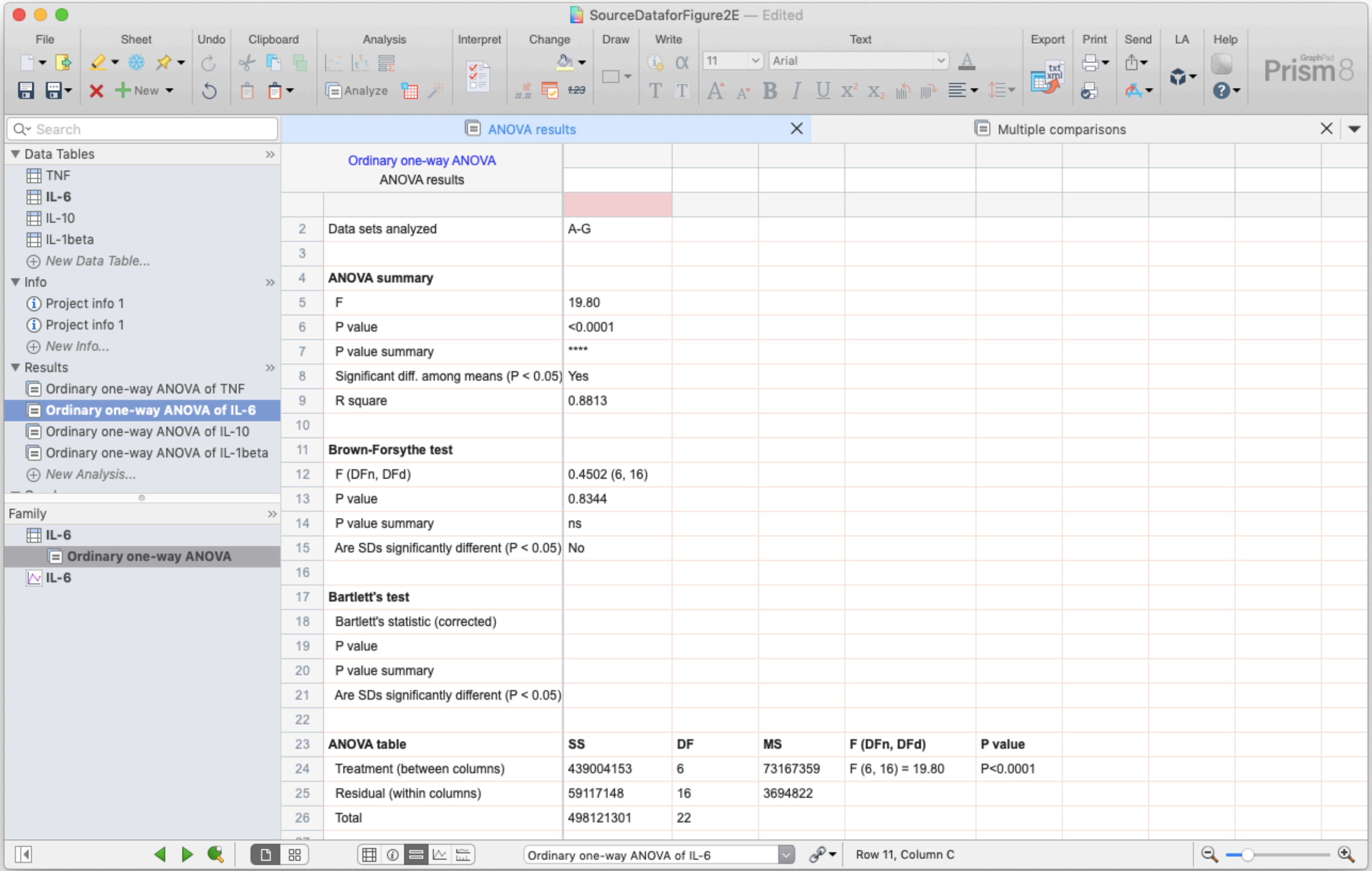

Ordinary one-way ANOVA

ANOVA results

2	Data sets analyzed	A-G				
3						
4	**ANOVA summary**					
5	F	19.80				
6	P value	<0.0001				
7	P value summary	****				
8	Significant diff. among means (P < 0.05)	Yes				
9	R square	0.8813				
10						
11	**Brown-Forsythe test**					
12	F (DFn, DFd)	0.4502 (6, 16)				
13	P value	0.8344				
14	P value summary	ns				
15	Are SDs significantly different (P < 0.05)	No				
16						
17	**Bartlett's test**					
18	Bartlett's statistic (corrected)					
19	P value					
20	P value summary					
21	Are SDs significantly different (P < 0.05)					
22						
23	**ANOVA table**	**SS**	**DF**	**MS**	**F (DFn, DFd)**	**P value**
24	Treatment (between columns)	439004153	6	73167359	F (6, 16) = 19.80	P<0.0001
25	Residual (within columns)	59117148	16	3694822		
26	Total	498121301	22			

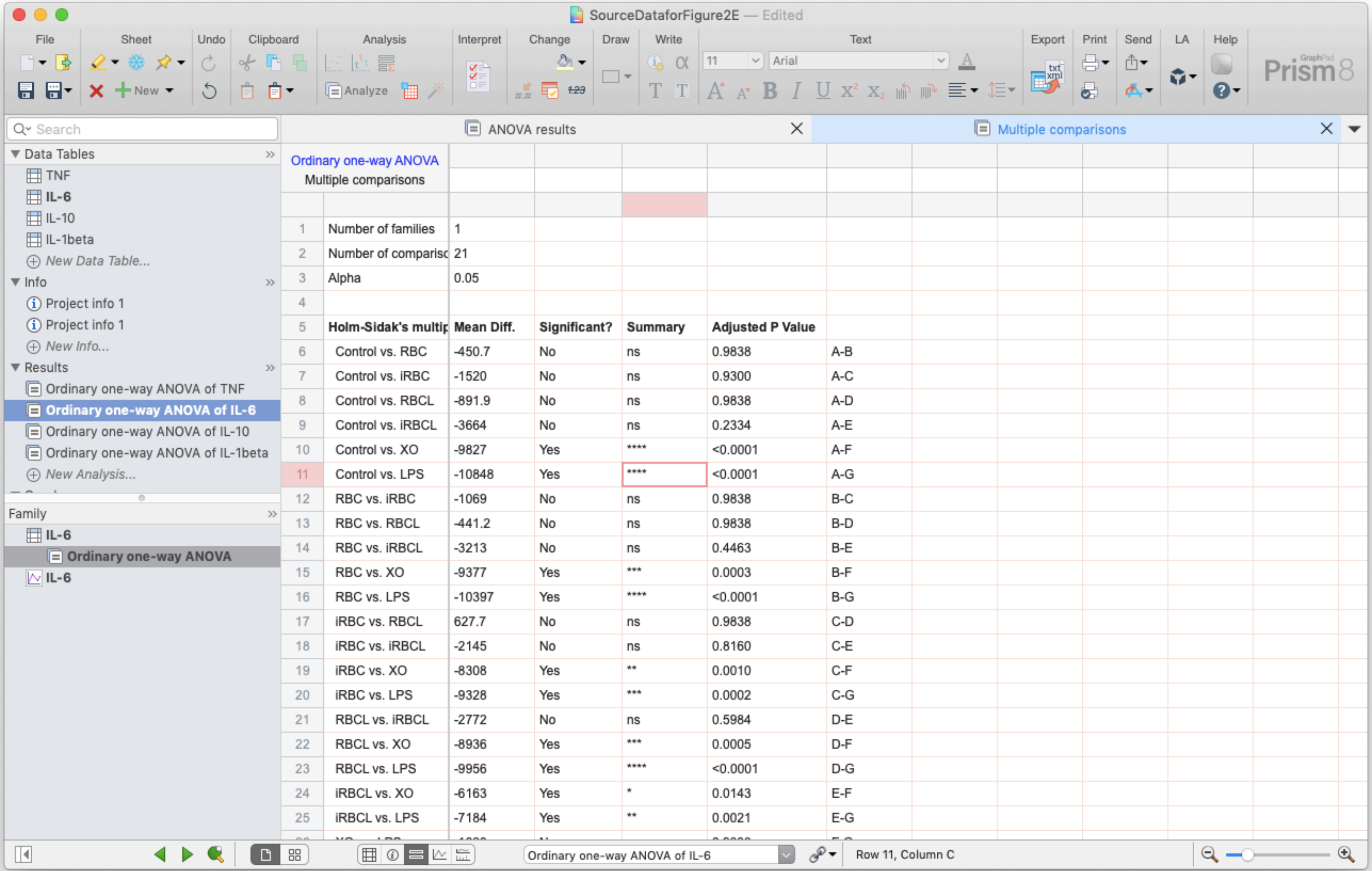

Ordinary one-way ANOVA
Multiple comparisons

1	Number of families	1				
2	Number of comparisc	21				
3	Alpha	0.05				
4						
5	**Holm-Sidak's multip**	**Mean Diff.**	**Significant?**	**Summary**	**Adjusted P Value**	
6	Control vs. RBC	-450.7	No	ns	0.9838	A-B
7	Control vs. iRBC	-1520	No	ns	0.9300	A-C
8	Control vs. RBCL	-891.9	No	ns	0.9838	A-D
9	Control vs. iRBCL	-3664	No	ns	0.2334	A-E
10	Control vs. XO	-9827	Yes	****	<0.0001	A-F
11	Control vs. LPS	-10848	Yes	****	<0.0001	A-G
12	RBC vs. iRBC	-1069	No	ns	0.9838	B-C
13	RBC vs. RBCL	-441.2	No	ns	0.9838	B-D
14	RBC vs. iRBCL	-3213	No	ns	0.4463	B-E
15	RBC vs. XO	-9377	Yes	***	0.0003	B-F
16	RBC vs. LPS	-10397	Yes	****	<0.0001	B-G
17	iRBC vs. RBCL	627.7	No	ns	0.9838	C-D
18	iRBC vs. iRBCL	-2145	No	ns	0.8160	C-E
19	iRBC vs. XO	-8308	Yes	**	0.0010	C-F
20	iRBC vs. LPS	-9328	Yes	***	0.0002	C-G
21	RBCL vs. iRBCL	-2772	No	ns	0.5984	D-E
22	RBCL vs. XO	-8936	Yes	***	0.0005	D-F
23	RBCL vs. LPS	-9956	Yes	****	<0.0001	D-G
24	iRBCL vs. XO	-6163	Yes	*	0.0143	E-F
25	iRBCL vs. LPS	-7184	Yes	**	0.0021	E-G
[illegible]	[illegible]	[illegible]	[illegible]		[illegible]	[illegible]

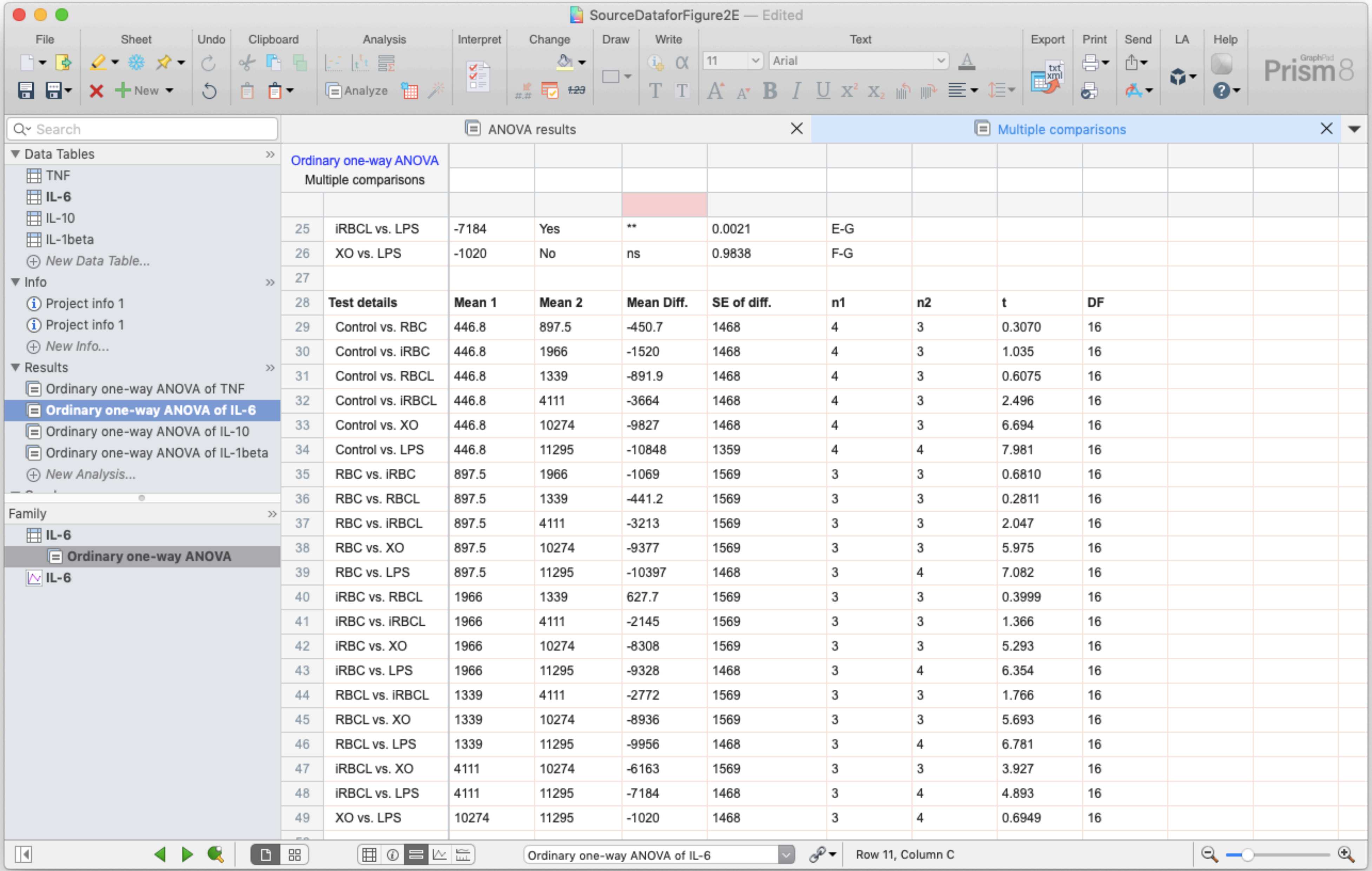

Ordinary one-way ANOVA
Multiple comparisons

25	iRBCL vs. LPS	-7184	Yes	**	0.0021	E-G			
26	XO vs. LPS	-1020	No	ns	0.9838	F-G			
27									
28	**Test details**	**Mean 1**	**Mean 2**	**Mean Diff.**	**SE of diff.**	**n1**	**n2**	**t**	**DF**
29	Control vs. RBC	446.8	897.5	-450.7	1468	4	3	0.3070	16
30	Control vs. iRBC	446.8	1966	-1520	1468	4	3	1.035	16
31	Control vs. RBCL	446.8	1339	-891.9	1468	4	3	0.6075	16
32	Control vs. iRBCL	446.8	4111	-3664	1468	4	3	2.496	16
33	Control vs. XO	446.8	10274	-9827	1468	4	3	6.694	16
34	Control vs. LPS	446.8	11295	-10848	1359	4	4	7.981	16
35	RBC vs. iRBC	897.5	1966	-1069	1569	3	3	0.6810	16
36	RBC vs. RBCL	897.5	1339	-441.2	1569	3	3	0.2811	16
37	RBC vs. iRBCL	897.5	4111	-3213	1569	3	3	2.047	16
38	RBC vs. XO	897.5	10274	-9377	1569	3	3	5.975	16
39	RBC vs. LPS	897.5	11295	-10397	1468	3	4	7.082	16
40	iRBC vs. RBCL	1966	1339	627.7	1569	3	3	0.3999	16
41	iRBC vs. iRBCL	1966	4111	-2145	1569	3	3	1.366	16
42	iRBC vs. XO	1966	10274	-8308	1569	3	3	5.293	16
43	iRBC vs. LPS	1966	11295	-9328	1468	3	4	6.354	16
44	RBCL vs. iRBCL	1339	4111	-2772	1569	3	3	1.766	16
45	RBCL vs. XO	1339	10274	-8936	1569	3	3	5.693	16
46	RBCL vs. LPS	1339	11295	-9956	1468	3	4	6.781	16
47	iRBCL vs. XO	4111	10274	-6163	1569	3	3	3.927	16
48	iRBCL vs. LPS	4111	11295	-7184	1468	3	4	4.893	16
49	XO vs. LPS	10274	11295	-1020	1468	3	4	0.6949	16

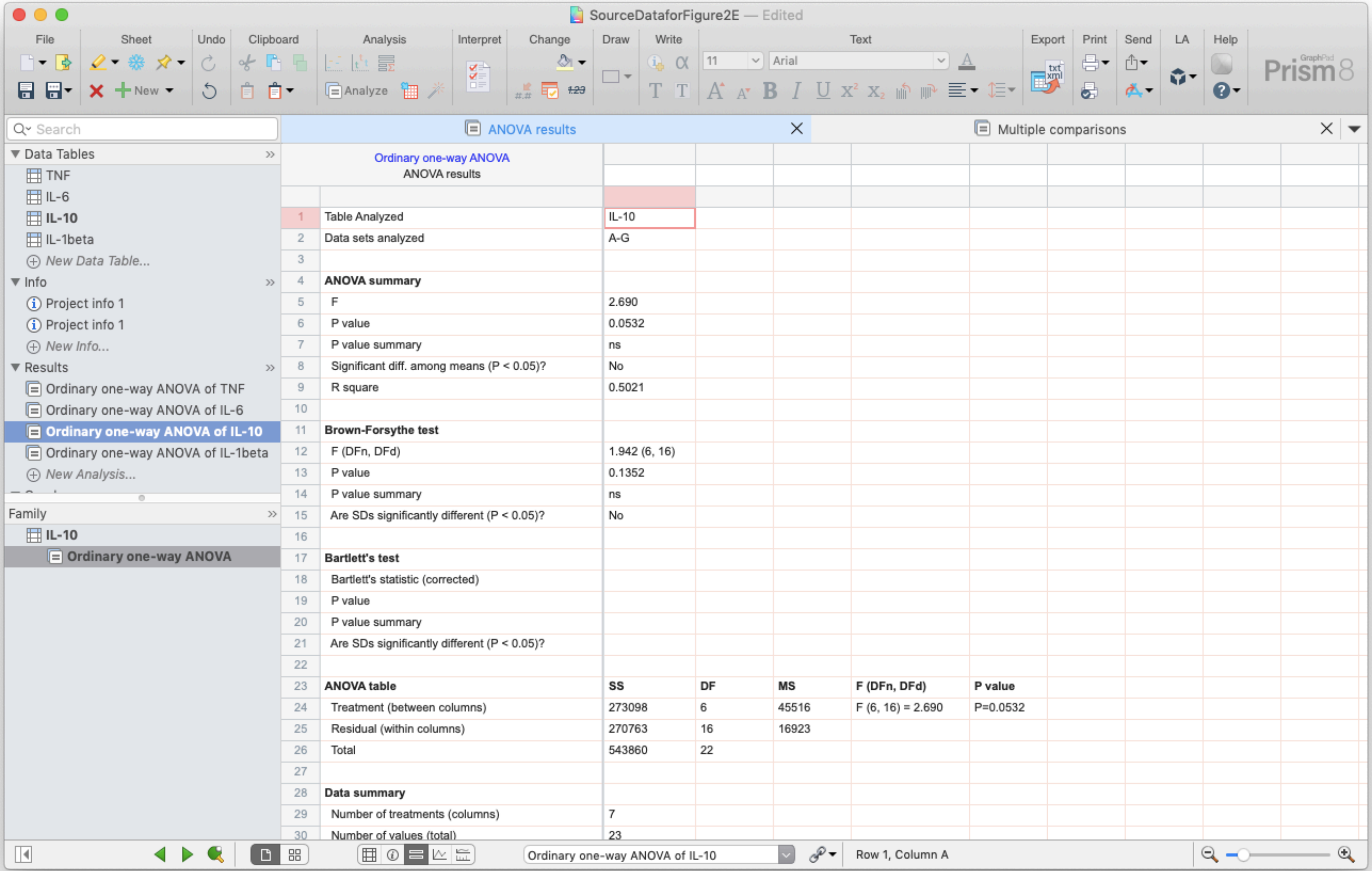

Ordinary one-way ANOVA
ANOVA results

1	Table Analyzed	IL-10				
2	Data sets analyzed	A-G				
3						
4	**ANOVA summary**					
5	F	2.690				
6	P value	0.0532				
7	P value summary	ns				
8	Significant diff. among means (P < 0.05)?	No				
9	R square	0.5021				
10						
11	**Brown-Forsythe test**					
12	F (DFn, DFd)	1.942 (6, 16)				
13	P value	0.1352				
14	P value summary	ns				
15	Are SDs significantly different (P < 0.05)?	No				
16						
17	**Bartlett's test**					
18	Bartlett's statistic (corrected)					
19	P value					
20	P value summary					
21	Are SDs significantly different (P < 0.05)?					
22						
23	**ANOVA table**	**SS**	**DF**	**MS**	**F (DFn, DFd)**	**P value**
24	Treatment (between columns)	273098	6	45516	F (6, 16) = 2.690	P=0.0532
25	Residual (within columns)	270763	16	16923		
26	Total	543860	22			
27						
28	**Data summary**					
29	Number of treatments (columns)	7				
30	Number of values (total)	23				

SourceDataforFigure2E — Edited

ANOVA results | Multiple comparisons

Data Tables: TNF, IL-6, **IL-10**, IL-1beta, New Data Table...

Info: Project info 1, Project info 1, New Info...

Results: Ordinary one-way ANOVA of TNF, Ordinary one-way ANOVA of IL-6, **Ordinary one-way ANOVA of IL-10**, Ordinary one-way ANOVA of IL-1beta, New Analysis...

Family: **IL-10**, **Ordinary one-way ANOVA**

Ordinary one-way ANOVA
Multiple comparisons

1	Number of families	1							
2	Number of comparisons per family	21							
3	Alpha	0.05							
4									
5	**Tukey's multiple comparisons test**	**Mean Diff.**	**95.00% CI of diff.**	**Significant?**	**Summary**	**Adjusted P Value**			
6	Control vs. RBC	-1.727	-334.8 to 331.3	No	ns	>0.9999	A-B		
7	Control vs. iRBC	-13.06	-346.1 to 320.0	No	ns	>0.9999	A-C		
8	Control vs. RBCL	-0.7064	-333.8 to 332.3	No	ns	>0.9999	A-D		
9	Control vs. iRBCL	-11.99	-345.0 to 321.1	No	ns	>0.9999	A-E		
10	Control vs. XO	-180.6	-513.7 to 152.4	No	ns	0.5555	A-F		
11	Control vs. LPS	-275.7	-584.0 to 32.69	No	ns	0.0962	A-G		
12	RBC vs. iRBC	-11.33	-367.4 to 344.7	No	ns	>0.9999	B-C		
13	RBC vs. RBCL	1.021	-355.0 to 357.1	No	ns	>0.9999	B-D		
14	RBC vs. iRBCL	-10.26	-366.3 to 345.8	No	ns	>0.9999	B-E		
15	RBC vs. XO	-178.9	-534.9 to 177.2	No	ns	0.6348	B-F		
16	RBC vs. LPS	-273.9	-607.0 to 59.13	No	ns	0.1462	B-G		
17	iRBC vs. RBCL	12.35	-343.7 to 368.4	No	ns	>0.9999	C-D		
18	iRBC vs. iRBCL	1.069	-355.0 to 357.1	No	ns	>0.9999	C-E		
19	iRBC vs. XO	-167.6	-523.6 to 188.5	No	ns	0.6973	C-F		
20	iRBC vs. LPS	-262.6	-595.6 to 70.46	No	ns	0.1769	C-G		
21	RBCL vs. iRBCL	-11.28	-367.3 to 344.8	No	ns	>0.9999	D-E		
22	RBCL vs. XO	-179.9	-536.0 to 176.1	No	ns	0.6291	D-F		
23	RBCL vs. LPS	-274.9	-608.0 to 58.11	No	ns	0.1436	D-G		
24	iRBCL vs. XO	-168.6	-524.7 to 187.4	No	ns	0.6915	E-F		
25	iRBCL vs. LPS	-263.7	-596.7 to 69.39	No	ns	0.1738	E-G		
26	XO vs. LPS	-95.03	-428.1 to 238.0	No	ns	0.9565	F-G		
27									
28	**Test details**	**Mean 1**	**Mean 2**	**Mean Diff.**	**SE of diff.**	**n1**	**n2**	**q**	**DF**
29	Control vs. RBC	1.629	3.357	-1.727	99.36	4	3	0.02459	16
30	Control vs. iRBC	1.629	14.69	-13.06	99.36	4	3	0.1859	16

Ordinary one-way ANOVA of IL-10 — Row 1, Column A

SourceDataforFigure2E — Edited

ANOVA results | Multiple comparisons

Data Tables: TNF, IL-6, IL-10, IL-1beta
Results: Ordinary one-way ANOVA of TNF; Ordinary one-way ANOVA of IL-6; Ordinary one-way ANOVA of IL-10; Ordinary one-way ANOVA of IL-1beta

Ordinary one-way ANOVA
Multiple comparisons

25	iRBCL vs. LPS	-263.7	-596.7 to 69.39	No	ns	0.1738	E-G		
26	XO vs. LPS	-95.03	-428.1 to 238.0	No	ns	0.9565	F-G		
27									
28	**Test details**	**Mean 1**	**Mean 2**	**Mean Diff.**	**SE of diff.**	**n1**	**n2**	**q**	**DF**
29	Control vs. RBC	1.629	3.357	-1.727	99.36	4	3	0.02459	16
30	Control vs. iRBC	1.629	14.69	-13.06	99.36	4	3	0.1859	16
31	Control vs. RBCL	1.629	2.336	-0.7064	99.36	4	3	0.01005	16
32	Control vs. iRBCL	1.629	13.62	-11.99	99.36	4	3	0.1707	16
33	Control vs. XO	1.629	182.2	-180.6	99.36	4	3	2.571	16
34	Control vs. LPS	1.629	277.3	-275.7	91.99	4	4	4.238	16
35	RBC vs. iRBC	3.357	14.69	-11.33	106.2	3	3	0.1509	16
36	RBC vs. RBCL	3.357	2.336	1.021	106.2	3	3	0.01360	16
37	RBC vs. iRBCL	3.357	13.62	-10.26	106.2	3	3	0.1367	16
38	RBC vs. XO	3.357	182.2	-178.9	106.2	3	3	2.382	16
39	RBC vs. LPS	3.357	277.3	-273.9	99.36	3	4	3.899	16
40	iRBC vs. RBCL	14.69	2.336	12.35	106.2	3	3	0.1645	16
41	iRBC vs. iRBCL	14.69	13.62	1.069	106.2	3	3	0.01423	16
42	iRBC vs. XO	14.69	182.2	-167.6	106.2	3	3	2.231	16
43	iRBC vs. LPS	14.69	277.3	-262.6	99.36	3	4	3.738	16
44	RBCL vs. iRBCL	2.336	13.62	-11.28	106.2	3	3	0.1502	16
45	RBCL vs. XO	2.336	182.2	-179.9	106.2	3	3	2.395	16
46	RBCL vs. LPS	2.336	277.3	-274.9	99.36	3	4	3.914	16
47	iRBCL vs. XO	13.62	182.2	-168.6	106.2	3	3	2.245	16
48	iRBCL vs. LPS	13.62	277.3	-263.7	99.36	3	4	3.753	16
49	XO vs. LPS	182.2	277.3	-95.03	99.36	3	4	1.353	16

Ordinary one-way ANOVA of IL-10 — Row 1, Column A

SourceDataforFigure2E — Edited

ANOVA results | Multiple comparisons

Ordinary one-way ANOVA
ANOVA results

1	Table Analyzed	IL-1beta				
2	Data sets analyzed	A-G				
3						
4	**ANOVA summary**					
5	F	33.74				
6	P value	<0.0001				
7	P value summary	****				
8	Significant diff. among means (P < 0.05)?	Yes				
9	R square	0.9267				
10						
11	**Brown-Forsythe test**					
12	F (DFn, DFd)	3.309 (6, 16)				
13	P value	0.0259				
14	P value summary	*				
15	Are SDs significantly different (P < 0.05)?	Yes				
16						
17	**Bartlett's test**					
18	Bartlett's statistic (corrected)					
19	P value					
20	P value summary					
21	Are SDs significantly different (P < 0.05)?					
22						
23	**ANOVA table**	**SS**	**DF**	**MS**	**F (DFn, DFd)**	**P value**
24	Treatment (between columns)	136779514	6	22796586	F (6, 16) = 33.74	P<0.0001
25	Residual (within columns)	10811025	16	675689		
26	Total	147590540	22			
27						
28	**Data summary**					
29	Number of treatments (columns)	7				
30	Number of values (total)	23				

Ordinary one-way ANOVA of IL-1beta — Row 11, Column D

SourceDataforFigure2E — Edited

ANOVA results | Multiple comparisons

Ordinary one-way ANOVA
Multiple comparisons

1	Number of families	1							
2	Number of comparisons pe	21							
3	Alpha	0.05							
4									
5	**Tukey's multiple compari**	**Mean Diff.**	**95.00% CI of diff.**	**Significant?**	**Summary**	**Adjusted P Value**			
6	Control vs. RBC	-2.123	-2107 to 2102	No	ns	>0.9999	A-B		
7	Control vs. iRBC	-75.41	-2180 to 2029	No	ns	>0.9999	A-C		
8	Control vs. RBCL	-9.570	-2114 to 2095	No	ns	>0.9999	A-D		
9	Control vs. iRBCL	-126.6	-2231 to 1978	No	ns	>0.9999	A-E		
10	Control vs. XO	-3381	-5486 to -1276	Yes	***	0.0010	A-F		
11	Control vs. LPS	-6299	-8247 to -4351	Yes	****	<0.0001	A-G		
12	RBC vs. iRBC	-73.29	-2323 to 2177	No	ns	>0.9999	B-C		
13	RBC vs. RBCL	-7.448	-2257 to 2242	No	ns	>0.9999	B-D		
14	RBC vs. iRBCL	-124.5	-2374 to 2125	No	ns	>0.9999	B-E		
15	RBC vs. XO	-3379	-5629 to -1129	Yes	**	0.0019	B-F		
16	RBC vs. LPS	-6297	-8401 to -4192	Yes	****	<0.0001	B-G		
17	iRBC vs. RBCL	65.84	-2184 to 2316	No	ns	>0.9999	C-D		
18	iRBC vs. iRBCL	-51.18	-2301 to 2199	No	ns	>0.9999	C-E		
19	iRBC vs. XO	-3306	-5555 to -1056	Yes	**	0.0023	C-F		
20	iRBC vs. LPS	-6223	-8328 to -4119	Yes	****	<0.0001	C-G		
21	RBCL vs. iRBCL	-117.0	-2367 to 2133	No	ns	>0.9999	D-E		
22	RBCL vs. XO	-3371	-5621 to -1122	Yes	**	0.0019	D-F		
23	RBCL vs. LPS	-6289	-8394 to -4185	Yes	****	<0.0001	D-G		
24	iRBCL vs. XO	-3254	-5504 to -1005	Yes	**	0.0027	E-F		
25	iRBCL vs. LPS	-6172	-8277 to -4068	Yes	****	<0.0001	E-G		
26	XO vs. LPS	-2918	-5022 to -813.4	Yes	**	0.0040	F-G		
27									
28	**Test details**	**Mean 1**	**Mean 2**	**Mean Diff.**	**SE of diff.**	**n1**	**n2**	**q**	**DF**
29	Control vs. RBC	12.40	14.52	-2.123	627.8	4	3	0.004781	16
30	Control vs. iRBC	12.40	87.81	-75.41	627.8	4	3	0.1699	16

Ordinary one-way ANOVA of IL-1beta — Row 2, Column B

SourceDataforFigure2E — Edited

ANOVA results | Multiple comparisons

Ordinary one-way ANOVA
Multiple comparisons

22	RBCL vs. XO	-3371	-5621 to -1122	Yes	**	0.0019	D-F		
23	RBCL vs. LPS	-6289	-8394 to -4185	Yes	****	<0.0001	D-G		
24	iRBCL vs. XO	-3254	-5504 to -1005	Yes	**	0.0027	E-F		
25	iRBCL vs. LPS	-6172	-8277 to -4068	Yes	****	<0.0001	E-G		
26	XO vs. LPS	-2918	-5022 to -813.4	Yes	**	0.0040	F-G		
27									
28	**Test details**	**Mean 1**	**Mean 2**	**Mean Diff.**	**SE of diff.**	**n1**	**n2**	**q**	**DF**
29	Control vs. RBC	12.40	14.52	-2.123	627.8	4	3	0.004781	16
30	Control vs. iRBC	12.40	87.81	-75.41	627.8	4	3	0.1699	16
31	Control vs. RBCL	12.40	21.97	-9.570	627.8	4	3	0.02156	16
32	Control vs. iRBCL	12.40	139.0	-126.6	627.8	4	3	0.2852	16
33	Control vs. XO	12.40	3393	-3381	627.8	4	3	7.616	16
34	Control vs. LPS	12.40	6311	-6299	581.2	4	4	15.33	16
35	RBC vs. iRBC	14.52	87.81	-73.29	671.2	3	3	0.1544	16
36	RBC vs. RBCL	14.52	21.97	-7.448	671.2	3	3	0.01569	16
37	RBC vs. iRBCL	14.52	139.0	-124.5	671.2	3	3	0.2623	16
38	RBC vs. XO	14.52	3393	-3379	671.2	3	3	7.120	16
39	RBC vs. LPS	14.52	6311	-6297	627.8	3	4	14.18	16
40	iRBC vs. RBCL	87.81	21.97	65.84	671.2	3	3	0.1387	16
41	iRBC vs. iRBCL	87.81	139.0	-51.18	671.2	3	3	0.1078	16
42	iRBC vs. XO	87.81	3393	-3306	671.2	3	3	6.965	16
43	iRBC vs. LPS	87.81	6311	-6223	627.8	3	4	14.02	16
44	RBCL vs. iRBCL	21.97	139.0	-117.0	671.2	3	3	0.2466	16
45	RBCL vs. XO	21.97	3393	-3371	671.2	3	3	7.104	16
46	RBCL vs. LPS	21.97	6311	-6289	627.8	3	4	14.17	16
47	iRBCL vs. XO	139.0	3393	-3254	671.2	3	3	6.857	16
48	iRBCL vs. LPS	139.0	6311	-6172	627.8	3	4	13.90	16
49	XO vs. LPS	3393	6311	-2918	627.8	3	4	6.573	16

Ordinary one-way ANOVA of IL-1beta — Row 2, Column B
