# Supplementary material for: Malaria inflammation by xanthine oxidase‐produced reactive oxygen species
Source: EMBO Mol Med. 2019 Jul 2;11(8):e9903. doi: 10.15252/emmm.201809903 (PMC6685105; doi:10.15252/emmm.201809903)
Supplement: Supplementary file 6 — Source Data for Figure 3 [file EMMM-11-e9903-s004.pdf]

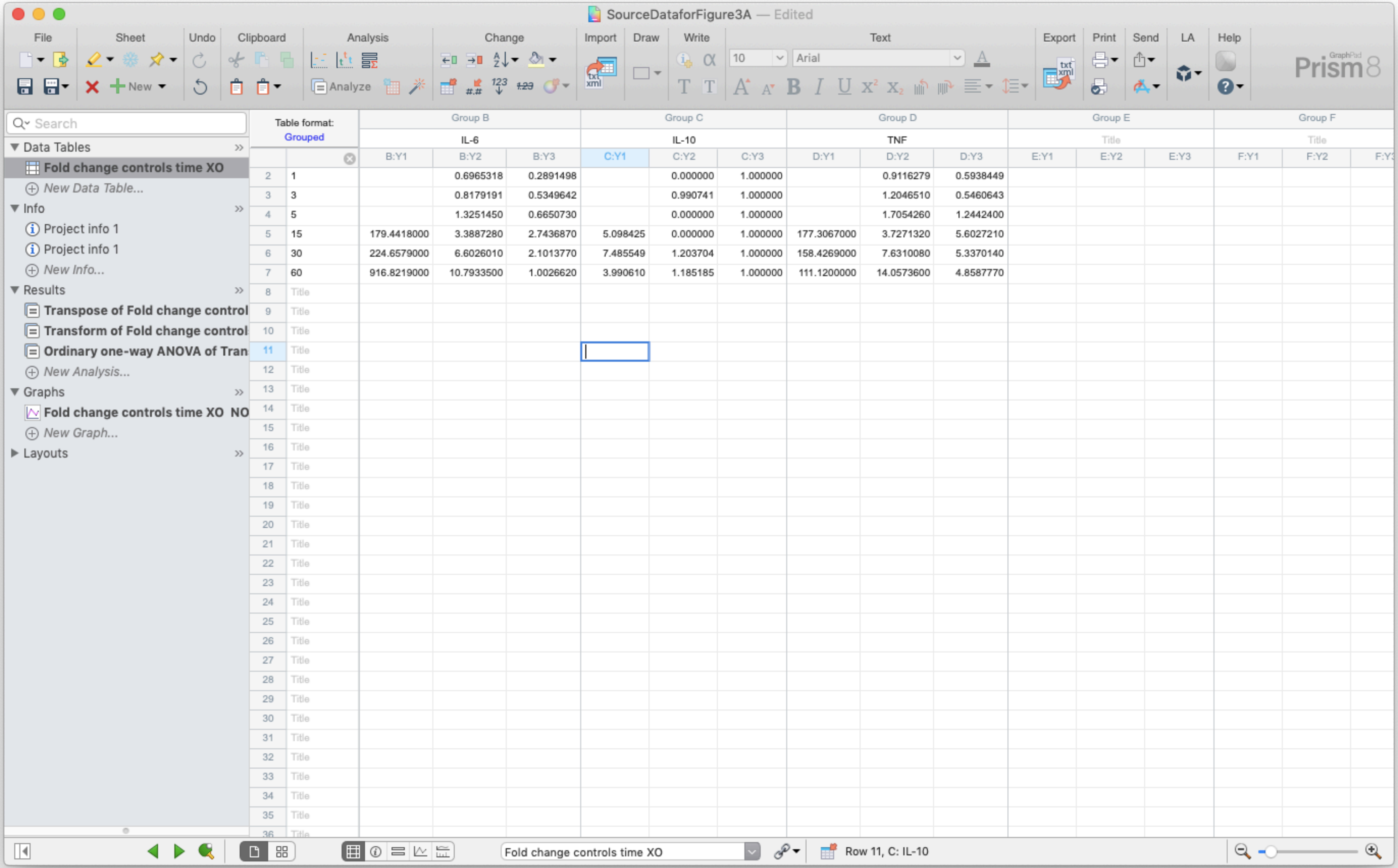

Table format: Grouped		Group B			Group C			Group D		
		IL-6			IL-10			TNF		
		B:Y1	B:Y2	B:Y3	C:Y1	C:Y2	C:Y3	D:Y1	D:Y2	D:Y3
2	1		0.6965318	0.2891498		0.000000	1.000000		0.9116279	0.5938449
3	3		0.8179191	0.5349642		0.990741	1.000000		1.2046510	0.5460643
4	5		1.3251450	0.6650730		0.000000	1.000000		1.7054260	1.2442400
5	15	179.4418000	3.3887280	2.7436870	5.098425	0.000000	1.000000	177.3067000	3.7271320	5.6027210
6	30	224.6579000	6.6026010	2.1013770	7.485549	1.203704	1.000000	158.4269000	7.6310080	5.3370140
7	60	916.8219000	10.7933500	1.0026620	3.990610	1.185185	1.000000	111.1200000	14.0573600	4.8587770

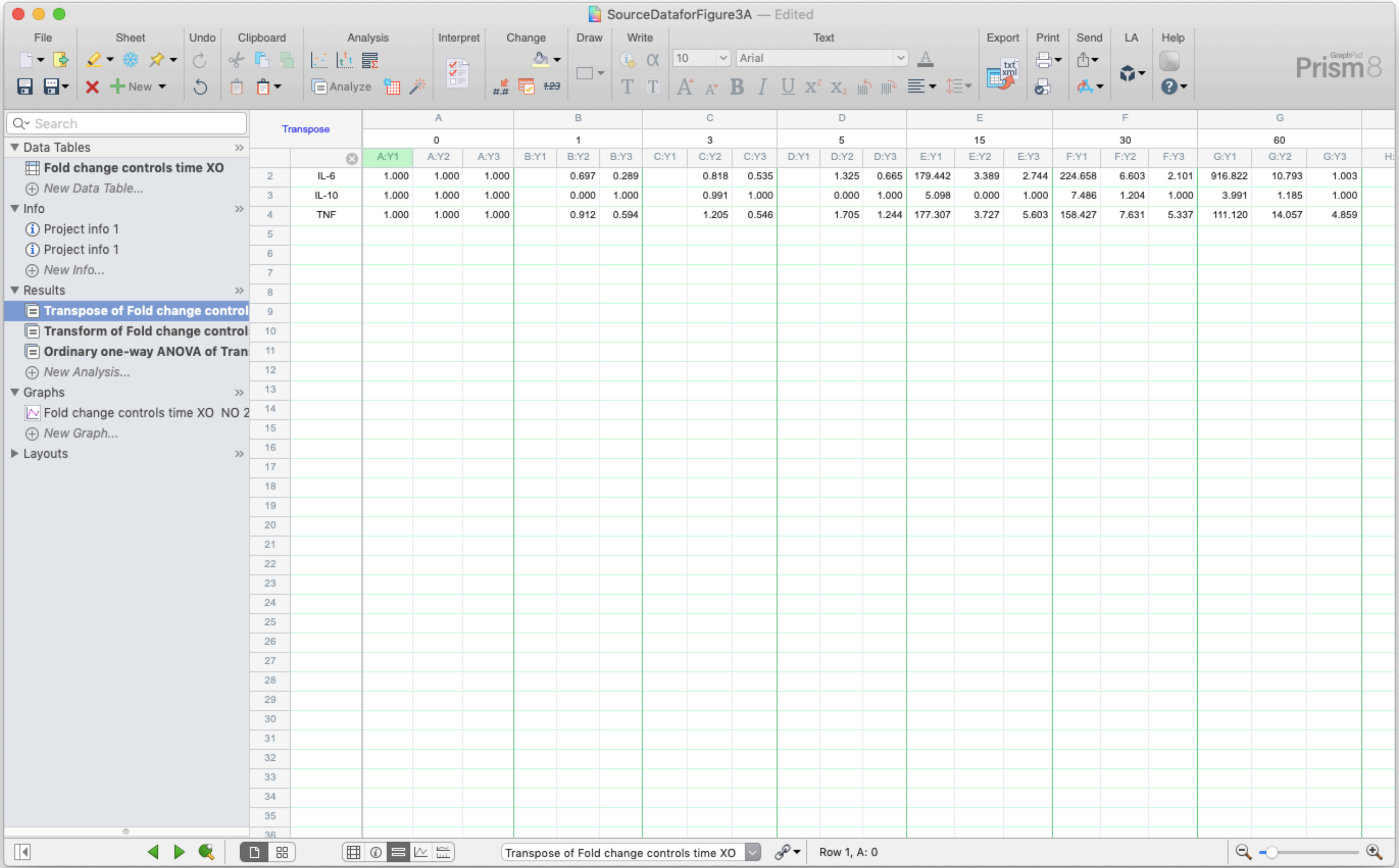

Transpose	A			B			C			D			E			F			G		
	0			1			3			5			15			30			60		
	A:Y1	A:Y2	A:Y3	B:Y1	B:Y2	B:Y3	C:Y1	C:Y2	C:Y3	D:Y1	D:Y2	D:Y3	E:Y1	E:Y2	E:Y3	F:Y1	F:Y2	F:Y3	G:Y1	G:Y2	G:Y3
IL-6	1.000	1.000	1.000		0.697	0.289		0.818	0.535		1.325	0.665	179.442	3.389	2.744	224.658	6.603	2.101	916.822	10.793	1.003
IL-10	1.000	1.000	1.000		0.000	1.000		0.991	1.000		0.000	1.000	5.098	0.000	1.000	7.486	1.204	1.000	3.991	1.185	1.000
TNF	1.000	1.000	1.000		0.912	0.594		1.205	0.546		1.705	1.244	177.307	3.727	5.603	158.427	7.631	5.337	111.120	14.057	4.859

SourceDataforFigure3A — Edited

File | Sheet | Undo | Clipboard | Analysis | Interpret | Change | Draw | Write | Text | Export | Print | Send | LA | Help

GraphPad Prism 8

Search

- Data Tables
  - Fold change controls time XO
  - New Data Table...
- Info
  - Project info 1
  - Project info 1
  - New Info...
- Results
  - Transpose of Fold change control
  - Transform of Fold change control
  - Ordinary one-way ANOVA of Tran
  - New Analysis...
- Graphs
  - Fold change controls time XO NO 2
  - New Graph...
- Layouts

Transform	A			B			C			D		
	0			1			3			5		
	A:Y1	A:Y2	A:Y3	B:Y1	B:Y2	B:Y3	C:Y1	C:Y2	C:Y3	D:Y1	D:Y2	D:Y3
1 IL-1beta	0.000	0.000	0.000		0.000	-0.012		0.000	0.014		0.000	0.005
2 IL-6	0.000	0.000	0.000		-0.157	-0.539		-0.087	-0.272		0.122	-0.177
3 IL-10	0.000	0.000	0.000			0.000		-0.004	0.000			0.000
4 TNF	0.000	0.000	0.000		-0.040	-0.226		0.081	-0.263		0.232	0.095

Transform	E			F			G			H
	15			30			60			
	E:Y1	E:Y2	E:Y3	F:Y1	F:Y2	F:Y3	G:Y1	G:Y2	G:Y3	H:Y1
1 IL-1beta	0.907	0.000	0.290	0.670	0.000	0.007	0.637	0.000	0.016	
2 IL-6	2.254	0.530	0.438	2.352	0.820	0.323	2.962	1.033	0.001	
3 IL-10	0.707		0.000	0.874	0.081	0.000	0.601	0.074	0.000	
4 TNF	2.249	0.571	0.748	2.200	0.883	0.727	2.046	1.148	0.687	

Transform of Fold change controls time XO | Row 6, Column RT

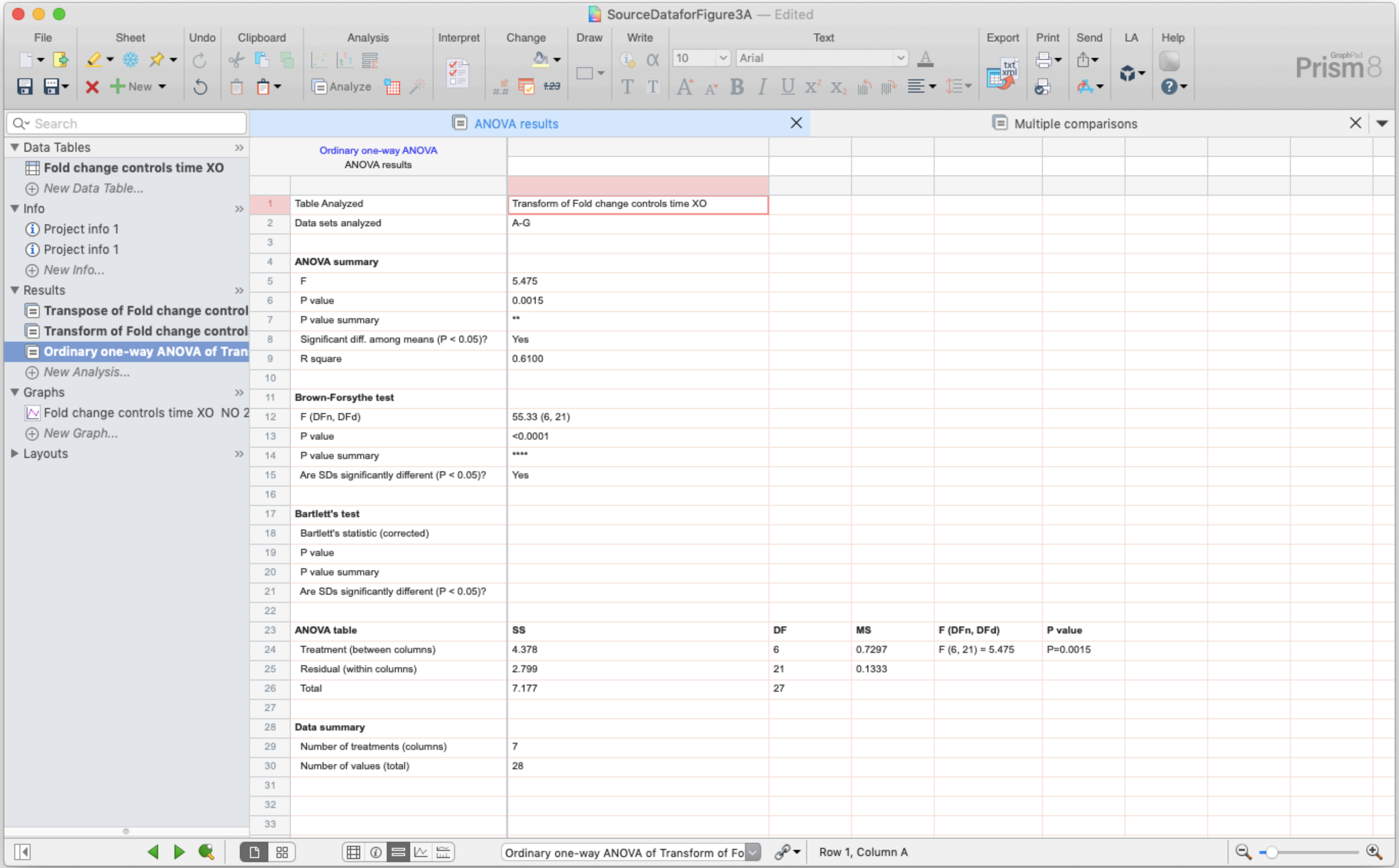

Ordinary one-way ANOVA
ANOVA results

1	Table Analyzed	Transform of Fold change controls time XO				
2	Data sets analyzed	A-G				
3						
4	**ANOVA summary**					
5	F	5.475				
6	P value	0.0015				
7	P value summary	**				
8	Significant diff. among means (P < 0.05)?	Yes				
9	R square	0.6100				
10						
11	**Brown-Forsythe test**					
12	F (DFn, DFd)	55.33 (6, 21)				
13	P value	<0.0001				
14	P value summary	****				
15	Are SDs significantly different (P < 0.05)?	Yes				
16						
17	**Bartlett's test**					
18	Bartlett's statistic (corrected)					
19	P value					
20	P value summary					
21	Are SDs significantly different (P < 0.05)?					
22						
23	**ANOVA table**	**SS**	**DF**	**MS**	**F (DFn, DFd)**	**P value**
24	Treatment (between columns)	4.378	6	0.7297	F (6, 21) = 5.475	P=0.0015
25	Residual (within columns)	2.799	21	0.1333		
26	Total	7.177	27			
27						
28	**Data summary**					
29	Number of treatments (columns)	7				
30	Number of values (total)	28				
31						
32						
33						

SourceDataforFigure3A — Edited

ANOVA results | Multiple comparisons

Ordinary one-way ANOVA
Multiple comparisons

1	Number of families	1							
2	Number of comparisons per family	21							
3	Alpha	0.05							
4									
5	**Tukey's multiple comparisons tes**	**Mean Diff.**	**95.00% CI of diff.**	**Significant?**	**Summary**	**Adjusted P Value**			
6	0 vs. 1	0.1218	-0.7174 to 0.9610	No	ns	0.9990	A-B		
7	0 vs. 3	0.06632	-0.7729 to 0.9055	No	ns	>0.9999	A-C		
8	0 vs. 5	-0.03464	-0.8738 to 0.8045	No	ns	>0.9999	A-D		
9	0 vs. 15	-0.7542	-1.593 to 0.08503	No	ns	0.0969	A-E		
10	0 vs. 30	-0.7446	-1.584 to 0.09459	No	ns	0.1041	A-F		
11	0 vs. 60	-0.7671	-1.606 to 0.07211	No	ns	0.0879	A-G		
12	1 vs. 3	-0.05550	-0.8947 to 0.7837	No	ns	>0.9999	B-C		
13	1 vs. 5	-0.1565	-0.9956 to 0.6827	No	ns	0.9959	B-D		
14	1 vs. 15	-0.8760	-1.715 to -0.03679	Yes	*	0.0371	B-E		
15	1 vs. 30	-0.8664	-1.706 to -0.02722	Yes	*	0.0401	B-F		
16	1 vs. 60	-0.8889	-1.728 to -0.04970	Yes	*	0.0333	B-G		
17	3 vs. 5	-0.1010	-0.9401 to 0.7382	No	ns	0.9996	C-D		
18	3 vs. 15	-0.8205	-1.660 to 0.01871	No	ns	0.0581	C-E		
19	3 vs. 30	-0.8109	-1.650 to 0.02827	No	ns	0.0626	C-F		
20	3 vs. 60	-0.8334	-1.673 to 0.005794	No	ns	0.0524	C-G		
21	5 vs. 15	-0.7195	-1.559 to 0.1197	No	ns	0.1252	D-E		
22	5 vs. 30	-0.7099	-1.549 to 0.1292	No	ns	0.1341	D-F		
23	5 vs. 60	-0.7324	-1.572 to 0.1068	No	ns	0.1139	D-G		
24	15 vs. 30	0.009566	-0.8296 to 0.8487	No	ns	>0.9999	E-F		
25	15 vs. 60	-0.01291	-0.8521 to 0.8263	No	ns	>0.9999	E-G		
26	30 vs. 60	-0.02248	-0.8617 to 0.8167	No	ns	>0.9999	F-G		
27									
28	**Test details**	**Mean 1**	**Mean 2**	**Mean Diff.**	**SE of diff.**	**n1**	**n2**	**q**	**DF**
29	0 vs. 1	0.000	-0.1218	0.1218	0.2581	4	4	0.6674	21
30	0 vs. 3	0.000	-0.06632	0.06632	0.2581	4	4	0.3633	21
31	0 vs. 5	0.000	0.03464	-0.03464	0.2581	4	4	0.1898	21
32	0 vs. 15	0.000	0.7542	-0.7542	0.2581	4	4	4.131	21
33	0 vs. 30	0.000	0.7446	-0.7446	0.2581	4	4	4.079	21

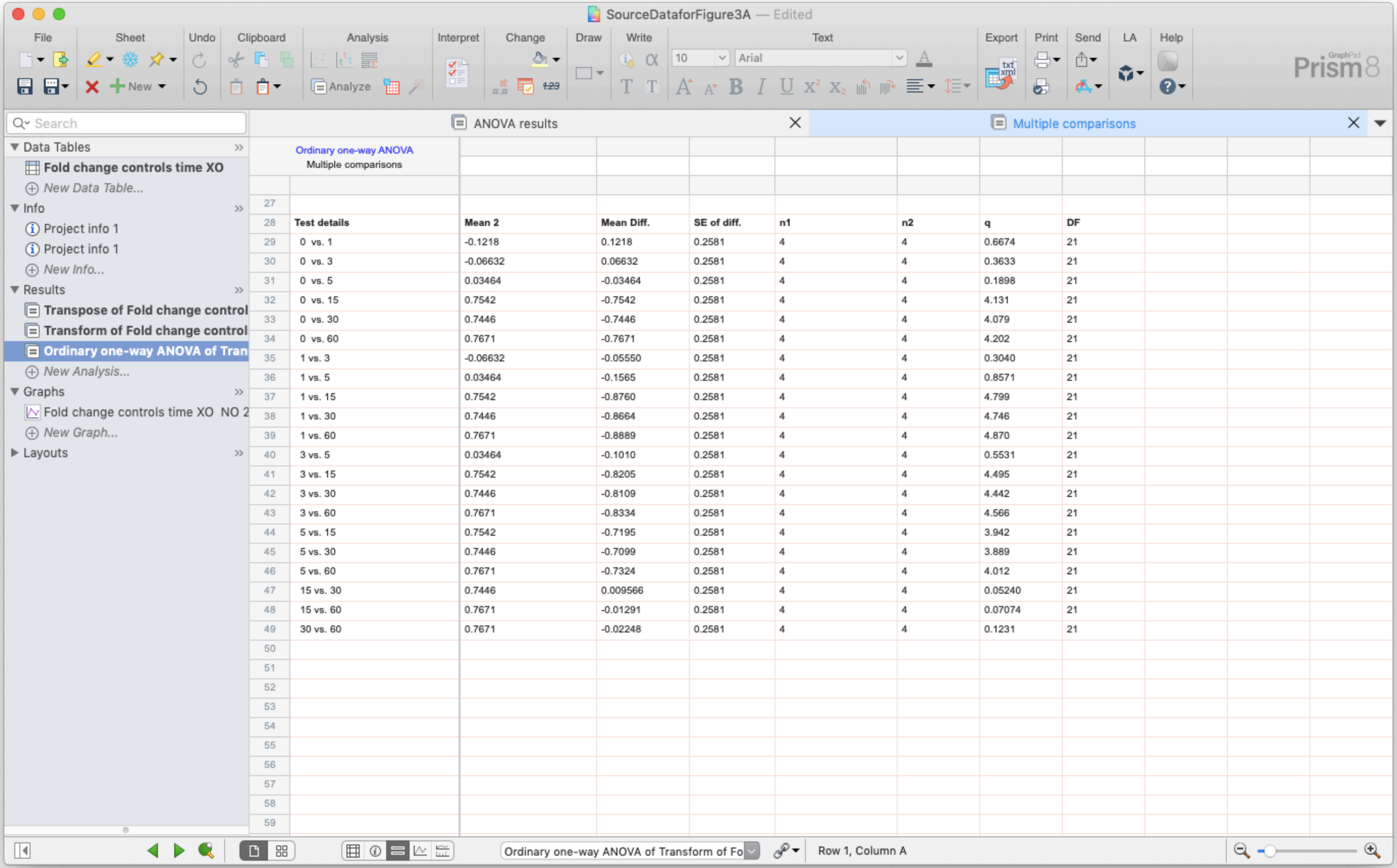

Ordinary one-way ANOVA
Multiple comparisons

Test details	Mean 2	Mean Diff.	SE of diff.	n1	n2	q	DF
0 vs. 1	-0.1218	0.1218	0.2581	4	4	0.6674	21
0 vs. 3	-0.06632	0.06632	0.2581	4	4	0.3633	21
0 vs. 5	0.03464	-0.03464	0.2581	4	4	0.1898	21
0 vs. 15	0.7542	-0.7542	0.2581	4	4	4.131	21
0 vs. 30	0.7446	-0.7446	0.2581	4	4	4.079	21
0 vs. 60	0.7671	-0.7671	0.2581	4	4	4.202	21
1 vs. 3	-0.06632	-0.05550	0.2581	4	4	0.3040	21
1 vs. 5	0.03464	-0.1565	0.2581	4	4	0.8571	21
1 vs. 15	0.7542	-0.8760	0.2581	4	4	4.799	21
1 vs. 30	0.7446	-0.8664	0.2581	4	4	4.746	21
1 vs. 60	0.7671	-0.8889	0.2581	4	4	4.870	21
3 vs. 5	0.03464	-0.1010	0.2581	4	4	0.5531	21
3 vs. 15	0.7542	-0.8205	0.2581	4	4	4.495	21
3 vs. 30	0.7446	-0.8109	0.2581	4	4	4.442	21
3 vs. 60	0.7671	-0.8334	0.2581	4	4	4.566	21
5 vs. 15	0.7542	-0.7195	0.2581	4	4	3.942	21
5 vs. 30	0.7446	-0.7099	0.2581	4	4	3.889	21
5 vs. 60	0.7671	-0.7324	0.2581	4	4	4.012	21
15 vs. 30	0.7446	0.009566	0.2581	4	4	0.05240	21
15 vs. 60	0.7671	-0.01291	0.2581	4	4	0.07074	21
30 vs. 60	0.7671	-0.02248	0.2581	4	4	0.1231	21

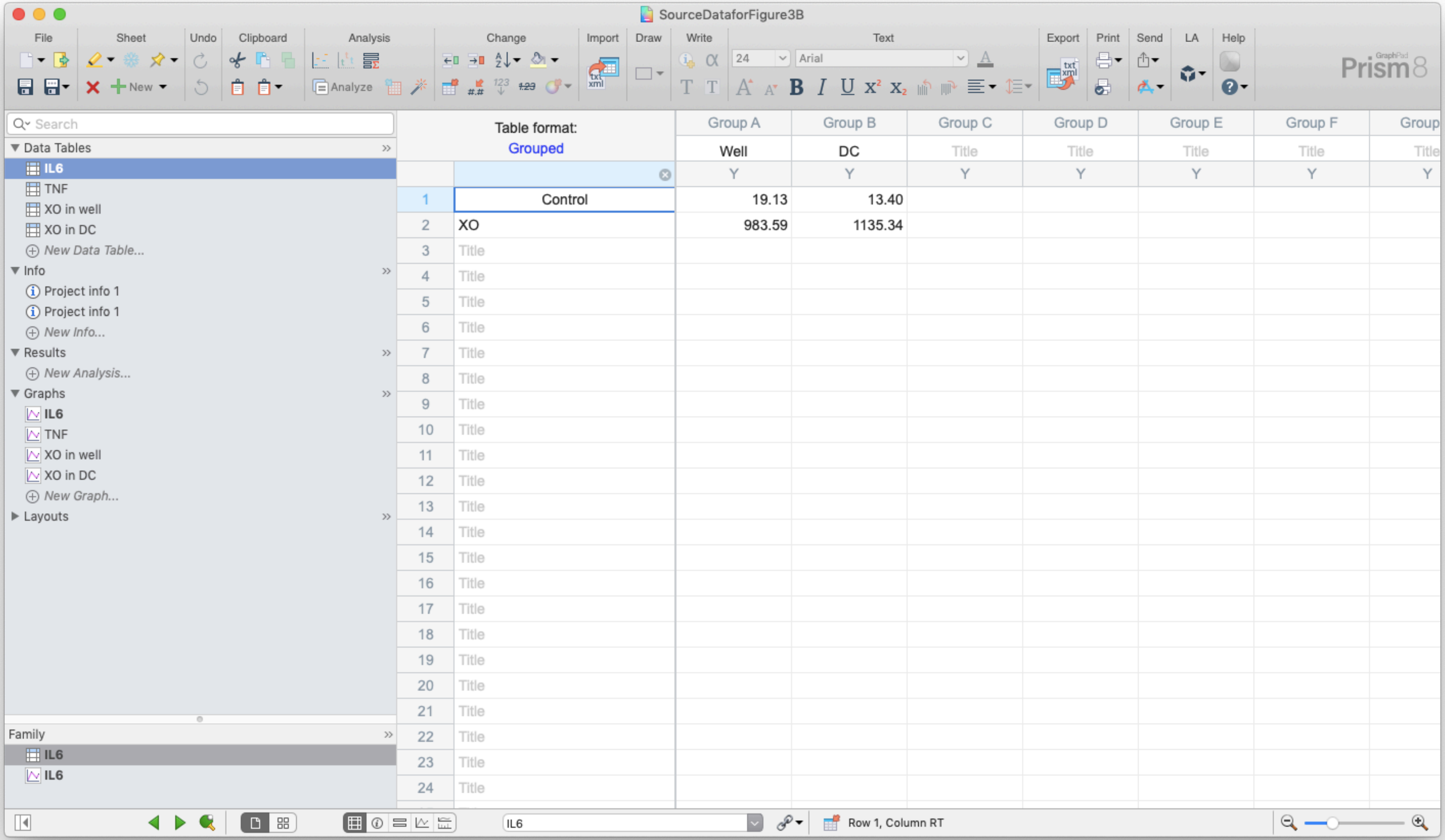

	Group A: Well (Y)	Group B: DC (Y)
1 Control	19.13	13.40
2 XO	983.59	1135.34

SourceDataforFigure3B

Data Tables
- IL6
- TNF
- XO in well
- XO in DC

Info
- Project info 1
- Project info 1

Graphs
- IL6
- TNF
- XO in well
- XO in DC

Table format: Grouped

	Group A	Group B
	Well	DC
	Y	Y
Control	3.72	2.62
XO	241.61	248.27

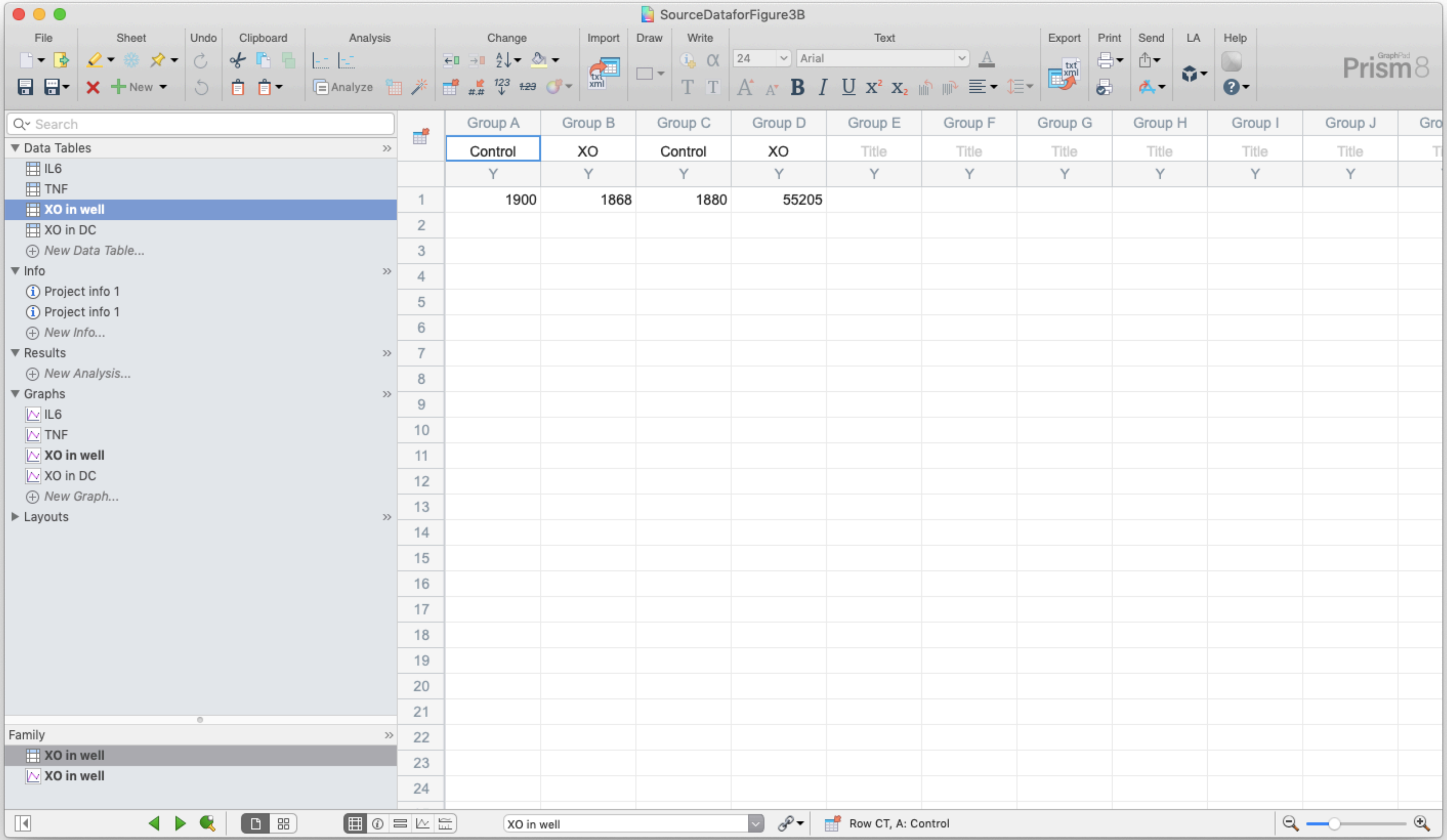

SourceDataforFigure3B

XO in well

	Group A	Group B	Group C	Group D
	Control	XO	Control	XO
	Y	Y	Y	Y
1	1900	1868	1880	55205

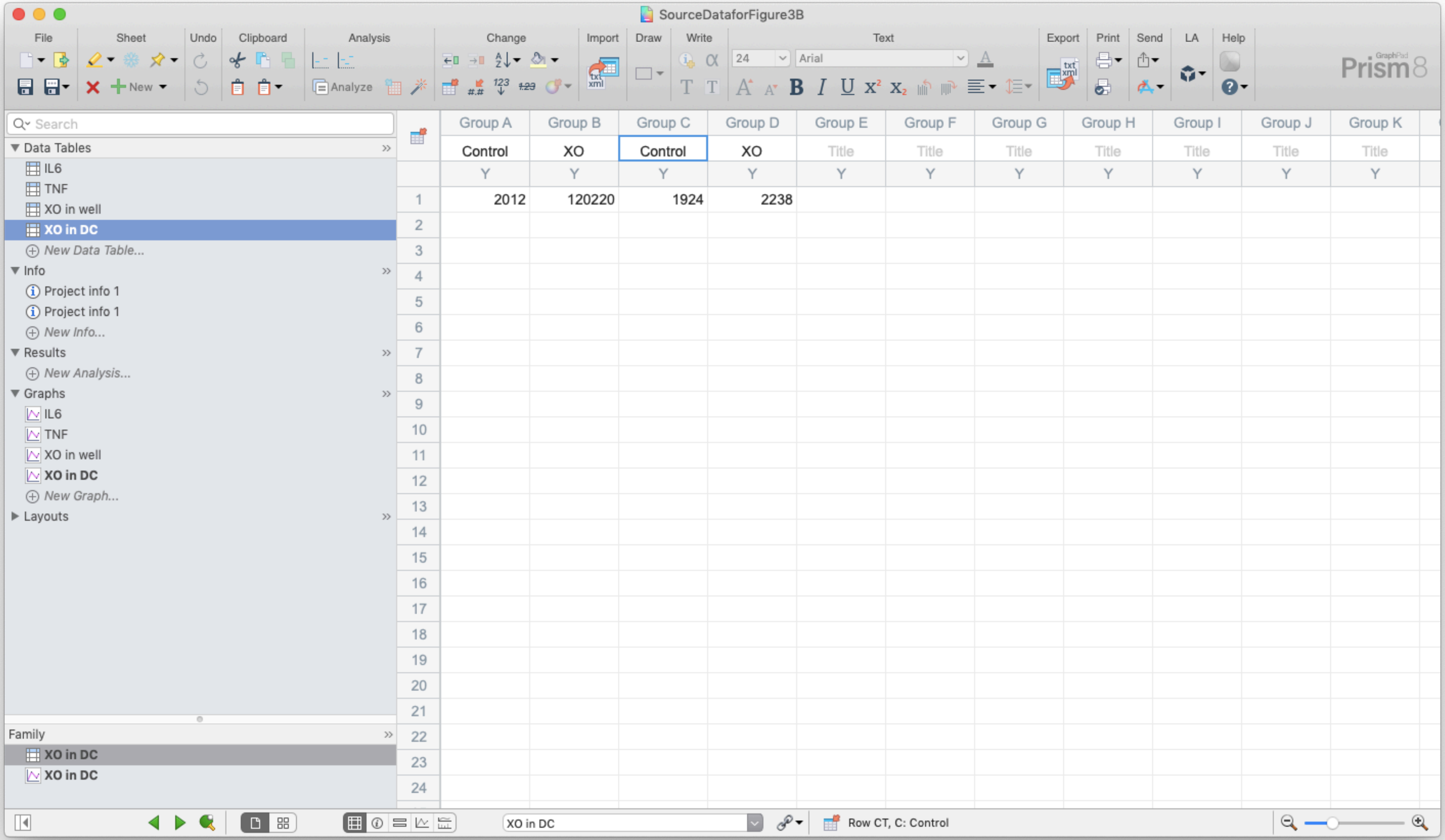
SourceDataforFigure3B
File
Sheet
Undo
Clipboard
Analysis
Change
Import
Draw
Write
Text
Export
Print
Send
LA
Help
Prism8
Search
Data Tables
IL6
TNF
XO in well
XO in DC
New Data Table...
Info
Project info 1
Project info 1
New Info...
Results
New Analysis...
Graphs
IL6
TNF
XO in well
XO in DC
New Graph...
Layouts
Family
XO in DC
XO in DC
Group A
Group B
Group C
Group D
Group E
Group F
Group G
Group H
Group I
Group J
Group K
Control
XO
Control
XO
Title
Y
2012
120220
1924
2238
Row CT, C: Control
