# Supplementary material for: Malaria inflammation by xanthine oxidase‐produced reactive oxygen species
Source: EMBO Mol Med. 2019 Jul 2;11(8):e9903. doi: 10.15252/emmm.201809903 (PMC6685105; doi:10.15252/emmm.201809903)
Supplement: Supplementary file 7 — Source Data for Figure 4 [file EMMM-11-e9903-s005.pdf]

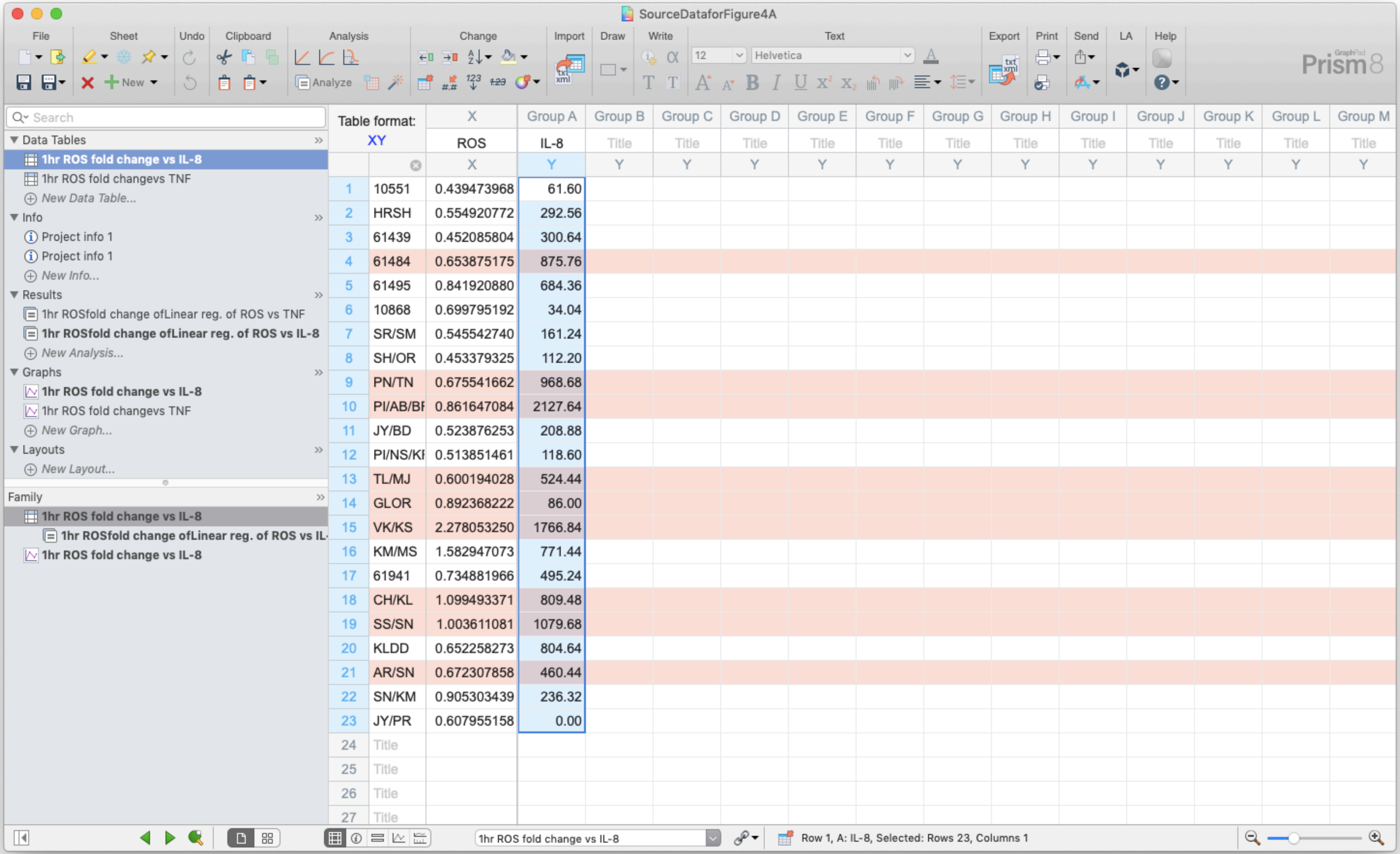

Table format: XY		X	Group A
		ROS	IL-8
		X	Y
1	10551	0.439473968	61.60
2	HRSH	0.554920772	292.56
3	61439	0.452085804	300.64
4	61484	0.653875175	875.76
5	61495	0.841920880	684.36
6	10868	0.699795192	34.04
7	SR/SM	0.545542740	161.24
8	SH/OR	0.453379325	112.20
9	PN/TN	0.675541662	968.68
10	PI/AB/B[illegible]	0.861647084	2127.64
11	JY/BD	0.523876253	208.88
12	PI/NS/K[illegible]	0.513851461	118.60
13	TL/MJ	0.600194028	524.44
14	GLOR	0.892368222	86.00
15	VK/KS	2.278053250	1766.84
16	KM/MS	1.582947073	771.44
17	61941	0.734881966	495.24
18	CH/KL	1.099493371	809.48
19	SS/SN	1.003611081	1079.68
20	KLDD	0.652258273	804.64
21	AR/SN	0.672307858	460.44
22	SN/KM	0.905303439	236.32
23	JY/PR	0.607955158	0.00

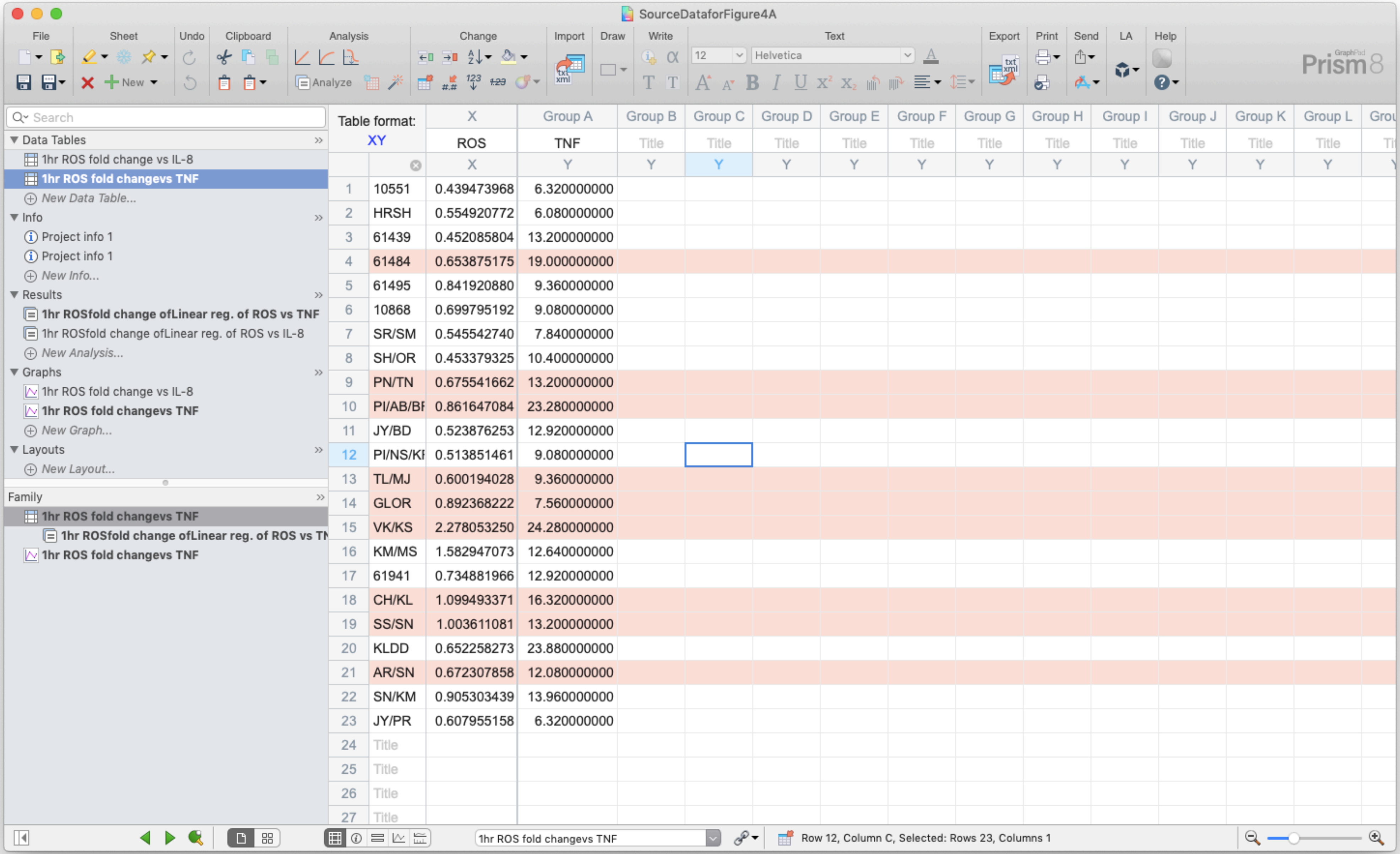

Table format: XY

		X	Group A
		ROS	TNF
		X	Y
1	10551	0.439473968	6.320000000
2	HRSH	0.554920772	6.080000000
3	61439	0.452085804	13.200000000
4	61484	0.653875175	19.000000000
5	61495	0.841920880	9.360000000
6	10868	0.699795192	9.080000000
7	SR/SM	0.545542740	7.840000000
8	SH/OR	0.453379325	10.400000000
9	PN/TN	0.675541662	13.200000000
10	PI/AB/BF	0.861647084	23.280000000
11	JY/BD	0.523876253	12.920000000
12	PI/NS/KI	0.513851461	9.080000000
13	TL/MJ	0.600194028	9.360000000
14	GLOR	0.892368222	7.560000000
15	VK/KS	2.278053250	24.280000000
16	KM/MS	1.582947073	12.640000000
17	61941	0.734881966	12.920000000
18	CH/KL	1.099493371	16.320000000
19	SS/SN	1.003611081	13.200000000
20	KLDD	0.652258273	23.880000000
21	AR/SN	0.672307858	12.080000000
22	SN/KM	0.905303439	13.960000000
23	JY/PR	0.607955158	6.320000000
24	Title		
25	Title		
26	Title		
27	Title		

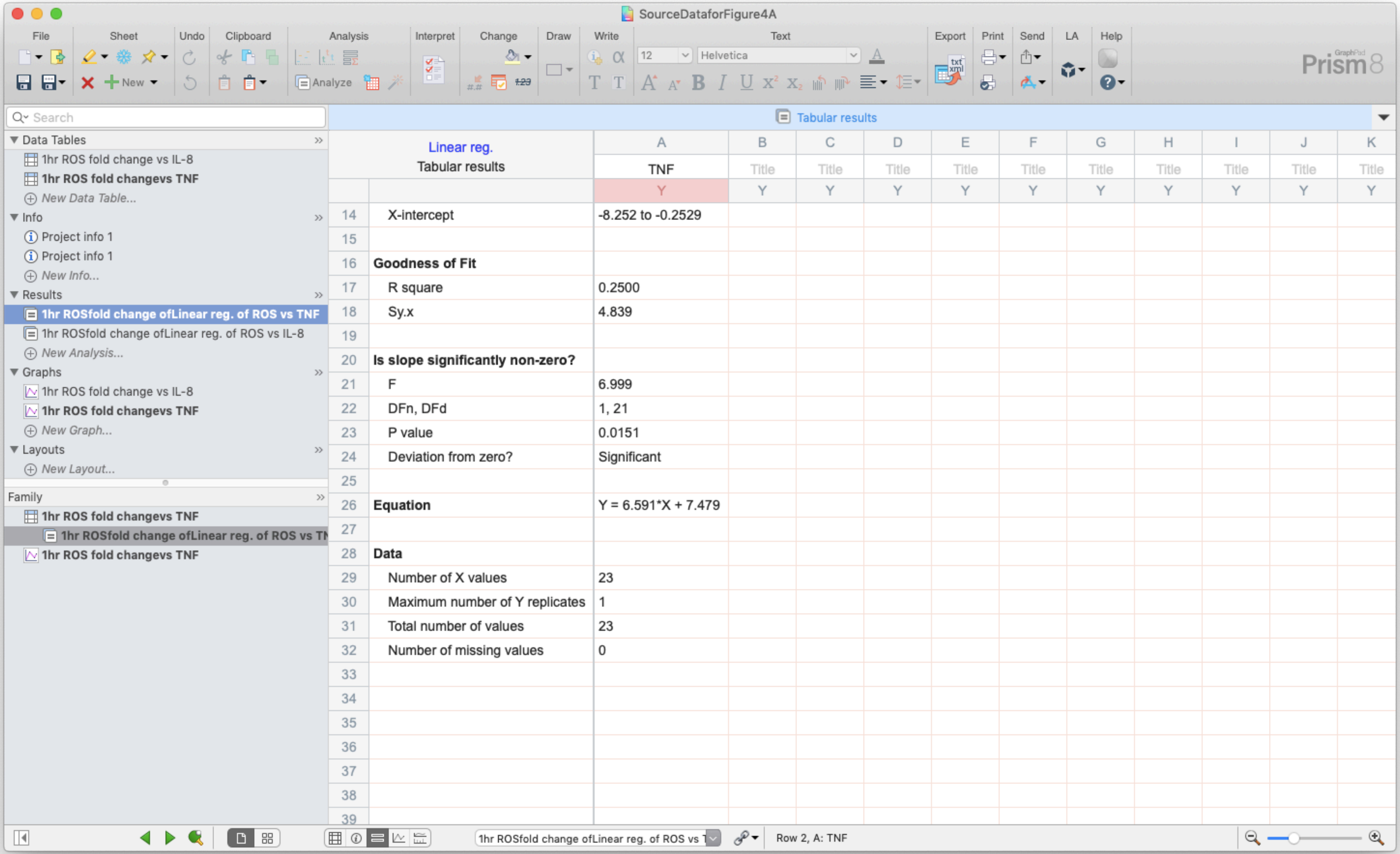
SourceDataforFigure4A
File
Sheet
Undo
Clipboard
Analysis
Interpret
Change
Draw
Write
Text
Export
Print
Send
LA
Help
New
Analyze
Helvetica
12
Prism 8
Search
Tabular results
Data Tables
1hr ROS fold change vs IL-8
1hr ROS fold changevs TNF
New Data Table...
Info
Project info 1
Project info 1
New Info...
Results
1hr ROSfold change ofLinear reg. of ROS vs TNF
1hr ROSfold change ofLinear reg. of ROS vs IL-8
New Analysis...
Graphs
1hr ROS fold change vs IL-8
1hr ROS fold changevs TNF
New Graph...
Layouts
New Layout...
Family
1hr ROS fold changevs TNF
1hr ROSfold change ofLinear reg. of ROS vs TN
1hr ROS fold changevs TNF
Linear reg.
Tabular results
A
TNF
Y
14 X-intercept -8.252 to -0.2529
15
16 Goodness of Fit
17 R square 0.2500
18 Sy.x 4.839
19
20 Is slope significantly non-zero?
21 F 6.999
22 DFn, DFd 1, 21
23 P value 0.0151
24 Deviation from zero? Significant
25
26 Equation Y = 6.591*X + 7.479
27
28 Data
29 Number of X values 23
30 Maximum number of Y replicates 1
31 Total number of values 23
32 Number of missing values 0
1hr ROSfold change ofLinear reg. of ROS vs T
Row 2, A: TNF

SourceDataforFigure4A

Tabular results

- Data Tables
  - 1hr ROS fold change vs IL-8
  - 1hr ROS fold changevs TNF
  - New Data Table...
- Info
  - Project info 1
  - Project info 1
  - New Info...
- Results
  - 1hr ROSfold change ofLinear reg. of ROS vs TNF
  - 1hr ROSfold change ofLinear reg. of ROS vs IL-8
  - New Analysis...
- Graphs
  - 1hr ROS fold change vs IL-8
  - 1hr ROS fold changevs TNF
  - New Graph...
- Layouts
  - New Layout...

Family
- 1hr ROS fold change vs IL-8
  - 1hr ROSfold change ofLinear reg. of ROS vs IL-
- 1hr ROS fold change vs IL-8

	Linear reg. Tabular results	A IL-8 Y
1	**Best-fit values**	
2	Slope	800.7
3	Y-intercept	-70.78
4	X-intercept	0.08840
5	1/slope	0.001249
6		
7	**Std. Error**	
8	Slope	230.6
9	Y-intercept	205.4
10		
11	**95% Confidence Intervals**	
12	Slope	321.1 to 1280
13	Y-intercept	-497.9 to 356.4
14	X-intercept	-1.031 to 0.4188
15		
16	**Goodness of Fit**	
17	R square	0.3647
18	Sy.x	447.9
19		
20	**Is slope significantly non-zero?**	
21	F	12.06
22	DFn, DFd	1, 21
23	P value	0.0023
24	Deviation from zero?	Significant
25		
26	**Equation**	Y = 800.7*X - 70.78
27		

Row 2, A: IL-8

SourceDataforFigure4B

File | Sheet | Undo | Clipboard | Analysis | Change | Import | Draw | Write | Text | Export | Print | Send | LA | Help

Prism 8

Data Tables
- ROS CM & UM (fold change)
- *New Data Table...*

Info
- Project info 1
- Project info 1
- *New Info...*

Results
- Mann-Whitney test of ROS CM & UM (fold change)
- Normality and Lognormality Tests of ROS CM & UM (fold change)
- *New Analysis...*

Graphs
- ROS CM & UM (fold change)
- Normal QQ plot: Normality and Lognormality Tests of ROS CM & UM (fold
- *New Graph...*

Layouts
- *New Layout...*

Family
- ROS CM & UM (fold change)
  - Mann-Whitney test
  - Normality and Lognormality Tests
- ROS CM & UM (fold change)
- Normal QQ plot: Normality and Lognormality Tests of ROS CM & UM (fold

	Group A	Group B	Group C	Group D	Group E	Group F	Group G	Group H	Group I	Group J	Group K
	UM	CM	Title	Title	Title	Title	Title	Title	Title	Title	Title
	Y	Y	Y	Y	Y	Y	Y	Y	Y	Y	Y
1	0.439473968	0.653875175									
2	0.554920772	0.675541662									
3	0.452085804	0.861647084									
4	0.841920880	0.600194028									
5	0.699795192	0.892368222									
6	0.545542740	2.278053250									
7	0.453379325	1.099493371									
8	0.523876253	1.003611081									
9	0.513851461	0.672307858									
10	1.582947073										
11	0.734881966										
12	0.652258273										
13	0.905303439										
14	0.607955158										
15											
16											
17											
18											
19											
20											
21											
22											
23											
24											
25											
26											

ROS CM & UM (fold change) | Row 10, A: UM

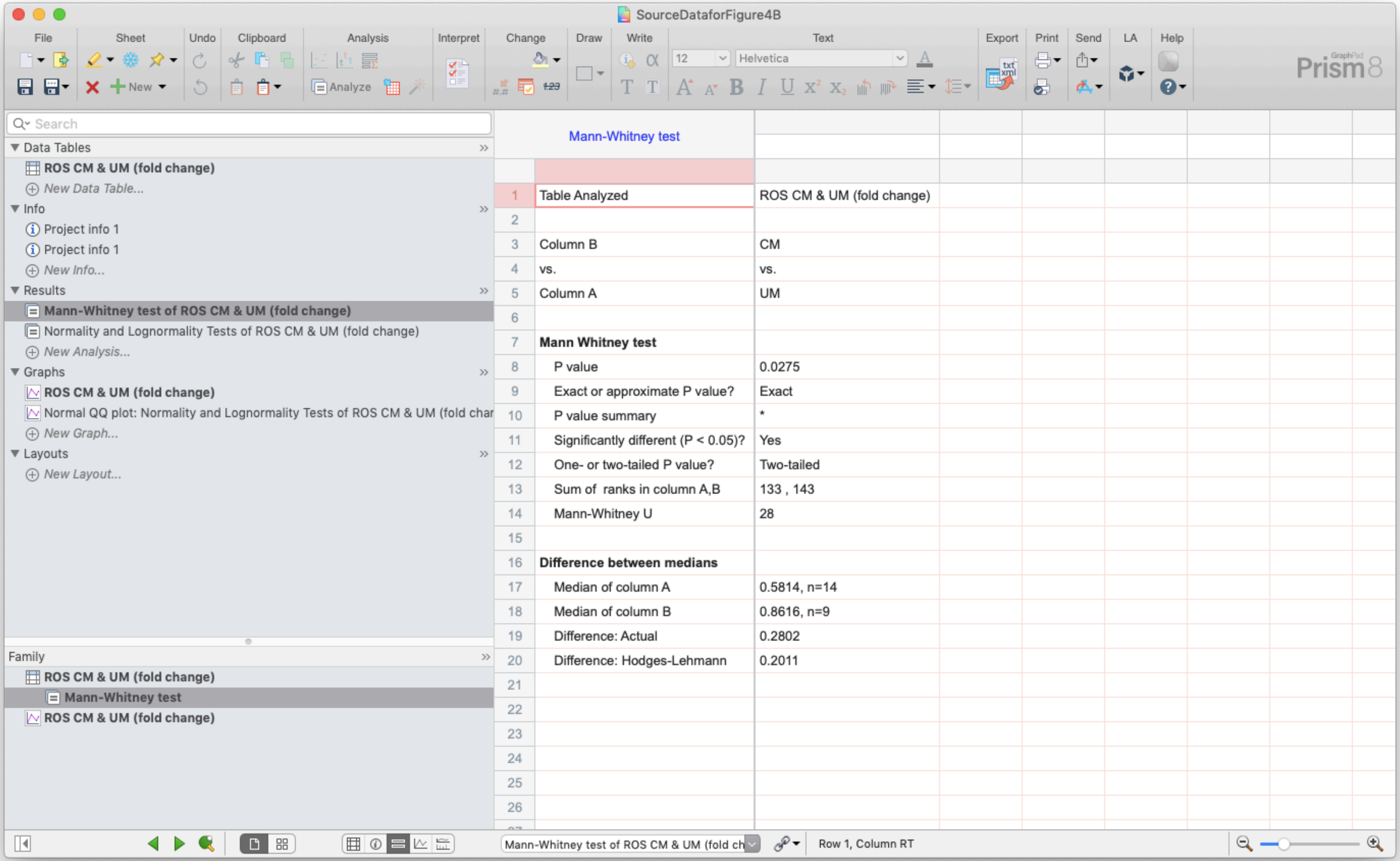

	Mann-Whitney test	
1	Table Analyzed	ROS CM & UM (fold change)
2		
3	Column B	CM
4	vs.	vs.
5	Column A	UM
6		
7	**Mann Whitney test**	
8	P value	0.0275
9	Exact or approximate P value?	Exact
10	P value summary	*
11	Significantly different (P < 0.05)?	Yes
12	One- or two-tailed P value?	Two-tailed
13	Sum of ranks in column A,B	133 , 143
14	Mann-Whitney U	28
15		
16	**Difference between medians**	
17	Median of column A	0.5814, n=14
18	Median of column B	0.8616, n=9
19	Difference: Actual	0.2802
20	Difference: Hodges-Lehmann	0.2011

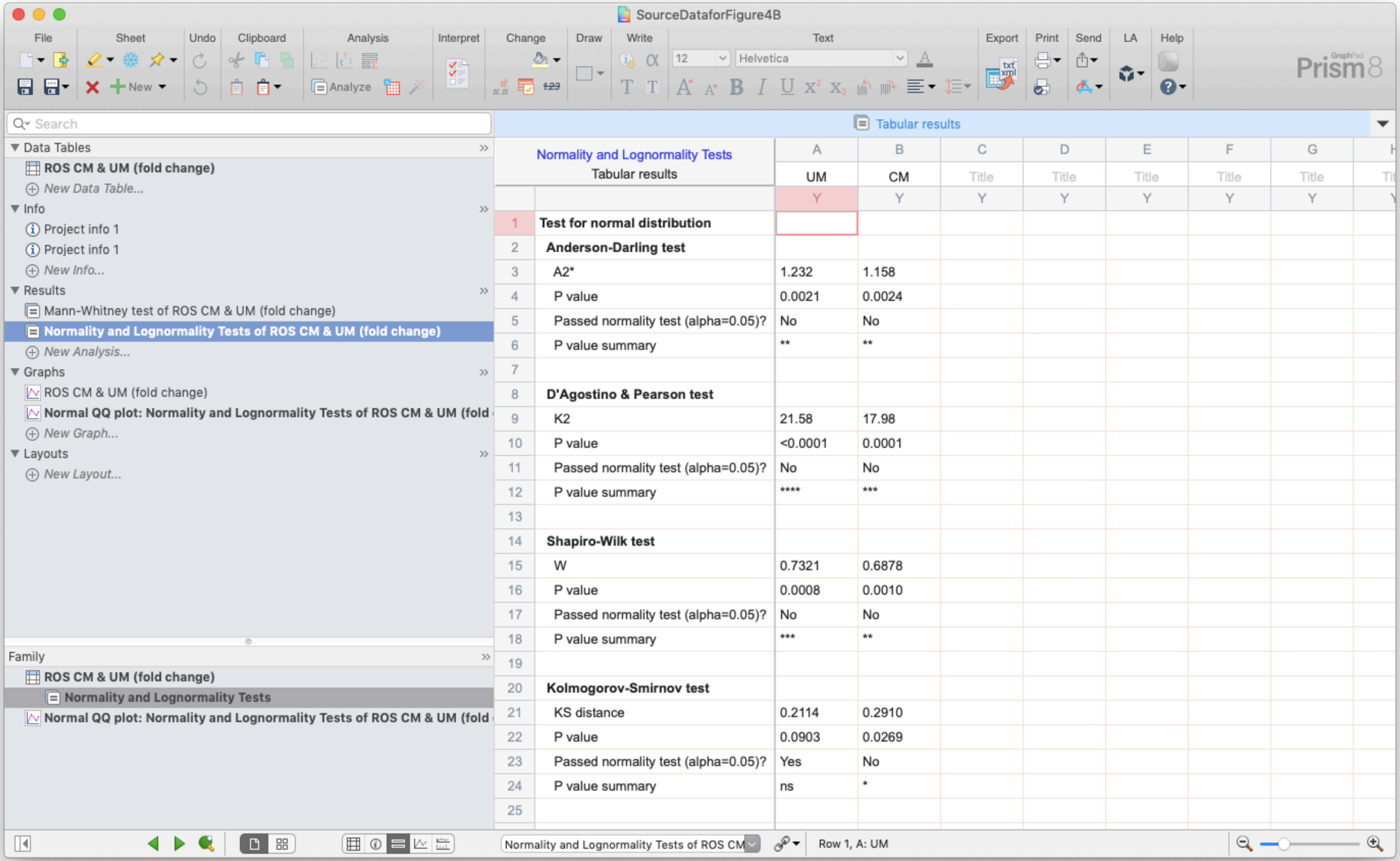

Normality and Lognormality Tests Tabular results		A UM Y	B CM Y
1	Test for normal distribution		
2	Anderson-Darling test		
3	A2*	1.232	1.158
4	P value	0.0021	0.0024
5	Passed normality test (alpha=0.05)?	No	No
6	P value summary	**	**
7			
8	D'Agostino & Pearson test		
9	K2	21.58	17.98
10	P value	<0.0001	0.0001
11	Passed normality test (alpha=0.05)?	No	No
12	P value summary	****	***
13			
14	Shapiro-Wilk test		
15	W	0.7321	0.6878
16	P value	0.0008	0.0010
17	Passed normality test (alpha=0.05)?	No	No
18	P value summary	***	**
19			
20	Kolmogorov-Smirnov test		
21	KS distance	0.2114	0.2910
22	P value	0.0903	0.0269
23	Passed normality test (alpha=0.05)?	Yes	No
24	P value summary	ns	*
25			

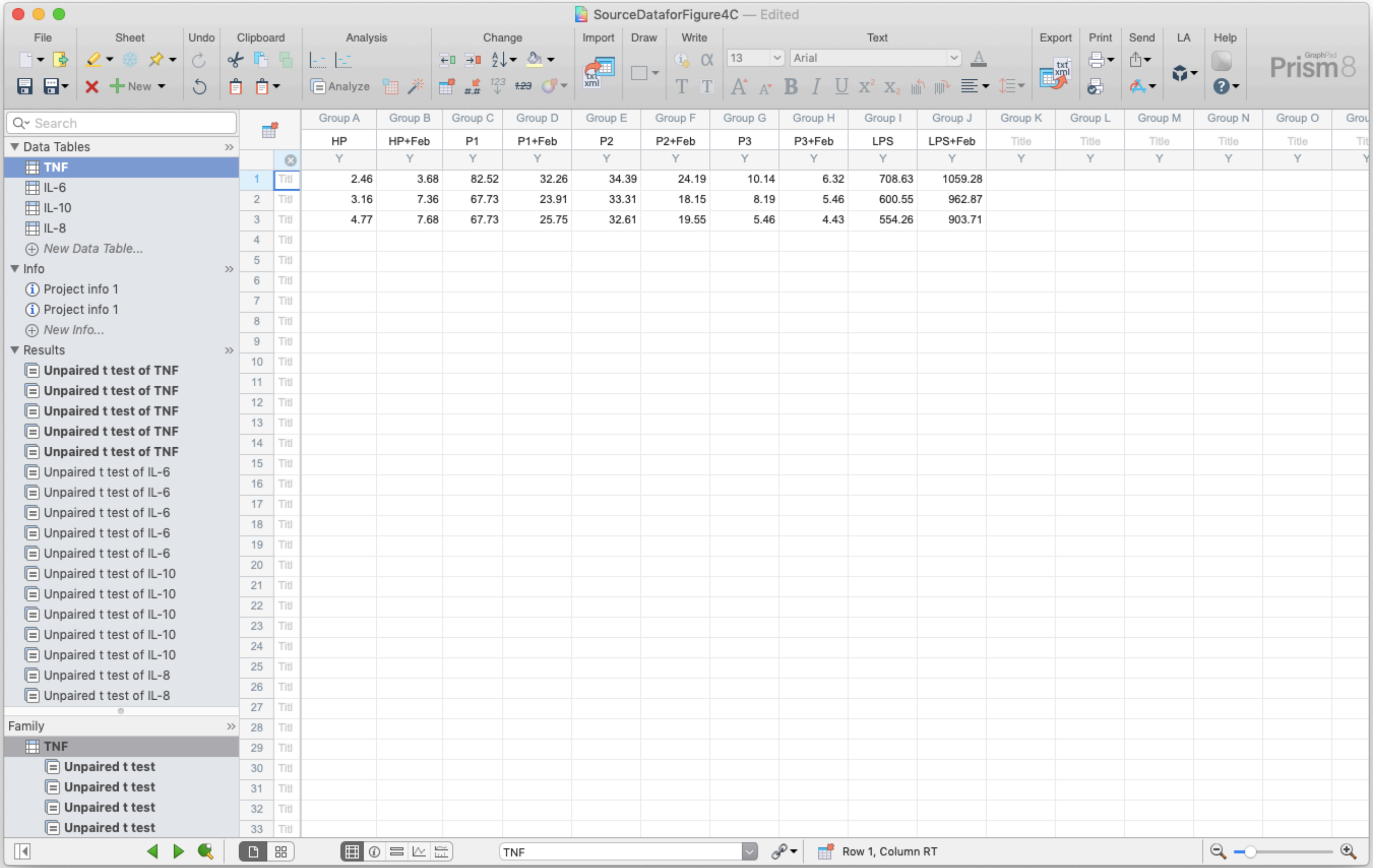

	Group A	Group B	Group C	Group D	Group E	Group F	Group G	Group H	Group I	Group J
	HP	HP+Feb	P1	P1+Feb	P2	P2+Feb	P3	P3+Feb	LPS	LPS+Feb
	Y	Y	Y	Y	Y	Y	Y	Y	Y	Y
1	2.46	3.68	82.52	32.26	34.39	24.19	10.14	6.32	708.63	1059.28
2	3.16	7.36	67.73	23.91	33.31	18.15	8.19	5.46	600.55	962.87
3	4.77	7.68	67.73	25.75	32.61	19.55	5.46	4.43	554.26	903.71

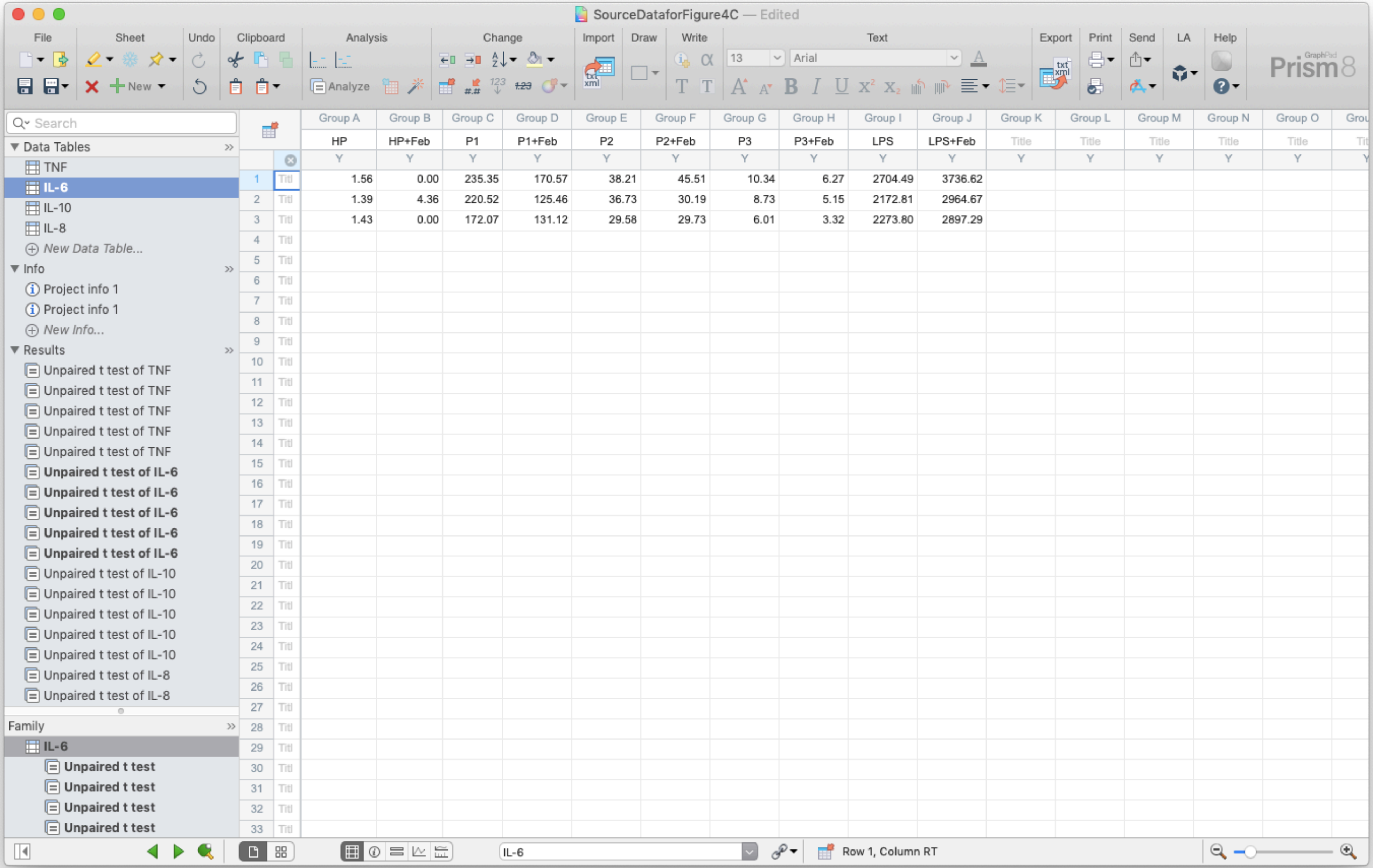

	Group A	Group B	Group C	Group D	Group E	Group F	Group G	Group H	Group I	Group J
	HP	HP+Feb	P1	P1+Feb	P2	P2+Feb	P3	P3+Feb	LPS	LPS+Feb
	Y	Y	Y	Y	Y	Y	Y	Y	Y	Y
1	1.56	0.00	235.35	170.57	38.21	45.51	10.34	6.27	2704.49	3736.62
2	1.39	4.36	220.52	125.46	36.73	30.19	8.73	5.15	2172.81	2964.67
3	1.43	0.00	172.07	131.12	29.58	29.73	6.01	3.32	2273.80	2897.29

SourceDataforFigure4C — Edited

IL-10

	HP	HP+Feb	P1	P1+Feb	P2	P2+Feb	P3	P3+Feb	LPS	LPS+Feb
	Group A	Group B	Group C	Group D	Group E	Group F	Group G	Group H	Group I	Group J
1	0.00	0.00	4.83	3.65	2.43	1.74	1.27	0.00	20.85	32.85
2	0.00	0.00	4.95	2.06	1.47	1.71	0.00	1.53	16.01	25.50
3	1.27	0.00	3.34	1.65	1.77	0.00	0.00	0.00	15.31	23.14

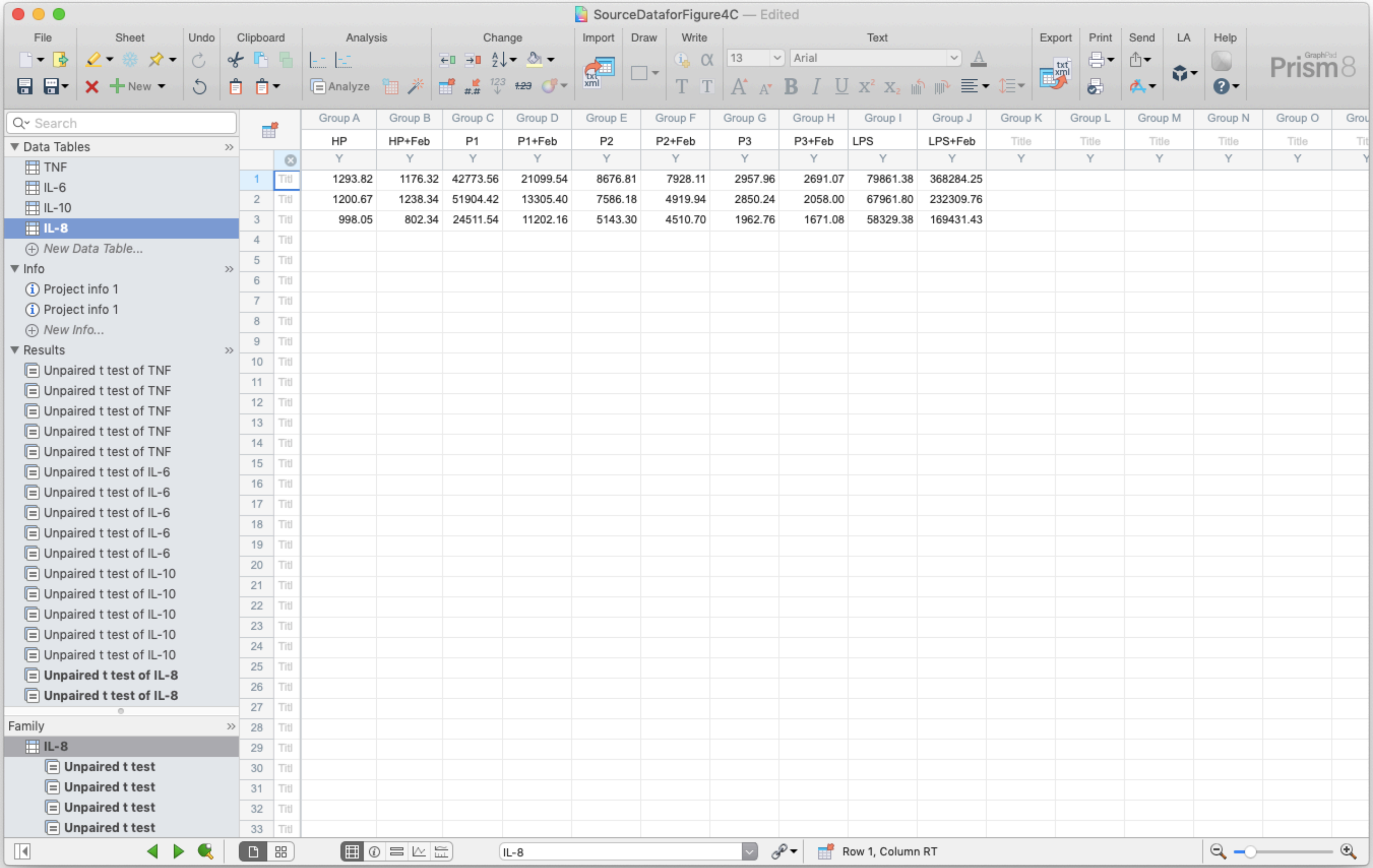

	Group A	Group B	Group C	Group D	Group E	Group F	Group G	Group H	Group I	Group J
	HP	HP+Feb	P1	P1+Feb	P2	P2+Feb	P3	P3+Feb	LPS	LPS+Feb
	Y	Y	Y	Y	Y	Y	Y	Y	Y	Y
1	1293.82	1176.32	42773.56	21099.54	8676.81	7928.11	2957.96	2691.07	79861.38	368284.25
2	1200.67	1238.34	51904.42	13305.40	7586.18	4919.94	2850.24	2058.00	67961.80	232309.76
3	998.05	802.34	24511.54	11202.16	5143.30	4510.70	1962.76	1671.08	58329.38	169431.43

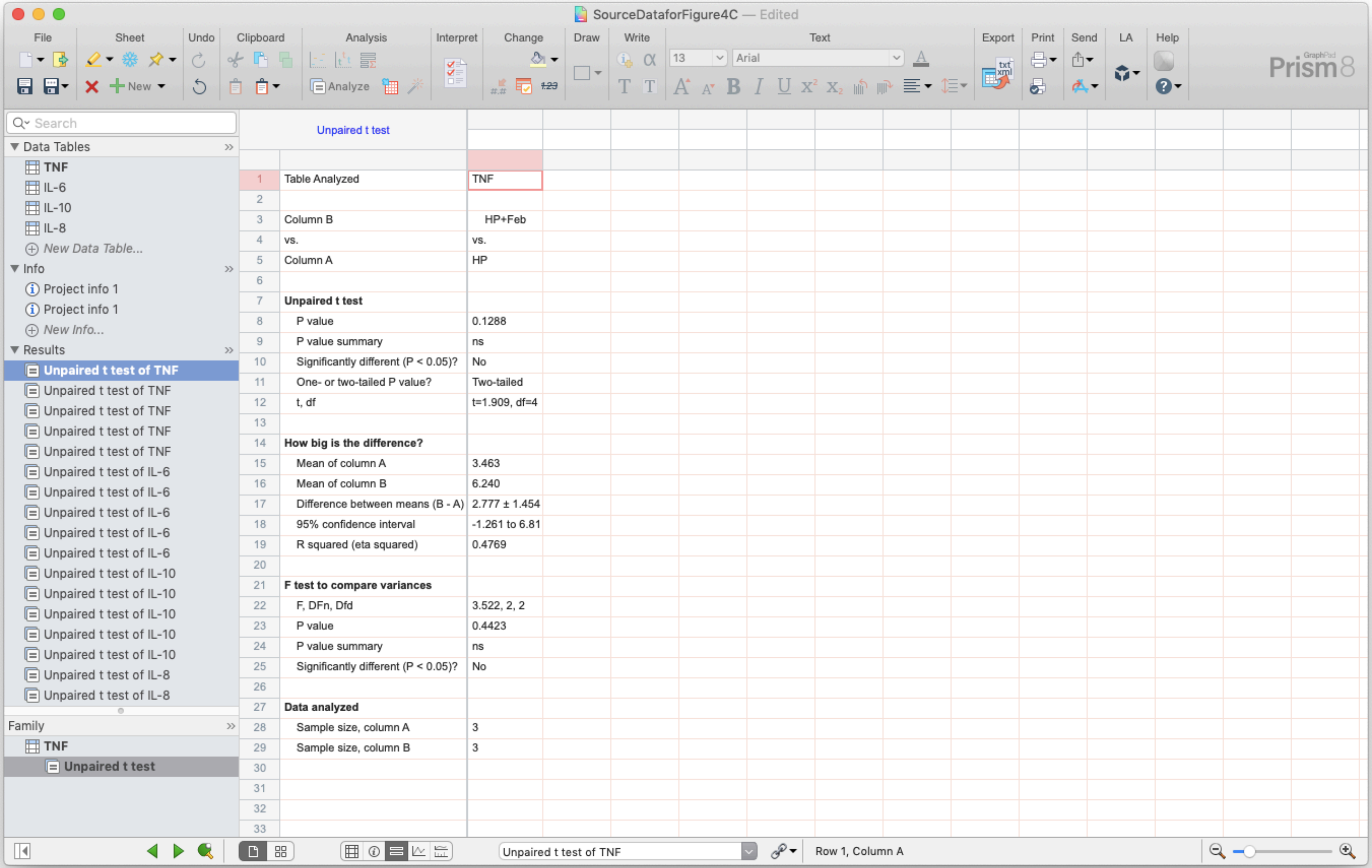

	Unpaired t test	
1	Table Analyzed	TNF
2		
3	Column B	HP+Feb
4	vs.	vs.
5	Column A	HP
6		
7	**Unpaired t test**	
8	P value	0.1288
9	P value summary	ns
10	Significantly different (P < 0.05)?	No
11	One- or two-tailed P value?	Two-tailed
12	t, df	t=1.909, df=4
13		
14	**How big is the difference?**	
15	Mean of column A	3.463
16	Mean of column B	6.240
17	Difference between means (B - A)	2.777 ± 1.454
18	95% confidence interval	-1.261 to 6.81
19	R squared (eta squared)	0.4769
20		
21	**F test to compare variances**	
22	F, DFn, Dfd	3.522, 2, 2
23	P value	0.4423
24	P value summary	ns
25	Significantly different (P < 0.05)?	No
26		
27	**Data analyzed**	
28	Sample size, column A	3
29	Sample size, column B	3

SourceDataforFigure4C — Edited

Unpaired t test

1	Table Analyzed	TNF
2		
3	Column D	P1+Feb
4	vs.	vs.
5	Column C	P1
6		
7	**Unpaired t test**	
8	P value	0.0012
9	P value summary	**
10	Significantly different (P < 0.05)?	Yes
11	One- or two-tailed P value?	Two-tailed
12	t, df	t=8.183, df=4
13		
14	**How big is the difference?**	
15	Mean of column C	72.66
16	Mean of column D	27.31
17	Difference between means (D - C)	-45.35 ± 5.54[illegible]
18	95% confidence interval	-60.74 to -29.[illegible]
19	R squared (eta squared)	0.9436
20		
21	**F test to compare variances**	
22	F, DFn, Dfd	3.788, 2, 2
23	P value	0.4177
24	P value summary	ns
25	Significantly different (P < 0.05)?	No
26		
27	**Data analyzed**	
28	Sample size, column C	3
29	Sample size, column D	3

Row 9, Column A

SourceDataforFigure4C — Edited

Unpaired t test of TNF

	Unpaired t test	
1	Table Analyzed	TNF
2		
3	Column H	P3+Feb
4	vs.	vs.
5	Column G	P3
6		
7	**Unpaired t test**	
8	P value	0.1592
9	P value summary	ns
10	Significantly different (P < 0.05)?	No
11	One- or two-tailed P value?	Two-tailed
12	t, df	t=1.727, df=4
13		
14	**How big is the difference?**	
15	Mean of column G	7.930
16	Mean of column H	5.403
17	Difference between means (H - G)	-2.527 ± 1.46[illegible]
18	95% confidence interval	-6.589 to 1.53
19	R squared (eta squared)	0.4271
20		
21	**F test to compare variances**	
22	F, DFn, Dfd	6.172, 2, 2
23	P value	0.2789
24	P value summary	ns
25	Significantly different (P < 0.05)?	No
26		
27	**Data analyzed**	
28	Sample size, column G	3
29	Sample size, column H	3

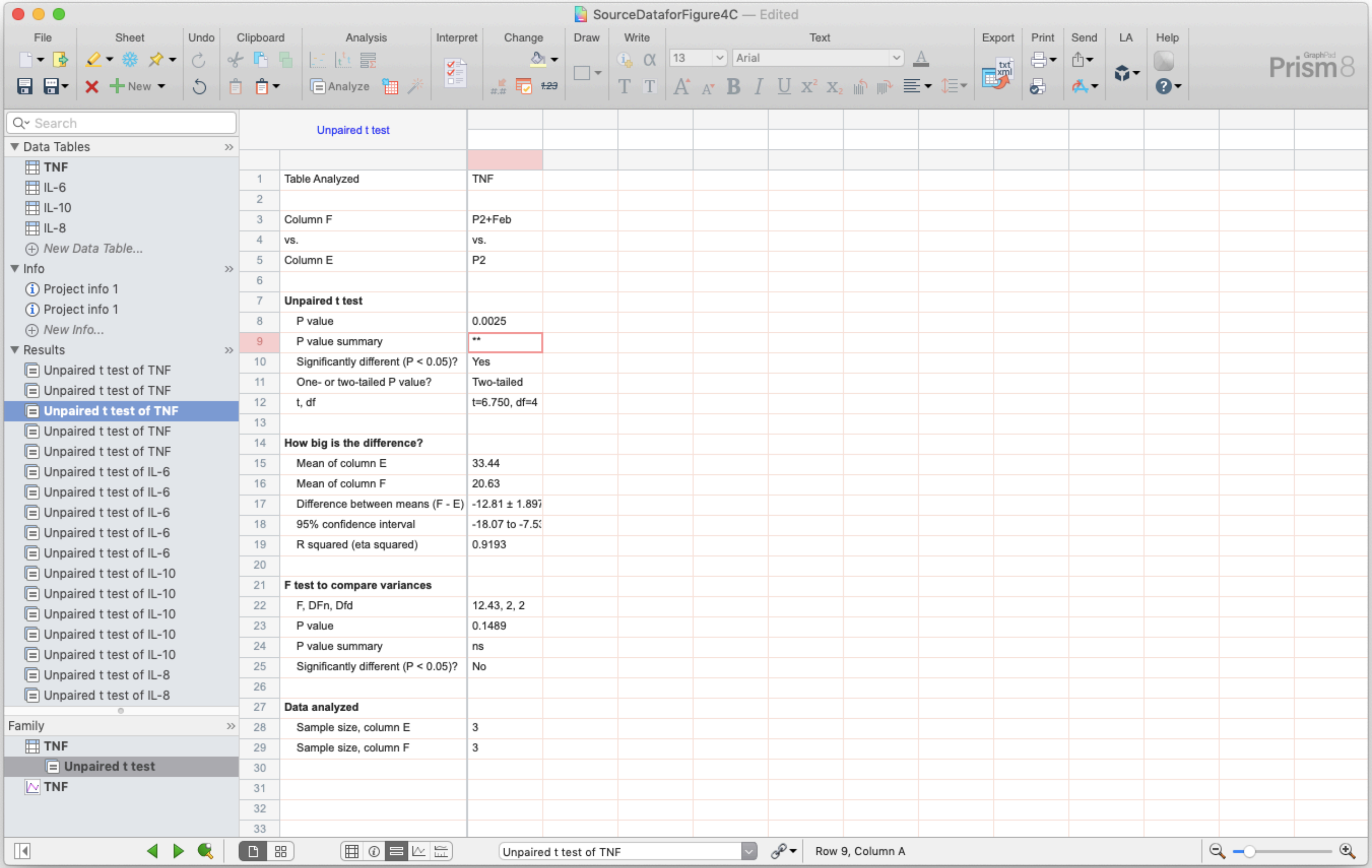

SourceDataforFigure4C — Edited

	Unpaired t test	
1	Table Analyzed	TNF
2		
3	Column F	P2+Feb
4	vs.	vs.
5	Column E	P2
6		
7	**Unpaired t test**	
8	P value	0.0025
9	P value summary	**
10	Significantly different (P < 0.05)?	Yes
11	One- or two-tailed P value?	Two-tailed
12	t, df	t=6.750, df=4
13		
14	**How big is the difference?**	
15	Mean of column E	33.44
16	Mean of column F	20.63
17	Difference between means (F - E)	-12.81 ± 1.89[illegible]
18	95% confidence interval	-18.07 to -7.5[illegible]
19	R squared (eta squared)	0.9193
20		
21	**F test to compare variances**	
22	F, DFn, Dfd	12.43, 2, 2
23	P value	0.1489
24	P value summary	ns
25	Significantly different (P < 0.05)?	No
26		
27	**Data analyzed**	
28	Sample size, column E	3
29	Sample size, column F	3

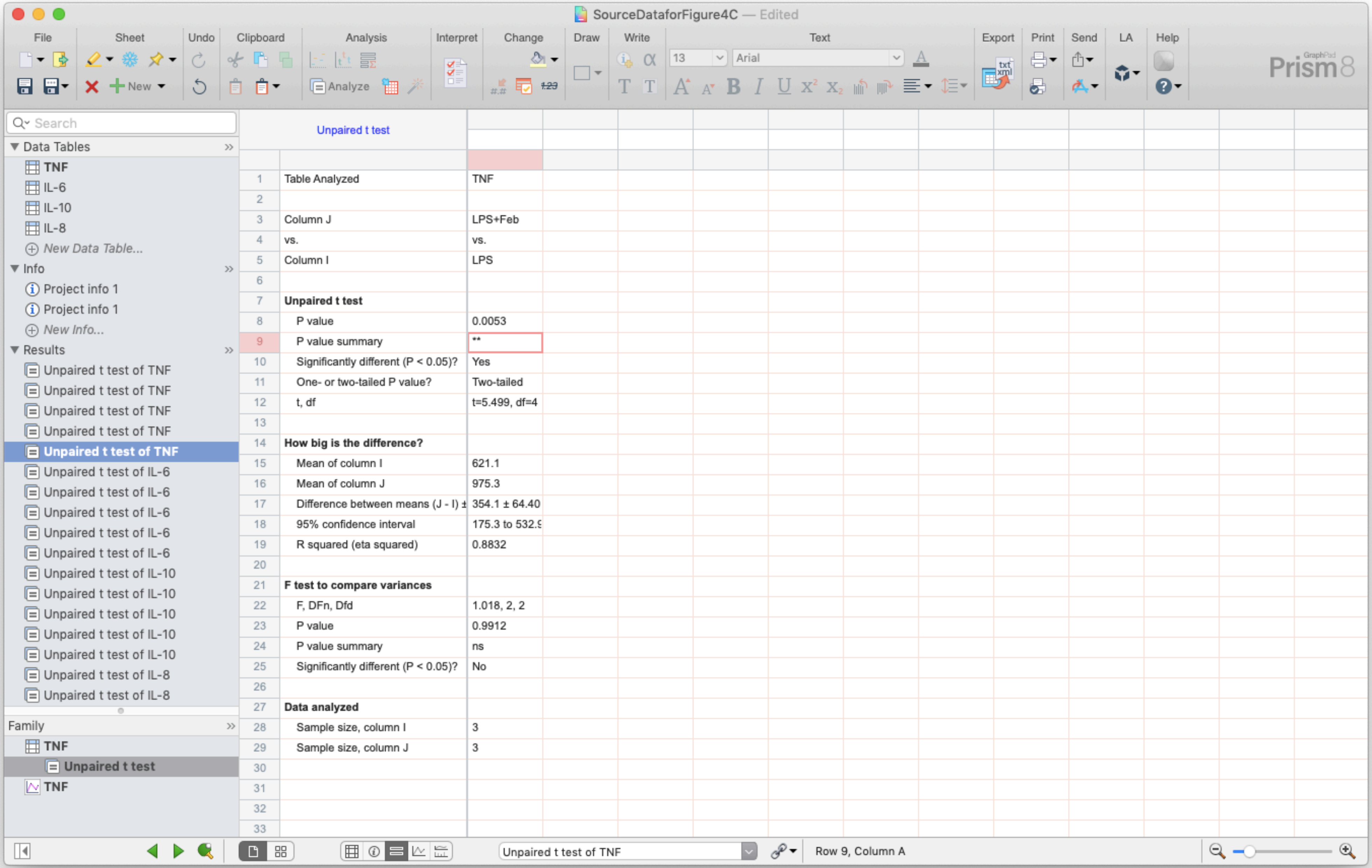

	Unpaired t test	
1	Table Analyzed	TNF
2		
3	Column J	LPS+Feb
4	vs.	vs.
5	Column I	LPS
6		
7	**Unpaired t test**	
8	P value	0.0053
9	P value summary	**
10	Significantly different (P < 0.05)?	Yes
11	One- or two-tailed P value?	Two-tailed
12	t, df	t=5.499, df=4
13		
14	**How big is the difference?**	
15	Mean of column I	621.1
16	Mean of column J	975.3
17	Difference between means (J - I) ±	354.1 ± 64.40
18	95% confidence interval	175.3 to 532.9
19	R squared (eta squared)	0.8832
20		
21	**F test to compare variances**	
22	F, DFn, Dfd	1.018, 2, 2
23	P value	0.9912
24	P value summary	ns
25	Significantly different (P < 0.05)?	No
26		
27	**Data analyzed**	
28	Sample size, column I	3
29	Sample size, column J	3

SourceDataforFigure4C — Edited

Unpaired t test

1	Table Analyzed	IL-6
2		
3	Column B	HP+Feb
4	vs.	vs.
5	Column A	HP
6		
7	**Unpaired t test**	
8	P value	0.9966
9	P value summary	ns
10	Significantly different (P < 0.05)?	No
11	One- or two-tailed P value?	Two-tailed
12	t, df	t=0.004584, d[illegible]
13		
14	**How big is the difference?**	
15	Mean of column A	1.460
16	Mean of column B	1.453
17	Difference between means (B - A)	-0.006667 ± 1[illegible]
18	95% confidence interval	-4.044 to 4.03[illegible]
19	R squared (eta squared)	5.254e-006
20		
21	**F test to compare variances**	
22	F, DFn, Dfd	802.1, 2, 2
23	P value	0.0025
24	P value summary	**
25	Significantly different (P < 0.05)?	Yes
26		
27	**Data analyzed**	
28	Sample size, column A	3
29	Sample size, column B	3

SourceDataforFigure4C — Edited

Data Tables: TNF, IL-6, IL-10, IL-8, New Data Table...

Info: Project info 1, Project info 1, New Info...

Results: Unpaired t test of TNF, Unpaired t test of TNF, Unpaired t test of TNF, Unpaired t test of TNF, Unpaired t test of TNF, Unpaired t test of IL-6, **Unpaired t test of IL-6**, Unpaired t test of IL-6, Unpaired t test of IL-6, Unpaired t test of IL-6, Unpaired t test of IL-10, Unpaired t test of IL-10, Unpaired t test of IL-10, Unpaired t test of IL-10, Unpaired t test of IL-10, Unpaired t test of IL-8, Unpaired t test of IL-8

Family: IL-6, Unpaired t test, IL-6

Unpaired t test

1	Table Analyzed	IL-6
2		
3	Column D	P1+Feb
4	vs.	vs.
5	Column C	P1
6		
7	**Unpaired t test**	
8	P value	0.0482
9	P value summary	*
10	Significantly different (P < 0.05)?	Yes
11	One- or two-tailed P value?	Two-tailed
12	t, df	t=2.812, df=4
13		
14	**How big is the difference?**	
15	Mean of column C	209.3
16	Mean of column D	142.4
17	Difference between means (D - C)	-66.93 ± 23.8[illegible]
18	95% confidence interval	-133.0 to -0.8[illegible]
19	R squared (eta squared)	0.6641
20		
21	**F test to compare variances**	
22	F, DFn, Dfd	1.814, 2, 2
23	P value	0.7108
24	P value summary	ns
25	Significantly different (P < 0.05)?	No
26		
27	**Data analyzed**	
28	Sample size, column C	3
29	Sample size, column D	3

Unpaired t test of IL-6 — Row 9, Column A

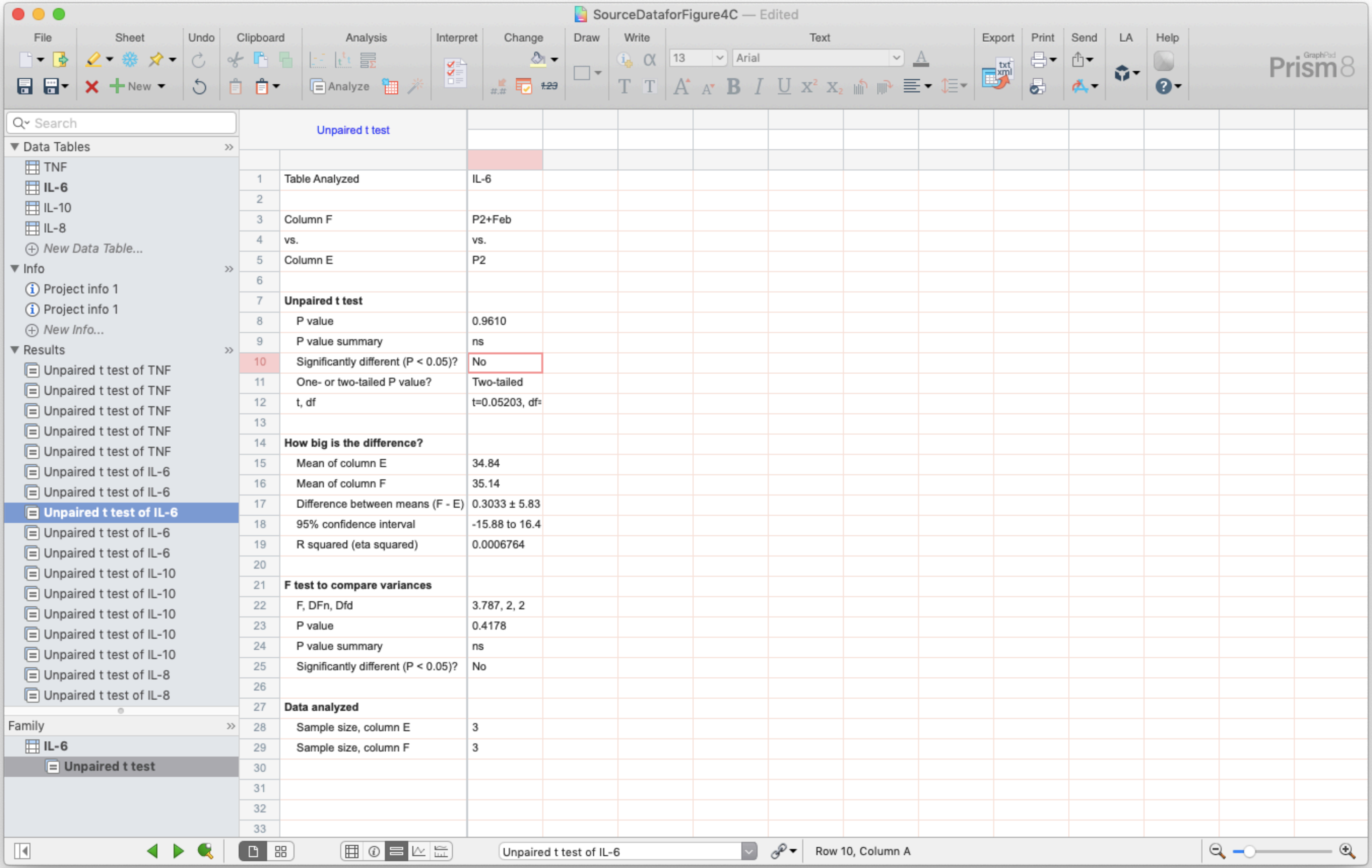

	Unpaired t test	
1	Table Analyzed	IL-6
2		
3	Column F	P2+Feb
4	vs.	vs.
5	Column E	P2
6		
7	**Unpaired t test**	
8	P value	0.9610
9	P value summary	ns
10	Significantly different (P < 0.05)?	No
11	One- or two-tailed P value?	Two-tailed
12	t, df	t=0.05203, df=
13		
14	**How big is the difference?**	
15	Mean of column E	34.84
16	Mean of column F	35.14
17	Difference between means (F - E)	0.3033 ± 5.83
18	95% confidence interval	-15.88 to 16.4
19	R squared (eta squared)	0.0006764
20		
21	**F test to compare variances**	
22	F, DFn, Dfd	3.787, 2, 2
23	P value	0.4178
24	P value summary	ns
25	Significantly different (P < 0.05)?	No
26		
27	**Data analyzed**	
28	Sample size, column E	3
29	Sample size, column F	3
30		
31		
32		
33		

SourceDataforFigure4C — Edited

- Data Tables
  - TNF
  - **IL-6**
  - IL-10
  - IL-8
  - New Data Table...
- Info
  - Project info 1
  - Project info 1
  - New Info...
- Results
  - Unpaired t test of TNF
  - Unpaired t test of TNF
  - Unpaired t test of TNF
  - Unpaired t test of TNF
  - Unpaired t test of TNF
  - Unpaired t test of IL-6
  - Unpaired t test of IL-6
  - Unpaired t test of IL-6
  - **Unpaired t test of IL-6**
  - Unpaired t test of IL-6
  - Unpaired t test of IL-10
  - Unpaired t test of IL-10
  - Unpaired t test of IL-10
  - Unpaired t test of IL-10
  - Unpaired t test of IL-10
  - Unpaired t test of IL-8
  - Unpaired t test of IL-8
- Family
  - **IL-6**
    - **Unpaired t test**

Unpaired t test

	Unpaired t test	
1	Table Analyzed	IL-6
2		
3	Column H	P3+Feb
4	vs.	vs.
5	Column G	P3
6		
7	**Unpaired t test**	
8	P value	0.0871
9	P value summary	ns
10	Significantly different (P < 0.05)?	No
11	One- or two-tailed P value?	Two-tailed
12	t, df	t=2.255, df=4
13		
14	**How big is the difference?**	
15	Mean of column G	8.360
16	Mean of column H	4.913
17	Difference between means (H - G) ± SEM	-3.447 ± 1.52[illegible]
18	95% confidence interval	-7.690 to 0.79[illegible]
19	R squared (eta squared)	0.5597
20		
21	**F test to compare variances**	
22	F, DFn, Dfd	2.160, 2, 2
23	P value	0.6329
24	P value summary	ns
25	Significantly different (P < 0.05)?	No
26		
27	**Data analyzed**	
28	Sample size, column G	3
29	Sample size, column H	3

Unpaired t test of IL-6 — Row 1, Column A

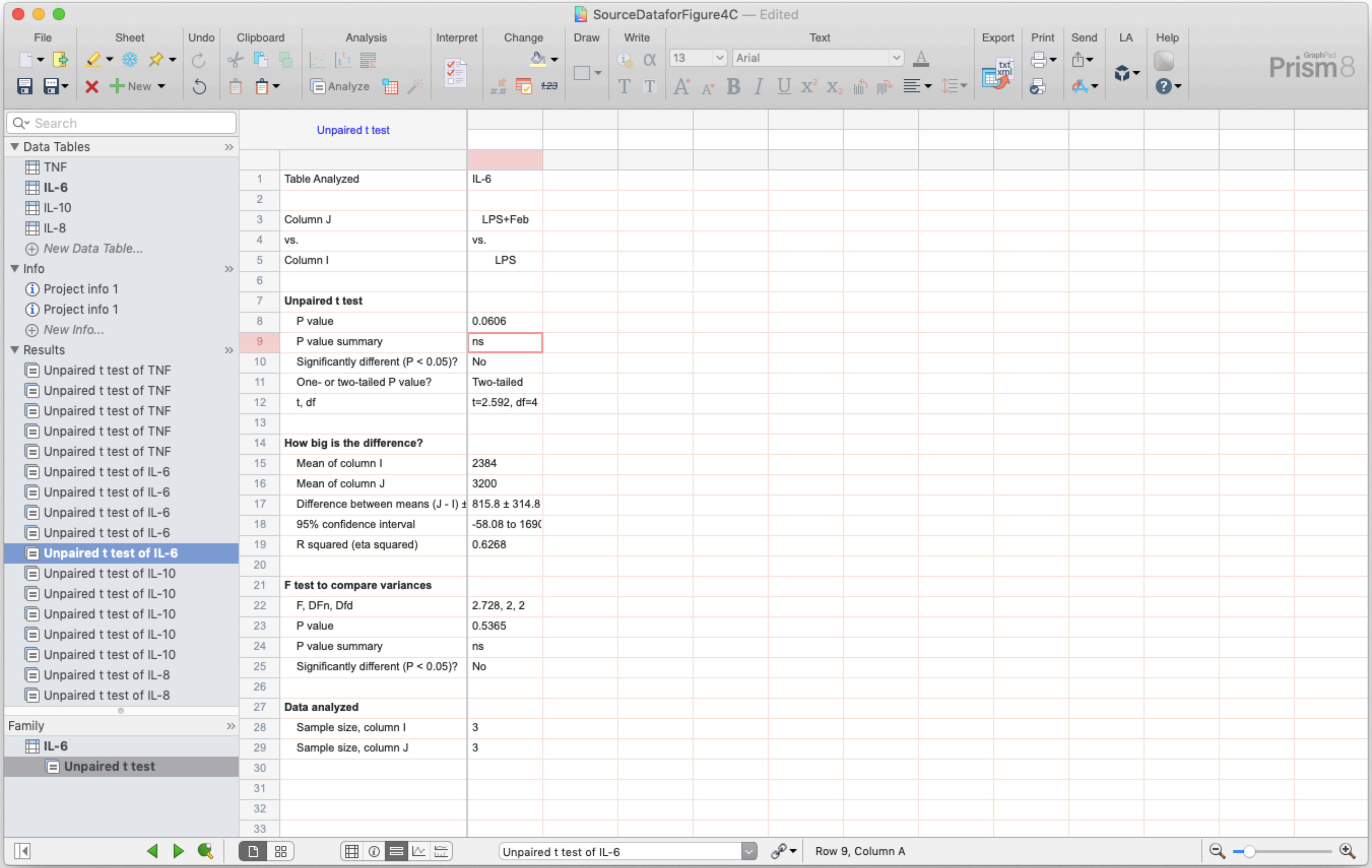

1	Table Analyzed	IL-6
2		
3	Column J	LPS+Feb
4	vs.	vs.
5	Column I	LPS
6		
7	**Unpaired t test**	
8	P value	0.0606
9	P value summary	ns
10	Significantly different (P < 0.05)?	No
11	One- or two-tailed P value?	Two-tailed
12	t, df	t=2.592, df=4
13		
14	**How big is the difference?**	
15	Mean of column I	2384
16	Mean of column J	3200
17	Difference between means (J - I) ±	815.8 ± 314.8
18	95% confidence interval	-58.08 to 169[illegible]
19	R squared (eta squared)	0.6268
20		
21	**F test to compare variances**	
22	F, DFn, Dfd	2.728, 2, 2
23	P value	0.5365
24	P value summary	ns
25	Significantly different (P < 0.05)?	No
26		
27	**Data analyzed**	
28	Sample size, column I	3
29	Sample size, column J	3

SourceDataforFigure4C — Edited

Data Tables
- TNF
- IL-6
- **IL-10**
- IL-8
- New Data Table...

Info
- Project info 1
- Project info 1
- New Info...

Results
- Unpaired t test of TNF
- Unpaired t test of TNF
- Unpaired t test of TNF
- Unpaired t test of TNF
- Unpaired t test of TNF
- Unpaired t test of IL-6
- Unpaired t test of IL-6
- Unpaired t test of IL-6
- Unpaired t test of IL-6
- Unpaired t test of IL-6
- **Unpaired t test of IL-10**
- Unpaired t test of IL-10
- Unpaired t test of IL-10
- Unpaired t test of IL-10
- Unpaired t test of IL-10
- Unpaired t test of IL-8
- Unpaired t test of IL-8

Family
- **IL-10**
  - **Unpaired t test**

	Unpaired t test	
1	Table Analyzed	IL-10
2		
3	Column B	HP+Feb
4	vs.	vs.
5	Column A	HP
6		
7	**Unpaired t test**	
8	P value	0.3739
9	P value summary	ns
10	Significantly different (P < 0.05)?	No
11	One- or two-tailed P value?	Two-tailed
12	t, df	t=1.000, df=4
13		
14	**How big is the difference?**	
15	Mean of column A	0.4233
16	Mean of column B	0.000
17	Difference between means (B - A)	-0.4233 ± 0.42
18	95% confidence interval	-1.599 to 0.75
19	R squared (eta squared)	0.2000
20		
21	**F test to compare variances**	
22	F, DFn, Dfd	Infinity, 2, 2
23	P value	<0.0001
24	P value summary	****
25	Significantly different (P < 0.05)?	Yes
26		
27	**Data analyzed**	
28	Sample size, column A	3
29	Sample size, column B	3
30		
31		
32		
33		

Unpaired t test of IL-10 | Row 1, Column A

SourceDataforFigure4C — Edited

Data Tables: TNF, IL-6, IL-10, IL-8

Results: Unpaired t test of TNF (×5), Unpaired t test of IL-6 (×5), Unpaired t test of IL-10 (×6), Unpaired t test of IL-8 (×2)

Family: IL-10 — Unpaired t test

	Unpaired t test	
1	Table Analyzed	IL-10
2		
3	Column D	P1+Feb
4	vs.	vs.
5	Column C	P1
6		
7	**Unpaired t test**	
8	P value	0.0744
9	P value summary	ns
10	Significantly different (P < 0.05)?	No
11	One- or two-tailed P value?	Two-tailed
12	t, df	t=2.400, df=4
13		
14	**How big is the difference?**	
15	Mean of column C	4.373
16	Mean of column D	2.453
17	Difference between means (D - C)	-1.920 ± 0.80[illegible]
18	95% confidence interval	-4.141 to 0.30[illegible]
19	R squared (eta squared)	0.5901
20		
21	**F test to compare variances**	
22	F, DFn, Dfd	1.387, 2, 2
23	P value	0.8377
24	P value summary	ns
25	Significantly different (P < 0.05)?	No
26		
27	**Data analyzed**	
28	Sample size, column C	3
29	Sample size, column D	3

Unpaired t test of IL-10 — Row 1, Column A

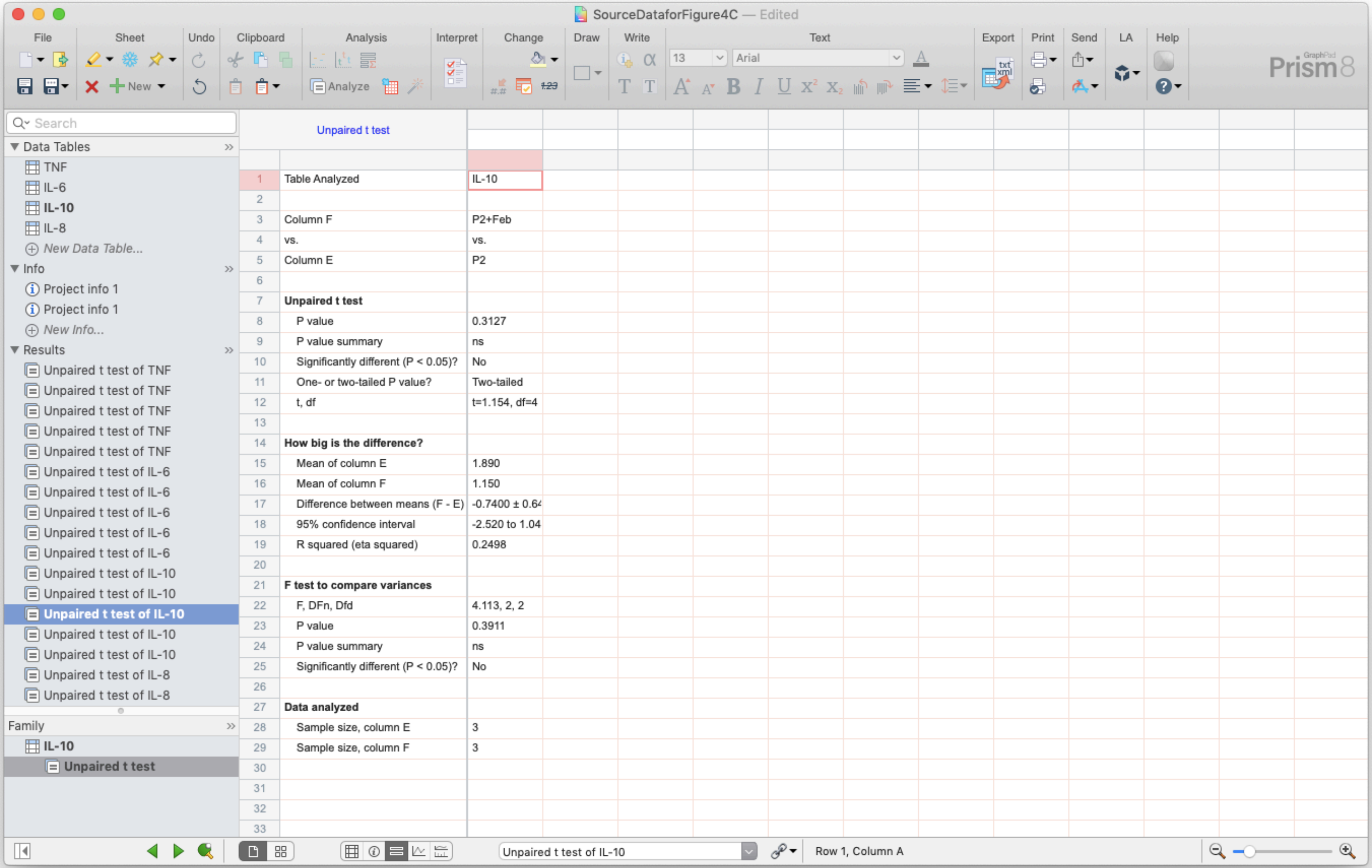

	Unpaired t test	
1	Table Analyzed	IL-10
2		
3	Column F	P2+Feb
4	vs.	vs.
5	Column E	P2
6		
7	**Unpaired t test**	
8	P value	0.3127
9	P value summary	ns
10	Significantly different (P < 0.05)?	No
11	One- or two-tailed P value?	Two-tailed
12	t, df	t=1.154, df=4
13		
14	**How big is the difference?**	
15	Mean of column E	1.890
16	Mean of column F	1.150
17	Difference between means (F - E)	-0.7400 ± 0.64
18	95% confidence interval	-2.520 to 1.04
19	R squared (eta squared)	0.2498
20		
21	**F test to compare variances**	
22	F, DFn, Dfd	4.113, 2, 2
23	P value	0.3911
24	P value summary	ns
25	Significantly different (P < 0.05)?	No
26		
27	**Data analyzed**	
28	Sample size, column E	3
29	Sample size, column F	3
30		
31		
32		
33		

SourceDataforFigure4C — Edited

Unpaired t test

1	Table Analyzed	IL-10
2		
3	Column H	P3+Feb
4	vs.	vs.
5	Column G	P3
6		
7	**Unpaired t test**	
8	P value	0.9023
9	P value summary	ns
10	Significantly different (P < 0.05)?	No
11	One- or two-tailed P value?	Two-tailed
12	t, df	t=0.1308, df=[illegible]
13		
14	**How big is the difference?**	
15	Mean of column G	0.4233
16	Mean of column H	0.5100
17	Difference between means (H - G)	0.08667 ± 0.6[illegible]
18	95% confidence interval	-1.754 to 1.92[illegible]
19	R squared (eta squared)	0.004256
20		
21	**F test to compare variances**	
22	F, DFn, Dfd	1.451, 2, 2
23	P value	0.8159
24	P value summary	ns
25	Significantly different (P < 0.05)?	No
26		
27	**Data analyzed**	
28	Sample size, column G	3
29	Sample size, column H	3

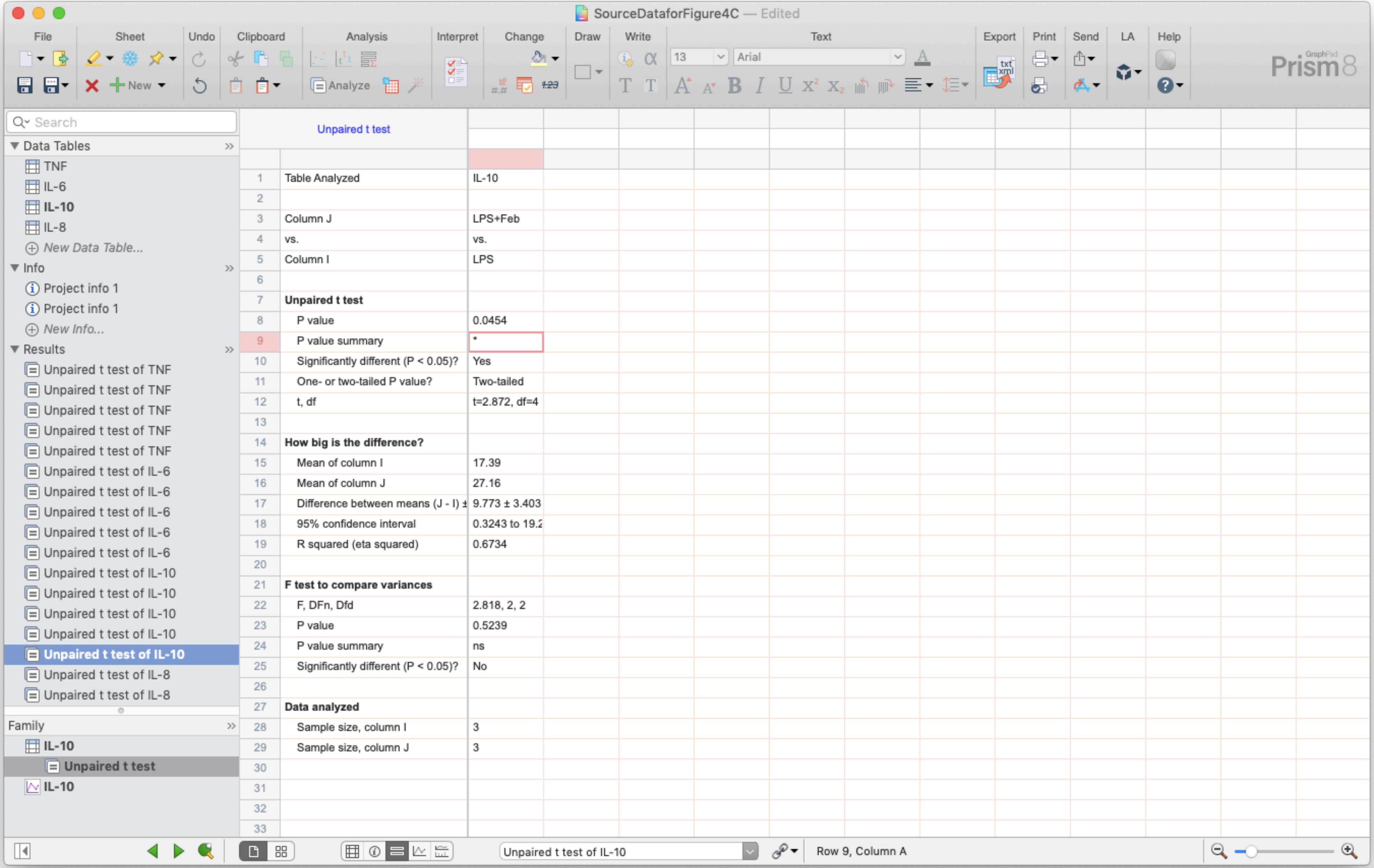

		Unpaired t test	
1	Table Analyzed	IL-10	
2			
3	Column J	LPS+Feb	
4	vs.	vs.	
5	Column I	LPS	
6			
7	**Unpaired t test**		
8	P value	0.0454	
9	P value summary	*	
10	Significantly different (P < 0.05)?	Yes	
11	One- or two-tailed P value?	Two-tailed	
12	t, df	t=2.872, df=4	
13			
14	**How big is the difference?**		
15	Mean of column I	17.39	
16	Mean of column J	27.16	
17	Difference between means (J - I) ±	9.773 ± 3.403	
18	95% confidence interval	0.3243 to 19.2	
19	R squared (eta squared)	0.6734	
20			
21	**F test to compare variances**		
22	F, DFn, Dfd	2.818, 2, 2	
23	P value	0.5239	
24	P value summary	ns	
25	Significantly different (P < 0.05)?	No	
26			
27	**Data analyzed**		
28	Sample size, column I	3	
29	Sample size, column J	3	

SourceDataforFigure4C — Edited

Data Tables
- TNF
- IL-6
- IL-10
- IL-8
- New Data Table...

Info
- Project info 1
- Project info 1
- New Info...

Results
- Unpaired t test of TNF
- Unpaired t test of TNF
- Unpaired t test of TNF
- Unpaired t test of TNF
- Unpaired t test of TNF
- Unpaired t test of IL-6
- Unpaired t test of IL-6
- Unpaired t test of IL-6
- Unpaired t test of IL-6
- Unpaired t test of IL-6
- Unpaired t test of IL-10
- Unpaired t test of IL-10
- Unpaired t test of IL-10
- Unpaired t test of IL-10
- Unpaired t test of IL-10
- Unpaired t test of IL-8
- Unpaired t test of IL-8

Family
- IL-8
  - Unpaired t test

Unpaired t test

1	Table Analyzed	IL-8
2		
3	Column B	HP+Feb
4	vs.	vs.
5	Column A	HP
6		
7	**Unpaired t test**	
8	P value	0.6005
9	P value summary	ns
10	Significantly different (P < 0.05)?	No
11	One- or two-tailed P value?	Two-tailed
12	t, df	t=0.5678, df=[illegible]
13		
14	**How big is the difference?**	
15	Mean of column A	1164
16	Mean of column B	1072
17	Difference between means (B - A)	-91.85 ± 161.[illegible]
18	95% confidence interval	-541.0 to 357.[illegible]
19	R squared (eta squared)	0.07458
20		
21	**F test to compare variances**	
22	F, DFn, Dfd	2.433, 2, 2
23	P value	0.5826
24	P value summary	ns
25	Significantly different (P < 0.05)?	No
26		
27	**Data analyzed**	
28	Sample size, column A	3
29	Sample size, column B	3

Unpaired t test of IL-8 — Row 1, Column A

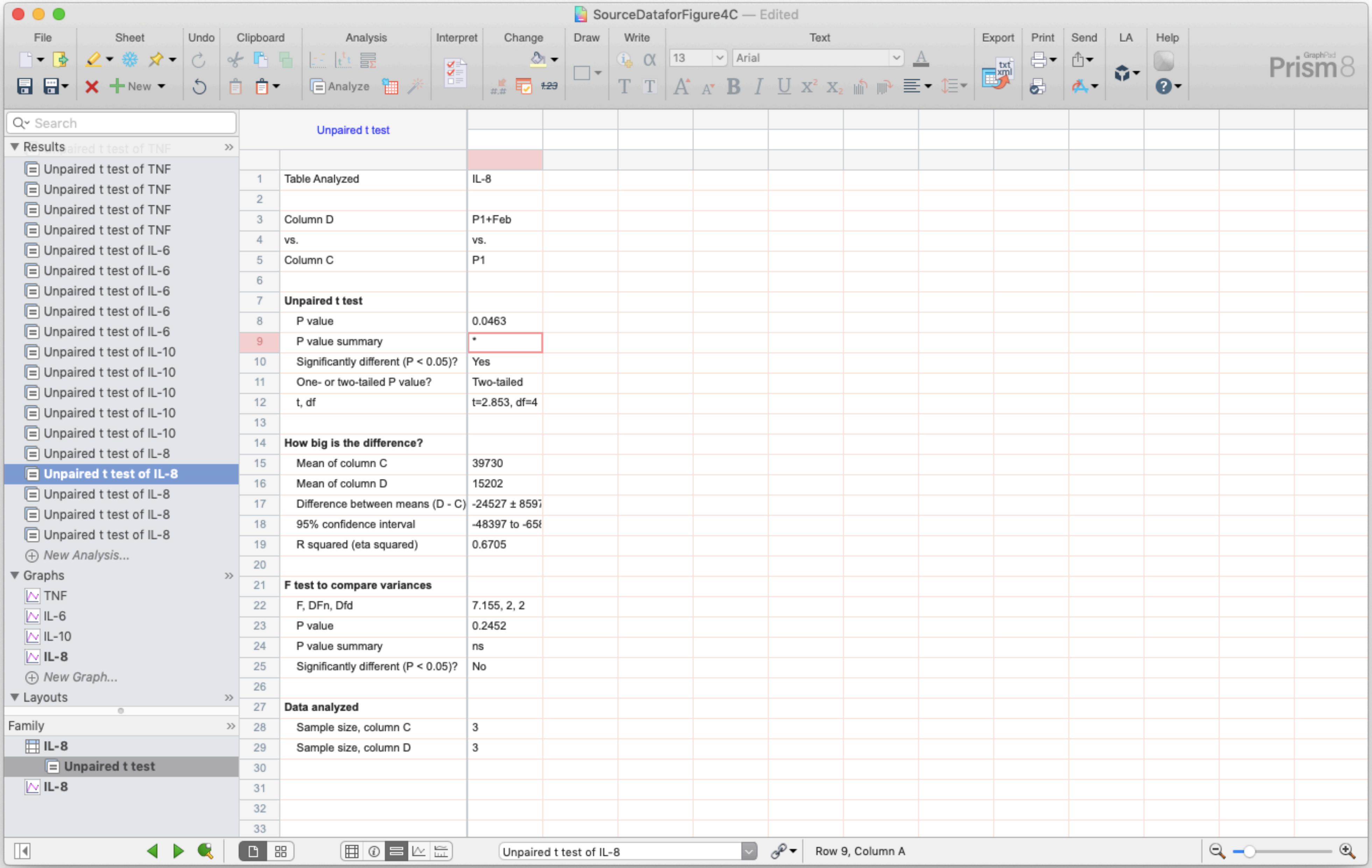

	Unpaired t test	
1	Table Analyzed	IL-8
2		
3	Column D	P1+Feb
4	vs.	vs.
5	Column C	P1
6		
7	**Unpaired t test**	
8	P value	0.0463
9	P value summary	*
10	Significantly different (P < 0.05)?	Yes
11	One- or two-tailed P value?	Two-tailed
12	t, df	t=2.853, df=4
13		
14	**How big is the difference?**	
15	Mean of column C	39730
16	Mean of column D	15202
17	Difference between means (D - C)	-24527 ± 8597
18	95% confidence interval	-48397 to -65[illegible]
19	R squared (eta squared)	0.6705
20		
21	**F test to compare variances**	
22	F, DFn, Dfd	7.155, 2, 2
23	P value	0.2452
24	P value summary	ns
25	Significantly different (P < 0.05)?	No
26		
27	**Data analyzed**	
28	Sample size, column C	3
29	Sample size, column D	3
30		
31		
32		
33		

SourceDataforFigure4C — Edited

Unpaired t test

1	Table Analyzed	IL-8
2		
3	Column F	P2+Feb
4	vs.	vs.
5	Column E	P2
6		
7	**Unpaired t test**	
8	P value	0.4195
9	P value summary	ns
10	Significantly different (P < 0.05)?	No
11	One- or two-tailed P value?	Two-tailed
12	t, df	t=0.8990, df=4
13		
14	**How big is the difference?**	
15	Mean of column E	7135
16	Mean of column F	5786
17	Difference between means (F - E)	-1349 ± 1501
18	95% confidence interval	-5516 to 2817
19	R squared (eta squared)	0.1681
20		
21	**F test to compare variances**	
22	F, DFn, Dfd	1.064, 2, 2
23	P value	0.9691
24	P value summary	ns
25	Significantly different (P < 0.05)?	No
26		
27	**Data analyzed**	
28	Sample size, column E	3
29	Sample size, column F	3

Unpaired t test of IL-8 — Row 9, Column A

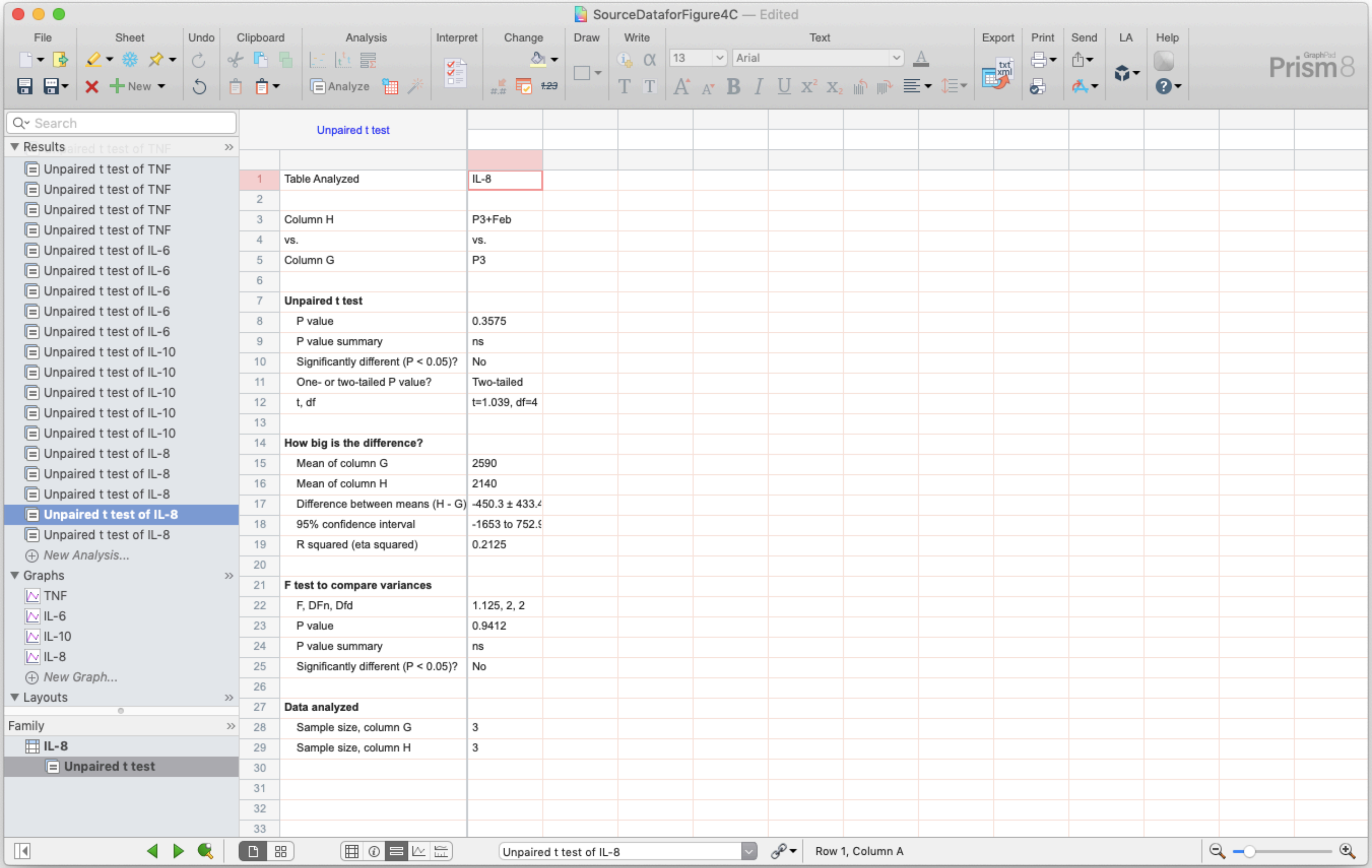

	Unpaired t test	A
1	Table Analyzed	IL-8
2		
3	Column H	P3+Feb
4	vs.	vs.
5	Column G	P3
6		
7	**Unpaired t test**	
8	P value	0.3575
9	P value summary	ns
10	Significantly different (P < 0.05)?	No
11	One- or two-tailed P value?	Two-tailed
12	t, df	t=1.039, df=4
13		
14	**How big is the difference?**	
15	Mean of column G	2590
16	Mean of column H	2140
17	Difference between means (H - G)	-450.3 ± 433.4
18	95% confidence interval	-1653 to 752.[illegible]
19	R squared (eta squared)	0.2125
20		
21	**F test to compare variances**	
22	F, DFn, Dfd	1.125, 2, 2
23	P value	0.9412
24	P value summary	ns
25	Significantly different (P < 0.05)?	No
26		
27	**Data analyzed**	
28	Sample size, column G	3
29	Sample size, column H	3

SourceDataforFigure4C — Edited

Unpaired t test

1	Table Analyzed	IL-8
2		
3	Column J	LPS+Feb
4	vs.	vs.
5	Column I	LPS
6		
7	**Unpaired t test**	
8	P value	0.0334
9	P value summary	*
10	Significantly different (P < 0.05)?	Yes
11	One- or two-tailed P value?	Two-tailed
12	t, df	t=3.185, df=4
13		
14	**How big is the difference?**	
15	Mean of column I	68718
16	Mean of column J	256675
17	Difference between means (J - I) ±	187958 ± 590
18	95% confidence interval	24114 to 3518
19	R squared (eta squared)	0.7172
20		
21	**F test to compare variances**	
22	F, DFn, Dfd	88.80, 2, 2
23	P value	0.0223
24	P value summary	*
25	Significantly different (P < 0.05)?	Yes
26		
27	**Data analyzed**	
28	Sample size, column I	3
29	Sample size, column J	3

Results
- Unpaired t test of TNF
- Unpaired t test of TNF
- Unpaired t test of TNF
- Unpaired t test of TNF
- Unpaired t test of IL-6
- Unpaired t test of IL-6
- Unpaired t test of IL-6
- Unpaired t test of IL-6
- Unpaired t test of IL-6
- Unpaired t test of IL-10
- Unpaired t test of IL-10
- Unpaired t test of IL-10
- Unpaired t test of IL-10
- Unpaired t test of IL-10
- Unpaired t test of IL-8
- Unpaired t test of IL-8
- Unpaired t test of IL-8
- Unpaired t test of IL-8
- **Unpaired t test of IL-8**

Graphs
- TNF
- IL-6
- IL-10
- **IL-8**

Family
- **IL-8**
- **Unpaired t test**
- **IL-8**

Row 9, Column A
