# Supplementary material for: Malaria inflammation by xanthine oxidase‐produced reactive oxygen species
Source: EMBO Mol Med. 2019 Jul 2;11(8):e9903. doi: 10.15252/emmm.201809903 (PMC6685105; doi:10.15252/emmm.201809903)
Supplement: Supplementary file 8 — Source Data for Figure 5 [file EMMM-11-e9903-s006.pdf]

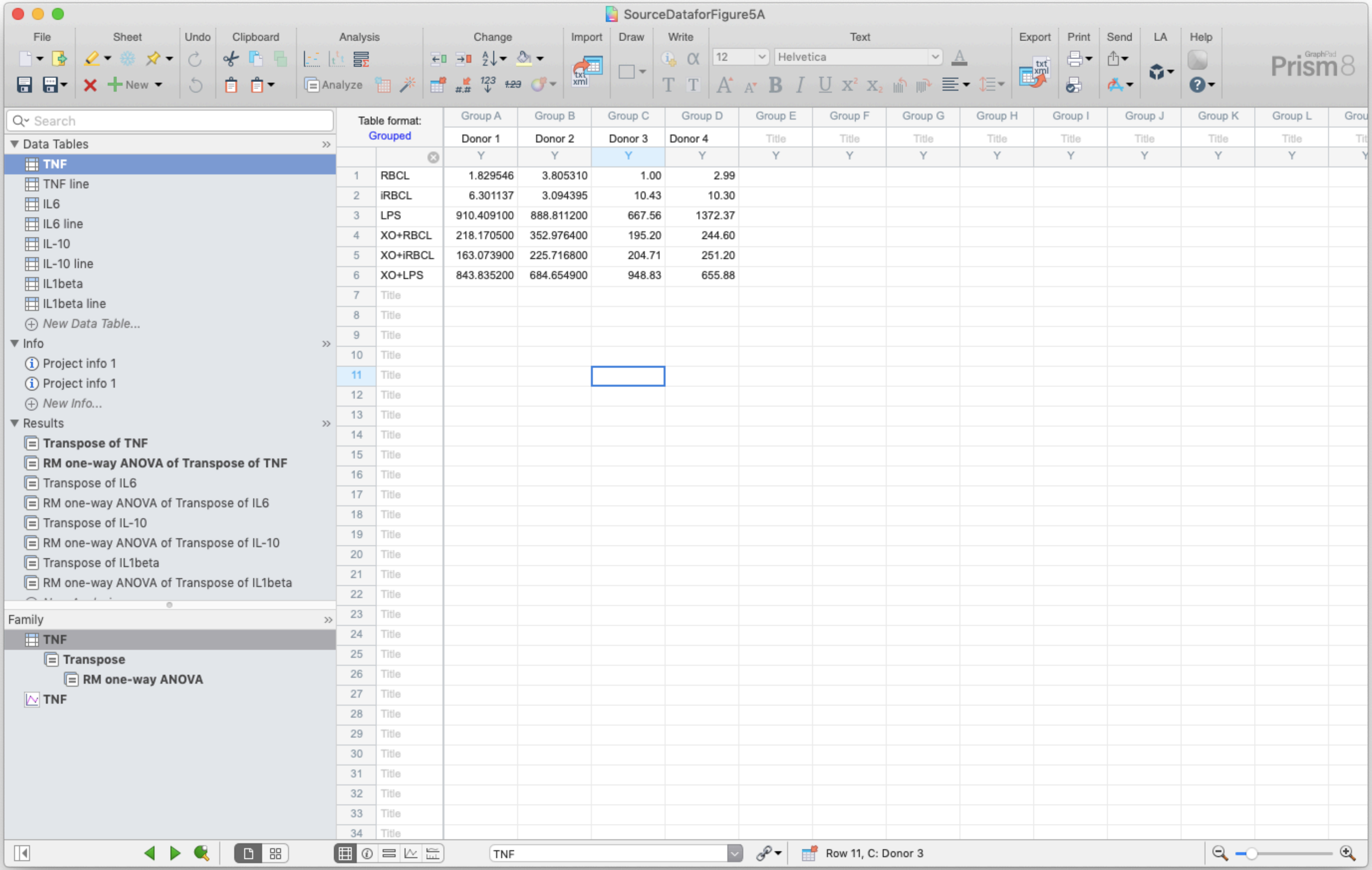

SourceDataforFigure5A

Table format: Grouped	Group A	Group B	Group C	Group D
	Donor 1	Donor 2	Donor 3	Donor 4
	Y	Y	Y	Y
RBCL	1.829546	3.805310	1.00	2.99
iRBCL	6.301137	3.094395	10.43	10.30
LPS	910.409100	888.811200	667.56	1372.37
XO+RBCL	218.170500	352.976400	195.20	244.60
XO+iRBCL	163.073900	225.716800	204.71	251.20
XO+LPS	843.835200	684.654900	948.83	655.88

SourceDataforFigure5A

Data Tables
- TNF
- TNF line
- IL6
- IL6 line
- IL-10
- IL-10 line
- IL1beta
- IL1beta line
- New Data Table...

Info
- Project info 1
- Project info 1
- New Info...

Results
- Transpose of TNF
- RM one-way ANOVA of Transpose of TNF
- Transpose of IL6
- RM one-way ANOVA of Transpose of IL6
- Transpose of IL-10
- RM one-way ANOVA of Transpose of IL-10
- Transpose of IL1beta
- RM one-way ANOVA of Transpose of IL1beta

Family
- TNF
  - Transpose
    - RM one-way ANOVA
- TNF

Table format: Grouped

		Group A	Group B	Group C	Group D
		Donor 1	Donor 2	Donor 3	Donor 4
		Y	Y	Y	Y
1	RBCL	1.829546	3.805310	1.00	2.99
2	iRBCL	6.301137	3.094395	10.43	10.30
3	LPS	910.409100	888.811200	667.56	1372.37
4	XO+RBCL	218.170500	352.976400	195.20	244.60
5	XO+iRBCL	163.073900	225.716800	204.71	251.20
6	XO+LPS	843.835200	684.654900	948.83	655.88

TNF

Row 11, C: Donor 3

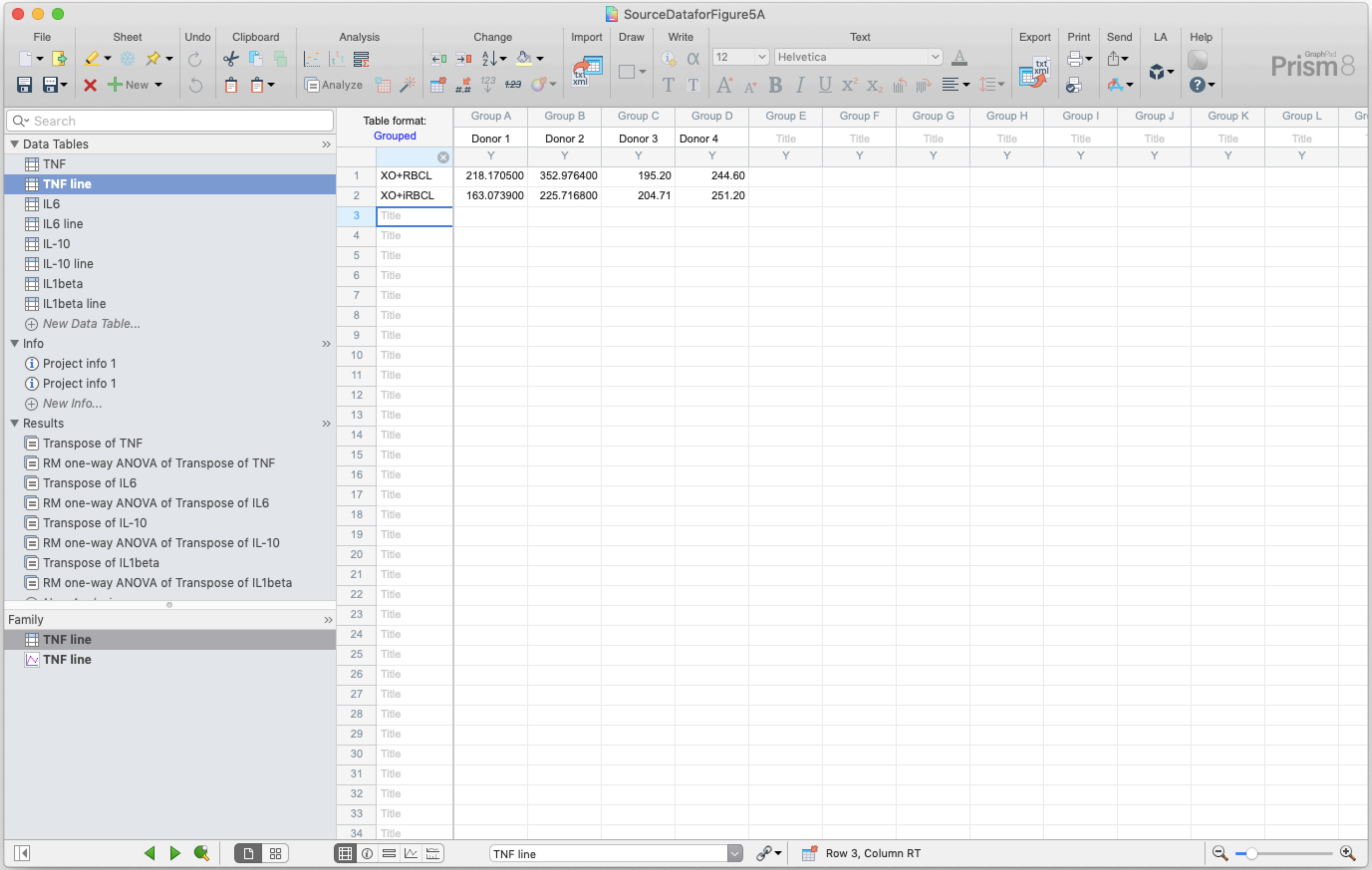

Table format: Grouped	Group A	Group B	Group C	Group D
	Donor 1	Donor 2	Donor 3	Donor 4
	Y	Y	Y	Y
1 XO+RBCL	218.170500	352.976400	195.20	244.60
2 XO+iRBCL	163.073900	225.716800	204.71	251.20

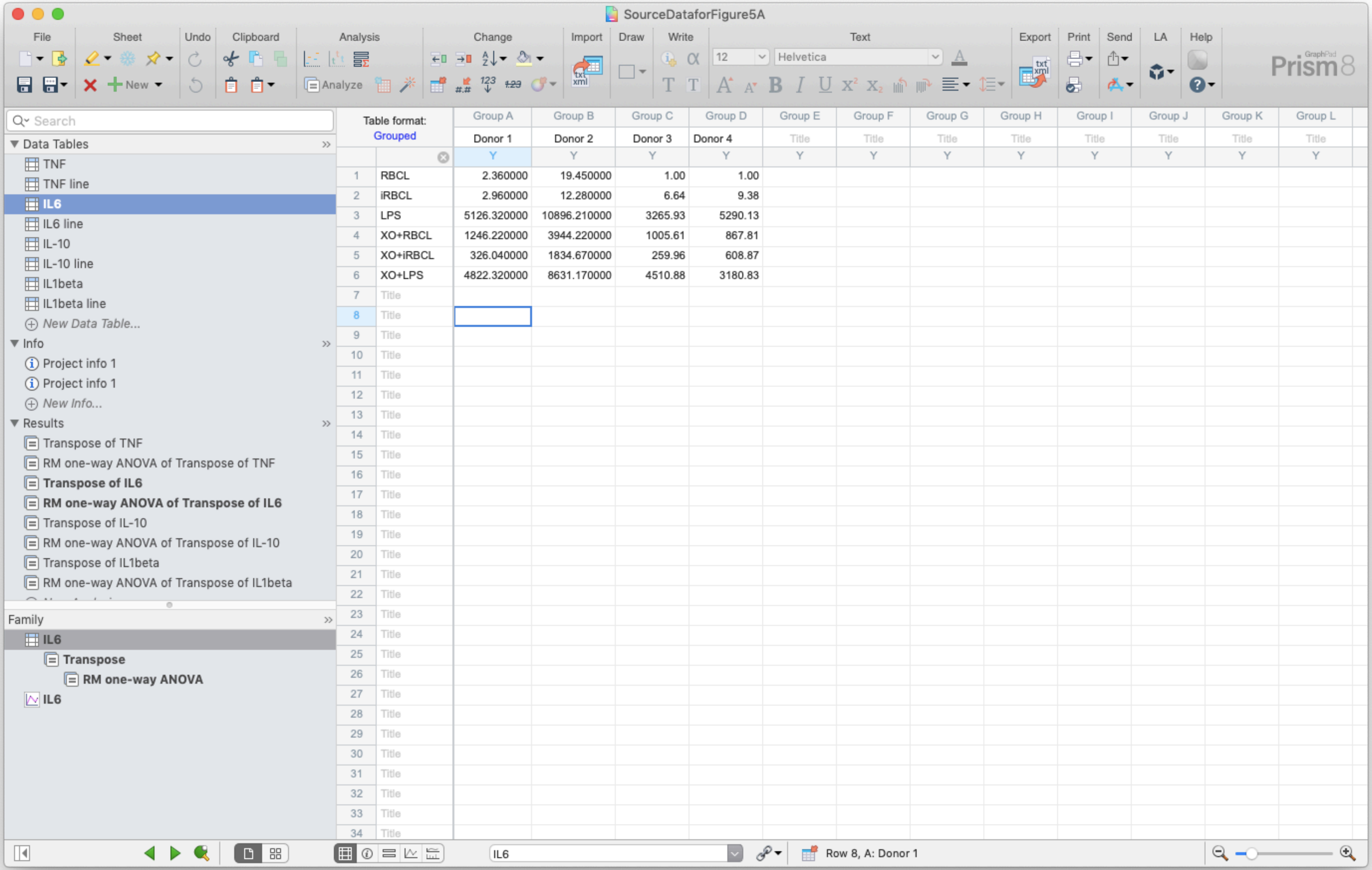

		Donor 1	Donor 2	Donor 3	Donor 4
1	RBCL	2.360000	19.450000	1.00	1.00
2	iRBCL	2.960000	12.280000	6.64	9.38
3	LPS	5126.320000	10896.210000	3265.93	5290.13
4	XO+RBCL	1246.220000	3944.220000	1005.61	867.81
5	XO+iRBCL	326.040000	1834.670000	259.96	608.87
6	XO+LPS	4822.320000	8631.170000	4510.88	3180.83

SourceDataforFigure5A

Table format: Grouped

IL6 line

	Group A	Group B	Group C	Group D
	Donor 1	Donor 2	Donor 3	Donor 4
XO+RBCL	1246.220000	3944.220000	1005.61	867.81
XO+iRBCL	326.040000	1834.670000	259.96	608.87

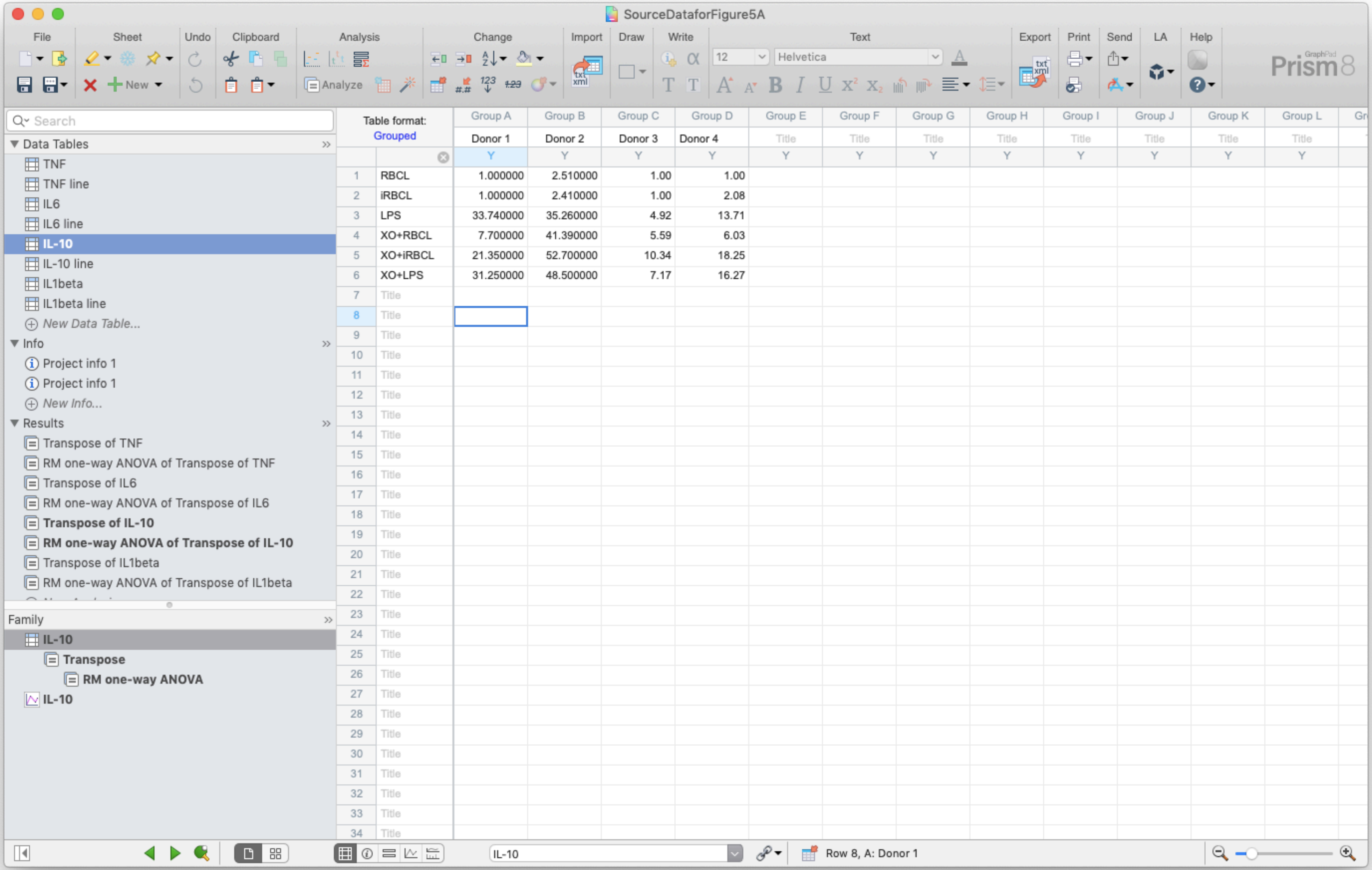

SourceDataforFigure5A

IL-10

	Donor 1	Donor 2	Donor 3	Donor 4
RBCL	1.000000	2.510000	1.00	1.00
iRBCL	1.000000	2.410000	1.00	2.08
LPS	33.740000	35.260000	4.92	13.71
XO+RBCL	7.700000	41.390000	5.59	6.03
XO+iRBCL	21.350000	52.700000	10.34	18.25
XO+LPS	31.250000	48.500000	7.17	16.27

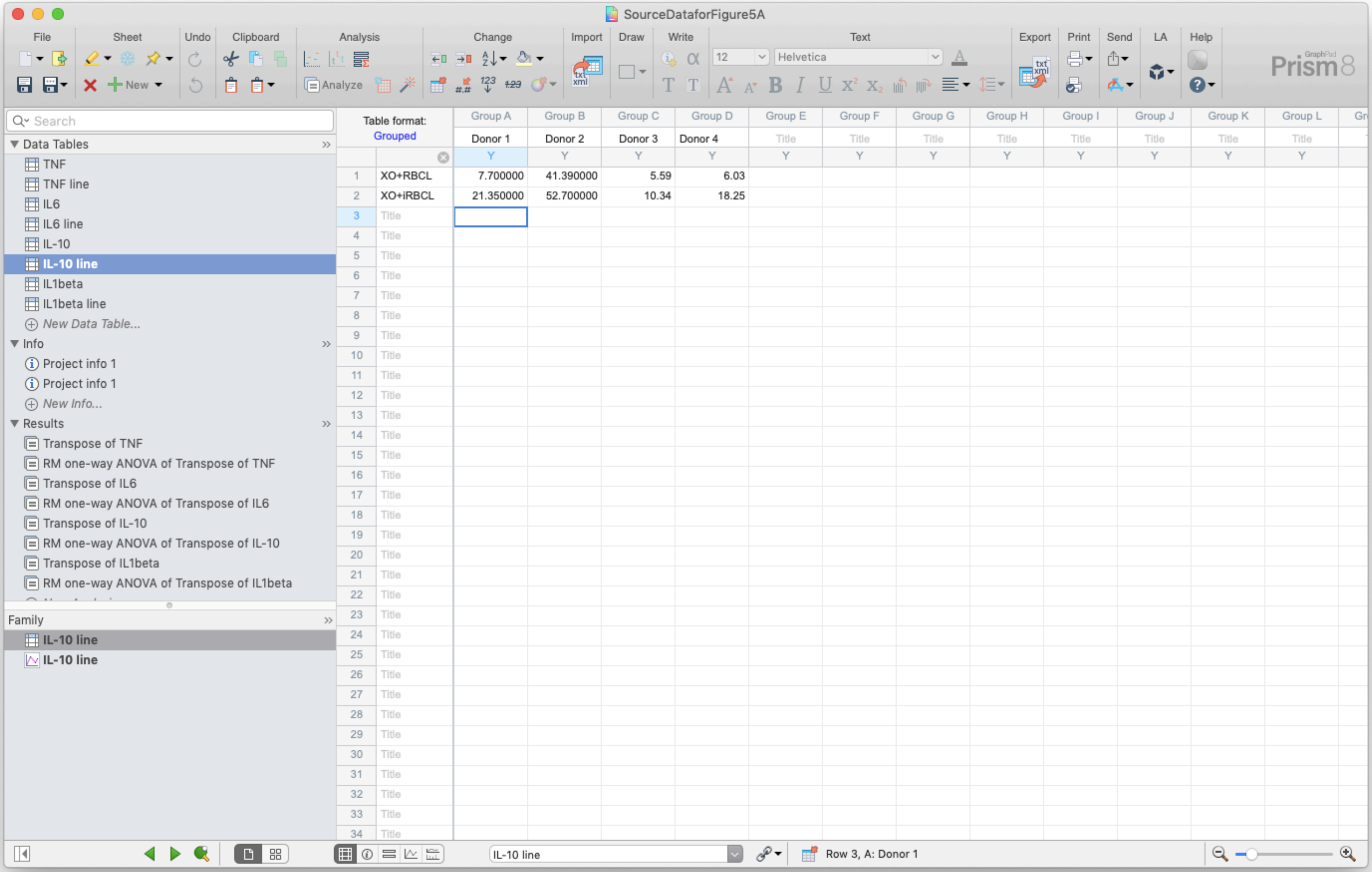

	Group A	Group B	Group C	Group D
	Donor 1	Donor 2	Donor 3	Donor 4
XO+RBCL	7.700000	41.390000	5.59	6.03
XO+iRBCL	21.350000	52.700000	10.34	18.25

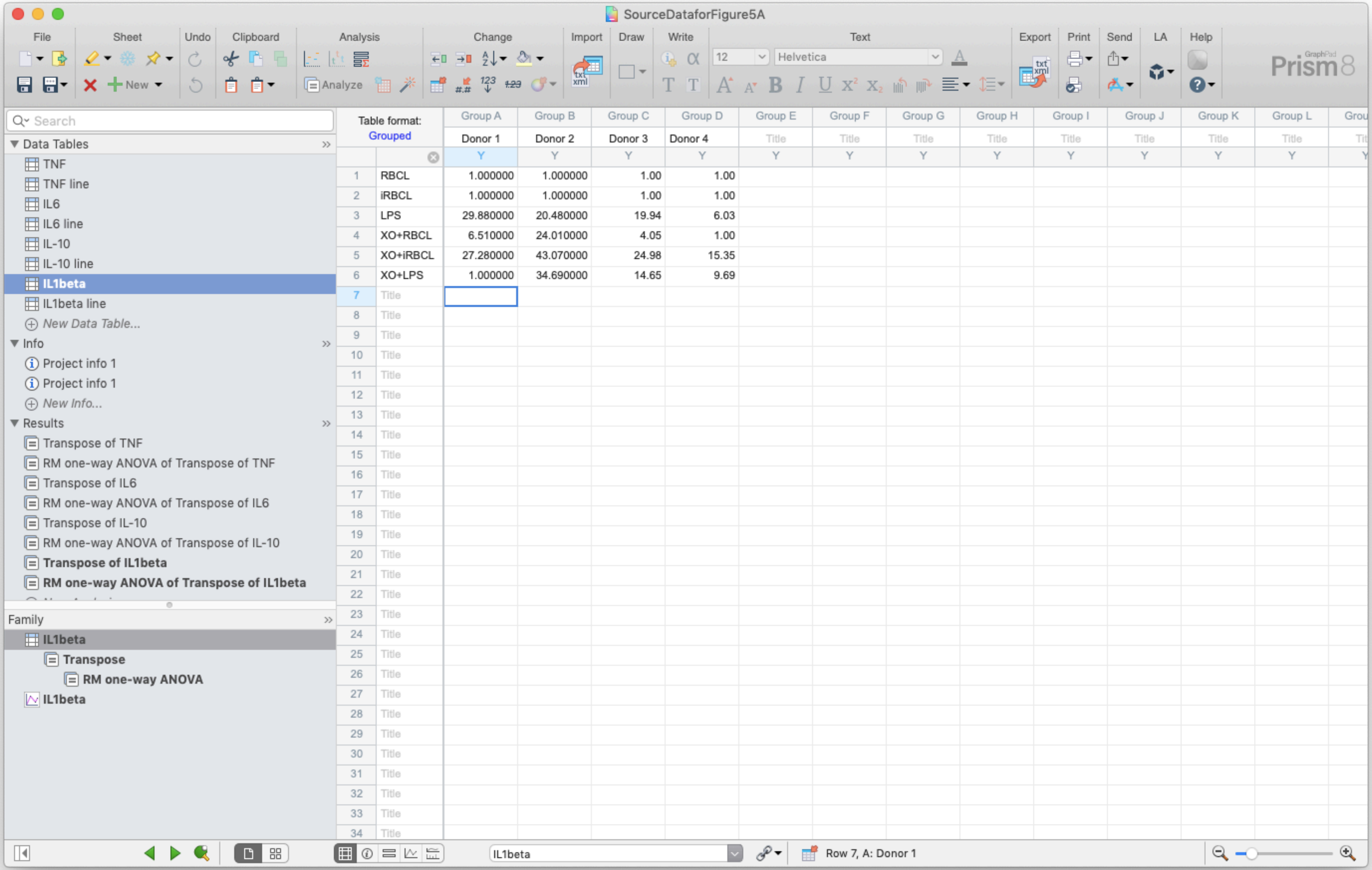

		Group A	Group B	Group C	Group D
		Donor 1	Donor 2	Donor 3	Donor 4
1	RBCL	1.000000	1.000000	1.00	1.00
2	iRBCL	1.000000	1.000000	1.00	1.00
3	LPS	29.880000	20.480000	19.94	6.03
4	XO+RBCL	6.510000	24.010000	4.05	1.00
5	XO+iRBCL	27.280000	43.070000	24.98	15.35
6	XO+LPS	1.000000	34.690000	14.65	9.69

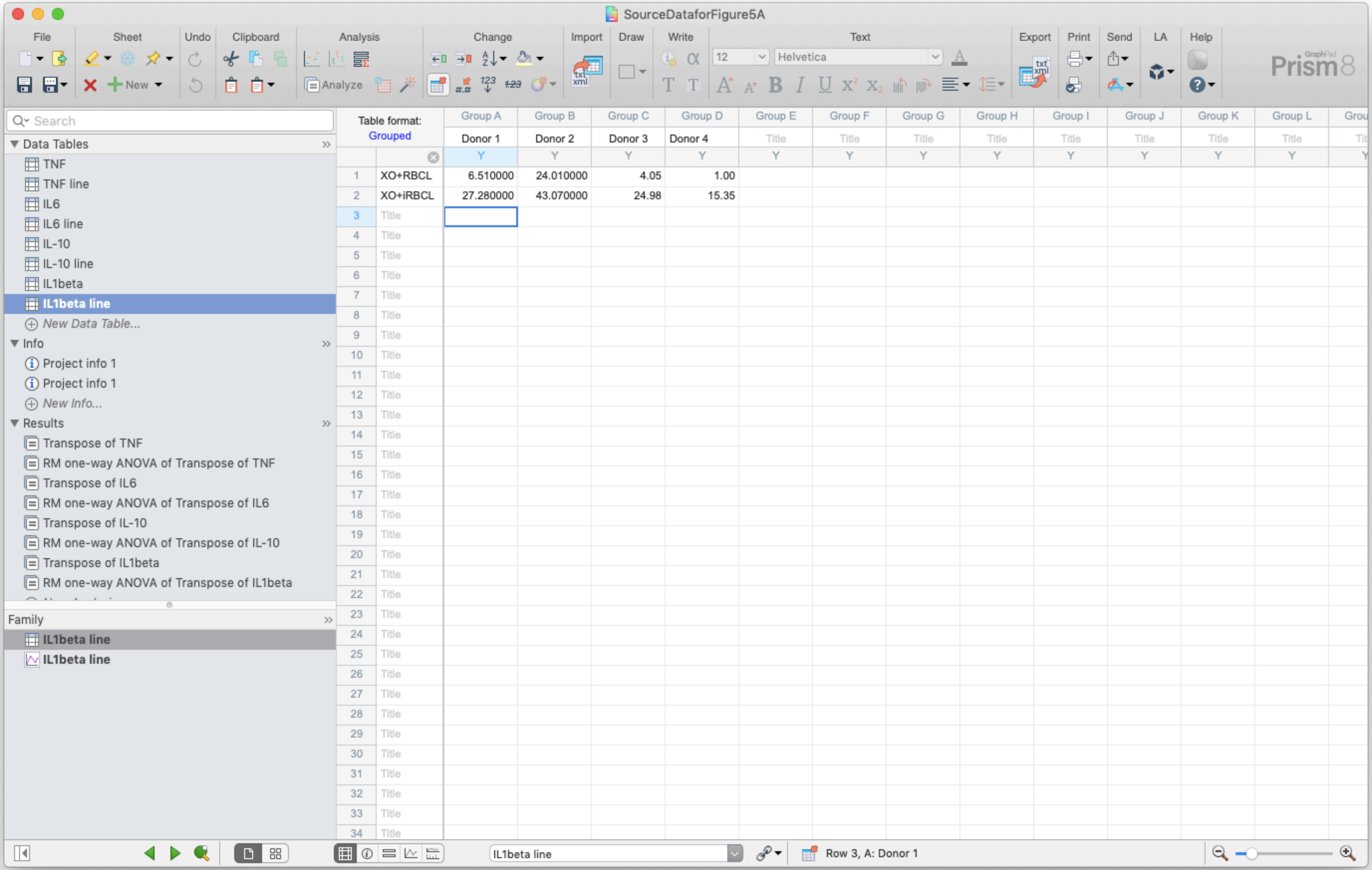

	Group A	Group B	Group C	Group D
	Donor 1	Donor 2	Donor 3	Donor 4
XO+RBCL	6.510000	24.010000	4.05	1.00
XO+iRBCL	27.280000	43.070000	24.98	15.35

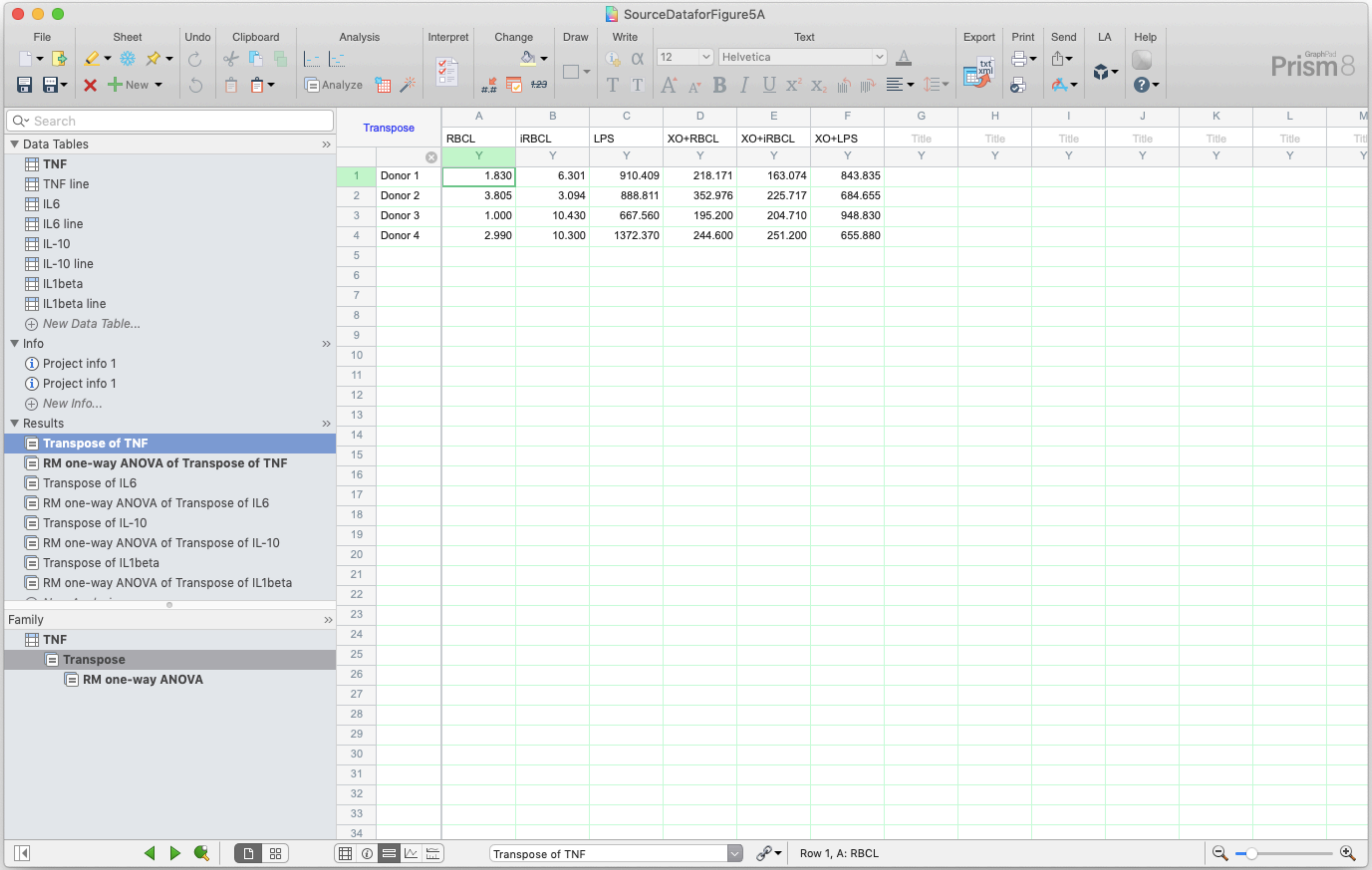

Transpose	A RBCL	B iRBCL	C LPS	D XO+RBCL	E XO+iRBCL	F XO+LPS
Donor 1	1.830	6.301	910.409	218.171	163.074	843.835
Donor 2	3.805	3.094	888.811	352.976	225.717	684.655
Donor 3	1.000	10.430	667.560	195.200	204.710	948.830
Donor 4	2.990	10.300	1372.370	244.600	251.200	655.880

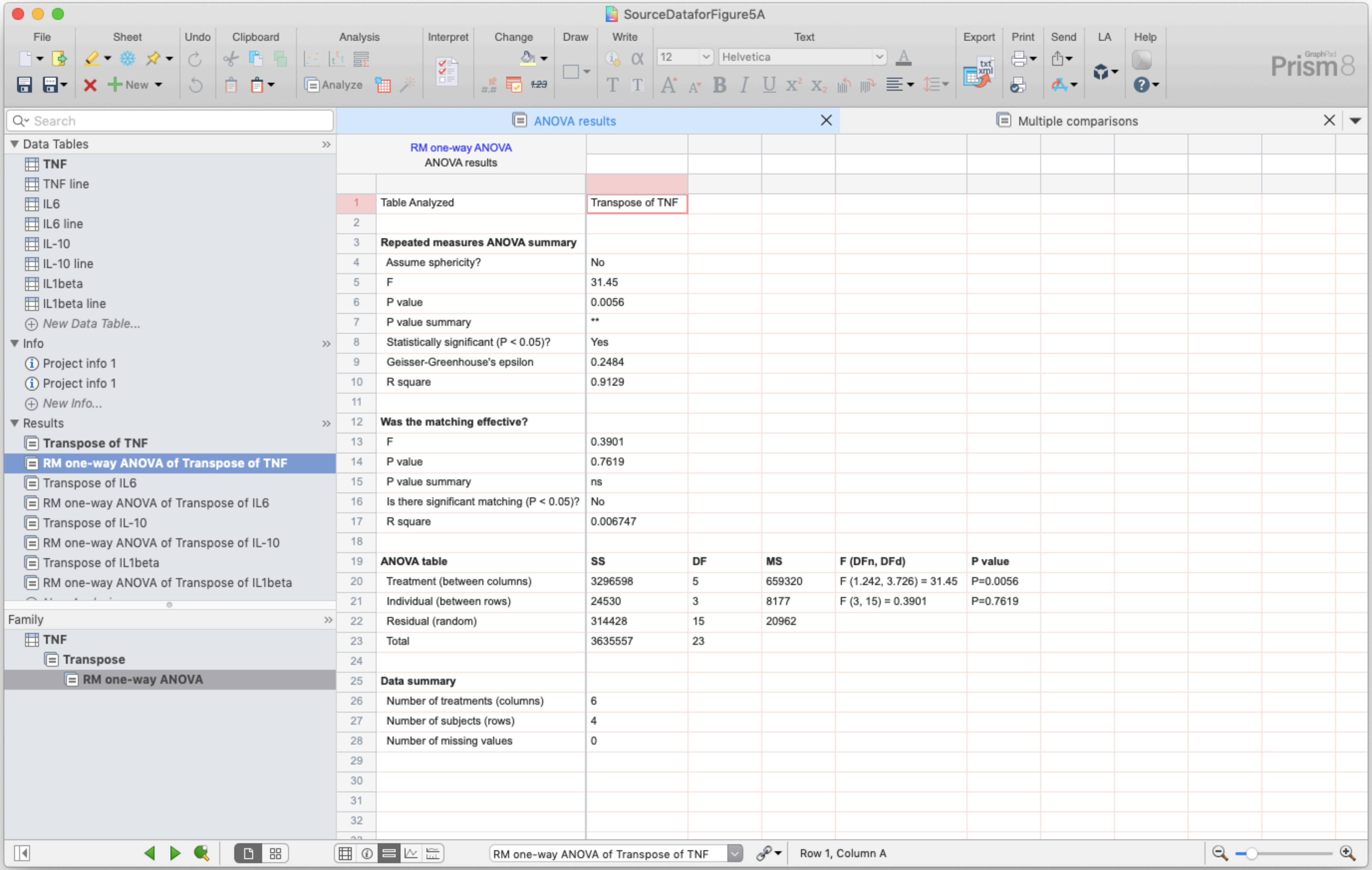

RM one-way ANOVA
ANOVA results

1	Table Analyzed	Transpose of TNF				
2						
3	**Repeated measures ANOVA summary**					
4	Assume sphericity?	No				
5	F	31.45				
6	P value	0.0056				
7	P value summary	**				
8	Statistically significant (P < 0.05)?	Yes				
9	Geisser-Greenhouse's epsilon	0.2484				
10	R square	0.9129				
11						
12	**Was the matching effective?**					
13	F	0.3901				
14	P value	0.7619				
15	P value summary	ns				
16	Is there significant matching (P < 0.05)?	No				
17	R square	0.006747				
18						
19	**ANOVA table**	**SS**	**DF**	**MS**	**F (DFn, DFd)**	**P value**
20	Treatment (between columns)	3296598	5	659320	F (1.242, 3.726) = 31.45	P=0.0056
21	Individual (between rows)	24530	3	8177	F (3, 15) = 0.3901	P=0.7619
22	Residual (random)	314428	15	20962		
23	Total	3635557	23			
24						
25	**Data summary**					
26	Number of treatments (columns)	6				
27	Number of subjects (rows)	4				
28	Number of missing values	0				

SourceDataforFigure5A

RM one-way ANOVA of Transpose of TNF — Multiple comparisons

	RM one-way ANOVA<br>Multiple comparisons							
1	Number of families							
2	Number of comparisons per family							
3	Alpha							
4								
5	**Tukey's multiple comparisons test**	**95.00% CI of diff.**	**Significant?**	**Summary**	**Adjusted P Value**			
6	RBCL vs. iRBCL	-17.60 to 7.347	No	ns	0.3835	A-B		
7	RBCL vs. LPS	-1797 to -117.7	Yes	*	0.0349	A-C		
8	RBCL vs. XO+RBCL	-445.5 to -55.17	Yes	*	0.0252	A-D		
9	RBCL vs. XO+iRBCL	-312.7 to -104.8	Yes	**	0.0069	A-E		
10	RBCL vs. XO+LPS	-1176 to -385.8	Yes	**	0.0073	A-F		
11	iRBCL vs. LPS	-1792 to -112.9	Yes	*	0.0354	B-C		
12	iRBCL vs. XO+RBCL	-451.7 to -38.73	Yes	*	0.0312	B-D		
13	iRBCL vs. XO+iRBCL	-307.8 to -99.46	Yes	**	0.0075	B-E		
14	iRBCL vs. XO+LPS	-1164 to -387.2	Yes	**	0.0070	B-F		
15	LPS vs. XO+RBCL	-132.1 to 1546	No	ns	0.0786	C-D		
16	LPS vs. XO+iRBCL	-34.36 to 1532	No	ns	0.0564	C-E		
17	LPS vs. XO+LPS	-999.7 to 1353	No	ns	0.9367	C-F		
18	XO+RBCL vs. XO+iRBCL	-141.5 to 224.7	No	ns	0.7805	D-E		
19	XO+RBCL vs. XO+LPS	-1081 to 19.91	No	ns	0.0552	D-F		
20	XO+iRBCL vs. XO+LPS	-1043 to -101.7	Yes	*	0.0292	E-F		
21								
22	**Test details**	**Mean 2**	**Mean Diff.**	**SE of diff.**	**n1**	**n2**	**q**	**DF**
23	RBCL vs. iRBCL	7.531	-5.125	2.195	4	4	3.303	3
24	RBCL vs. LPS	959.8	-957.4	147.7	4	4	9.164	3
25	RBCL vs. XO+RBCL	252.7	-250.3	34.34	4	4	10.31	3
26	RBCL vs. XO+iRBCL	211.2	-208.8	18.29	4	4	16.15	3
27	RBCL vs. XO+LPS	783.3	-780.9	69.52	4	4	15.89	3
28	iRBCL vs. LPS	959.8	-952.3	147.7	4	4	9.118	3
29	iRBCL vs. XO+RBCL	252.7	-245.2	36.33	4	4	9.545	3
30	iRBCL vs. XO+iRBCL	211.2	-203.6	18.33	4	4	15.71	3
31	iRBCL vs. XO+LPS	783.3	-775.8	68.37	4	4	16.05	3
32	LPS vs. XO+RBCL	252.7	707.1	147.7	4	4	6.772	3
33	[illegible]	[illegible]	[illegible]	[illegible]	[illegible]	[illegible]	[illegible]	[illegible]

SourceDataforFigure5A

RM one-way ANOVA
Multiple comparisons

16	LPS vs. XO+iRBCL	-34.36 to 1532	No	ns	0.0564	C-E		
17	LPS vs. XO+LPS	-999.7 to 1353	No	ns	0.9367	C-F		
18	XO+RBCL vs. XO+iRBCL	-141.5 to 224.7	No	ns	0.7805	D-E		
19	XO+RBCL vs. XO+LPS	-1081 to 19.91	No	ns	0.0552	D-F		
20	XO+iRBCL vs. XO+LPS	-1043 to -101.7	Yes	*	0.0292	E-F		
21								
22	**Test details**	**Mean 2**	**Mean Diff.**	**SE of diff.**	**n1**	**n2**	**q**	**DF**
23	RBCL vs. iRBCL	7.531	-5.125	2.195	4	4	3.303	3
24	RBCL vs. LPS	959.8	-957.4	147.7	4	4	9.164	3
25	RBCL vs. XO+RBCL	252.7	-250.3	34.34	4	4	10.31	3
26	RBCL vs. XO+iRBCL	211.2	-208.8	18.29	4	4	16.15	3
27	RBCL vs. XO+LPS	783.3	-780.9	69.52	4	4	15.89	3
28	iRBCL vs. LPS	959.8	-952.3	147.7	4	4	9.118	3
29	iRBCL vs. XO+RBCL	252.7	-245.2	36.33	4	4	9.545	3
30	iRBCL vs. XO+iRBCL	211.2	-203.6	18.33	4	4	15.71	3
31	iRBCL vs. XO+LPS	783.3	-775.8	68.37	4	4	16.05	3
32	LPS vs. XO+RBCL	252.7	707.1	147.7	4	4	6.772	3
33	LPS vs. XO+iRBCL	211.2	748.6	137.8	4	4	7.684	3
34	LPS vs. XO+LPS	783.3	176.5	207.0	4	4	1.206	3
35	XO+RBCL vs. XO+iRBCL	211.2	41.56	32.22	4	4	1.824	3
36	XO+RBCL vs. XO+LPS	783.3	-530.6	96.86	4	4	7.746	3
37	XO+iRBCL vs. XO+LPS	783.3	-572.1	82.78	4	4	9.774	3

RM one-way ANOVA of Transpose of TNF

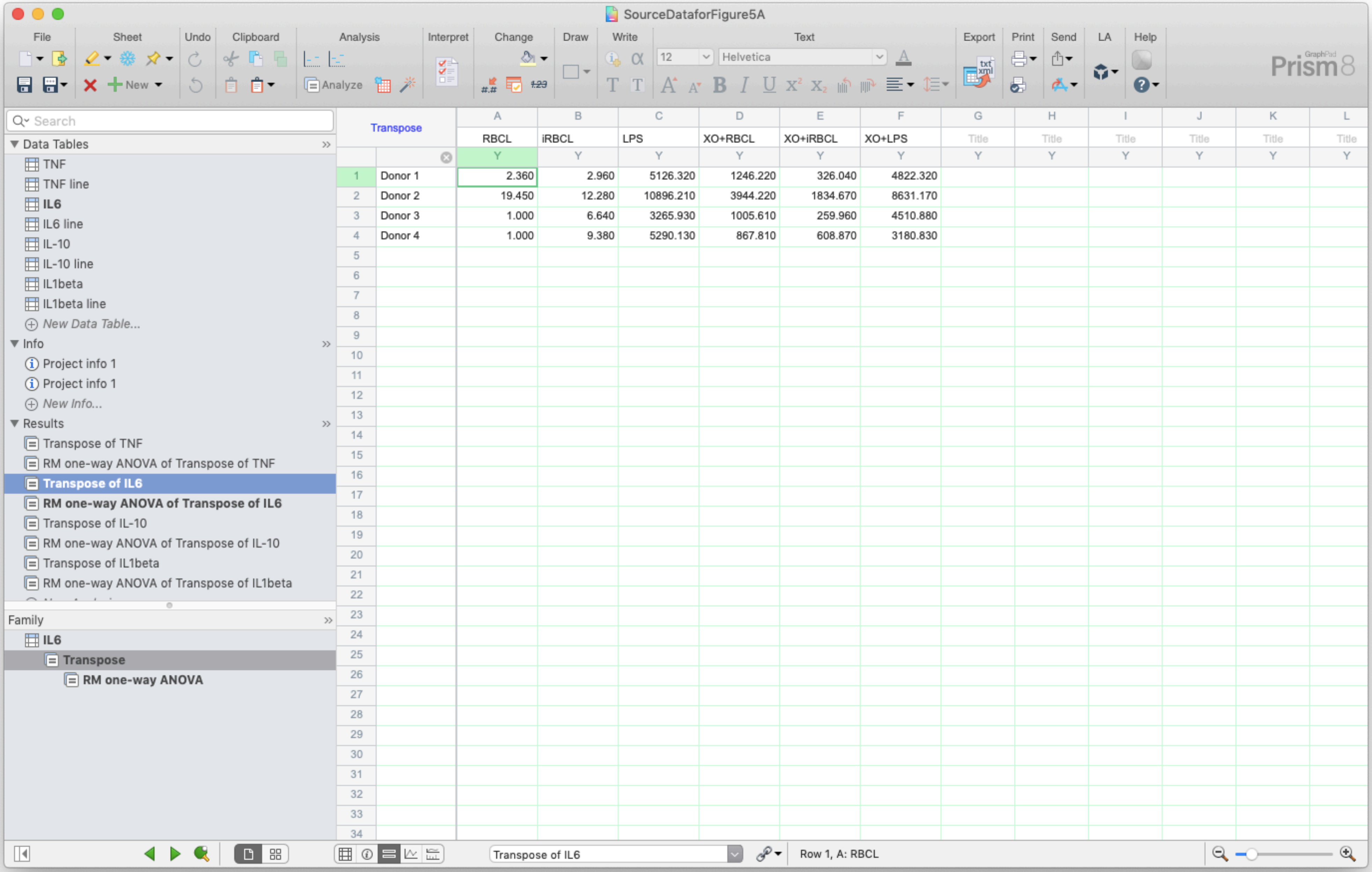

Transpose	RBCL	iRBCL	LPS	XO+RBCL	XO+iRBCL	XO+LPS
Donor 1	2.360	2.960	5126.320	1246.220	326.040	4822.320
Donor 2	19.450	12.280	10896.210	3944.220	1834.670	8631.170
Donor 3	1.000	6.640	3265.930	1005.610	259.960	4510.880
Donor 4	1.000	9.380	5290.130	867.810	608.870	3180.830

SourceDataforFigure5A

Data Tables
- TNF
- TNF line
- **IL6**
- IL6 line
- IL-10
- IL-10 line
- IL1beta
- IL1beta line
- *New Data Table...*

Info
- Project info 1
- Project info 1
- *New Info...*

Results
- Transpose of TNF
- RM one-way ANOVA of Transpose of TNF
- **Transpose of IL6**
- **RM one-way ANOVA of Transpose of IL6**
- Transpose of IL-10
- RM one-way ANOVA of Transpose of IL-10
- Transpose of IL1beta
- RM one-way ANOVA of Transpose of IL1beta

Family
- **IL6**
  - **Transpose**
    - **RM one-way ANOVA**

ANOVA results | Multiple comparisons

RM one-way ANOVA
ANOVA results

1	Table Analyzed	Transpose of IL6				
2						
3	**Repeated measures ANOVA summary**					
4	Assume sphericity?	No				
5	F	15.90				
6	P value	0.0184				
7	P value summary	*				
8	Statistically significant (P < 0.05)?	Yes				
9	Geisser-Greenhouse's epsilon	0.2427				
10	R square	0.8413				
11						
12	**Was the matching effective?**					
13	F	5.254				
14	P value	0.0112				
15	P value summary	*				
16	Is there significant matching (P < 0.05)?	Yes				
17	R square	0.1430				
18						
19	**ANOVA table**	**SS**	**DF**	**MS**	**F (DFn, DFd)**	**P value**
20	Treatment (between columns)	147504859	5	29500972	F (1.213, 3.640) = 15.90	P=0.0184
21	Individual (between rows)	29250943	3	9750314	F (3, 15) = 5.254	P=0.0112
22	Residual (random)	27834455	15	1855630		
23	Total	204590257	23			
24						
25	**Data summary**					
26	Number of treatments (columns)	6				
27	Number of subjects (rows)	4				
28	Number of missing values	0				

RM one-way ANOVA of Transpose of IL6 — Row 1, Column A

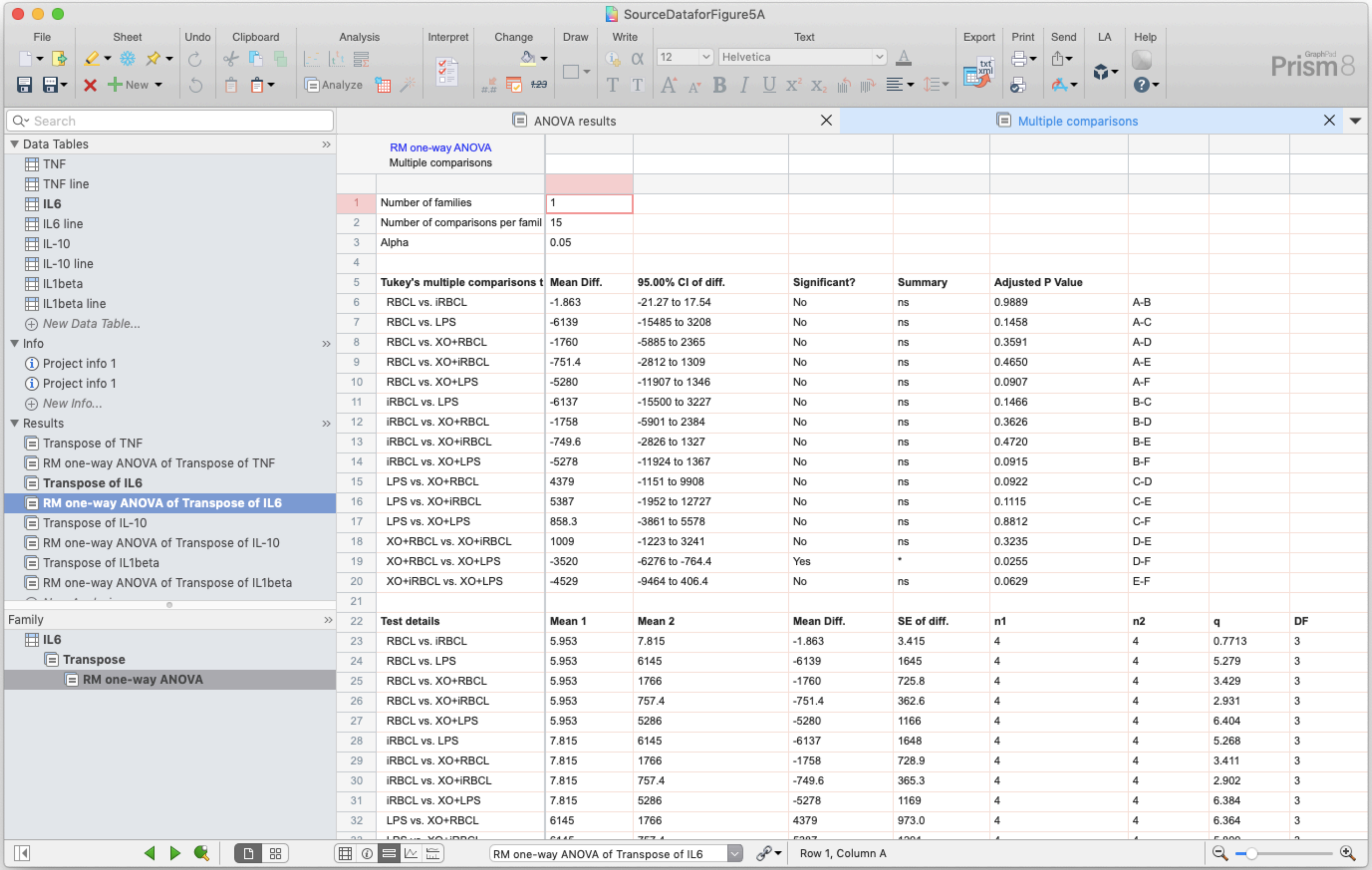

RM one-way ANOVA Multiple comparisons									
1	Number of families	1							
2	Number of comparisons per famil	15							
3	Alpha	0.05							
4									
5	Tukey's multiple comparisons t	Mean Diff.	95.00% CI of diff.	Significant?	Summary	Adjusted P Value			
6	RBCL vs. iRBCL	-1.863	-21.27 to 17.54	No	ns	0.9889	A-B		
7	RBCL vs. LPS	-6139	-15485 to 3208	No	ns	0.1458	A-C		
8	RBCL vs. XO+RBCL	-1760	-5885 to 2365	No	ns	0.3591	A-D		
9	RBCL vs. XO+iRBCL	-751.4	-2812 to 1309	No	ns	0.4650	A-E		
10	RBCL vs. XO+LPS	-5280	-11907 to 1346	No	ns	0.0907	A-F		
11	iRBCL vs. LPS	-6137	-15500 to 3227	No	ns	0.1466	B-C		
12	iRBCL vs. XO+RBCL	-1758	-5901 to 2384	No	ns	0.3626	B-D		
13	iRBCL vs. XO+iRBCL	-749.6	-2826 to 1327	No	ns	0.4720	B-E		
14	iRBCL vs. XO+LPS	-5278	-11924 to 1367	No	ns	0.0915	B-F		
15	LPS vs. XO+RBCL	4379	-1151 to 9908	No	ns	0.0922	C-D		
16	LPS vs. XO+iRBCL	5387	-1952 to 12727	No	ns	0.1115	C-E		
17	LPS vs. XO+LPS	858.3	-3861 to 5578	No	ns	0.8812	C-F		
18	XO+RBCL vs. XO+iRBCL	1009	-1223 to 3241	No	ns	0.3235	D-E		
19	XO+RBCL vs. XO+LPS	-3520	-6276 to -764.4	Yes	*	0.0255	D-F		
20	XO+iRBCL vs. XO+LPS	-4529	-9464 to 406.4	No	ns	0.0629	E-F		
21									
22	Test details	Mean 1	Mean 2	Mean Diff.	SE of diff.	n1	n2	q	DF
23	RBCL vs. iRBCL	5.953	7.815	-1.863	3.415	4	4	0.7713	3
24	RBCL vs. LPS	5.953	6145	-6139	1645	4	4	5.279	3
25	RBCL vs. XO+RBCL	5.953	1766	-1760	725.8	4	4	3.429	3
26	RBCL vs. XO+iRBCL	5.953	757.4	-751.4	362.6	4	4	2.931	3
27	RBCL vs. XO+LPS	5.953	5286	-5280	1166	4	4	6.404	3
28	iRBCL vs. LPS	7.815	6145	-6137	1648	4	4	5.268	3
29	iRBCL vs. XO+RBCL	7.815	1766	-1758	728.9	4	4	3.411	3
30	iRBCL vs. XO+iRBCL	7.815	757.4	-749.6	365.3	4	4	2.902	3
31	iRBCL vs. XO+LPS	7.815	5286	-5278	1169	4	4	6.384	3
32	LPS vs. XO+RBCL	6145	1766	4379	973.0	4	4	6.364	3
33	[illegible]	[illegible]	[illegible]	[illegible]	[illegible]	[illegible]	[illegible]	[illegible]	[illegible]

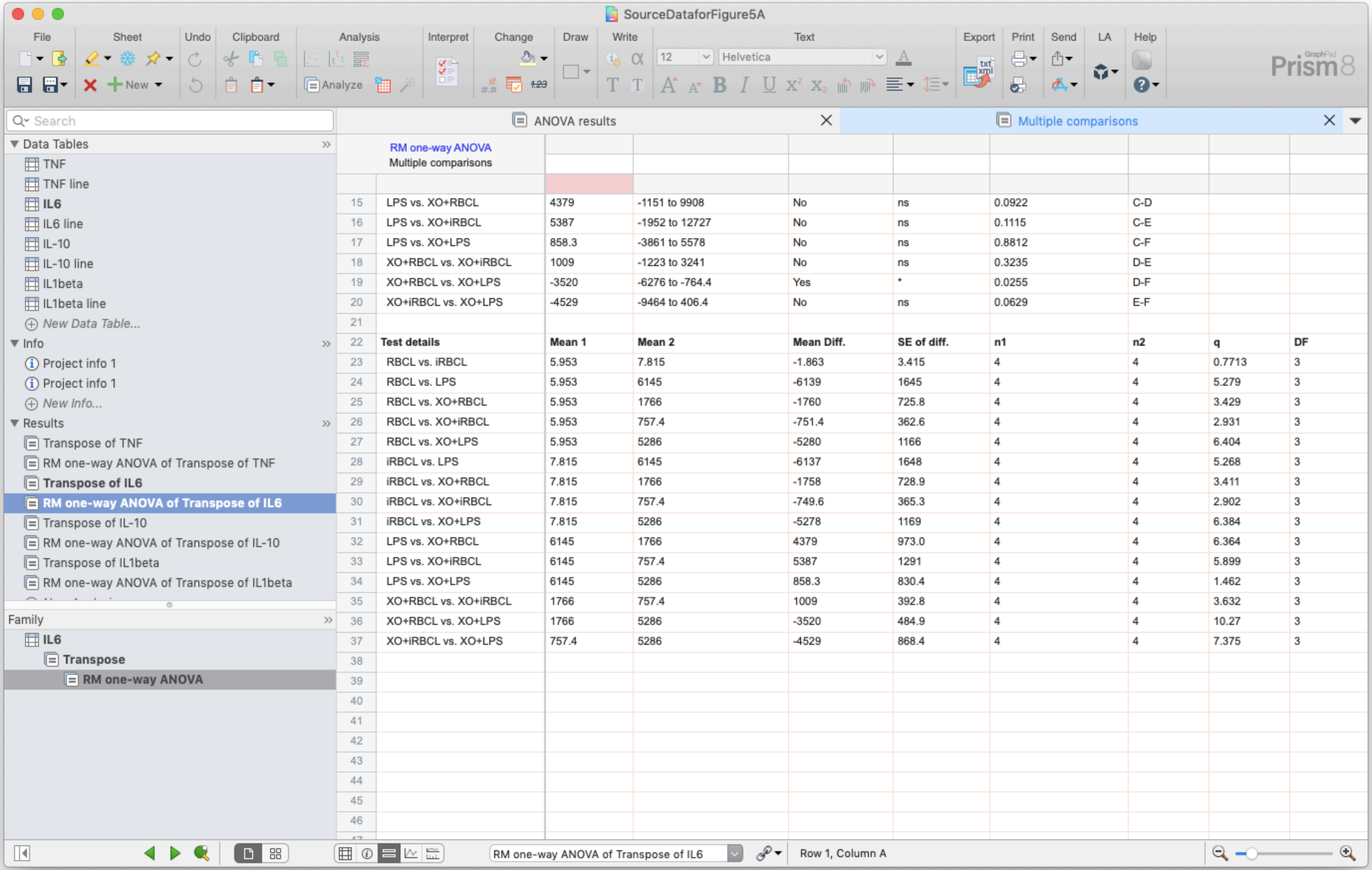

	RM one-way ANOVA<br>Multiple comparisons								
15	LPS vs. XO+RBCL	4379	-1151 to 9908	No	ns	0.0922	C-D		
16	LPS vs. XO+iRBCL	5387	-1952 to 12727	No	ns	0.1115	C-E		
17	LPS vs. XO+LPS	858.3	-3861 to 5578	No	ns	0.8812	C-F		
18	XO+RBCL vs. XO+iRBCL	1009	-1223 to 3241	No	ns	0.3235	D-E		
19	XO+RBCL vs. XO+LPS	-3520	-6276 to -764.4	Yes	*	0.0255	D-F		
20	XO+iRBCL vs. XO+LPS	-4529	-9464 to 406.4	No	ns	0.0629	E-F		
21									
22	**Test details**	**Mean 1**	**Mean 2**	**Mean Diff.**	**SE of diff.**	**n1**	**n2**	**q**	**DF**
23	RBCL vs. iRBCL	5.953	7.815	-1.863	3.415	4	4	0.7713	3
24	RBCL vs. LPS	5.953	6145	-6139	1645	4	4	5.279	3
25	RBCL vs. XO+RBCL	5.953	1766	-1760	725.8	4	4	3.429	3
26	RBCL vs. XO+iRBCL	5.953	757.4	-751.4	362.6	4	4	2.931	3
27	RBCL vs. XO+LPS	5.953	5286	-5280	1166	4	4	6.404	3
28	iRBCL vs. LPS	7.815	6145	-6137	1648	4	4	5.268	3
29	iRBCL vs. XO+RBCL	7.815	1766	-1758	728.9	4	4	3.411	3
30	iRBCL vs. XO+iRBCL	7.815	757.4	-749.6	365.3	4	4	2.902	3
31	iRBCL vs. XO+LPS	7.815	5286	-5278	1169	4	4	6.384	3
32	LPS vs. XO+RBCL	6145	1766	4379	973.0	4	4	6.364	3
33	LPS vs. XO+iRBCL	6145	757.4	5387	1291	4	4	5.899	3
34	LPS vs. XO+LPS	6145	5286	858.3	830.4	4	4	1.462	3
35	XO+RBCL vs. XO+iRBCL	1766	757.4	1009	392.8	4	4	3.632	3
36	XO+RBCL vs. XO+LPS	1766	5286	-3520	484.9	4	4	10.27	3
37	XO+iRBCL vs. XO+LPS	757.4	5286	-4529	868.4	4	4	7.375	3

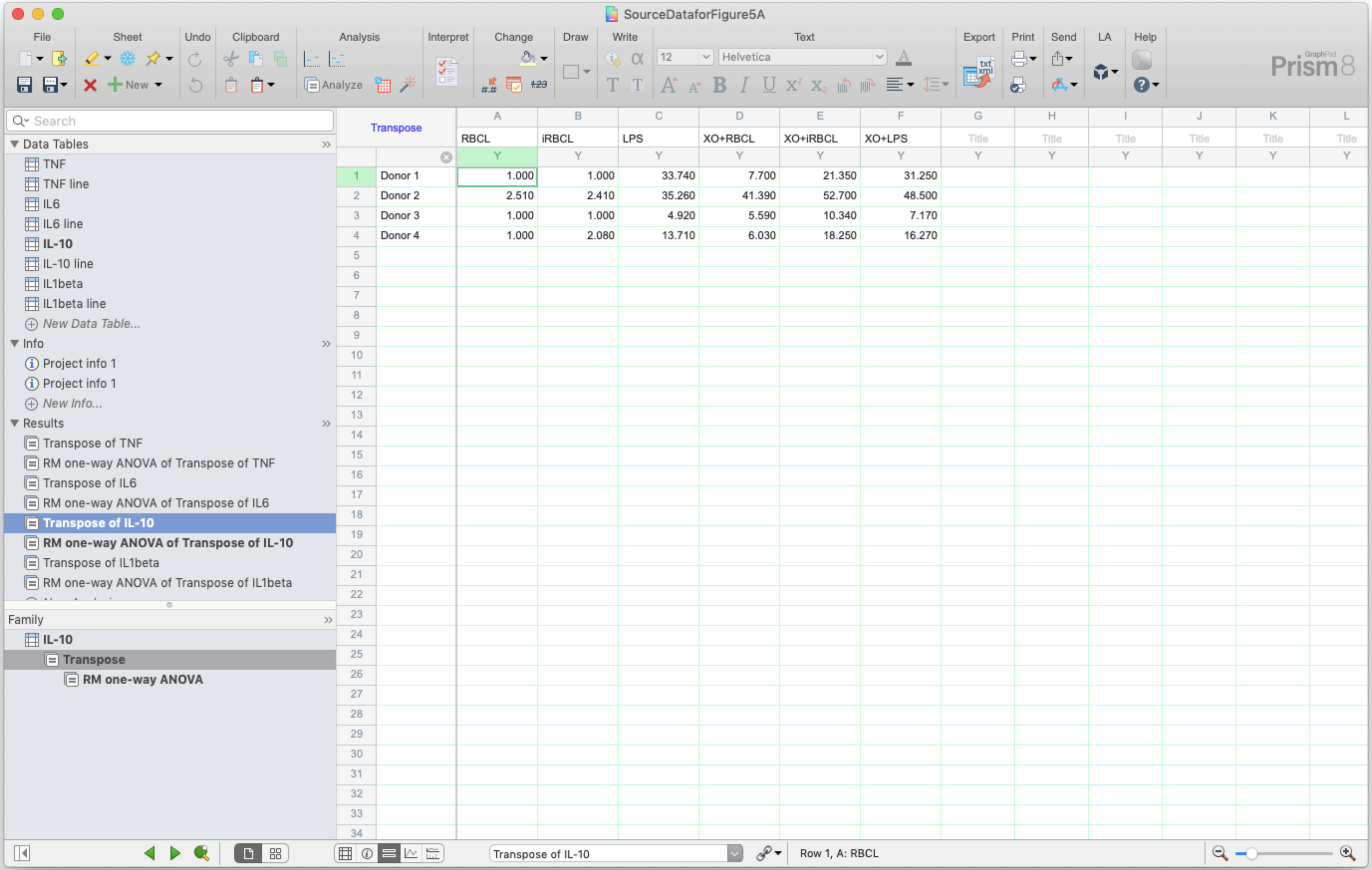

Transpose	RBCL	iRBCL	LPS	XO+RBCL	XO+iRBCL	XO+LPS
	Y	Y	Y	Y	Y	Y
Donor 1	1.000	1.000	33.740	7.700	21.350	31.250
Donor 2	2.510	2.410	35.260	41.390	52.700	48.500
Donor 3	1.000	1.000	4.920	5.590	10.340	7.170
Donor 4	1.000	2.080	13.710	6.030	18.250	16.270

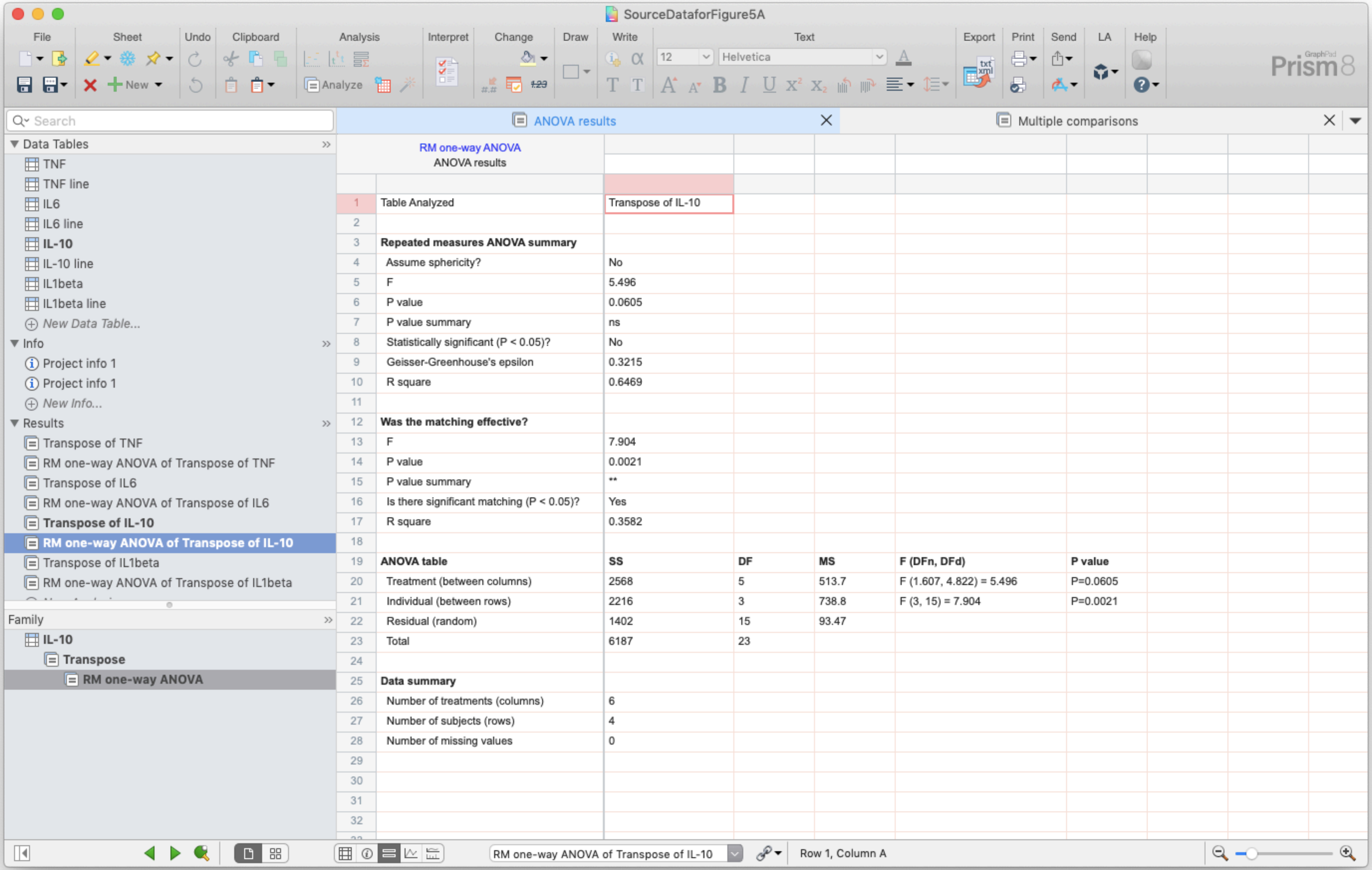

RM one-way ANOVA
ANOVA results

1	Table Analyzed	Transpose of IL-10				
2						
3	**Repeated measures ANOVA summary**					
4	Assume sphericity?	No				
5	F	5.496				
6	P value	0.0605				
7	P value summary	ns				
8	Statistically significant (P < 0.05)?	No				
9	Geisser-Greenhouse's epsilon	0.3215				
10	R square	0.6469				
11						
12	**Was the matching effective?**					
13	F	7.904				
14	P value	0.0021				
15	P value summary	**				
16	Is there significant matching (P < 0.05)?	Yes				
17	R square	0.3582				
18						
19	**ANOVA table**	**SS**	**DF**	**MS**	**F (DFn, DFd)**	**P value**
20	Treatment (between columns)	2568	5	513.7	F (1.607, 4.822) = 5.496	P=0.0605
21	Individual (between rows)	2216	3	738.8	F (3, 15) = 7.904	P=0.0021
22	Residual (random)	1402	15	93.47		
23	Total	6187	23			
24						
25	**Data summary**					
26	Number of treatments (columns)	6				
27	Number of subjects (rows)	4				
28	Number of missing values	0				

SourceDataforFigure5A

ANOVA results | Multiple comparisons

RM one-way ANOVA
Multiple comparisons

1	Number of families	1							
2	Number of comparisons per family	15							
3	Alpha	0.05							
4									
5	**Tukey's multiple comparisons test**	**Mean Diff.**	**95.00% CI of diff.**	**Significant?**	**Summary**	**Adjusted P Value**			
6	RBCL vs. iRBCL	-0.2450	-1.832 to 1.342	No	ns	0.9302	A-B		
7	RBCL vs. LPS	-20.53	-61.89 to 20.83	No	ns	0.2696	A-C		
8	RBCL vs. XO+RBCL	-13.80	-61.38 to 33.78	No	ns	0.6269	A-D		
9	RBCL vs. XO+iRBCL	-24.28	-75.10 to 26.53	No	ns	0.2907	A-E		
10	RBCL vs. XO+LPS	-24.42	-74.07 to 25.23	No	ns	0.2747	A-F		
11	iRBCL vs. LPS	-20.29	-62.30 to 21.73	No	ns	0.2849	B-C		
12	iRBCL vs. XO+RBCL	-13.56	-61.83 to 34.72	No	ns	0.6491	B-D		
13	iRBCL vs. XO+iRBCL	-24.04	-75.41 to 27.34	No	ns	0.3029	B-E		
14	iRBCL vs. XO+LPS	-24.18	-74.50 to 26.15	No	ns	0.2878	B-F		
15	LPS vs. XO+RBCL	6.730	-33.25 to 46.71	No	ns	0.9068	C-D		
16	LPS vs. XO+iRBCL	-3.753	-38.61 to 31.10	No	ns	0.9821	C-E		
17	LPS vs. XO+LPS	-3.890	-22.78 to 15.00	No	ns	0.8298	C-F		
18	XO+RBCL vs. XO+iRBCL	-10.48	-21.68 to 0.7165	No	ns	0.0597	D-E		
19	XO+RBCL vs. XO+LPS	-10.62	-37.14 to 15.90	No	ns	0.4007	D-F		
20	XO+iRBCL vs. XO+LPS	-0.1375	-18.81 to 18.53	No	ns	>0.9999	E-F		
21									
22	**Test details**	**Mean 1**	**Mean 2**	**Mean Diff.**	**SE of diff.**	**n1**	**n2**	**q**	**DF**
23	RBCL vs. iRBCL	1.378	1.623	-0.2450	0.2793	4	4	1.240	3
24	RBCL vs. LPS	1.378	21.91	-20.53	7.277	4	4	3.990	3
25	RBCL vs. XO+RBCL	1.378	15.18	-13.80	8.372	4	4	2.331	3
26	RBCL vs. XO+iRBCL	1.378	25.66	-24.28	8.941	4	4	3.841	3
27	RBCL vs. XO+LPS	1.378	25.80	-24.42	8.737	4	4	3.953	3
28	iRBCL vs. LPS	1.623	21.91	-20.29	7.392	4	4	3.881	3
29	iRBCL vs. XO+RBCL	1.623	15.18	-13.56	8.495	4	4	2.256	3
30	iRBCL vs. XO+iRBCL	1.623	25.66	-24.04	9.040	4	4	3.760	3
31	iRBCL vs. XO+LPS	1.623	25.80	-24.18	8.856	4	4	3.861	3
32	LPS vs. XO+RBCL	21.91	15.18	6.730	7.035	4	4	1.353	3
[illegible]	[illegible]	[illegible]	[illegible]	[illegible]	[illegible]	[illegible]	[illegible]	[illegible]	[illegible]

RM one-way ANOVA of Transpose of IL-10 — Row 1, Column A

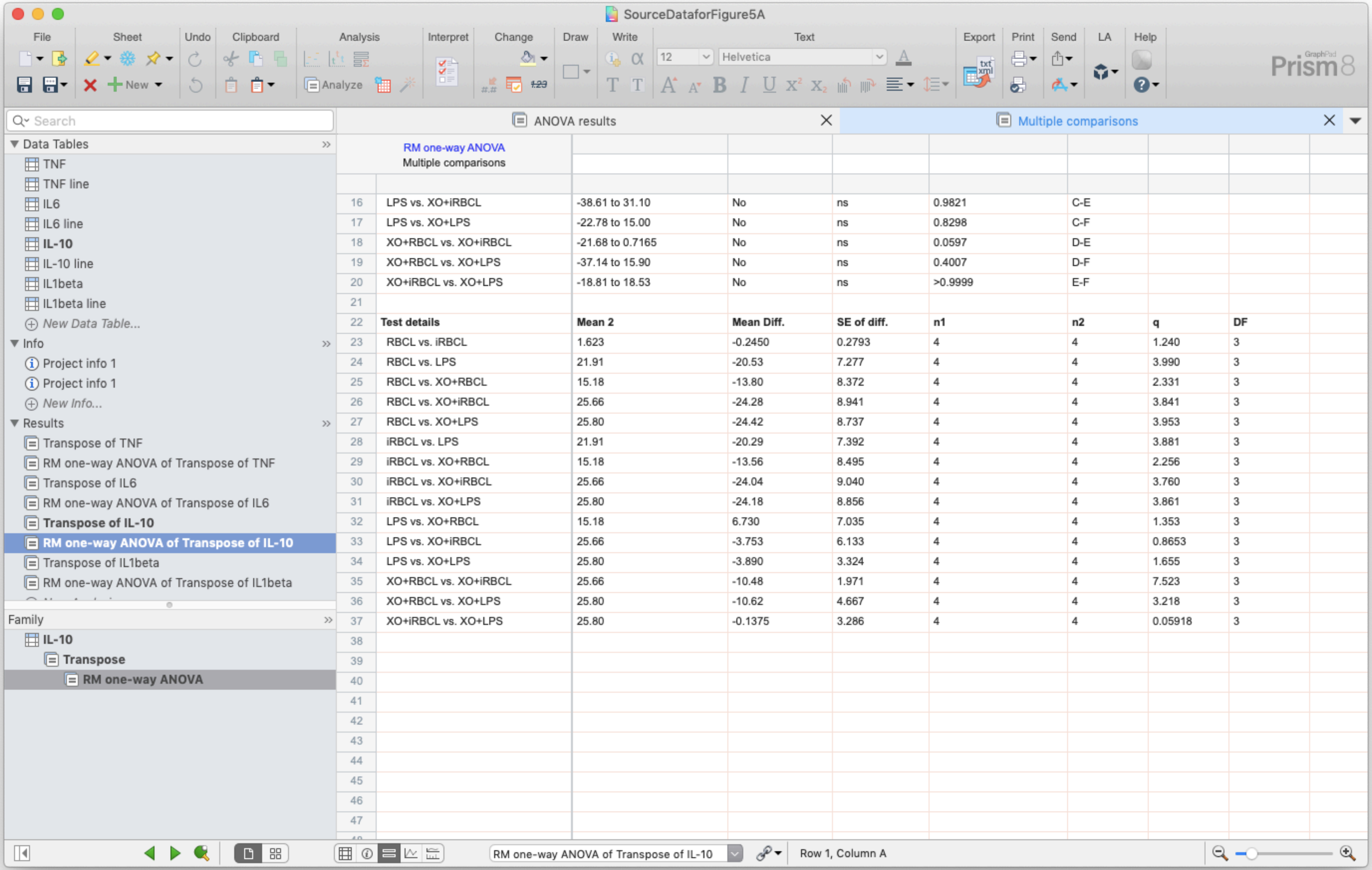

	RM one-way ANOVA Multiple comparisons							
16	LPS vs. XO+iRBCL	-38.61 to 31.10	No	ns	0.9821	C-E		
17	LPS vs. XO+LPS	-22.78 to 15.00	No	ns	0.8298	C-F		
18	XO+RBCL vs. XO+iRBCL	-21.68 to 0.7165	No	ns	0.0597	D-E		
19	XO+RBCL vs. XO+LPS	-37.14 to 15.90	No	ns	0.4007	D-F		
20	XO+iRBCL vs. XO+LPS	-18.81 to 18.53	No	ns	>0.9999	E-F		
21								
22	**Test details**	**Mean 2**	**Mean Diff.**	**SE of diff.**	**n1**	**n2**	**q**	**DF**
23	RBCL vs. iRBCL	1.623	-0.2450	0.2793	4	4	1.240	3
24	RBCL vs. LPS	21.91	-20.53	7.277	4	4	3.990	3
25	RBCL vs. XO+RBCL	15.18	-13.80	8.372	4	4	2.331	3
26	RBCL vs. XO+iRBCL	25.66	-24.28	8.941	4	4	3.841	3
27	RBCL vs. XO+LPS	25.80	-24.42	8.737	4	4	3.953	3
28	iRBCL vs. LPS	21.91	-20.29	7.392	4	4	3.881	3
29	iRBCL vs. XO+RBCL	15.18	-13.56	8.495	4	4	2.256	3
30	iRBCL vs. XO+iRBCL	25.66	-24.04	9.040	4	4	3.760	3
31	iRBCL vs. XO+LPS	25.80	-24.18	8.856	4	4	3.861	3
32	LPS vs. XO+RBCL	15.18	6.730	7.035	4	4	1.353	3
33	LPS vs. XO+iRBCL	25.66	-3.753	6.133	4	4	0.8653	3
34	LPS vs. XO+LPS	25.80	-3.890	3.324	4	4	1.655	3
35	XO+RBCL vs. XO+iRBCL	25.66	-10.48	1.971	4	4	7.523	3
36	XO+RBCL vs. XO+LPS	25.80	-10.62	4.667	4	4	3.218	3
37	XO+iRBCL vs. XO+LPS	25.80	-0.1375	3.286	4	4	0.05918	3

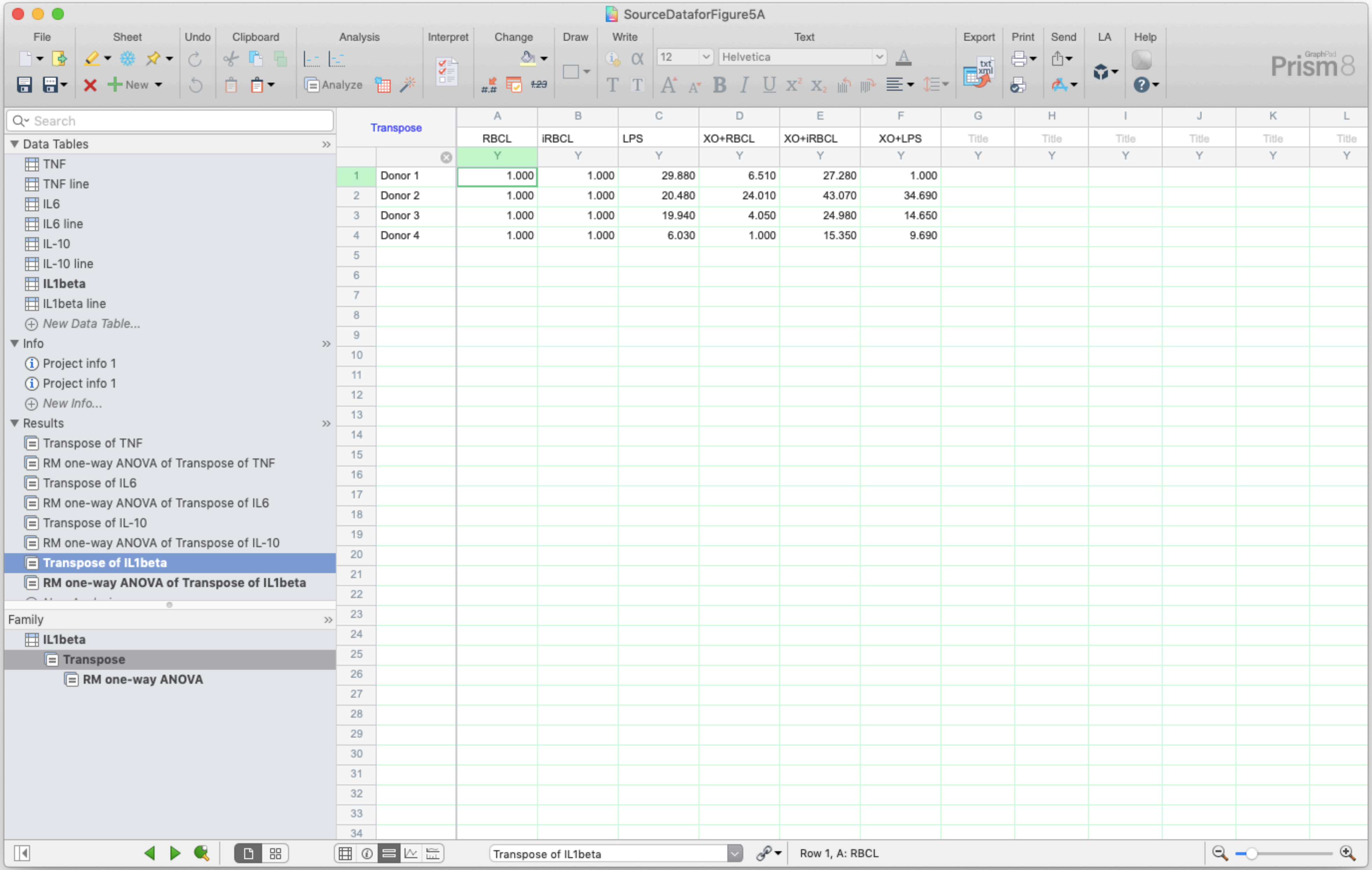

Transpose	RBCL	iRBCL	LPS	XO+RBCL	XO+iRBCL	XO+LPS
Donor 1	1.000	1.000	29.880	6.510	27.280	1.000
Donor 2	1.000	1.000	20.480	24.010	43.070	34.690
Donor 3	1.000	1.000	19.940	4.050	24.980	14.650
Donor 4	1.000	1.000	6.030	1.000	15.350	9.690

SourceDataforFigure5A

ANOVA results

RM one-way ANOVA
ANOVA results

8	Statistically significant (P < 0.05)?	Yes				
9	Geisser-Greenhouse's epsilon	0.3595				
10	R square	0.7093				
11						
12	**Was the matching effective?**					
13	F	3.871				
14	P value	0.0311				
15	P value summary	*				
16	Is there significant matching (P < 0.05)?	Yes				
17	R square	0.1837				
18						
19	**ANOVA table**	**SS**	**DF**	**MS**	**F (DFn, DFd)**	**P value**
20	Treatment (between columns)	2225	5	445.1	F (1.798, 5.393	P=0.0305
21	Individual (between rows)	706.0	3	235.3	F (3, 15) = 3.87	P=0.0311
22	Residual (random)	911.9	15	60.79		
23	Total	3843	23			
24						
25	**Data summary**					
26	Number of treatments (columns)	6				
27	Number of subjects (rows)	4				
28	Number of missing values	0				

RM one-way ANOVA of Transpose of IL1beta

SourceDataforFigure5A

Data Tables: IL6 line, IL-10, IL-10 line, IL1beta, IL1beta line, New Data Table...

Info: Project info 1, Project info 1, New Info...

Results: Transpose of TNF, RM one-way ANOVA of Transpose of TNF, Transpose of IL6, RM one-way ANOVA of Transpose of IL6, Transpose of IL-10, RM one-way ANOVA of Transpose of IL-10, Transpose of IL1beta, RM one-way ANOVA of Transpose of IL1beta, New Analysis...

Graphs: TNF, TNF line

Family: IL1beta > Transpose > RM one-way ANOVA

ANOVA results | Multiple comparisons

RM one-way ANOVA
Multiple comparisons

1	Number of families	1							
2	Number of comparisons per f	14							
3	Alpha	0.05							
4									
5	**Tukey's multiple compariso**	**Mean Diff.**	**95.00% CI of diff.**	**Significant?**	**Summary**	**Adjusted P Value**			
6	RBCL vs. LPS	-18.08	-33.72 to -2.447	Yes			A-C		
7	RBCL vs. XO+RBCL	-7.893	-24.33 to 8.540	No			A-D		
8	RBCL vs. XO+iRBCL	-26.67	-44.96 to -8.381	Yes			A-E		
9	RBCL vs. XO+LPS	-14.01	-36.73 to 8.720	No			A-F		
10	iRBCL vs. LPS	-18.08	-33.72 to -2.447	Yes			B-C		
11	iRBCL vs. XO+RBCL	-7.893	-24.33 to 8.540	No			B-D		
12	iRBCL vs. XO+iRBCL	-26.67	-44.96 to -8.381	Yes			B-E		
13	iRBCL vs. XO+LPS	-14.01	-36.73 to 8.720	No			B-F		
14	LPS vs. XO+RBCL	10.19	-8.661 to 29.04	No			C-D		
15	LPS vs. XO+iRBCL	-8.588	-25.39 to 8.211	No			C-E		
16	LPS vs. XO+LPS	4.075	-25.14 to 33.29	No			C-F		
17	XO+RBCL vs. XO+iRBCL	-18.78	-23.66 to -13.89	Yes			D-E		
18	XO+RBCL vs. XO+LPS	-6.115	-18.53 to 6.304	No			D-F		
19	XO+iRBCL vs. XO+LPS	12.66	-2.101 to 27.43	No			E-F		
20									
21	**Test details**	**Mean 1**	**Mean 2**	**Mean Diff.**	**SE of diff.**	**n1**	**n2**	**q**	**DF**
22	RBCL vs. iRBCL	1.000	1.000	0.000	0.000	4	4		
23	RBCL vs. LPS	1.000	19.08	-18.08	4.913	4	4	3.681	3
24	RBCL vs. XO+RBCL	1.000	8.893	-7.893	5.164	4	4	1.528	3
25	RBCL vs. XO+iRBCL	1.000	27.67	-26.67	5.747	4	4	4.641	3
26	RBCL vs. XO+LPS	1.000	15.01	-14.01	7.142	4	4	1.961	3
27	iRBCL vs. LPS	1.000	19.08	-18.08	4.913	4	4	3.681	3
28	iRBCL vs. XO+RBCL	1.000	8.893	-7.893	5.164	4	4	1.528	3
29	iRBCL vs. XO+iRBCL	1.000	27.67	-26.67	5.747	4	4	4.641	3
30	iRBCL vs. XO+LPS	1.000	15.01	-14.01	7.142	4	4	1.961	3
31	LPS vs. XO+RBCL	19.08	8.893	10.19	5.924	4	4	1.720	3
32	LPS vs. XO+iRBCL	19.08	27.67	-8.588	5.279	4	4	1.627	3
[illegible]	[illegible]	[illegible]	[illegible]	[illegible]	[illegible]	[illegible]	[illegible]	[illegible]	[illegible]

RM one-way ANOVA of Transpose of IL1beta — Row 1, Column A

SourceDataforFigure5A

File | Sheet | Undo | Clipboard | Analysis | Interpret | Change | Draw | Write | Text | Export | Print | Send | LA | Help

Prism 8

Data Tables
- IL6 line
- IL-10
- IL-10 line
- **IL1beta**
- IL1beta line
- *New Data Table...*

Info
- Project info 1
- Project info 1
- *New Info...*

Results
- Transpose of TNF
- RM one-way ANOVA of Transpose of TNF
- Transpose of IL6
- RM one-way ANOVA of Transpose of IL6
- Transpose of IL-10
- RM one-way ANOVA of Transpose of IL-10
- **Transpose of IL1beta**
- **RM one-way ANOVA of Transpose of IL1beta**
- *New Analysis...*

Graphs
- TNF
- TNF line

Family
- **IL1beta**
  - **Transpose**
    - **RM one-way ANOVA**

ANOVA results | Multiple comparisons

RM one-way ANOVA
Multiple comparisons

18	XO+RBCL vs. XO+LPS	-6.115	-18.53 to 6.304	No			D-F		
19	XO+iRBCL vs. XO+LPS	12.66	-2.101 to 27.43	No			E-F		
20									
21	**Test details**	**Mean 1**	**Mean 2**	**Mean Diff.**	**SE of diff.**	**n1**	**n2**	**q**	**DF**
22	RBCL vs. iRBCL	1.000	1.000	0.000	0.000	4	4		
23	RBCL vs. LPS	1.000	19.08	-18.08	4.913	4	4	3.681	3
24	RBCL vs. XO+RBCL	1.000	8.893	-7.893	5.164	4	4	1.528	3
25	RBCL vs. XO+iRBCL	1.000	27.67	-26.67	5.747	4	4	4.641	3
26	RBCL vs. XO+LPS	1.000	15.01	-14.01	7.142	4	4	1.961	3
27	iRBCL vs. LPS	1.000	19.08	-18.08	4.913	4	4	3.681	3
28	iRBCL vs. XO+RBCL	1.000	8.893	-7.893	5.164	4	4	1.528	3
29	iRBCL vs. XO+iRBCL	1.000	27.67	-26.67	5.747	4	4	4.641	3
30	iRBCL vs. XO+LPS	1.000	15.01	-14.01	7.142	4	4	1.961	3
31	LPS vs. XO+RBCL	19.08	8.893	10.19	5.924	4	4	1.720	3
32	LPS vs. XO+iRBCL	19.08	27.67	-8.588	5.279	4	4	1.627	3
33	LPS vs. XO+LPS	19.08	15.01	4.075	9.178	4	4	0.4440	3
34	XO+RBCL vs. XO+iRBCL	8.893	27.67	-18.78	1.535	4	4	12.23	3
35	XO+RBCL vs. XO+LPS	8.893	15.01	-6.115	3.902	4	4	1.567	3
36	XO+iRBCL vs. XO+LPS	27.67	15.01	12.66	4.639	4	4	2.730	3

RM one-way ANOVA of Transpose of IL1beta | Row 1, Column A

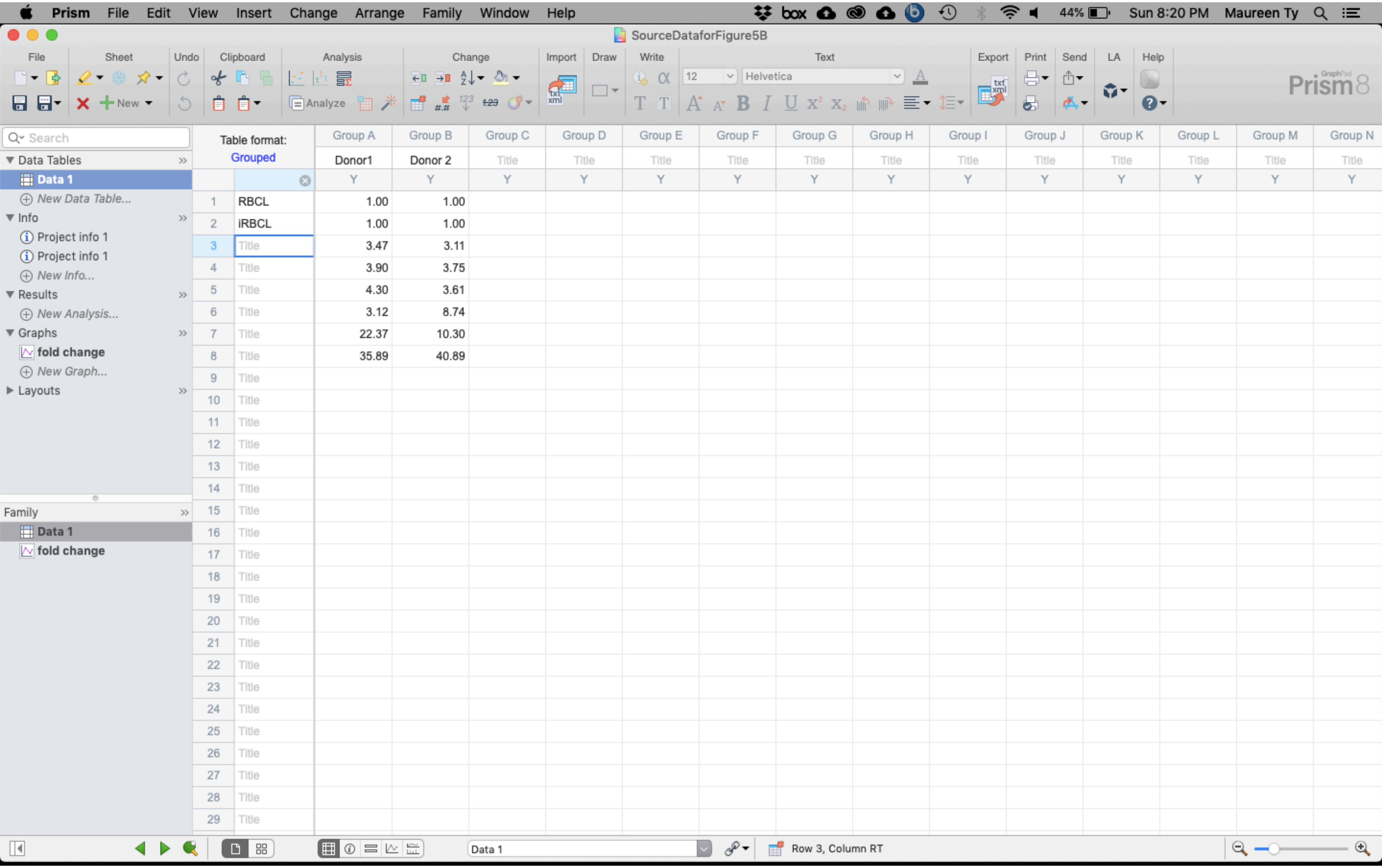
Prism File Edit View Insert Change Arrange Family Window Help
Sun 8:20 PM Maureen Ty
SourceDataforFigure5B
Data Tables
Data 1
New Data Table...
Info
Project info 1
Project info 1
New Info...
Results
New Analysis...
Graphs
fold change
New Graph...
Layouts
Family
Data 1
fold change
Table format: Grouped
Group A Donor1
Group B Donor 2
1 RBCL 1.00 1.00
2 iRBCL 1.00 1.00
3 3.47 3.11
4 3.90 3.75
5 4.30 3.61
6 3.12 8.74
7 22.37 10.30
8 35.89 40.89
Data 1
Row 3, Column RT

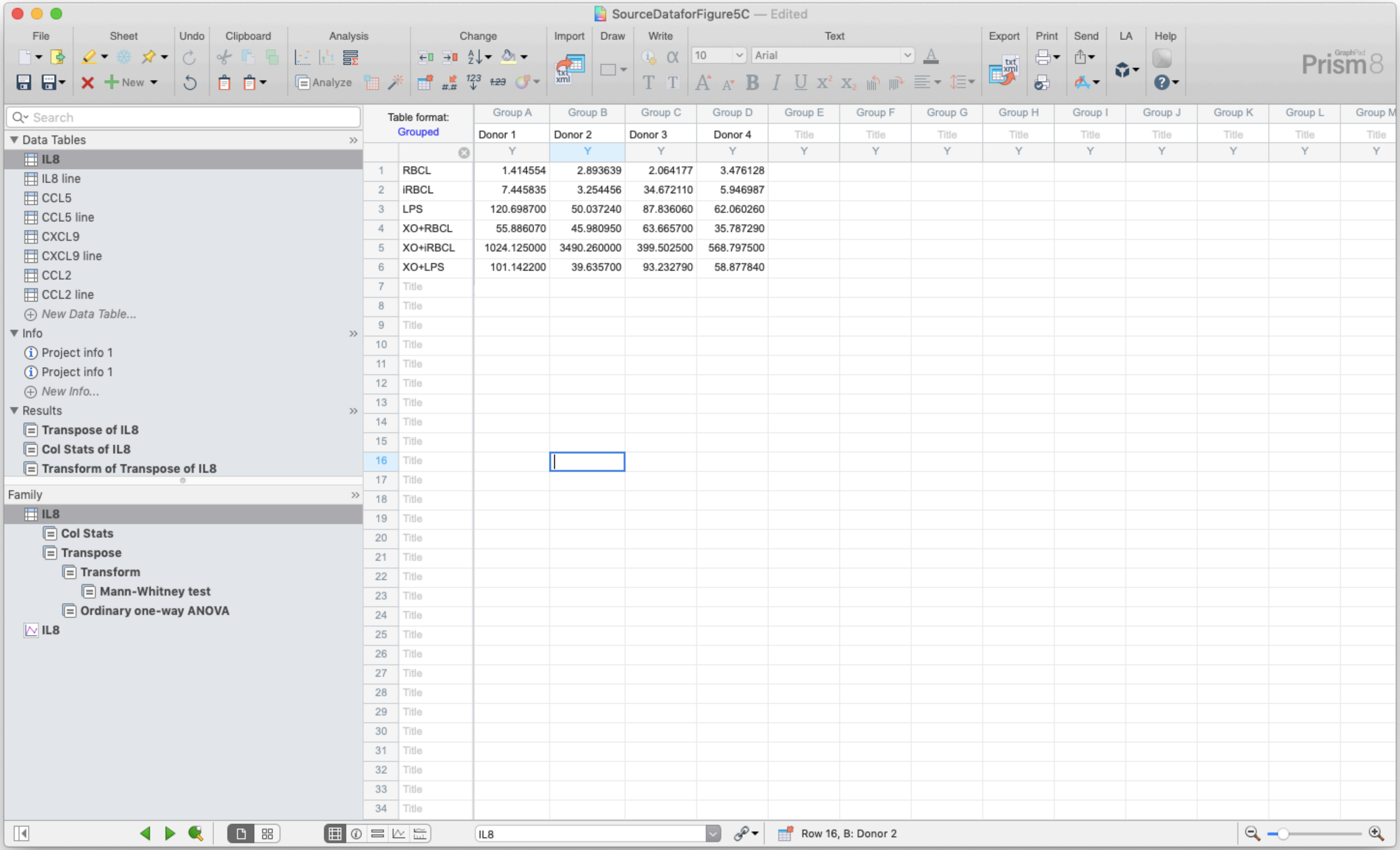

		Group A	Group B	Group C	Group D
		Donor 1	Donor 2	Donor 3	Donor 4
		Y	Y	Y	Y
1	RBCL	1.414554	2.893639	2.064177	3.476128
2	iRBCL	7.445835	3.254456	34.672110	5.946987
3	LPS	120.698700	50.037240	87.836060	62.060260
4	XO+RBCL	55.886070	45.980950	63.665700	35.787290
5	XO+iRBCL	1024.125000	3490.260000	399.502500	568.797500
6	XO+LPS	101.142200	39.635700	93.232790	58.877840

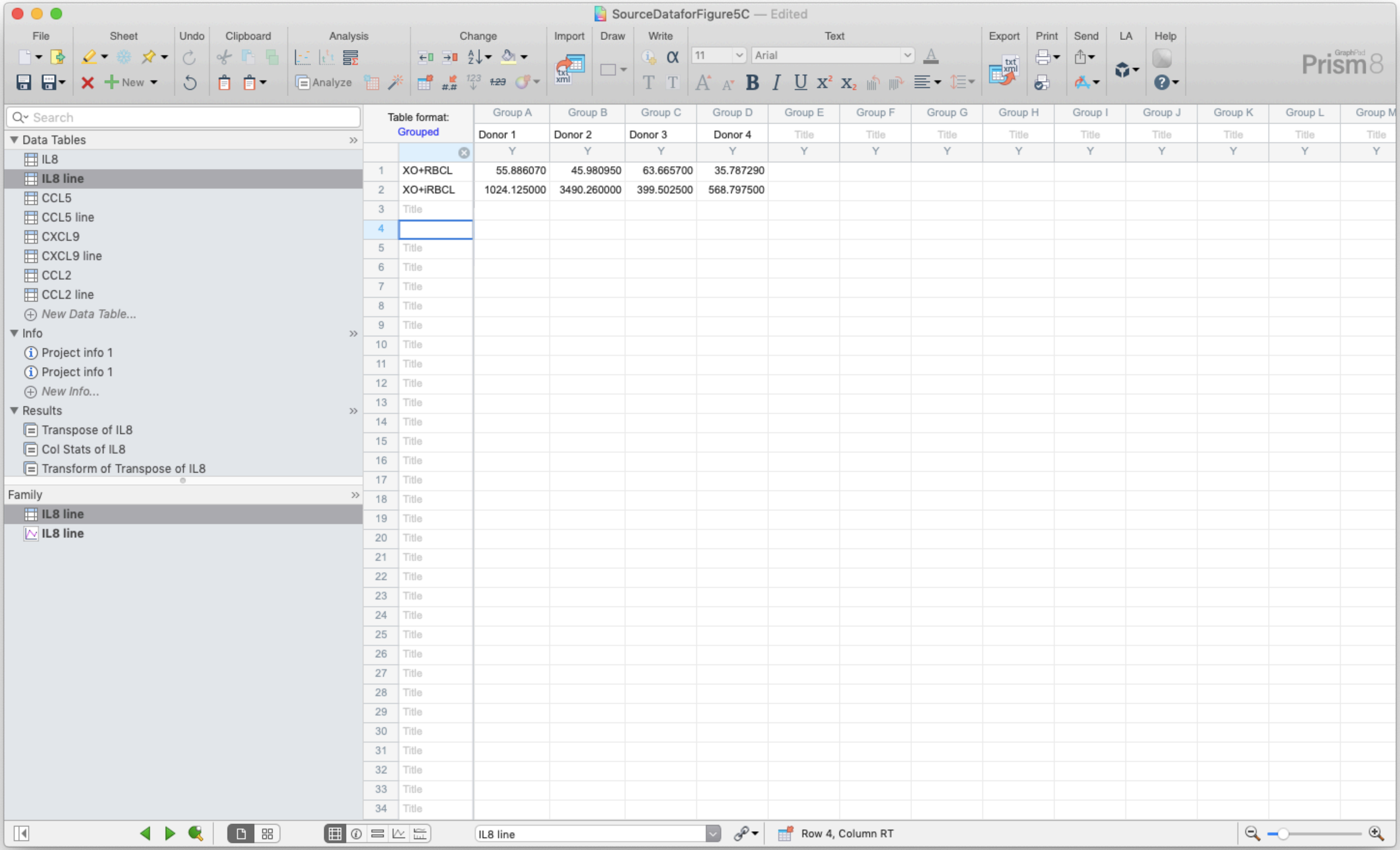
SourceDataforFigure5C — Edited
File
Sheet
Undo
Clipboard
Analysis
Change
Import
Draw
Write
Text
Export
Print
Send
LA
Help
Prism 8
Search
Data Tables
IL8
IL8 line
CCL5
CCL5 line
CXCL9
CXCL9 line
CCL2
CCL2 line
New Data Table...
Info
Project info 1
Project info 1
New Info...
Results
Transpose of IL8
Col Stats of IL8
Transform of Transpose of IL8
Family
IL8 line
IL8 line
Table format:
Grouped
Group A
Group B
Group C
Group D
Donor 1
Donor 2
Donor 3
Donor 4
XO+RBCL
55.886070
45.980950
63.665700
35.787290
XO+iRBCL
1024.125000
3490.260000
399.502500
568.797500
IL8 line
Row 4, Column RT

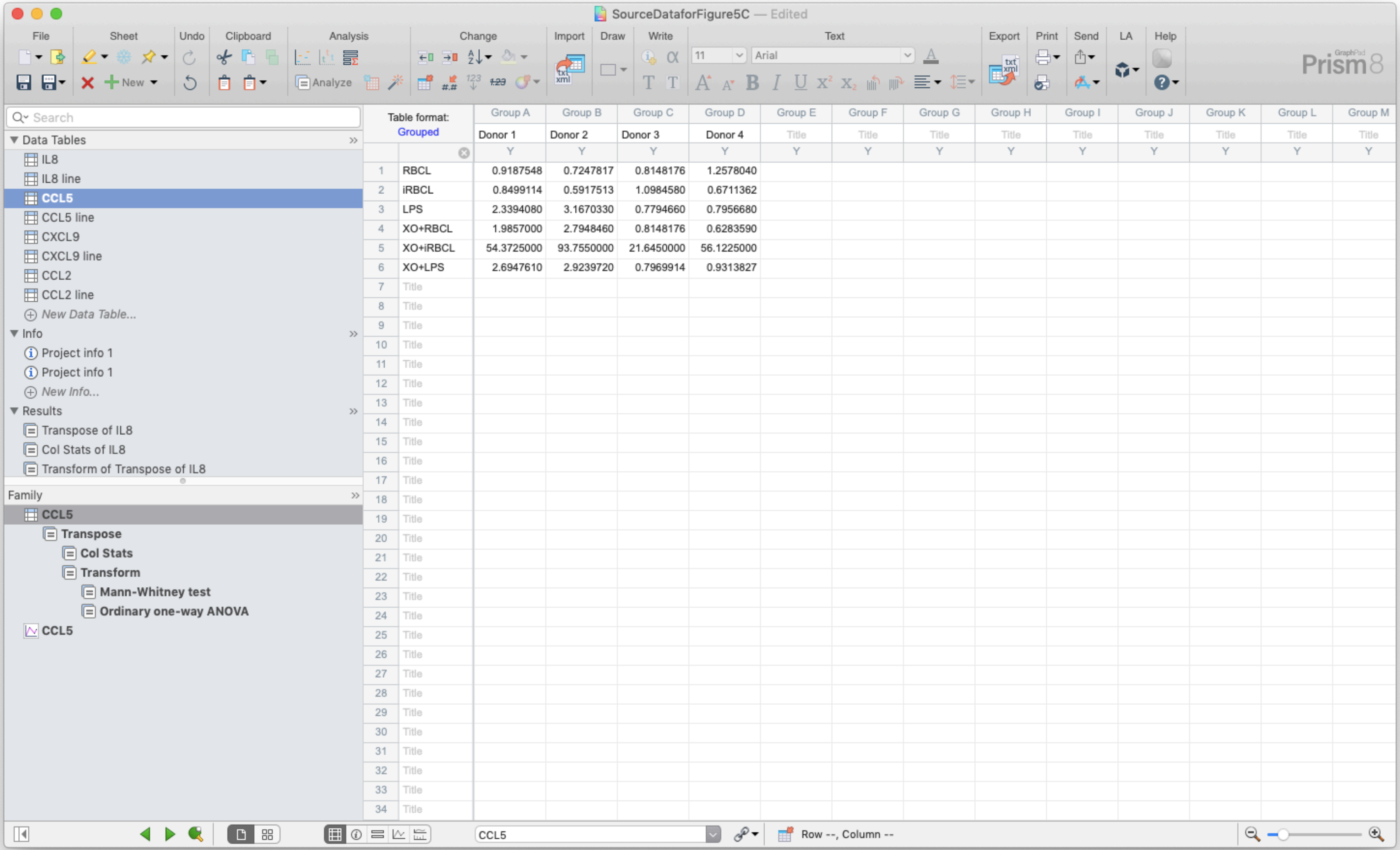

SourceDataforFigure5C — Edited

Table format: Grouped

	Group A	Group B	Group C	Group D
	Donor 1	Donor 2	Donor 3	Donor 4
RBCL	0.9187548	0.7247817	0.8148176	1.2578040
iRBCL	0.8499114	0.5917513	1.0984580	0.6711362
LPS	2.3394080	3.1670330	0.7794660	0.7956680
XO+RBCL	1.9857000	2.7948460	0.8148176	0.6283590
XO+iRBCL	54.3725000	93.7550000	21.6450000	56.1225000
XO+LPS	2.6947610	2.9239720	0.7969914	0.9313827

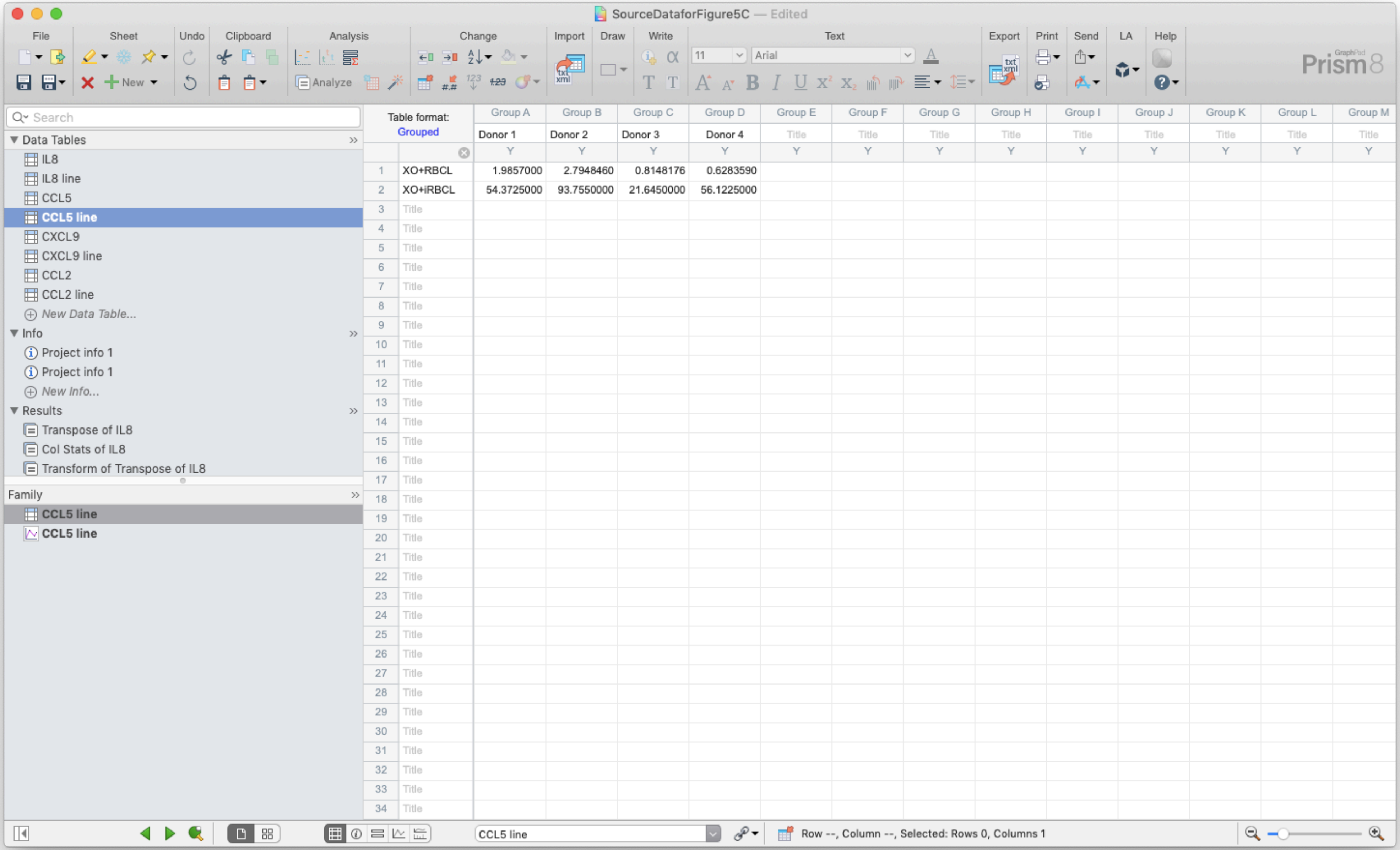
SourceDataforFigure5C — Edited
File Sheet Undo Clipboard Analysis Change Import Draw Write Text Export Print Send LA Help
Prism 8
Data Tables
IL8
IL8 line
CCL5
CCL5 line
CXCL9
CXCL9 line
CCL2
CCL2 line
New Data Table...
Info
Project info 1
Project info 1
New Info...
Results
Transpose of IL8
Col Stats of IL8
Transform of Transpose of IL8
Family
CCL5 line
CCL5 line
Table format: Grouped
Group A Group B Group C Group D
Donor 1 Donor 2 Donor 3 Donor 4
1 XO+RBCL 1.9857000 2.7948460 0.8148176 0.6283590
2 XO+iRBCL 54.3725000 93.7550000 21.6450000 56.1225000
CCL5 line
Row --, Column --, Selected: Rows 0, Columns 1

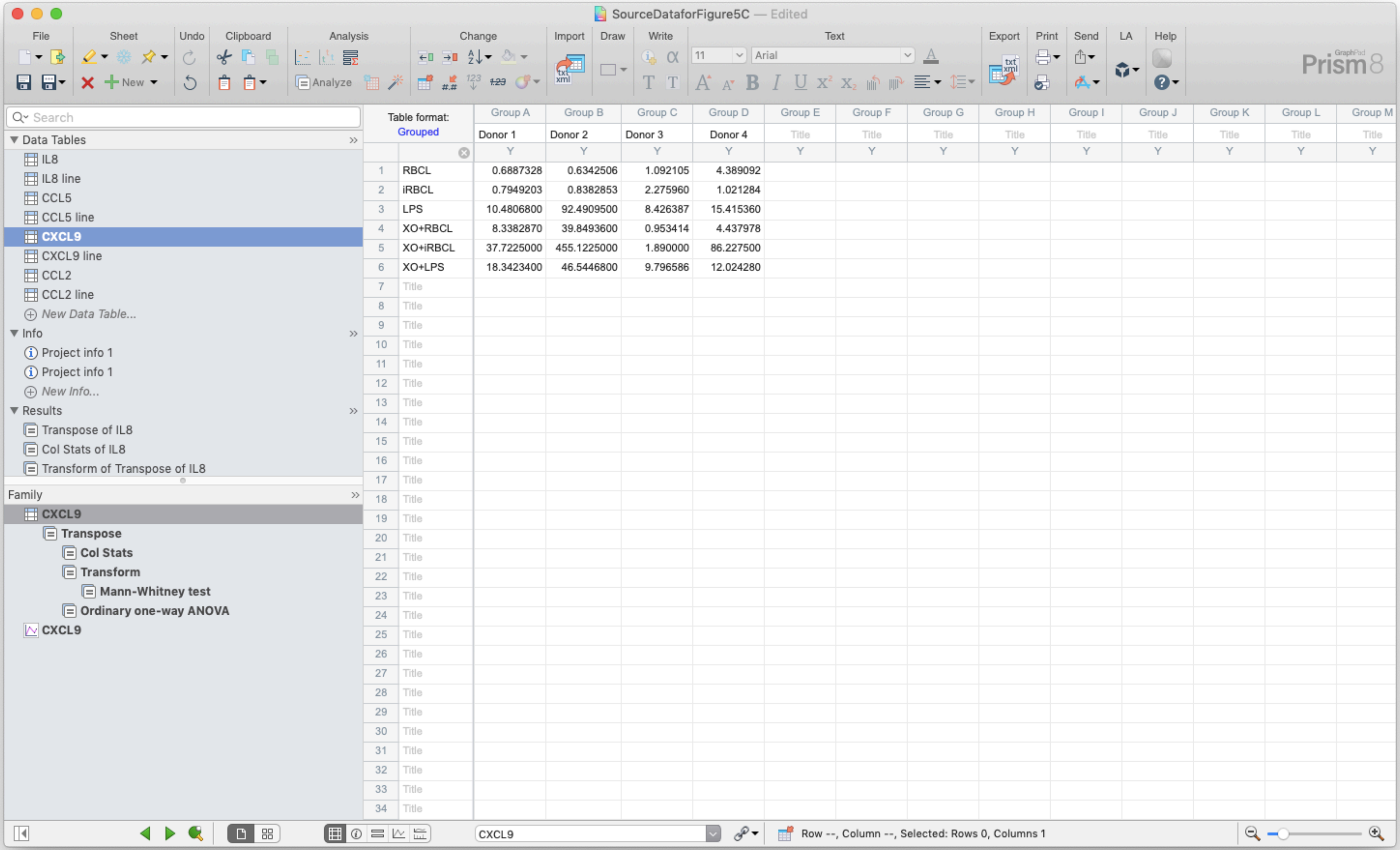

	Table format: Grouped	Group A	Group B	Group C	Group D
		Donor 1	Donor 2	Donor 3	Donor 4
		Y	Y	Y	Y
1	RBCL	0.6887328	0.6342506	1.092105	4.389092
2	iRBCL	0.7949203	0.8382853	2.275960	1.021284
3	LPS	10.4806800	92.4909500	8.426387	15.415360
4	XO+RBCL	8.3382870	39.8493600	0.953414	4.437978
5	XO+iRBCL	37.7225000	455.1225000	1.890000	86.227500
6	XO+LPS	18.3423400	46.5446800	9.796586	12.024280

SourceDataforFigure5C — Edited

Table format: Grouped

CXCL9 line

	Group A	Group B	Group C	Group D
	Donor 1	Donor 2	Donor 3	Donor 4
	Y	Y	Y	Y
1 XO+RBCL	8.3382870	39.8493600	0.953414	4.437978
2 XO+iRBCL	37.7225000	455.1225000	1.890000	86.227500

Data Tables: IL8, IL8 line, CCL5, CCL5 line, CXCL9, CXCL9 line, CCL2, CCL2 line

Info: Project info 1, Project info 1

Results: Transpose of IL8, Col Stats of IL8, Transform of Transpose of IL8

SourceDataforFigure5C — Edited

Data Tables
- IL8
- IL8 line
- CCL5
- CCL5 line
- CXCL9
- CXCL9 line
- CCL2
- CCL2 line
- New Data Table...

Info
- Project info 1
- Project info 1
- New Info...

Results
- Transpose of IL8
- Col Stats of IL8
- Transform of Transpose of IL8

Family
- CCL2
  - Transpose
    - Col Stats
    - Transform
      - Mann-Whitney test
      - Ordinary one-way ANOVA
- CCL2

Table format: Grouped

	Group A	Group B	Group C	Group D
	Donor 1	Donor 2	Donor 3	Donor 4
	Y	Y	Y	Y
RBCL	1.078116	3.304261	0.9106138	2.403658
iRBCL	3.002600	7.001407	4.2781820	2.323692
LPS	3.920284	7.819363	1.5309630	3.688431
XO+RBCL	5.864973	14.456020	3.4418510	4.516589
XO+iRBCL	230.157500	564.680000	59.6150000	106.227500
XO+LPS	5.472895	7.819363	1.9949200	3.275627

CCL2

Row --, Column --, Selected: Rows 0, Columns 1

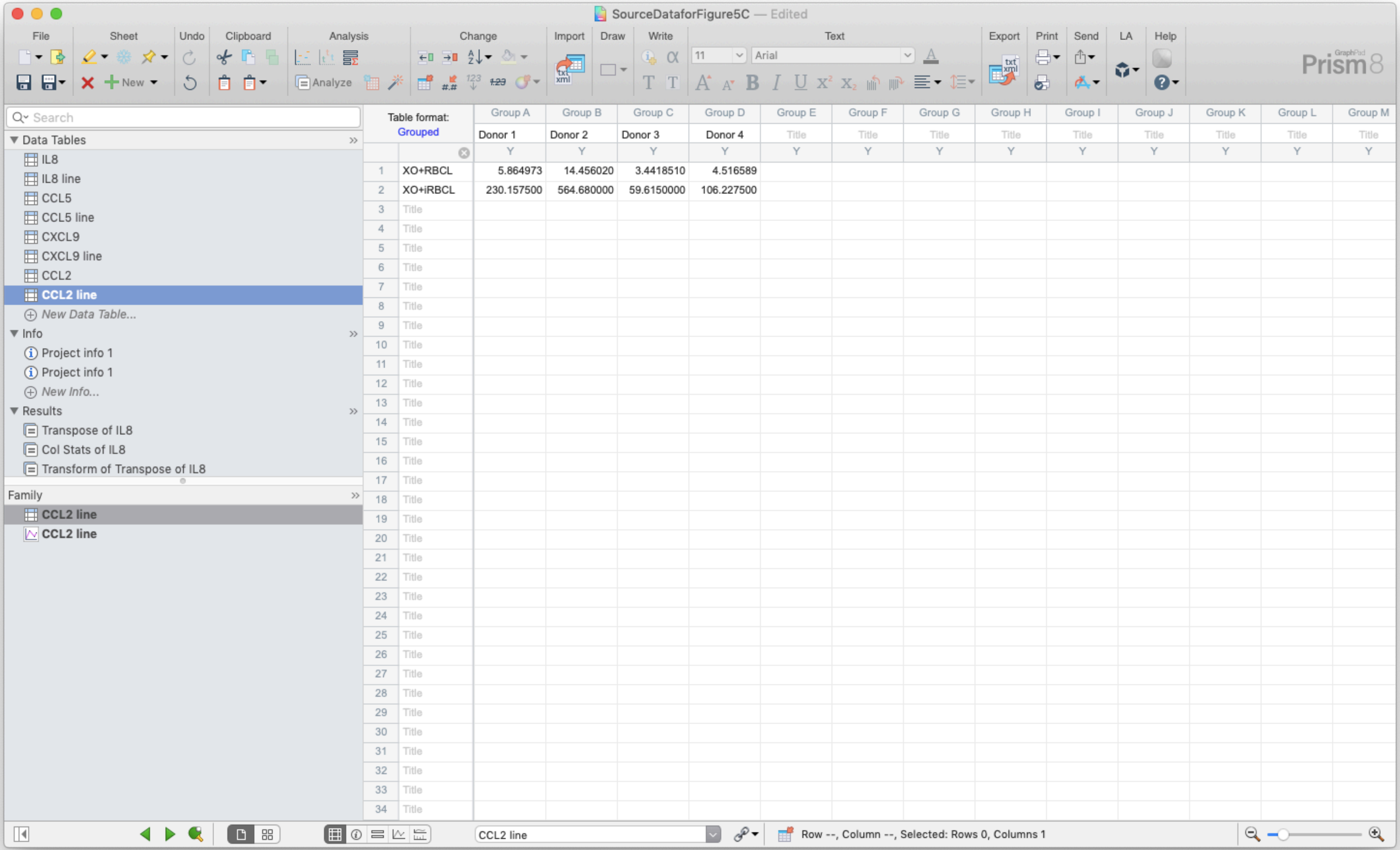
SourceDataforFigure5C — Edited
File Sheet Undo Clipboard Analysis Change Import Draw Write Text Export Print Send LA Help
Prism 8
Data Tables
IL8
IL8 line
CCL5
CCL5 line
CXCL9
CXCL9 line
CCL2
CCL2 line
New Data Table...
Info
Project info 1
Project info 1
New Info...
Results
Transpose of IL8
Col Stats of IL8
Transform of Transpose of IL8
Family
CCL2 line
CCL2 line
Table format: Grouped
Group A Group B Group C Group D
Donor 1 Donor 2 Donor 3 Donor 4
1 XO+RBCL 5.864973 14.456020 3.4418510 4.516589
2 XO+iRBCL 230.157500 564.680000 59.6150000 106.227500
Row --, Column --, Selected: Rows 0, Columns 1

SourceDataforFigure5C — Edited

Info
- Project info 1
- Project info 1
- *New Info...*

Results
- **Transpose of IL8**
- Col Stats of IL8
- **Transform of Transpose of IL8**
- **Mann-Whitney test of Transform of Transpose of IL8**
- **Ordinary one-way ANOVA of Transpose of IL8**
- Transpose of CCL5
- Col Stats of Transpose of CCL5
- Transform of Transpose of CCL5
- Mann-Whitney test of Transform of Transpose of CCL5
- Ordinary one-way ANOVA of Transform of Transpose of CC
- Transpose of CXCL9
- Col Stats of Transpose of CXCL9
- Transform of Transpose of CXCL9

Family
- **IL8**
  - **Transpose**
    - **Transform**
      - **Mann-Whitney test**
    - **Ordinary one-way ANOVA**

	Transpose	A RBCL (Y)	B iRBCL (Y)	C LPS (Y)	D XO+RBCL (Y)	E XO+iRBCL (Y)	F XO+LPS (Y)
1	Donor 1	1.415	7.446	120.699	55.886	1024.125	101.142
2	Donor 2	2.894	3.254	50.037	45.981	3490.260	39.636
3	Donor 3	2.064	34.672	87.836	63.666	399.503	93.233
4	Donor 4	3.476	5.947	62.060	35.787	568.798	58.878

Transpose of IL8

Row 1, A: RBCL

SourceDataforFigure5C — Edited

Info
- Project info 1
- Project info 1
- New Info...

Results
- Transpose of IL8
- **Col Stats of IL8**
- Transform of Transpose of IL8
- Mann-Whitney test of Transform of Transpose of IL8
- Ordinary one-way ANOVA of Transpose of IL8
- Transpose of CCL5
- Col Stats of Transpose of CCL5
- Transform of Transpose of CCL5
- Mann-Whitney test of Transform of Transpose of CCL5
- Ordinary one-way ANOVA of Transform of Transpose of CC
- Transpose of CXCL9
- Col Stats of Transpose of CXCL9
- Transform of Transpose of CXCL9

Family
- **IL8**
  - **Col Stats**

	Col Stats	A Donor 1	B Donor 2	C Donor 3	D Donor 4
		Y	Y	Y	Y
1	Number of values	6	6	6	6
2					
3	Minimum	1.415	2.894	2.064	3.476
4	25% Percentile	5.938	3.164	26.52	5.329
5	Median	78.51	42.81	75.75	47.33
6	75% Percentile	346.6	910.1	169.8	188.7
7	Maximum	1024	3490	399.5	568.8
8					
9	Mean	218.5	605.3	113.5	122.5
10	Std. Deviation	397.6	1413	144.2	220.1
11	Std. Error of Mean	162.3	577.0	58.87	89.84
12					
13	Lower 95% CI of mean	-198.8	-878.0	-37.84	-108.5
14	Upper 95% CI of mean	635.7	2089	264.8	353.4
15					
16	Sum	1311	3632	681.0	734.9
17					
18	**D'Agostino & Pearson normality test**				
19	K2	N too small	N too small	N too small	N too small
20	P value				
21	Passed normality test (alpha=0.05)?				
22	P value summary				
23					
24	**Shapiro-Wilk normality test**				
25	W	0.6082	0.5095	0.7226	0.6003
26	P value	0.0006	<0.0001	0.0106	0.0005
27	Passed normality test (alpha=0.05)?	No	No	No	No
28	P value summary	***	****	*	***
29					
30	**KS normality test**				
31	KS distance	0.4304	0.4861	0.3892	0.4415
32	P value	0.0009	<0.0001	0.0049	0.0005
33	Passed normality test (alpha=0.05)?	No	No	No	No
34	P value summary	***	****	**	***

Col Stats of IL8 — Row 1, A: Donor 1

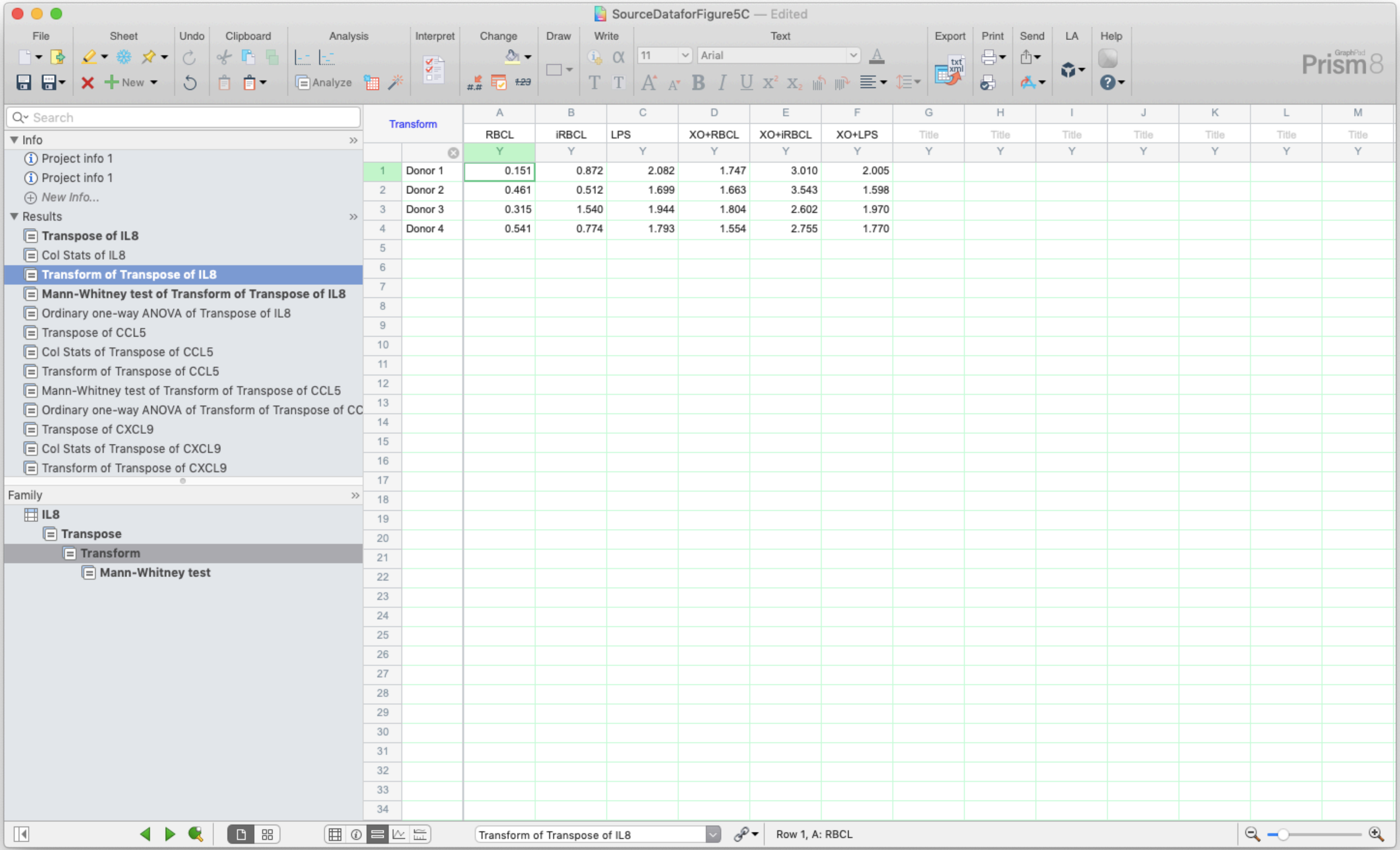

Transform	RBCL	iRBCL	LPS	XO+RBCL	XO+iRBCL	XO+LPS
Donor 1	0.151	0.872	2.082	1.747	3.010	2.005
Donor 2	0.461	0.512	1.699	1.663	3.543	1.598
Donor 3	0.315	1.540	1.944	1.804	2.602	1.970
Donor 4	0.541	0.774	1.793	1.554	2.755	1.770

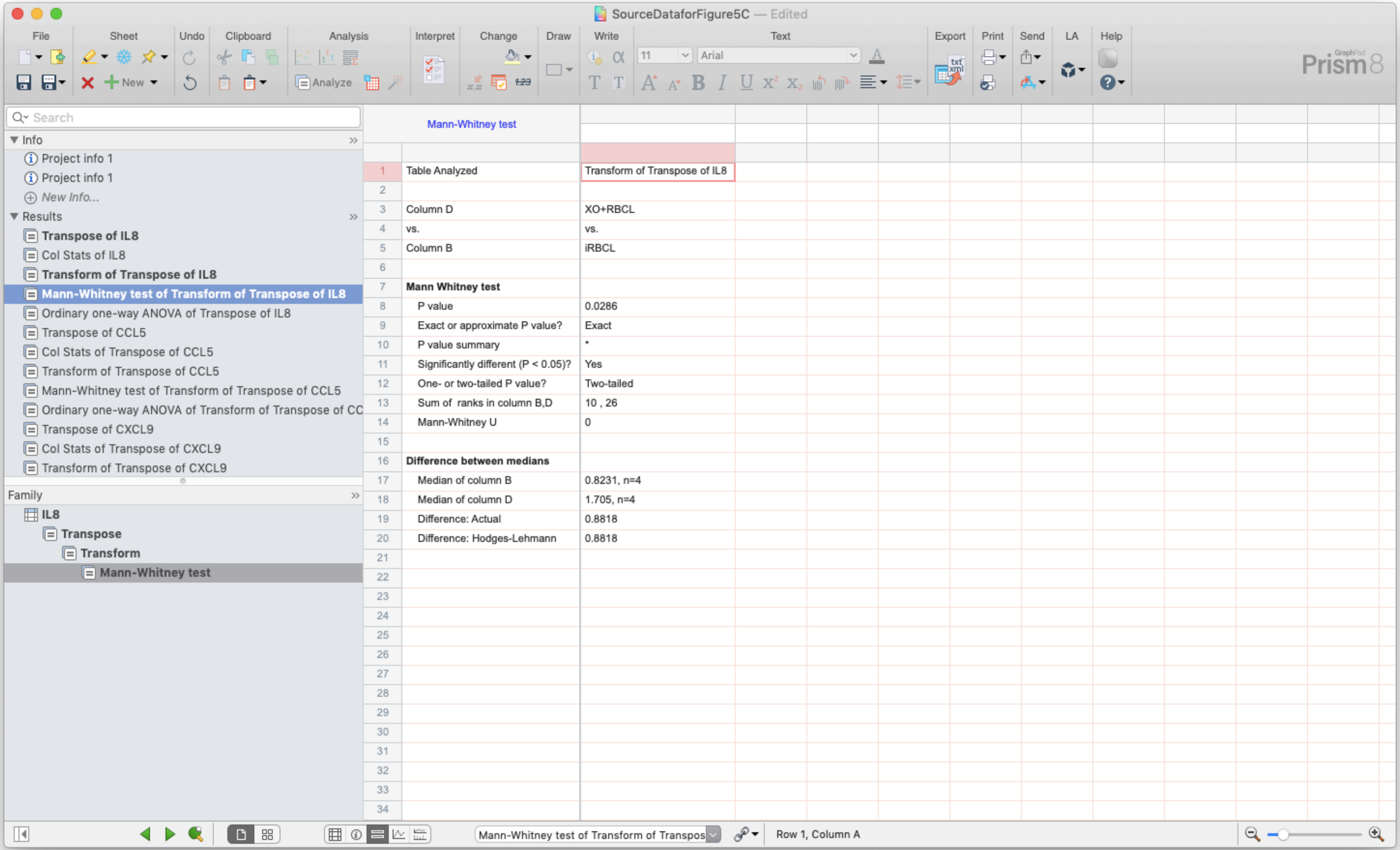

	Mann-Whitney test	
1	Table Analyzed	Transform of Transpose of IL8
2		
3	Column D	XO+RBCL
4	vs.	vs.
5	Column B	iRBCL
6		
7	**Mann Whitney test**	
8	P value	0.0286
9	Exact or approximate P value?	Exact
10	P value summary	*
11	Significantly different (P < 0.05)?	Yes
12	One- or two-tailed P value?	Two-tailed
13	Sum of ranks in column B,D	10 , 26
14	Mann-Whitney U	0
15		
16	**Difference between medians**	
17	Median of column B	0.8231, n=4
18	Median of column D	1.705, n=4
19	Difference: Actual	0.8818
20	Difference: Hodges-Lehmann	0.8818

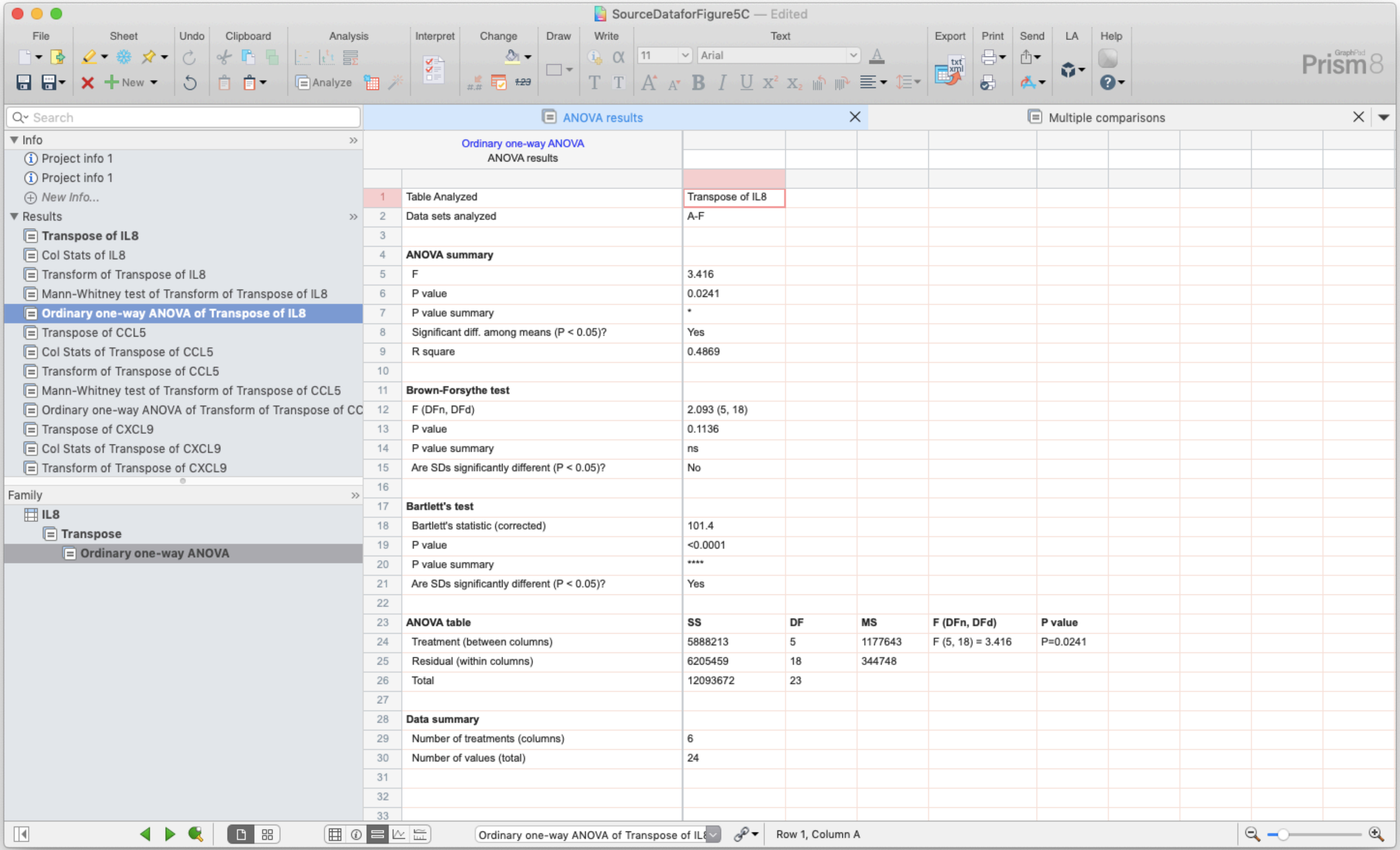

1	Table Analyzed	Transpose of IL8				
2	Data sets analyzed	A-F				
3						
4	**ANOVA summary**					
5	F	3.416				
6	P value	0.0241				
7	P value summary	*				
8	Significant diff. among means (P < 0.05)?	Yes				
9	R square	0.4869				
10						
11	**Brown-Forsythe test**					
12	F (DFn, DFd)	2.093 (5, 18)				
13	P value	0.1136				
14	P value summary	ns				
15	Are SDs significantly different (P < 0.05)?	No				
16						
17	**Bartlett's test**					
18	Bartlett's statistic (corrected)	101.4				
19	P value	<0.0001				
20	P value summary	****				
21	Are SDs significantly different (P < 0.05)?	Yes				
22						
23	**ANOVA table**	**SS**	**DF**	**MS**	**F (DFn, DFd)**	**P value**
24	Treatment (between columns)	5888213	5	1177643	F (5, 18) = 3.416	P=0.0241
25	Residual (within columns)	6205459	18	344748		
26	Total	12093672	23			
27						
28	**Data summary**					
29	Number of treatments (columns)	6				
30	Number of values (total)	24				
31						
32						
33						

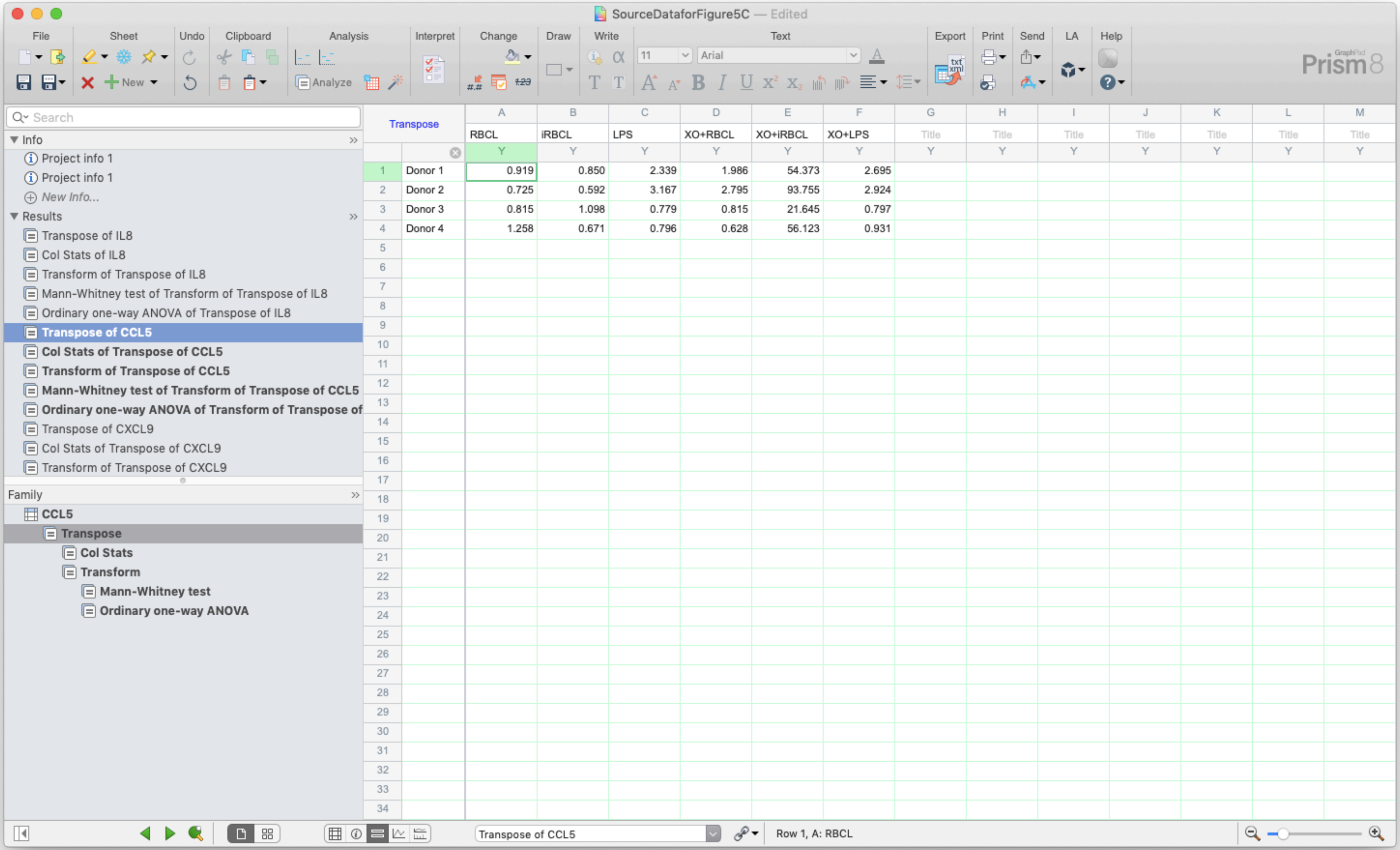

SourceDataforFigure5C — Edited
File
Sheet
Undo
Clipboard
Analysis
Interpret
Change
Draw
Write
Text
Export
Print
Send
LA
Help
New
Analyze
Arial
11
Prism 8
Search
Info
Project info 1
Project info 1
New Info...
Results
Transpose of IL8
Col Stats of IL8
Transform of Transpose of IL8
Mann-Whitney test of Transform of Transpose of IL8
Ordinary one-way ANOVA of Transpose of IL8
Transpose of CCL5
Col Stats of Transpose of CCL5
Transform of Transpose of CCL5
Mann-Whitney test of Transform of Transpose of CCL5
Ordinary one-way ANOVA of Transform of Transpose of
Transpose of CXCL9
Col Stats of Transpose of CXCL9
Transform of Transpose of CXCL9
Family
CCL5
Transpose
Col Stats
Transform
Mann-Whitney test
Ordinary one-way ANOVA
Transpose
A B C D E F G H I J K L M
RBCL iRBCL LPS XO+RBCL XO+iRBCL XO+LPS
1 Donor 1 0.919 0.850 2.339 1.986 54.373 2.695
2 Donor 2 0.725 0.592 3.167 2.795 93.755 2.924
3 Donor 3 0.815 1.098 0.779 0.815 21.645 0.797
4 Donor 4 1.258 0.671 0.796 0.628 56.123 0.931
Transpose of CCL5
Row 1, A: RBCL

SourceDataforFigure5C — Edited

File | Sheet | Undo | Clipboard | Analysis | Interpret | Change | Draw | Write | Text | Export | Print | Send | LA | Help

Prism 8

Info

- Project info 1
- New Info...

Results

- Transpose of IL8
- Col Stats of IL8
- Transform of Transpose of IL8
- Mann-Whitney test of Transform of Transpose of IL8
- Ordinary one-way ANOVA of Transpose of IL8
- **Transpose of CCL5**
- **Col Stats of Transpose of CCL5**
- Transform of Transpose of CCL5
- Mann-Whitney test of Transform of Transpose of CCL5
- Ordinary one-way ANOVA of Transform of Transpose of CC
- Transpose of CXCL9
- Col Stats of Transpose of CXCL9
- Transform of Transpose of CXCL9
- Mann-Whitney test of Transform of Transpose of CXCL9

Family

- **CCL5**
  - **Transpose**
    - **Col Stats**

	Col Stats	A RBCL	B iRBCL	C LPS	D XO+RBCL	E XO+iRBCL	F XO+LPS	G Title	H Title	I Title	J Title
		Y	Y	Y	Y	Y	Y	Y	Y	Y	Y
1	Number of values	4	4	4	4	4	4				
2											
3	Minimum	0.7248	0.5918	0.7795	0.6284	21.65	0.7970				
4	25% Percentile	0.7473	0.6116	0.7835	0.6750	29.83	0.8306				
5	Median	0.8668	0.7605	1.568	1.400	55.25	1.813				
6	75% Percentile	1.173	1.036	2.960	2.593	84.35	2.867				
7	Maximum	1.258	1.098	3.167	2.795	93.76	2.924				
8											
9	Mean	0.9290	0.8028	1.770	1.556	56.47	1.837				
10	Std. Deviation	0.2331	0.2247	1.184	1.021	29.48	1.128				
11	Std. Error of Mean	0.1165	0.1124	0.5921	0.5107	14.74	0.5641				
12											
13	Lower 95% CI of mean	0.5582	0.4452	-0.1138	-0.06921	9.562	0.04144				
14	Upper 95% CI of mean	1.300	1.160	3.655	3.181	103.4	3.632				
15											
16	Sum	3.716	3.211	7.082	6.224	225.9	7.347				
17											
18	**D'Agostino & Pearson normality test**										
19	K2	N too small	N too small	N too small	N too small	N too small	N too small				
20	P value										
21	Passed normality test (alpha=0.05)?										
22	P value summary										
23											
24	**Shapiro-Wilk normality test**										
25	W	0.9031	0.9430	0.8561	0.9059	0.9530	0.8066				
26	P value	0.4464	0.6724	0.2466	0.4611	0.7351	0.1146				
27	Passed normality test (alpha=0.05)?	Yes	Yes	Yes	Yes	Yes	Yes				
28	P value summary	ns	ns	ns	ns	ns	ns				
29											
30	**KS normality test**										
31	KS distance	N too small	N too small	N too small	N too small	N too small	N too small				
32	P value										
33	Passed normality test (alpha=0.05)?										
34	P value summary										

Col Stats of Transpose of CCL5 | Row 2, Column RT

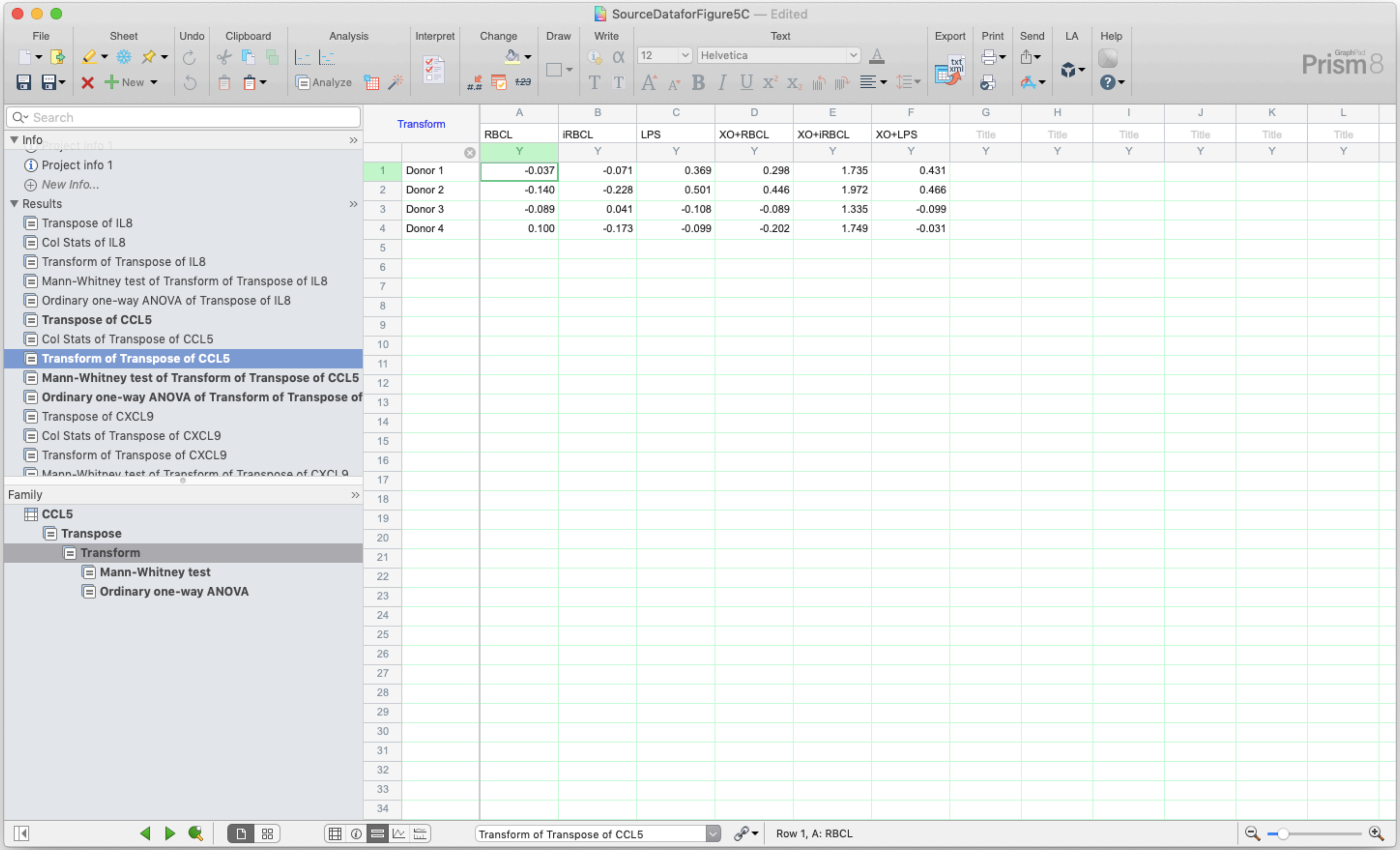

Transform	RBCL	iRBCL	LPS	XO+RBCL	XO+iRBCL	XO+LPS
Donor 1	-0.037	-0.071	0.369	0.298	1.735	0.431
Donor 2	-0.140	-0.228	0.501	0.446	1.972	0.466
Donor 3	-0.089	0.041	-0.108	-0.089	1.335	-0.099
Donor 4	0.100	-0.173	-0.099	-0.202	1.749	-0.031

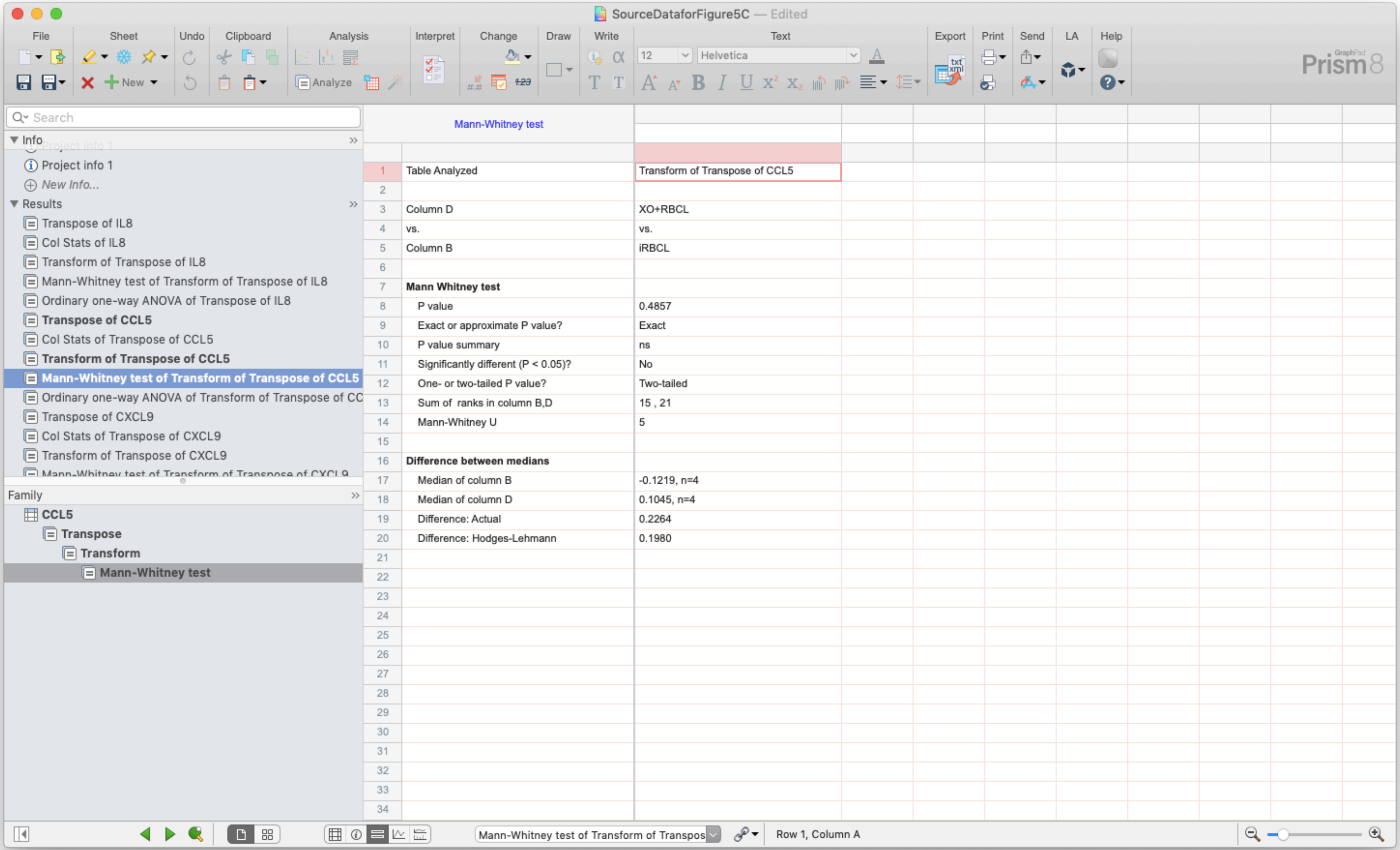

Mann-Whitney test

1	Table Analyzed	Transform of Transpose of CCL5
2		
3	Column D	XO+RBCL
4	vs.	vs.
5	Column B	iRBCL
6		
7	**Mann Whitney test**	
8	P value	0.4857
9	Exact or approximate P value?	Exact
10	P value summary	ns
11	Significantly different (P < 0.05)?	No
12	One- or two-tailed P value?	Two-tailed
13	Sum of ranks in column B,D	15 , 21
14	Mann-Whitney U	5
15		
16	**Difference between medians**	
17	Median of column B	-0.1219, n=4
18	Median of column D	0.1045, n=4
19	Difference: Actual	0.2264
20	Difference: Hodges-Lehmann	0.1980

SourceDataforFigure5C — Edited

Results
- Transpose of IL8
- Col Stats of IL8
- Transform of Transpose of IL8
- Mann-Whitney test of Transform of Transpose of IL8
- Ordinary one-way ANOVA of Transpose of IL8
- **Transpose of CCL5**
- Col Stats of Transpose of CCL5
- **Transform of Transpose of CCL5**
- Mann-Whitney test of Transform of Transpose of CCL5
- **Ordinary one-way ANOVA of Transform of Transpose of**
- Transpose of CXCL9
- Col Stats of Transpose of CXCL9
- Transform of Transpose of CXCL9

Family
- CCL5
  - Transpose
    - Transform
      - Ordinary one-way ANOVA

ANOVA results | Multiple comparisons

Ordinary one-way ANOVA
ANOVA results

#						
1	Table Analyzed	Transform of Transpose of CCL5				
2	Data sets analyzed	A-F				
3						
4	**ANOVA summary**					
5	F	29.11				
6	P value	<0.0001				
7	P value summary	****				
8	Significant diff. among means (P < 0.05)?	Yes				
9	R square	0.8899				
10						
11	**Brown-Forsythe test**					
12	F (DFn, DFd)	3.383 (5, 18)				
13	P value	0.0250				
14	P value summary	*				
15	Are SDs significantly different (P < 0.05)?	Yes				
16						
17	**Bartlett's test**					
18	Bartlett's statistic (corrected)	5.219				
19	P value	0.3898				
20	P value summary	ns				
21	Are SDs significantly different (P < 0.05)?	No				
22						
23	**ANOVA table**	**SS**	**DF**	**MS**	**F (DFn, DFd)**	**P value**
24	Treatment (between columns)	9.175	5	1.835	F (5, 18) = 29.11	P<0.0001
25	Residual (within columns)	1.135	18	0.06303		
26	Total	10.31	23			
27						
28	**Data summary**					
29	Number of treatments (columns)	6				
30	Number of values (total)	24				

Ordinary one-way ANOVA of Transform of Tra… Row 1, Column A

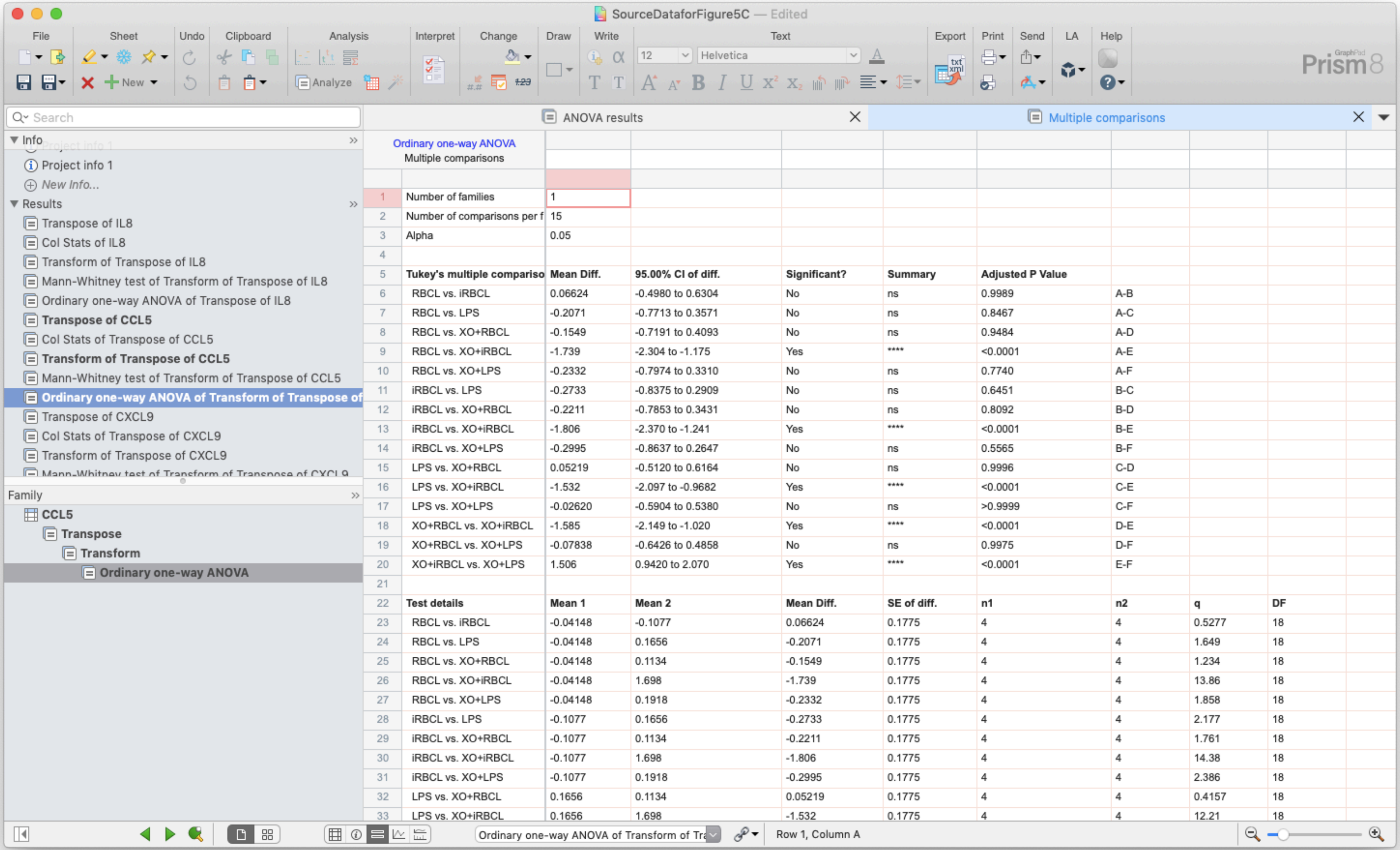

Ordinary one-way ANOVA
Multiple comparisons

1	Number of families	1							
2	Number of comparisons per f	15							
3	Alpha	0.05							
4									
5	**Tukey's multiple compariso**	**Mean Diff.**	**95.00% CI of diff.**	**Significant?**	**Summary**	**Adjusted P Value**			
6	RBCL vs. iRBCL	0.06624	-0.4980 to 0.6304	No	ns	0.9989	A-B		
7	RBCL vs. LPS	-0.2071	-0.7713 to 0.3571	No	ns	0.8467	A-C		
8	RBCL vs. XO+RBCL	-0.1549	-0.7191 to 0.4093	No	ns	0.9484	A-D		
9	RBCL vs. XO+iRBCL	-1.739	-2.304 to -1.175	Yes	****	<0.0001	A-E		
10	RBCL vs. XO+LPS	-0.2332	-0.7974 to 0.3310	No	ns	0.7740	A-F		
11	iRBCL vs. LPS	-0.2733	-0.8375 to 0.2909	No	ns	0.6451	B-C		
12	iRBCL vs. XO+RBCL	-0.2211	-0.7853 to 0.3431	No	ns	0.8092	B-D		
13	iRBCL vs. XO+iRBCL	-1.806	-2.370 to -1.241	Yes	****	<0.0001	B-E		
14	iRBCL vs. XO+LPS	-0.2995	-0.8637 to 0.2647	No	ns	0.5565	B-F		
15	LPS vs. XO+RBCL	0.05219	-0.5120 to 0.6164	No	ns	0.9996	C-D		
16	LPS vs. XO+iRBCL	-1.532	-2.097 to -0.9682	Yes	****	<0.0001	C-E		
17	LPS vs. XO+LPS	-0.02620	-0.5904 to 0.5380	No	ns	>0.9999	C-F		
18	XO+RBCL vs. XO+iRBCL	-1.585	-2.149 to -1.020	Yes	****	<0.0001	D-E		
19	XO+RBCL vs. XO+LPS	-0.07838	-0.6426 to 0.4858	No	ns	0.9975	D-F		
20	XO+iRBCL vs. XO+LPS	1.506	0.9420 to 2.070	Yes	****	<0.0001	E-F		
21									
22	**Test details**	**Mean 1**	**Mean 2**	**Mean Diff.**	**SE of diff.**	**n1**	**n2**	**q**	**DF**
23	RBCL vs. iRBCL	-0.04148	-0.1077	0.06624	0.1775	4	4	0.5277	18
24	RBCL vs. LPS	-0.04148	0.1656	-0.2071	0.1775	4	4	1.649	18
25	RBCL vs. XO+RBCL	-0.04148	0.1134	-0.1549	0.1775	4	4	1.234	18
26	RBCL vs. XO+iRBCL	-0.04148	1.698	-1.739	0.1775	4	4	13.86	18
27	RBCL vs. XO+LPS	-0.04148	0.1918	-0.2332	0.1775	4	4	1.858	18
28	iRBCL vs. LPS	-0.1077	0.1656	-0.2733	0.1775	4	4	2.177	18
29	iRBCL vs. XO+RBCL	-0.1077	0.1134	-0.2211	0.1775	4	4	1.761	18
30	iRBCL vs. XO+iRBCL	-0.1077	1.698	-1.806	0.1775	4	4	14.38	18
31	iRBCL vs. XO+LPS	-0.1077	0.1918	-0.2995	0.1775	4	4	2.386	18
32	LPS vs. XO+RBCL	0.1656	0.1134	0.05219	0.1775	4	4	0.4157	18
33	LPS vs. XO+iRBCL	0.1656	1.698	-1.532	0.1775	4	4	12.21	18

SourceDataforFigure5C — Edited

Search

Info
- Project info 1
- New Info...

Results
- Transpose of IL8
- Col Stats of IL8
- Transform of Transpose of IL8
- Mann-Whitney test of Transform of Transpose of IL8
- Ordinary one-way ANOVA of Transpose of IL8
- **Transpose of CCL5**
- Col Stats of Transpose of CCL5
- **Transform of Transpose of CCL5**
- Mann-Whitney test of Transform of Transpose of CCL5
- **Ordinary one-way ANOVA of Transform of Transpose of**
- Transpose of CXCL9
- Col Stats of Transpose of CXCL9
- Transform of Transpose of CXCL9
- Mann-Whitney test of Transform of Transpose of CXCL9

Family
- **CCL5**
  - **Transpose**
    - **Transform**
      - **Ordinary one-way ANOVA**

ANOVA results | Multiple comparisons

Ordinary one-way ANOVA
Multiple comparisons

11	iRBCL vs. LPS	-0.2733	-0.8375 to 0.2909	No	ns	0.6451	B-C		
12	iRBCL vs. XO+RBCL	-0.2211	-0.7853 to 0.3431	No	ns	0.8092	B-D		
13	iRBCL vs. XO+iRBCL	-1.806	-2.370 to -1.241	Yes	****	<0.0001	B-E		
14	iRBCL vs. XO+LPS	-0.2995	-0.8637 to 0.2647	No	ns	0.5565	B-F		
15	LPS vs. XO+RBCL	0.05219	-0.5120 to 0.6164	No	ns	0.9996	C-D		
16	LPS vs. XO+iRBCL	-1.532	-2.097 to -0.9682	Yes	****	<0.0001	C-E		
17	LPS vs. XO+LPS	-0.02620	-0.5904 to 0.5380	No	ns	>0.9999	C-F		
18	XO+RBCL vs. XO+iRBCL	-1.585	-2.149 to -1.020	Yes	****	<0.0001	D-E		
19	XO+RBCL vs. XO+LPS	-0.07838	-0.6426 to 0.4858	No	ns	0.9975	D-F		
20	XO+iRBCL vs. XO+LPS	1.506	0.9420 to 2.070	Yes	****	<0.0001	E-F		
21									
22	**Test details**	**Mean 1**	**Mean 2**	**Mean Diff.**	**SE of diff.**	**n1**	**n2**	**q**	**DF**
23	RBCL vs. iRBCL	-0.04148	-0.1077	0.06624	0.1775	4	4	0.5277	18
24	RBCL vs. LPS	-0.04148	0.1656	-0.2071	0.1775	4	4	1.649	18
25	RBCL vs. XO+RBCL	-0.04148	0.1134	-0.1549	0.1775	4	4	1.234	18
26	RBCL vs. XO+iRBCL	-0.04148	1.698	-1.739	0.1775	4	4	13.86	18
27	RBCL vs. XO+LPS	-0.04148	0.1918	-0.2332	0.1775	4	4	1.858	18
28	iRBCL vs. LPS	-0.1077	0.1656	-0.2733	0.1775	4	4	2.177	18
29	iRBCL vs. XO+RBCL	-0.1077	0.1134	-0.2211	0.1775	4	4	1.761	18
30	iRBCL vs. XO+iRBCL	-0.1077	1.698	-1.806	0.1775	4	4	14.38	18
31	iRBCL vs. XO+LPS	-0.1077	0.1918	-0.2995	0.1775	4	4	2.386	18
32	LPS vs. XO+RBCL	0.1656	0.1134	0.05219	0.1775	4	4	0.4157	18
33	LPS vs. XO+iRBCL	0.1656	1.698	-1.532	0.1775	4	4	12.21	18
34	LPS vs. XO+LPS	0.1656	0.1918	-0.02620	0.1775	4	4	0.2087	18
35	XO+RBCL vs. XO+iRBCL	0.1134	1.698	-1.585	0.1775	4	4	12.62	18
36	XO+RBCL vs. XO+LPS	0.1134	0.1918	-0.07838	0.1775	4	4	0.6244	18
37	XO+iRBCL vs. XO+LPS	1.698	0.1918	1.506	0.1775	4	4	12.00	18

Ordinary one-way ANOVA of Transform of Tra

Row 1, Column A

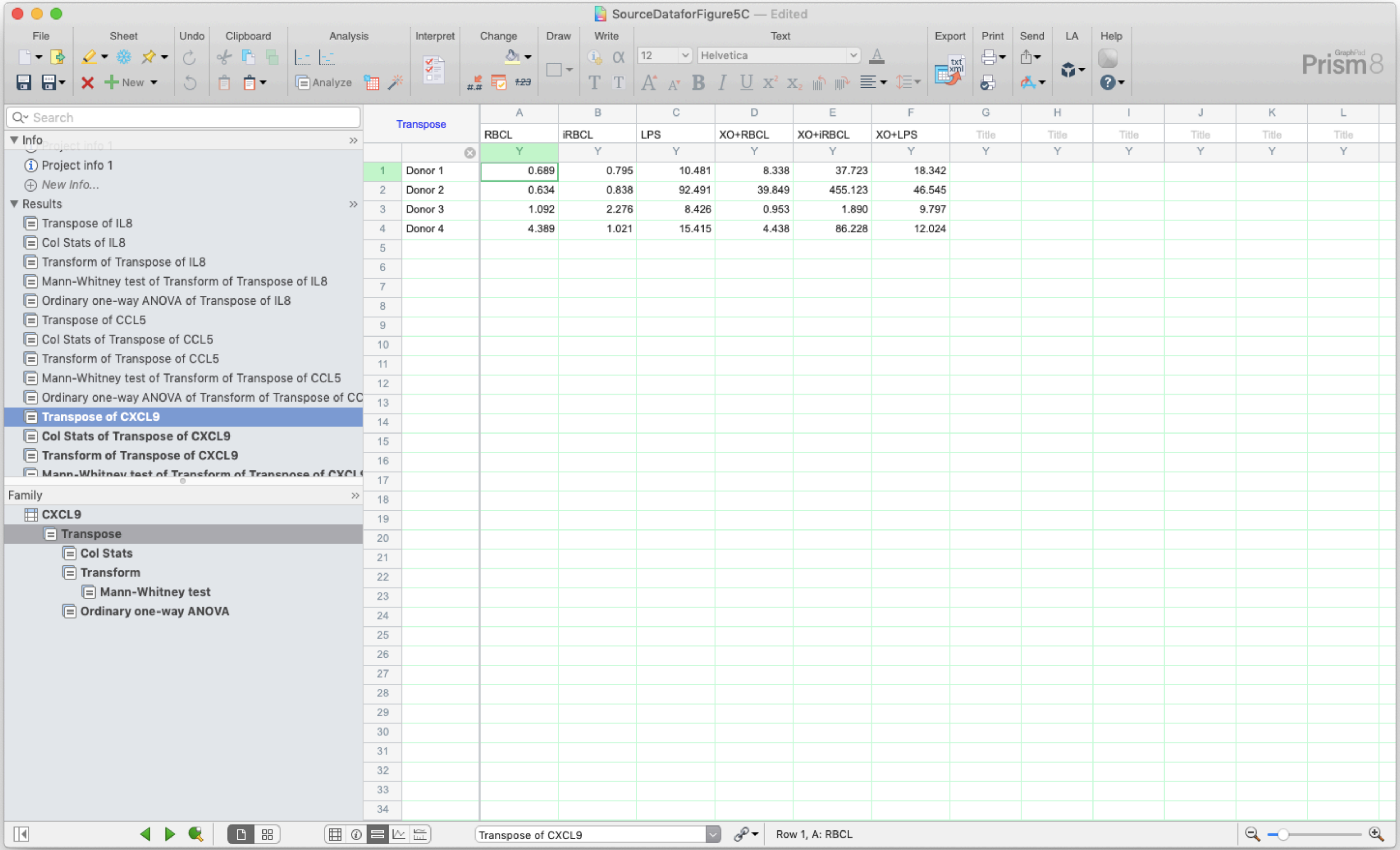

Transpose	RBCL	iRBCL	LPS	XO+RBCL	XO+iRBCL	XO+LPS
Donor 1	0.689	0.795	10.481	8.338	37.723	18.342
Donor 2	0.634	0.838	92.491	39.849	455.123	46.545
Donor 3	1.092	2.276	8.426	0.953	1.890	9.797
Donor 4	4.389	1.021	15.415	4.438	86.228	12.024

SourceDataforFigure5C — Edited

Col Stats of Transpose of CXCL9

	Col Stats	RBCL	iRBCL	LPS	XO+RBCL	XO+iRBCL	XO+LPS
19	K2	N too small	N too small	N too small	N too small	N too small	N too small
20	P value						
21	Passed normality test (alpha=0.05)?						
22	P value summary						
23							
24	**Shapiro-Wilk normality test**						
25	W	0.7183	0.7424	0.6932	0.7806	0.7759	0.8011
26	P value	0.0186	0.0327	0.0097	0.0717	0.0655	0.1042
27	Passed normality test (alpha=0.05)?	No	No	No	Yes	Yes	Yes
28	P value summary	*	*	**	ns	ns	ns
29							
30	**KS normality test**						
31	KS distance	N too small	N too small	N too small	N too small	N too small	N too small
32	P value						
33	Passed normality test (alpha=0.05)?						
34	P value summary						

Results:
- Transpose of IL8
- Col Stats of IL8
- Transform of Transpose of IL8
- Mann-Whitney test of Transform of Transpose of IL8
- Ordinary one-way ANOVA of Transpose of IL8
- Transpose of CCL5
- Col Stats of Transpose of CCL5
- Transform of Transpose of CCL5
- Mann-Whitney test of Transform of Transpose of CCL5
- Ordinary one-way ANOVA of Transform of Transpose of CC
- Transpose of CXCL9
- Col Stats of Transpose of CXCL9
- Transform of Transpose of CXCL9

Family: CXCL9 > Transpose > Col Stats

Row 1, A: RBCL

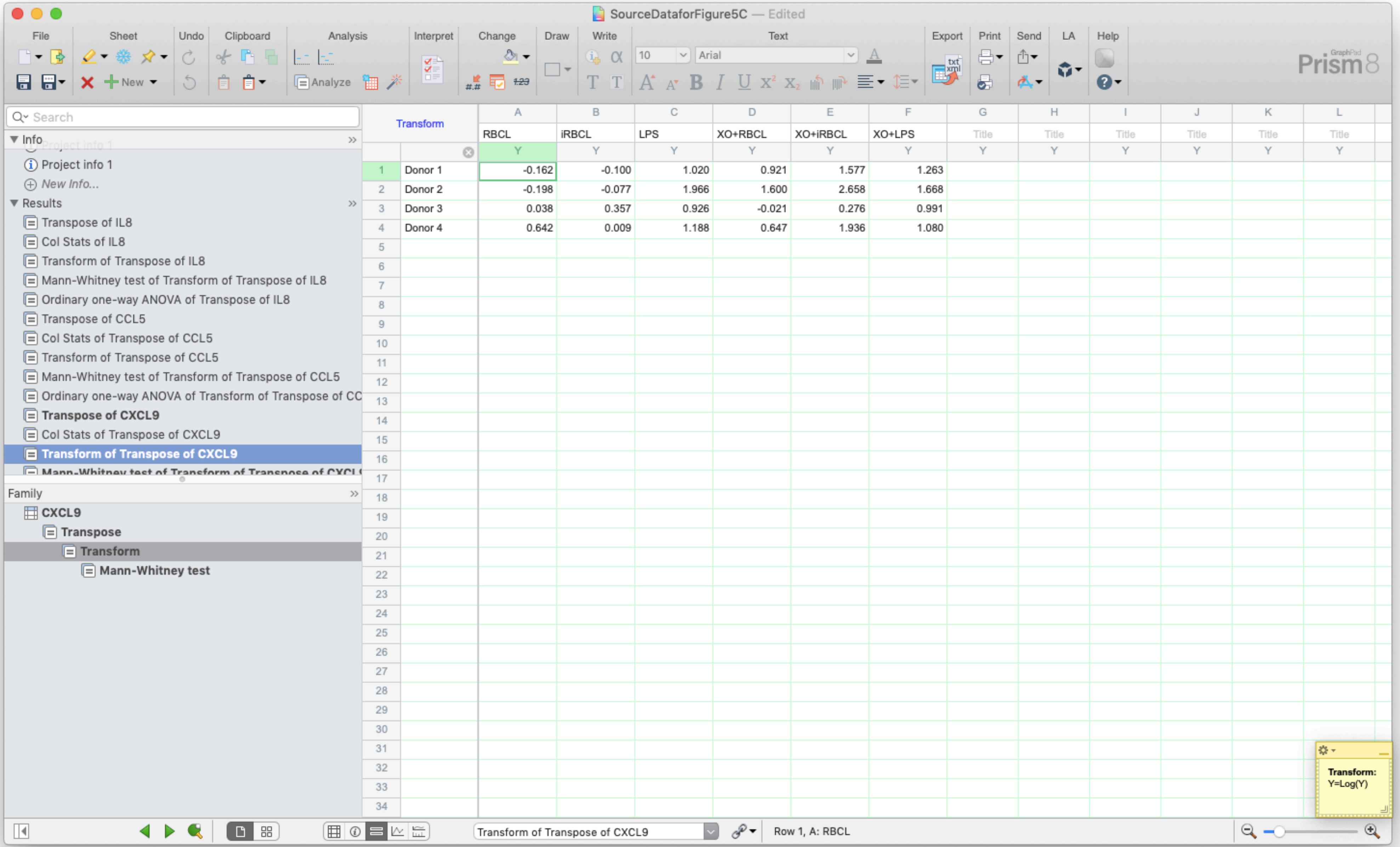

	RBCL	iRBCL	LPS	XO+RBCL	XO+iRBCL	XO+LPS
Donor 1	-0.162	-0.100	1.020	0.921	1.577	1.263
Donor 2	-0.198	-0.077	1.966	1.600	2.658	1.668
Donor 3	0.038	0.357	0.926	-0.021	0.276	0.991
Donor 4	0.642	0.009	1.188	0.647	1.936	1.080

SourceDataforFigure5C — Edited

Info
- Project info 1
- New Info...

Results
- Transpose of IL8
- Col Stats of IL8
- Transform of Transpose of IL8
- Mann-Whitney test of Transform of Transpose of IL8
- Ordinary one-way ANOVA of Transpose of IL8
- Transpose of CCL5
- Col Stats of Transpose of CCL5
- Transform of Transpose of CCL5
- Mann-Whitney test of Transform of Transpose of CCL5
- Ordinary one-way ANOVA of Transform of Transpose of CC
- **Transpose of CXCL9**
- Col Stats of Transpose of CXCL9
- **Transform of Transpose of CXCL9**
- **Mann-Whitney test of Transform of Transpose of CXCL**

Family
- CXCL9
  - Transpose
    - Transform
      - Mann-Whitney test

	Mann-Whitney test	
1	Table Analyzed	Transform of Transpose of CXCL9
2		
3	Column D	XO+RBCL
4	vs.	vs.
5	Column B	iRBCL
6		
7	**Mann Whitney test**	
8	P value	0.1143
9	Exact or approximate P value?	Exact
10	P value summary	ns
11	Significantly different (P < 0.05)?	No
12	One- or two-tailed P value?	Two-tailed
13	Sum of ranks in column B,D	12 , 24
14	Mann-Whitney U	2
15		
16	**Difference between medians**	
17	Median of column B	-0.03373, n=4
18	Median of column D	0.7841, n=4
19	Difference: Actual	0.8179
20	Difference: Hodges-Lehmann	0.7353

Mann-Whitney test of Transform of Transpos

Row 1, Column A

SourceDataforFigure5C — Edited

Results
- Transform of Transpose of CCL5
- Mann-Whitney test of Transform of Transpose of CCL5
- Ordinary one-way ANOVA of Transform of Transpose of CC
- **Transpose of CXCL9**
- Col Stats of Transpose of CXCL9
- Transform of Transpose of CXCL9
- Mann-Whitney test of Transform of Transpose of CXCL9
- **Ordinary one-way ANOVA of Transpose of CXCL9**
- Transpose of CCL2
- Col Stats of Transpose of CCL2
- Transform of Transpose of CCL2
- Mann-Whitney test of Transform of Transpose of CCL2
- Ordinary one-way ANOVA of Transform of Transpose of CC
- *New Analysis...*

Graphs
- IL8
- IL8 line

Family
- **CXCL9**
  - **Transpose**
    - **Ordinary one-way ANOVA**

ANOVA results | Multiple comparisons

Ordinary one-way ANOVA
ANOVA results

1	Table Analyzed	Transpose of CXCL9				
2	Data sets analyzed	A-F				
3						
4	**ANOVA summary**					
5	F	1.566				
6	P value	0.2198				
7	P value summary	ns				
8	Significant diff. among means (P < 0.05)?	No				
9	R square	0.3032				
10						
11	**Brown-Forsythe test**					
12	F (DFn, DFd)	1.638 (5, 18)				
13	P value	0.2008				
14	P value summary	ns				
15	Are SDs significantly different (P < 0.05)?	No				
16						
17	**Bartlett's test**					
18	Bartlett's statistic (corrected)	62.89				
19	P value	<0.0001				
20	P value summary	****				
21	Are SDs significantly different (P < 0.05)?	Yes				
22						
23	**ANOVA table**	**SS**	**DF**	**MS**	**F (DFn, DFd)**	**P value**
24	Treatment (between columns)	60213	5	12043	F (5, 18) = 1.566	P=0.2198
25	Residual (within columns)	138407	18	7689		
26	Total	198620	23			
27						
28	**Data summary**					
29	Number of treatments (columns)	6				
30	Number of values (total)	24				
31						
32						
33						

Ordinary one-way ANOVA of Transpose of CX
Row 1, Column A

SourceDataforFigure5C — Edited

Results

- Transform of Transpose of CCL5
- Mann-Whitney test of Transform of Transpose of CCL5
- Ordinary one-way ANOVA of Transform of Transpose of CC
- **Transpose of CXCL9**
- Col Stats of Transpose of CXCL9
- Transform of Transpose of CXCL9
- Mann-Whitney test of Transform of Transpose of CXCL9
- **Ordinary one-way ANOVA of Transpose of CXCL9**
- Transpose of CCL2
- Col Stats of Transpose of CCL2
- Transform of Transpose of CCL2
- Mann-Whitney test of Transform of Transpose of CCL2
- Ordinary one-way ANOVA of Transform of Transpose of CC
- New Analysis...

Graphs

- IL8
- IL8 line

Family

- **CXCL9**
  - **Transpose**
    - **Ordinary one-way ANOVA**

ANOVA results | Multiple comparisons

Ordinary one-way ANOVA
Multiple comparisons

1	Number of families	1							
2	Number of comparisons per family	15							
3	Alpha	0.05							
4									
5	**Tukey's multiple comparisons tes**	**Mean Diff.**	**95.00% CI of diff.**	**Significant?**	**Summary**	**Adjusted P Value**			
6	RBCL vs. iRBCL	0.4684	-196.6 to 197.5	No	ns	>0.9999	A-B		
7	RBCL vs. LPS	-30.00	-227.1 to 167.1	No	ns	0.9962	A-C		
8	RBCL vs. XO+RBCL	-11.69	-208.7 to 185.4	No	ns	>0.9999	A-D		
9	RBCL vs. XO+iRBCL	-143.5	-340.6 to 53.51	No	ns	0.2384	A-E		
10	RBCL vs. XO+LPS	-19.98	-217.0 to 177.1	No	ns	0.9995	A-F		
11	iRBCL vs. LPS	-30.47	-227.5 to 166.6	No	ns	0.9959	B-C		
12	iRBCL vs. XO+RBCL	-12.16	-209.2 to 184.9	No	ns	>0.9999	B-D		
13	iRBCL vs. XO+iRBCL	-144.0	-341.1 to 53.05	No	ns	0.2356	B-E		
14	iRBCL vs. XO+LPS	-20.44	-217.5 to 176.6	No	ns	0.9994	B-F		
15	LPS vs. XO+RBCL	18.31	-178.7 to 215.4	No	ns	0.9996	C-D		
16	LPS vs. XO+iRBCL	-113.5	-310.6 to 83.52	No	ns	0.4719	C-E		
17	LPS vs. XO+LPS	10.03	-187.0 to 207.1	No	ns	>0.9999	C-F		
18	XO+RBCL vs. XO+iRBCL	-131.8	-328.9 to 65.21	No	ns	0.3179	D-E		
19	XO+RBCL vs. XO+LPS	-8.282	-205.3 to 188.8	No	ns	>0.9999	D-F		
20	XO+iRBCL vs. XO+LPS	123.6	-73.49 to 320.6	No	ns	0.3835	E-F		
21									
22	**Test details**	**Mean 1**	**Mean 2**	**Mean Diff.**	**SE of diff.**	**n1**	**n2**	**q**	**DF**
23	RBCL vs. iRBCL	1.701	1.233	0.4684	62.01	4	4	0.01068	18
24	RBCL vs. LPS	1.701	31.70	-30.00	62.01	4	4	0.6843	18
25	RBCL vs. XO+RBCL	1.701	13.39	-11.69	62.01	4	4	0.2667	18
26	RBCL vs. XO+iRBCL	1.701	145.2	-143.5	62.01	4	4	3.274	18
27	RBCL vs. XO+LPS	1.701	21.68	-19.98	62.01	4	4	0.4556	18
28	iRBCL vs. LPS	1.233	31.70	-30.47	62.01	4	4	0.6950	18
29	iRBCL vs. XO+RBCL	1.233	13.39	-12.16	62.01	4	4	0.2774	18
30	iRBCL vs. XO+iRBCL	1.233	145.2	-144.0	62.01	4	4	3.285	18
31	iRBCL vs. XO+LPS	1.233	21.68	-20.44	62.01	4	4	0.4663	18
32	LPS vs. XO+RBCL	31.70	13.39	18.31	62.01	4	4	0.4176	18
33	LPS vs. XO+iRBCL	31.70	145.2	-113.5	62.01	4	4	2.590	18

Ordinary one-way ANOVA of Transpose of CX

Row 1, Column A

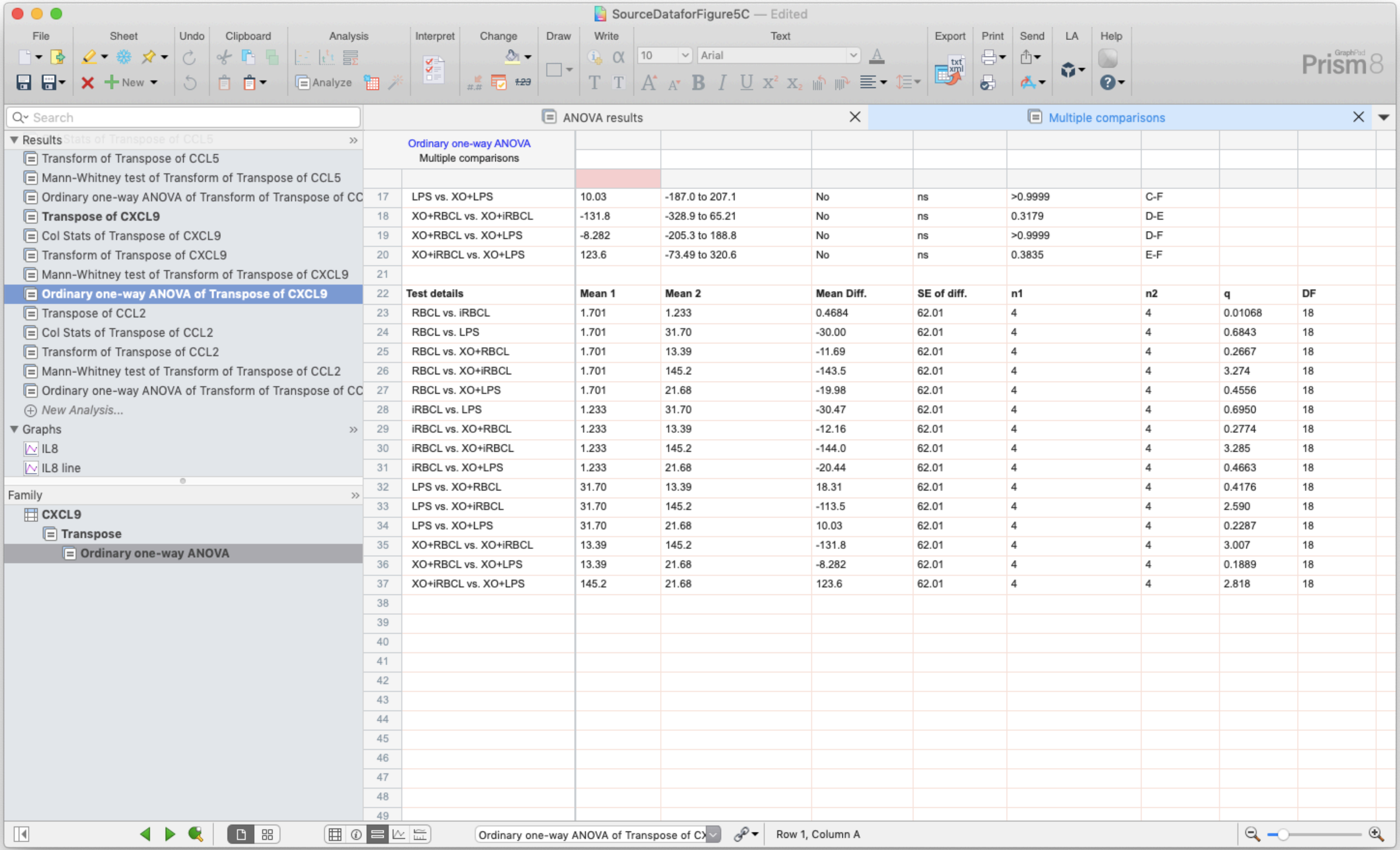

	Ordinary one-way ANOVA<br>Multiple comparisons								
17	LPS vs. XO+LPS	10.03	-187.0 to 207.1	No	ns	>0.9999	C-F		
18	XO+RBCL vs. XO+iRBCL	-131.8	-328.9 to 65.21	No	ns	0.3179	D-E		
19	XO+RBCL vs. XO+LPS	-8.282	-205.3 to 188.8	No	ns	>0.9999	D-F		
20	XO+iRBCL vs. XO+LPS	123.6	-73.49 to 320.6	No	ns	0.3835	E-F		
21									
22	**Test details**	**Mean 1**	**Mean 2**	**Mean Diff.**	**SE of diff.**	**n1**	**n2**	**q**	**DF**
23	RBCL vs. iRBCL	1.701	1.233	0.4684	62.01	4	4	0.01068	18
24	RBCL vs. LPS	1.701	31.70	-30.00	62.01	4	4	0.6843	18
25	RBCL vs. XO+RBCL	1.701	13.39	-11.69	62.01	4	4	0.2667	18
26	RBCL vs. XO+iRBCL	1.701	145.2	-143.5	62.01	4	4	3.274	18
27	RBCL vs. XO+LPS	1.701	21.68	-19.98	62.01	4	4	0.4556	18
28	iRBCL vs. LPS	1.233	31.70	-30.47	62.01	4	4	0.6950	18
29	iRBCL vs. XO+RBCL	1.233	13.39	-12.16	62.01	4	4	0.2774	18
30	iRBCL vs. XO+iRBCL	1.233	145.2	-144.0	62.01	4	4	3.285	18
31	iRBCL vs. XO+LPS	1.233	21.68	-20.44	62.01	4	4	0.4663	18
32	LPS vs. XO+RBCL	31.70	13.39	18.31	62.01	4	4	0.4176	18
33	LPS vs. XO+iRBCL	31.70	145.2	-113.5	62.01	4	4	2.590	18
34	LPS vs. XO+LPS	31.70	21.68	10.03	62.01	4	4	0.2287	18
35	XO+RBCL vs. XO+iRBCL	13.39	145.2	-131.8	62.01	4	4	3.007	18
36	XO+RBCL vs. XO+LPS	13.39	21.68	-8.282	62.01	4	4	0.1889	18
37	XO+iRBCL vs. XO+LPS	145.2	21.68	123.6	62.01	4	4	2.818	18

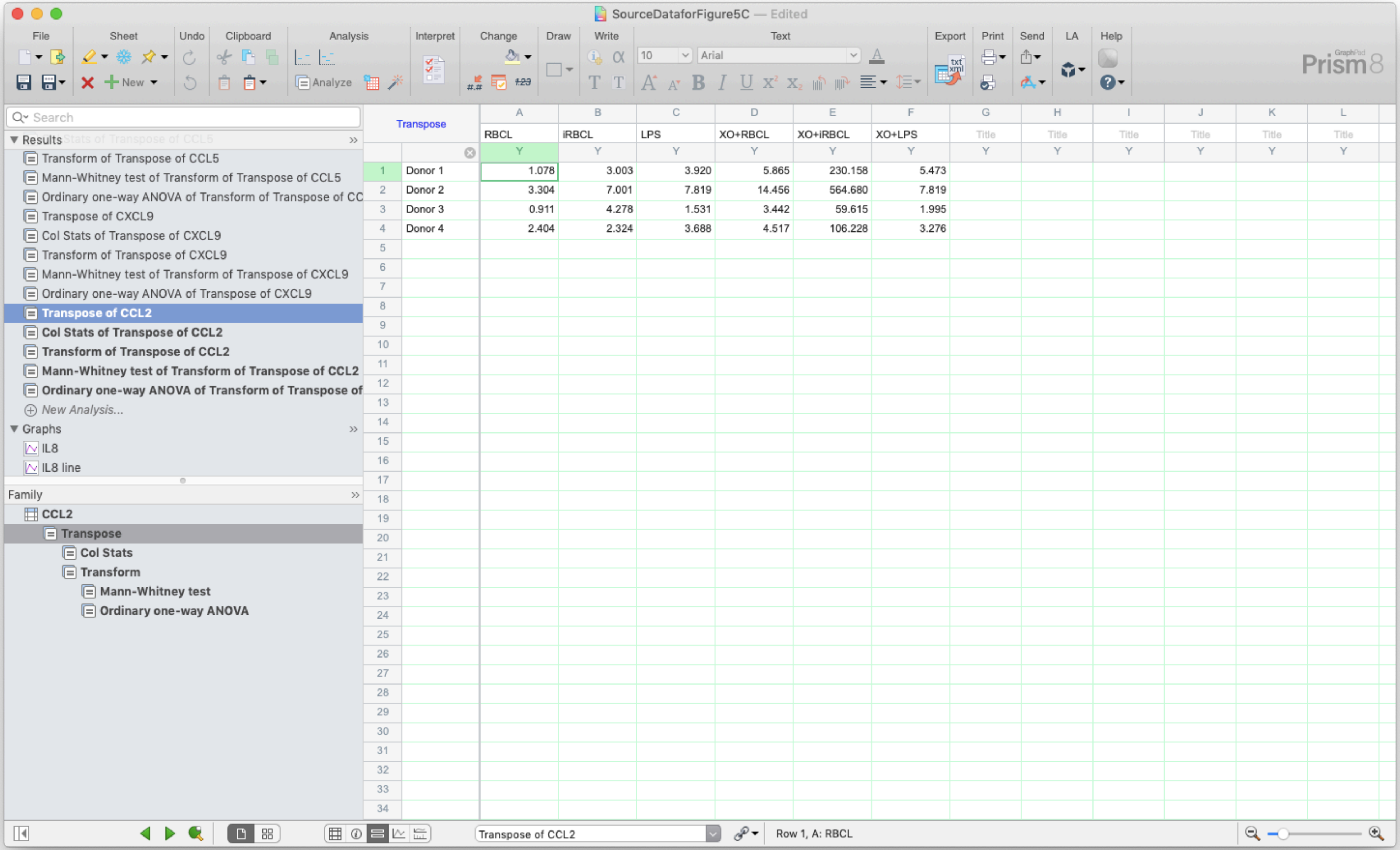

Transpose	RBCL	iRBCL	LPS	XO+RBCL	XO+iRBCL	XO+LPS
Donor 1	1.078	3.003	3.920	5.865	230.158	5.473
Donor 2	3.304	7.001	7.819	14.456	564.680	7.819
Donor 3	0.911	4.278	1.531	3.442	59.615	1.995
Donor 4	2.404	2.324	3.688	4.517	106.228	3.276

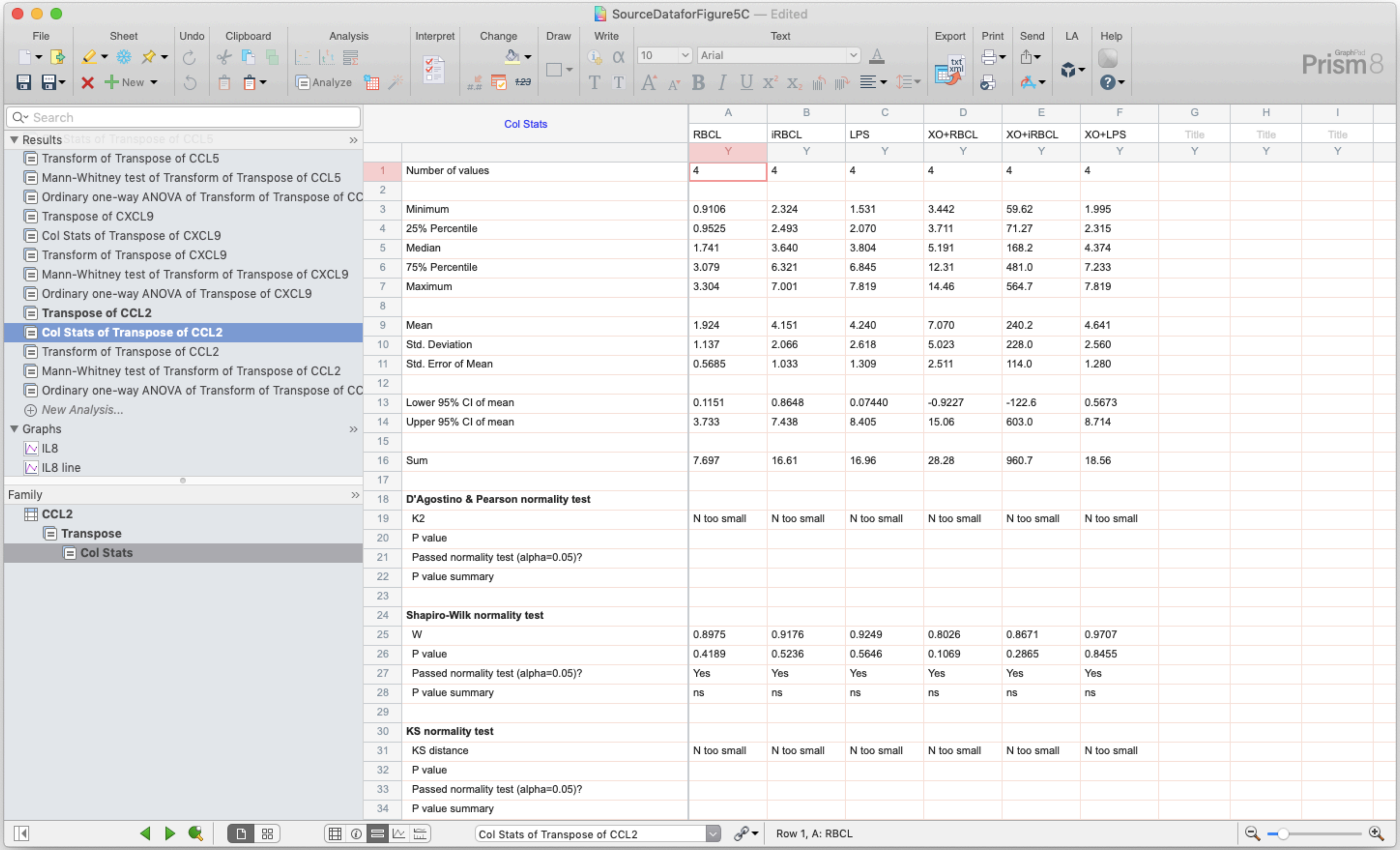

Col Stats	RBCL	iRBCL	LPS	XO+RBCL	XO+iRBCL	XO+LPS
Number of values	4	4	4	4	4	4
Minimum	0.9106	2.324	1.531	3.442	59.62	1.995
25% Percentile	0.9525	2.493	2.070	3.711	71.27	2.315
Median	1.741	3.640	3.804	5.191	168.2	4.374
75% Percentile	3.079	6.321	6.845	12.31	481.0	7.233
Maximum	3.304	7.001	7.819	14.46	564.7	7.819
Mean	1.924	4.151	4.240	7.070	240.2	4.641
Std. Deviation	1.137	2.066	2.618	5.023	228.0	2.560
Std. Error of Mean	0.5685	1.033	1.309	2.511	114.0	1.280
Lower 95% CI of mean	0.1151	0.8648	0.07440	-0.9227	-122.6	0.5673
Upper 95% CI of mean	3.733	7.438	8.405	15.06	603.0	8.714
Sum	7.697	16.61	16.96	28.28	960.7	18.56
**D'Agostino & Pearson normality test**						
K2	N too small	N too small	N too small	N too small	N too small	N too small
P value						
Passed normality test (alpha=0.05)?						
P value summary						
**Shapiro-Wilk normality test**						
W	0.8975	0.9176	0.9249	0.8026	0.8671	0.9707
P value	0.4189	0.5236	0.5646	0.1069	0.2865	0.8455
Passed normality test (alpha=0.05)?	Yes	Yes	Yes	Yes	Yes	Yes
P value summary	ns	ns	ns	ns	ns	ns
**KS normality test**						
KS distance	N too small	N too small	N too small	N too small	N too small	N too small
P value						
Passed normality test (alpha=0.05)?						
P value summary						

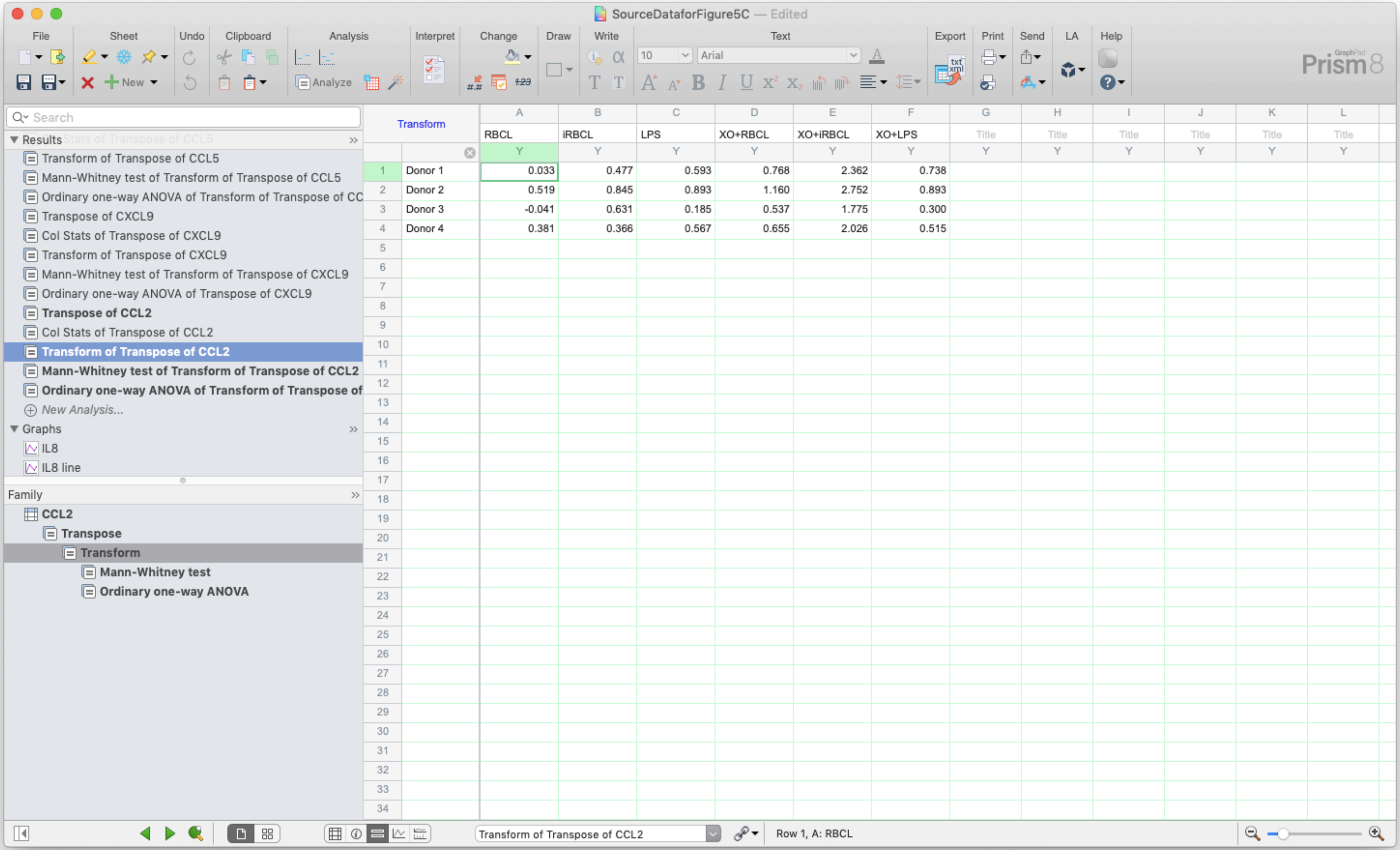

Transform	RBCL	iRBCL	LPS	XO+RBCL	XO+iRBCL	XO+LPS
Donor 1	0.033	0.477	0.593	0.768	2.362	0.738
Donor 2	0.519	0.845	0.893	1.160	2.752	0.893
Donor 3	-0.041	0.631	0.185	0.537	1.775	0.300
Donor 4	0.381	0.366	0.567	0.655	2.026	0.515

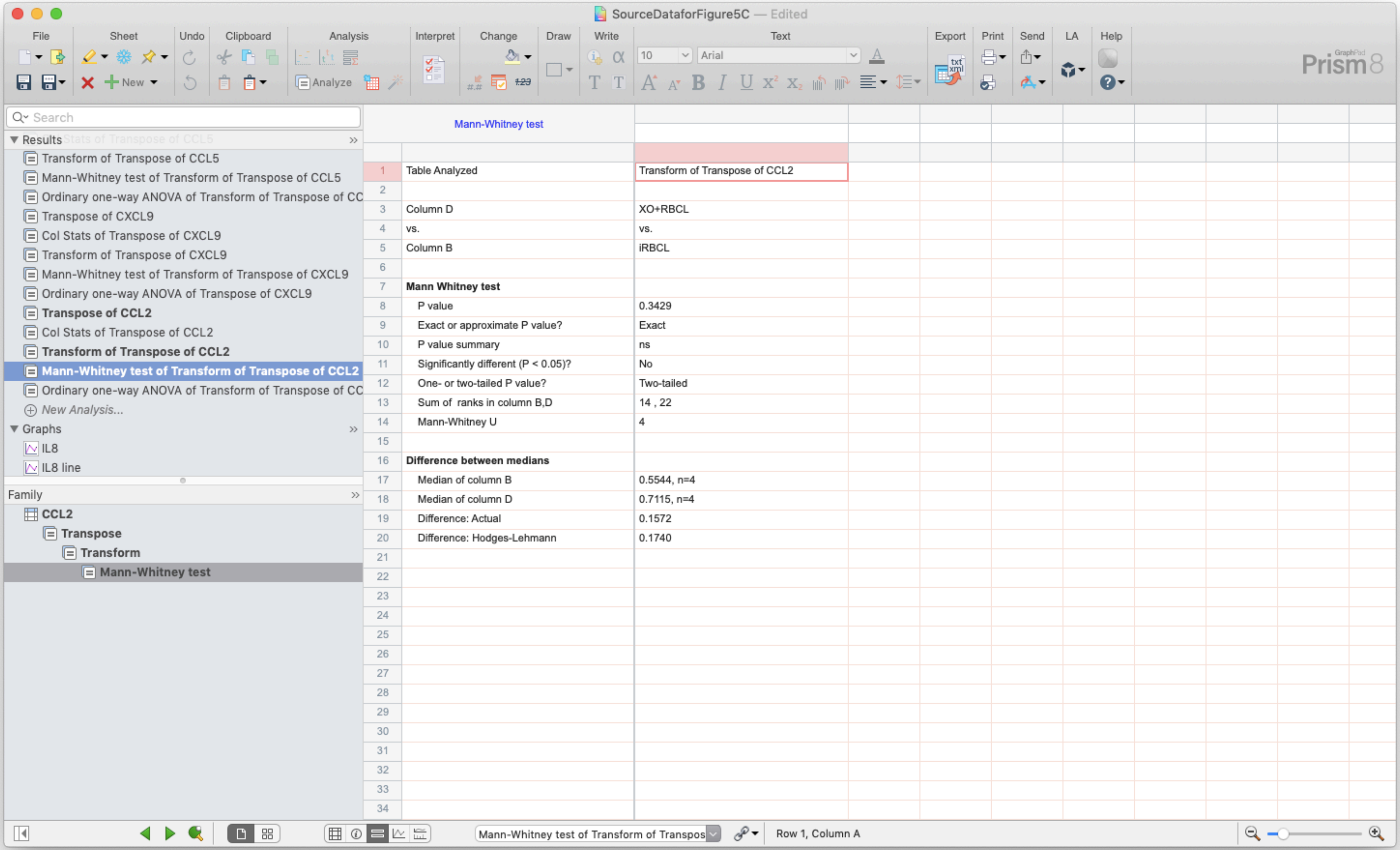

	Mann-Whitney test	
1	Table Analyzed	Transform of Transpose of CCL2
2		
3	Column D	XO+RBCL
4	vs.	vs.
5	Column B	iRBCL
6		
7	**Mann Whitney test**	
8	P value	0.3429
9	Exact or approximate P value?	Exact
10	P value summary	ns
11	Significantly different (P < 0.05)?	No
12	One- or two-tailed P value?	Two-tailed
13	Sum of ranks in column B,D	14 , 22
14	Mann-Whitney U	4
15		
16	**Difference between medians**	
17	Median of column B	0.5544, n=4
18	Median of column D	0.7115, n=4
19	Difference: Actual	0.1572
20	Difference: Hodges-Lehmann	0.1740

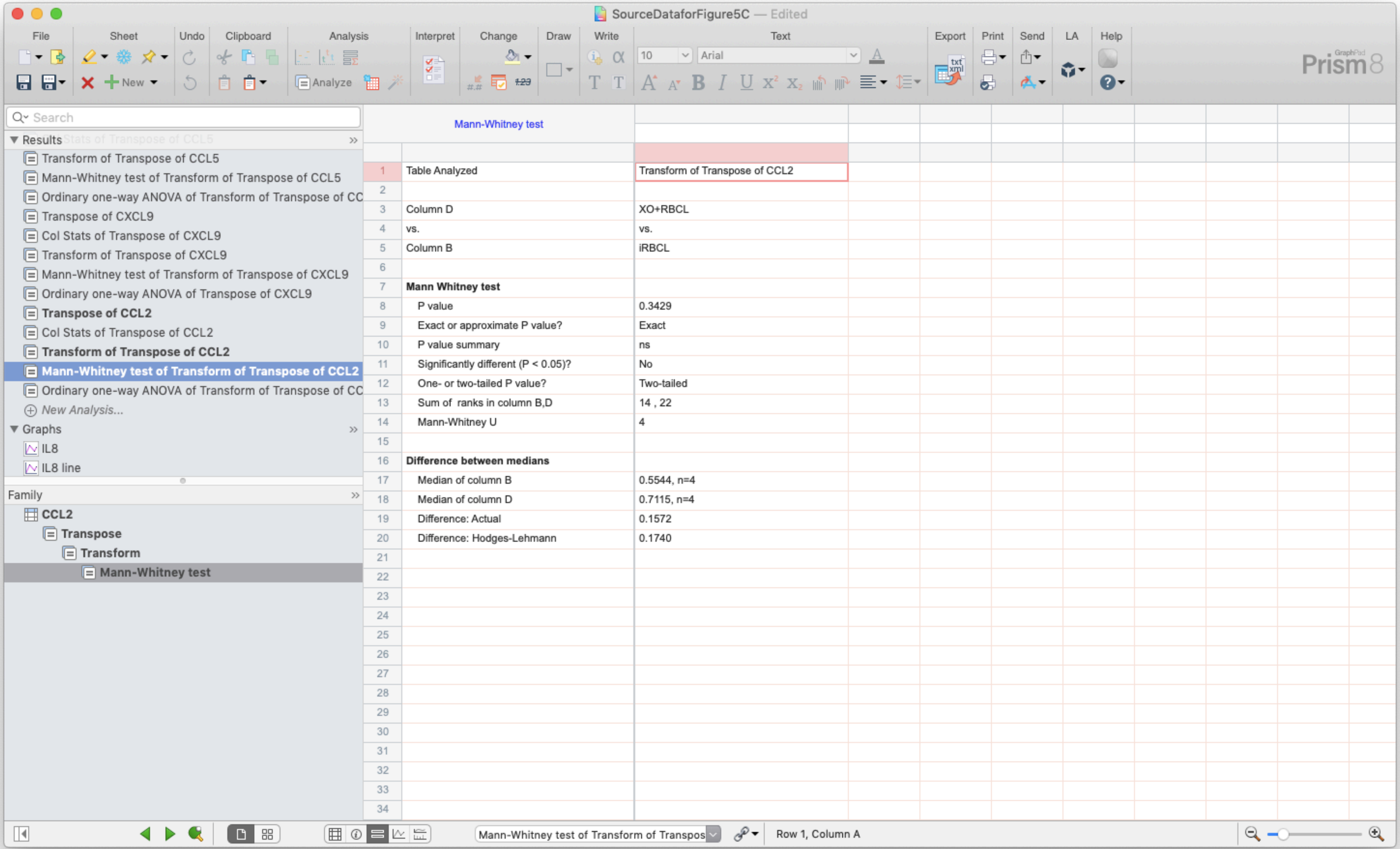

Mann-Whitney test	
Table Analyzed	Transform of Transpose of CCL2
Column D	XO+RBCL
vs.	vs.
Column B	iRBCL
**Mann Whitney test**	
P value	0.3429
Exact or approximate P value?	Exact
P value summary	ns
Significantly different (P < 0.05)?	No
One- or two-tailed P value?	Two-tailed
Sum of ranks in column B,D	14 , 22
Mann-Whitney U	4
**Difference between medians**	
Median of column B	0.5544, n=4
Median of column D	0.7115, n=4
Difference: Actual	0.1572
Difference: Hodges-Lehmann	0.1740

SourceDataforFigure5C — Edited

File | Sheet | Undo | Clipboard | Analysis | Interpret | Change | Draw | Write | Text | Export | Print | Send | LA | Help

Results
- Transform of Transpose of CCL5
- Mann-Whitney test of Transform of Transpose of CCL5
- Ordinary one-way ANOVA of Transform of Transpose of CC
- Transpose of CXCL9
- Col Stats of Transpose of CXCL9
- Transform of Transpose of CXCL9
- Mann-Whitney test of Transform of Transpose of CXCL9
- Ordinary one-way ANOVA of Transpose of CXCL9
- **Transpose of CCL2**
- Col Stats of Transpose of CCL2
- **Transform of Transpose of CCL2**
- Mann-Whitney test of Transform of Transpose of CCL2
- **Ordinary one-way ANOVA of Transform of Transpose of**
- *New Analysis...*

Graphs
- IL8
- IL8 line

Family
- **CCL2**
  - **Transpose**
    - **Transform**
      - **Ordinary one-way ANOVA**

ANOVA results | Multiple comparisons

	Ordinary one-way ANOVA<br>ANOVA results					
1	Table Analyzed	Transform of Transpose of CCL2				
2	Data sets analyzed	A-F				
3						
4	**ANOVA summary**					
5	F	23.19				
6	P value	<0.0001				
7	P value summary	****				
8	Significant diff. among means (P < 0.05)?	Yes				
9	R square	0.8656				
10						
11	**Brown-Forsythe test**					
12	F (DFn, DFd)	0.6310 (5, 18)				
13	P value	0.6786				
14	P value summary	ns				
15	Are SDs significantly different (P < 0.05)?	No				
16						
17	**Bartlett's test**					
18	Bartlett's statistic (corrected)	1.584				
19	P value	0.9032				
20	P value summary	ns				
21	Are SDs significantly different (P < 0.05)?	No				
22						
23	**ANOVA table**	**SS**	**DF**	**MS**	**F (DFn, DFd)**	**P value**
24	Treatment (between columns)	10.04	5	2.009	F (5, 18) = 23.19	P<0.0001
25	Residual (within columns)	1.559	18	0.08663		
26	Total	11.60	23			
27						
28	**Data summary**					
29	Number of treatments (columns)	6				
30	Number of values (total)	24				
31						
32						
33						

Ordinary one-way ANOVA of Transform of Tr | Row 1, Column A

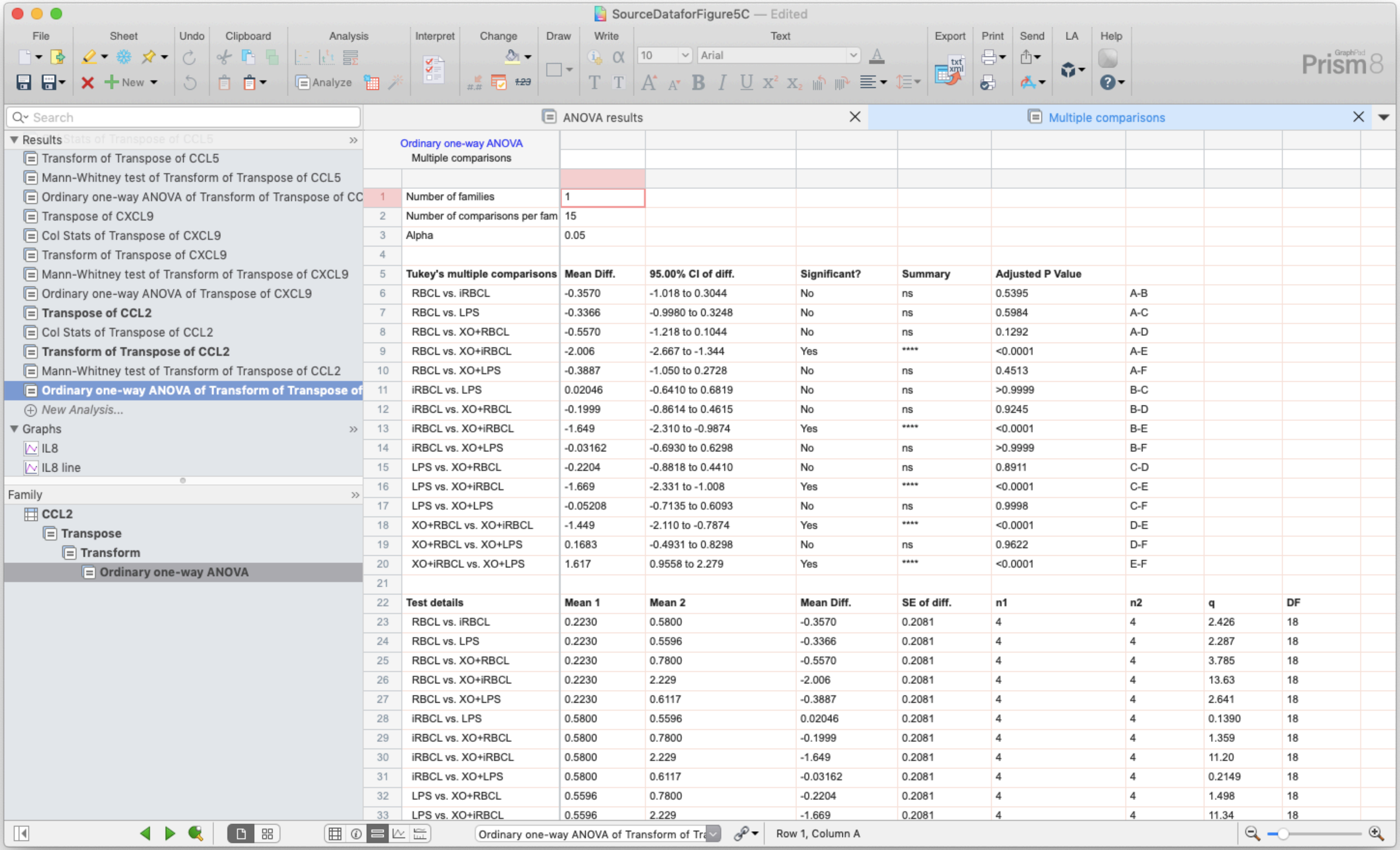

Ordinary one-way ANOVA
Multiple comparisons

1	Number of families	1
2	Number of comparisons per fam	15
3	Alpha	0.05
4		

	Tukey's multiple comparisons	Mean Diff.	95.00% CI of diff.	Significant?	Summary	Adjusted P Value	
6	RBCL vs. iRBCL	-0.3570	-1.018 to 0.3044	No	ns	0.5395	A-B
7	RBCL vs. LPS	-0.3366	-0.9980 to 0.3248	No	ns	0.5984	A-C
8	RBCL vs. XO+RBCL	-0.5570	-1.218 to 0.1044	No	ns	0.1292	A-D
9	RBCL vs. XO+iRBCL	-2.006	-2.667 to -1.344	Yes	****	<0.0001	A-E
10	RBCL vs. XO+LPS	-0.3887	-1.050 to 0.2728	No	ns	0.4513	A-F
11	iRBCL vs. LPS	0.02046	-0.6410 to 0.6819	No	ns	>0.9999	B-C
12	iRBCL vs. XO+RBCL	-0.1999	-0.8614 to 0.4615	No	ns	0.9245	B-D
13	iRBCL vs. XO+iRBCL	-1.649	-2.310 to -0.9874	Yes	****	<0.0001	B-E
14	iRBCL vs. XO+LPS	-0.03162	-0.6930 to 0.6298	No	ns	>0.9999	B-F
15	LPS vs. XO+RBCL	-0.2204	-0.8818 to 0.4410	No	ns	0.8911	C-D
16	LPS vs. XO+iRBCL	-1.669	-2.331 to -1.008	Yes	****	<0.0001	C-E
17	LPS vs. XO+LPS	-0.05208	-0.7135 to 0.6093	No	ns	0.9998	C-F
18	XO+RBCL vs. XO+iRBCL	-1.449	-2.110 to -0.7874	Yes	****	<0.0001	D-E
19	XO+RBCL vs. XO+LPS	0.1683	-0.4931 to 0.8298	No	ns	0.9622	D-F
20	XO+iRBCL vs. XO+LPS	1.617	0.9558 to 2.279	Yes	****	<0.0001	E-F
21							

	Test details	Mean 1	Mean 2	Mean Diff.	SE of diff.	n1	n2	q	DF
23	RBCL vs. iRBCL	0.2230	0.5800	-0.3570	0.2081	4	4	2.426	18
24	RBCL vs. LPS	0.2230	0.5596	-0.3366	0.2081	4	4	2.287	18
25	RBCL vs. XO+RBCL	0.2230	0.7800	-0.5570	0.2081	4	4	3.785	18
26	RBCL vs. XO+iRBCL	0.2230	2.229	-2.006	0.2081	4	4	13.63	18
27	RBCL vs. XO+LPS	0.2230	0.6117	-0.3887	0.2081	4	4	2.641	18
28	iRBCL vs. LPS	0.5800	0.5596	0.02046	0.2081	4	4	0.1390	18
29	iRBCL vs. XO+RBCL	0.5800	0.7800	-0.1999	0.2081	4	4	1.359	18
30	iRBCL vs. XO+iRBCL	0.5800	2.229	-1.649	0.2081	4	4	11.20	18
31	iRBCL vs. XO+LPS	0.5800	0.6117	-0.03162	0.2081	4	4	0.2149	18
32	LPS vs. XO+RBCL	0.5596	0.7800	-0.2204	0.2081	4	4	1.498	18
33	LPS vs. XO+iRBCL	0.5596	2.229	-1.669	0.2081	4	4	11.34	18

SourceDataforFigure5C — Edited

File | Sheet | Undo | Clipboard | Analysis | Interpret | Change | Draw | Write | Text | Export | Print | Send | LA | Help

Prism 8

ANOVA results | Multiple comparisons

Results
- Transform of Transpose of CCL5
- Mann-Whitney test of Transform of Transpose of CCL5
- Ordinary one-way ANOVA of Transform of Transpose of CC
- Transpose of CXCL9
- Col Stats of Transpose of CXCL9
- Transform of Transpose of CXCL9
- Mann-Whitney test of Transform of Transpose of CXCL9
- Ordinary one-way ANOVA of Transpose of CXCL9
- **Transpose of CCL2**
- Col Stats of Transpose of CCL2
- **Transform of Transpose of CCL2**
- Mann-Whitney test of Transform of Transpose of CCL2
- **Ordinary one-way ANOVA of Transform of Transpose of**
- *New Analysis...*

Graphs
- IL8
- IL8 line

Family
- **CCL2**
  - **Transpose**
    - **Transform**
      - **Ordinary one-way ANOVA**

Ordinary one-way ANOVA
Multiple comparisons

14	iRBCL vs. XO+LPS	-0.03162	-0.6930 to 0.6298	No	ns	>0.9999	B-F		
15	LPS vs. XO+RBCL	-0.2204	-0.8818 to 0.4410	No	ns	0.8911	C-D		
16	LPS vs. XO+iRBCL	-1.669	-2.331 to -1.008	Yes	****	<0.0001	C-E		
17	LPS vs. XO+LPS	-0.05208	-0.7135 to 0.6093	No	ns	0.9998	C-F		
18	XO+RBCL vs. XO+iRBCL	-1.449	-2.110 to -0.7874	Yes	****	<0.0001	D-E		
19	XO+RBCL vs. XO+LPS	0.1683	-0.4931 to 0.8298	No	ns	0.9622	D-F		
20	XO+iRBCL vs. XO+LPS	1.617	0.9558 to 2.279	Yes	****	<0.0001	E-F		
21									
22	**Test details**	**Mean 1**	**Mean 2**	**Mean Diff.**	**SE of diff.**	**n1**	**n2**	**q**	**DF**
23	RBCL vs. iRBCL	0.2230	0.5800	-0.3570	0.2081	4	4	2.426	18
24	RBCL vs. LPS	0.2230	0.5596	-0.3366	0.2081	4	4	2.287	18
25	RBCL vs. XO+RBCL	0.2230	0.7800	-0.5570	0.2081	4	4	3.785	18
26	RBCL vs. XO+iRBCL	0.2230	2.229	-2.006	0.2081	4	4	13.63	18
27	RBCL vs. XO+LPS	0.2230	0.6117	-0.3887	0.2081	4	4	2.641	18
28	iRBCL vs. LPS	0.5800	0.5596	0.02046	0.2081	4	4	0.1390	18
29	iRBCL vs. XO+RBCL	0.5800	0.7800	-0.1999	0.2081	4	4	1.359	18
30	iRBCL vs. XO+iRBCL	0.5800	2.229	-1.649	0.2081	4	4	11.20	18
31	iRBCL vs. XO+LPS	0.5800	0.6117	-0.03162	0.2081	4	4	0.2149	18
32	LPS vs. XO+RBCL	0.5596	0.7800	-0.2204	0.2081	4	4	1.498	18
33	LPS vs. XO+iRBCL	0.5596	2.229	-1.669	0.2081	4	4	11.34	18
34	LPS vs. XO+LPS	0.5596	0.6117	-0.05208	0.2081	4	4	0.3539	18
35	XO+RBCL vs. XO+iRBCL	0.7800	2.229	-1.449	0.2081	4	4	9.845	18
36	XO+RBCL vs. XO+LPS	0.7800	0.6117	0.1683	0.2081	4	4	1.144	18
37	XO+iRBCL vs. XO+LPS	2.229	0.6117	1.617	0.2081	4	4	10.99	18

Ordinary one-way ANOVA of Transform of Tra | Row 1, Column A
