# Supplementary material for: Malaria inflammation by xanthine oxidase‐produced reactive oxygen species
Source: EMBO Mol Med. 2019 Jul 2;11(8):e9903. doi: 10.15252/emmm.201809903 (PMC6685105; doi:10.15252/emmm.201809903)
Supplement: Supplementary file 9 — Source Data for Figure 6 [file EMMM-11-e9903-s007.pdf]

SourceDataforFigure6A

irbc cytokines

Table format: Grouped

		Group A	
		Data Set-A	
		A:Y1	A:Y2
1	TNF	-1.0168	1.4593
2	CXCL8	1.1584	-1.3612
3	IL18	1.5380	-1.1173
4	IL17A	1.0481	-1.1173
5	IL13	1.0481	-1.1173
6	IL10	1.7894	-1.1173
7	IL6	-2.1296	-1.8562
8	IL5	1.0481	-1.1173
9	IL4	1.0481	-1.1173
10	IL2	1.0481	-1.1173
11	IL1R1	-2.1809	1.4937
12	IL1B	2.2392	-1.7822
13	IL1A	-1.1755	-1.0373

SourceDataforFigure6A

Data Tables: irbc cytokines; xo + rbc cytokines; xo + irbcL cytokines

Graphs: irbc cytokines; xo + rbc cytokines; xo + irbcL cytokines

Table format: Grouped

		Group A: RBC Lysates + XO	
		A:Y1	A:Y2
1	TNF	15.459800	173.531700
2	CXCL8	11.617600	45.947400
3	IL18	6.843600	30.163600
4	IL17A	1.111700	0.512400
5	IL13	1.111700	0.512400
6	IL10	10.801100	6.349400
7	IL6	464.684700	1001.479400
8	IL5	1.111700	0.512400
9	IL4	1.111700	0.512400
10	IL2	1.111700	0.512400
11	IL1R1	1.887100	3.299800
12	IL1B	167.673800	843.592300
13	IL1A	96.698100	188.409200

Row 15, A: RBC Lysates + XO

SourceDataforFigure6A

Data Tables: irbc cytokines; xo + rbc cytokines; xo + irbcL cytokines

Graphs: irbc cytokines; xo + rbc cytokines; xo + irbcL cytokines

Family: xo + irbcL cytokines

Table format: Grouped

		Group A: iRBC Lysates + XO	
		A:Y1	A:Y2
1	TNF	20.653400	180.838300
2	CXCL8	12.092900	36.170200
3	IL18	6.705900	38.111400
4	IL17A	3.137100	0.651000
5	IL13	1.738300	0.651000
6	IL10	20.604800	5.660100
7	IL6	473.760500	795.094200
8	IL5	5.167900	0.651000
9	IL4	4.193600	0.651000
10	IL2	2.289700	0.651000
11	IL1R1	2.237400	2.071300
12	IL1B	221.929100	976.124400
13	IL1A	138.460500	148.704400

Row 18, A: iRBC Lysates + XO

SourceDataforFigure6B

Table format: Grouped

	Group A		Group B		Group C		Group D		Group E		Group F		Group G		Gro[illegible]
	Control		RBCL		iRBCL		LPS		XO		XO+RBCL		XO+IRBCL		XO+[illegible]
	A:Y1	A:Y2	B:Y1	B:Y2	C:Y1	C:Y2	D:Y1	D:Y2	E:Y1	E:Y2	F:Y1	F:Y2	G:Y1	G:Y2	H:Y1
1 Title	1.000000000	1.000000000	1.279188690	1.364228773	1.289736531	1.773013053	21.64030521	27.21391262	17.15675329	24.50901959	18.11457135	20.16325229	18.36657027	15.79911713	29.33624241

Data Tables: Relative abundance
Graphs: Relative abundance

Row CT, H: XO+LPS

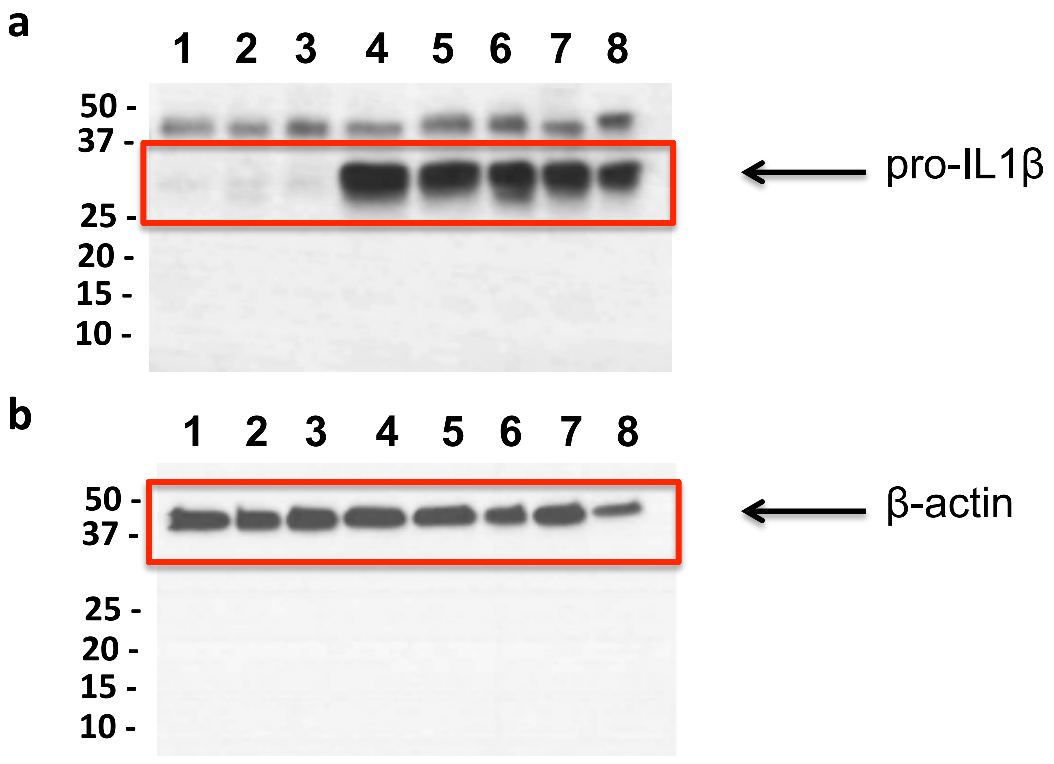

Source Data for Figure 6B: Addition of XO leads to the expression of pro-IL1β. Western blot was probed with (A) 1:1000 IL1β (rabbit, Abcam) antibody and (B) 1:1000 β-actin (Cell Signaling), followed by 1:2000 HRP-coupled secondary antibody (Cell Signaling) . Lanes are labeled as follows: (1) Media (2) RBCL (3) iRBCL (4) LPS (5) XO (6) XO+ rbcL (7) XO + iRBCL (8) XO+LPS. Red boxes indicate the lanes that were chosen for the bar graph (prism file) and the part of the blot chosen for the final figure.

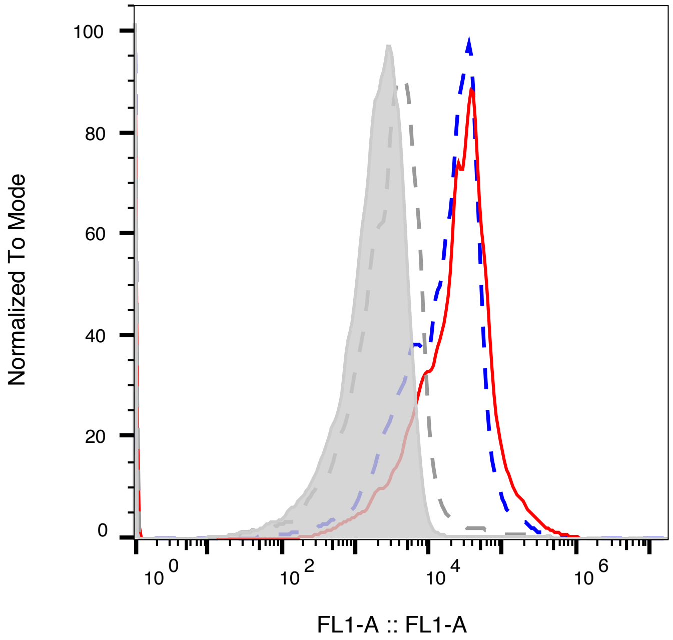

	Sample Name	Subset Name	Count
	C02 rbcL.fcs	Singlets	28828
	D02 xo rbcL.fcs	Singlets	7081
	C03 irbcL.fcs	Singlets	5498
	D03 xo irbcL.fcs	Singlets	9770

SourceDataforFigure6D

Table format: Grouped

		Group A	Group B	Group C
		negative siRNA	siRNA NLRP3-1	SiRNA NLRP3-2
		Y	Y	Y
1	RBCL	1.00	1.00	1.00
2	XO+RBCL	18.17	3.10	1.91
3	iRBCL	1.00	1.00	1.00
4	XO+iRBCL	207.26	31.56	39.53

Data Tables: donor 1 - fig6d; donor 2
Info: Project info 1; Project info 1
Graphs: donor 1 - fig6d; donor 2

Row 11, B: siRNA NLRP3-1

SourceDataforFigure6D

Table format: Grouped

	Group A: negative siRNA	Group B: siRNA NLRP3-1	Group C: SiRNA NLRP3-2
RBCL	1.00	1.00	1.00
XO+RBCL	1.00	1.00	1.00
iRBCL	1.00	1.00	1.00
XO+iRBCL	13.90	1.70	2.69

Data Tables: donor 1 - fig6d; donor 2

Graphs: donor 1 - fig6d; donor 2
